# Impact of variance components on reliability of absolute quantification using digital PCR

**DOI:** 10.1186/1471-2105-15-283

**Published:** 2014-08-22

**Authors:** Bart KM Jacobs, Els Goetghebeur, Lieven Clement

**Affiliations:** Department of Applied Mathematics, Computer Science and Statistics, Ghent University, Krijgslaan 281, S9, 9000 Ghent, Belgium

**Keywords:** Digital PCR, Absolute nucleic acid quantification, CNV, Variance component, Precision, Accuracy, Reliability, Experimental design, Polymerase chain reaction

## Abstract

**Background:**

Digital polymerase chain reaction (dPCR) is an increasingly popular technology for detecting and quantifying target nucleic acids. Its advertised strength is high precision absolute quantification without needing reference curves. The standard data analytic approach follows a seemingly straightforward theoretical framework but ignores sources of variation in the data generating process. These stem from both technical and biological factors, where we distinguish features that are 1) hard-wired in the equipment, 2) user-dependent and 3) provided by manufacturers but may be adapted by the user. The impact of the corresponding variance components on the accuracy and precision of target concentration estimators presented in the literature is studied through simulation.

**Results:**

We reveal how system-specific technical factors influence accuracy as well as precision of concentration estimates. We find that a well-chosen sample dilution level and modifiable settings such as the fluorescence cut-off for target copy detection have a substantial impact on reliability and can be adapted to the sample analysed in ways that matter. User-dependent technical variation, including pipette inaccuracy and specific sources of sample heterogeneity, leads to a steep increase in uncertainty of estimated concentrations. Users can discover this through replicate experiments and derived variance estimation. Finally, the detection performance can be improved by optimizing the fluorescence intensity cut point as suboptimal thresholds reduce the accuracy of concentration estimates considerably.

**Conclusions:**

Like any other technology, dPCR is subject to variation induced by natural perturbations, systematic settings as well as user-dependent protocols. Corresponding uncertainty may be controlled with an adapted experimental design. Our findings point to modifiable key sources of uncertainty that form an important starting point for the development of guidelines on dPCR design and data analysis with correct precision bounds. Besides clever choices of sample dilution levels, experiment-specific tuning of machine settings can greatly improve results. Well-chosen data-driven fluorescence intensity thresholds in particular result in major improvements in target presence detection. We call on manufacturers to provide sufficiently detailed output data that allows users to maximize the potential of the method in their setting and obtain high precision and accuracy for their experiments.

**Electronic supplementary material:**

The online version of this article (doi:10.1186/1471-2105-15-283) contains supplementary material, which is available to authorized users.

## Background

Advances in the field of polymerase chain reaction have enabled researchers to detect and quantify nucleic acids with increasing precision and accuracy. Until recently, real-time quantitative PCR was the gold standard for determining the concentration of a known target DNA or RNA sequence in a sample [1]. More than two decades ago, digital PCR was introduced as a potential alternative for detecting and quantifying nucleic acids [2]. The proof of concept followed a few years later [3]. Building on the necessary technological advances in the field of nanofluidics, commercially viable products were recently developed by 4 major players on the current market [4, 5].

Promising applications are found in food safety [6], forensic research, cancer diagnostics detection [7, 8], pathogen detection [9–11], rare allele detection [12], development of biomarkers [5] and sample preparation for next-generation sequencing [13] among others. The most popular applications so far are low copy number detection [14, 15] and copy number variation [16, 17].

Digital PCR uses microfluidic droplets or chips to divide a sample in hundreds, thousands or millions of tiny partitions. This is followed by a classical PCR amplification step. The endpoint fluorescence signal is used to classify partitions in two distinct groups: those that contain at least one target sequence and those that do not. From this, the percentage of partitions that are void of copies is obtained. The concentration of the target sequence can now be estimated as the number of copies per partition follows a Poisson distribution under regularity conditions.

Technical and biological factors that influence the concentration estimates have been studied extensively for quantitative PCR, which resulted in the formulation of guidelines for scientific authors [1]. Similar efforts to raise awareness and formulate guidelines for digital PCR have been very recently published [18]. Some of the relevant sources of variation largely remain to be explored however. One study examined the assumption that target copies are randomly distributed among partitions for a chip based system [19] while another focused on the presence of variance components in a droplet based system [20]. Experimental comparative studies between real-time quantitative PCR and digital PCR have found similar performance [9, 10, 21] while others are claiming digital PCR can measure smaller copy number variations than quantitative PCR [14, 16]. We study how the precision and accuracy of digital PCR results is affected by realistic levels of variation likely present in either system, and derive some guidelines for establishing more reliable estimates.

### dPCR Workflow

The digital PCR workflow allows for quick quantification of target sequences. The typical dPCR protocol reads as follows: (1) Extracting RNA or DNA from the biological sample. (2) Preparing the PCR master mix and including a quantity of extract. (3) Dividing the reaction mix over a large number of partitions. (4) Amplifying the target material present in the partitions over a selected number of amplification cycles and measuring the endpoint fluorescence. (5) Estimating the target concentration and quantifying the uncertainty on the estimates.

**Figure 1:**
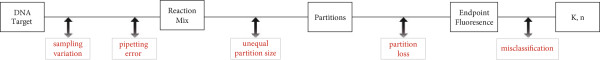
**Visualisation of the different steps in a typical digital PCR workflow.** Important variance components are included as arrows between the appropriate steps. The steps are: (1) extracting RNA or DNA from the biological sample, (2) preparing the PCR master mix and including a quantity of extract, (3) dividing the reaction mix over a large number of partitions (droplets or cells), (4) amplifying the target material present in the partitions over a selected number of amplification cycles and measuring the endpoint fluorescence and (5) estimating the target concentration and quantifying the uncertainty on the estimates. Variance components are (i) technical variation: sampling variation and pipette error, (ii) machine-specific variation: unequal partition size and possible partition loss, and (iii) possibly user-optimized (mis)classification of endpoint fluorescence.

Below, we discuss the different steps in the dPCR workflow together with their key sources of variation in the data production process as visualized in Figure 1 and summarized in Table 1.

**Table 1:** **Digital PCR Workflow**

Step	Output	Associated variation
1	Extracted DNA	Inhibition, overdilution, underdilution
2	Reaction mix	Pipette error, sample heterogeneity
3	Partitions	Loss of partitions, unequal partition size
4	Fluorescence signal	Loss of partitions, amplification efficiency
5	Estimated	Misclassification, model uncertainty,
	concentration	inaccurate partition size

A summary of the steps in digital PCR with associated variance components.

Digital PCR starts from an extracted DNA or RNA sample in a similar fashion as qPCR (step 1). Imperfections in the extract can lead to inhibited amplification of the target sequence. A dilution step may often be indicated.

Next, a predetermined amount of the (diluted) NA extract is mixed with the PCR Master Mix to create the reaction mix (step 2). The importance of transferring extracted NA accurately into the reaction mix is well recognized, yet small pipette errors are unavoidable for volumetric dilutions. These errors are typically much smaller for gravimetric dilutions although errors due to the balance and measurement method may still exist. Technical replicates of the same experiment may be prepared simultaneously, aiming for identical stochastic properties and sampling variation stemming from the Poisson process only. In practice they are subject to additional technical variation as a result of pipette error and sample heterogeneity among other technical factors. The magnitude of pipette error can be estimated from known systematic and random errors of pipettes.

From this moment on, the digital PCR workflow deviates from classic PCR. In the following dPCR step, each replicate sample is divided into a large number of partitions (step 3). Using microfluidics for instance, partitions are created which are either water-in-oil droplets or microchambers filled with reaction mix. The theoretical framework assumes that partitions are of equal size. In practice, droplets vary in size while chambers do not contain the exact same volume [19, 20]. In [19], the within-array coefficient of variation was estimated at around 10% for one of the chip-based systems.

The partitions are subsequently thermally amplified as in a classical PCR. Fluorescence levels are read for each partition and at the endpoint only in most systems (step 4). As in classical PCR, the experimenter is free to choose the number of amplification cycles. Most commercial machines include a default protocol with a fixed number of cycles.

Between partitioning and the fluorescence measurement, partitions may be lost in a random fashion for various reasons. In droplet-based systems, this might be induced by droplets that stick to the sides of the tube, clog the reader or coalesce together. In chip-based systems, spatial effects may play a role as adjacent chambers are more likely to be both lost, for example because of small hairs. Losses of about 30% seem normal for droplet-based systems [4, 12, 20].

**Figure 2:**
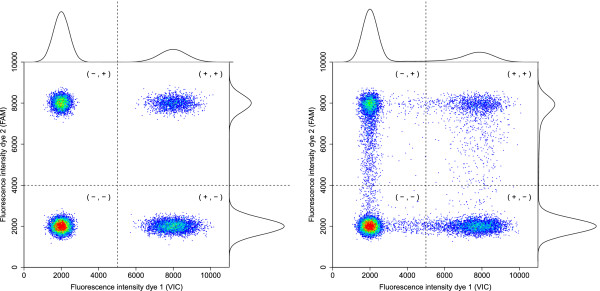
**Example of the endpoint fluorescence for two dyes.** In the left panel, the endpoint fluorescence of an artificial experiment without rain is shown, in the right panel the result of an artificial experiment with about 6% rain. For both dyes, the distribution is a mixture of two components, composed of output from both positive and negative partitions as shown with appropriate density functions on top and on the right of both graphs. An arbitrary threshold to separate both groups is added for each dye, dividing the area in four classification quadrants.

Raw fluorescence levels are finally transformed into a binary variable by applying a threshold obtained through data-analysis. Figure 2 illustrates the fluorescence pattern for an experiment with two dyes with arbitrary thresholds of 5000 and 4000. When end-point fluorescence exceeds this threshold, the partition is labelled positive and assumed to have at least one initial target copy. Meanwhile, a partition for which the fluorescence level does not reach the threshold is labelled negative and declared void of target copies. Current systems embed their own thresholds before labelling fluorescence values obtained at the end of the amplification cycle as signal of target presence rather than noise. Inhibition, slow starting reactions, primer depletion and other sources of technical and biological variation may result in misclassification for some partitions. The influence of inhibition on efficiency has been modelled for qPCR [22]. Increased inhibition has been shown to slow down the reaction considerably. In digital PCR, inhibitors or slow starting reactions may result in misclassification as partitions fail to reach the fluorescence threshold while still containing at least one initial target copy. Resulting false negatives hence reduce sensitivity for the detection of positive partitions.

On the other hand, the presence of highly homologous sequences and other contaminations may lead to non-specific binding of primers and can cause positive signals in the absence of a target sequence. These false positives correspondingly reduce specificity.

From [12, 14, 20, 23], we see that the amount of false positives for NTC’s (no template controls) is relatively small and often 0. Experiments on mutant DNA that include a wildtype reference sample provide similar results. The amount of false negatives may be much larger as we often see a (downward) bias [12, 14, 23].

Additionally, we noticed up to about 10% so called ‘rain’, which are partitions with an endpoint fluorescence measurement that can not be clearly classified as positive or negative based on the visible clusters. A visual example with about 6% rain can be seen in the right panel of Figure 2. The impact of changes in the threshold is confided to the labelling of observations in the rain as cluster members tend to be clearly positively or negatively labelled.

It was empirically verified in [23] that an increased number of amplification cycles tends to increase the percentage of amplified partitions. Consequently, the number of partitions that are difficult to classify, visualised as rain, are reduced and with it misclassification rates.

The choice of the threshold and subsequent labelling of the partitions is considered the first part of the data-analysis (step 5). Although the cut-off is somewhat arbitrarily and automatically chosen in most systems, it is possible for the researcher to set a user defined threshold.

Finally, the proportion of positive partitions is counted and the concentration of the target gene derived. Define *X* as the number of copies in a partition and *λ* as our parameter of interest: the expected number of target copies per partition. When the number of copies in a constant volume of a homogeneous mix is Poisson distributed [24, 25], we expect a proportion *p*=*P*(*X*=0)=*e*^-*λ*^ of partitions that is void of target copies. Let *K* be the number of partitions with a negative signal and *n*_*r*_ the total number of partitions for which results are returned. We can estimate *P*(*X*=0) by , the proportion of observed partitions void of target copies in our sample and we have:


Manufacturers of commercial systems provide an average partition size or volume *v*, in nanoliter say. The concentration estimate  of target copies per nanoliter then follows directly as . When the designated volume *v* is inaccurate, this leads to biased concentration estimates . This error is systematic and in addition to any random between-replicate variability on the average partition volume. In practice, small deviations exist. In [20], an overall average droplet size of 0.868 nL in 1122 droplets was observed, not significantly different from the estimate (*v*=0.89 nL) provided independently by the manufacturer. For a hypothetical sample with average partition size , the use of *v*=0.89 leads to a 2.5% downward bias of the concentration *θ*.

**Figure 3:**
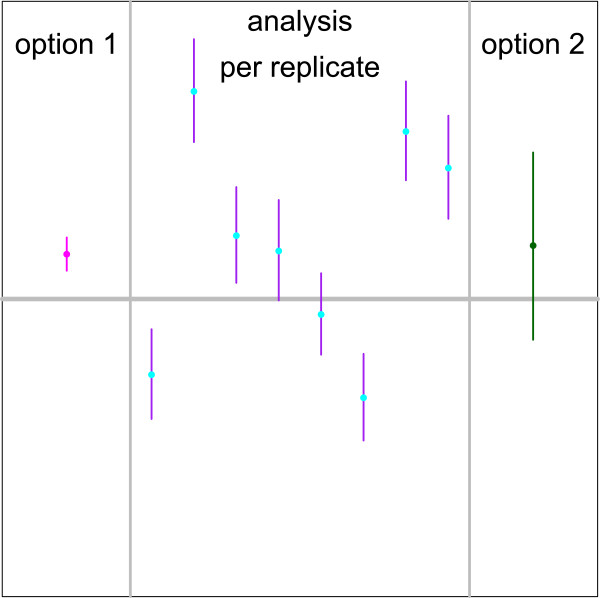
**Comparison of 95** ***%*** **confidence intervals on the target concentration for different estimation procedures.** The analysis per replicate shows typical 95% confidence intervals for single samples. Each replicate presents a random sample with expected concentration *λ*=1.25 target copies per partition and 5% pipette error. Option 1 on the left shows the 95% confidence interval calculated with a single sample method pooling the partitions of the 8 technical replicates before estimating the concentration and its variance with Poisson statistics. Option 2 on the right uses a replicate based method to estimate a 95% confidence interval based on the 8 individual replicates. Both the concentration and its variance were calculated using the empirical mean and variance of the concentration estimates of 8 independent replicates.

When technical replicates are available, results can be combined in two ways as shown in Figure 3. When the replicates are pooled, the formula above can be applied on the total number of partitions of all replicates to obtain a single concentration estimate. Alternatively, the concentration estimates can be calculated separately for all replicates and combined into a single number by taking the empirical average.

We simulate this digital PCR procedure while taking into account the different sources of variation to get a better understanding of the reliability of the proposed estimation procedures under the presence of these variation components. We quantify the influence of each source of variation on the accuracy and precision of the concentration estimates. Some sources of variation may be fixed by manufacturers such as equal partition sizes, but most factors that strongly influence both the accuracy and precision of concentration estimates are under experimental control or can be if the manufacturer allows it. This includes the number of amplification cycles, dilution levels of the sample and the classification method to determine the percentage of negative partitions. We study the relative importance and discuss the ability to improve results by well-chosen experimental set-ups and corresponding analyses.

Although the values in the simulation protocol below may not reflect the exact set-up of a specific machine, both the theoretical considerations and obtained results are relevant for all systems including, but not limited to, all commercial systems currently in use [4].

## Methods

Below, we detail our simulation set-up presenting plausible departures from the theoretical model described above. From each generated dataset, we estimate the number of target copies in the sample and derive the concentration of the original dilution relying on the assumptions of the simple working model, which may ignore components of variation in the data-generating model. For each set-up 1000 simulations are run to get stable results.

### Data-generating model

We generate data according to the steps described in Table 1 and Figure 1 and evaluate multiple scenarios that combine different sources of variation in different simulations.

In step 1, we simulate the process for several orders of magnitude of NA concentration reflecting dilution levels used in practice. We therefore let the expected number of target copies per partition *λ* range from 0.0001 (1 in 10 000) up to 5.

In step 2, we add random pipette errors to our simulations. Pipette error results in a small deviation of the expected target sequence concentration in the reaction mix from the original concentration in the dilution. We simulate random pipette errors, without the non-stochastic systematic pipette error. Our pipetted volume is normally distributed with a coefficient of variation of 0% to 10%. These deviations are based on the maximum allowed pipette error guidelines (ISO 8655-7:2005) combined with possible heterogeneity of the original dilution. All other sources of between-replicate technical variability, including between-replicate variation of partition size, are lumped in what we generally refer to as pipette error. In [20], a between-well coefficient of variation of 2.8% was found based on 16 wells in a droplet based system. In [19], a between-array coefficient of variation of 4.9% can be crudely estimated based on 2 arrays for a chip based system. In each simulation run, we consider 8 technical replicates from the same biological sample. Consequently, they keep technical variability as a direct result of the pipette error described above among other sources of technical variation. Hence, our simulations can be interpreted as repeated experiments under the same conditions performed by the same experimenter with the same pipette.

In step 3, we study the difference in partition size, or equivalently in partition volume between the different partitions within a replicate. We assume that sizes vary independently and follow the same distribution in each replicate. We model this size as a log-normal distribution with parameters *μ*=0 and *σ*=0.1 which is approximately equal to a normal distribution with a coefficient of variation of 10*%*. The expected number of target copies in a partition is modelled to be proportional to the size of the partition.

In step 4, the fluorescence levels of all partitions are measured. During this process, some partitions may be lost for unknown reasons. We assume random loss implying missingness is completely at random with respect to outcome. If this is true, lost partitions are as likely to return a positive signal as returned partitions. This is equivalent to an experiment in which fewer partitions were created and none lost.

We did our simulations for a system with 20 000 partitions. To examine the influence of random partition loss, we varied the number of returned partitions between replicates and simulations independently with an expected value and standard deviation of approximately 14 000 and 1800 respectively, as in [20].

In Additional file 1, we derive precision estimates. For the large number of partitions currently generated, the anticipated loss imply but a slight loss in precision amounting to a negligible source of variation.

In step 5, the fluorescence level of each partition is transformed into a binary 0/1-signal after applying a somewhat arbitrarily chosen threshold based on the data. In simple experiments, the positive and negative partitions can be easily separated by the observed fluorescence and as such the number and proportion of partitions with a positive signal can be determined with minimal error. This is shown in Figure 2 on the left.

In our simulations, we look at a fixed underlying misclassification probability without appointing a specific cause for this misclassification. This allows us to study the effect of the misclassification itself without putting much emphasis on the reason behind it. Every partition containing a target copy has a given conditional probability to return a negative signal (the expected false negative rate, 1-sensitivity) while every partition without target copy has a given conditional probability to return a positive signal (the expected false positive rate, 1-specificity). We assess the following false positive-false negative (FPR,FNR) combinations: (0.01%; 0%), (0.01%; 0.2%), (0.01%; 1%), (0.01%; 5%), (0.01%; 20%), (0%; 1%), (0.1%; 1%), (1%; 1%).

This set-up allows for a broad range for the false negative rate under a fixed specificity of 99.99% as experiments tend to be more vulnerable to not detecting true positive partitions. The influence of the false positive rate is limited to smaller deviations with a specificity at least 99% in each simulation under a fixed realistic sensitivity of 99%.

We include the different variance components both in parallel simulations (each separately) as well as sequentially. In the latter case, sources were added in the following order: random partition loss, pipette error, unequal partition size, misclassification.

### Generated parameter estimates

We calculate a concentration estimate , the bias , the associated asymptotic variance and a 95% confidence interval for each of the 8 replicates of each experiment (see Additional file 1 for the calculation and derivation of the asymptotic variance and associated confidence interval). We chose 8 replicates as the number of reactions that can run simultaneously by the different systems is typically a multiple of 8. Most systems use 12×8=96 well plates for preparing the reaction mix.

Additionally, the results from the 8 simulations are combined in two different ways.

The first option that we consider, is pooling the replicates as if it were one sample with  partitions. The estimate, its bias, asymptotic variance and confidence interval are calculated, again as if it was one sample. This method is still used in the literature and stems from initial papers on digital PCR which deal with small numbers of partitions and pooled repeated experiments to obtain the required accuracy [12, 16, 21, 26].

As a second option, we study the variation between the replicates by assuming the 8 estimates stem from independent results, which may show some between-replicate variation. We calculate the empirical average and empirical variance of the 8 separate estimates and derive a 95% confidence interval under the assumption that the estimates of different replicates follow a normal distribution. This is a realistic assumption since the number of target copies in each replicate follows an approximately normal distribution (Poisson distribution) for a constant volume under the theoretical assumptions [24, 25].

## Results and discussion

In what follows, we discuss how the impact of each commonly encountered source of variation in the data generating process can be quantified. These results form a starting point for optimizing tuning parameters of the method and guide the experimental design.

### Optimal concentration and loss of partitions

The first simulated scenario follows the simple theoretical model which includes random partition loss as the only source of variation. When the loss is completely at random, the precision of the concentration estimate solely depends on the model-based variability for a given number of partitions. This describes the best-case scenario where random sampling variation as described by the Poisson process is the only source of variation as in [26].

**Figure 4:**
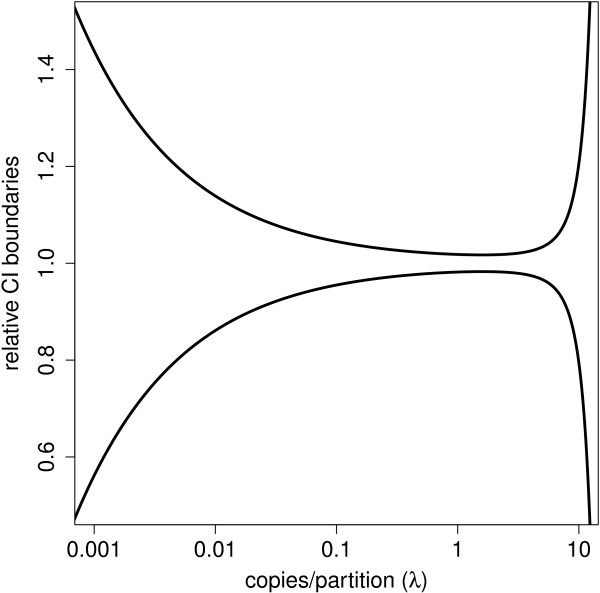
**Theoretical confidence interval limits of the estimated concentration relative to the true concentration.** The theoretical limits of a 95% confidence interval of the concentration estimate  divided by the true concentration *λ* as a function of this concentration (in copies per partition) for 20 000 analysed partitions. The limits are shown relative to the true concentration such that the precision of different dilutions of the same sample can be assessed on the same scale. Although the application can be used for concentration ranges of up to 5 orders of magnitude, very precise estimates are theoretically only possible for about 2 orders of magnitude.

Since model-based variability is driven by the target DNA concentration in the sample, an optimal proportion of positive partitions leads to the most precise estimates. This can be achieved for an average of 1.59 target copies per partition (see Additional file 1). Figure 4 shows theoretical relative boundaries of the confidence interval for any given concentration as a function of the true generated *λ*. The most narrow intervals close to the optimal concentration grow into much larger intervals as boundary conditions are reached with few negative or positive partitions.

Our simulations confirmed this trend. Estimators are unbiased while the variance and thus the width of the 95% confidence interval decreases for increasing concentration until an optimum is reached around 1.5 target copies per partition. From 1.5 onwards the variance and CI width start to increase again. A more detailed figure is shown in Additional file 2.

A random loss of partitions translates into a small decrease of precision. We simulated samples under theoretical conditions with on average 20 000 created partitions. Randomly removing approximately 30% of the partitions increased the estimated asymptotic relative standard deviations under the Poisson assumption by on average about 20%. The standard deviation based on estimates from 1000 simulation runs was 44% higher. These numbers are consistent with theoretical expectations and small compared to other sources of variation discussed below.

### Pipette error leads to underestimation of the variation

In a next group of simulations, we study the impact of additional variation between technical replicates induced by pipette errors, sample heterogeneity and between-replicate variation in average partition size. This generates slightly different amounts of target NA and varying volumes in each replicate. We expect the number of target copies in a constant volume to vary between replicates due to pipette errors and sample heterogeneity on top of the inherent Poisson variability.

**Figure 5:**
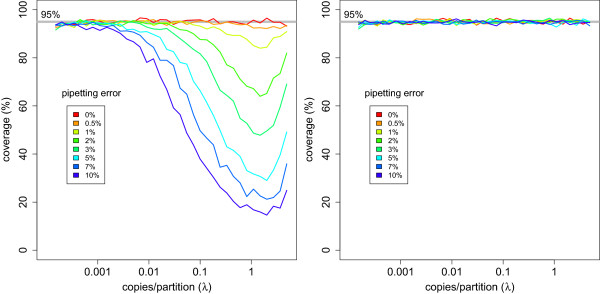
**Coverage of 95% confidence intervals of target concentration in the presence of pipette error.** For a given concentration *λ*, the coverage was calculated as the ratio of the number of confidence intervals out of 1000 simulations that contain the true concentration *λ* divided by the total number of confidence intervals calculated (1000). The left panel shows results for confidence intervals calculated using a single sample method after pooling the partitions of the 8 technical replicates before estimating the concentration and its variance with Poisson statistics. The right panel shows results for confidence intervals calculated using a replicate based method. The concentration and its variance were calculated using the empirical mean and variance of the concentration estimates of 8 independent replicates. The pooled method shows a dramatic loss of coverage while the replicate based method shows correct coverage.

Since the theoretical model does not account for variation between replicates (see Additional file 1), it underestimates the variance and overestimates the coverage of the confidence intervals as illustrated in Figure 5. This is most problematic for the concentrations where the Poisson model yields the smallest confidence intervals. The theoretical model assumes that the model variance is lower close to 1.59 copies/partition, but the technical variance as a result of pipette error is similar for most concentrations. Both Poisson and technical variability contribute to the total variation. The technical variation appears to dominate the Poisson variation for concentrations close to the optimum under typical experimental conditions. Consequently, the precision decreases considerably.

The extra variance can not be estimated from a single reaction but replicates allow for realistic estimates on the precision of the results. The replicate based variance estimator has the advantages of being unbiased and capturing the total variance. The resulting intervals do show a correct coverage, as illustrated on the right panel of Figure 5. Naive pooling of partitions from different replicates seems to increase precision, but in fact it dramatically underestimates the variance and it must be avoided. In Figure 3, we see how the small confidence interval resulting from pooling (option 1) may not contain the true parameter value. The replicate based variance estimator (option 2) captures the variance both within (purple lines) and between (blue dots) replicates.

Since the width of a replicate based confidence interval is a decreasing function of the number of replicates *m*, a large number of replicates is preferred such that the confidence interval is as small as possible. Conversely, we would like to keep *m* small for cost-efficiency. In Table 2, we calculated the width of the confidence intervals relative to the standard deviation and added the expected reduction of the width as a result of every additional technical replicate. It can be clearly observed that 4 or more technical replicates would be preferred to get a decent confidence interval, while more than 8 technical replicates does not improve the results considerably. Additionally, biological repeats are essential in most applications to capture any existing between-sample variation. In the latter case, at least 4 technical replicates are advised for each biological repeat.

**Table 2:** **Width of a replicate based confidence interval**

Replicates ( ***m*** )	Width	Improvement
2	1.000	
3	0.276	72.35%
4	0.177	35.94%
5	0.138	21.97%
6	0.117	15.48%
7	0.103	11.87%
8	0.093	9.60%
9	0.086	8.06%
10	0.080	6.94%
11	0.075	6.09%
12	0.071	5.42%

The relative width of a confidence interval (proportional to ) for a constant standard deviation is a function of the number of technical replicates and the t-quantile. The rightmost column gives the improvement (percentage decrease in width) when increasing the number of technical replicates with one. Note, that in this table we only consider uncertainty due to technical variability and that reducing technical variability does not eliminate biological variability. Hence, in experiments for comparing nucleic acid content across biological conditions an appropriate number of biological repeats, each with technical replicates, will always be required.

### Unequal partition size leads to downward bias

In this section, we assume that the size for a given partition is no longer constant, but varies randomly, independent of any other variable. We assume that there is no intra- nor inter-run effect and thus the size follows the same distribution between replicates.

**Figure 6:**
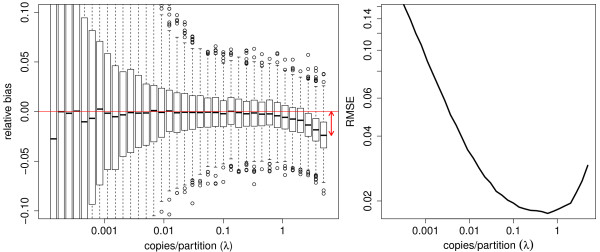
**Relative bias and RMSE of the target concentration estimates in the presence of unequal partition size.** When droplets or chips do not contain the same volume, bias is introduced. In the left panel, a boxplot shows relative estimates for 1000 simulated experiments at given concentration *λ* (copies/partition). The relative bias is calculated using a replicate based method as  for 1000 simulated experiments of 8 replicates. High concentrations show a downward bias. In the right panel, the associated root mean squared error  is shown, estimated as the square root of the sum of the relative variance and squared relative bias for *S*=1000 simulated experiments of 8 replicates. For a given concentration *λ*, this combines the errors as a result of the variance and the bias in a single number based on the results of 1000 simulated experiments. The best combination of accuracy and precision is achieved when the function hits its minimum.

Theoretical derivations (see Additional file 1) indicate that underestimation is to be expected especially for samples with high concentration. In Figure 6, relative estimates are summarized for normally distributed sizes with a relative deviation of 10%. The estimators show a systematic downward bias that is negligible for small concentrations and maximally 2.5% for the highest concentration in this set-up. The variance is similar to that of the equivalent simulation with constant partition size although slightly lower for higher concentrations as it is directly related to the estimated concentration, which is in turn underestimated.

We use the , estimated as the square root of the sum of the variance and the squared bias, to take both the variance and the bias into account and give accuracy and precision an equal weight. In the right panel of Figure 6, the RMSE reaches its minimum around 0.5 copies per partition with decent performance between 0.1 and 2 target copies per partition for this specific set-up.

We note that the influence of unequal partition size is limited and can be easily avoided by diluting a sample. Since this is a fixed machine setting, manufacturers should guarantee that sizes of the partitions created by or present in their products are somewhat comparable.

### Misclassification of target presence leads to bias

Next, we study the misclassification. We assess the following false positive-false negative (FPR,FNR) combinations discussed above: (0.01%; 0%), (0.01%; 0.2%), (0.01%; 1%), (0.01%; 5%), (0.01%; 20%), (0%; 1%), (0.1%; 1%), (1%; 1%).

In the first data-generating model, misclassification is the only source of variation while in the other model all previously discussed sources of variation are included in addition to the misclassification.

**Figure 7:**
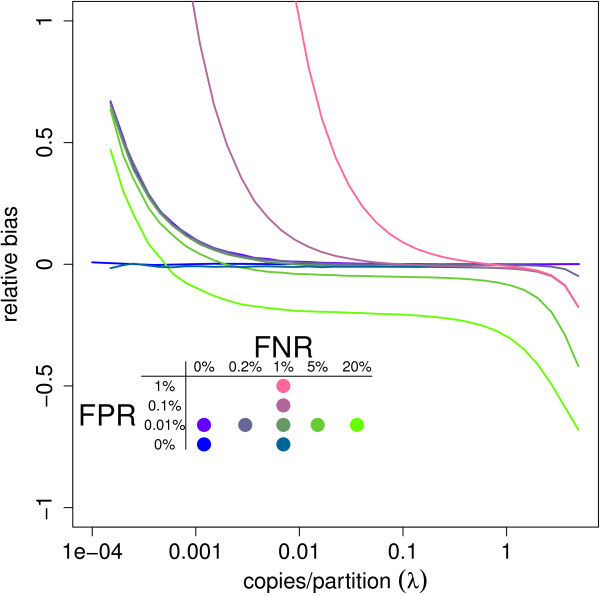
**Relative bias of the target concentration estimates under theoretical assumptions and misclassification.** The relative bias is calculated as  for 1000 simulated experiments of 8 replicates without any additional sources of variation. As results are relative to the true concentration *λ*, the precision of different dilutions of the same sample can be assessed on the same scale. Results were plotted for different misclassification probabilities (FPR, FNR) with FPR = false positive rate = 1-specificity and FNR = false negative rate = 1-sensitivity. False positives have considerable influence on the estimates for low concentrations, while false negatives substantially influence the results for highly concentrated samples.

In Figure 7, we see the results for the bias for simulations without other variance components. Misclassification creates bias since false negatives lead to underestimation and false positives to overestimation of *λ*. A few false positives already have a big impact on samples with few target copies, while increasing false negatives especially has a very high impact on samples with a higher concentration.

Since the variance is proportional to the rate itself, its estimate decreases as false negatives increase and increases with more false positives. The false negative (positive) risk has a bigger impact with higher (lower) concentrations.

Interestingly, every line in Figure 7 that includes both sources of misclassification crosses 0 at some point. This means that for a given combination of false positive and false negative risks, we can find a dilution for which the estimator is unbiased.

Based on a dilution series with enough points, one of the patterns in Figure 7 may be recognized when plotting the estimated concentration against the dilution rate. This is similar to the linearity and precision plots that are already used in the literature [20] and may help the user to assess possible bias.

Conversely, we can derive the ratio of false negatives over false positives that results in unbiased estimates for a given concentration (See Additional file 1). This too has practical relevance. The threshold to discriminate between positive and negative partitions can be manually adapted to allow more positive partitions when false negatives may be most problematic. This is assumingly the case in most experiments. The threshold should be increased to allow less positive partitions when false positives are expected to dominate the estimation error. This would be especially useful in experiments with small concentrations or that focus on detection.

Users may choose to add a two-step procedure to improve the threshold in their protocol. In the first step, an initial concentration estimate can be obtained with the standard threshold. In the second step, the threshold may be changed based on the concentration estimate obtained in the first step and optional prior information on the expected misclassification rates.

**Figure 8:**
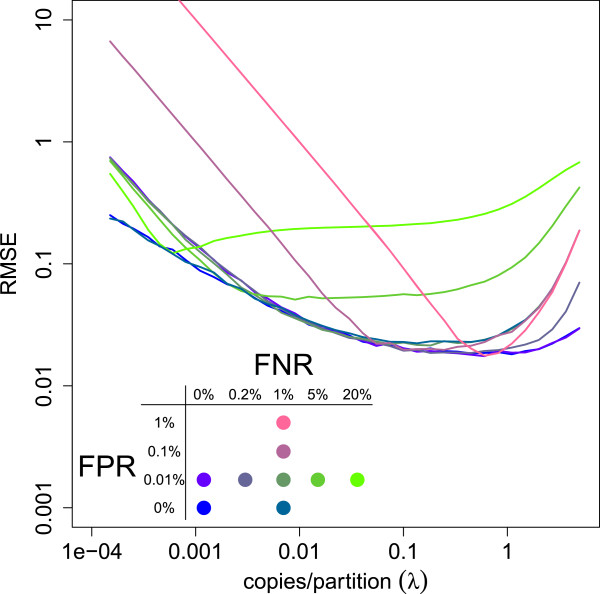
**Relative RMSE of the target concentration estimates under realistic assumptions and misclassification.** The  is estimated as the square root of the sum of the relative variance and squared relative bias for *S*=1000 simulated experiments of 8 replicates. As results are relative to the true concentration *λ*, the precision of different dilutions of the same sample can be assessed on the same scale. Results were plotted for different misclassification probabilities (FPR, FNR) with FPR = false positive rate = 1-specificity and FNR = false negative rate = 1-sensitivity and in the presence of pipette error and unequal partition size. When misclassification is limited, a relatively wide window of dilution exists in which high accuracy and precision can be achieved.

**Figure 9:**
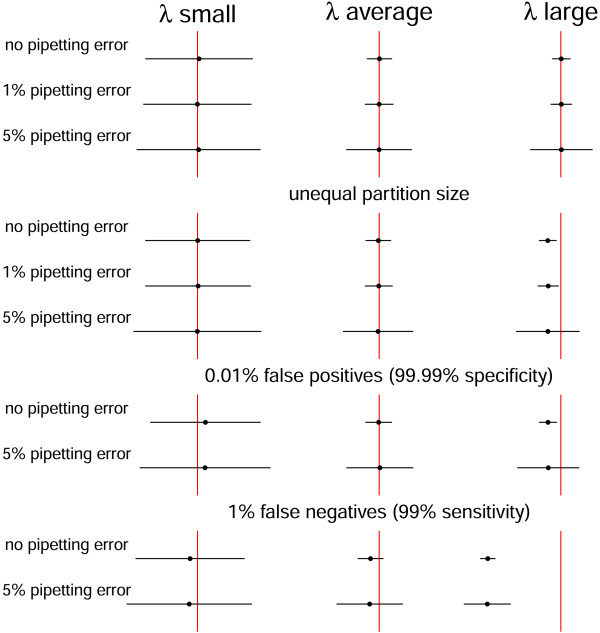
**Influence of different sources of variation on the width and location of confidence intervals for the target concentration.** The influence of the different sources of variation is shown on the width of 95% confidence intervals calculated with the replicate based method for *λ* small (≈0.009), average (≈0.18) and large (≈3.65). Unequal partition size is included in the misclassification examples.

The bias as a result of misclassification dwarfs any possible bias that may be present due to unequal partition sizes if the partition sizes are somewhat similar. In Figure 8, we see that the lines with respect to high misclassification rise quickly while the bias as a consequence of unequal partition size is hardly visible as it is a small part of the rise of the curves for high concentrations. This is more clearly visible in Figure 9. We see that a small, realistic false negative rate of 1% leads to increasing bias for increasing *λ* while the influence of unequal partition sizes is limited for average to small concentrations.

The optimal window that gives a concentration estimate  with the highest precision is strongly dependent on the proportion false positives and false negatives. In Figure 8, all sources of variation discussed are combined and the relative RMSE as a combined measure of the bias and variance is plotted against the true concentration. Larger numbers of false positives and false negatives lead to smaller windows with optimal estimates. When there is limited misclassification, relatively large windows with both accurate and precise estimates exist.

Note that one strategy to influence and reduce the misclassification rates in practice may involve changing the number of amplification cycles. Additionally, reference materials, qPCR and no-template controls can help to assess the vulnerability of a sample to misclassification and may allow for a crude estimate of expected misclassification rates.

### Non-stochastic errors

While our simulations focus on stochastic settings, systematic errors may be present as well.

Systematic pipette error, for instance, introduces underestimated (overestimated) concentration estimates and data-analytic methods cannot correct for a lack (excess) of NA material in the reaction mix. Systematic volumetric pipette error can however be estimated with gravimetric procedures and be reduced by recalibrating pipettes regularly.

The partition volume *v* supplied by the manufacturer enters in the denominator of the final concentration estimate as a constant assumed to be correct. When the actual mean partition volume deviates from *v*, systematic bias is added when the concentration *θ* is reported in copies per nanoliter, *λ*/*v*. We demonstrated how small deviations in partition volume within a replicate create only limited bias for high concentrations. Systematic deviations of the average partition size from *v* can create a much larger bias. The small, non-significant difference of 2.5% found in [20] for instance induces more bias than 10% within replicate variation. It is therefore essential that manufacturers invest in accurate partition volume estimates.

### Combining sources of variation

In practice, all of the aforementioned sources of variation are present in experiments in one way or another. It is not feasible to describe all combinations jointly. Additional file 3 provides the R-code used in this article and enables the user to simulate the outcome of an experiment with specific settings for each source of variation discussed above. Additional file 4 consists of an interactive tool embedded in a mini-website that allows researchers to study results that can be expected from a useful range of combinations of these sources of variation. The tool provides valuable information on the joint effect of different realistic sources of variation present in most experiments. Note, that our results can guide dPCR users to optimise their experiments with respect to signal bias or RMSE. This is useful as our results show that a well-chosen threshold (rightmost drop-down menu) combined with an optimal sample dilution (x-axis) can improve accuracy and precision considerably.

## Conclusions

We studied the influence of several sources of variation on estimators produced by digital PCR. We showed how some have higher impact than others and found certain background conditions to be more vulnerable to this than others. This impact may stay hidden to the naive user who could take away suboptimal results with a false sense of precision, accuracy and reliability.

A first source of variance is technical variation, which includes pipette error. Although careful sample preparation can keep this error relatively small, it is unavoidable and reduces precision. This can not be captured with a single replicate and previously published asymptotic confidence intervals. Replicates allow this source of variation to be included in the data-analytic process and provide correct precision estimates.

Unequal partition size reduces the accuracy for highly concentrated samples. Since this source of variation is dependent on the machine itself, it is one of the priorities for manufacturers to optimize it and keep the variation between partitions small. We have shown that the bias is small for realistic limited deviations and can be neglected when the concentration is not close to the upper limit. Users are advised to avoid strongly concentrated samples and dilute samples when necessary.

The fluorescence threshold chosen for target detection drives the misclassification rates and has a high impact on the results, reducing accuracy. Samples with few target copies and experiments with a very high concentration of target nucleic acid are especially vulnerable. In the former case, the focus may usefully shift to detection rather than quantification while in the latter, dilutions or qPCR may be advised. Misclassification to some extent is unavoidable, but the informed user can do a lot to reduce it.

The underlying continuous distribution of the fluorescence is a mixture distribution composed of output from both positive and negative partitions. These two parts may be partially overlapping as a result of biological factors such as inhibition, contamination or primer depletion. The choice of the threshold results in corresponding false positive and false negative rates. The optimal trade-off naturally depends on the concentration of target copies in the sample. When the software allows it, users can calibrate the threshold to reflect expected misclassification rates of their application and get more accurate results.

Additionally, dilution series may help to determine the concentration where the variance-bias trade-off is lowest and the measurement reflects the best combination of accuracy and precision. This is especially useful at high concentrations when the focus is on accurate quantification. Users can achieve this by comparing precision and linearity plots to the patterns in Figure 7 to picture the bias, while an estimate of the standard deviation follows from correctly analysed replicate experiments.

Since we identified misclassification as the major bottleneck that induces the largest accuracy drop, methods to optimise classification and accuracy are promising topics of future research.

Finally, it is worth emphasizing that our results have focussed on technical replicates involving variation in results generated by machine settings and human handling of a given biological sample. It is essential to acknowledge sources of variation such as systematic pipette error and correct for them when necessary. As for any biological measurement, additional sampling variation may be present in many experiments at several levels. This happens quite independently of the technology and is discussed widely in the literature [1]. A thoughtful protocol to correct also for this source of variation should be generally considered in addition to the specific digital PCR protocol.

Digital PCR is a promising tool for high precision estimation. We showed how several sources of variation can influence results and can be accommodated with the correct knowledge such that accurate and precise concentration estimates remain possible. Our findings indicate that reliability can be increased by well-chosen sample preparation and machine settings. Machine calibration in theory allows the researcher to adapt the technology to yield results optimized for each specific setting. While it is of course essential to provide default settings to simplify the process for the users, it is at least as important that manufacturers provide detailed output to facilitate personalized treatment and thus enhance the quality of their results.

## Electronic supplementary material

Additional file 1: **Mathematical derivations.** This PDF file includes mathematical derivations on the theoretical confidence interval, optimization of the theoretical precision, decomposition of the variance in the presence of pipette error, a model for unequal partition sizes and theoretical methods to optimize the threshold in the presence of misclassification. (PDF 165 KB)

Additional file 2: **Additional figures.** This PDF file includes two additional figures on the width of confidence intervals. (PDF 117 KB)

Additional file 3: **R-script.** Users can simulate their own experiments with this code as well as reproduce all the numerical results discussed above. (ZIP 4 KB)

Additional file 4: **Interactive tool.** In this mini-website, we provide an interactive tool to study the influence of specific sources of variation on the performance of the concentration estimators. This can serve as a guide when designing an experiment. All results are relative to the true concentration and based on 1000 simulations with 8 technical replicates. (ZIP 17 MB)
