# Supplementary material for: Impact of variance components on reliability of absolute quantification using digital PCR
Source: BMC Bioinformatics. 2014 Aug 22;15(1):283. doi: 10.1186/1471-2105-15-283 (PMC4261249; doi:10.1186/1471-2105-15-283)
Supplement: Supplementary file 1 — Additional file 1: Mathematical derivations. This PDF file includes mathematical derivations on the theoretical confidence interval, optimization of the theoretical precision, decomposition of the variance in the presence of pipette error, a model for unequal partition sizes and theoretical methods to optimize the threshold in the presence of misclassification. (PDF 165 KB) [file 12859_2014_6687_MOESM1_ESM.pdf]

Impact of variance components on reliability of absolute quantification using digital PCR - Additional File 1: Mathematical Derivations

Bart KM Jacobs*, Els Goetghebeur and Lieven Clement*

*Correspondence:
BartKM.Jacobs@UGent.be,
Lieven.Clement@UGent.be
Department of Applied Mathematics, Computer Science and Statistics, Ghent University Krijgslaan 281, S9, 9000 Ghent, Belgium

In what follows, we will consider experiments with given number n_r of retained partitions. We assume n_r is fixed and known. All results are derived conditional on n_r. To improve readability, we will omit this in the notation.

Derivation of the confidence interval

Under regularity assumptions, the number of target copies X in a constant volume follows a Poisson distribution $Pois(\lambda)$ [1], [2]. Since $E[X] = \lambda$, this can be defined as the expected number of copies per partition if we assume that the partition volume is constant. Define K the number of partitions that return a negative signal out of the n_r retained partitions. The probability that a partition did not contain an initial target copy $p = P(X = 0) = \exp(-\lambda)$ can estimated as $\frac{K}{n_r}$ and λ can be estimated as

$$\hat{\lambda} = -\log\left(\frac{K}{n_r}\right)$$

It is shown in [1] that this is the maximum likelihood estimator (MLE) under the following model: Let Y be a binary indicator that is $Y = 1$ if a given partition does not contain a target copy $(X = 0)$ and $Y = 0$ if it does contain a target copy $(X > 0)$. Under the assumptions that the number of copies in a constant volume is Poisson distributed and all partitions have the same probability $1 - p$ to contain a target copy, the number of partitions with a negative signal K equals:

$$K = \sum_{i=1}^{n_r} Y_i \text{ with } Y_i \sim B(1; p) \Rightarrow K \sim B(n_r; p) \Rightarrow K \sim B\left(n_r; e^{-\lambda}\right)$$

where B denotes the binomial distribution. Using maximum likelihood theory, we can immediately obtain an estimate for the variance by inverting the Fisher information matrix:

$$Var\left(\hat{\lambda}\right) = \frac{1 - e^{-\lambda}}{n_r e^{-\lambda}}$$

For more details, we refer to [1].
A plug-in estimator for the variance of the number of target copies per partition is obtained after replacing $e^{-\lambda}$ by its estimator $\frac{K}{n_r}$: $Var\left(\hat{\lambda}\right) = \frac{n_r - K}{K n_r}$.

Since $\hat{\lambda}$ is an MLE, the asymptotic 95% confidence interval can be calculated as:

$$\left[\hat{\lambda} - 1.96\sqrt{\frac{n_r - K}{Kn_r}}; \hat{\lambda} + 1.96\sqrt{\frac{n_r - K}{Kn_r}}\right]$$

The confidence interval above gives highly similar results to the one derived in [3]. But, it is more accurate close to the right border (few negative partitions).

Optimization of the theoretical precision

The width of the confidence interval of the concentration is dependent on the asymptotic variance, which is a function of λ, the number of copies per partition. As such, it can be minimized with respect to the parameter λ. We aim to find the concentration such that the relative variance per copy number $Var\left(\frac{\hat{\lambda}}{\lambda}\right)$ is minimal. This is equivalent to optimizing the dilution for most accurate measurements. We minimize the following loss function:

$$f(\lambda) = Var\left(\frac{\hat{\lambda}}{\lambda}\right) = \frac{1}{\lambda^2}\frac{1 - e^{-\lambda}}{n_r e^{-\lambda}} = \frac{e^{\lambda} - 1}{n_r \lambda^2}$$

After derivation, we get:

$$\frac{df(\lambda)}{d\lambda} = \frac{n_r\lambda^2 e^{\lambda} - 2n_r\lambda e^{\lambda} + 2n_r\lambda}{n_r^2\lambda^4} = \frac{\lambda e^{\lambda} - 2e^{\lambda} + 2}{n_r\lambda^3} = \frac{e^{\lambda}}{n_r\lambda^3}\left(\lambda - 2 + 2e^{-\lambda}\right)$$

Since $\lambda > 0$, we solve $\lambda - 2 + 2e^{-\lambda} = 0$ for λ. This has no closed-form solution, but can easily numerically approximated. We get $\lambda = 1.59$ which means the most precise estimates can be obtained for 1.59 copies per partition. Note, that the same result can be found by maximizing the Fisher information of $log(\lambda)$ [1].

Decomposition of the variance in the presence of pipette error

Suppose we examine a sample and we prepare a reaction mix in several replicates to determine the concentration of a target gene. We define θ as the concentration of target nucleic acids (NA) in our raw material. When preparing the technical replicates, we mix the purified NA with appropriate primers, probes and other material necessary for the PCR reaction. Under the assumptions of the Poisson model, the concentration of each replicate, c_k, is drawn from a $Poisson(\eta_k)$ distribution with $\eta_k = \eta = \theta\frac{V^p}{V^r}$, V^p the pipetted volume and V^r the volume of each reaction mix. In practice, pipette errors and sample heterogeneity occur. Hence, we have to redefine $\eta_k = \theta\frac{V_k^p}{V_k^r}$ as the expected concentration in each replicate given the actual pipetted volume, V_k^p, and the actual volume of the reaction mix, V_k^r. We will thus estimate $\hat{\theta}_k = \hat{\eta}_k\frac{V^r}{V^p}$ for each replicate k. When technical replicates are prepared by the same operator and/or pipette, systematic pipette error can lead to bias: $E[\eta_k] = \eta' \neq \eta$ and $E[\hat{\theta}_k] \neq \theta$. Users can assess and correct for this in a controlled laboratory environment.

Additional variability cannot be avoided as every pipetting step introduces random error. We have as a general property:

$$Var\left(\hat{\theta}_k\right) = \underbrace{E\left[Var_k\left(\hat{\theta}_k\right)\right]}_{A} + \underbrace{Var\left[E_k\left(\hat{\theta}_k\right)\right]}_{B}$$

Only for an ideal pipette, $E_k\left(\hat{\eta}_k\right) = \eta_k = \eta$ and $E_k\left(\hat{\theta}_k\right) = \theta_k = \theta$ so term B equals 0 and we can use the asymptotic variance estimator.
In the presence of random pipette error and the absence of systematic errors, the η_k fluctuate randomly around η and this term will not disappear. Hence, the asymptotic variance estimator will underestimate the variance. Pipette error, thus, introduces an additional source of between replicate variation. Technical replicates can be used to account for this. Empirical variance estimators will capture both the variation of the individual estimates (term A) and the variation of the θ_k around θ (term B).

Derivations for a model with unequal partition sizes

We assume in what follows that the probability of containing a copy is proportional to the partition size. Using previous notation, we have:

$$K = \sum_{i=1}^{n_r} Y_i \text{ with } Y_i \sim B(1; p_i) \Rightarrow E[K] = \sum_{i=1}^{n_r} p_i$$

For an experiment unequal partition sizes s_i we have:

$$p_i = e^{-\lambda_i} \text{ with } \lambda_i \propto s_i$$

When we consider a hypothetical reference experiment on the same replicate with equal partition size then

$$\sum_{i=1}^{n_r} \lambda_i = n_r \lambda$$

and we see that

$$E[K] = \sum_{i=1}^{n_r} p_i = \sum_{i=1}^{n_r} e^{-\lambda_i} = n_r \overline{e^{-\lambda_i}} \geq n_r e^{\overline{-\lambda_i}} = n_r e^{-\lambda} = n_r p$$

where the inequality follows from the property that an arithmetic average is always at least as large as a geometric average. Consequently, we have shown that the expected number of partitions without a copy is larger than the expected number under the equal partition size assumption. Note, that when the s_i are similar, so are the λ_i and thus the difference will be small.
Although at first sight invisible in the formula, this difference is highly dependent on the number of target copies in the mix. We have:

$$\hat{\lambda} = -\log\left(\frac{K}{n_r}\right) \quad \Rightarrow \quad \frac{d\hat{\lambda}(K)}{dK} = \frac{-1}{K}$$

We can see that changes for K closer to 0 (few negative partitions, high concentration of target copies) have a much larger influence on the estimate than changes for K closer to n_r (many negative partitions, low concentration of target copies). Consequently, the downwards bias as a result of unequal partition sizes will be especially visible when there are many target copies present in the reaction mix.
It is difficult to give a theoretical estimate for the variance in this case as every partition has a unique λ_i.

An optimal ratio to minimize misclassification

We can write the ratio of false negatives to false positives as a function of the concentration for an unbiased estimator. The estimator is unbiased if the expected number of false positives equals the expected number of false negatives. Define $\pi_{FPR} = P(positive\,signal\,|\,no\,target)$ the false positive rate and $\pi_{FNR} = P(negative\,signal\,|target)$ the false negative rate. Assume for simplicity an experiment with equal partition size and no pipette error. We consider $E[K]$ and $E_0[K] = E\,[K|\pi_{FPR} = \pi_{FNR} = 0]$, the expected number of partitions that return a negative signal and its expected value when there is no misclassification. Additionally, we study $E[\hat{p}]$ and $E_0[\hat{p}] = E\,[\hat{p}|\pi_{FPR} = \pi_{FNR} = 0]$, the associated proportion of partitions that return a negative signal. We have

$$E[K] = E_0\,[K]\,(1 - \pi_{FPR}) + (n^r - E_0\,[K])\,\pi_{FNR}$$
$$E[\hat{p}] = E_0\,[\hat{p}]\,(1 - \pi_{FPR}) + (1 - E_0\,[\hat{p}])\,\pi_{FNR}$$

In the absence of bias, $E[\hat{p}] = E_0\,[\hat{p}]$. We can solve this for π_{FNR}/π_{FPR} and get an estimate of the necessary ratio to get an unbiased estimator if the concentration is given or already estimated.

$$\frac{\pi_{FNR}}{\pi_{FPR}} = \frac{E[\hat{p}]}{1 - E[\hat{p}]} = \frac{e^{-\lambda}}{1 - e^{-\lambda}}$$

This confirms the intuition that for a small number of target copies, we can accept a higher rate of false negatives if we keep the false positive rate small. For very concentrated samples, a higher rate of false positives is not problematic, but we want to keep the false negative rate small.
Alternatively, we can solve the equation to $E[\hat{p}]$ to know for which concentration the ratio of the proportions can be equal to a certain given ratio.

$$E[\hat{p}] = \frac{\pi_{FNR}}{\pi_{FNR} + \pi_{FPR}} \Rightarrow \lambda = -\log\left(\frac{\pi_{FNR}}{\pi_{FNR} + \pi_{FPR}}\right)$$

If we have an estimate of the false positive and false negative rate, we cannot only estimate the bias, but also find the optimal concentration λ for which its estimate is unbiased. This can be used in combination with results of a dilution series to reduce bias. Note, that we need a less concentrated sample to reduce the bias if we expect a higher probability of false negatives, which may seem counter-intuitive.

References
1. De St Groth, S.: The evaluation of limiting dilution assays. J Immunol Methods **49**(2), 11–23 (1982)
2. Gregory, J.: Turbidity fluctuations in flowing suspensions. J Colloid Interface Sci **105**(2), 357–371 (1985)
3. Dube, S., Qin, J., Ramakrishnan, R.: Mathematical analysis of copy number variation in a DNA sample using digital PCR on a nanofluidic device. PLoS One **3**(8), 2876 (2008)
