# Supplementary material for: Impact of variance components on reliability of absolute quantification using digital PCR
Source: BMC Bioinformatics. 2014 Aug 22;15(1):283. doi: 10.1186/1471-2105-15-283 (PMC4261249; doi:10.1186/1471-2105-15-283)
Supplement: Supplementary file 2 — Additional file 2: Additional figures. This PDF file includes two additional figures on the width of confidence intervals. (PDF 117 KB) [file 12859_2014_6687_MOESM2_ESM.pdf]

Impact of variance components on reliability of absolute quantification using digital PCR - Additional File 2: Additional figures

Bart KM Jacobs*, Els Goetghebeur and Lieven Clement*

*Correspondence:
BartKM.Jacobs@UGent.be,
Lieven.Clement@UGent.be
Department of Applied Mathematics, Computer Science and Statistics, Ghent University
Krijgslaan 281, S9, 9000 Ghent, Belgium

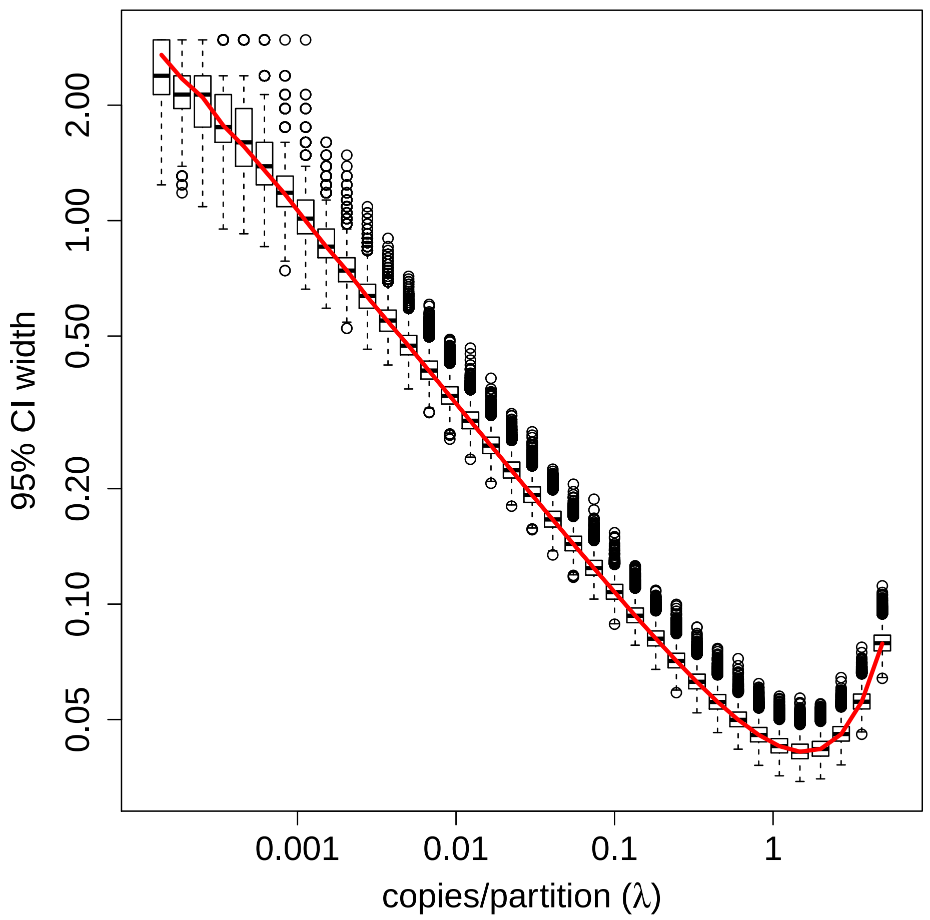

Figure S1 Relative width of confidence intervals of target concentration obtained under optimal theoretical condition. Each boxplot summarizes the widths of the 95% confidence intervals for the concentration in 8000 independent samples (8 replicates $\times 1000$ simulations). The width is given relative to the estimated concentration $\hat{\lambda}$, such that the precision of different dilutions of the same sample can be assessed on the same scale. The red line shows the theoretical expected width of a confidence interval for the concentration under the assumptions of the Poisson model. Since the number of partitions was fixed at $20\,000$, confidence intervals have exactly the same relative width if samples contain the same number of target copies. Circles in the upper left corner, outliers for a small number of targets, represent up to hundreds of coinciding partitions.

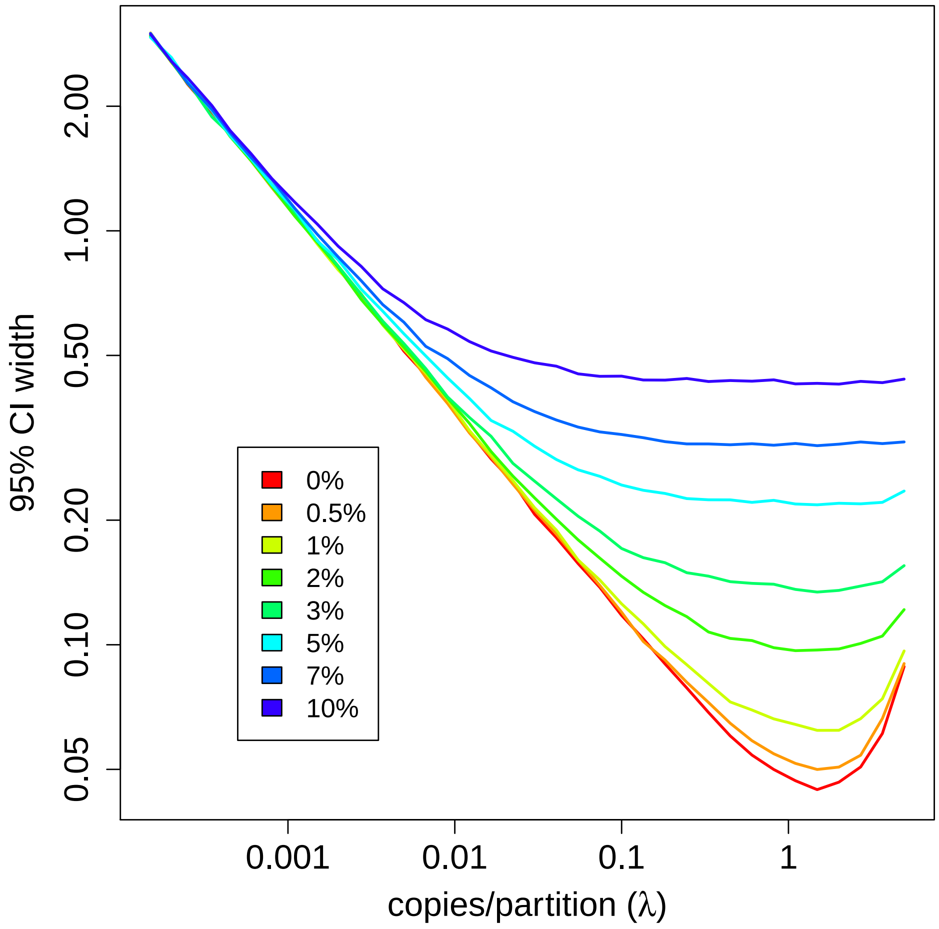

Figure S2 Relative width of 95% confidence intervals of target concentration in the presence of pipette error. For a given concentration λ (copies/partition), the width of a 95% confidence interval with correct coverage is calculated in the presence of different amounts of pipette error. The width is calculated by taking the average of the widths of 1000 confidence intervals obtained with the replicate based method. Results are divided by the true concentration λ such that the precision of different dilutions of the same sample can be assessed on the same scale. The width is similar for a wide range of concentrations when pipette error is large and much smaller for specific concentrations around 1 copy per partition when pipette error is small.
