# Supplementary material for: Impact of variance components on reliability of absolute quantification using digital PCR
Source: BMC Bioinformatics. 2014 Aug 22;15(1):283. doi: 10.1186/1471-2105-15-283 (PMC4261249; doi:10.1186/1471-2105-15-283)
Supplement: Supplementary file 4 — Additional file 4: Interactive tool. In this mini-website, we provide an interactive tool to study the influence of specific sources of variation on the performance of the concentration estimators. This can serve as a guide when designing an experiment. All results are relative to the true concentration and based on 1000 simulations with 8 technical replicates. (ZIP 17 MB) [file 12859_2014_6687_MOESM4_ESM.zip › Additional file 4/index.html]

Jacobs et al.: Impact of variance components on reliability of absolute quantification using digital PCR


Performance of concentration estimators for absolute quantification with digital PCR

partition size
equal size
small differences
large differences
 
 pipette error
0%
1%
2.5%
5%
10%
 rain
no rain
light rain
heavy rain
 
 threshold
7500
7000
6000
5000
4000
3000
2500
for 1000 simulations

This interactive tool compares the performance of two concentration estimators for absolute quantification of Nucleic Acids with dPCR in the presence of technical replicates: the Pooled Estimator (PE) and Replicate based Estimator (RE). The PE (dashed red line) estimates the concentration and variance after pooling the partitions of the technical replicates based on Poisson statistics. The RE (full green line) consists of the empirical mean and variance of the concentration estimates of the independent replicates. The impact of different sources of variation and modifiable settings in the dPCR workflow are assessed, i.e.   partition size,  pipette error,  rain, and the choice of the  threshold to discriminate between positive and negative partitions.   
The top left panel displays an example of typical fluorescence outcomes for a dPCR experiment with 20 000 partitions. The color represents the density of partitions (color scheme in right axis 1-5 partitions). The black line indicates the threshold and can be adapted with the dropdown menu.   
In the remaining panels results are given relative to the true concentration λ. Hence, the precision and accuracy of different dilutions of the same sample can be compared on the same scale.  
The top center panel shows the relative standard deviation estimated by the PE and RE averaged over 1000 simulations. When pipetting error is present, the pooled method underestimates the standard deviation.   
In the top right panel the coverage of 95% confidence intervals for the two methods is given. The coverage was calculated as the ratio of the number of confidence intervals out of 1000 simulations that contain the true concentration λ divided by the total number of confidence intervals calculated (1000).   
The lower left panel shows the relative empirical standard deviation estimated from the results of 1000 simulations for the two methods. This is calculated by taking the standard deviation of the concentration estimates over the 1000 simulations and gives a better view on the true standard deviation of each method.
  
In lower center panel the relative bias averaged over 1000 simulations is plotted for each method.   
The lower right panel shows the relative RMSE estimated from the results of 1000 simulations for the two methods. This is estimated as the square root of the sum of the relative variance and squared relative bias.

©2014 Bart KM Jacobs, Els Goetghebeur and Lieven Clement
