# Supplementary figures and images for: Impact of variance components on reliability of absolute quantification using digital PCR (part 2 of 2)
Source: BMC Bioinformatics. 2014 Aug 22;15(1):283. doi: 10.1186/1471-2105-15-283 (PMC4261249; doi:10.1186/1471-2105-15-283)

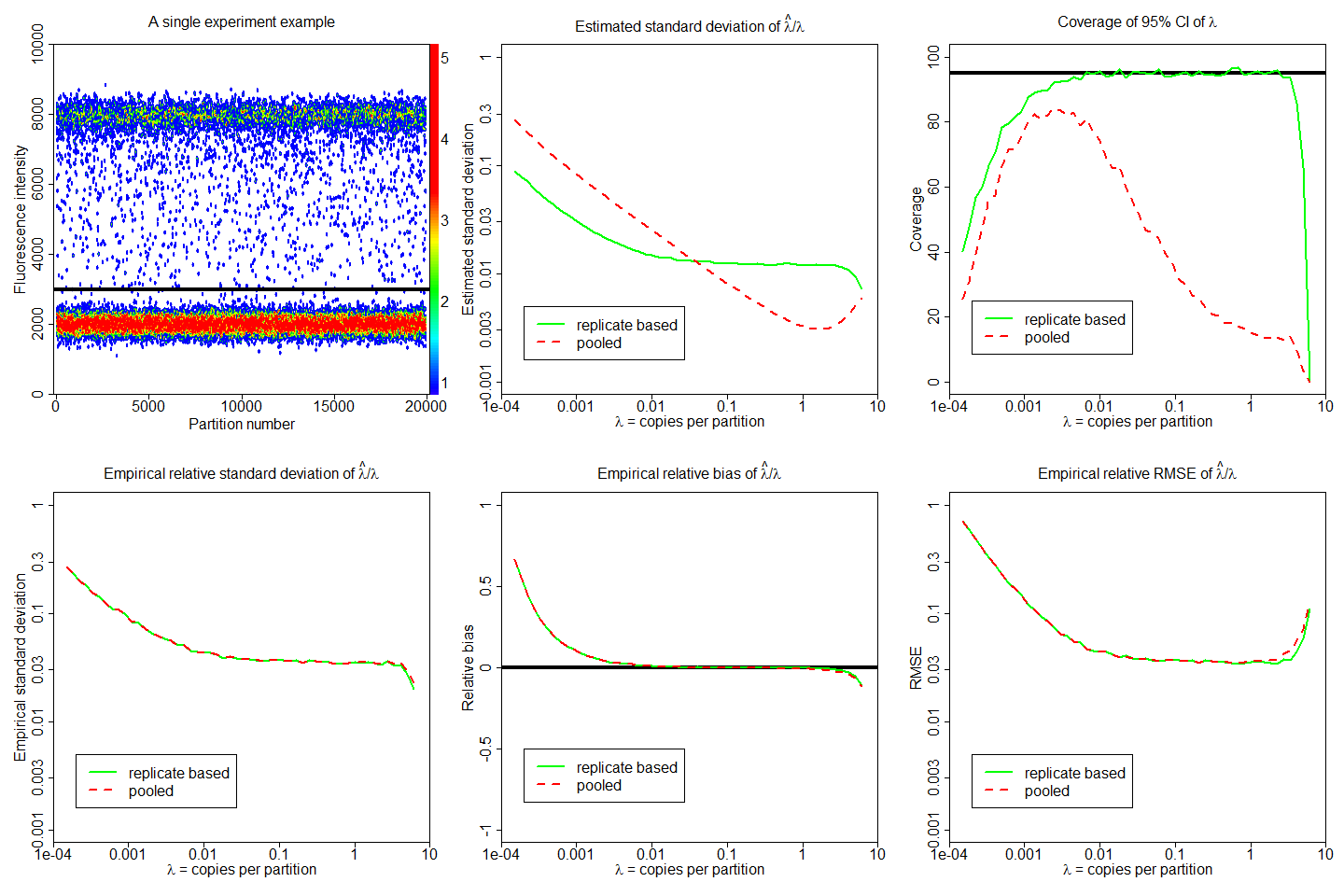

Supplement: Supplementary file 4 — Additional file 4: Interactive tool. In this mini-website, we provide an interactive tool to study the influence of specific sources of variation on the performance of the concentration estimators. This can serve as a guide when designing an experiment. All results are relative to the true concentration and based on 1000 simulations with 8 technical replicates. (ZIP 17 MB) [file 12859_2014_6687_MOESM4_ESM.zip › Additional file 4/RES/RES1562B.png]

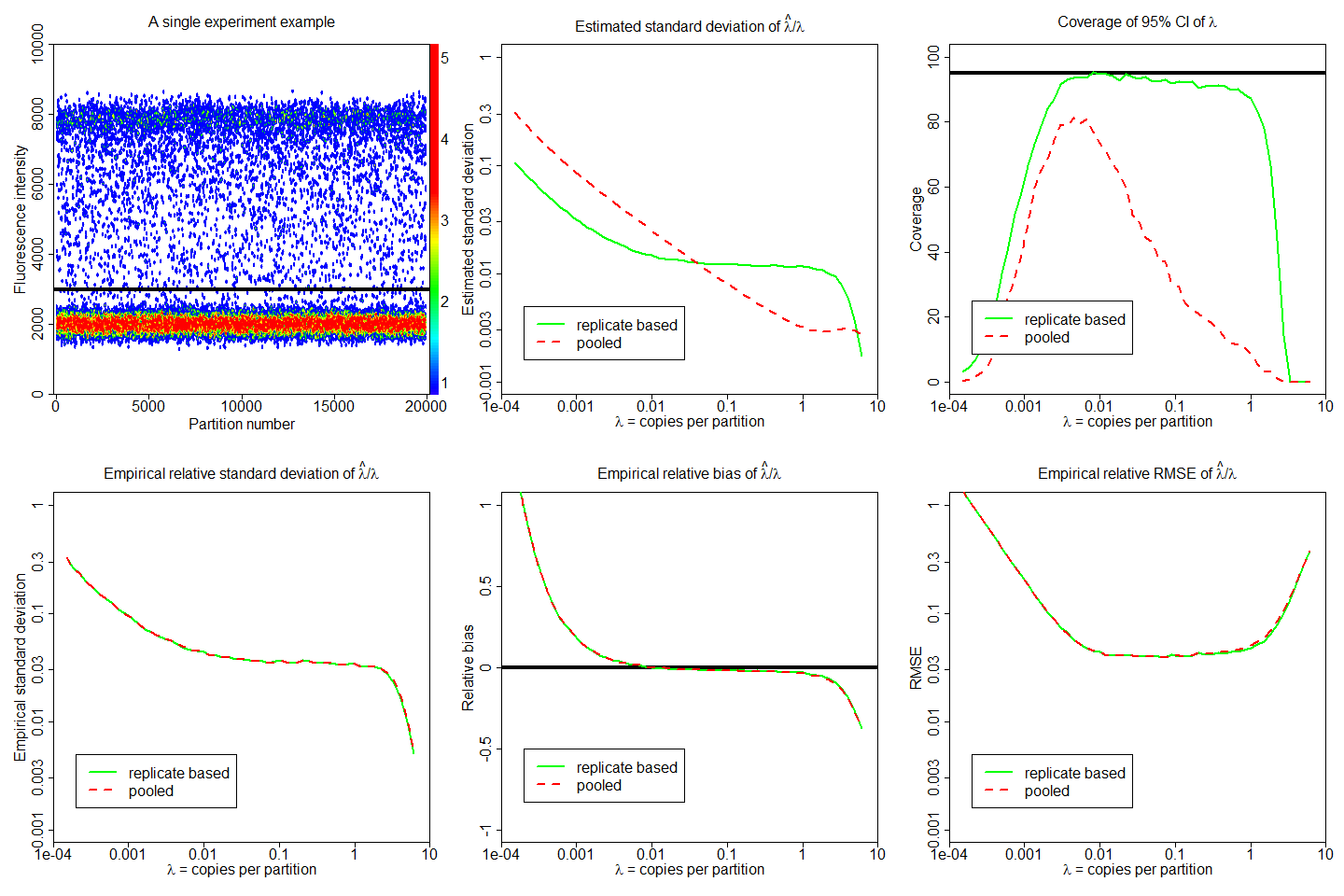

Supplement: Supplementary file 4 — Additional file 4: Interactive tool. In this mini-website, we provide an interactive tool to study the influence of specific sources of variation on the performance of the concentration estimators. This can serve as a guide when designing an experiment. All results are relative to the true concentration and based on 1000 simulations with 8 technical replicates. (ZIP 17 MB) [file 12859_2014_6687_MOESM4_ESM.zip › Additional file 4/RES/RES1563B.png]

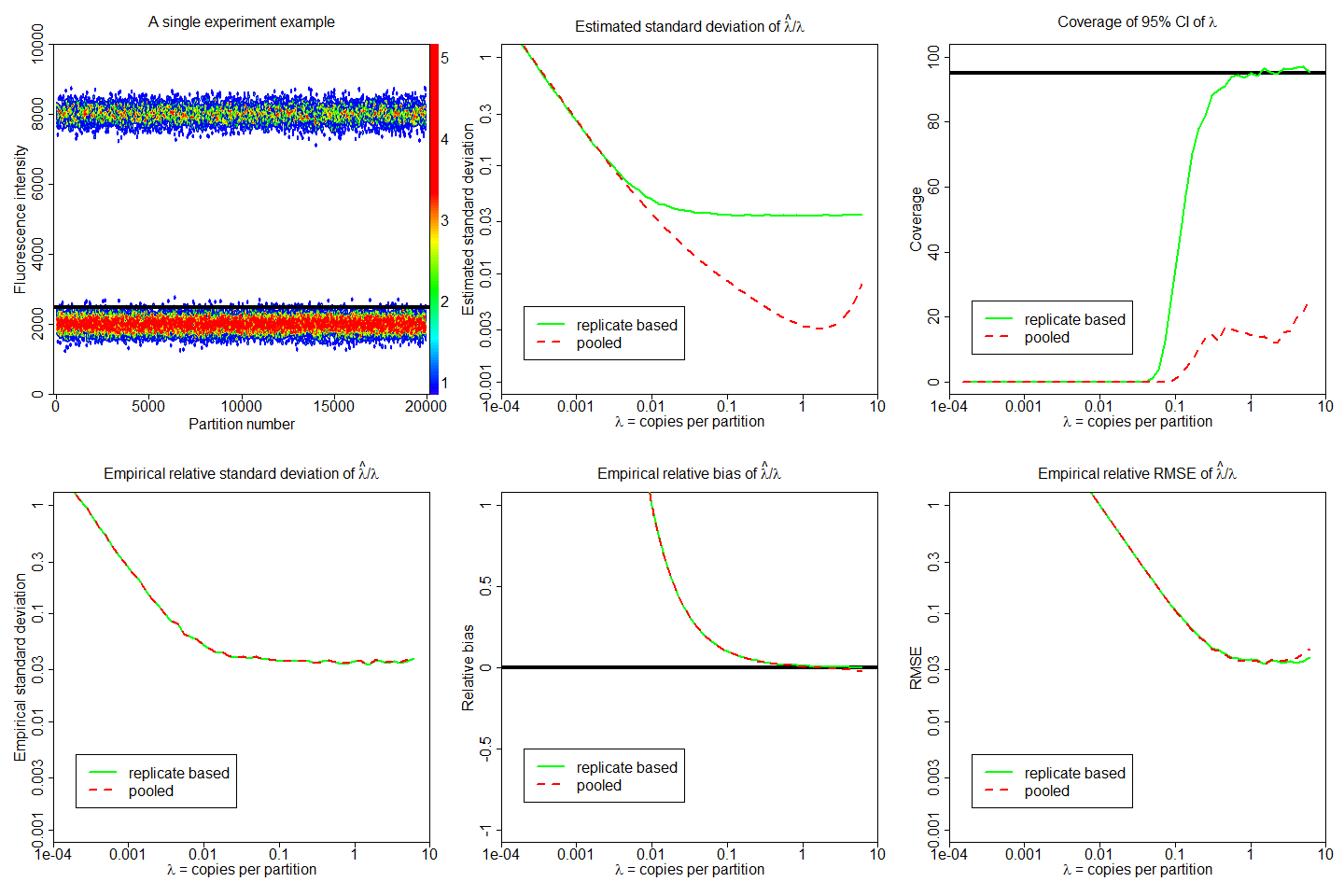

Supplement: Supplementary file 4 — Additional file 4: Interactive tool. In this mini-website, we provide an interactive tool to study the influence of specific sources of variation on the performance of the concentration estimators. This can serve as a guide when designing an experiment. All results are relative to the true concentration and based on 1000 simulations with 8 technical replicates. (ZIP 17 MB) [file 12859_2014_6687_MOESM4_ESM.zip › Additional file 4/RES/RES1571B.png]

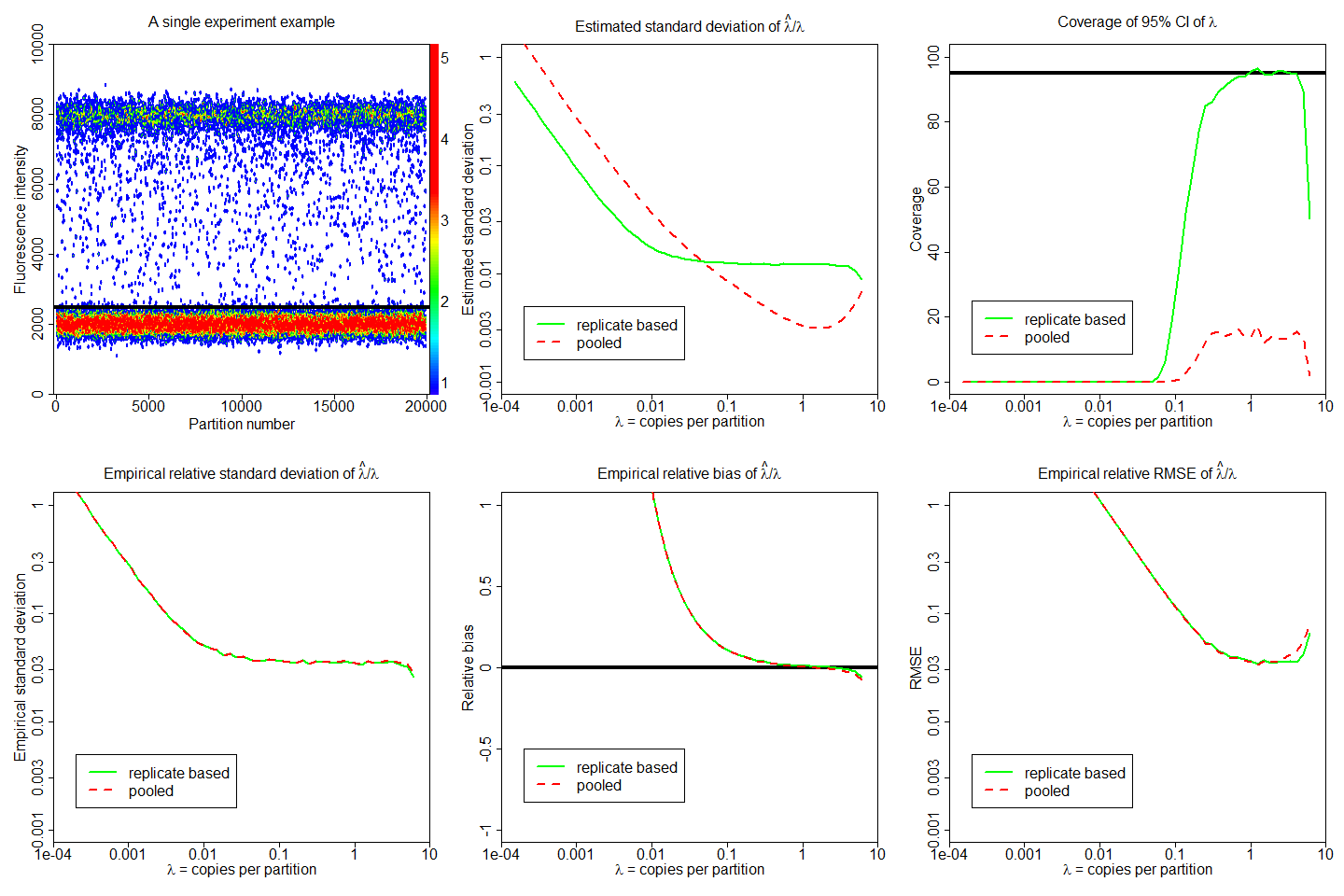

Supplement: Supplementary file 4 — Additional file 4: Interactive tool. In this mini-website, we provide an interactive tool to study the influence of specific sources of variation on the performance of the concentration estimators. This can serve as a guide when designing an experiment. All results are relative to the true concentration and based on 1000 simulations with 8 technical replicates. (ZIP 17 MB) [file 12859_2014_6687_MOESM4_ESM.zip › Additional file 4/RES/RES1572B.png]

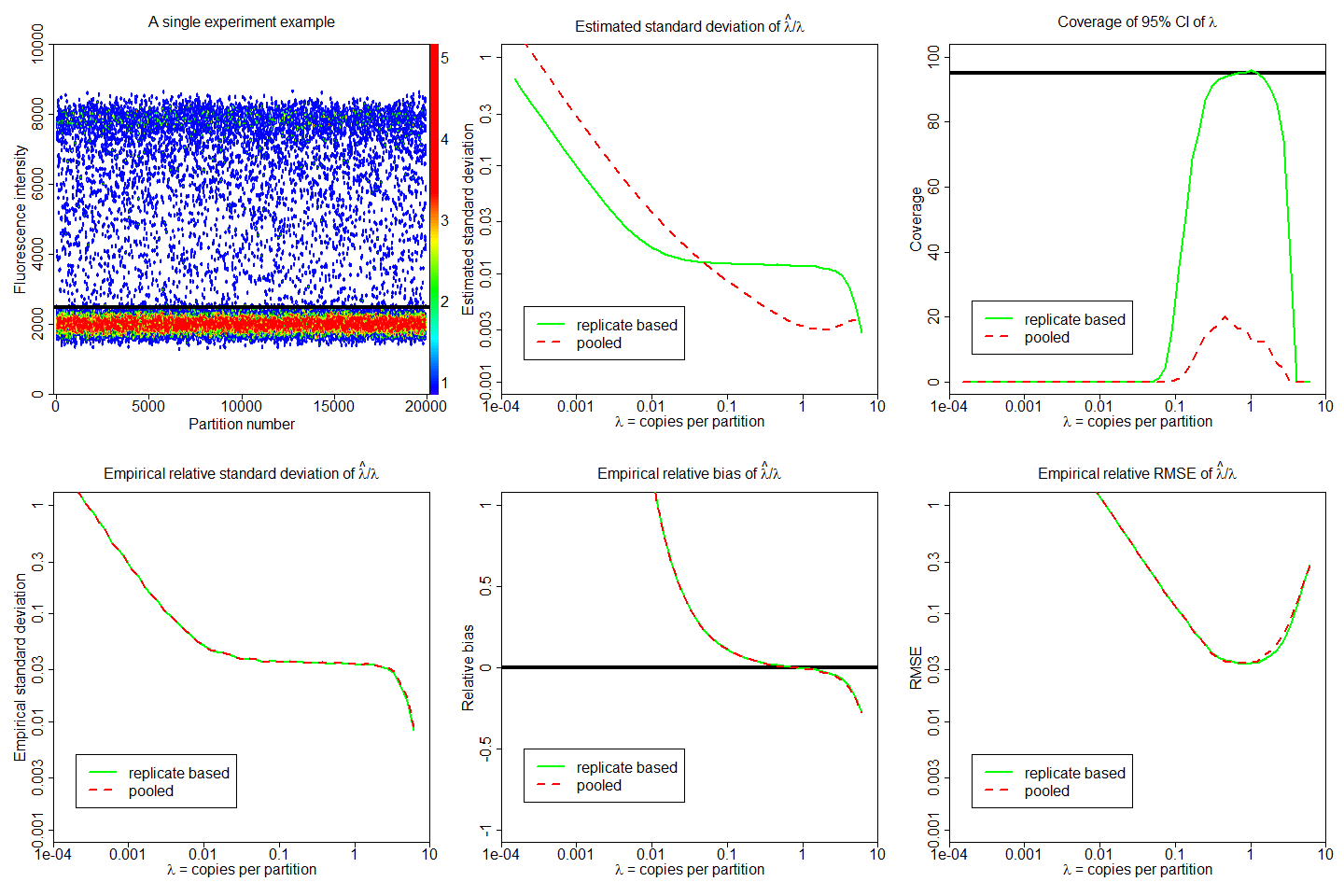

Supplement: Supplementary file 4 — Additional file 4: Interactive tool. In this mini-website, we provide an interactive tool to study the influence of specific sources of variation on the performance of the concentration estimators. This can serve as a guide when designing an experiment. All results are relative to the true concentration and based on 1000 simulations with 8 technical replicates. (ZIP 17 MB) [file 12859_2014_6687_MOESM4_ESM.zip › Additional file 4/RES/RES1573B.png]

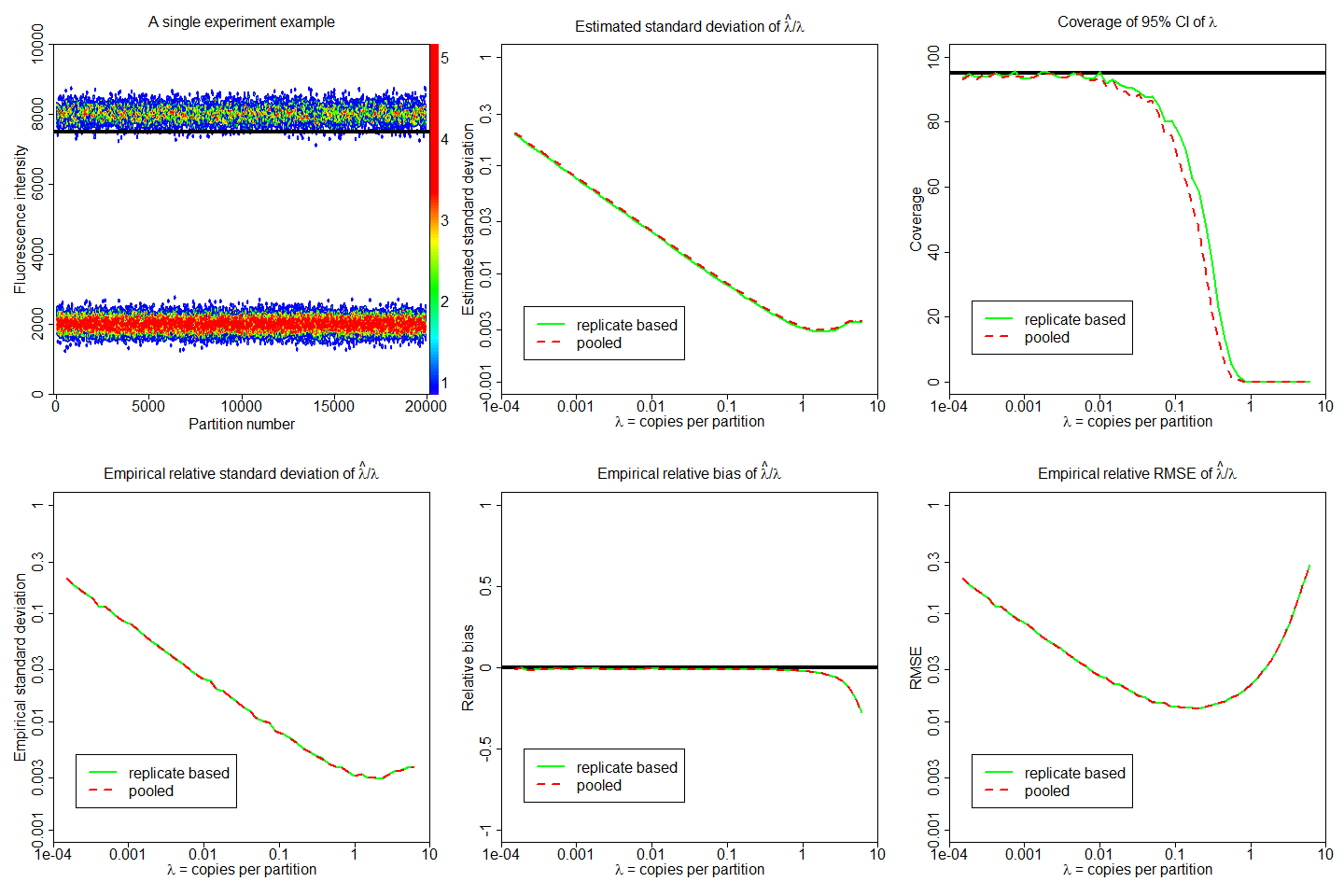

Supplement: Supplementary file 4 — Additional file 4: Interactive tool. In this mini-website, we provide an interactive tool to study the influence of specific sources of variation on the performance of the concentration estimators. This can serve as a guide when designing an experiment. All results are relative to the true concentration and based on 1000 simulations with 8 technical replicates. (ZIP 17 MB) [file 12859_2014_6687_MOESM4_ESM.zip › Additional file 4/RES/RES2111B.png]

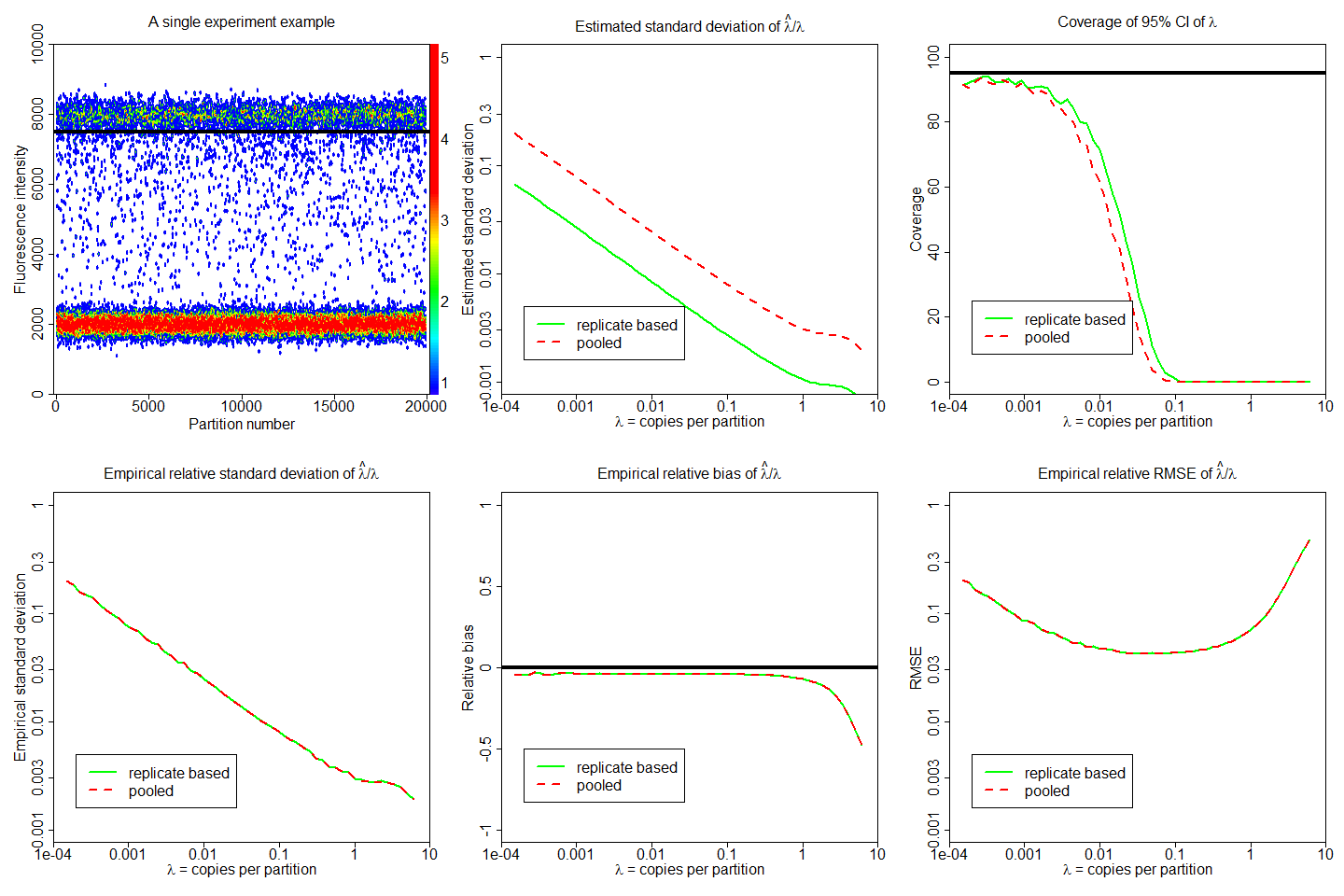

Supplement: Supplementary file 4 — Additional file 4: Interactive tool. In this mini-website, we provide an interactive tool to study the influence of specific sources of variation on the performance of the concentration estimators. This can serve as a guide when designing an experiment. All results are relative to the true concentration and based on 1000 simulations with 8 technical replicates. (ZIP 17 MB) [file 12859_2014_6687_MOESM4_ESM.zip › Additional file 4/RES/RES2112B.png]

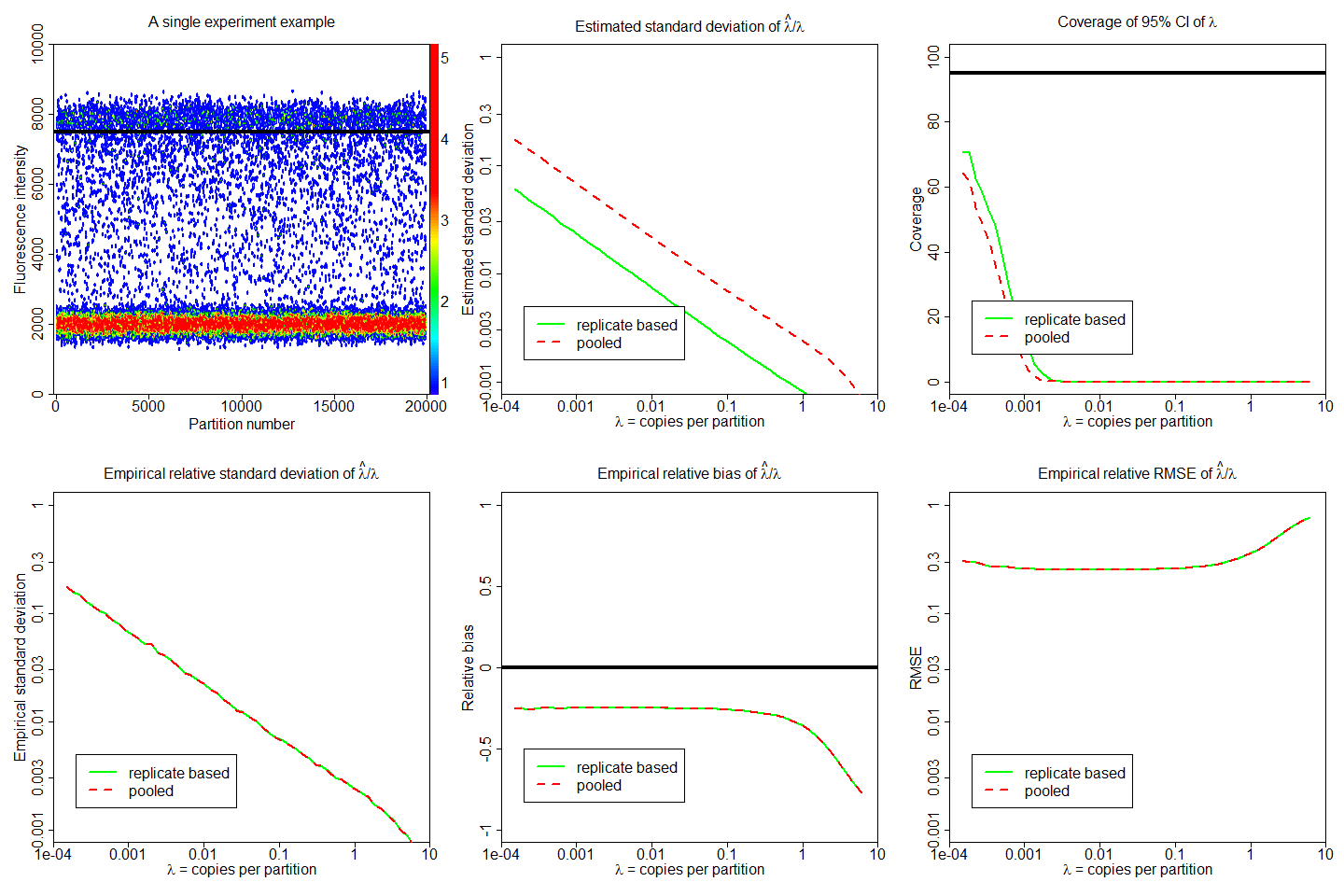

Supplement: Supplementary file 4 — Additional file 4: Interactive tool. In this mini-website, we provide an interactive tool to study the influence of specific sources of variation on the performance of the concentration estimators. This can serve as a guide when designing an experiment. All results are relative to the true concentration and based on 1000 simulations with 8 technical replicates. (ZIP 17 MB) [file 12859_2014_6687_MOESM4_ESM.zip › Additional file 4/RES/RES2113B.png]

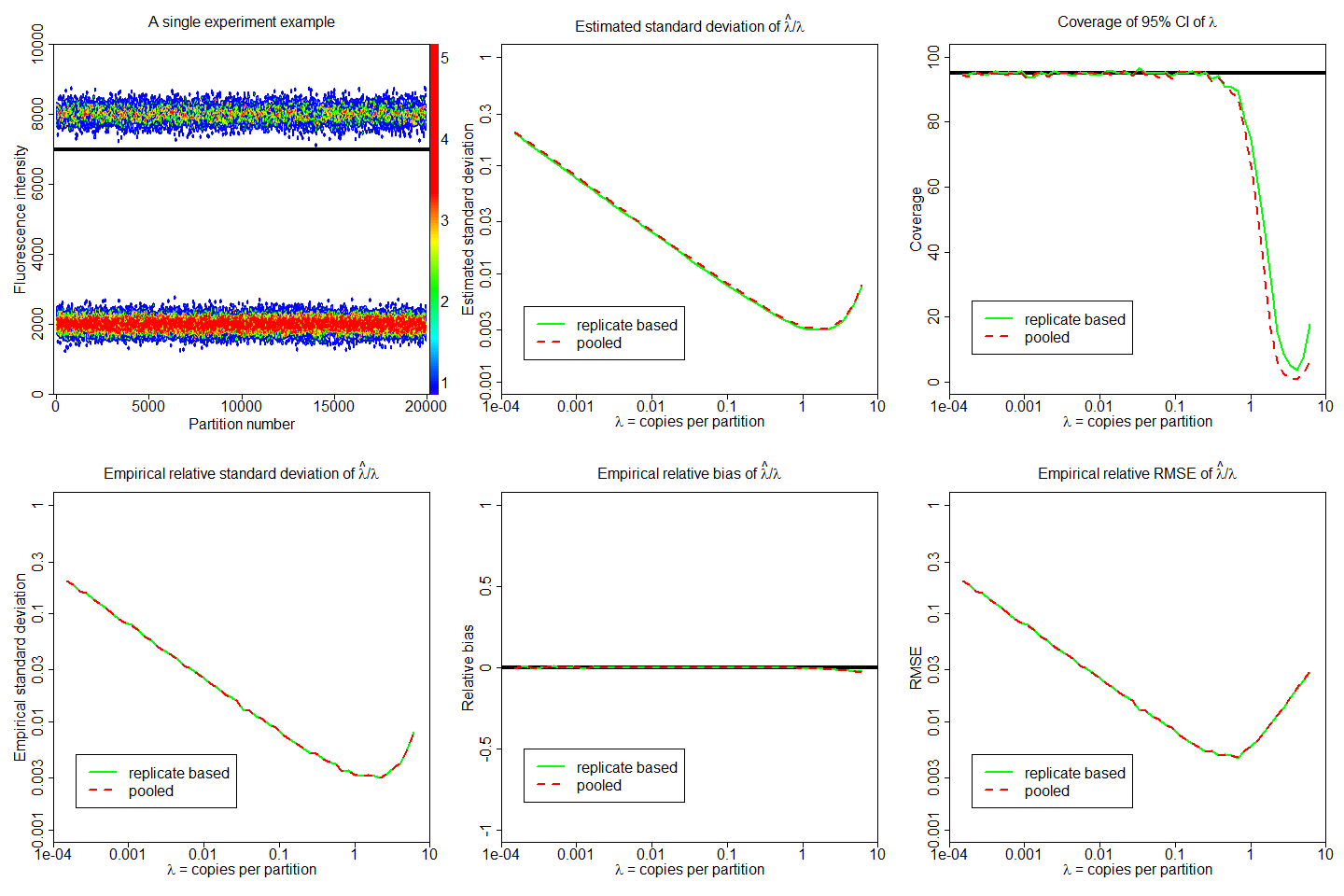

Supplement: Supplementary file 4 — Additional file 4: Interactive tool. In this mini-website, we provide an interactive tool to study the influence of specific sources of variation on the performance of the concentration estimators. This can serve as a guide when designing an experiment. All results are relative to the true concentration and based on 1000 simulations with 8 technical replicates. (ZIP 17 MB) [file 12859_2014_6687_MOESM4_ESM.zip › Additional file 4/RES/RES2121B.png]

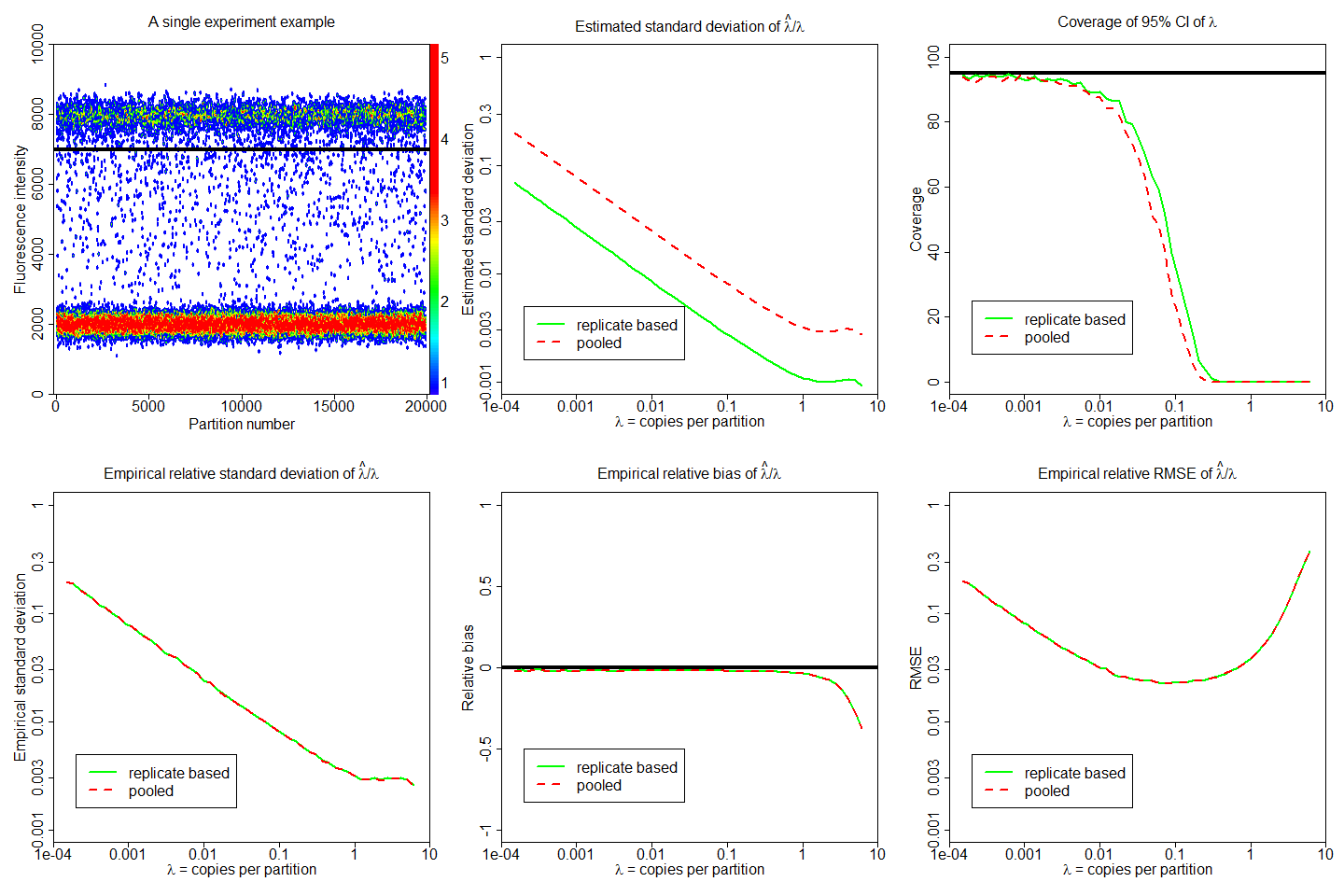

Supplement: Supplementary file 4 — Additional file 4: Interactive tool. In this mini-website, we provide an interactive tool to study the influence of specific sources of variation on the performance of the concentration estimators. This can serve as a guide when designing an experiment. All results are relative to the true concentration and based on 1000 simulations with 8 technical replicates. (ZIP 17 MB) [file 12859_2014_6687_MOESM4_ESM.zip › Additional file 4/RES/RES2122B.png]

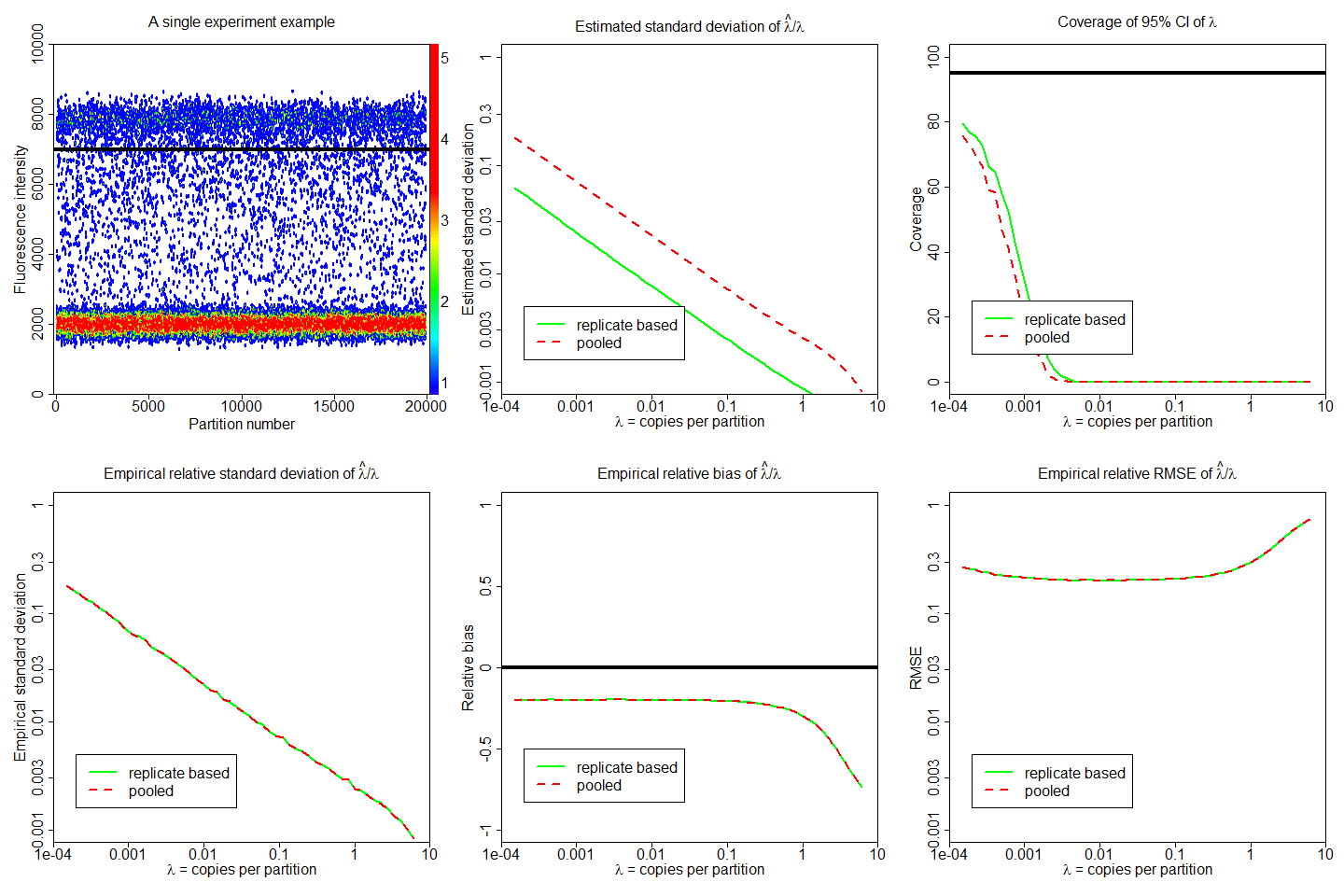

Supplement: Supplementary file 4 — Additional file 4: Interactive tool. In this mini-website, we provide an interactive tool to study the influence of specific sources of variation on the performance of the concentration estimators. This can serve as a guide when designing an experiment. All results are relative to the true concentration and based on 1000 simulations with 8 technical replicates. (ZIP 17 MB) [file 12859_2014_6687_MOESM4_ESM.zip › Additional file 4/RES/RES2123B.png]

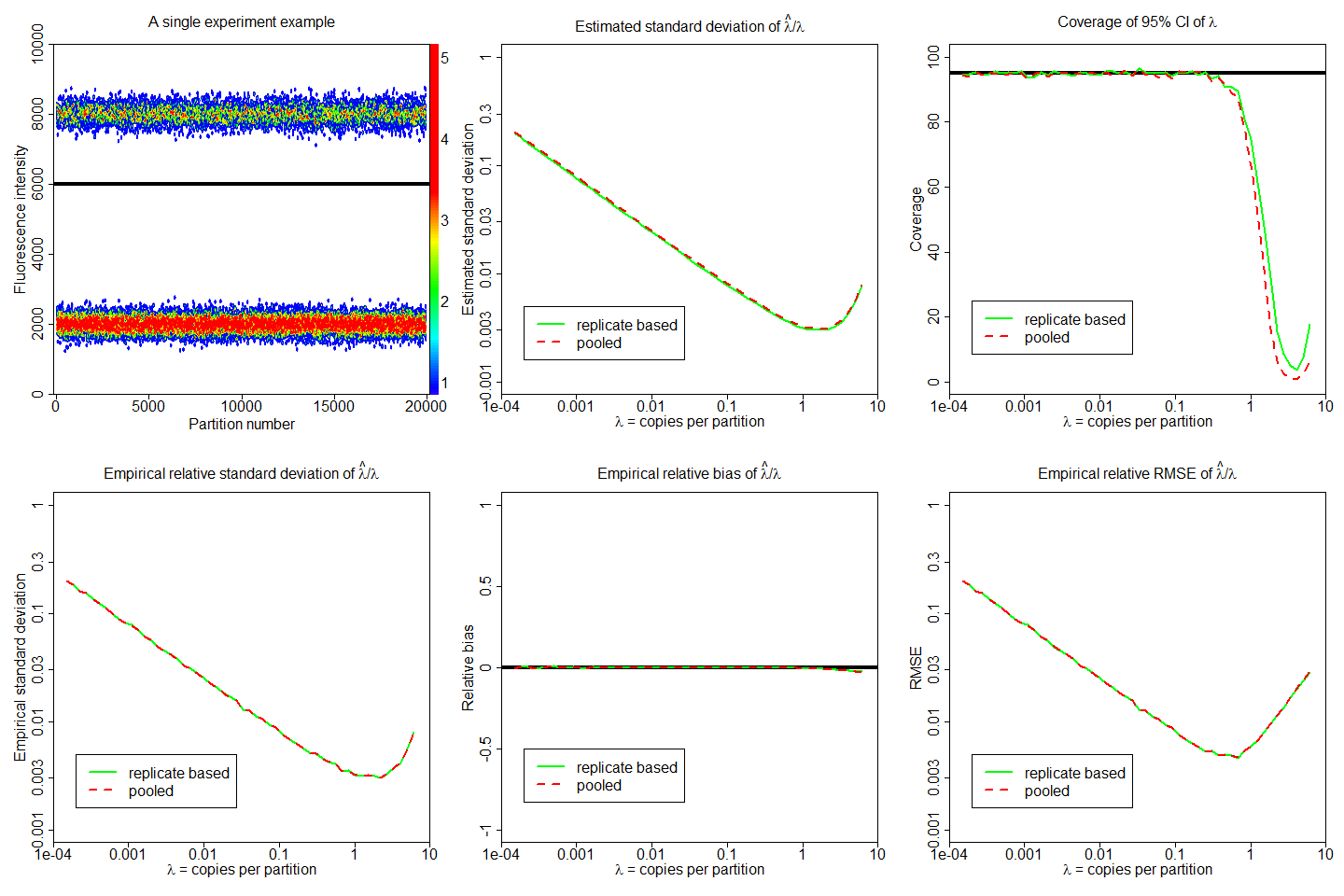

Supplement: Supplementary file 4 — Additional file 4: Interactive tool. In this mini-website, we provide an interactive tool to study the influence of specific sources of variation on the performance of the concentration estimators. This can serve as a guide when designing an experiment. All results are relative to the true concentration and based on 1000 simulations with 8 technical replicates. (ZIP 17 MB) [file 12859_2014_6687_MOESM4_ESM.zip › Additional file 4/RES/RES2131B.png]

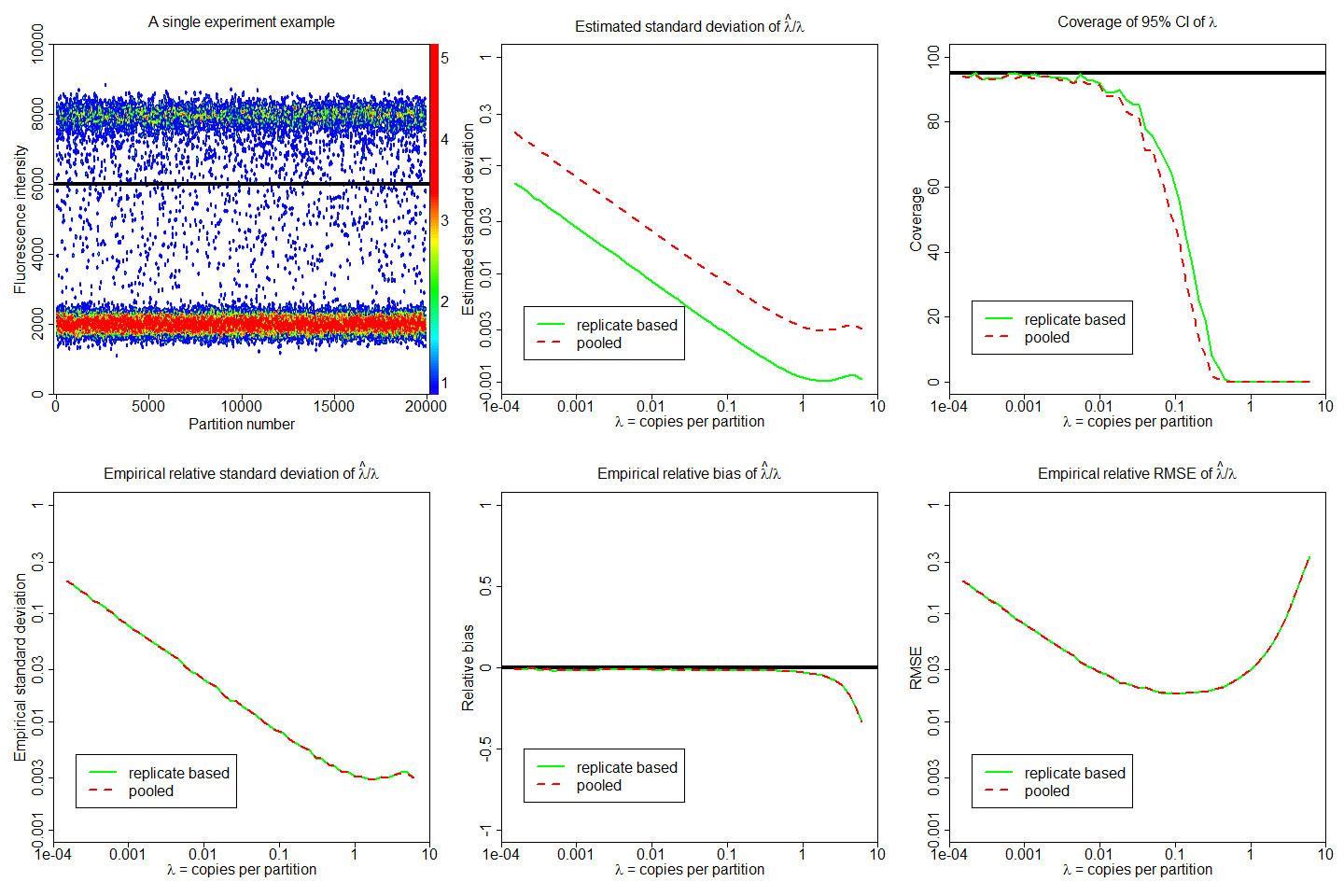

Supplement: Supplementary file 4 — Additional file 4: Interactive tool. In this mini-website, we provide an interactive tool to study the influence of specific sources of variation on the performance of the concentration estimators. This can serve as a guide when designing an experiment. All results are relative to the true concentration and based on 1000 simulations with 8 technical replicates. (ZIP 17 MB) [file 12859_2014_6687_MOESM4_ESM.zip › Additional file 4/RES/RES2132B.png]

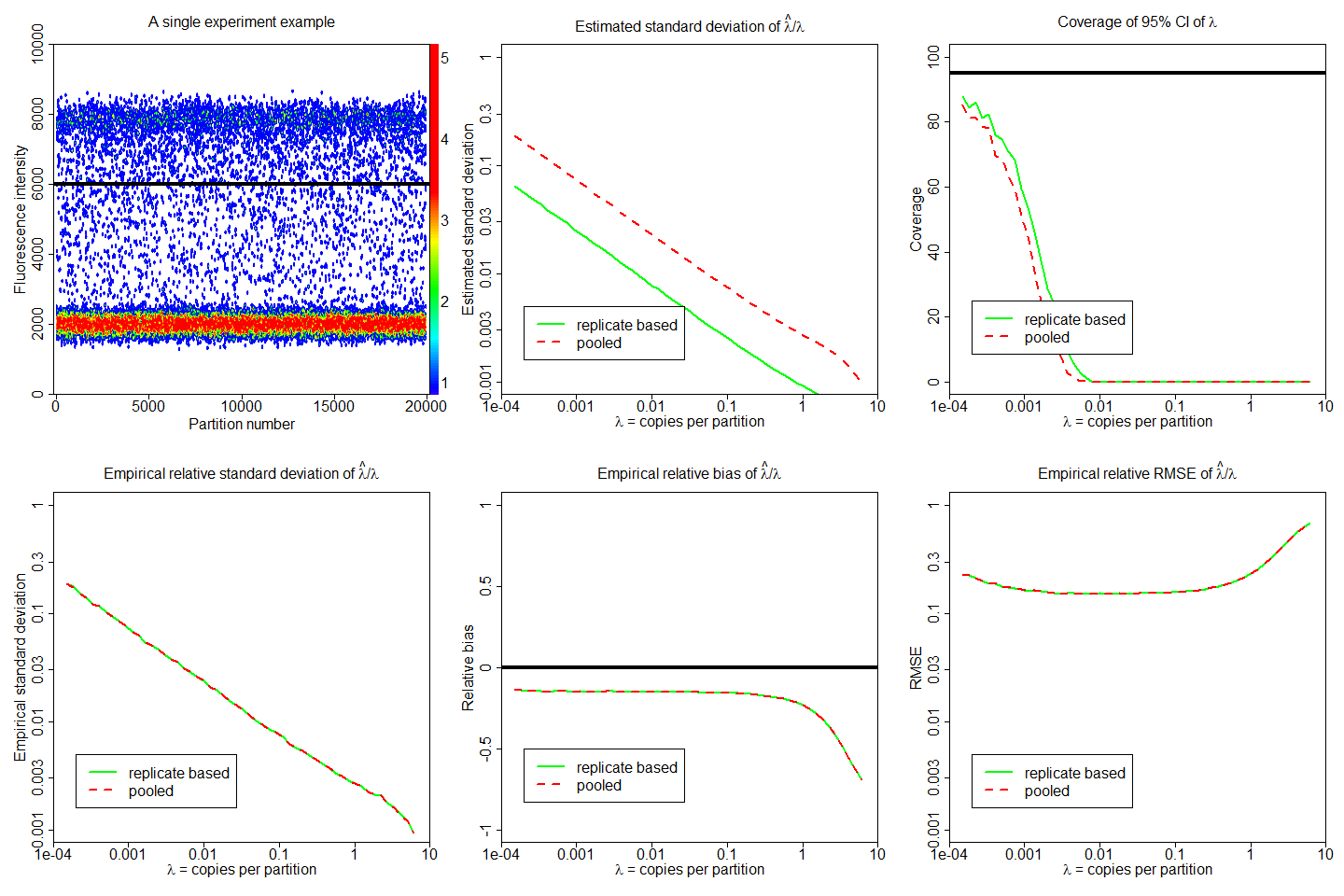

Supplement: Supplementary file 4 — Additional file 4: Interactive tool. In this mini-website, we provide an interactive tool to study the influence of specific sources of variation on the performance of the concentration estimators. This can serve as a guide when designing an experiment. All results are relative to the true concentration and based on 1000 simulations with 8 technical replicates. (ZIP 17 MB) [file 12859_2014_6687_MOESM4_ESM.zip › Additional file 4/RES/RES2133B.png]

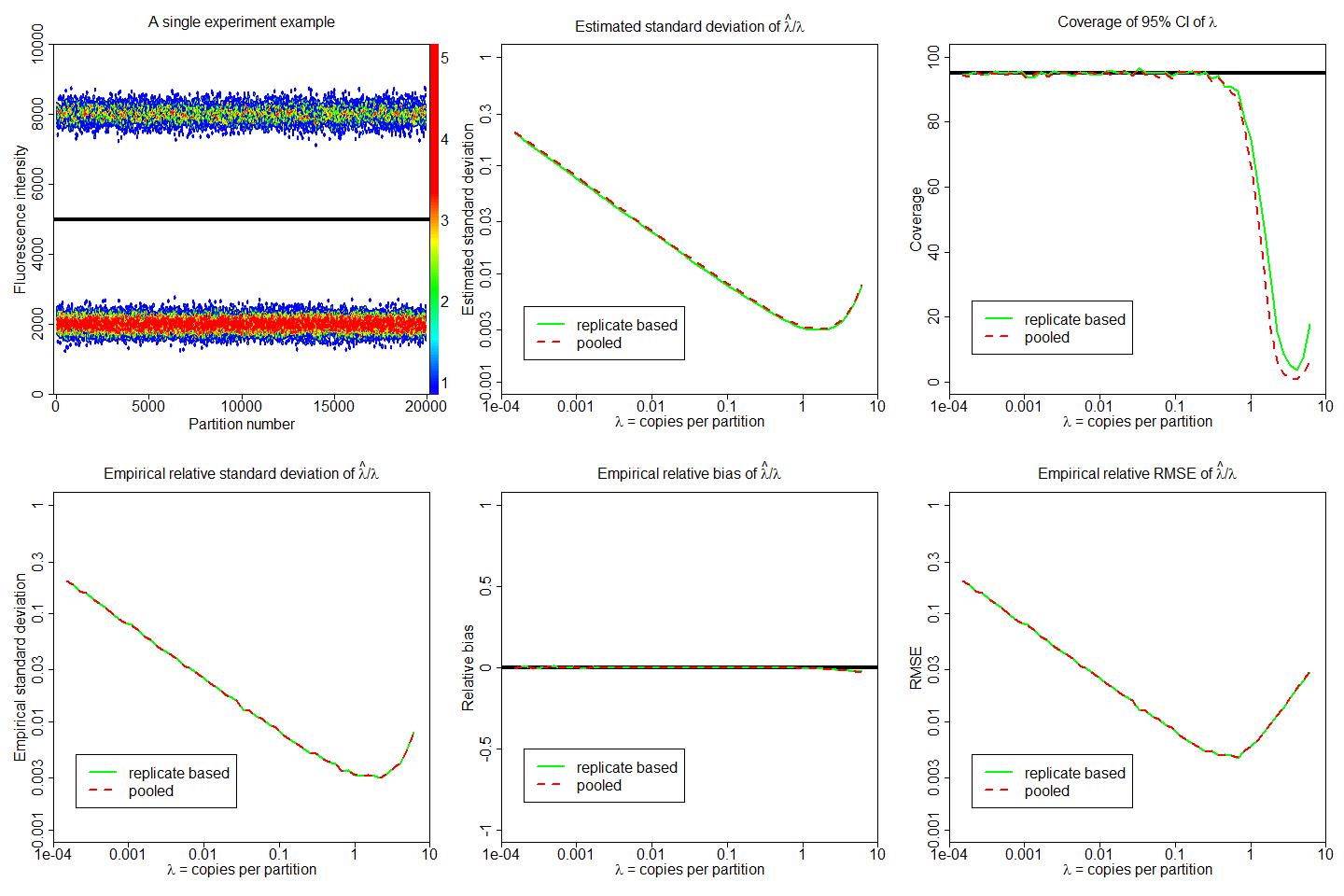

Supplement: Supplementary file 4 — Additional file 4: Interactive tool. In this mini-website, we provide an interactive tool to study the influence of specific sources of variation on the performance of the concentration estimators. This can serve as a guide when designing an experiment. All results are relative to the true concentration and based on 1000 simulations with 8 technical replicates. (ZIP 17 MB) [file 12859_2014_6687_MOESM4_ESM.zip › Additional file 4/RES/RES2141B.png]

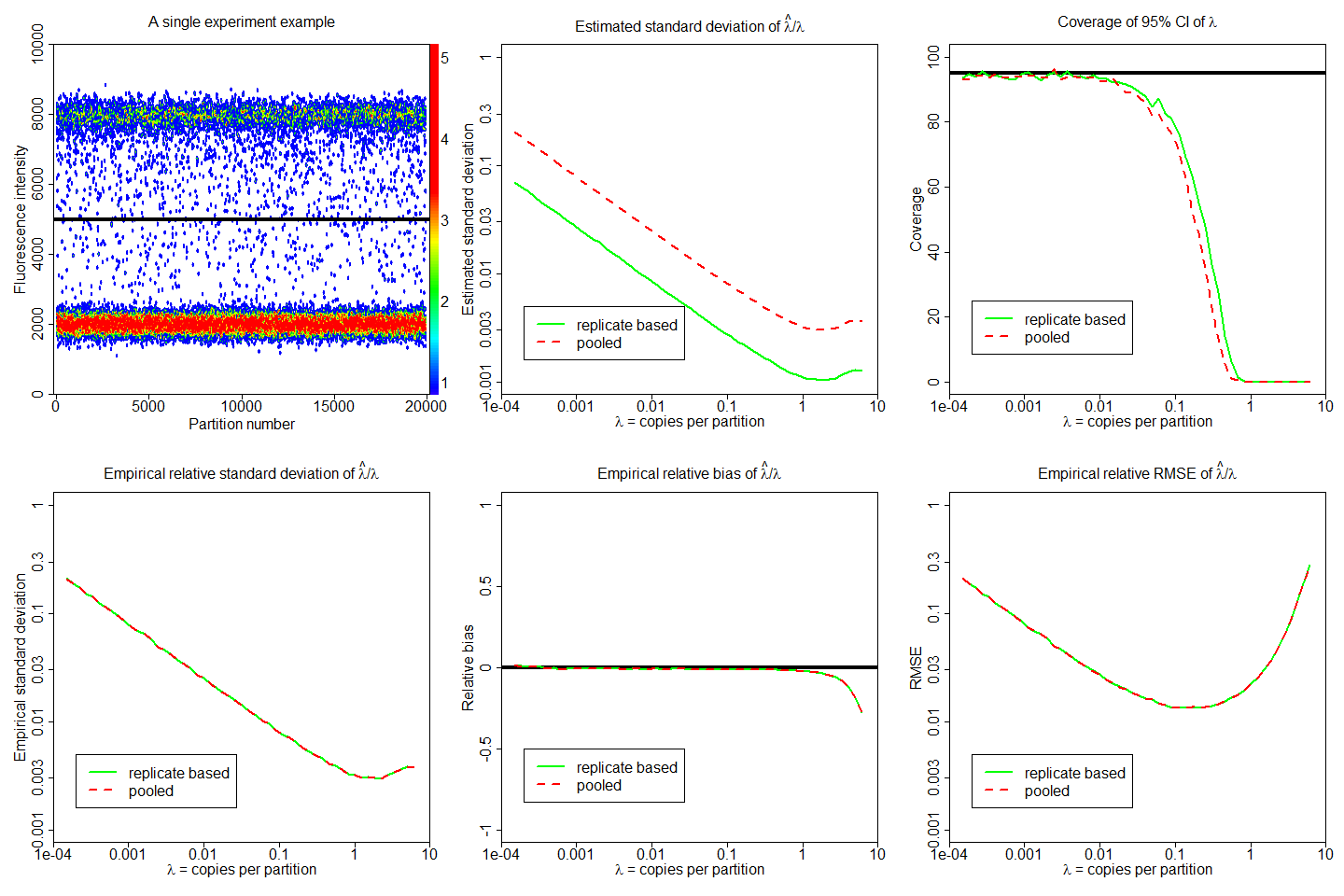

Supplement: Supplementary file 4 — Additional file 4: Interactive tool. In this mini-website, we provide an interactive tool to study the influence of specific sources of variation on the performance of the concentration estimators. This can serve as a guide when designing an experiment. All results are relative to the true concentration and based on 1000 simulations with 8 technical replicates. (ZIP 17 MB) [file 12859_2014_6687_MOESM4_ESM.zip › Additional file 4/RES/RES2142B.png]

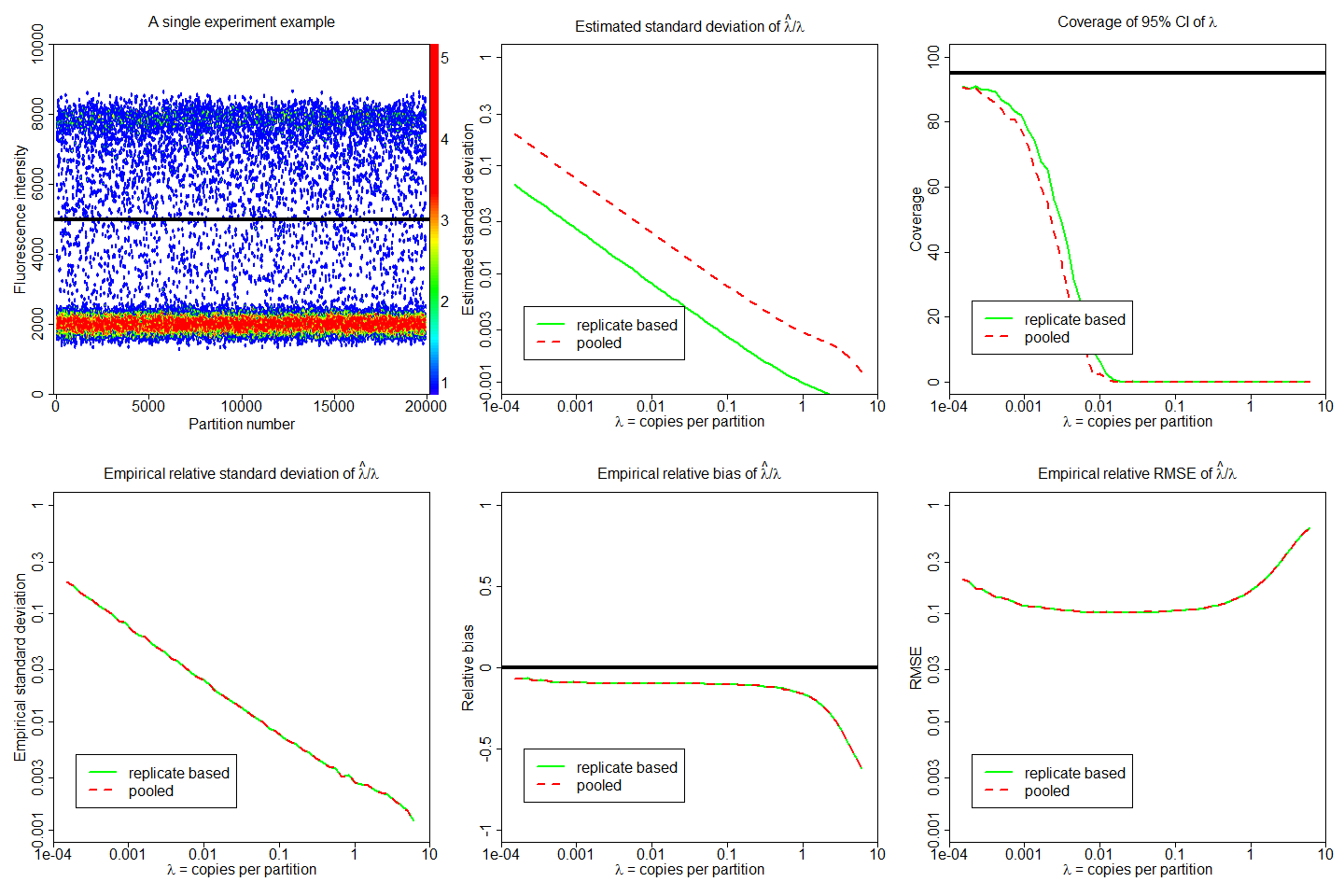

Supplement: Supplementary file 4 — Additional file 4: Interactive tool. In this mini-website, we provide an interactive tool to study the influence of specific sources of variation on the performance of the concentration estimators. This can serve as a guide when designing an experiment. All results are relative to the true concentration and based on 1000 simulations with 8 technical replicates. (ZIP 17 MB) [file 12859_2014_6687_MOESM4_ESM.zip › Additional file 4/RES/RES2143B.png]

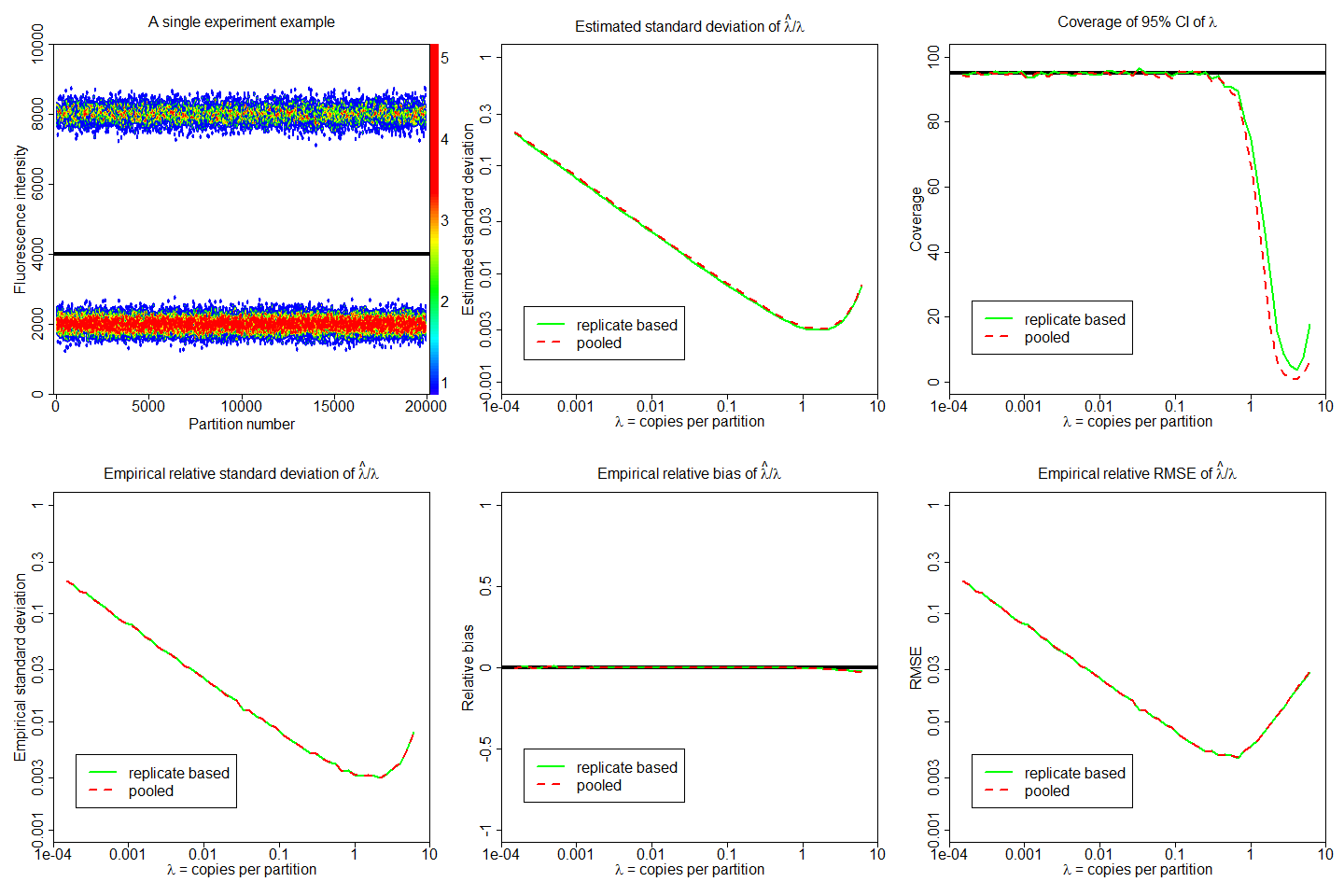

Supplement: Supplementary file 4 — Additional file 4: Interactive tool. In this mini-website, we provide an interactive tool to study the influence of specific sources of variation on the performance of the concentration estimators. This can serve as a guide when designing an experiment. All results are relative to the true concentration and based on 1000 simulations with 8 technical replicates. (ZIP 17 MB) [file 12859_2014_6687_MOESM4_ESM.zip › Additional file 4/RES/RES2151B.png]

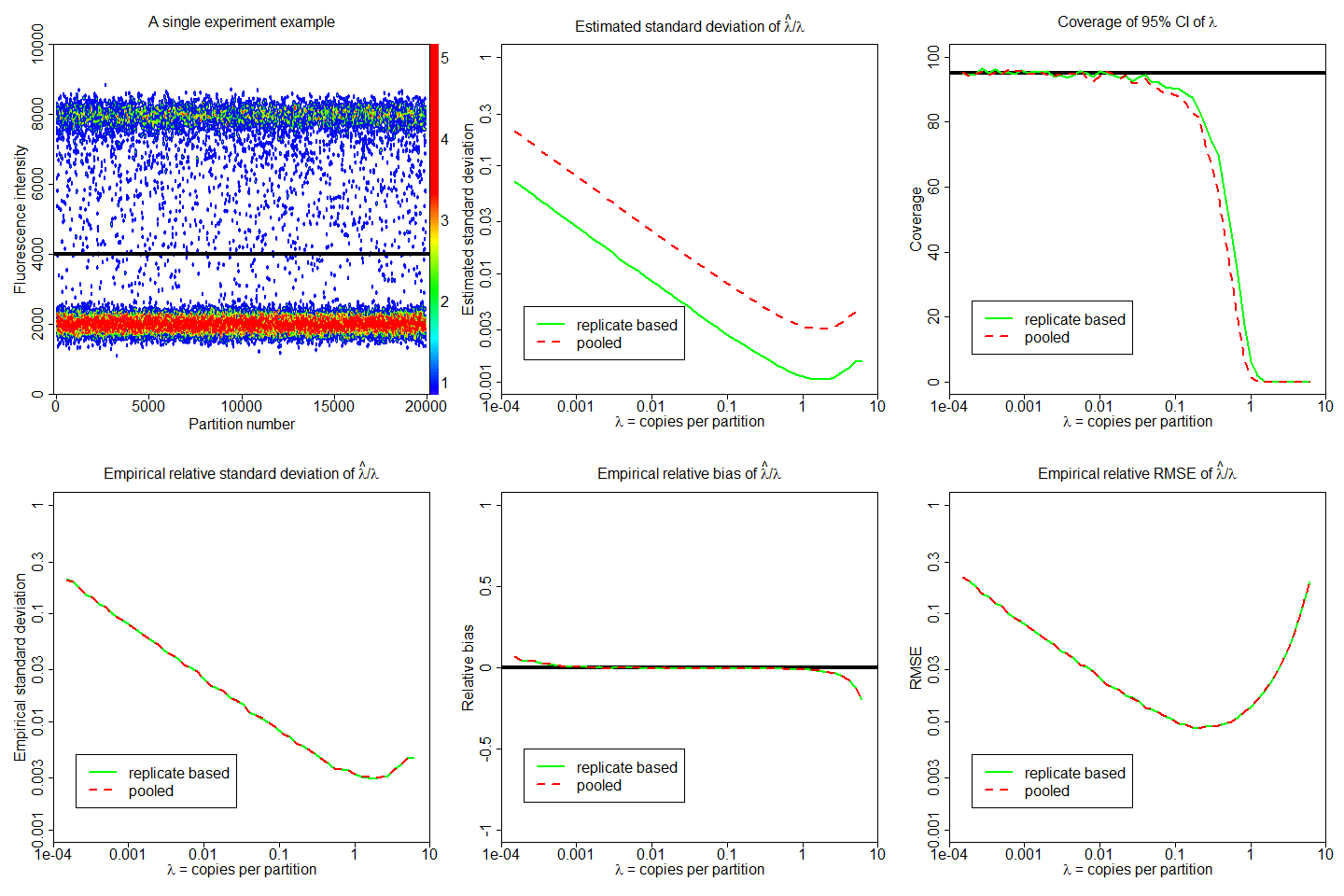

Supplement: Supplementary file 4 — Additional file 4: Interactive tool. In this mini-website, we provide an interactive tool to study the influence of specific sources of variation on the performance of the concentration estimators. This can serve as a guide when designing an experiment. All results are relative to the true concentration and based on 1000 simulations with 8 technical replicates. (ZIP 17 MB) [file 12859_2014_6687_MOESM4_ESM.zip › Additional file 4/RES/RES2152B.png]

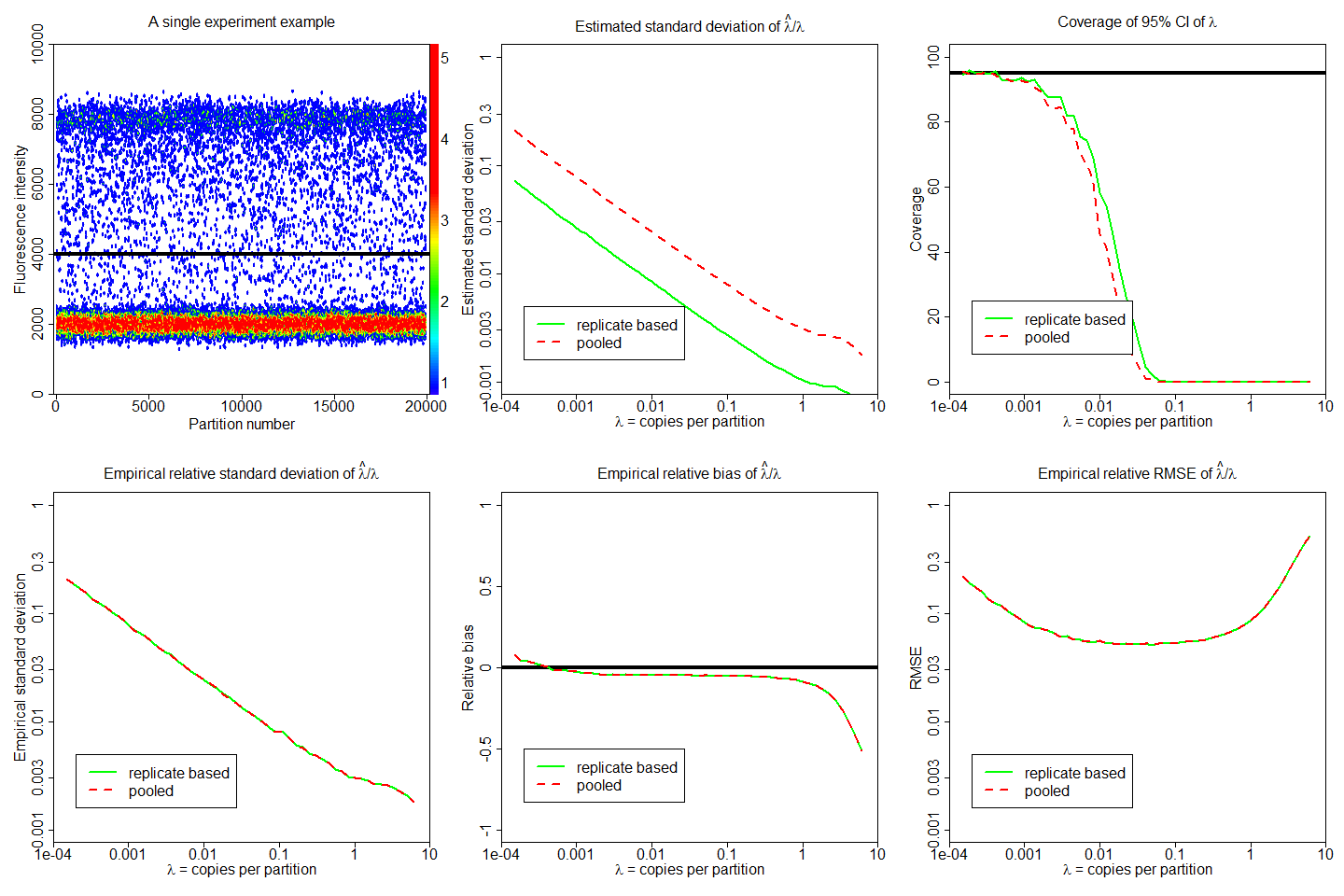

Supplement: Supplementary file 4 — Additional file 4: Interactive tool. In this mini-website, we provide an interactive tool to study the influence of specific sources of variation on the performance of the concentration estimators. This can serve as a guide when designing an experiment. All results are relative to the true concentration and based on 1000 simulations with 8 technical replicates. (ZIP 17 MB) [file 12859_2014_6687_MOESM4_ESM.zip › Additional file 4/RES/RES2153B.png]

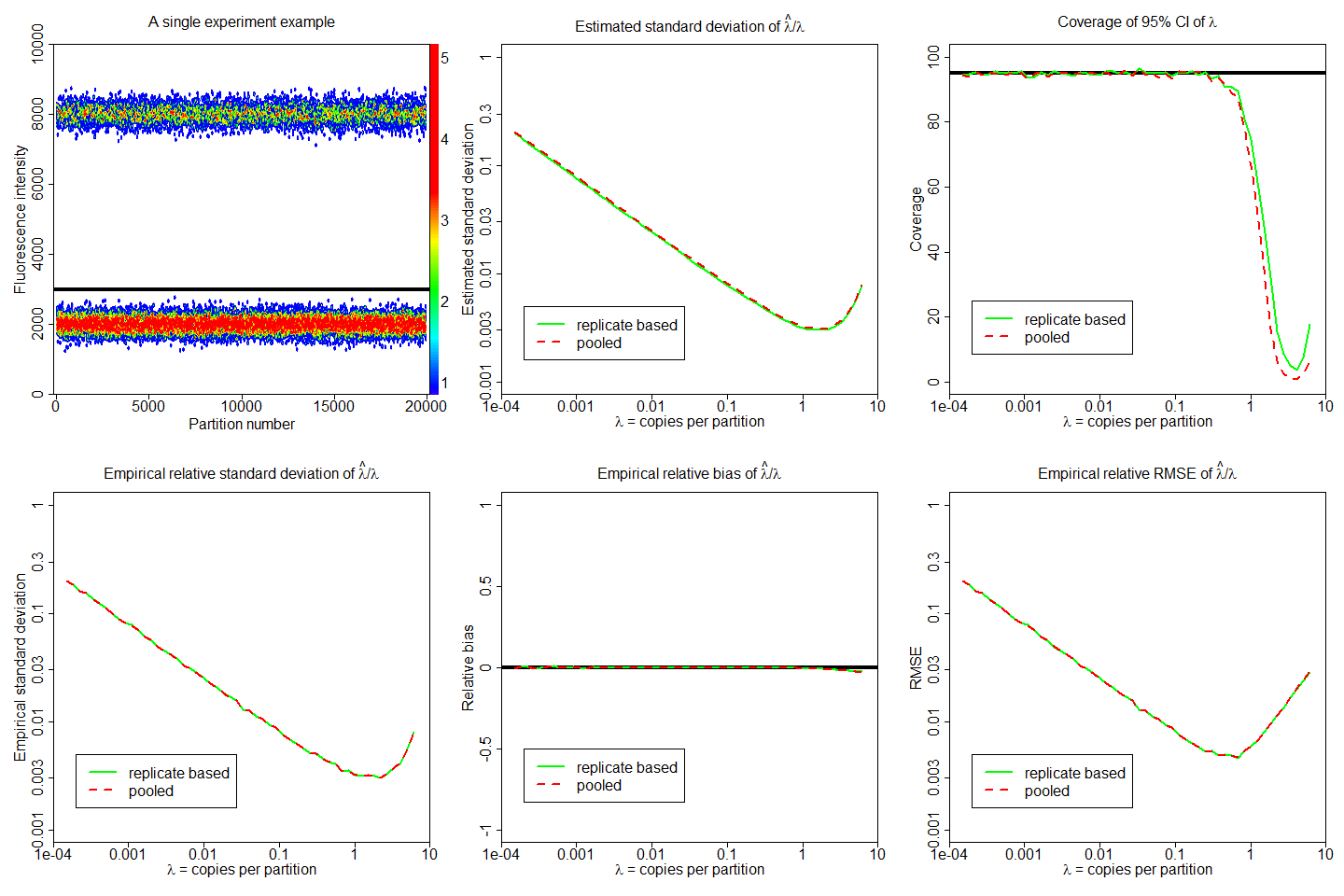

Supplement: Supplementary file 4 — Additional file 4: Interactive tool. In this mini-website, we provide an interactive tool to study the influence of specific sources of variation on the performance of the concentration estimators. This can serve as a guide when designing an experiment. All results are relative to the true concentration and based on 1000 simulations with 8 technical replicates. (ZIP 17 MB) [file 12859_2014_6687_MOESM4_ESM.zip › Additional file 4/RES/RES2161B.png]

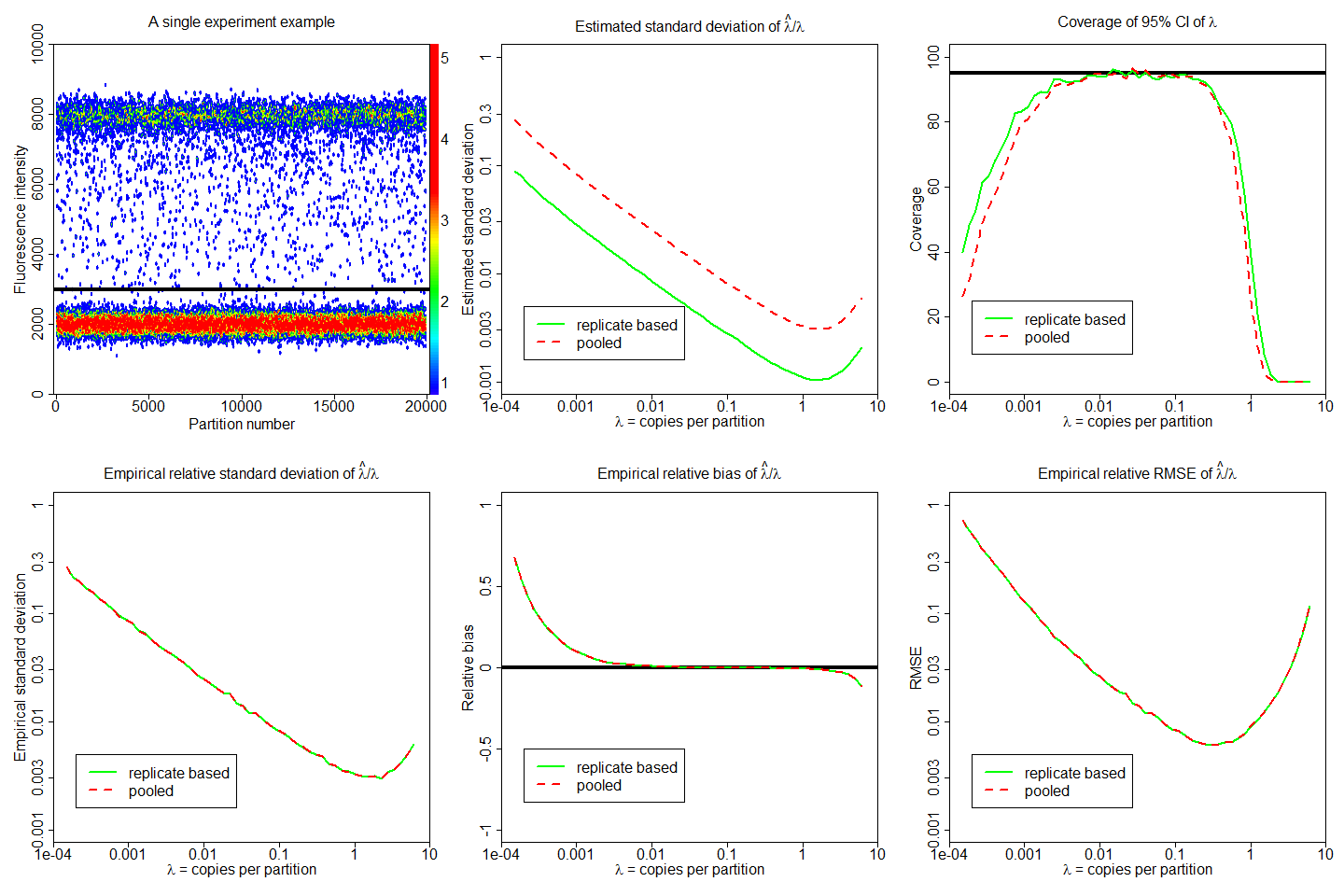

Supplement: Supplementary file 4 — Additional file 4: Interactive tool. In this mini-website, we provide an interactive tool to study the influence of specific sources of variation on the performance of the concentration estimators. This can serve as a guide when designing an experiment. All results are relative to the true concentration and based on 1000 simulations with 8 technical replicates. (ZIP 17 MB) [file 12859_2014_6687_MOESM4_ESM.zip › Additional file 4/RES/RES2162B.png]

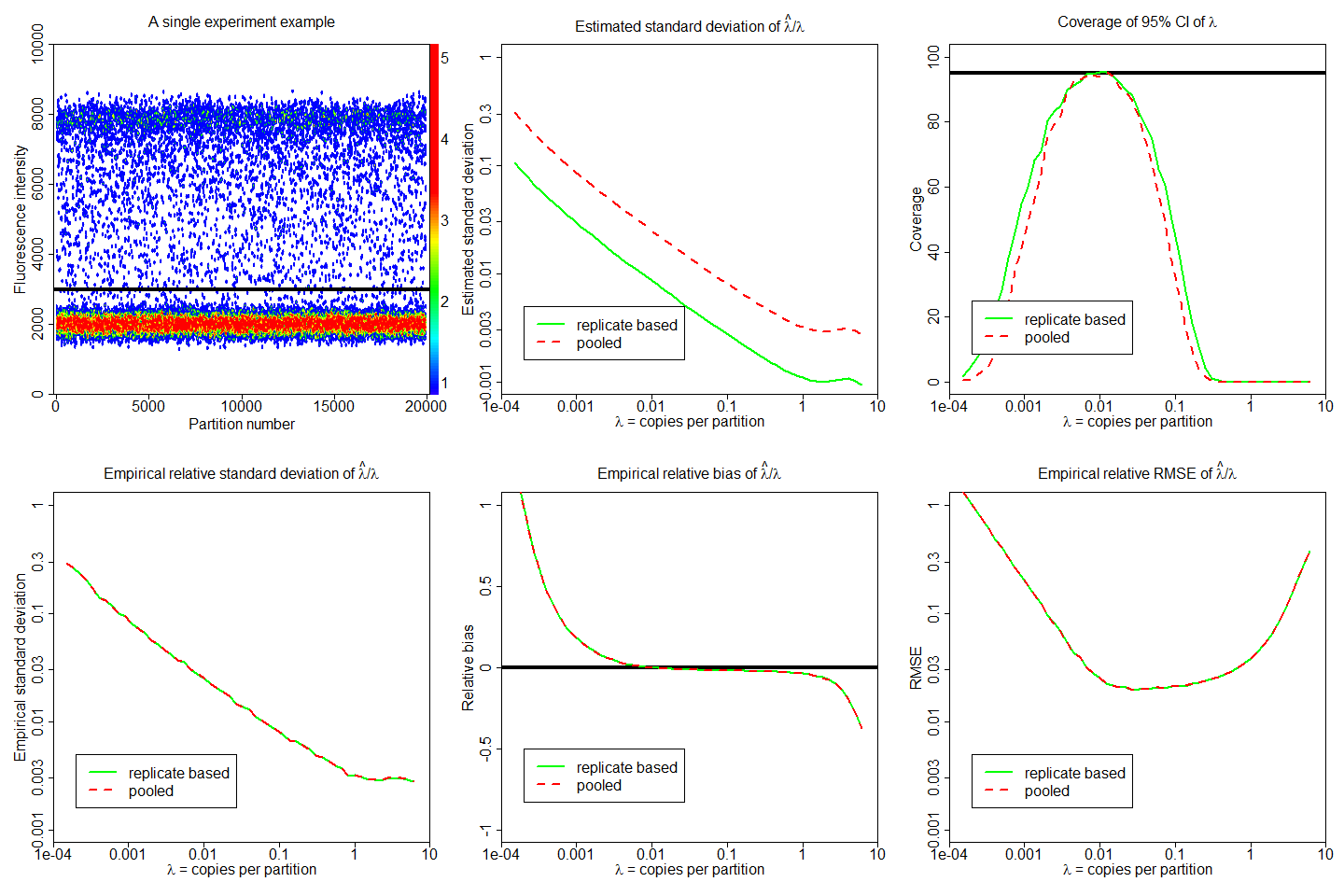

Supplement: Supplementary file 4 — Additional file 4: Interactive tool. In this mini-website, we provide an interactive tool to study the influence of specific sources of variation on the performance of the concentration estimators. This can serve as a guide when designing an experiment. All results are relative to the true concentration and based on 1000 simulations with 8 technical replicates. (ZIP 17 MB) [file 12859_2014_6687_MOESM4_ESM.zip › Additional file 4/RES/RES2163B.png]

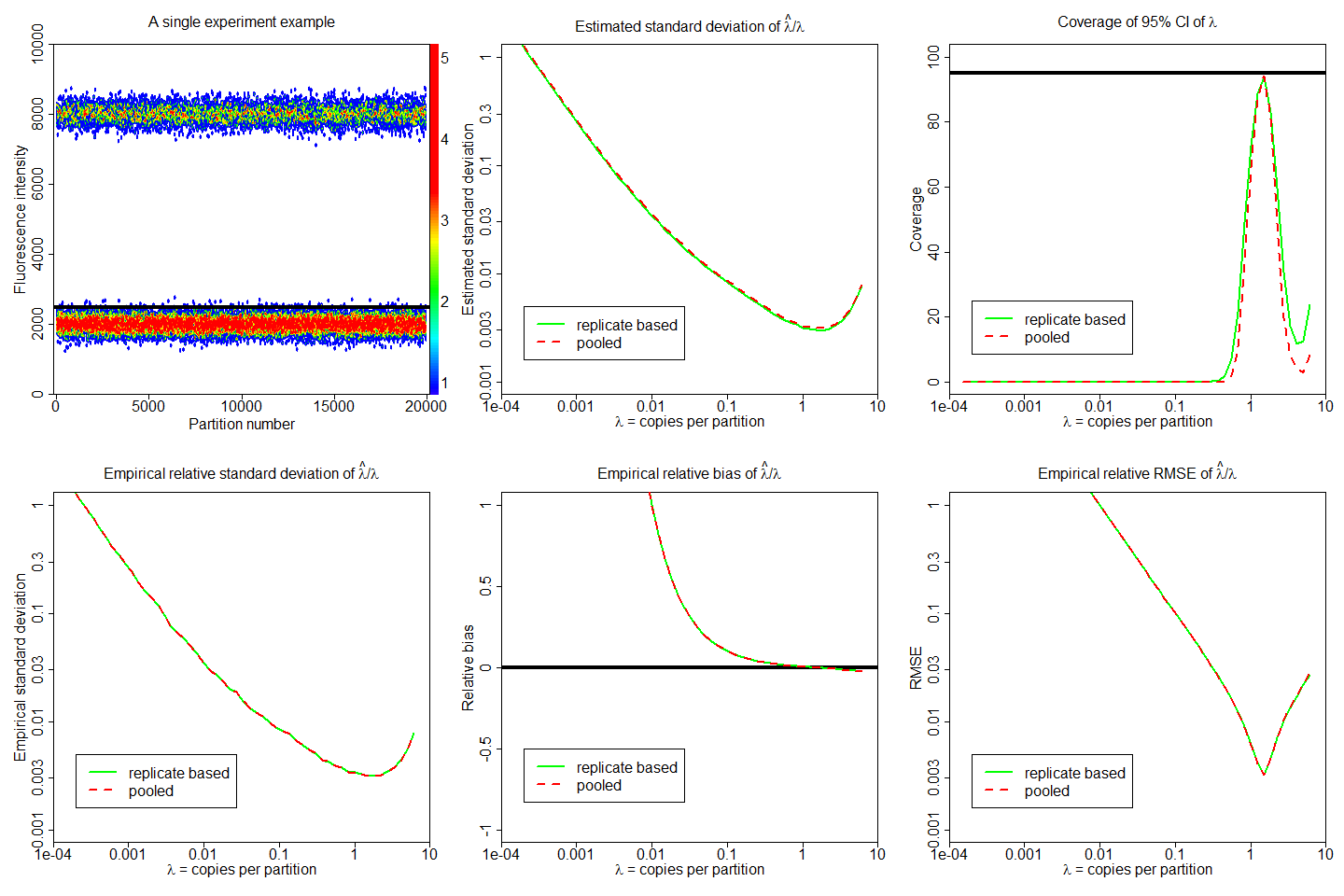

Supplement: Supplementary file 4 — Additional file 4: Interactive tool. In this mini-website, we provide an interactive tool to study the influence of specific sources of variation on the performance of the concentration estimators. This can serve as a guide when designing an experiment. All results are relative to the true concentration and based on 1000 simulations with 8 technical replicates. (ZIP 17 MB) [file 12859_2014_6687_MOESM4_ESM.zip › Additional file 4/RES/RES2171B.png]

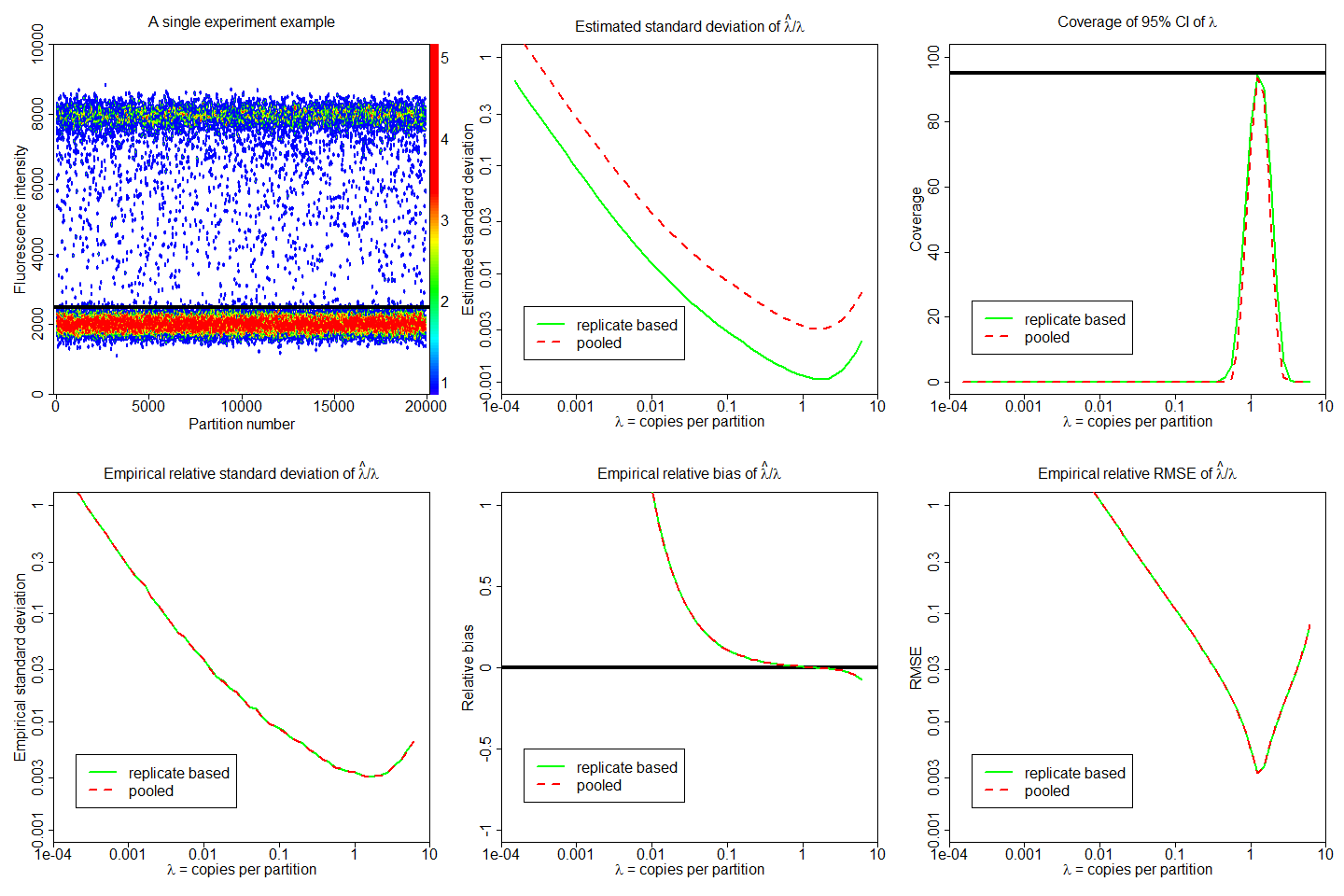

Supplement: Supplementary file 4 — Additional file 4: Interactive tool. In this mini-website, we provide an interactive tool to study the influence of specific sources of variation on the performance of the concentration estimators. This can serve as a guide when designing an experiment. All results are relative to the true concentration and based on 1000 simulations with 8 technical replicates. (ZIP 17 MB) [file 12859_2014_6687_MOESM4_ESM.zip › Additional file 4/RES/RES2172B.png]

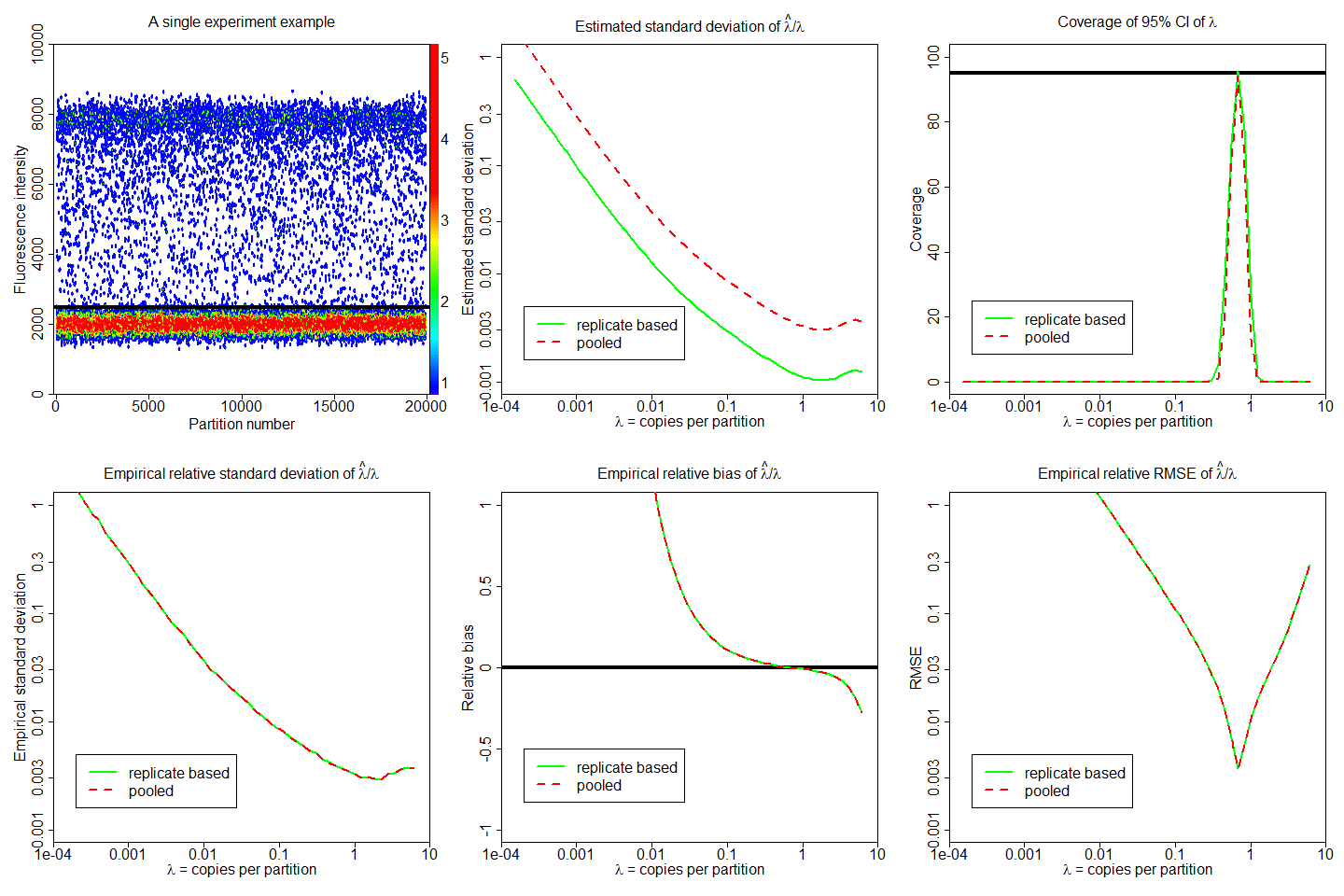

Supplement: Supplementary file 4 — Additional file 4: Interactive tool. In this mini-website, we provide an interactive tool to study the influence of specific sources of variation on the performance of the concentration estimators. This can serve as a guide when designing an experiment. All results are relative to the true concentration and based on 1000 simulations with 8 technical replicates. (ZIP 17 MB) [file 12859_2014_6687_MOESM4_ESM.zip › Additional file 4/RES/RES2173B.png]

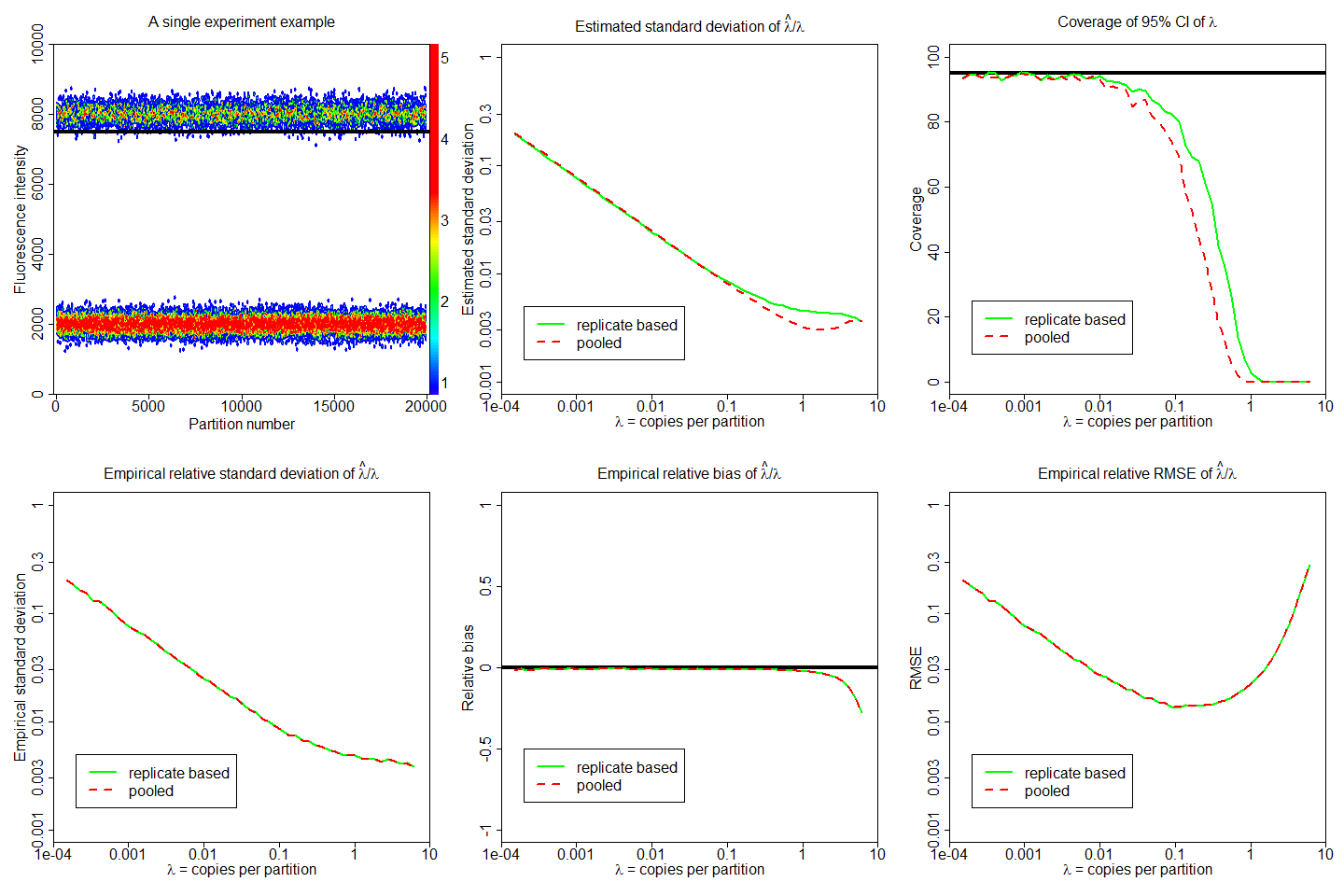

Supplement: Supplementary file 4 — Additional file 4: Interactive tool. In this mini-website, we provide an interactive tool to study the influence of specific sources of variation on the performance of the concentration estimators. This can serve as a guide when designing an experiment. All results are relative to the true concentration and based on 1000 simulations with 8 technical replicates. (ZIP 17 MB) [file 12859_2014_6687_MOESM4_ESM.zip › Additional file 4/RES/RES2211B.png]

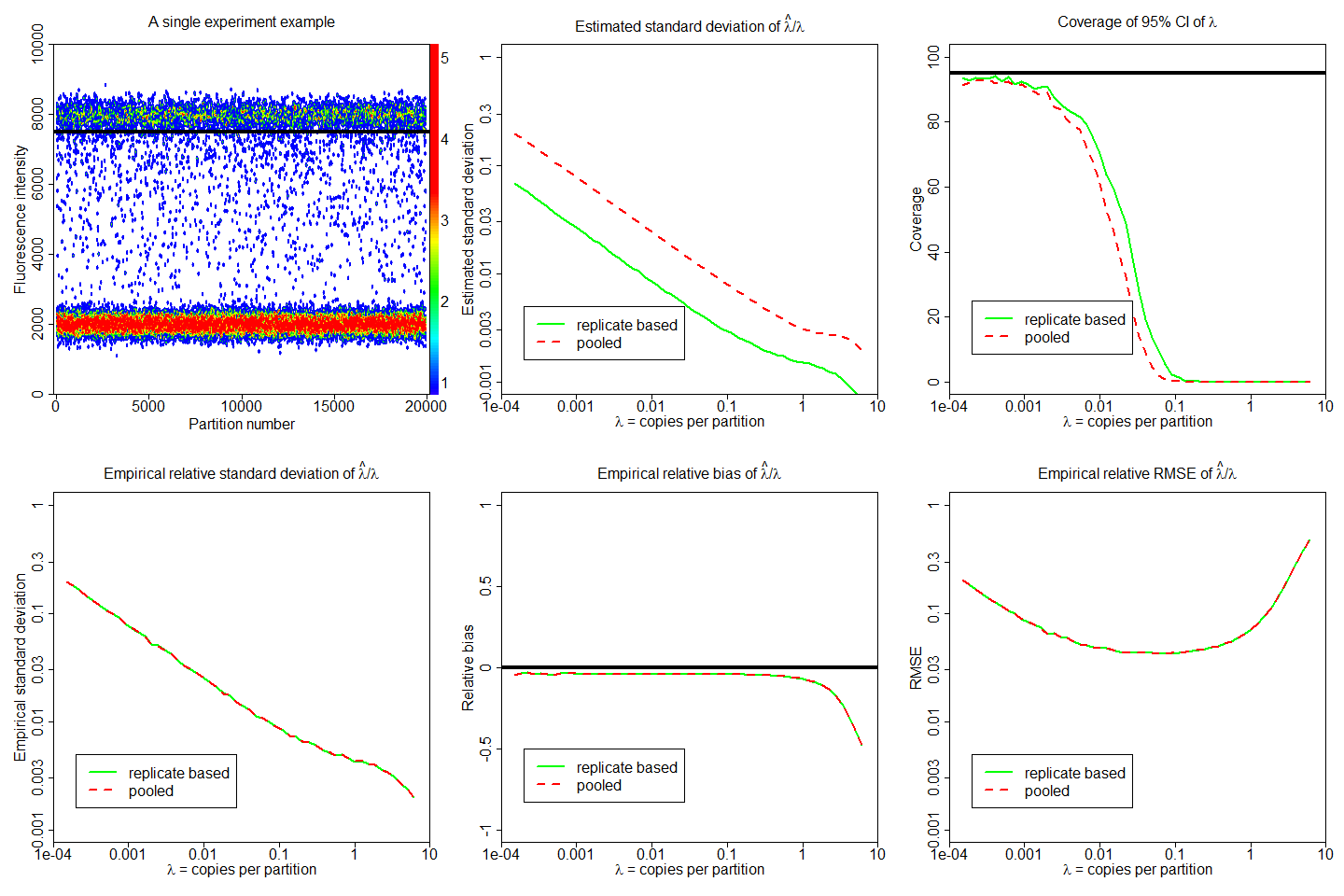

Supplement: Supplementary file 4 — Additional file 4: Interactive tool. In this mini-website, we provide an interactive tool to study the influence of specific sources of variation on the performance of the concentration estimators. This can serve as a guide when designing an experiment. All results are relative to the true concentration and based on 1000 simulations with 8 technical replicates. (ZIP 17 MB) [file 12859_2014_6687_MOESM4_ESM.zip › Additional file 4/RES/RES2212B.png]

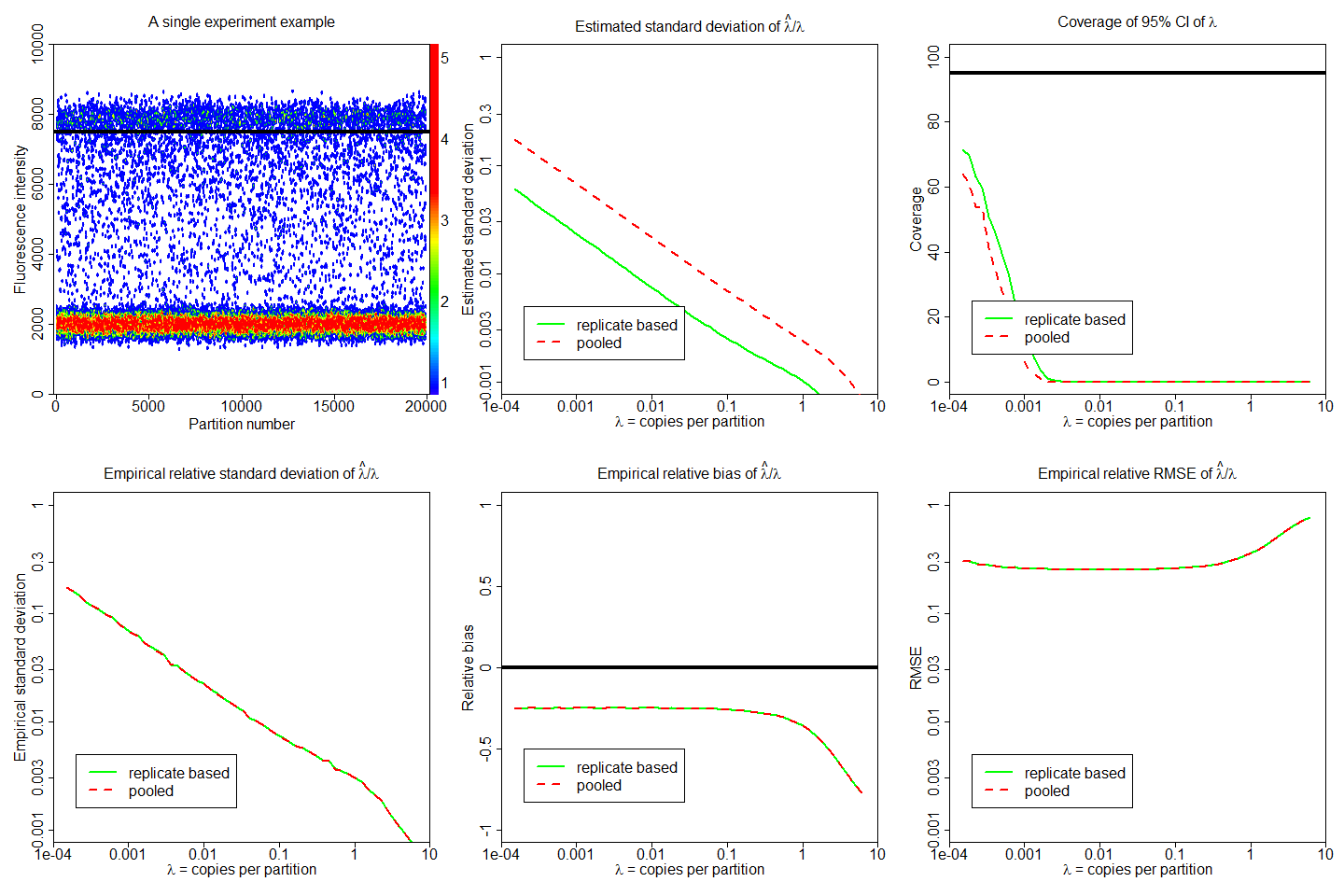

Supplement: Supplementary file 4 — Additional file 4: Interactive tool. In this mini-website, we provide an interactive tool to study the influence of specific sources of variation on the performance of the concentration estimators. This can serve as a guide when designing an experiment. All results are relative to the true concentration and based on 1000 simulations with 8 technical replicates. (ZIP 17 MB) [file 12859_2014_6687_MOESM4_ESM.zip › Additional file 4/RES/RES2213B.png]

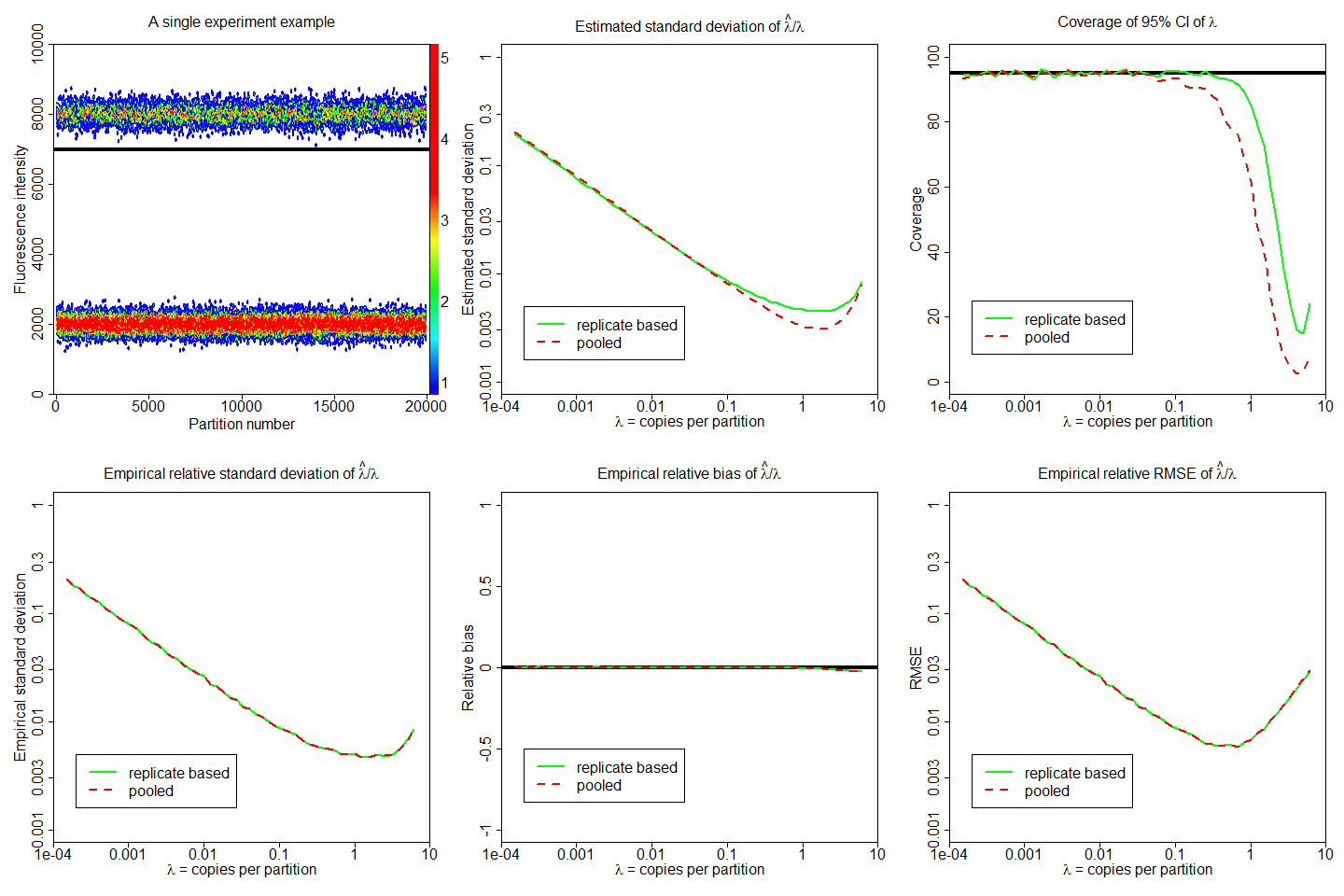

Supplement: Supplementary file 4 — Additional file 4: Interactive tool. In this mini-website, we provide an interactive tool to study the influence of specific sources of variation on the performance of the concentration estimators. This can serve as a guide when designing an experiment. All results are relative to the true concentration and based on 1000 simulations with 8 technical replicates. (ZIP 17 MB) [file 12859_2014_6687_MOESM4_ESM.zip › Additional file 4/RES/RES2221B.png]

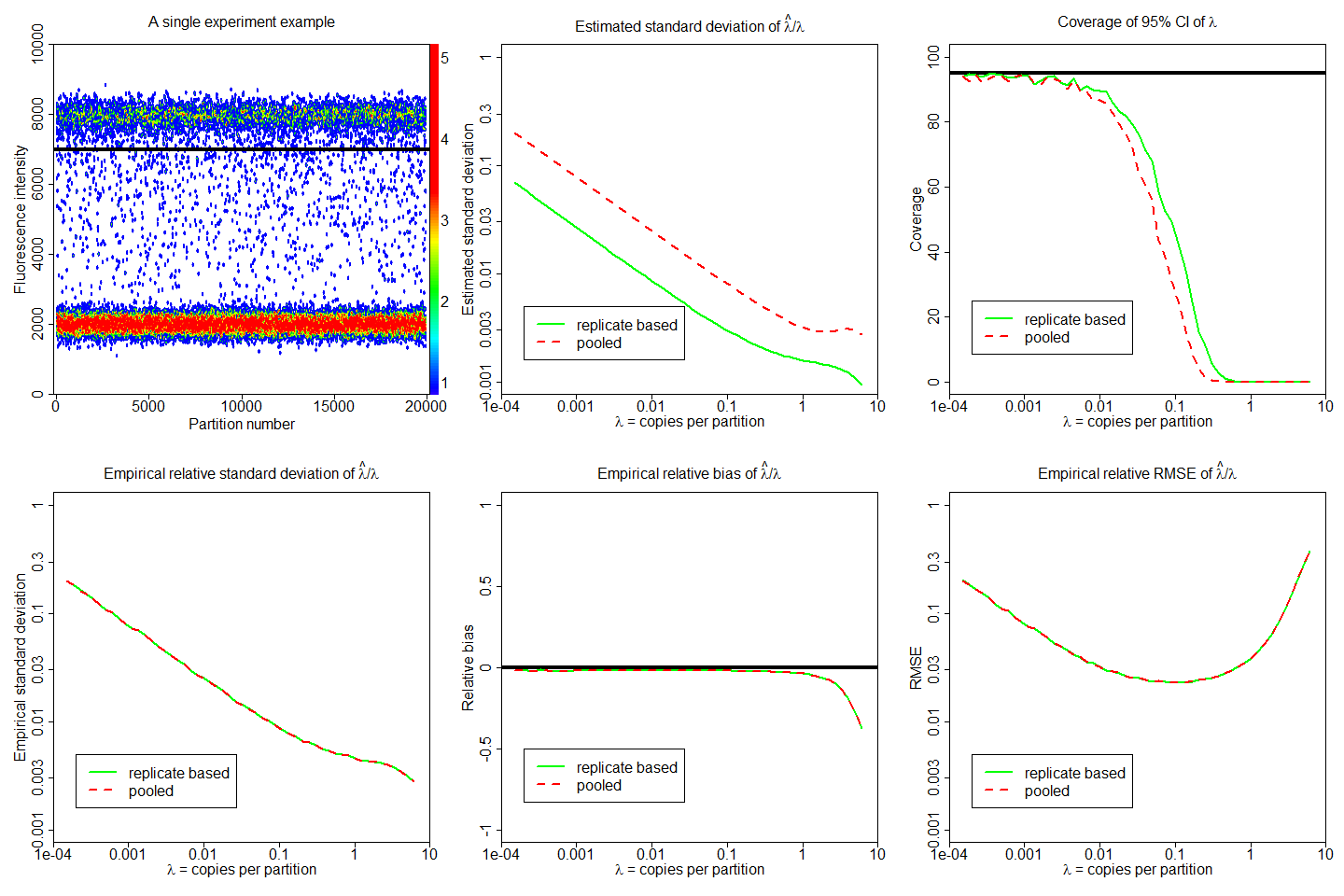

Supplement: Supplementary file 4 — Additional file 4: Interactive tool. In this mini-website, we provide an interactive tool to study the influence of specific sources of variation on the performance of the concentration estimators. This can serve as a guide when designing an experiment. All results are relative to the true concentration and based on 1000 simulations with 8 technical replicates. (ZIP 17 MB) [file 12859_2014_6687_MOESM4_ESM.zip › Additional file 4/RES/RES2222B.png]

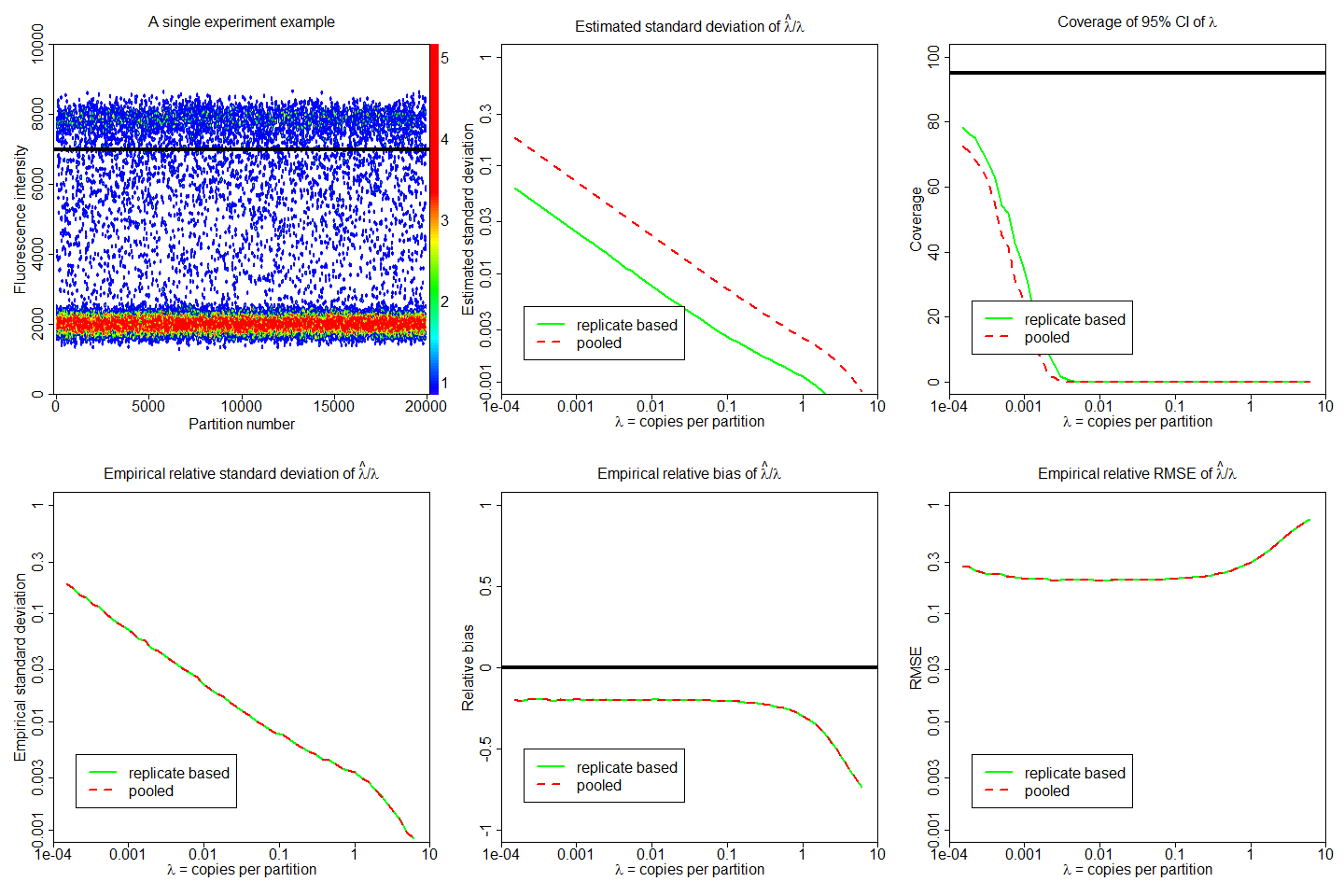

Supplement: Supplementary file 4 — Additional file 4: Interactive tool. In this mini-website, we provide an interactive tool to study the influence of specific sources of variation on the performance of the concentration estimators. This can serve as a guide when designing an experiment. All results are relative to the true concentration and based on 1000 simulations with 8 technical replicates. (ZIP 17 MB) [file 12859_2014_6687_MOESM4_ESM.zip › Additional file 4/RES/RES2223B.png]

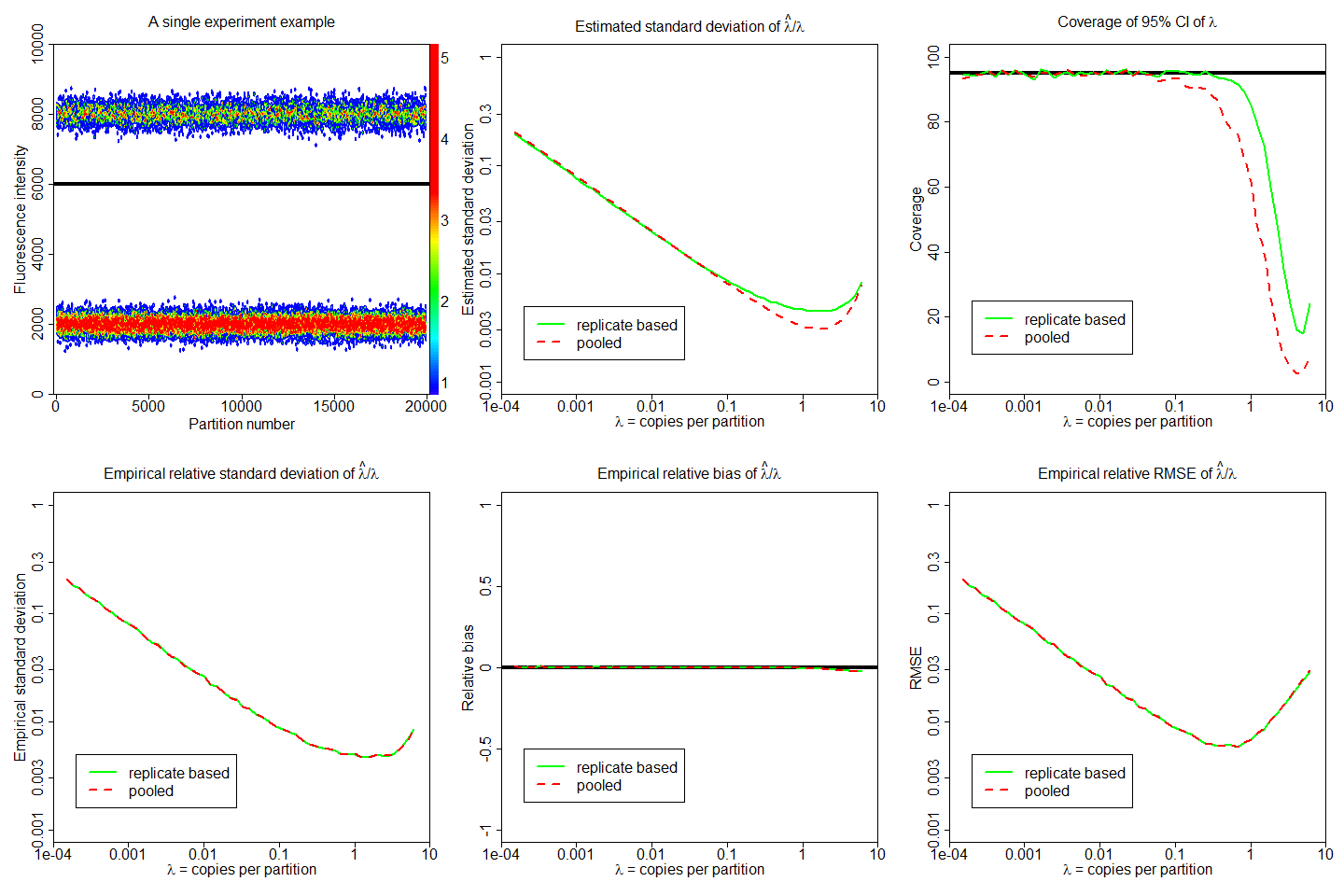

Supplement: Supplementary file 4 — Additional file 4: Interactive tool. In this mini-website, we provide an interactive tool to study the influence of specific sources of variation on the performance of the concentration estimators. This can serve as a guide when designing an experiment. All results are relative to the true concentration and based on 1000 simulations with 8 technical replicates. (ZIP 17 MB) [file 12859_2014_6687_MOESM4_ESM.zip › Additional file 4/RES/RES2231B.png]

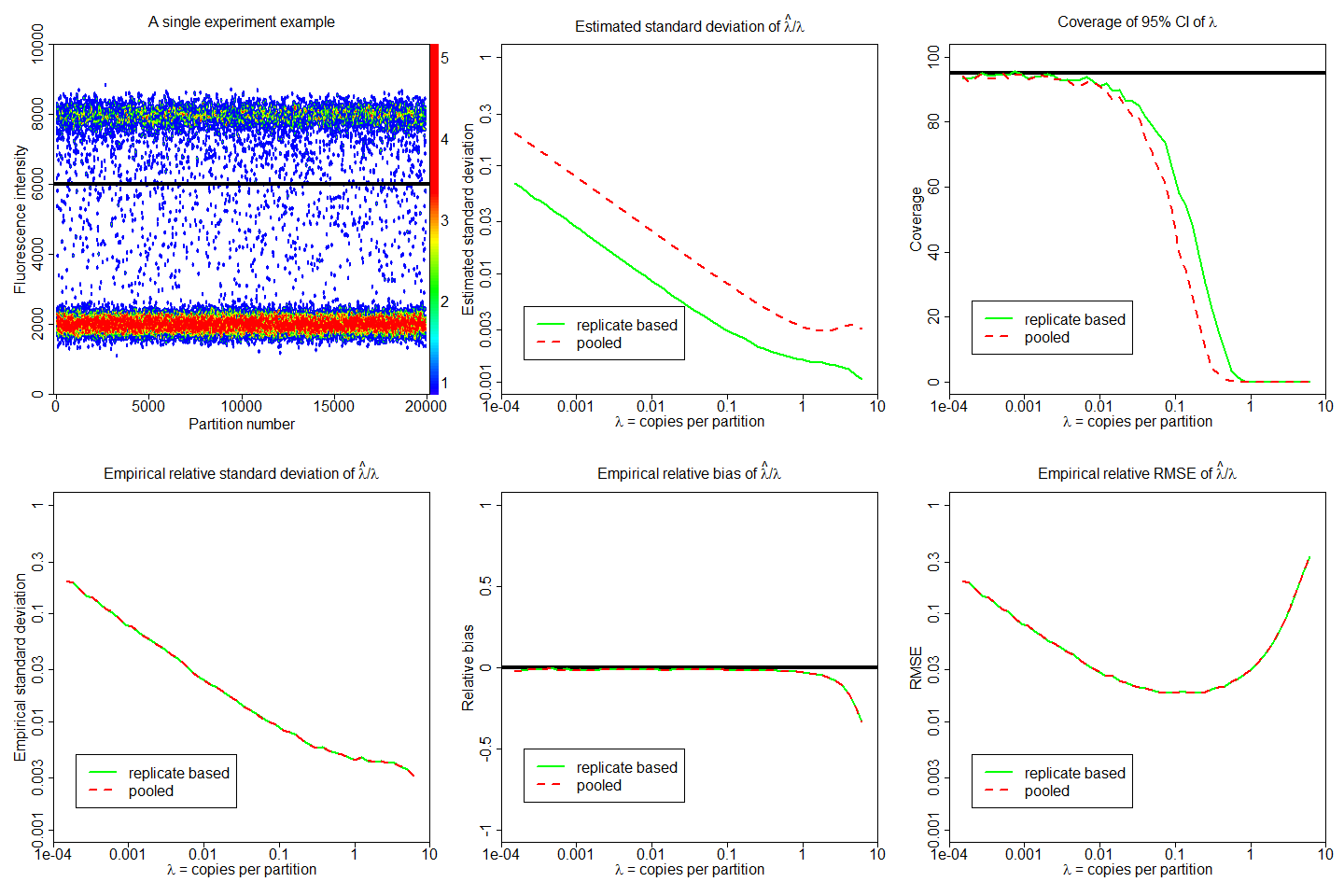

Supplement: Supplementary file 4 — Additional file 4: Interactive tool. In this mini-website, we provide an interactive tool to study the influence of specific sources of variation on the performance of the concentration estimators. This can serve as a guide when designing an experiment. All results are relative to the true concentration and based on 1000 simulations with 8 technical replicates. (ZIP 17 MB) [file 12859_2014_6687_MOESM4_ESM.zip › Additional file 4/RES/RES2232B.png]

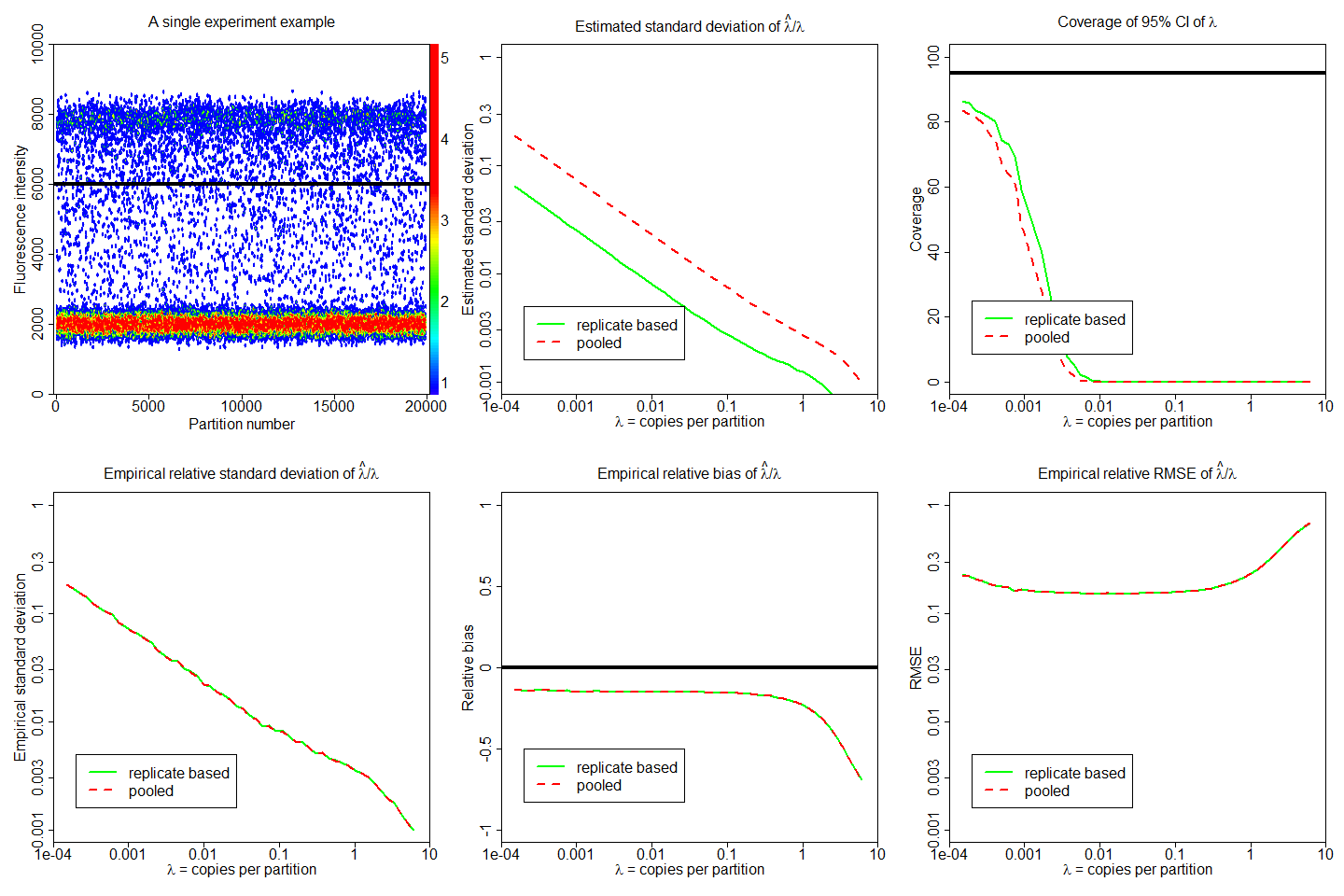

Supplement: Supplementary file 4 — Additional file 4: Interactive tool. In this mini-website, we provide an interactive tool to study the influence of specific sources of variation on the performance of the concentration estimators. This can serve as a guide when designing an experiment. All results are relative to the true concentration and based on 1000 simulations with 8 technical replicates. (ZIP 17 MB) [file 12859_2014_6687_MOESM4_ESM.zip › Additional file 4/RES/RES2233B.png]

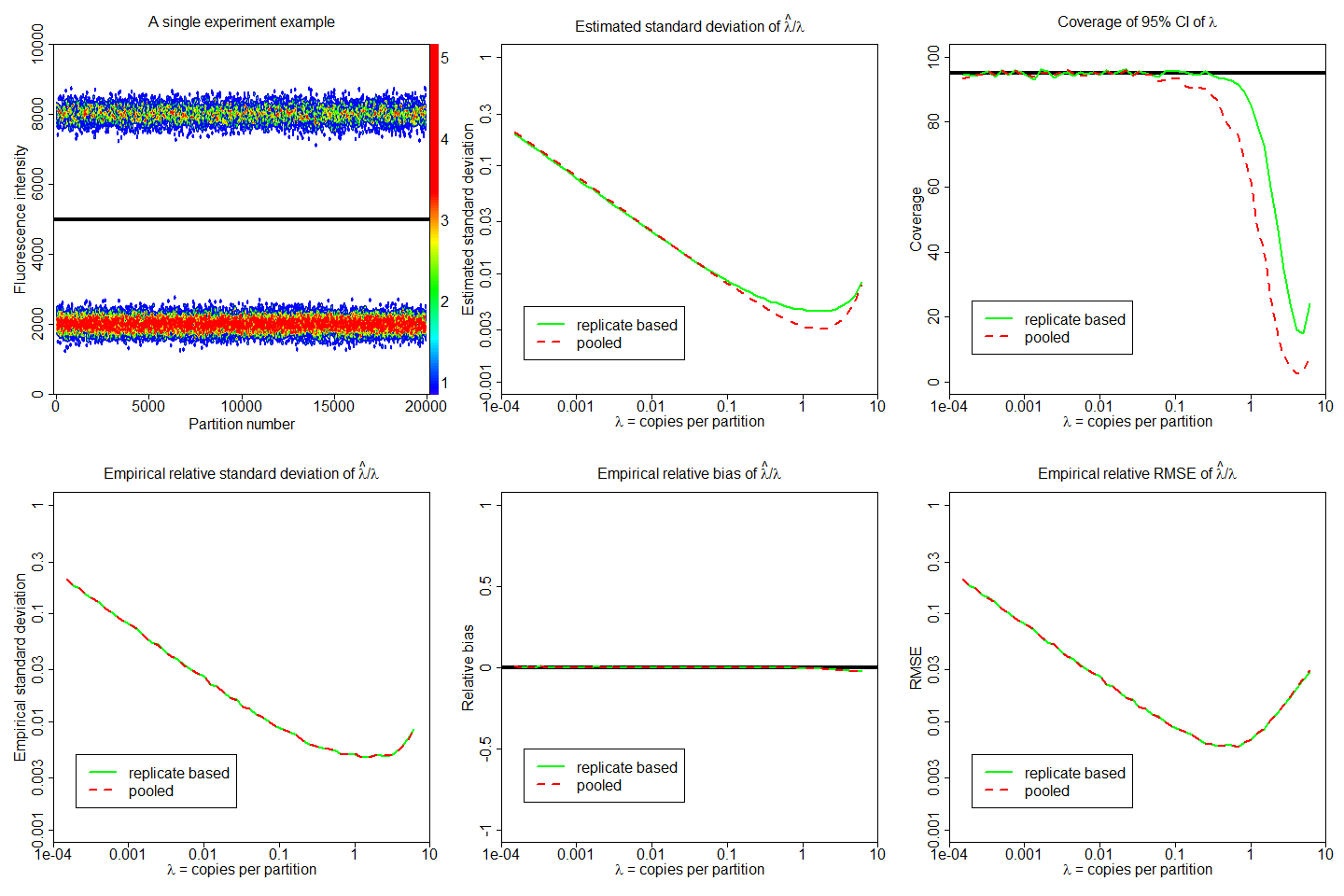

Supplement: Supplementary file 4 — Additional file 4: Interactive tool. In this mini-website, we provide an interactive tool to study the influence of specific sources of variation on the performance of the concentration estimators. This can serve as a guide when designing an experiment. All results are relative to the true concentration and based on 1000 simulations with 8 technical replicates. (ZIP 17 MB) [file 12859_2014_6687_MOESM4_ESM.zip › Additional file 4/RES/RES2241B.png]

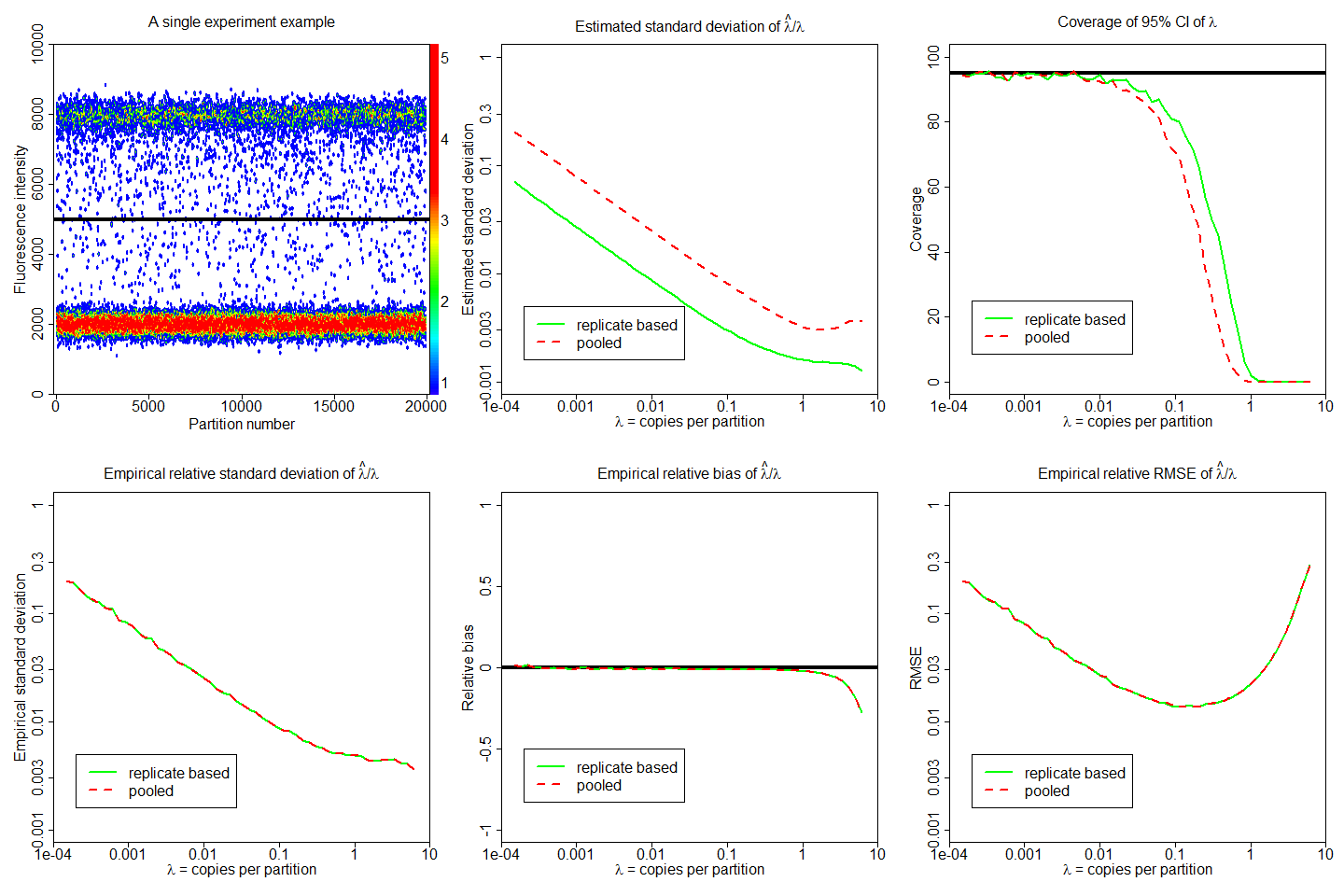

Supplement: Supplementary file 4 — Additional file 4: Interactive tool. In this mini-website, we provide an interactive tool to study the influence of specific sources of variation on the performance of the concentration estimators. This can serve as a guide when designing an experiment. All results are relative to the true concentration and based on 1000 simulations with 8 technical replicates. (ZIP 17 MB) [file 12859_2014_6687_MOESM4_ESM.zip › Additional file 4/RES/RES2242B.png]

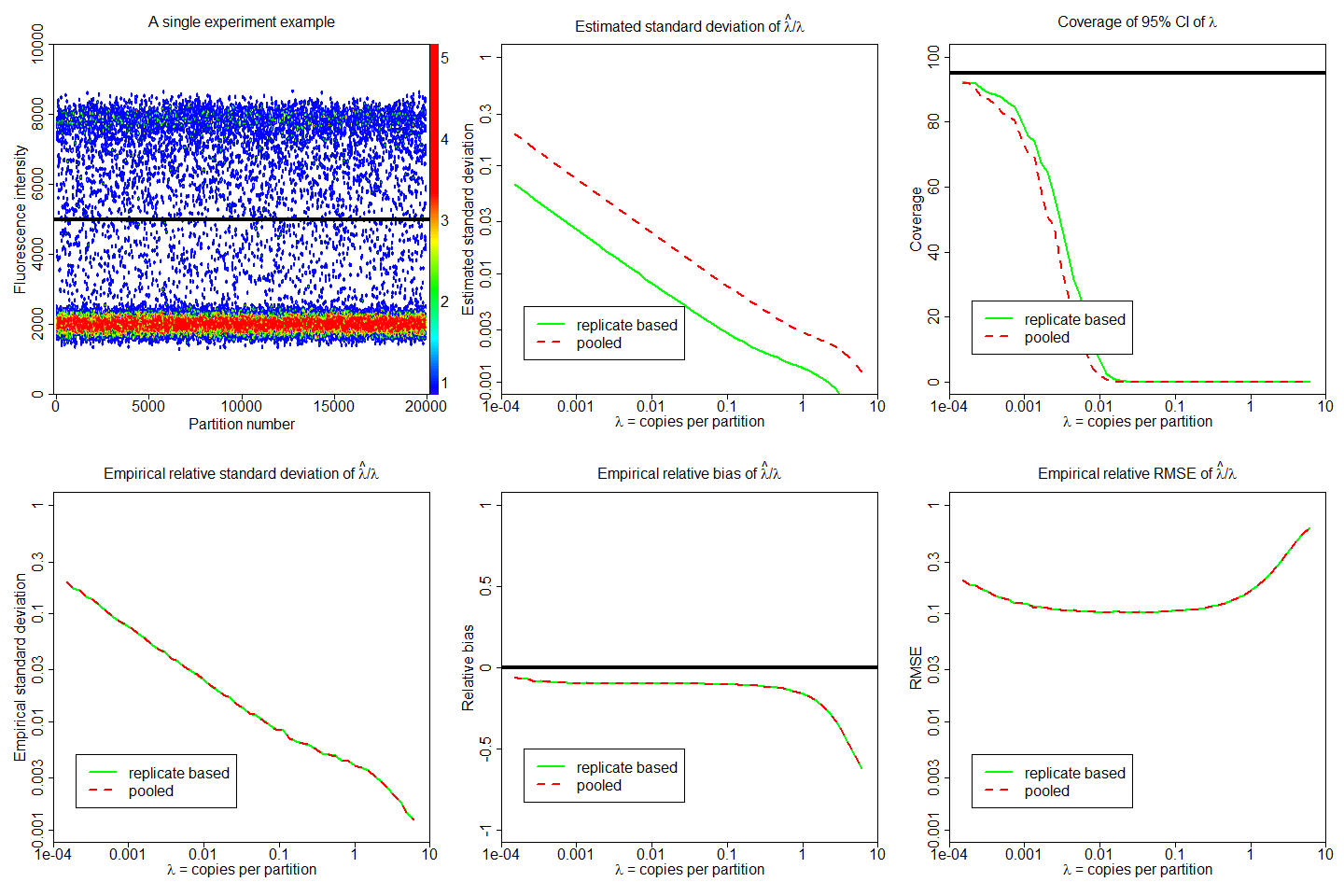

Supplement: Supplementary file 4 — Additional file 4: Interactive tool. In this mini-website, we provide an interactive tool to study the influence of specific sources of variation on the performance of the concentration estimators. This can serve as a guide when designing an experiment. All results are relative to the true concentration and based on 1000 simulations with 8 technical replicates. (ZIP 17 MB) [file 12859_2014_6687_MOESM4_ESM.zip › Additional file 4/RES/RES2243B.png]

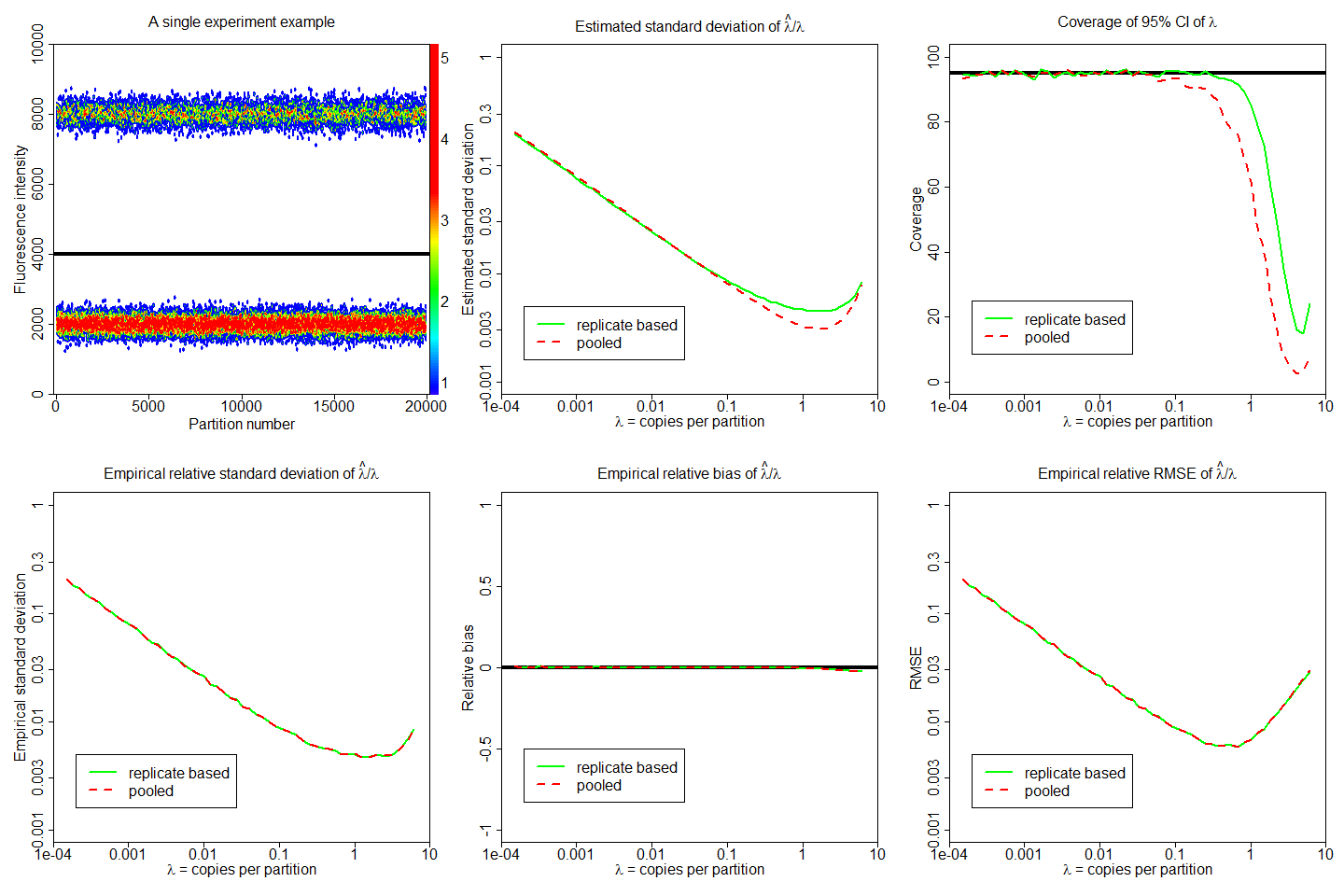

Supplement: Supplementary file 4 — Additional file 4: Interactive tool. In this mini-website, we provide an interactive tool to study the influence of specific sources of variation on the performance of the concentration estimators. This can serve as a guide when designing an experiment. All results are relative to the true concentration and based on 1000 simulations with 8 technical replicates. (ZIP 17 MB) [file 12859_2014_6687_MOESM4_ESM.zip › Additional file 4/RES/RES2251B.png]

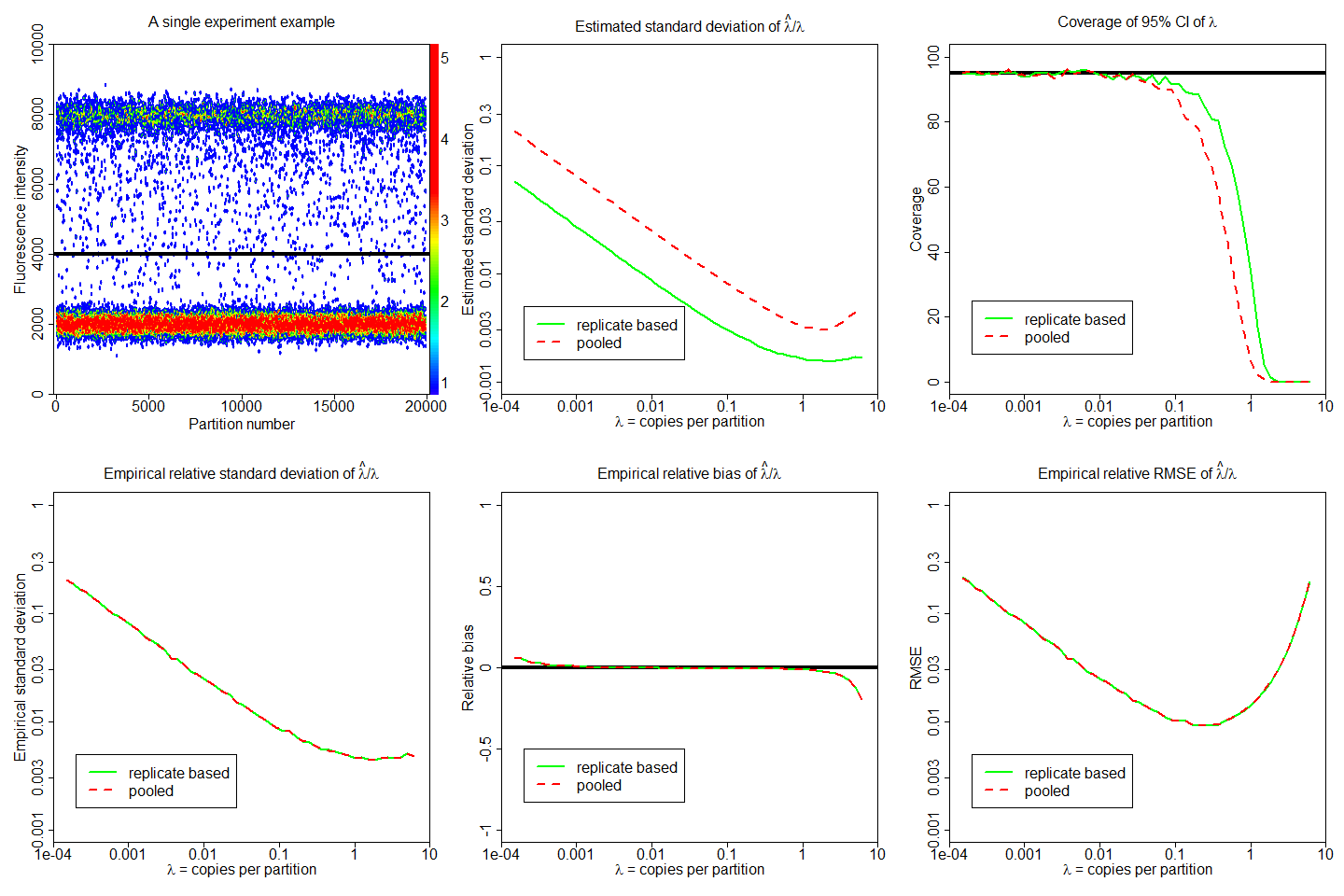

Supplement: Supplementary file 4 — Additional file 4: Interactive tool. In this mini-website, we provide an interactive tool to study the influence of specific sources of variation on the performance of the concentration estimators. This can serve as a guide when designing an experiment. All results are relative to the true concentration and based on 1000 simulations with 8 technical replicates. (ZIP 17 MB) [file 12859_2014_6687_MOESM4_ESM.zip › Additional file 4/RES/RES2252B.png]

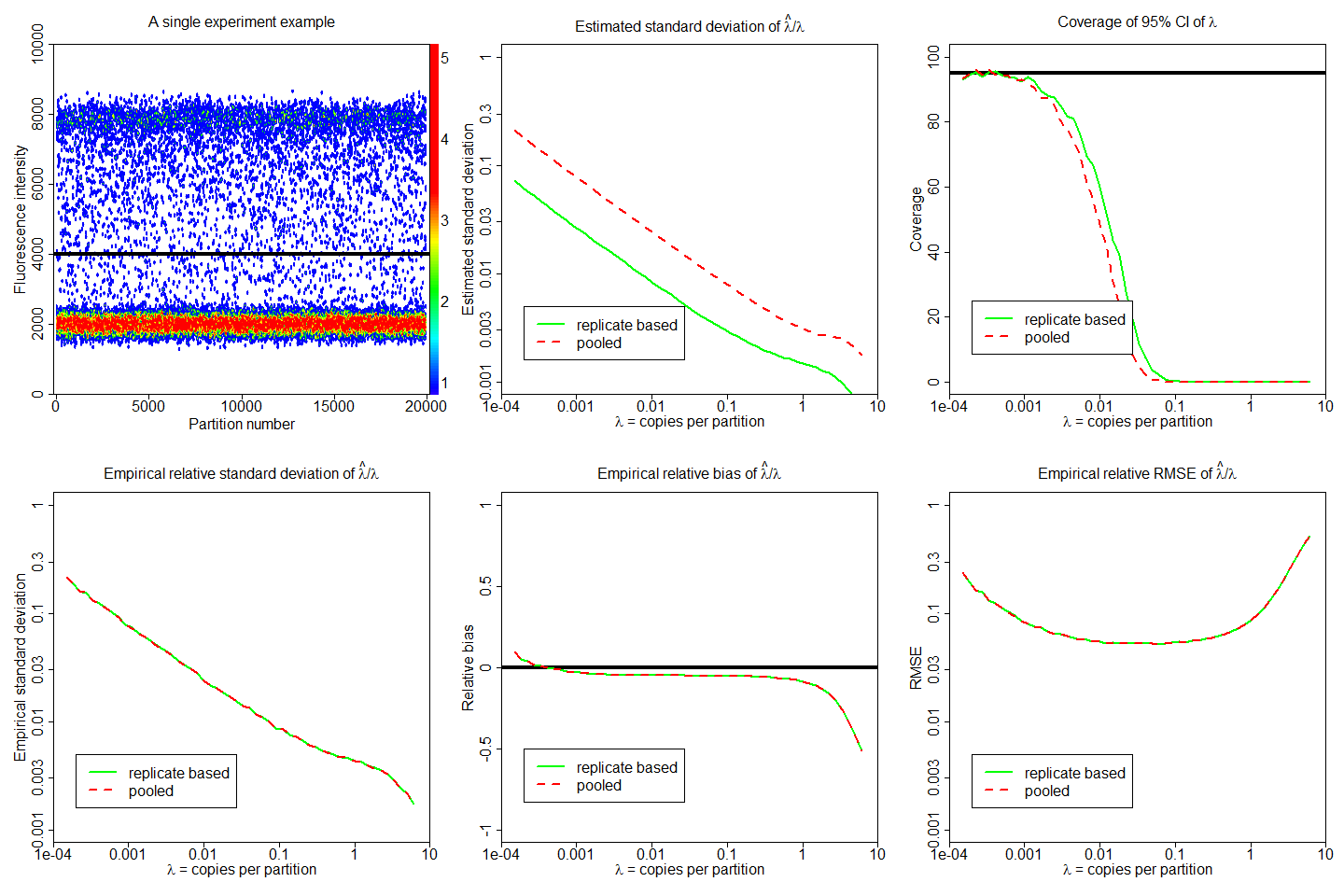

Supplement: Supplementary file 4 — Additional file 4: Interactive tool. In this mini-website, we provide an interactive tool to study the influence of specific sources of variation on the performance of the concentration estimators. This can serve as a guide when designing an experiment. All results are relative to the true concentration and based on 1000 simulations with 8 technical replicates. (ZIP 17 MB) [file 12859_2014_6687_MOESM4_ESM.zip › Additional file 4/RES/RES2253B.png]

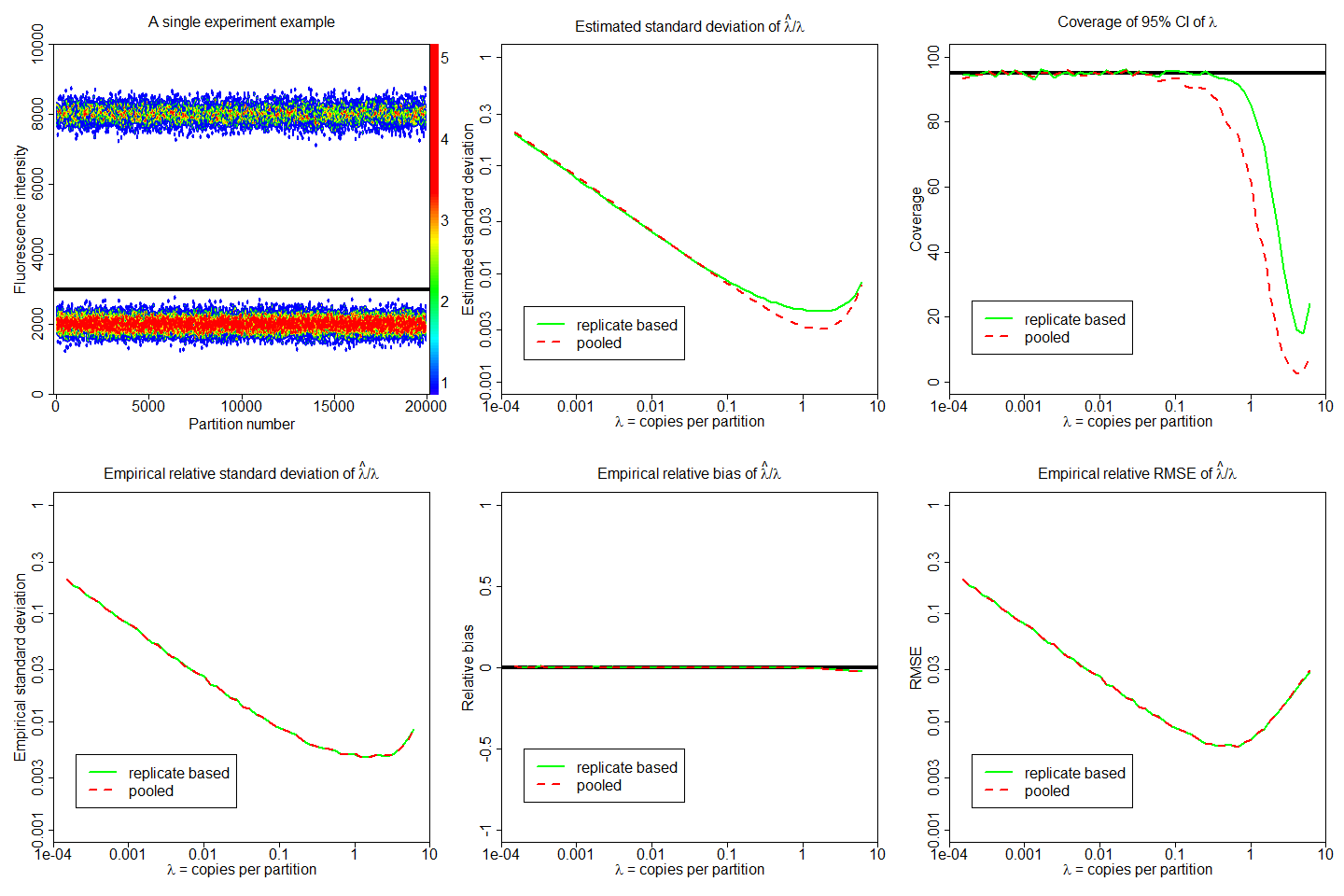

Supplement: Supplementary file 4 — Additional file 4: Interactive tool. In this mini-website, we provide an interactive tool to study the influence of specific sources of variation on the performance of the concentration estimators. This can serve as a guide when designing an experiment. All results are relative to the true concentration and based on 1000 simulations with 8 technical replicates. (ZIP 17 MB) [file 12859_2014_6687_MOESM4_ESM.zip › Additional file 4/RES/RES2261B.png]

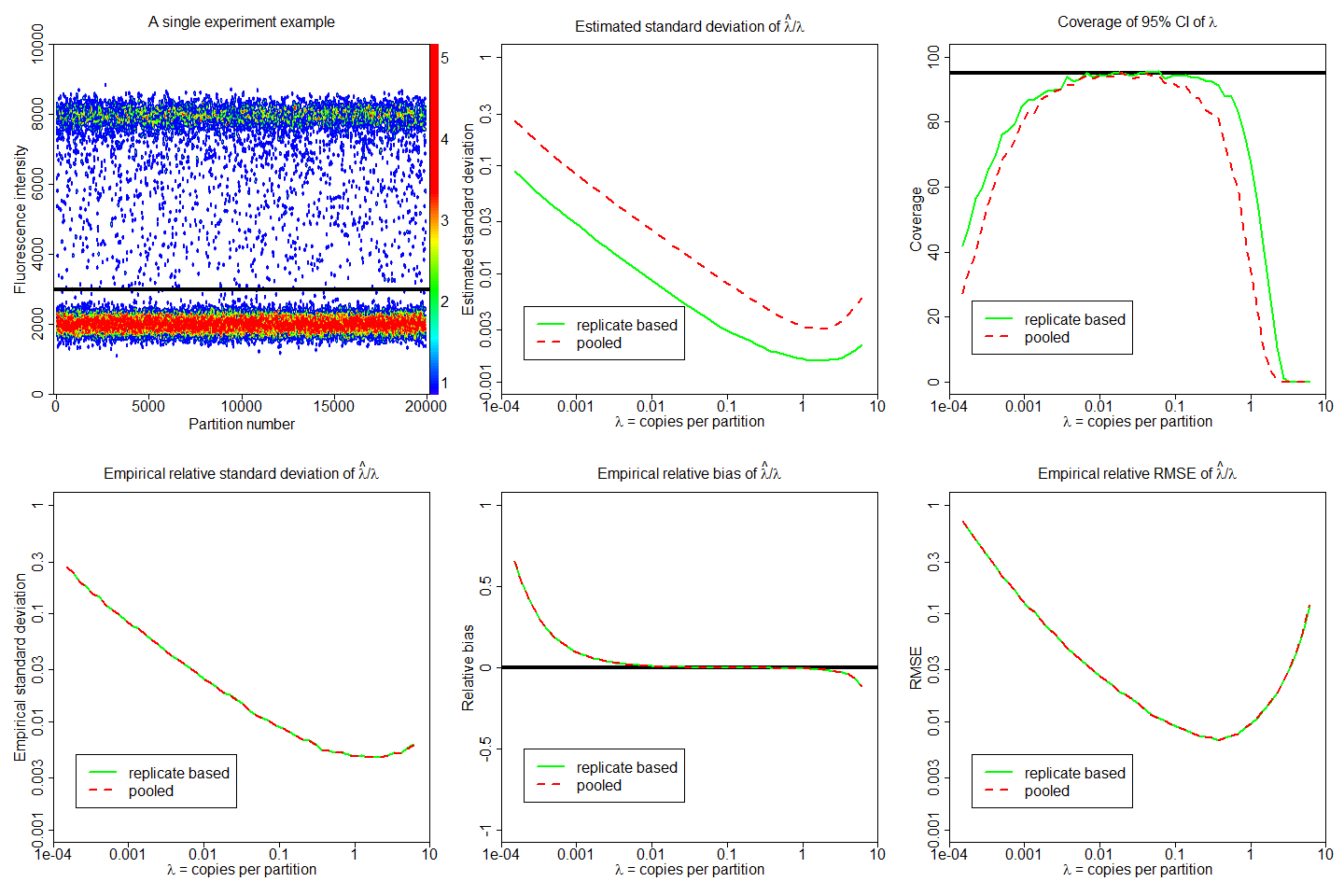

Supplement: Supplementary file 4 — Additional file 4: Interactive tool. In this mini-website, we provide an interactive tool to study the influence of specific sources of variation on the performance of the concentration estimators. This can serve as a guide when designing an experiment. All results are relative to the true concentration and based on 1000 simulations with 8 technical replicates. (ZIP 17 MB) [file 12859_2014_6687_MOESM4_ESM.zip › Additional file 4/RES/RES2262B.png]

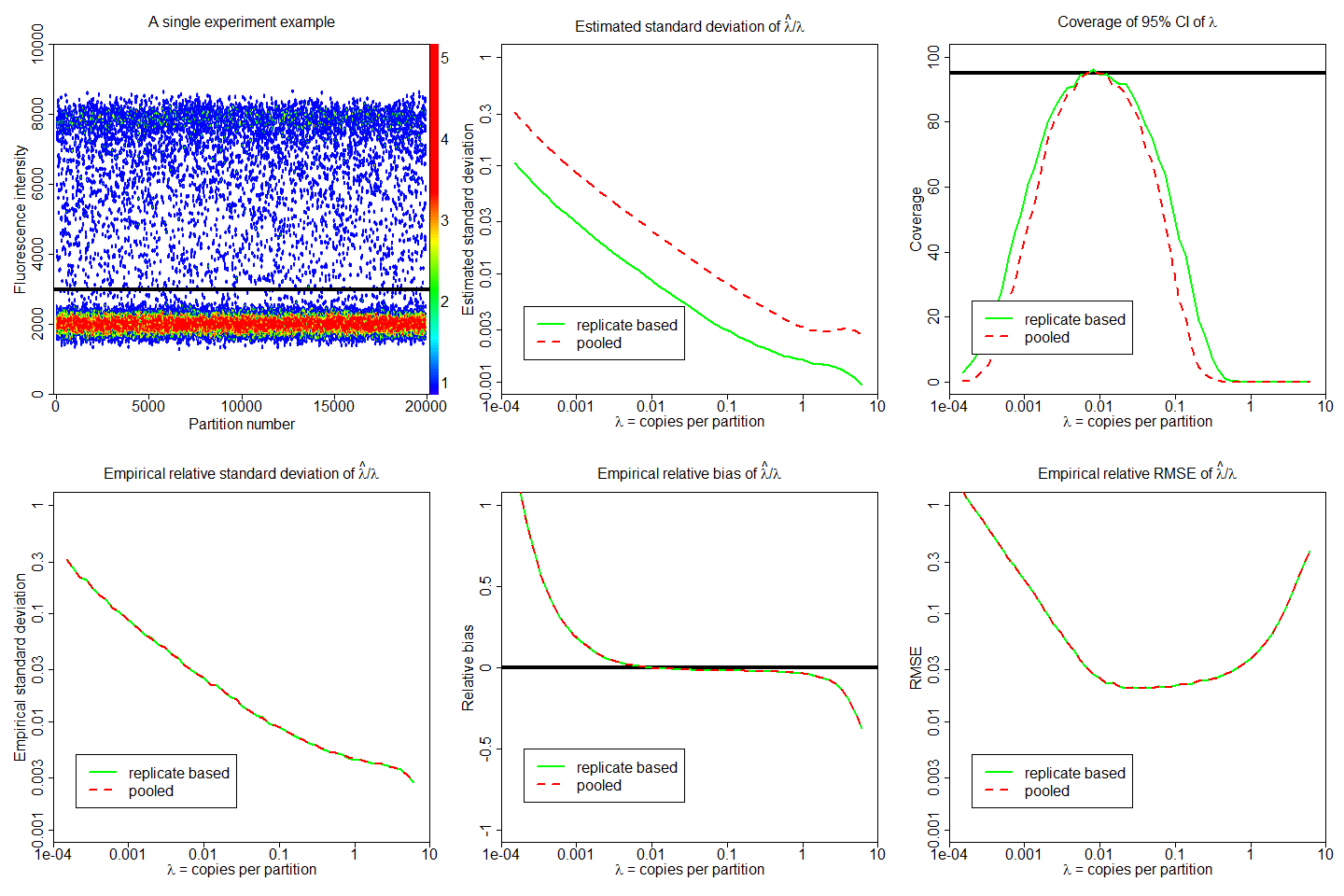

Supplement: Supplementary file 4 — Additional file 4: Interactive tool. In this mini-website, we provide an interactive tool to study the influence of specific sources of variation on the performance of the concentration estimators. This can serve as a guide when designing an experiment. All results are relative to the true concentration and based on 1000 simulations with 8 technical replicates. (ZIP 17 MB) [file 12859_2014_6687_MOESM4_ESM.zip › Additional file 4/RES/RES2263B.png]

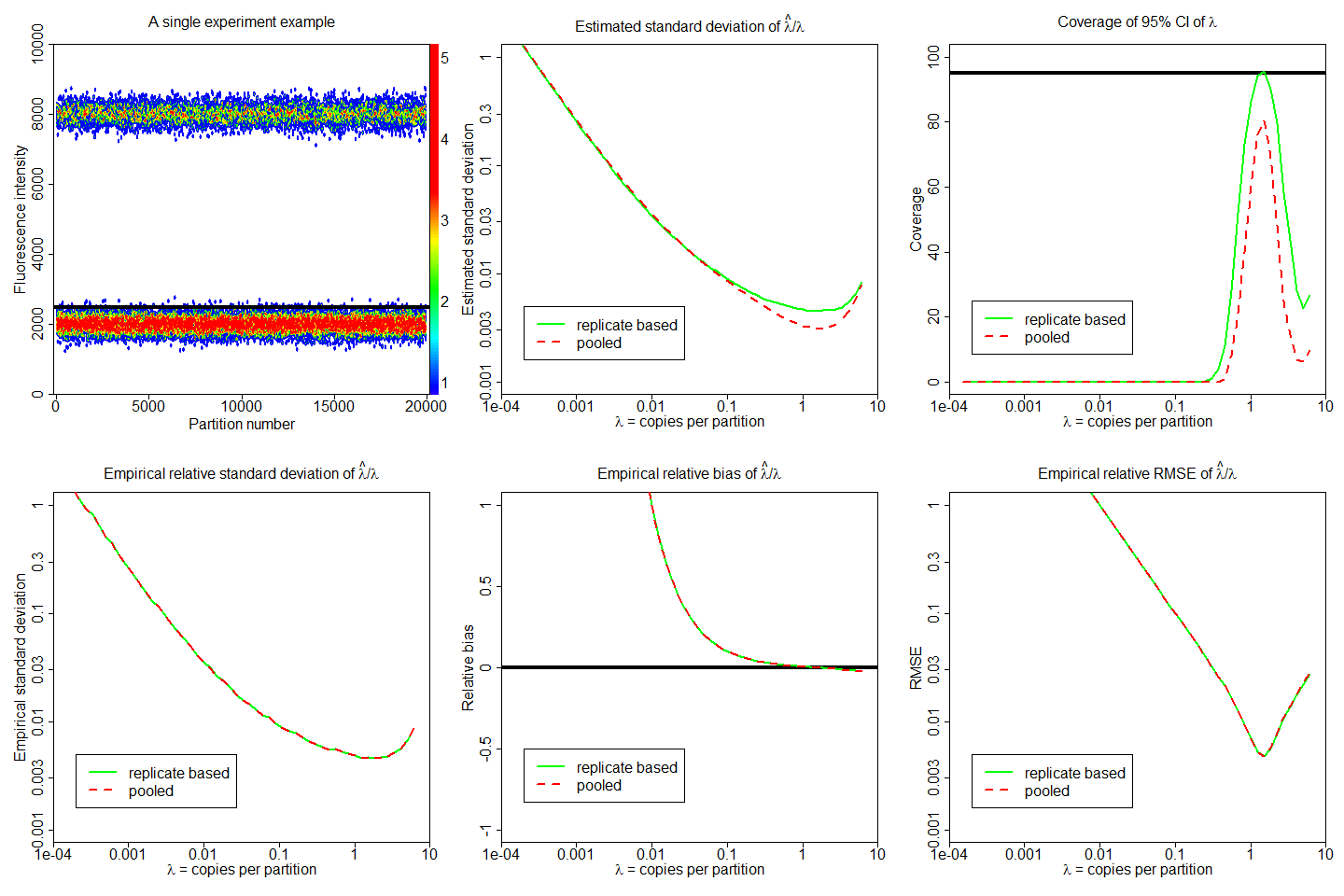

Supplement: Supplementary file 4 — Additional file 4: Interactive tool. In this mini-website, we provide an interactive tool to study the influence of specific sources of variation on the performance of the concentration estimators. This can serve as a guide when designing an experiment. All results are relative to the true concentration and based on 1000 simulations with 8 technical replicates. (ZIP 17 MB) [file 12859_2014_6687_MOESM4_ESM.zip › Additional file 4/RES/RES2271B.png]

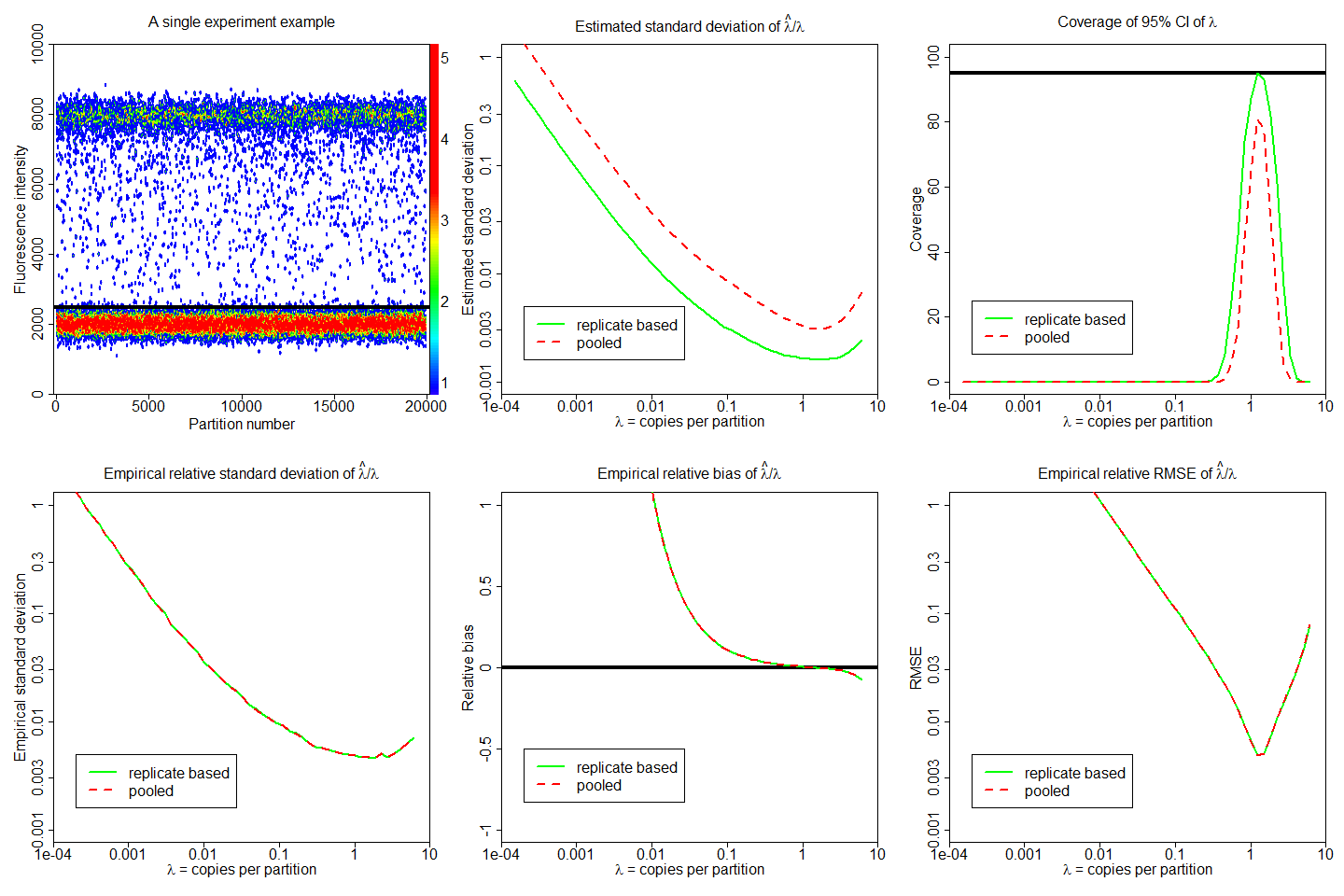

Supplement: Supplementary file 4 — Additional file 4: Interactive tool. In this mini-website, we provide an interactive tool to study the influence of specific sources of variation on the performance of the concentration estimators. This can serve as a guide when designing an experiment. All results are relative to the true concentration and based on 1000 simulations with 8 technical replicates. (ZIP 17 MB) [file 12859_2014_6687_MOESM4_ESM.zip › Additional file 4/RES/RES2272B.png]

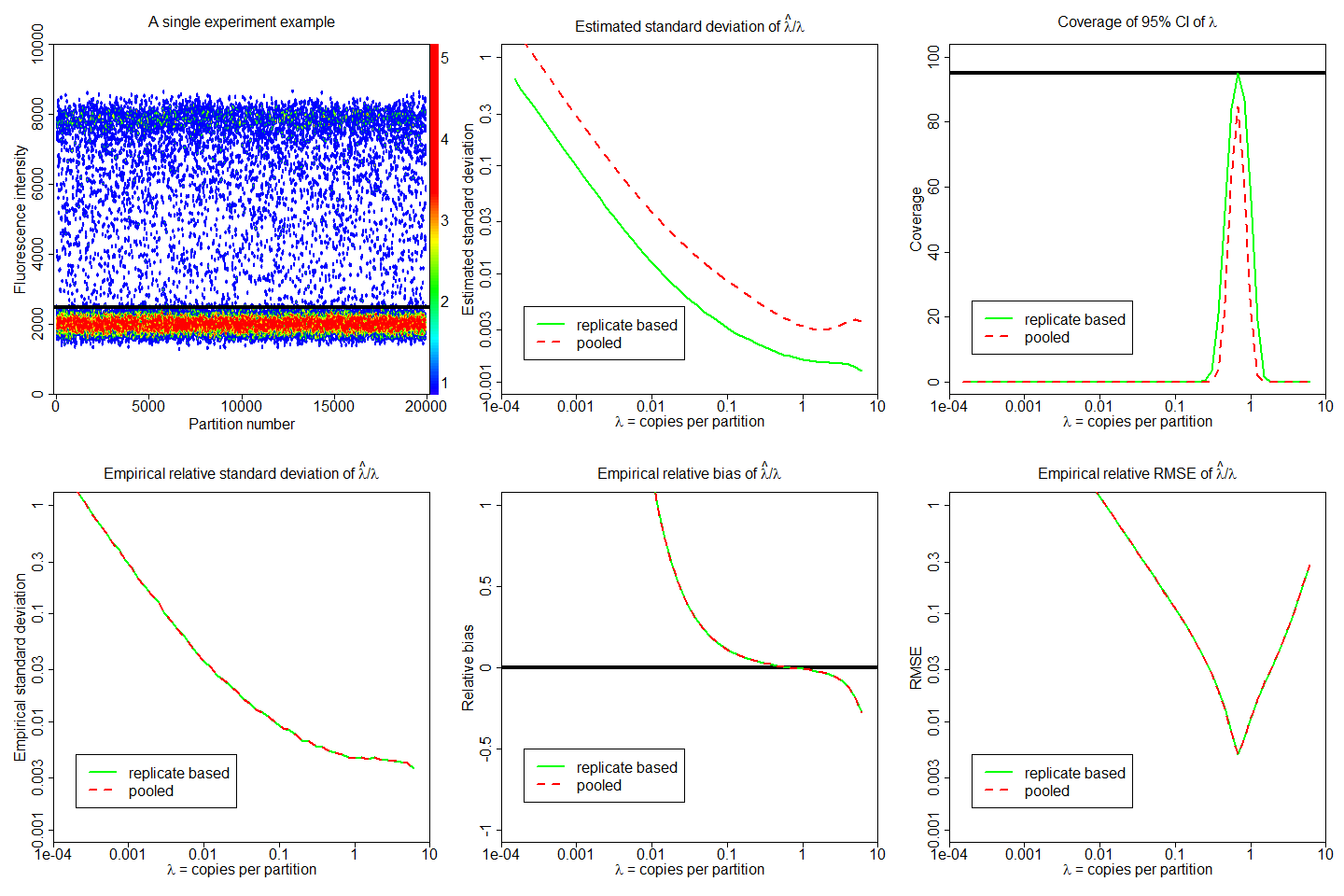

Supplement: Supplementary file 4 — Additional file 4: Interactive tool. In this mini-website, we provide an interactive tool to study the influence of specific sources of variation on the performance of the concentration estimators. This can serve as a guide when designing an experiment. All results are relative to the true concentration and based on 1000 simulations with 8 technical replicates. (ZIP 17 MB) [file 12859_2014_6687_MOESM4_ESM.zip › Additional file 4/RES/RES2273B.png]

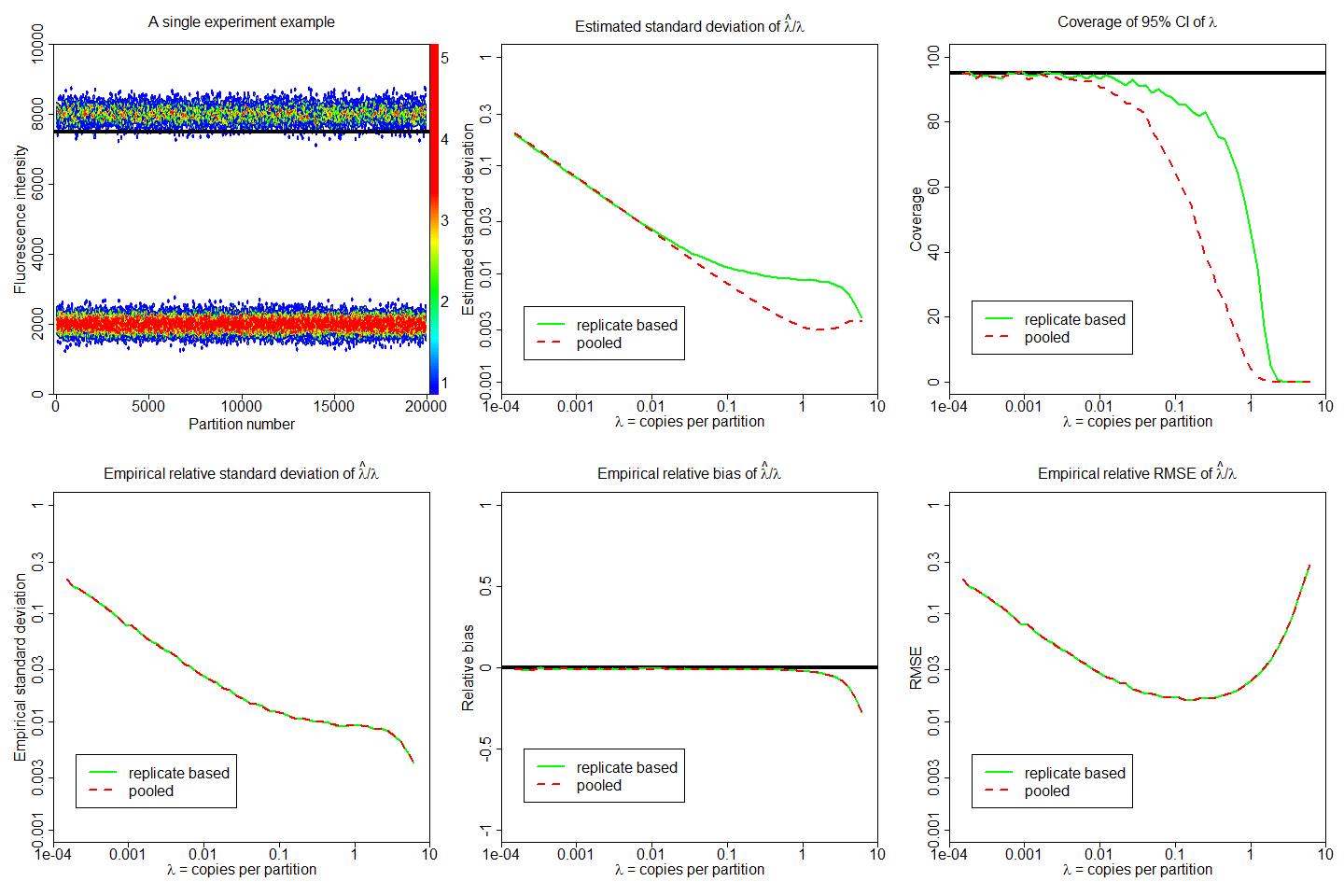

Supplement: Supplementary file 4 — Additional file 4: Interactive tool. In this mini-website, we provide an interactive tool to study the influence of specific sources of variation on the performance of the concentration estimators. This can serve as a guide when designing an experiment. All results are relative to the true concentration and based on 1000 simulations with 8 technical replicates. (ZIP 17 MB) [file 12859_2014_6687_MOESM4_ESM.zip › Additional file 4/RES/RES2311B.png]

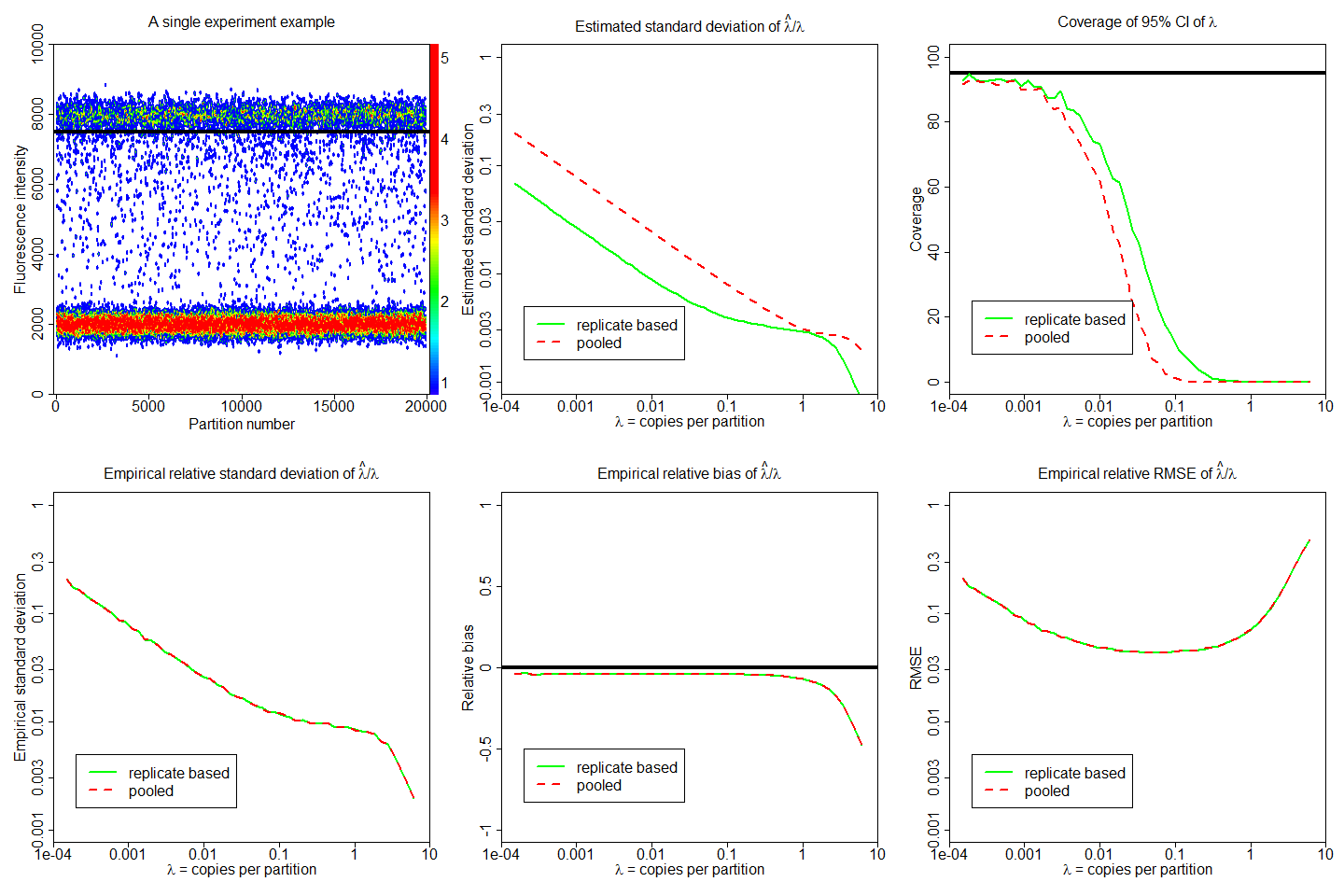

Supplement: Supplementary file 4 — Additional file 4: Interactive tool. In this mini-website, we provide an interactive tool to study the influence of specific sources of variation on the performance of the concentration estimators. This can serve as a guide when designing an experiment. All results are relative to the true concentration and based on 1000 simulations with 8 technical replicates. (ZIP 17 MB) [file 12859_2014_6687_MOESM4_ESM.zip › Additional file 4/RES/RES2312B.png]

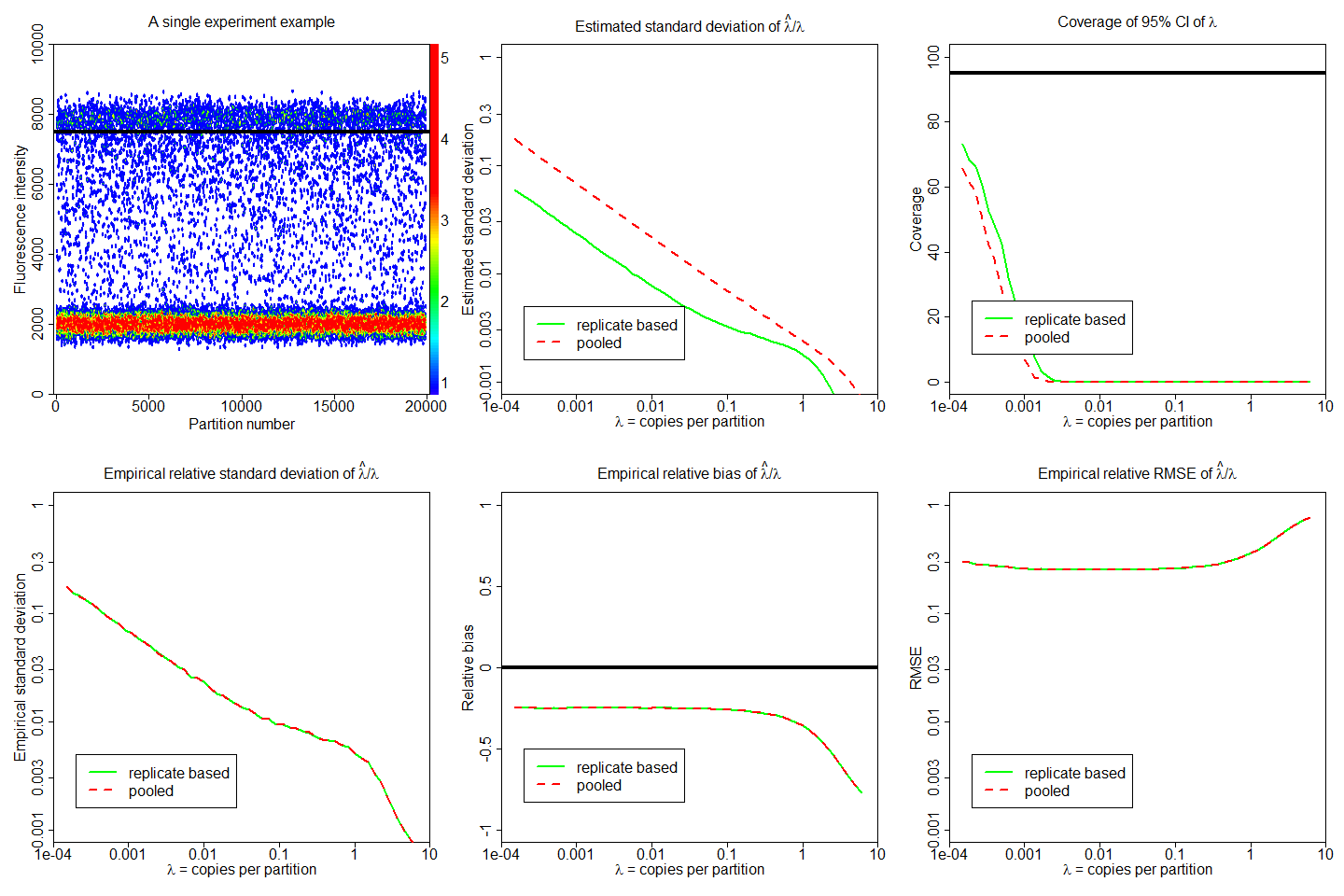

Supplement: Supplementary file 4 — Additional file 4: Interactive tool. In this mini-website, we provide an interactive tool to study the influence of specific sources of variation on the performance of the concentration estimators. This can serve as a guide when designing an experiment. All results are relative to the true concentration and based on 1000 simulations with 8 technical replicates. (ZIP 17 MB) [file 12859_2014_6687_MOESM4_ESM.zip › Additional file 4/RES/RES2313B.png]

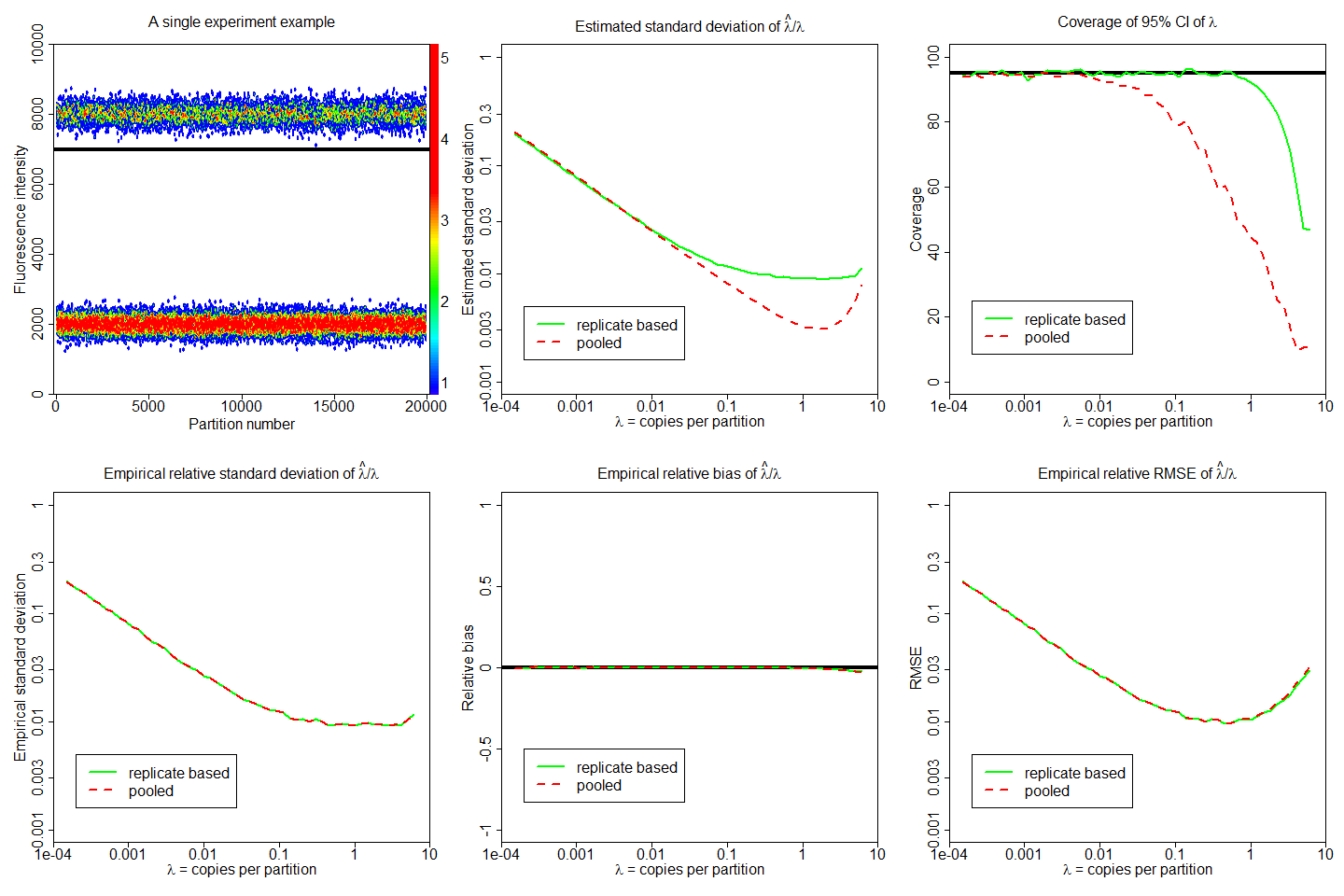

Supplement: Supplementary file 4 — Additional file 4: Interactive tool. In this mini-website, we provide an interactive tool to study the influence of specific sources of variation on the performance of the concentration estimators. This can serve as a guide when designing an experiment. All results are relative to the true concentration and based on 1000 simulations with 8 technical replicates. (ZIP 17 MB) [file 12859_2014_6687_MOESM4_ESM.zip › Additional file 4/RES/RES2321B.png]

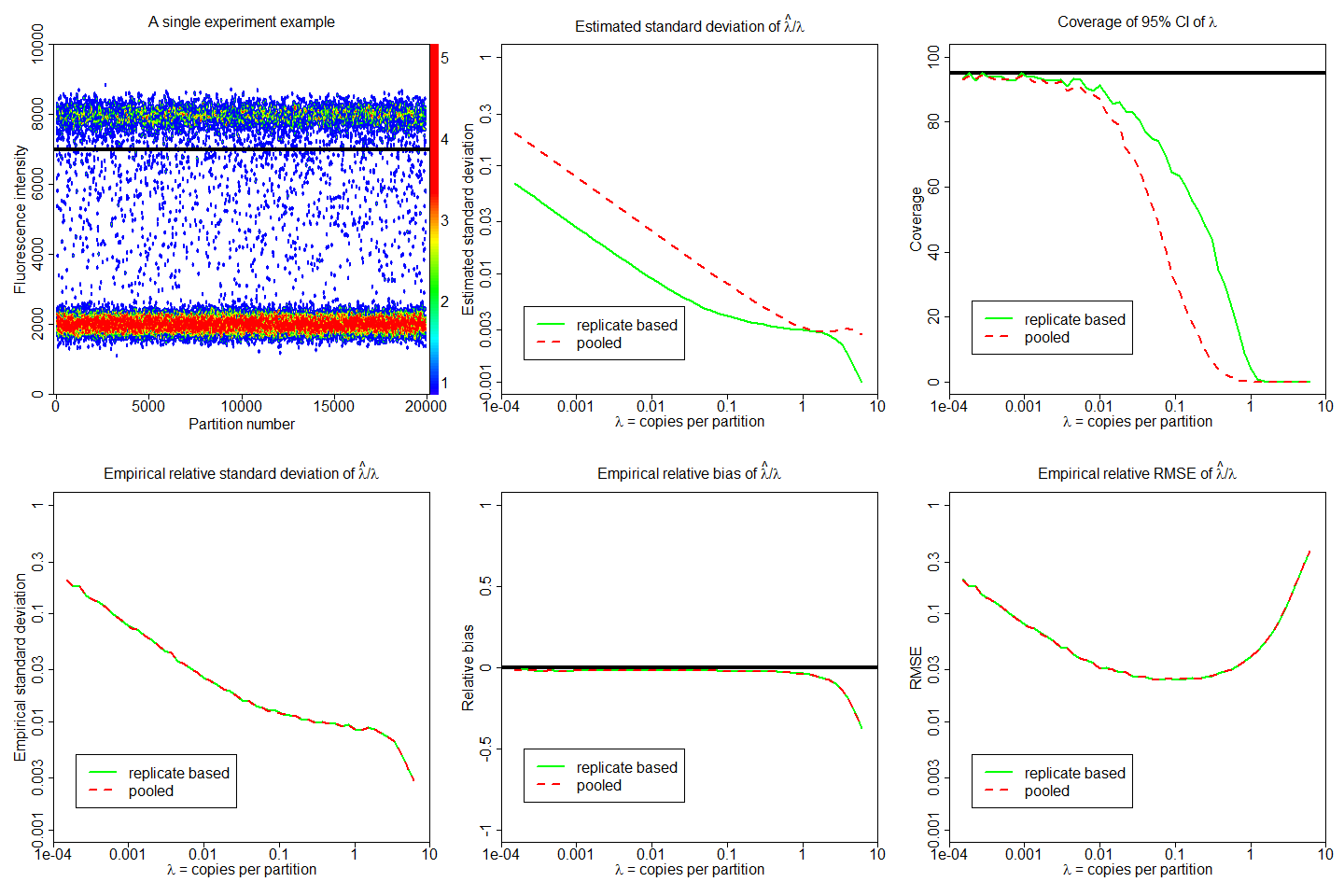

Supplement: Supplementary file 4 — Additional file 4: Interactive tool. In this mini-website, we provide an interactive tool to study the influence of specific sources of variation on the performance of the concentration estimators. This can serve as a guide when designing an experiment. All results are relative to the true concentration and based on 1000 simulations with 8 technical replicates. (ZIP 17 MB) [file 12859_2014_6687_MOESM4_ESM.zip › Additional file 4/RES/RES2322B.png]

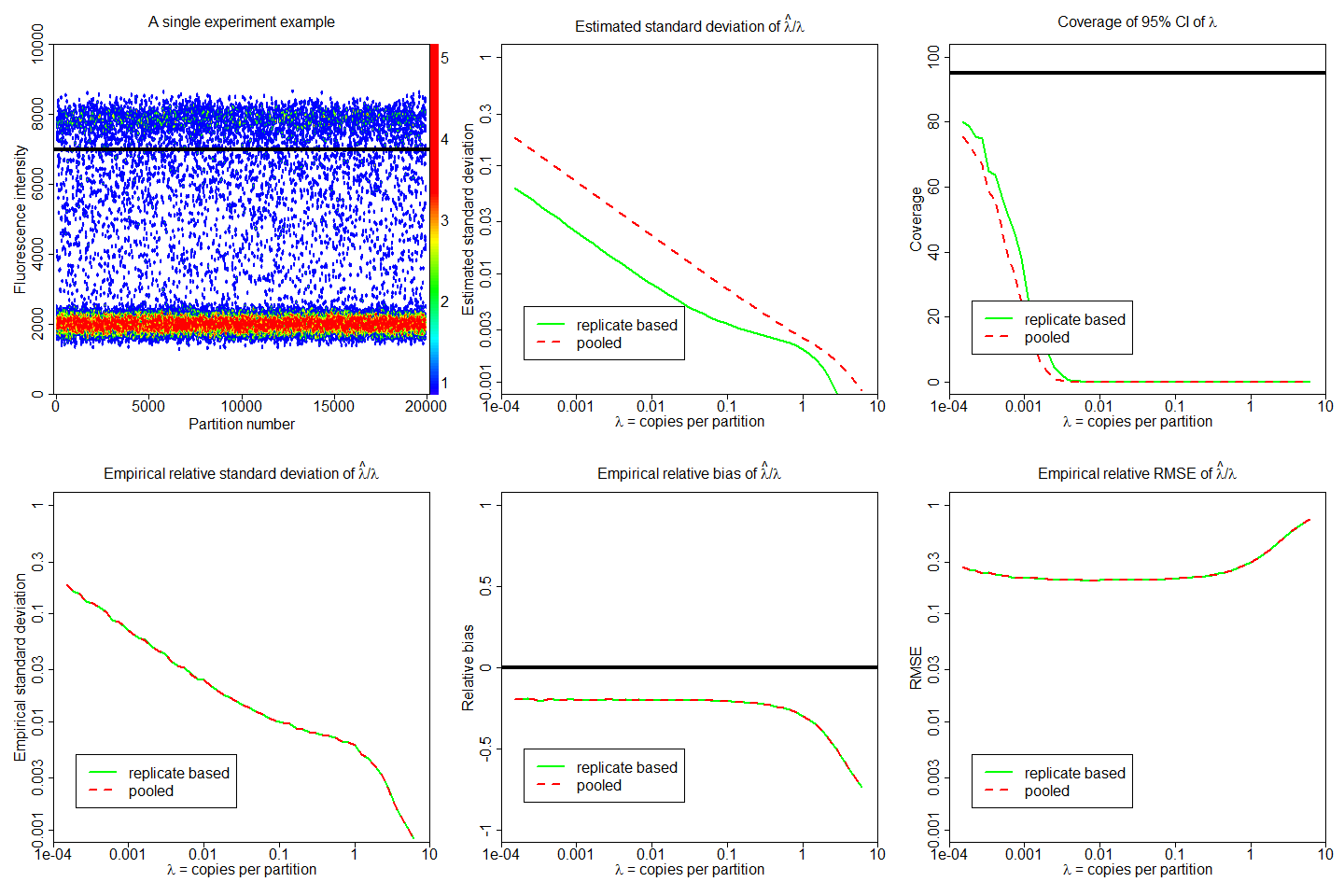

Supplement: Supplementary file 4 — Additional file 4: Interactive tool. In this mini-website, we provide an interactive tool to study the influence of specific sources of variation on the performance of the concentration estimators. This can serve as a guide when designing an experiment. All results are relative to the true concentration and based on 1000 simulations with 8 technical replicates. (ZIP 17 MB) [file 12859_2014_6687_MOESM4_ESM.zip › Additional file 4/RES/RES2323B.png]

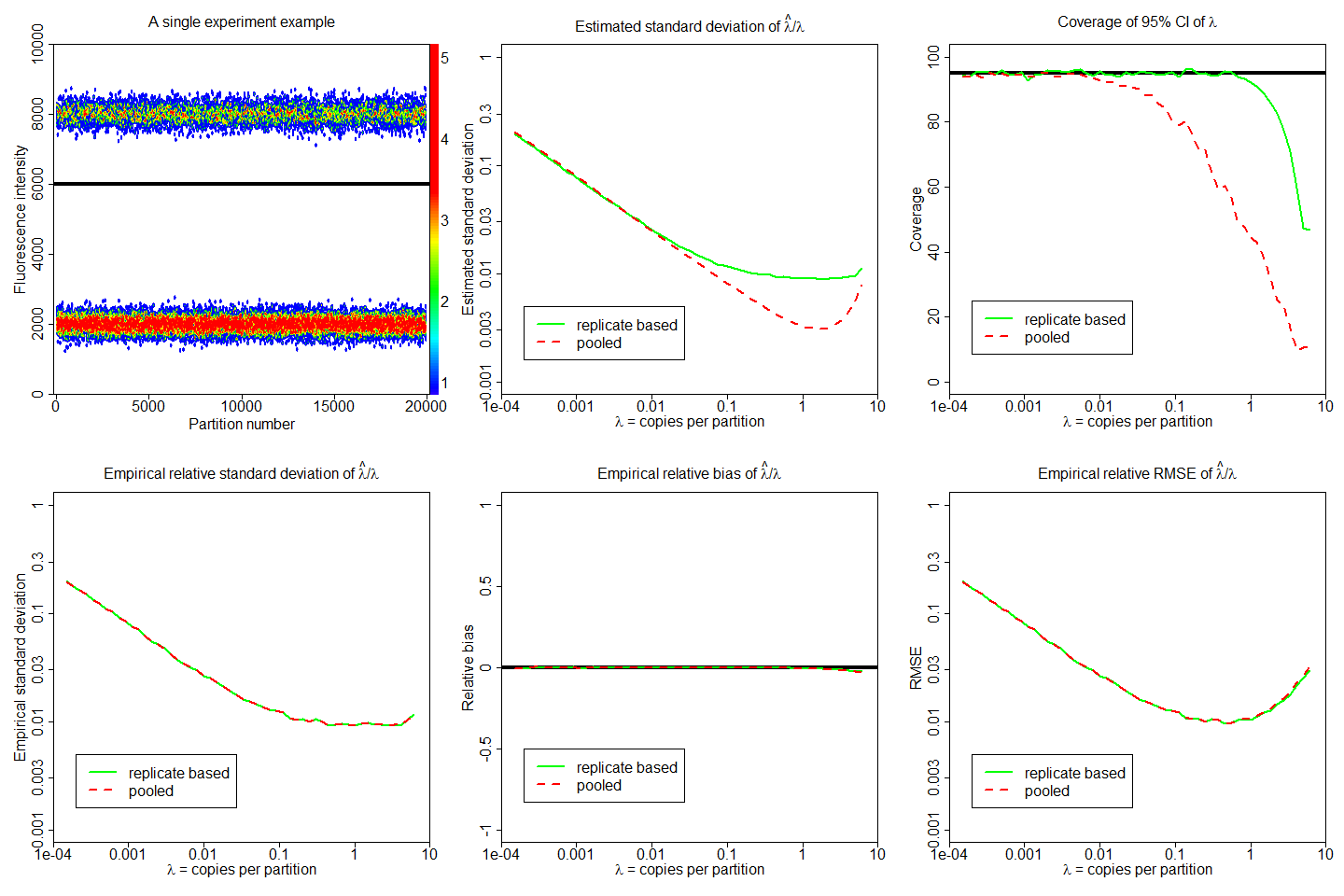

Supplement: Supplementary file 4 — Additional file 4: Interactive tool. In this mini-website, we provide an interactive tool to study the influence of specific sources of variation on the performance of the concentration estimators. This can serve as a guide when designing an experiment. All results are relative to the true concentration and based on 1000 simulations with 8 technical replicates. (ZIP 17 MB) [file 12859_2014_6687_MOESM4_ESM.zip › Additional file 4/RES/RES2331B.png]

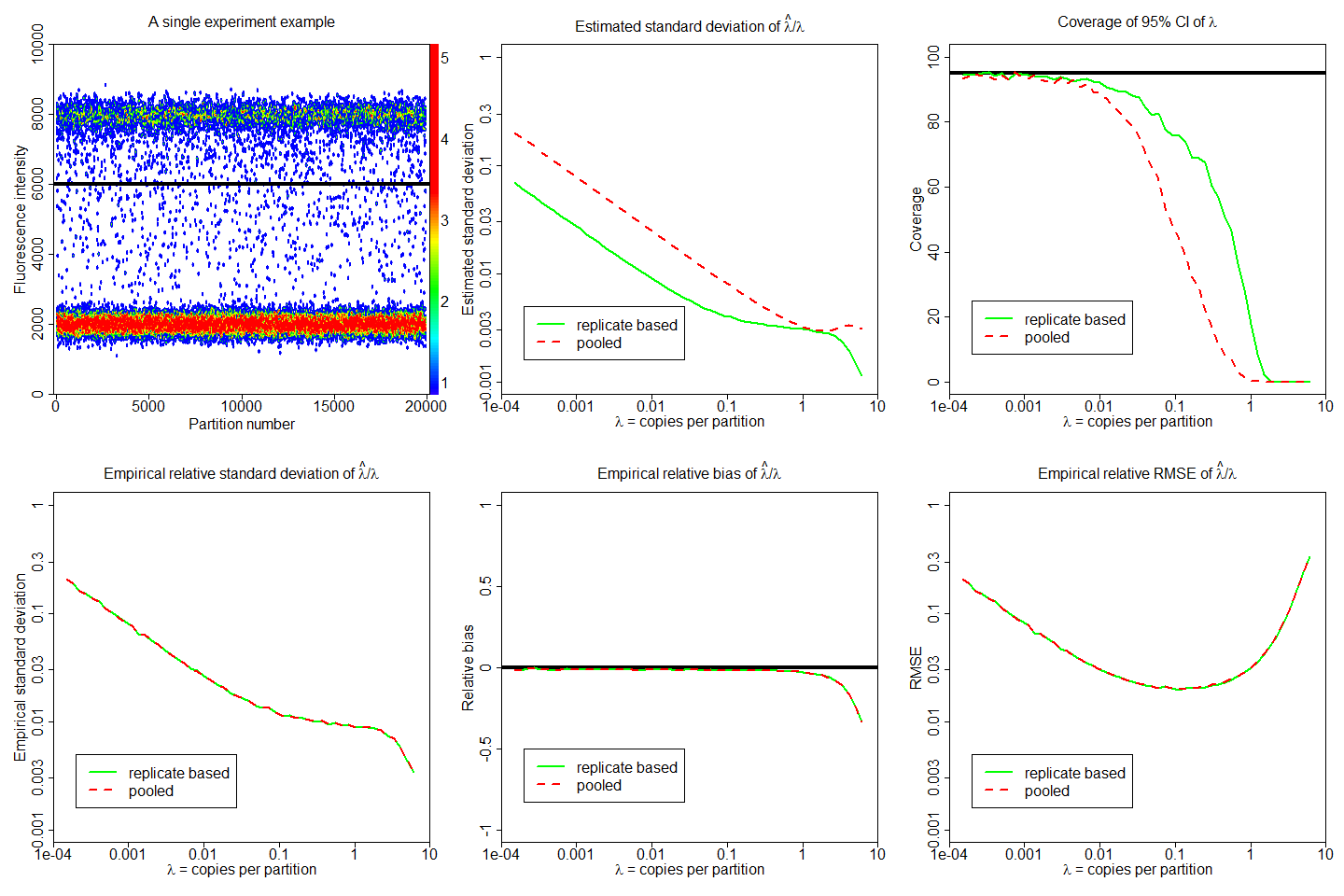

Supplement: Supplementary file 4 — Additional file 4: Interactive tool. In this mini-website, we provide an interactive tool to study the influence of specific sources of variation on the performance of the concentration estimators. This can serve as a guide when designing an experiment. All results are relative to the true concentration and based on 1000 simulations with 8 technical replicates. (ZIP 17 MB) [file 12859_2014_6687_MOESM4_ESM.zip › Additional file 4/RES/RES2332B.png]

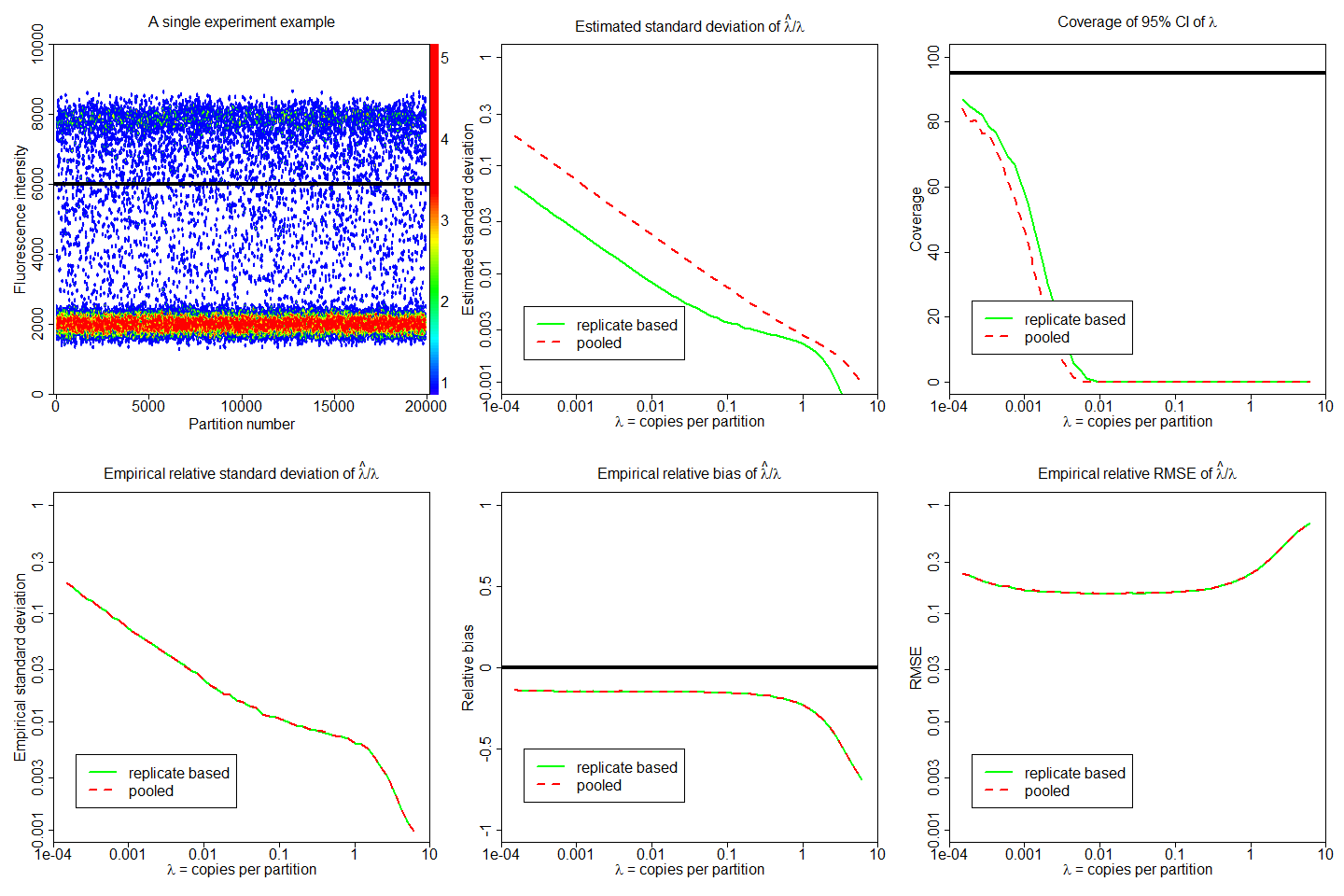

Supplement: Supplementary file 4 — Additional file 4: Interactive tool. In this mini-website, we provide an interactive tool to study the influence of specific sources of variation on the performance of the concentration estimators. This can serve as a guide when designing an experiment. All results are relative to the true concentration and based on 1000 simulations with 8 technical replicates. (ZIP 17 MB) [file 12859_2014_6687_MOESM4_ESM.zip › Additional file 4/RES/RES2333B.png]

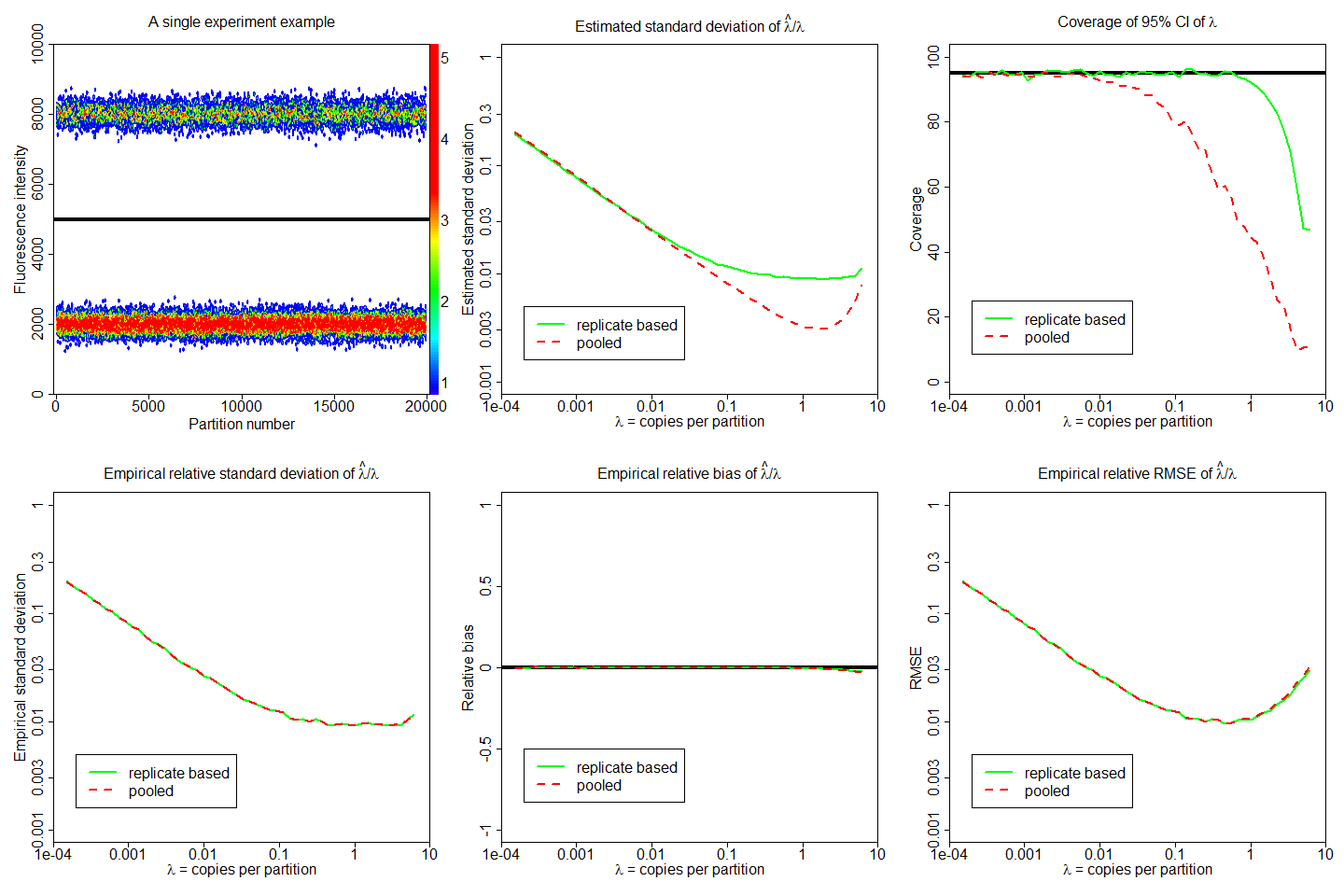

Supplement: Supplementary file 4 — Additional file 4: Interactive tool. In this mini-website, we provide an interactive tool to study the influence of specific sources of variation on the performance of the concentration estimators. This can serve as a guide when designing an experiment. All results are relative to the true concentration and based on 1000 simulations with 8 technical replicates. (ZIP 17 MB) [file 12859_2014_6687_MOESM4_ESM.zip › Additional file 4/RES/RES2341B.png]

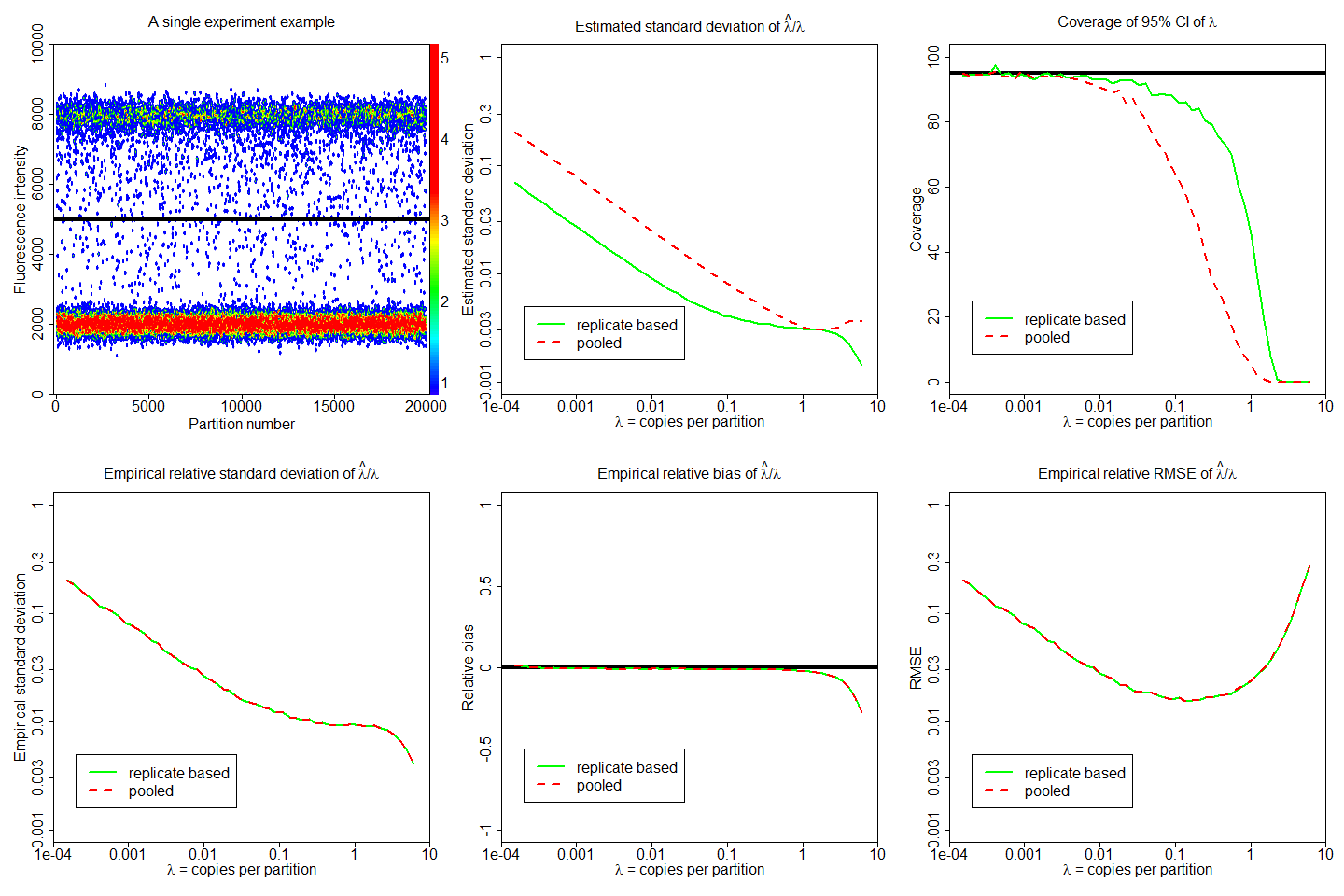

Supplement: Supplementary file 4 — Additional file 4: Interactive tool. In this mini-website, we provide an interactive tool to study the influence of specific sources of variation on the performance of the concentration estimators. This can serve as a guide when designing an experiment. All results are relative to the true concentration and based on 1000 simulations with 8 technical replicates. (ZIP 17 MB) [file 12859_2014_6687_MOESM4_ESM.zip › Additional file 4/RES/RES2342B.png]

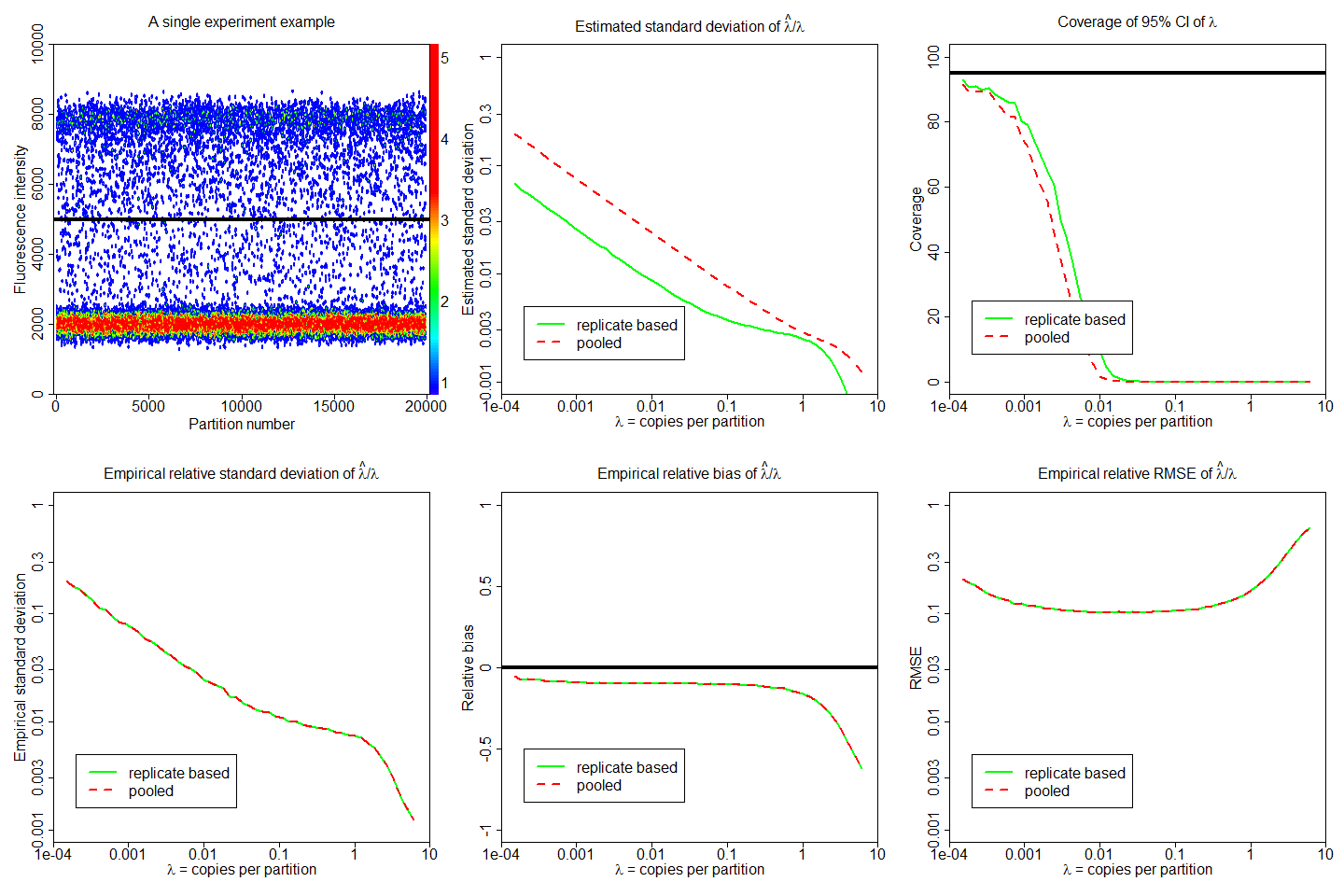

Supplement: Supplementary file 4 — Additional file 4: Interactive tool. In this mini-website, we provide an interactive tool to study the influence of specific sources of variation on the performance of the concentration estimators. This can serve as a guide when designing an experiment. All results are relative to the true concentration and based on 1000 simulations with 8 technical replicates. (ZIP 17 MB) [file 12859_2014_6687_MOESM4_ESM.zip › Additional file 4/RES/RES2343B.png]

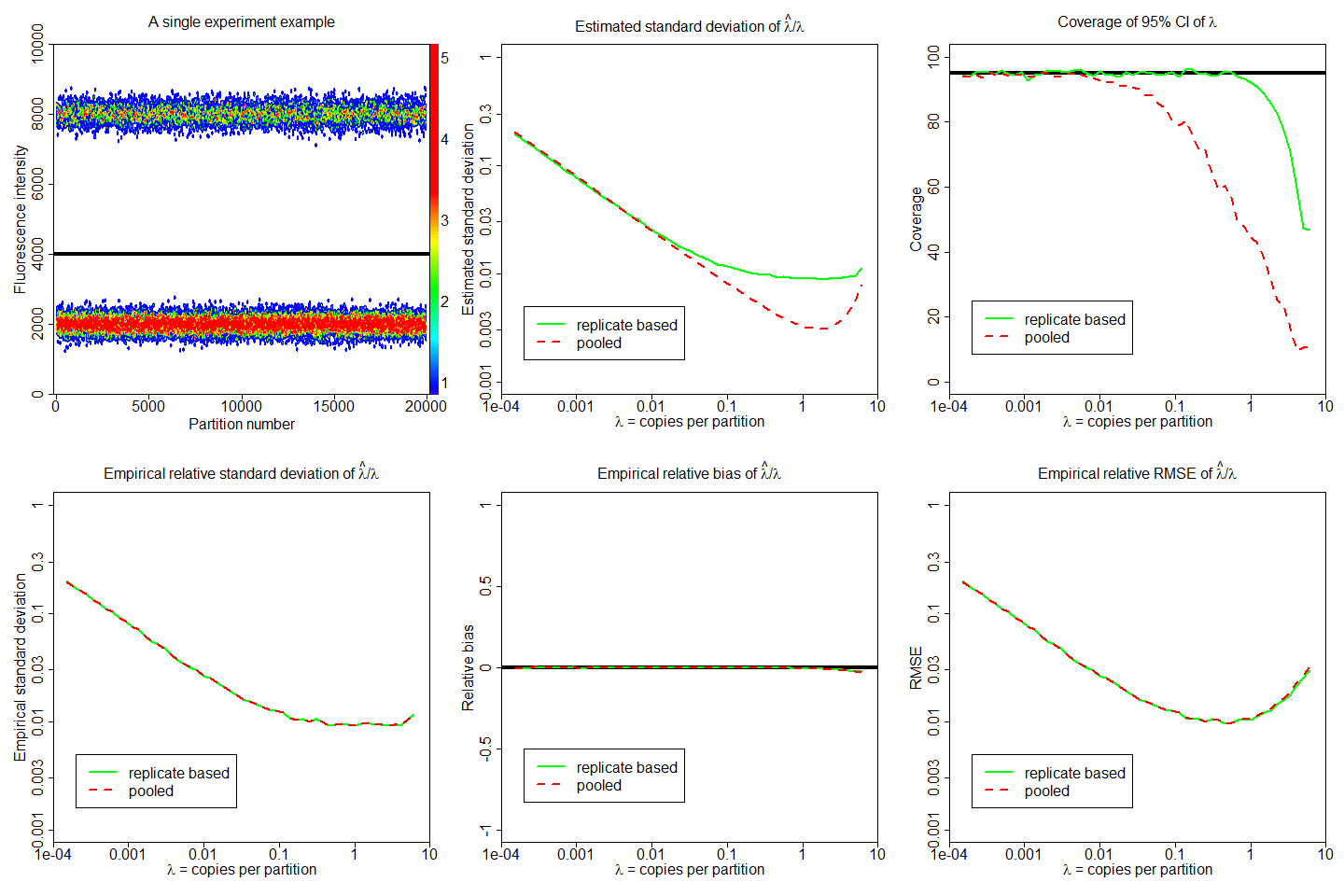

Supplement: Supplementary file 4 — Additional file 4: Interactive tool. In this mini-website, we provide an interactive tool to study the influence of specific sources of variation on the performance of the concentration estimators. This can serve as a guide when designing an experiment. All results are relative to the true concentration and based on 1000 simulations with 8 technical replicates. (ZIP 17 MB) [file 12859_2014_6687_MOESM4_ESM.zip › Additional file 4/RES/RES2351B.png]

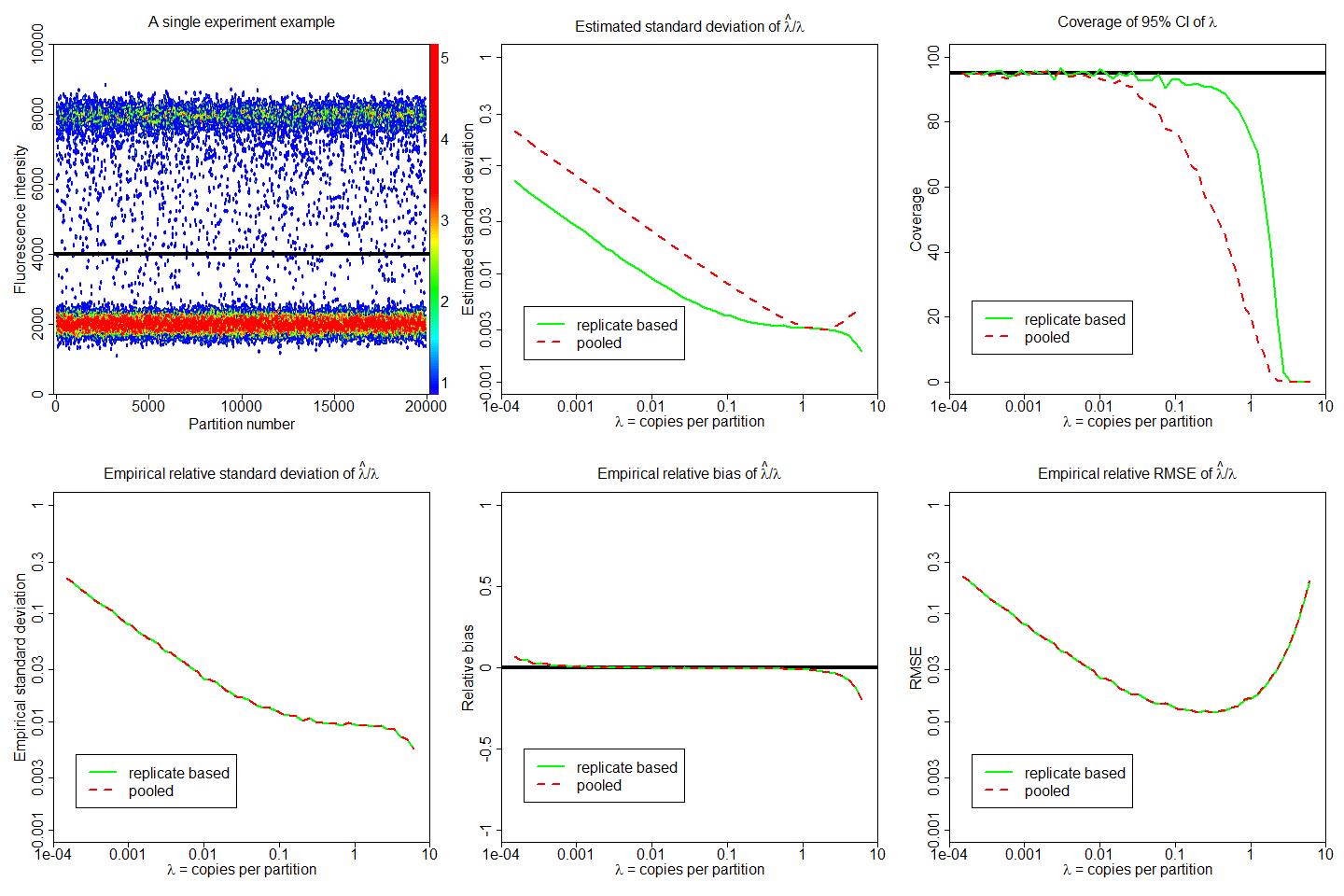

Supplement: Supplementary file 4 — Additional file 4: Interactive tool. In this mini-website, we provide an interactive tool to study the influence of specific sources of variation on the performance of the concentration estimators. This can serve as a guide when designing an experiment. All results are relative to the true concentration and based on 1000 simulations with 8 technical replicates. (ZIP 17 MB) [file 12859_2014_6687_MOESM4_ESM.zip › Additional file 4/RES/RES2352B.png]

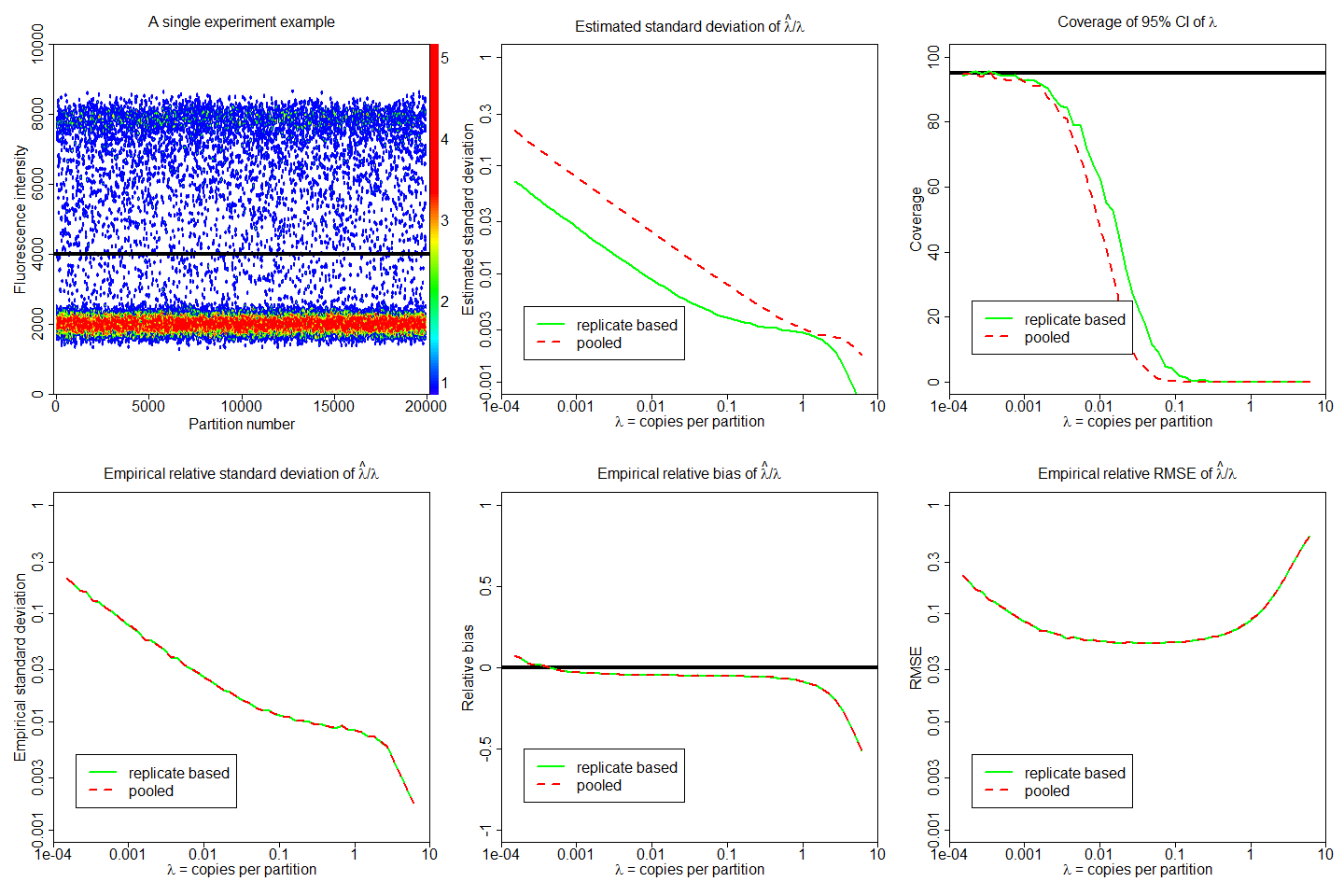

Supplement: Supplementary file 4 — Additional file 4: Interactive tool. In this mini-website, we provide an interactive tool to study the influence of specific sources of variation on the performance of the concentration estimators. This can serve as a guide when designing an experiment. All results are relative to the true concentration and based on 1000 simulations with 8 technical replicates. (ZIP 17 MB) [file 12859_2014_6687_MOESM4_ESM.zip › Additional file 4/RES/RES2353B.png]

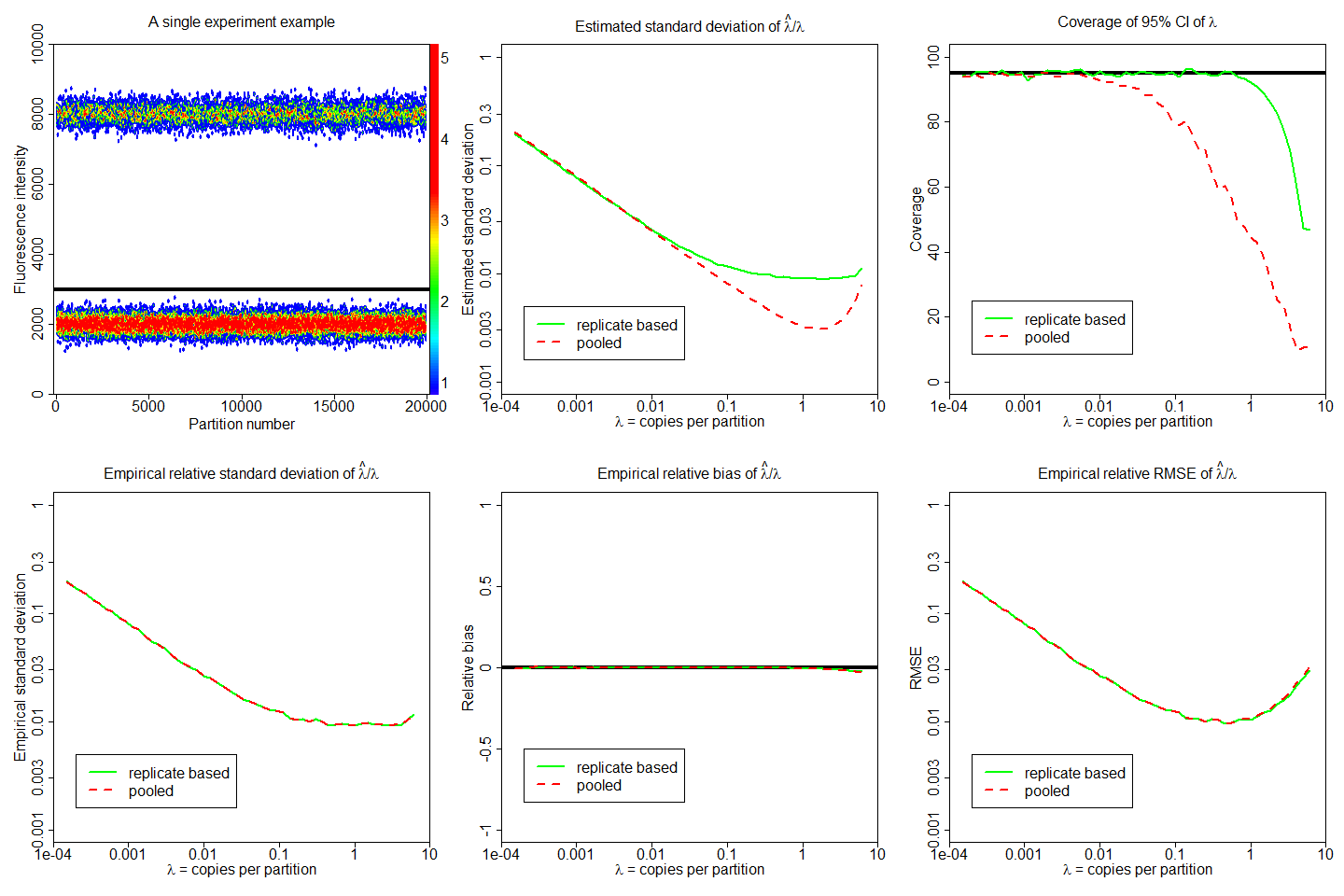

Supplement: Supplementary file 4 — Additional file 4: Interactive tool. In this mini-website, we provide an interactive tool to study the influence of specific sources of variation on the performance of the concentration estimators. This can serve as a guide when designing an experiment. All results are relative to the true concentration and based on 1000 simulations with 8 technical replicates. (ZIP 17 MB) [file 12859_2014_6687_MOESM4_ESM.zip › Additional file 4/RES/RES2361B.png]

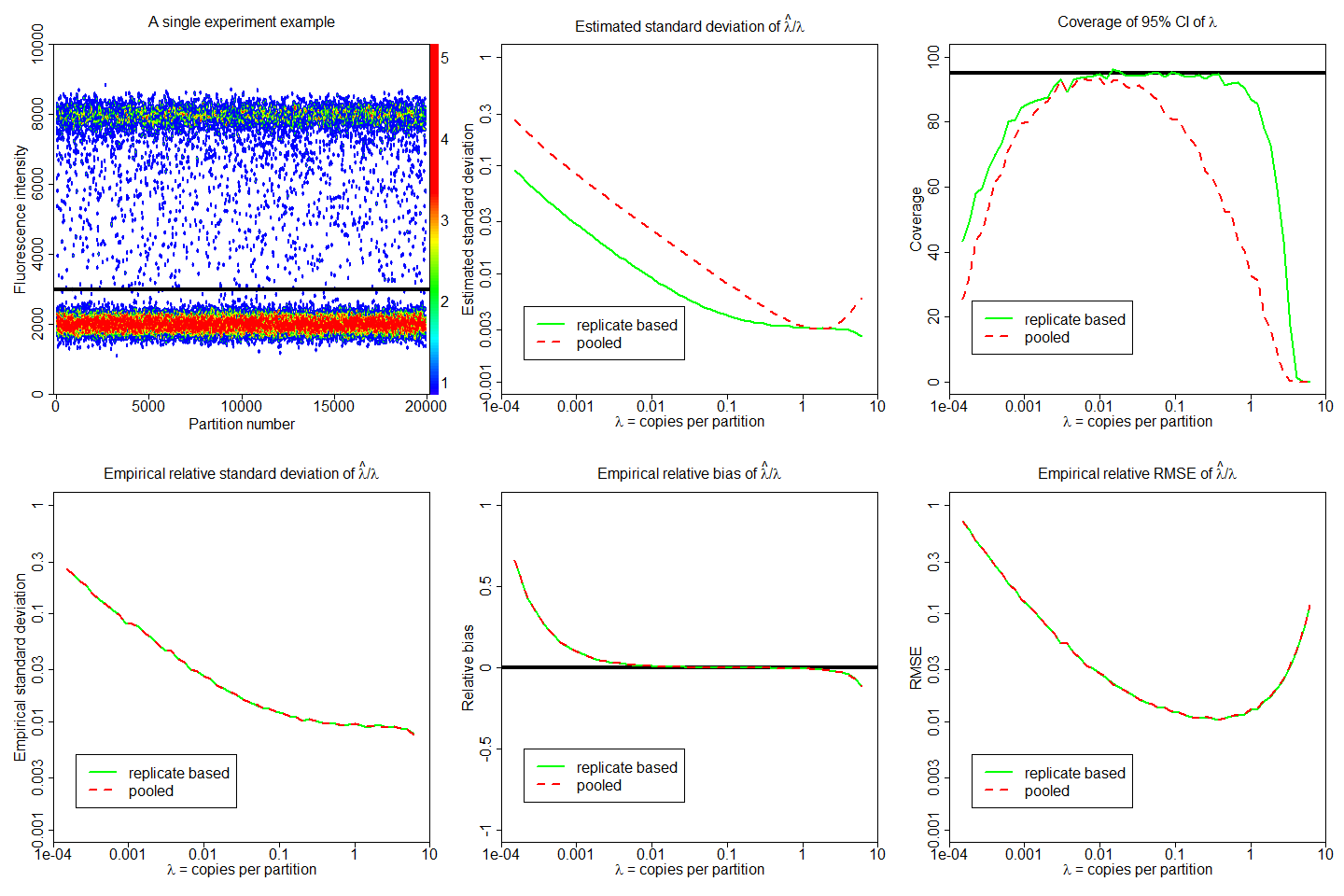

Supplement: Supplementary file 4 — Additional file 4: Interactive tool. In this mini-website, we provide an interactive tool to study the influence of specific sources of variation on the performance of the concentration estimators. This can serve as a guide when designing an experiment. All results are relative to the true concentration and based on 1000 simulations with 8 technical replicates. (ZIP 17 MB) [file 12859_2014_6687_MOESM4_ESM.zip › Additional file 4/RES/RES2362B.png]

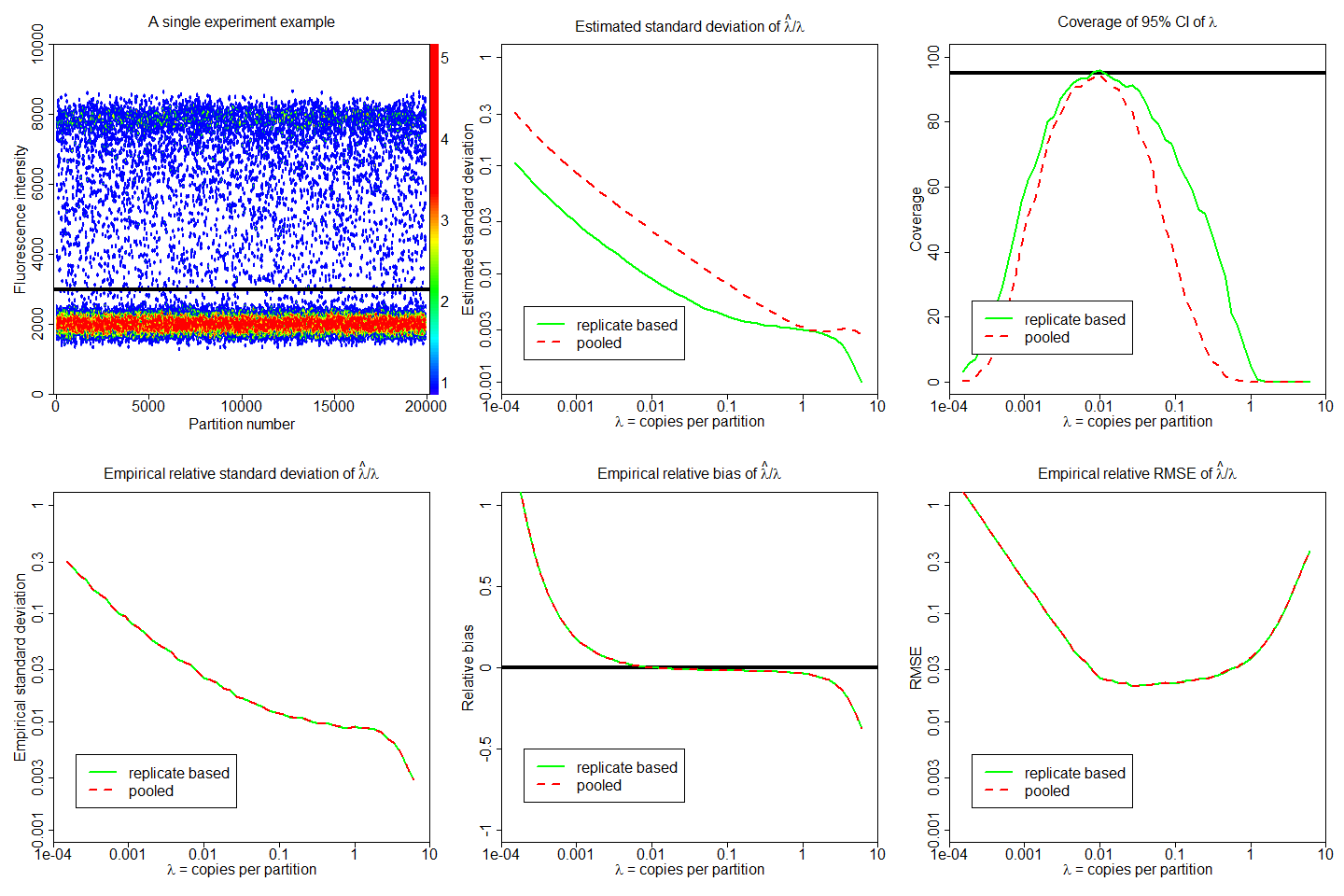

Supplement: Supplementary file 4 — Additional file 4: Interactive tool. In this mini-website, we provide an interactive tool to study the influence of specific sources of variation on the performance of the concentration estimators. This can serve as a guide when designing an experiment. All results are relative to the true concentration and based on 1000 simulations with 8 technical replicates. (ZIP 17 MB) [file 12859_2014_6687_MOESM4_ESM.zip › Additional file 4/RES/RES2363B.png]

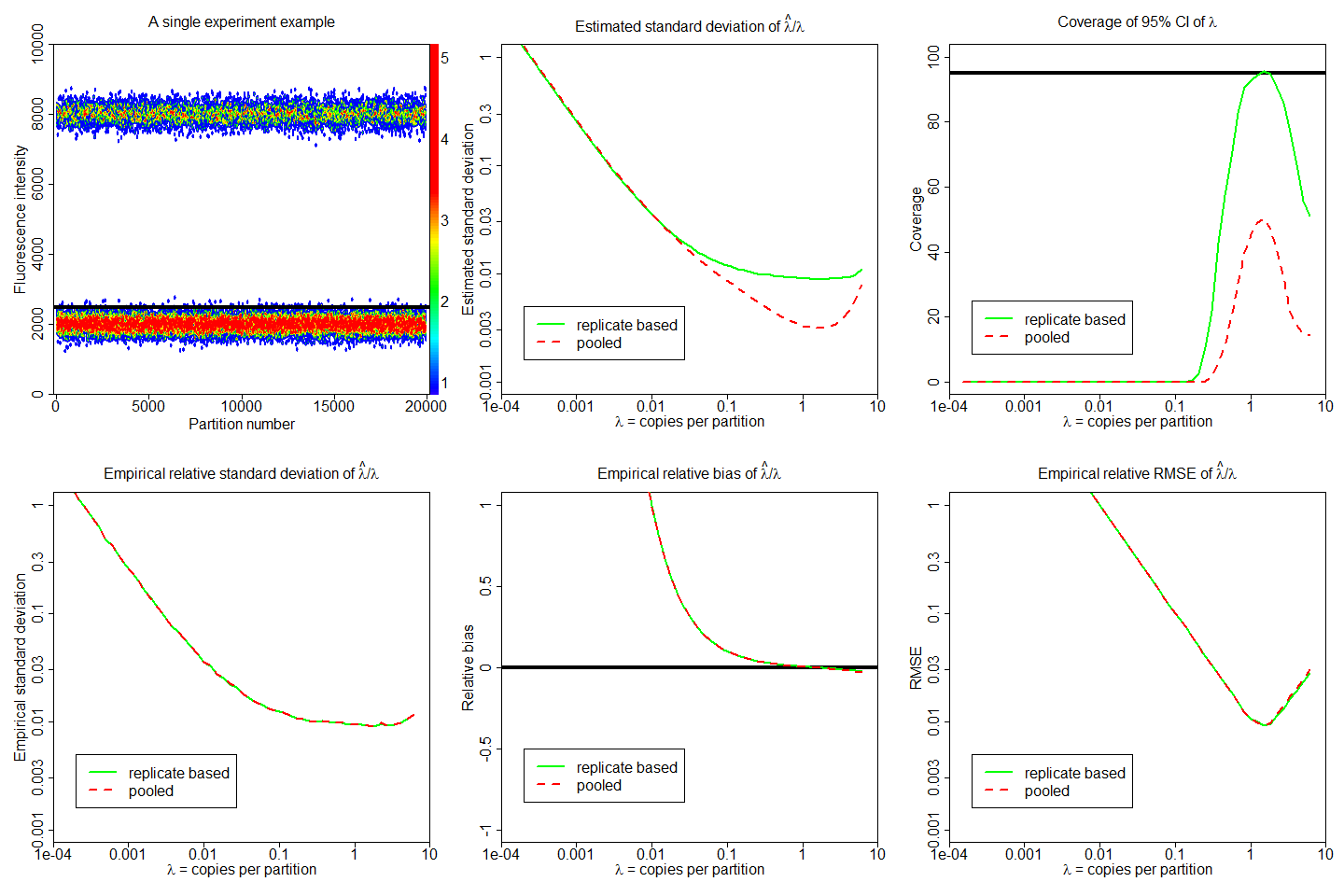

Supplement: Supplementary file 4 — Additional file 4: Interactive tool. In this mini-website, we provide an interactive tool to study the influence of specific sources of variation on the performance of the concentration estimators. This can serve as a guide when designing an experiment. All results are relative to the true concentration and based on 1000 simulations with 8 technical replicates. (ZIP 17 MB) [file 12859_2014_6687_MOESM4_ESM.zip › Additional file 4/RES/RES2371B.png]

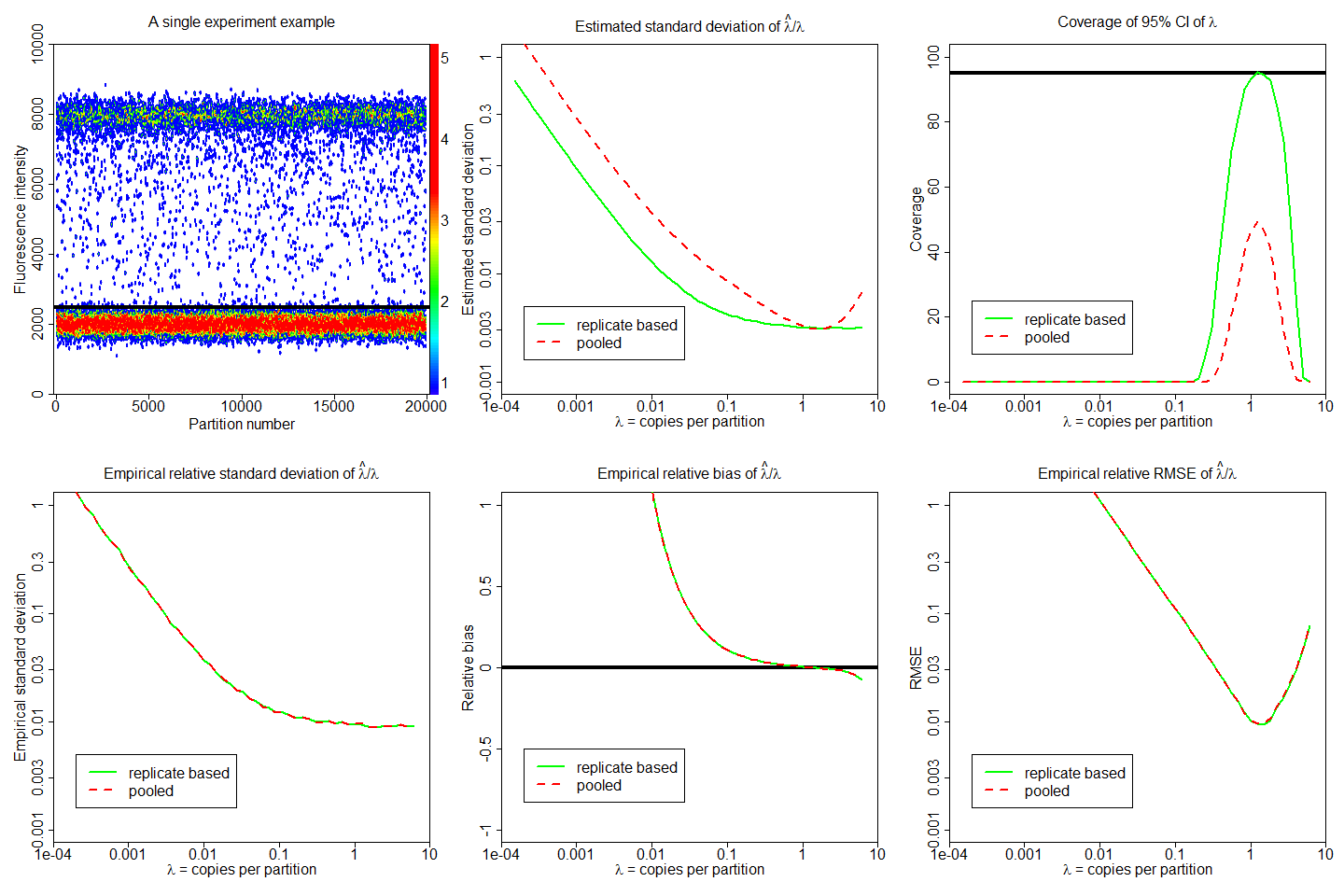

Supplement: Supplementary file 4 — Additional file 4: Interactive tool. In this mini-website, we provide an interactive tool to study the influence of specific sources of variation on the performance of the concentration estimators. This can serve as a guide when designing an experiment. All results are relative to the true concentration and based on 1000 simulations with 8 technical replicates. (ZIP 17 MB) [file 12859_2014_6687_MOESM4_ESM.zip › Additional file 4/RES/RES2372B.png]

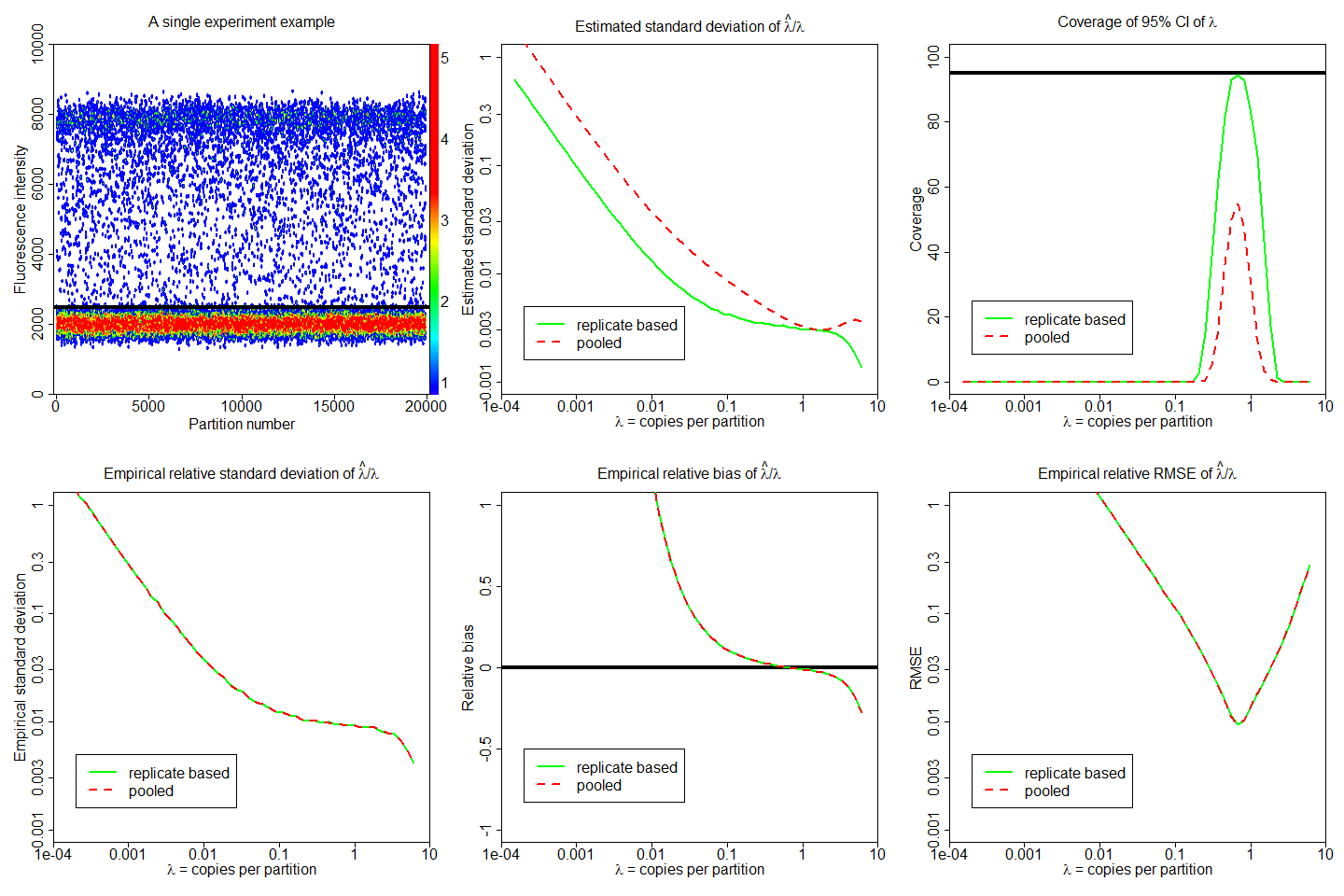

Supplement: Supplementary file 4 — Additional file 4: Interactive tool. In this mini-website, we provide an interactive tool to study the influence of specific sources of variation on the performance of the concentration estimators. This can serve as a guide when designing an experiment. All results are relative to the true concentration and based on 1000 simulations with 8 technical replicates. (ZIP 17 MB) [file 12859_2014_6687_MOESM4_ESM.zip › Additional file 4/RES/RES2373B.png]

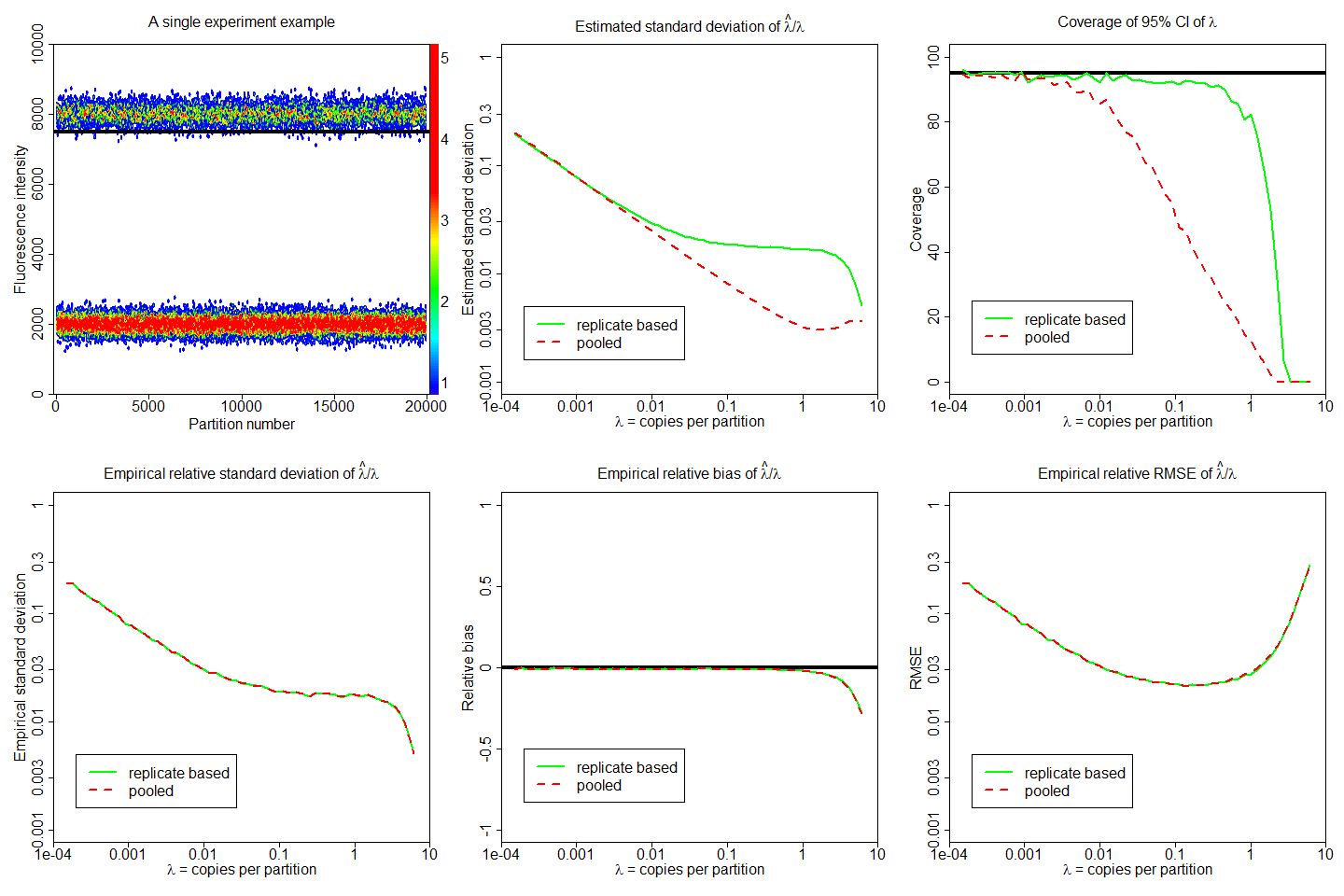

Supplement: Supplementary file 4 — Additional file 4: Interactive tool. In this mini-website, we provide an interactive tool to study the influence of specific sources of variation on the performance of the concentration estimators. This can serve as a guide when designing an experiment. All results are relative to the true concentration and based on 1000 simulations with 8 technical replicates. (ZIP 17 MB) [file 12859_2014_6687_MOESM4_ESM.zip › Additional file 4/RES/RES2411B.png]

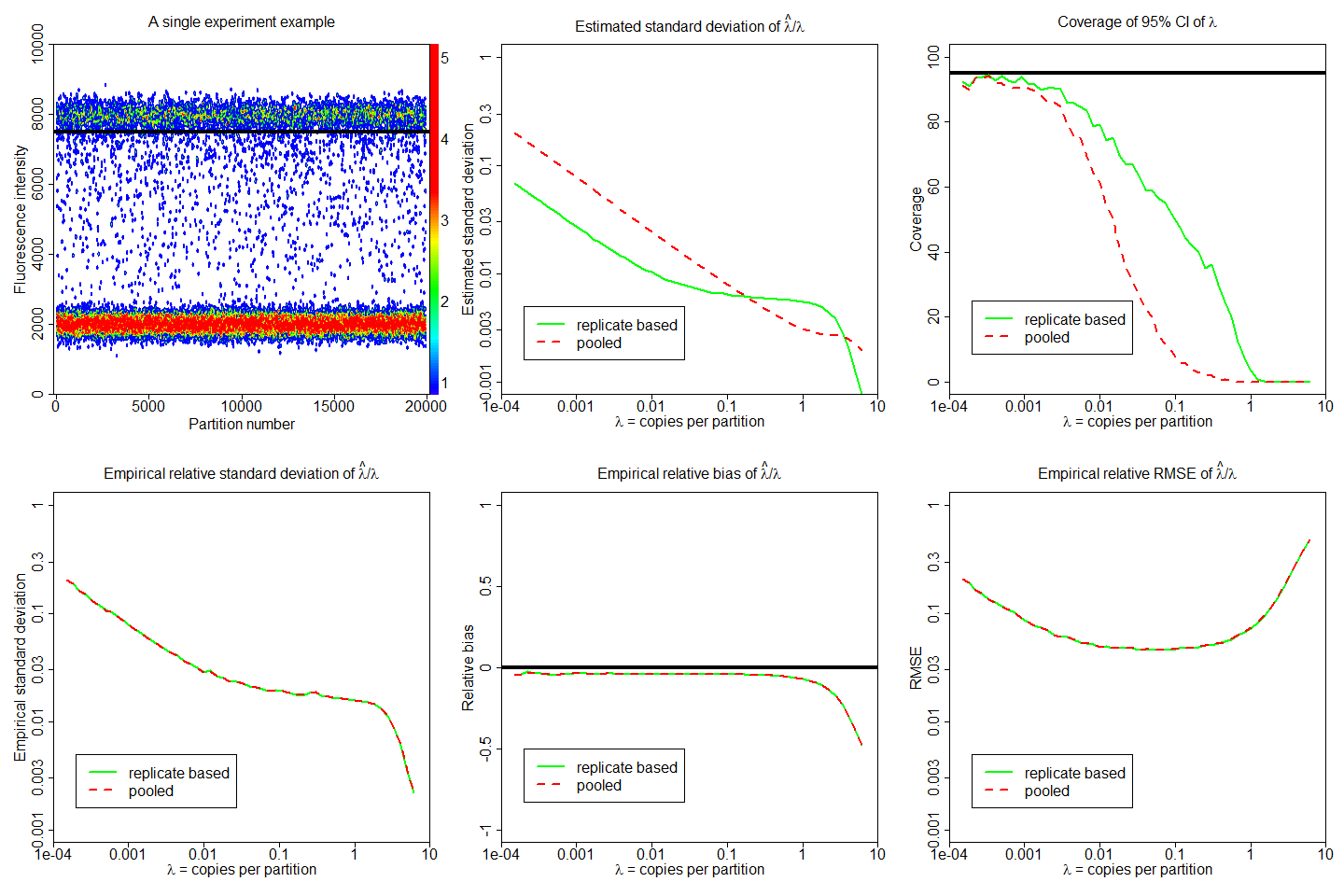

Supplement: Supplementary file 4 — Additional file 4: Interactive tool. In this mini-website, we provide an interactive tool to study the influence of specific sources of variation on the performance of the concentration estimators. This can serve as a guide when designing an experiment. All results are relative to the true concentration and based on 1000 simulations with 8 technical replicates. (ZIP 17 MB) [file 12859_2014_6687_MOESM4_ESM.zip › Additional file 4/RES/RES2412B.png]

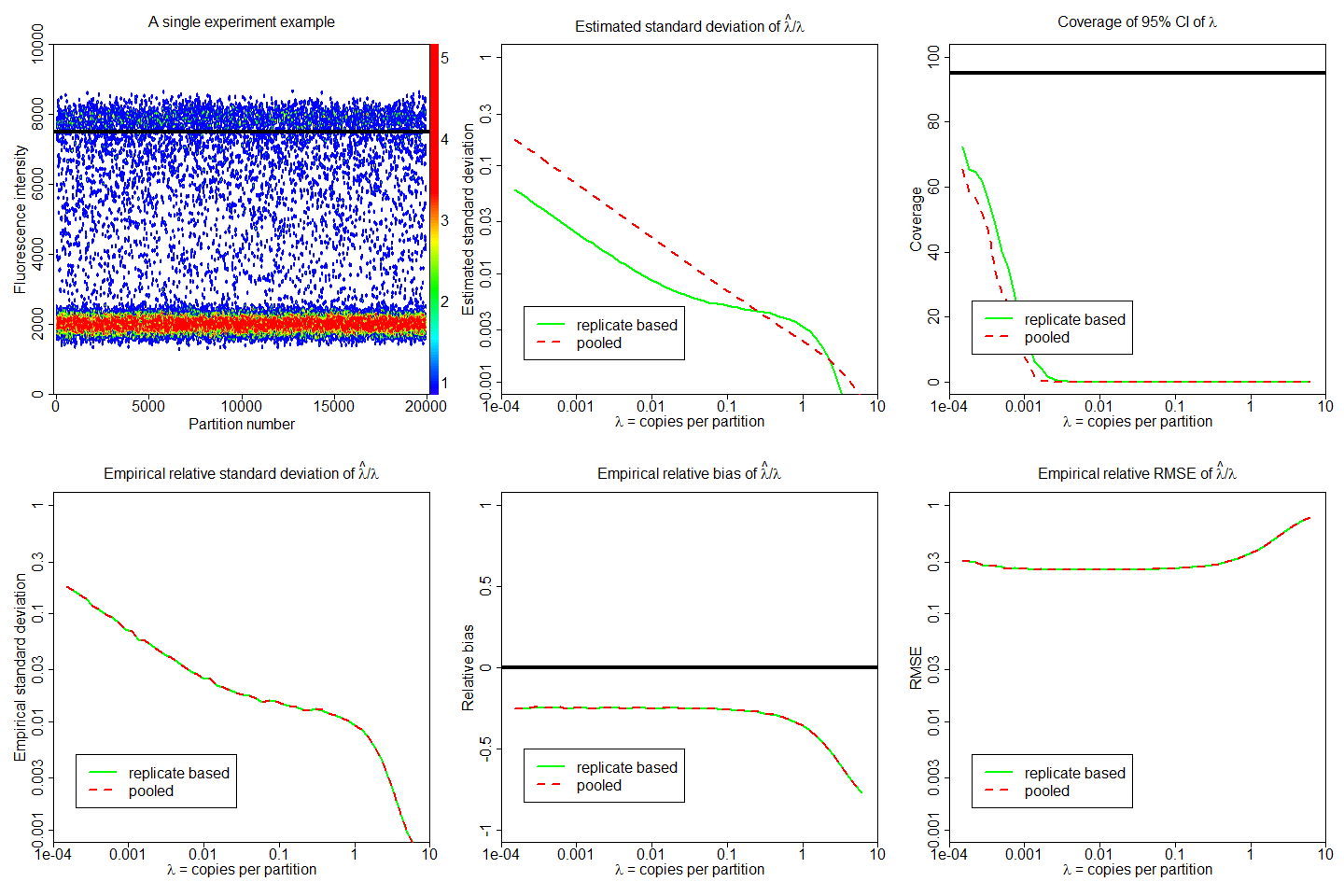

Supplement: Supplementary file 4 — Additional file 4: Interactive tool. In this mini-website, we provide an interactive tool to study the influence of specific sources of variation on the performance of the concentration estimators. This can serve as a guide when designing an experiment. All results are relative to the true concentration and based on 1000 simulations with 8 technical replicates. (ZIP 17 MB) [file 12859_2014_6687_MOESM4_ESM.zip › Additional file 4/RES/RES2413B.png]

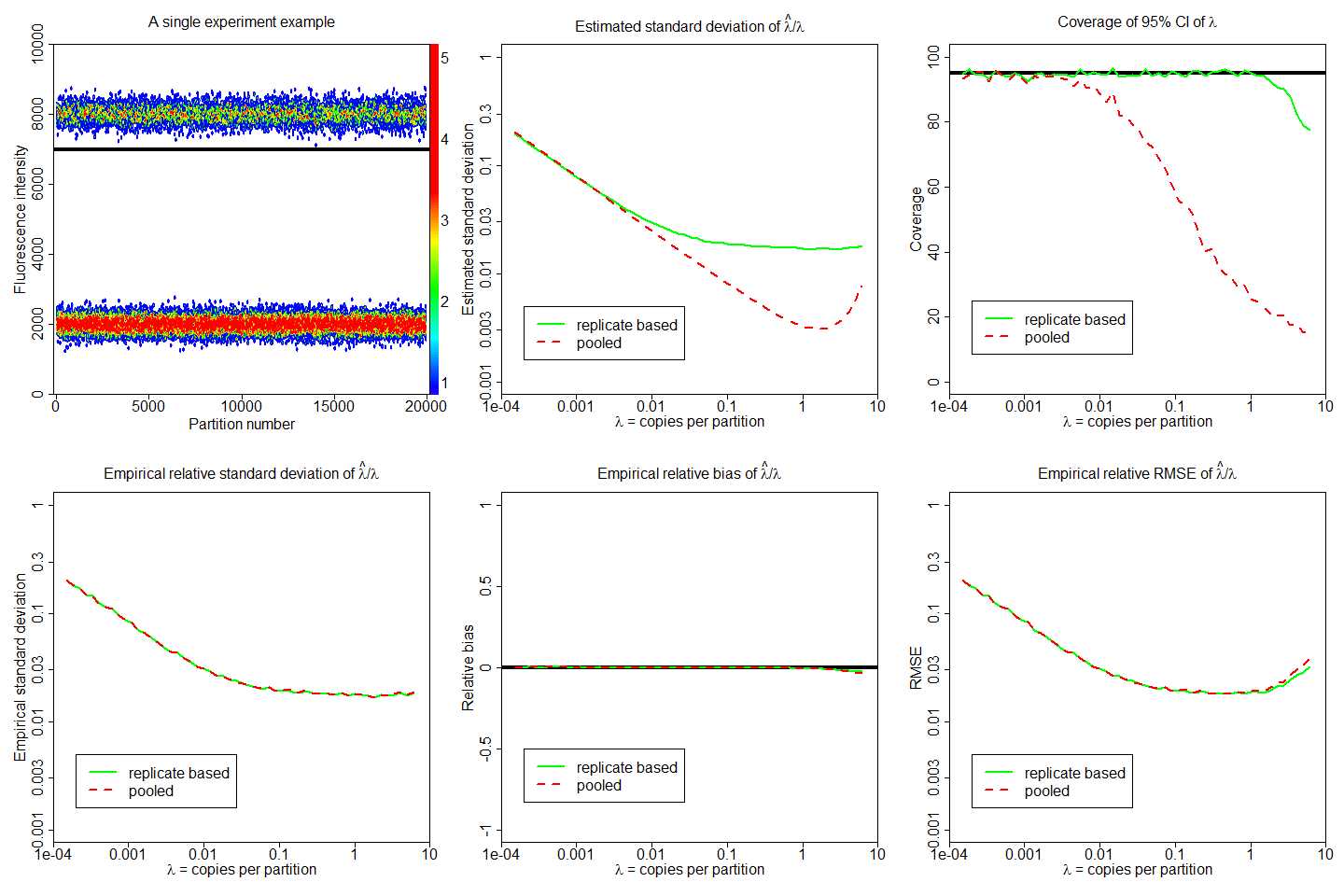

Supplement: Supplementary file 4 — Additional file 4: Interactive tool. In this mini-website, we provide an interactive tool to study the influence of specific sources of variation on the performance of the concentration estimators. This can serve as a guide when designing an experiment. All results are relative to the true concentration and based on 1000 simulations with 8 technical replicates. (ZIP 17 MB) [file 12859_2014_6687_MOESM4_ESM.zip › Additional file 4/RES/RES2421B.png]

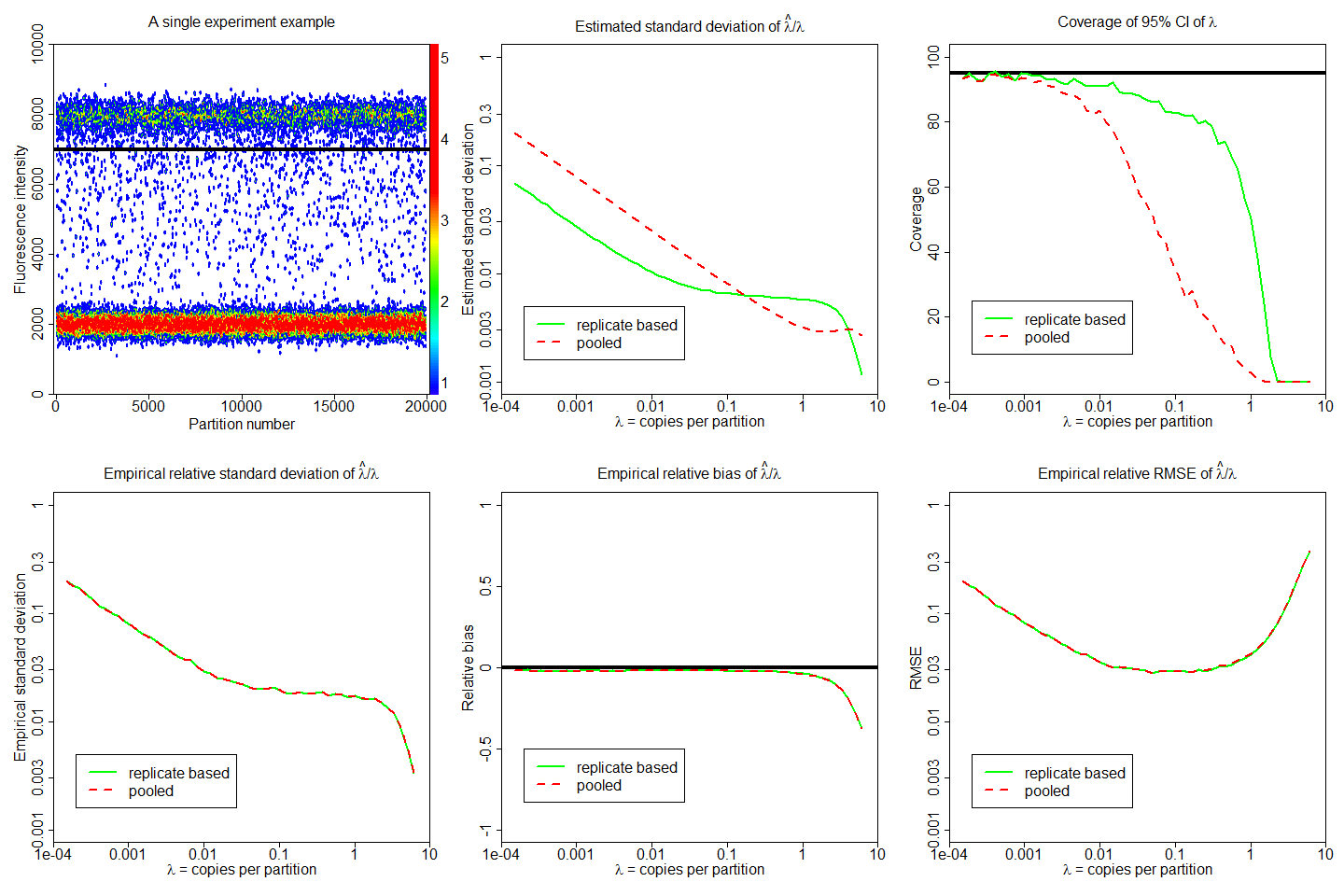

Supplement: Supplementary file 4 — Additional file 4: Interactive tool. In this mini-website, we provide an interactive tool to study the influence of specific sources of variation on the performance of the concentration estimators. This can serve as a guide when designing an experiment. All results are relative to the true concentration and based on 1000 simulations with 8 technical replicates. (ZIP 17 MB) [file 12859_2014_6687_MOESM4_ESM.zip › Additional file 4/RES/RES2422B.png]

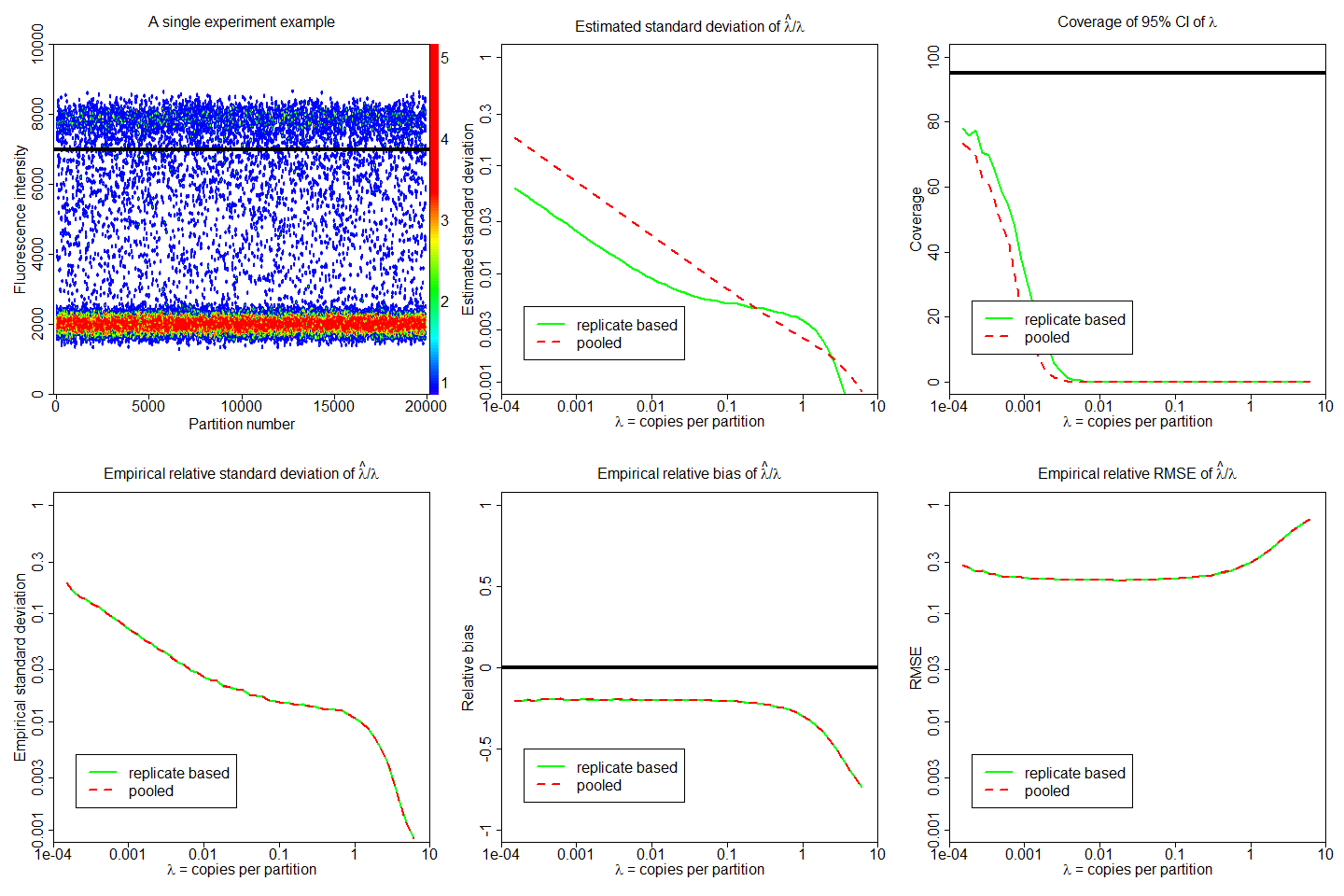

Supplement: Supplementary file 4 — Additional file 4: Interactive tool. In this mini-website, we provide an interactive tool to study the influence of specific sources of variation on the performance of the concentration estimators. This can serve as a guide when designing an experiment. All results are relative to the true concentration and based on 1000 simulations with 8 technical replicates. (ZIP 17 MB) [file 12859_2014_6687_MOESM4_ESM.zip › Additional file 4/RES/RES2423B.png]

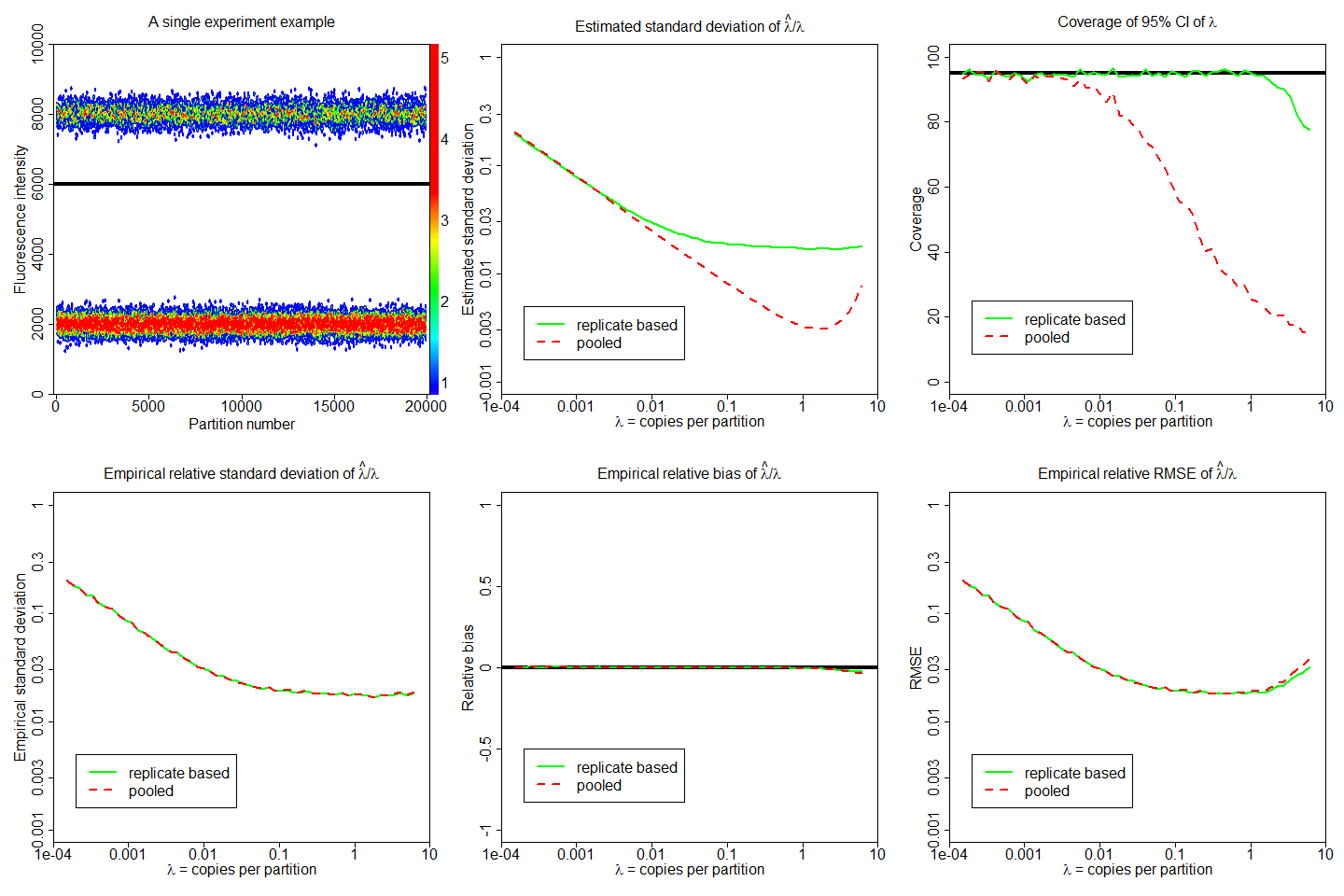

Supplement: Supplementary file 4 — Additional file 4: Interactive tool. In this mini-website, we provide an interactive tool to study the influence of specific sources of variation on the performance of the concentration estimators. This can serve as a guide when designing an experiment. All results are relative to the true concentration and based on 1000 simulations with 8 technical replicates. (ZIP 17 MB) [file 12859_2014_6687_MOESM4_ESM.zip › Additional file 4/RES/RES2431B.png]

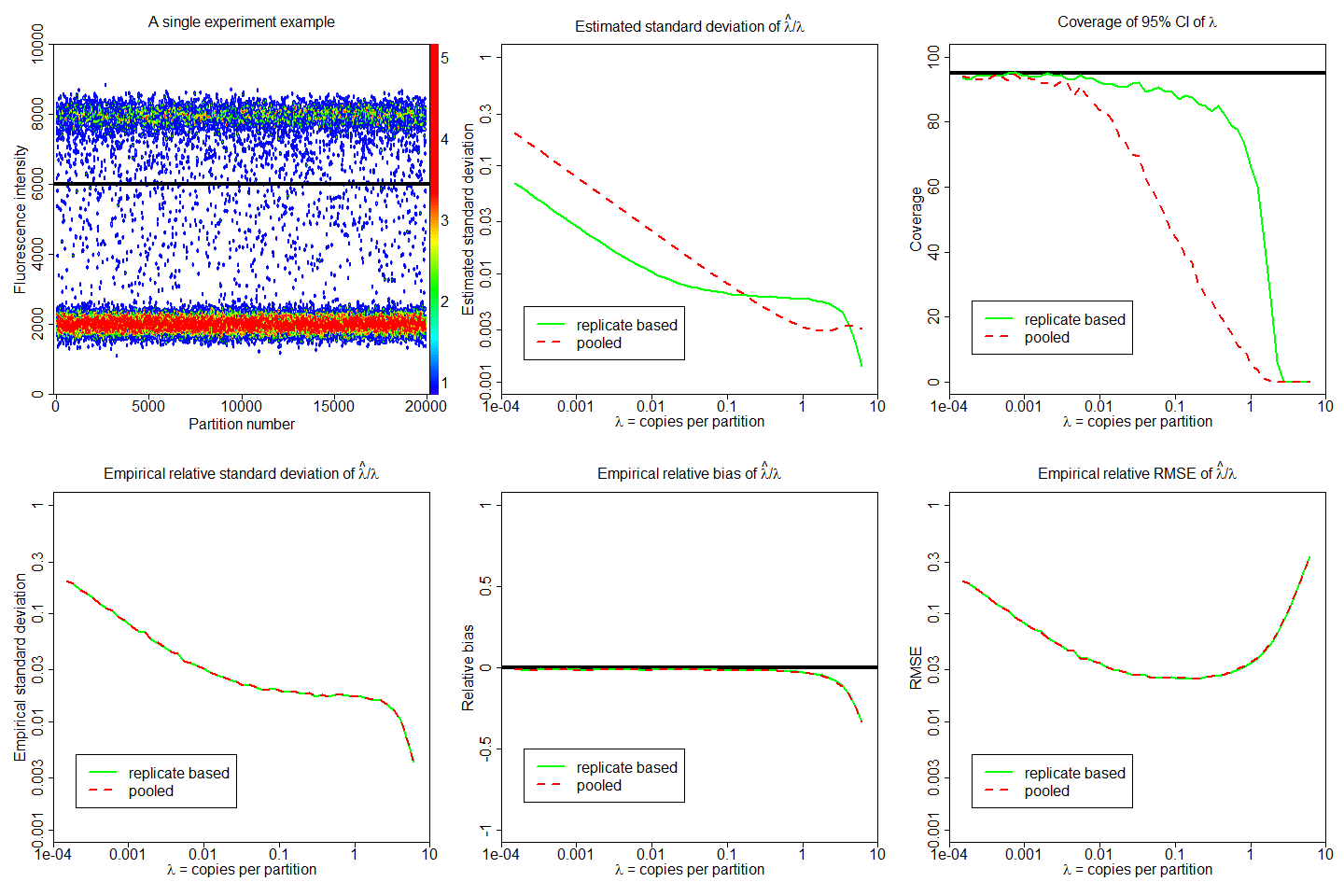

Supplement: Supplementary file 4 — Additional file 4: Interactive tool. In this mini-website, we provide an interactive tool to study the influence of specific sources of variation on the performance of the concentration estimators. This can serve as a guide when designing an experiment. All results are relative to the true concentration and based on 1000 simulations with 8 technical replicates. (ZIP 17 MB) [file 12859_2014_6687_MOESM4_ESM.zip › Additional file 4/RES/RES2432B.png]

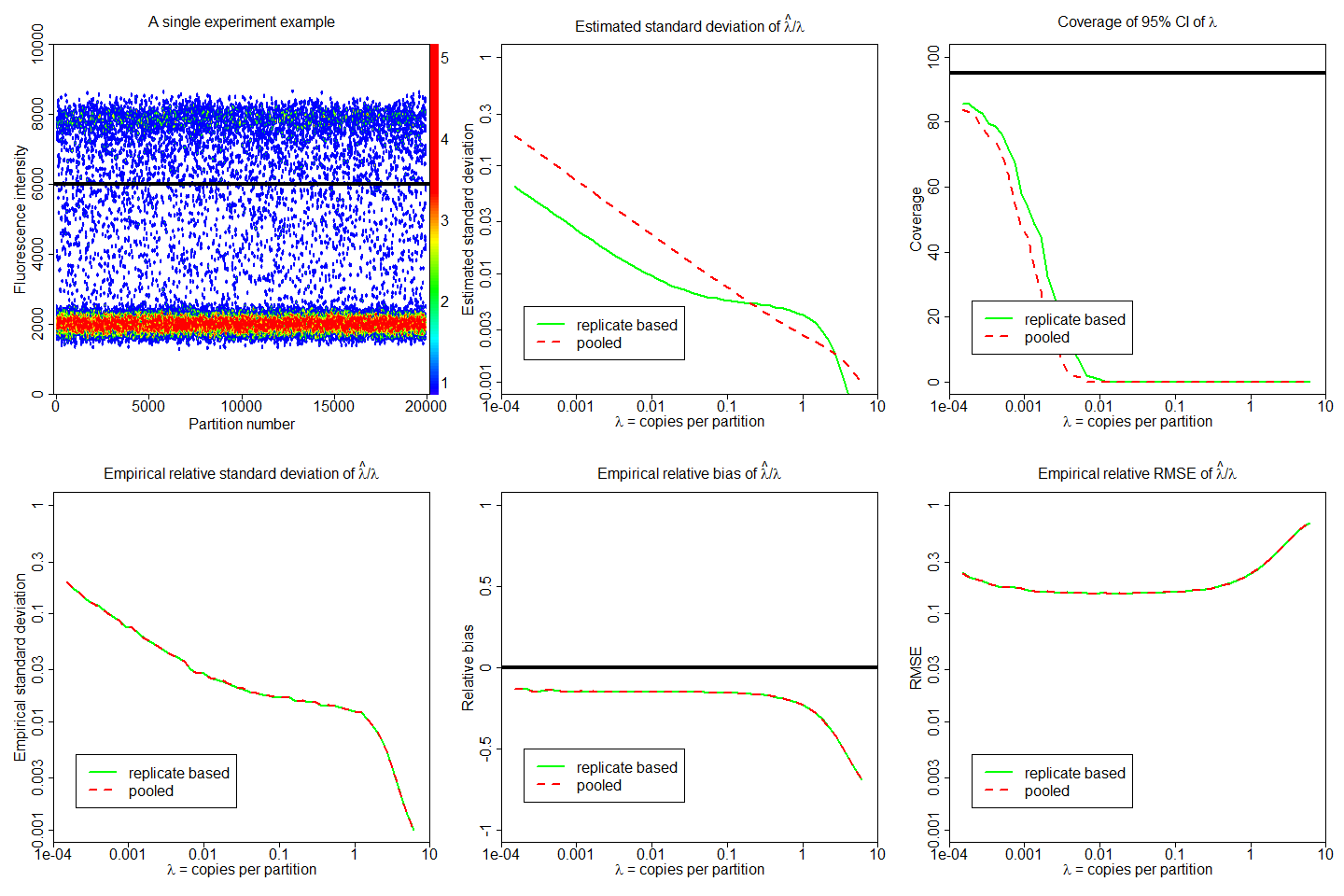

Supplement: Supplementary file 4 — Additional file 4: Interactive tool. In this mini-website, we provide an interactive tool to study the influence of specific sources of variation on the performance of the concentration estimators. This can serve as a guide when designing an experiment. All results are relative to the true concentration and based on 1000 simulations with 8 technical replicates. (ZIP 17 MB) [file 12859_2014_6687_MOESM4_ESM.zip › Additional file 4/RES/RES2433B.png]

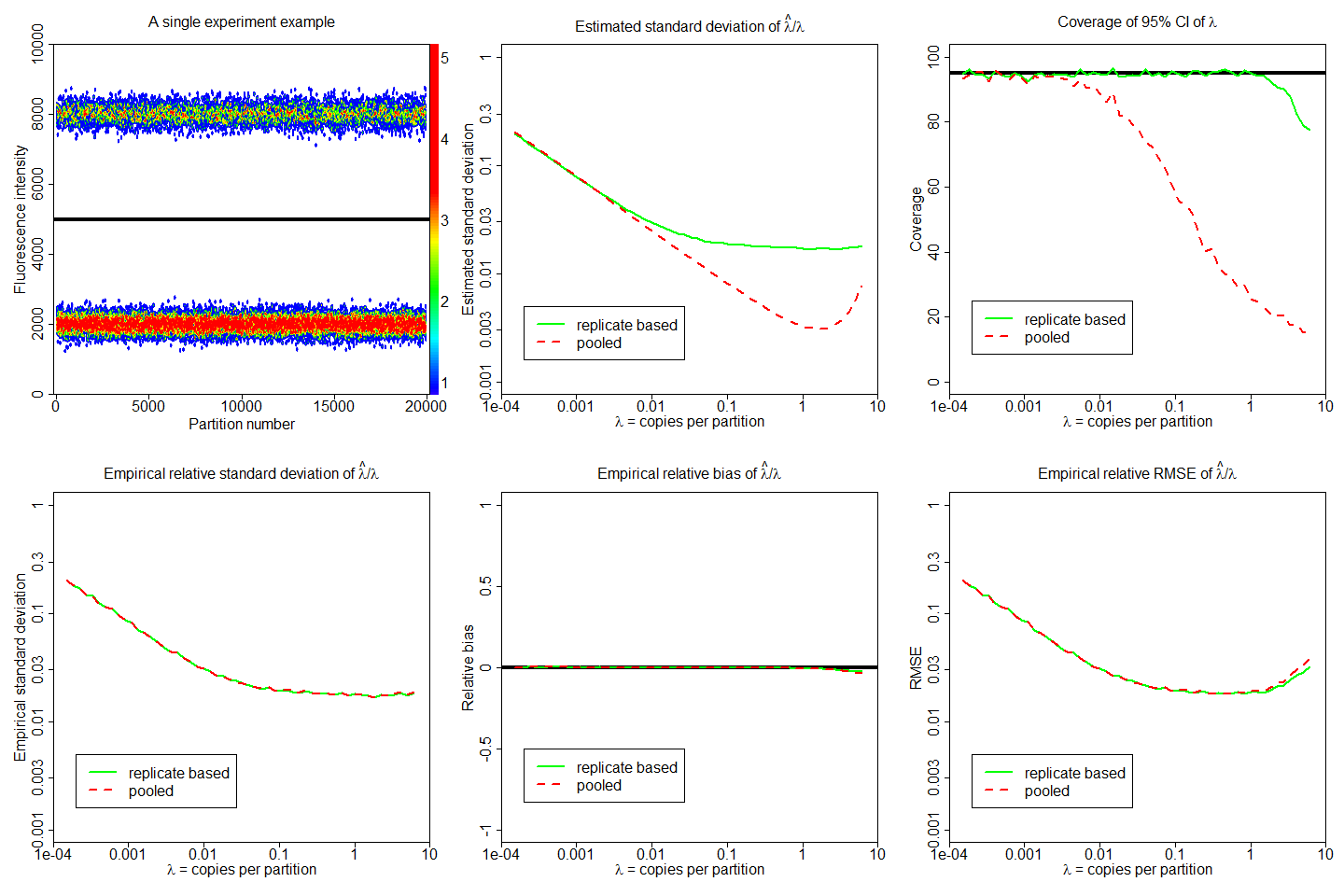

Supplement: Supplementary file 4 — Additional file 4: Interactive tool. In this mini-website, we provide an interactive tool to study the influence of specific sources of variation on the performance of the concentration estimators. This can serve as a guide when designing an experiment. All results are relative to the true concentration and based on 1000 simulations with 8 technical replicates. (ZIP 17 MB) [file 12859_2014_6687_MOESM4_ESM.zip › Additional file 4/RES/RES2441B.png]

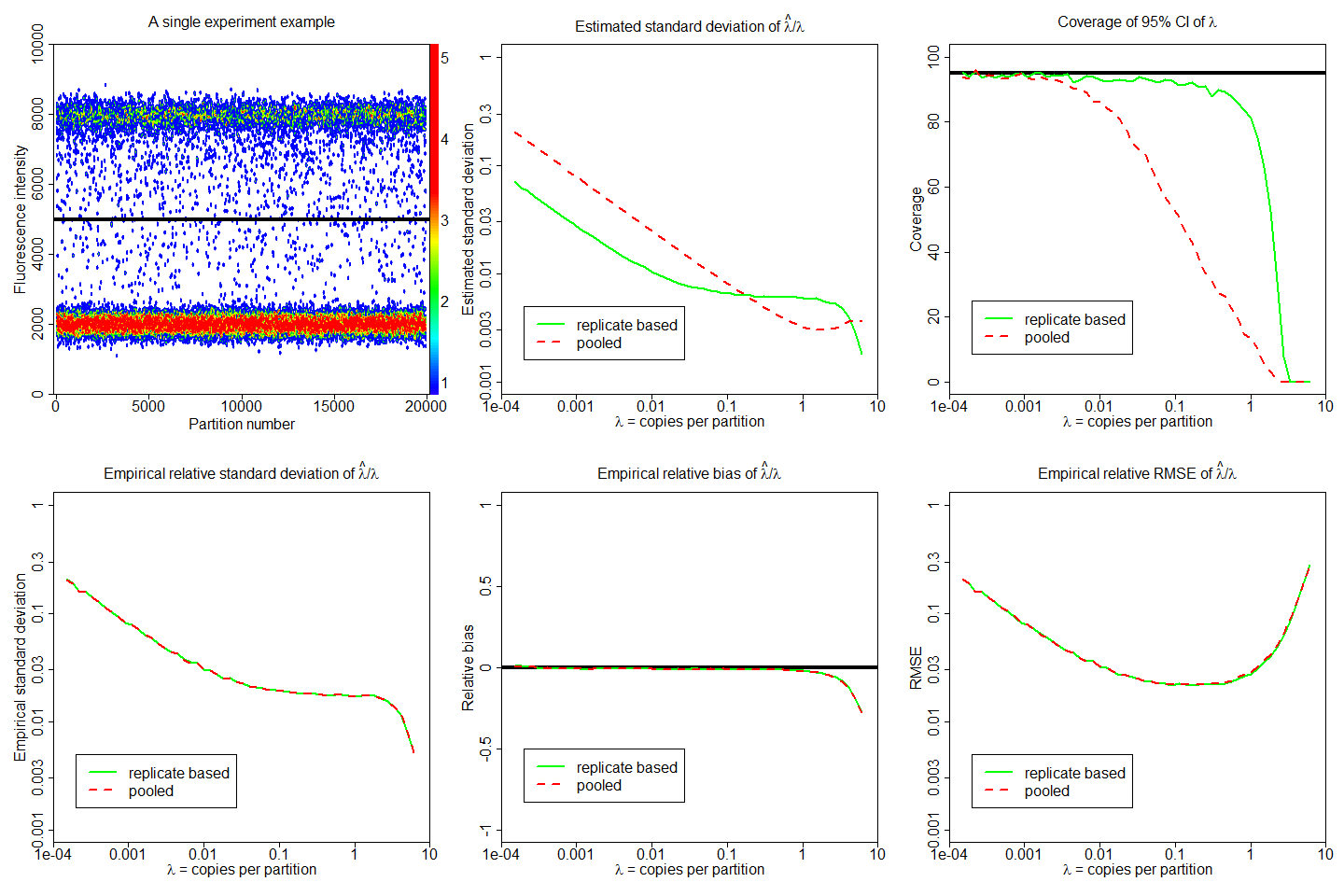

Supplement: Supplementary file 4 — Additional file 4: Interactive tool. In this mini-website, we provide an interactive tool to study the influence of specific sources of variation on the performance of the concentration estimators. This can serve as a guide when designing an experiment. All results are relative to the true concentration and based on 1000 simulations with 8 technical replicates. (ZIP 17 MB) [file 12859_2014_6687_MOESM4_ESM.zip › Additional file 4/RES/RES2442B.png]

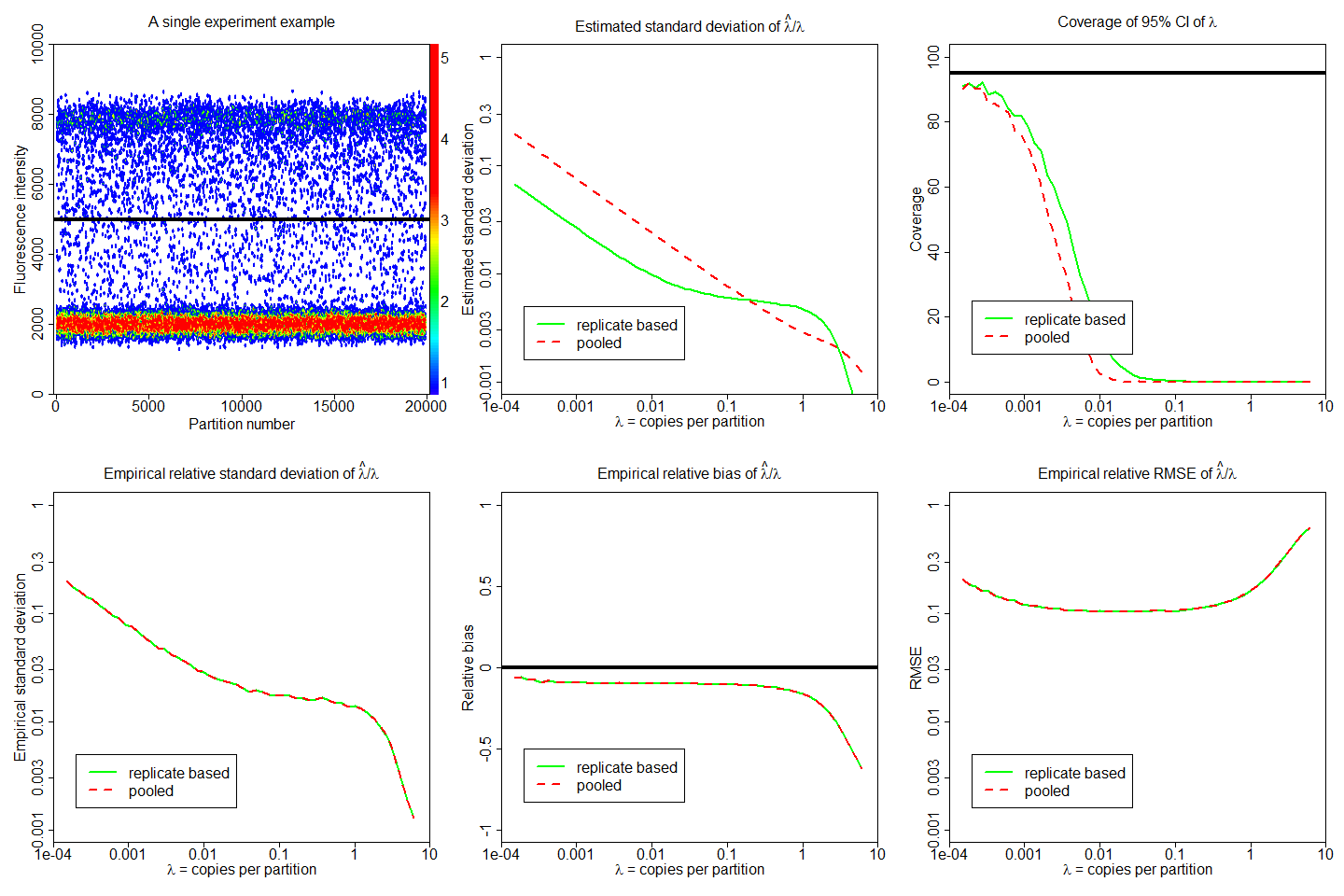

Supplement: Supplementary file 4 — Additional file 4: Interactive tool. In this mini-website, we provide an interactive tool to study the influence of specific sources of variation on the performance of the concentration estimators. This can serve as a guide when designing an experiment. All results are relative to the true concentration and based on 1000 simulations with 8 technical replicates. (ZIP 17 MB) [file 12859_2014_6687_MOESM4_ESM.zip › Additional file 4/RES/RES2443B.png]

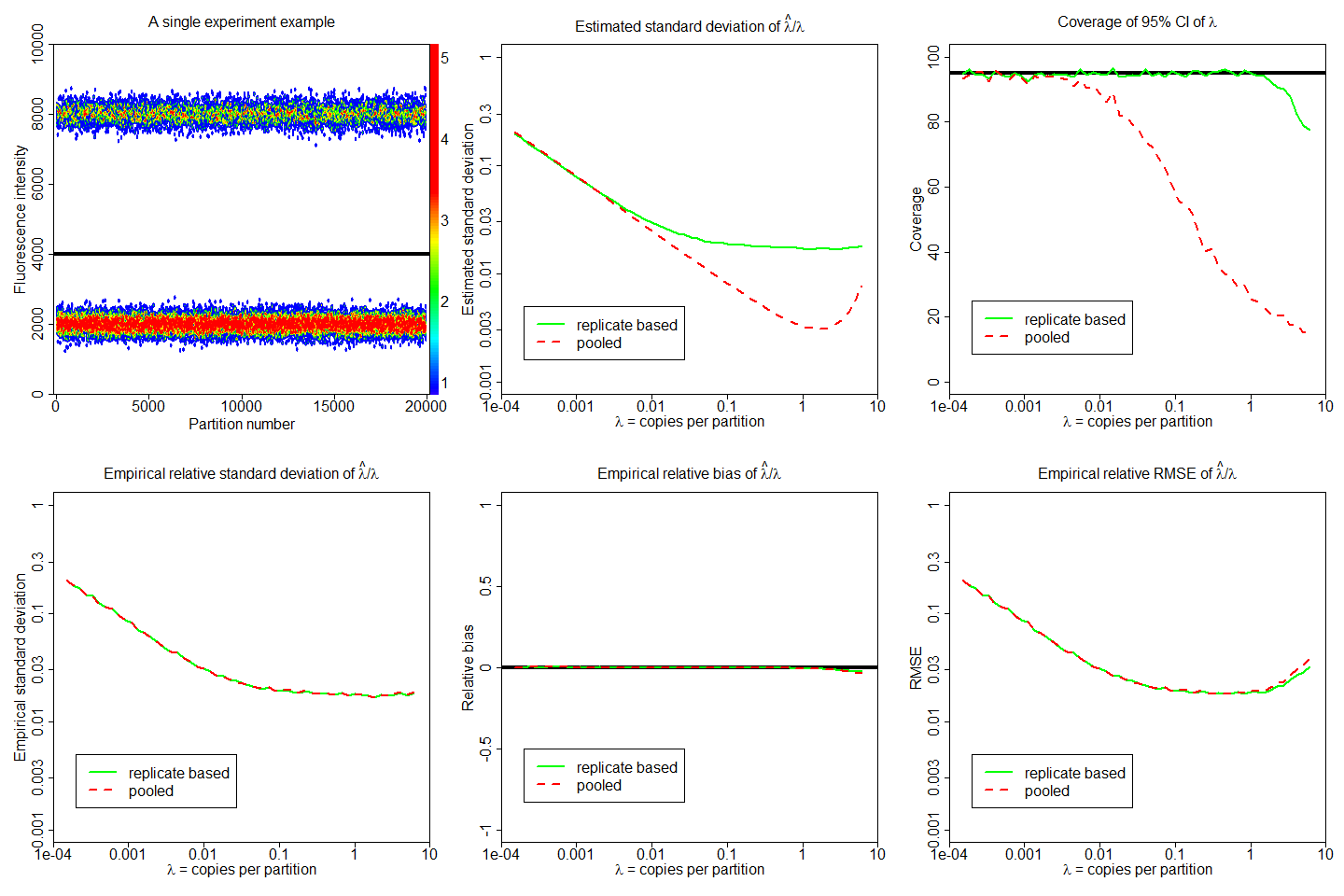

Supplement: Supplementary file 4 — Additional file 4: Interactive tool. In this mini-website, we provide an interactive tool to study the influence of specific sources of variation on the performance of the concentration estimators. This can serve as a guide when designing an experiment. All results are relative to the true concentration and based on 1000 simulations with 8 technical replicates. (ZIP 17 MB) [file 12859_2014_6687_MOESM4_ESM.zip › Additional file 4/RES/RES2451B.png]

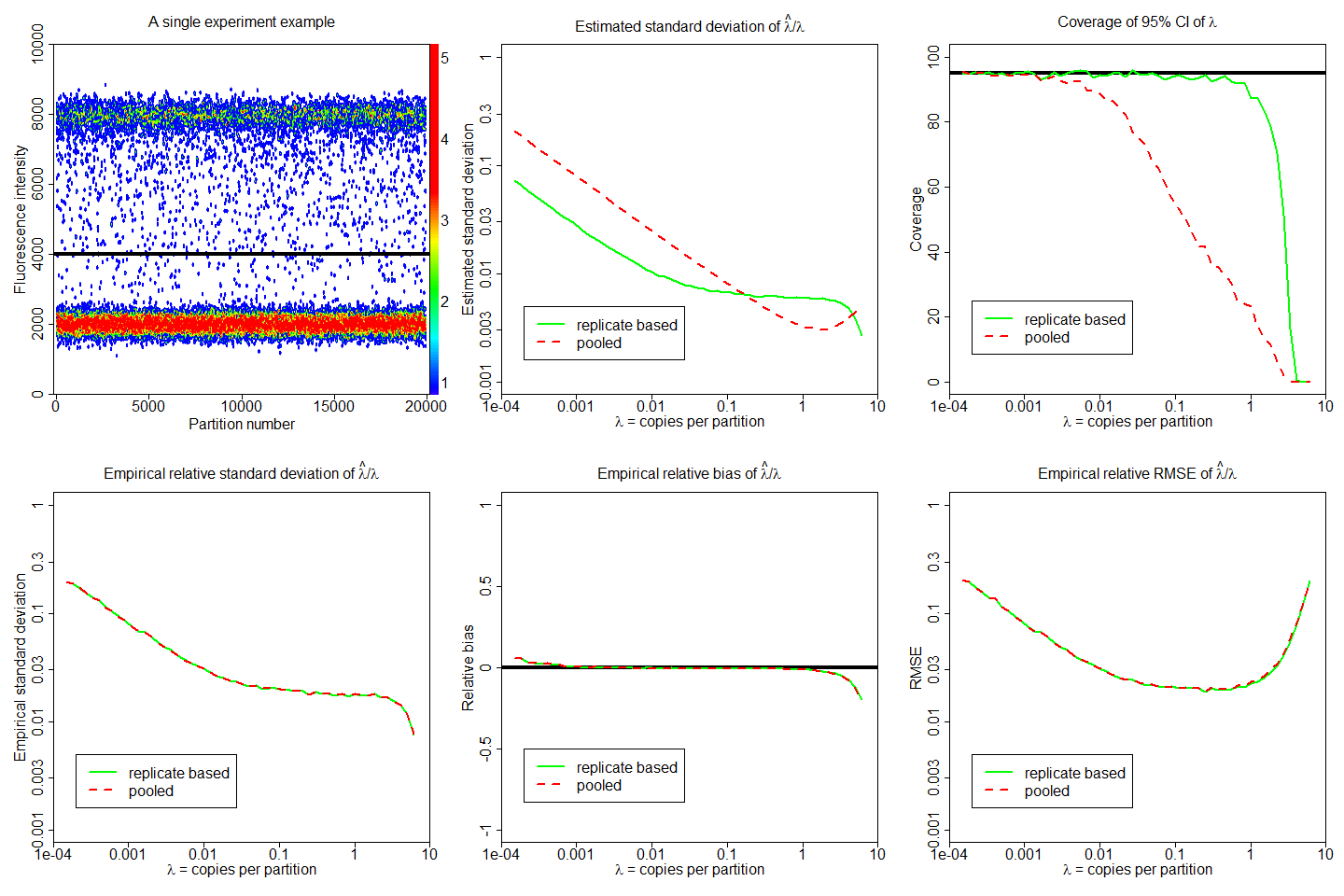

Supplement: Supplementary file 4 — Additional file 4: Interactive tool. In this mini-website, we provide an interactive tool to study the influence of specific sources of variation on the performance of the concentration estimators. This can serve as a guide when designing an experiment. All results are relative to the true concentration and based on 1000 simulations with 8 technical replicates. (ZIP 17 MB) [file 12859_2014_6687_MOESM4_ESM.zip › Additional file 4/RES/RES2452B.png]

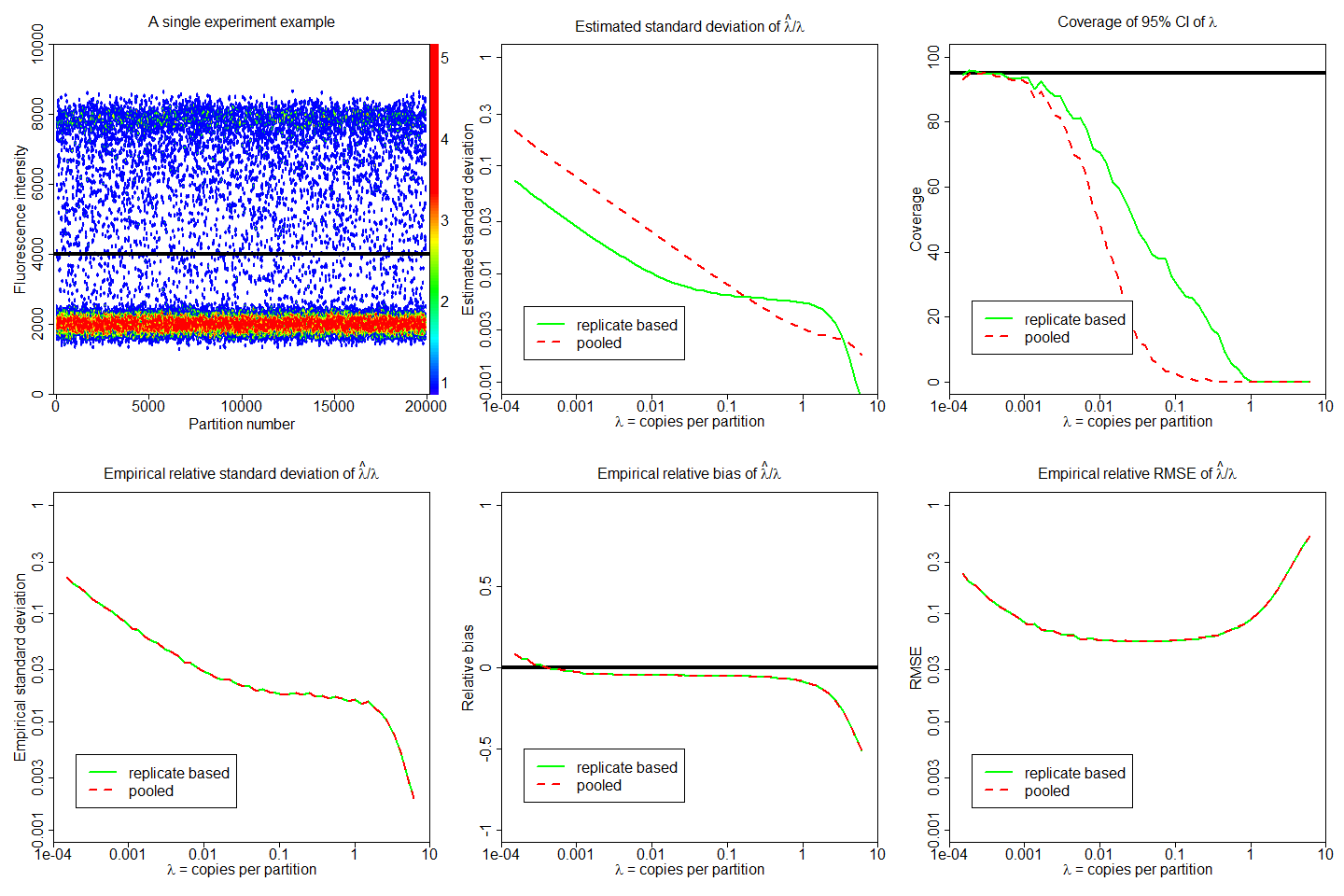

Supplement: Supplementary file 4 — Additional file 4: Interactive tool. In this mini-website, we provide an interactive tool to study the influence of specific sources of variation on the performance of the concentration estimators. This can serve as a guide when designing an experiment. All results are relative to the true concentration and based on 1000 simulations with 8 technical replicates. (ZIP 17 MB) [file 12859_2014_6687_MOESM4_ESM.zip › Additional file 4/RES/RES2453B.png]

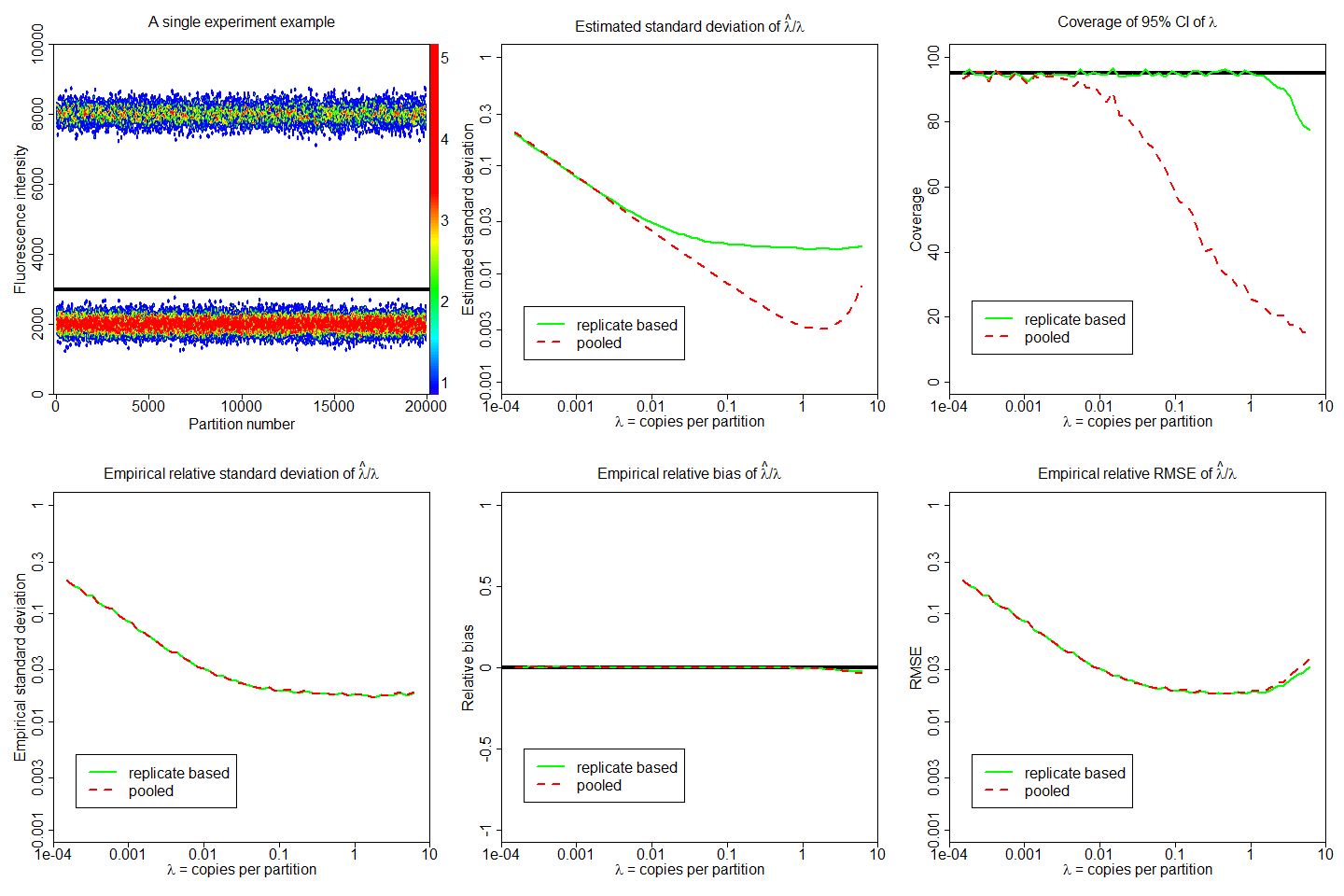

Supplement: Supplementary file 4 — Additional file 4: Interactive tool. In this mini-website, we provide an interactive tool to study the influence of specific sources of variation on the performance of the concentration estimators. This can serve as a guide when designing an experiment. All results are relative to the true concentration and based on 1000 simulations with 8 technical replicates. (ZIP 17 MB) [file 12859_2014_6687_MOESM4_ESM.zip › Additional file 4/RES/RES2461B.png]

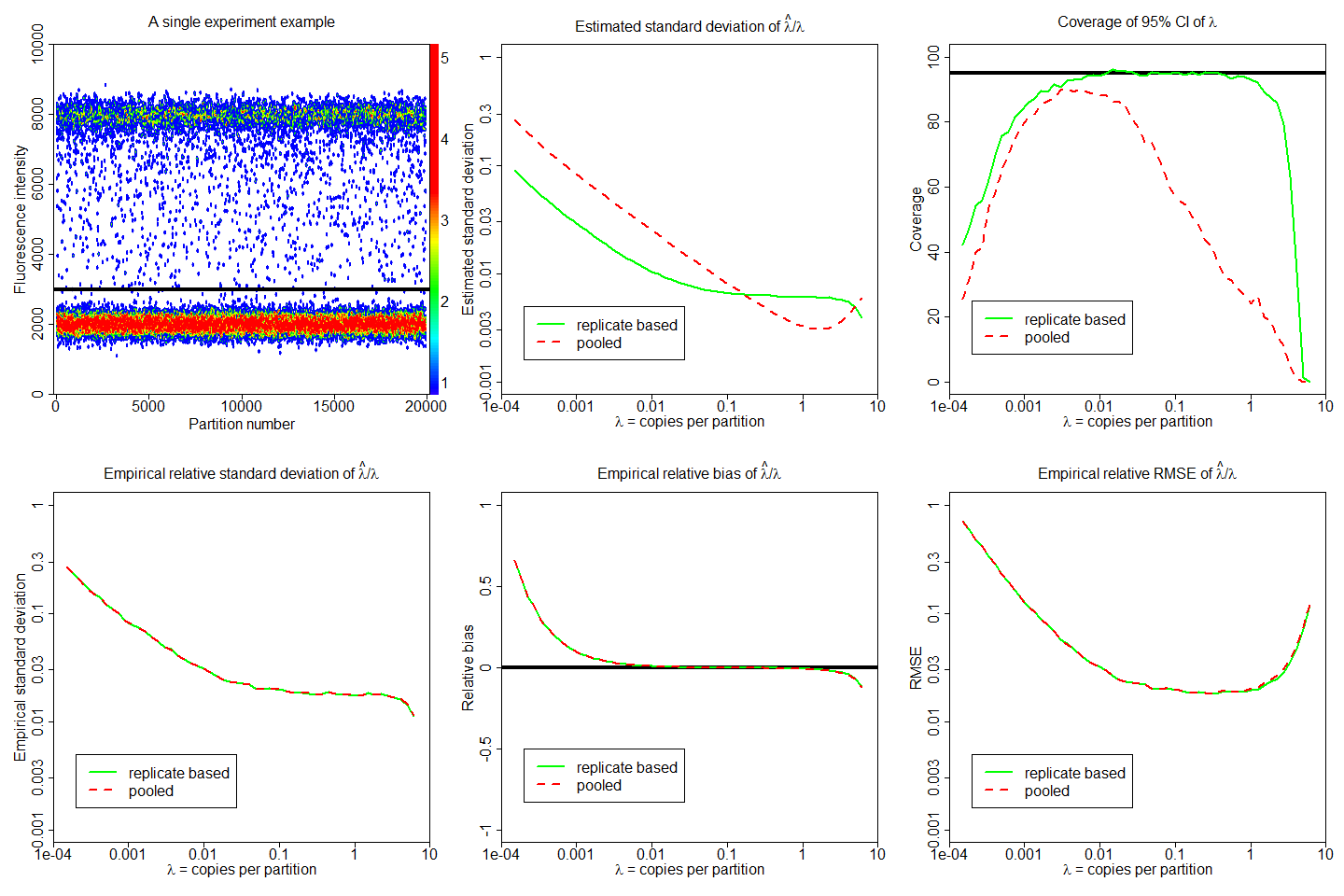

Supplement: Supplementary file 4 — Additional file 4: Interactive tool. In this mini-website, we provide an interactive tool to study the influence of specific sources of variation on the performance of the concentration estimators. This can serve as a guide when designing an experiment. All results are relative to the true concentration and based on 1000 simulations with 8 technical replicates. (ZIP 17 MB) [file 12859_2014_6687_MOESM4_ESM.zip › Additional file 4/RES/RES2462B.png]

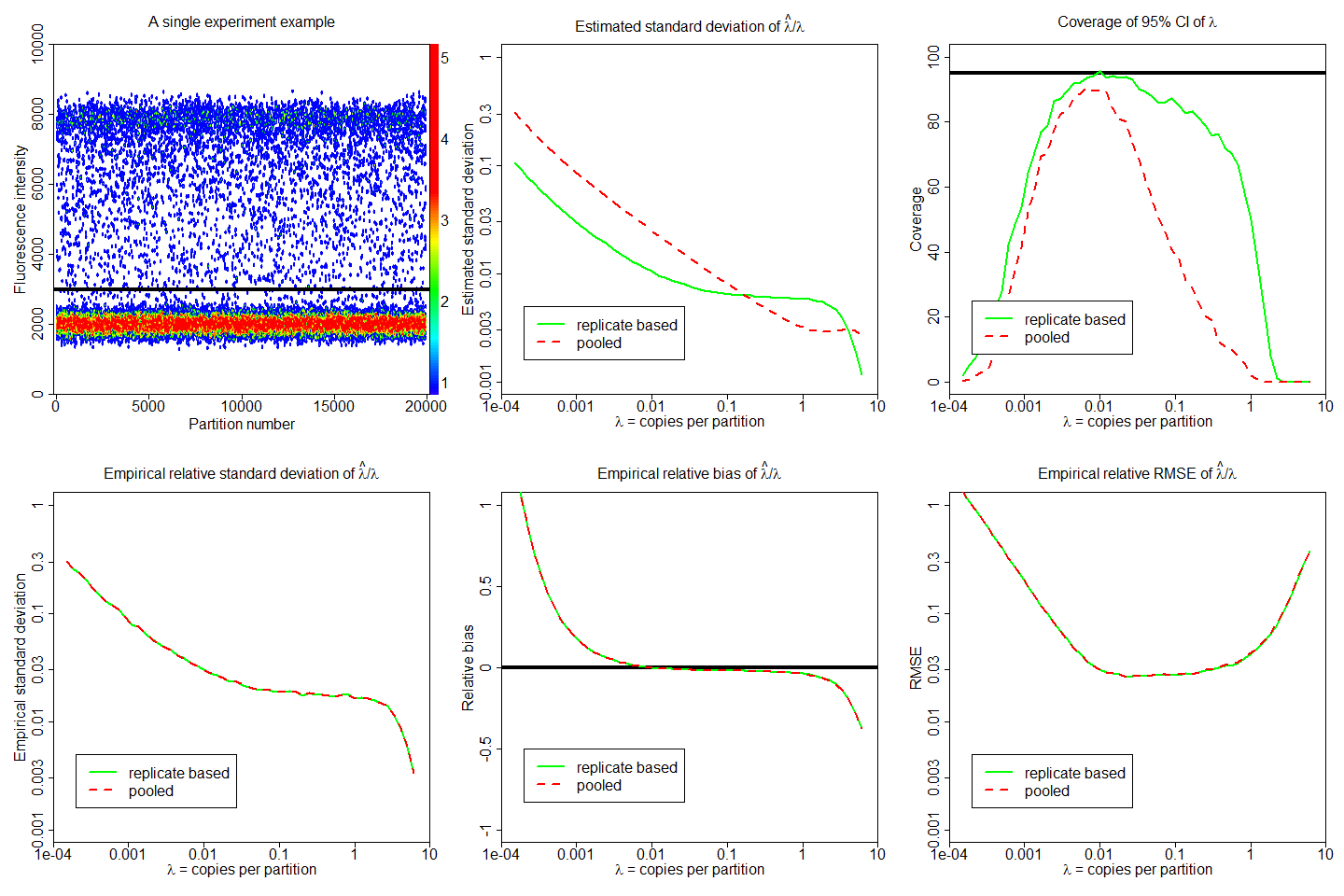

Supplement: Supplementary file 4 — Additional file 4: Interactive tool. In this mini-website, we provide an interactive tool to study the influence of specific sources of variation on the performance of the concentration estimators. This can serve as a guide when designing an experiment. All results are relative to the true concentration and based on 1000 simulations with 8 technical replicates. (ZIP 17 MB) [file 12859_2014_6687_MOESM4_ESM.zip › Additional file 4/RES/RES2463B.png]

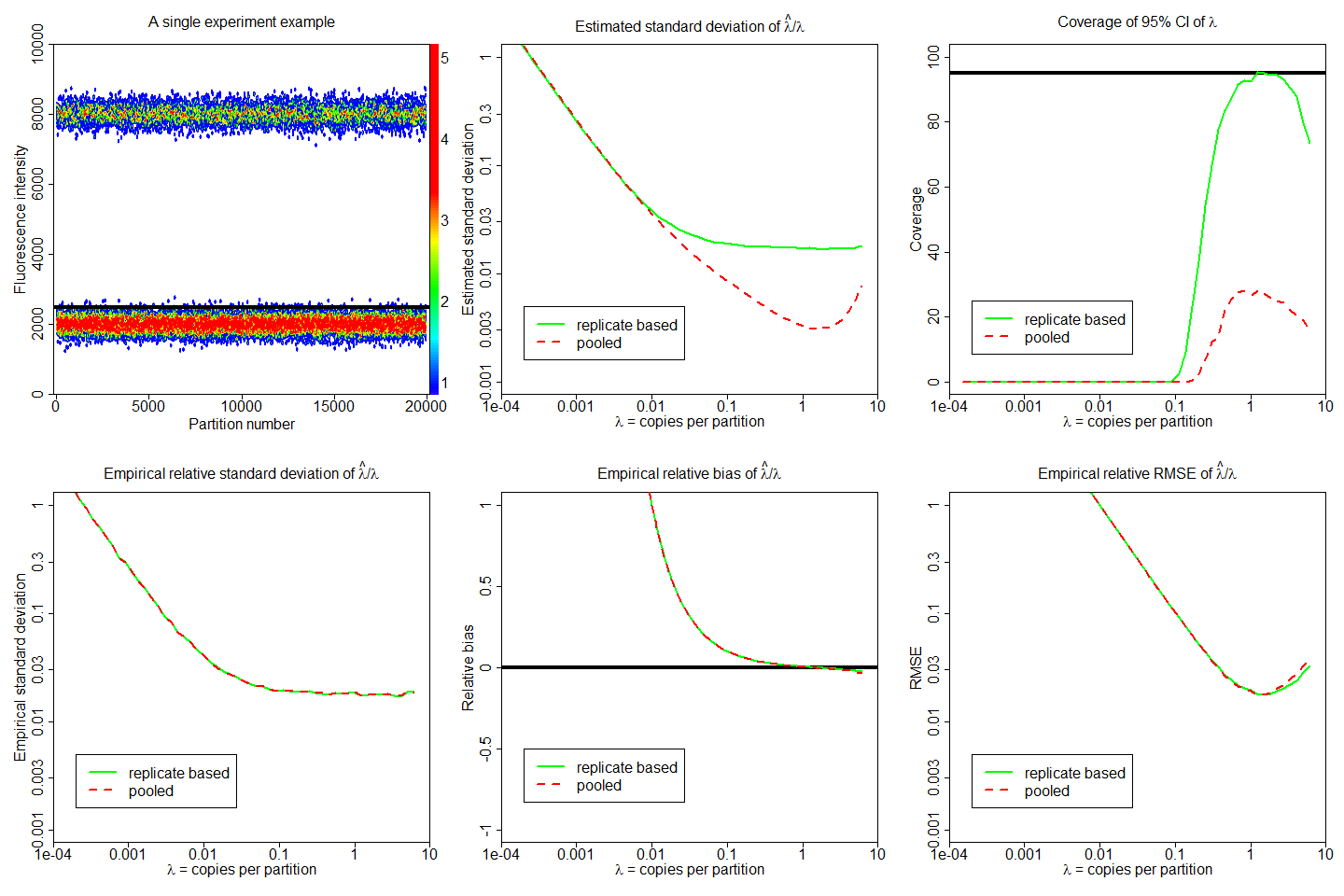

Supplement: Supplementary file 4 — Additional file 4: Interactive tool. In this mini-website, we provide an interactive tool to study the influence of specific sources of variation on the performance of the concentration estimators. This can serve as a guide when designing an experiment. All results are relative to the true concentration and based on 1000 simulations with 8 technical replicates. (ZIP 17 MB) [file 12859_2014_6687_MOESM4_ESM.zip › Additional file 4/RES/RES2471B.png]

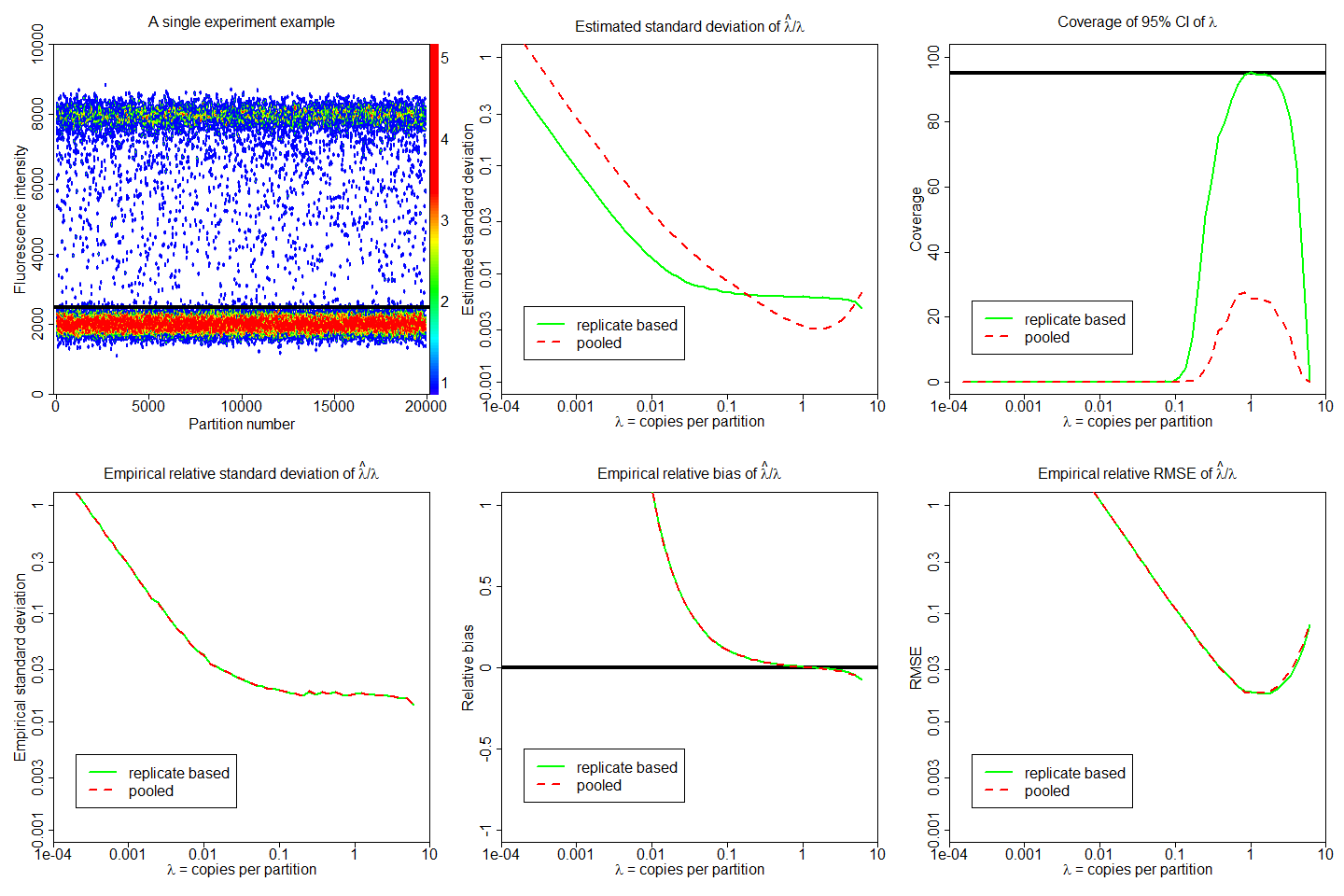

Supplement: Supplementary file 4 — Additional file 4: Interactive tool. In this mini-website, we provide an interactive tool to study the influence of specific sources of variation on the performance of the concentration estimators. This can serve as a guide when designing an experiment. All results are relative to the true concentration and based on 1000 simulations with 8 technical replicates. (ZIP 17 MB) [file 12859_2014_6687_MOESM4_ESM.zip › Additional file 4/RES/RES2472B.png]

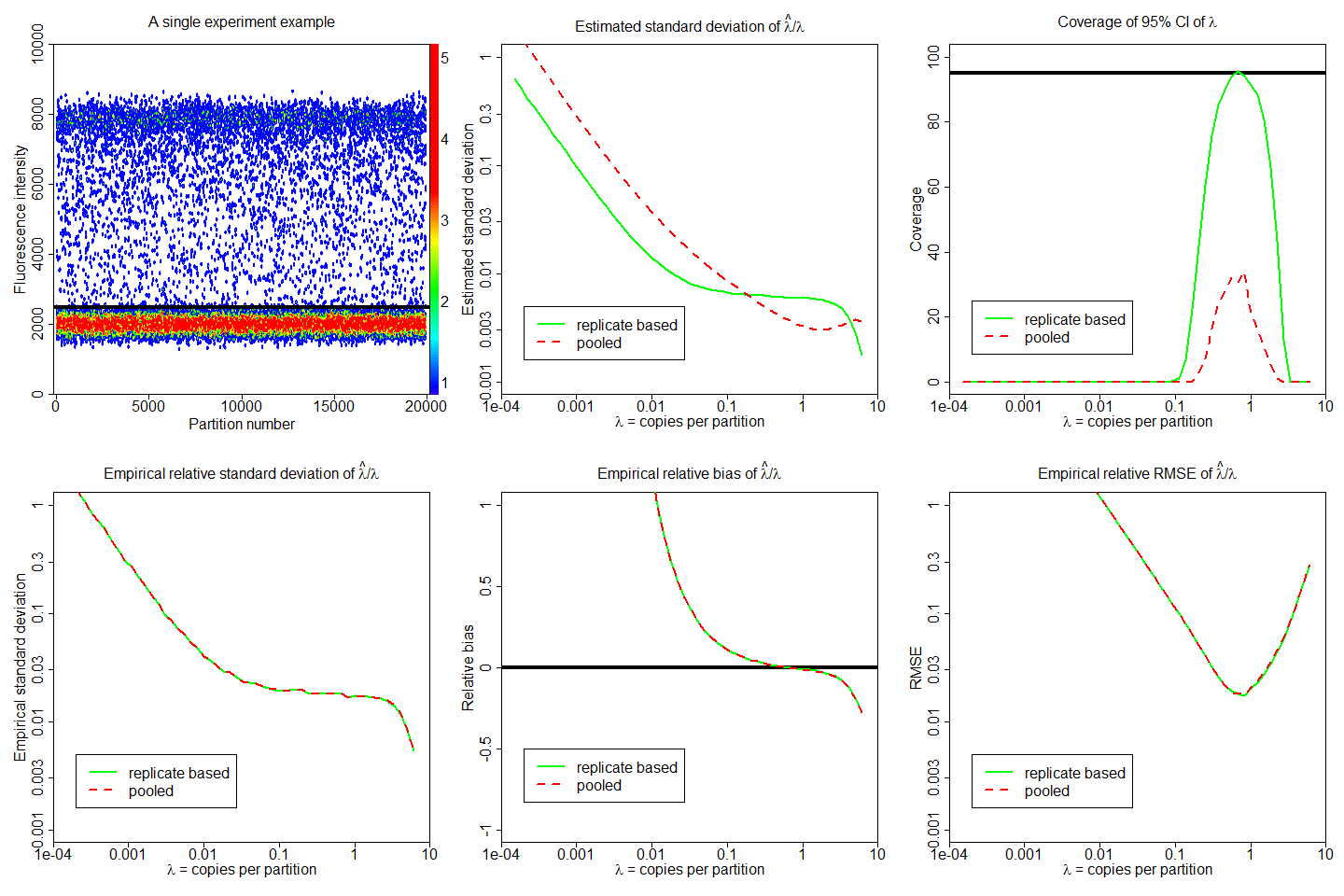

Supplement: Supplementary file 4 — Additional file 4: Interactive tool. In this mini-website, we provide an interactive tool to study the influence of specific sources of variation on the performance of the concentration estimators. This can serve as a guide when designing an experiment. All results are relative to the true concentration and based on 1000 simulations with 8 technical replicates. (ZIP 17 MB) [file 12859_2014_6687_MOESM4_ESM.zip › Additional file 4/RES/RES2473B.png]

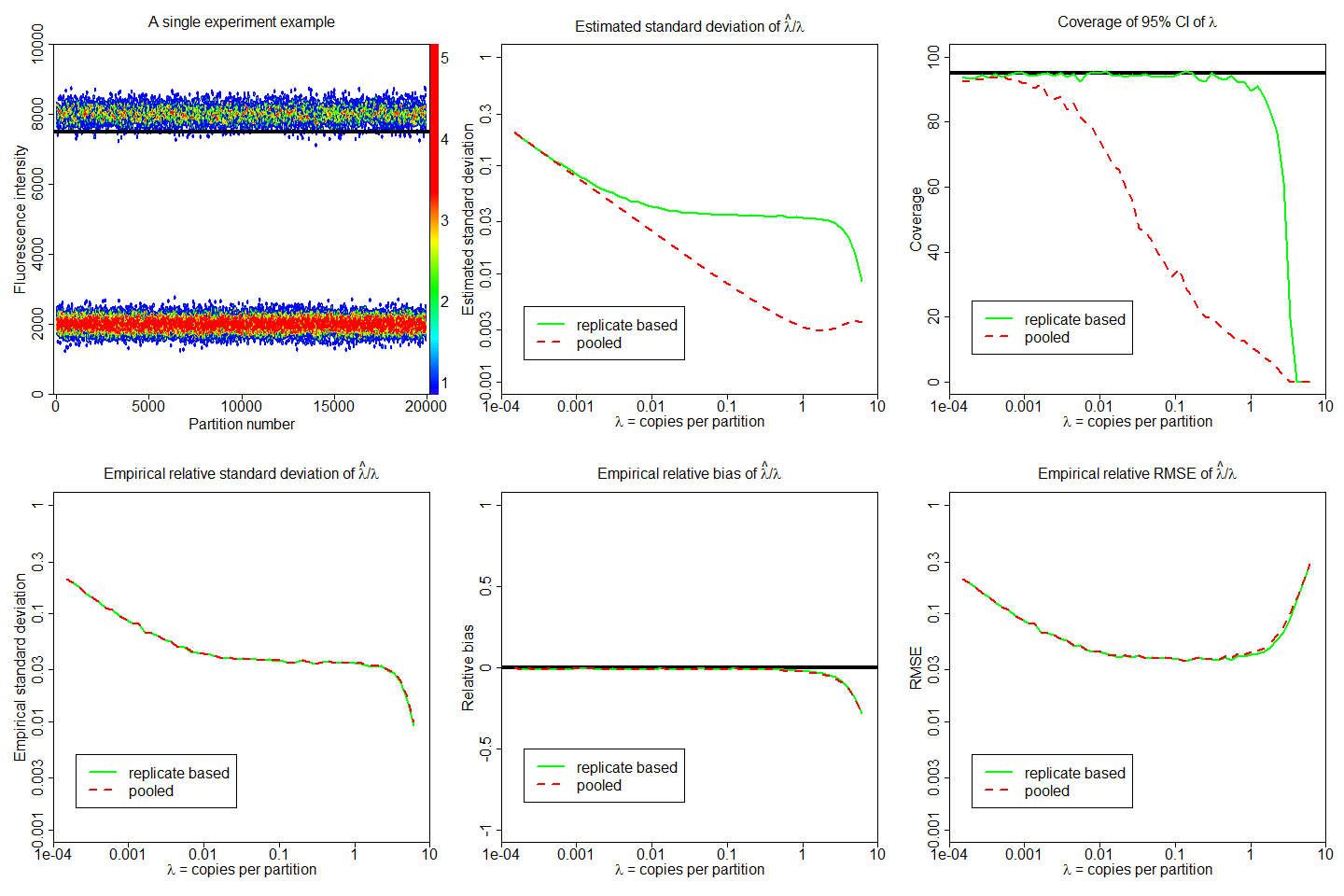

Supplement: Supplementary file 4 — Additional file 4: Interactive tool. In this mini-website, we provide an interactive tool to study the influence of specific sources of variation on the performance of the concentration estimators. This can serve as a guide when designing an experiment. All results are relative to the true concentration and based on 1000 simulations with 8 technical replicates. (ZIP 17 MB) [file 12859_2014_6687_MOESM4_ESM.zip › Additional file 4/RES/RES2511B.png]

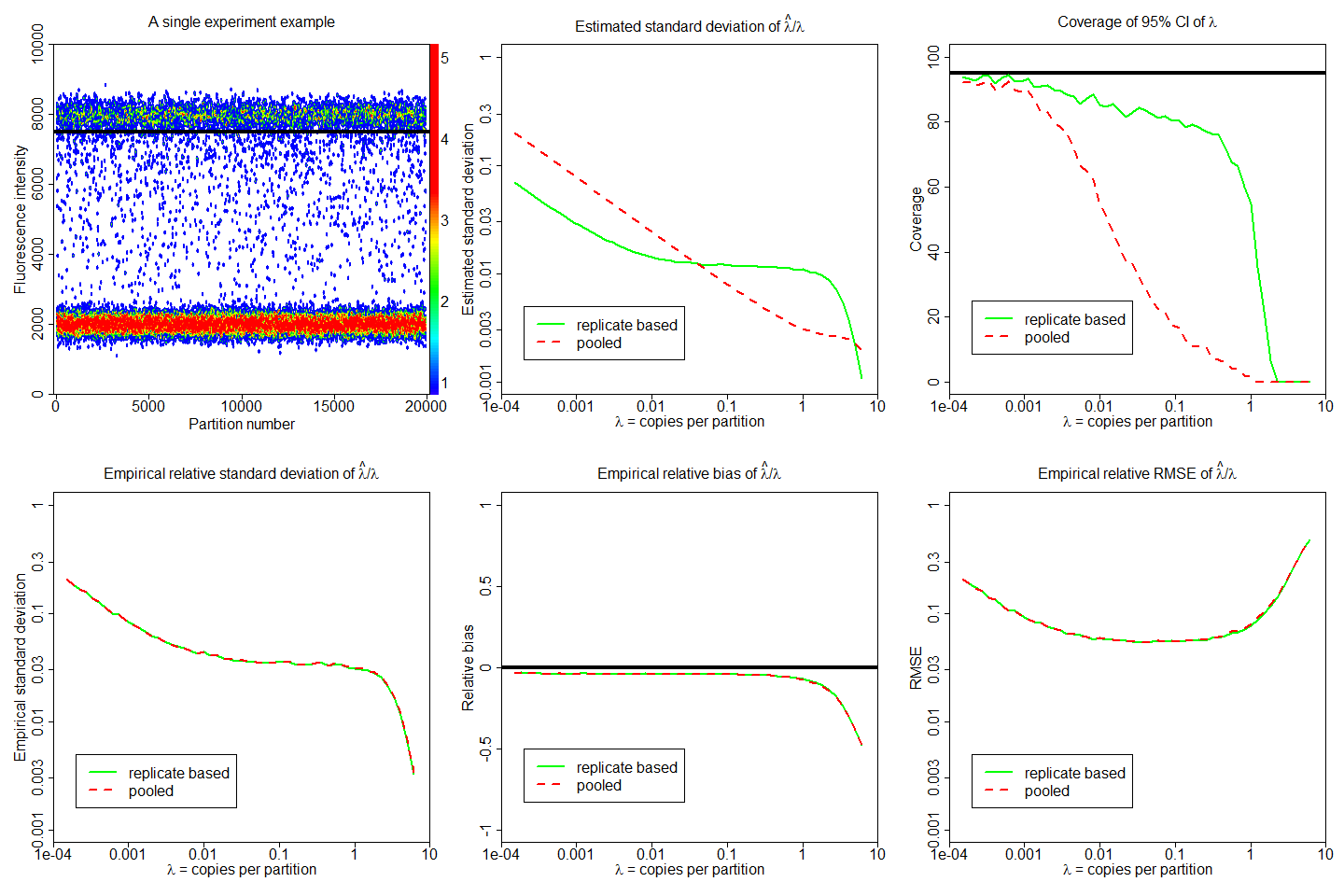

Supplement: Supplementary file 4 — Additional file 4: Interactive tool. In this mini-website, we provide an interactive tool to study the influence of specific sources of variation on the performance of the concentration estimators. This can serve as a guide when designing an experiment. All results are relative to the true concentration and based on 1000 simulations with 8 technical replicates. (ZIP 17 MB) [file 12859_2014_6687_MOESM4_ESM.zip › Additional file 4/RES/RES2512B.png]

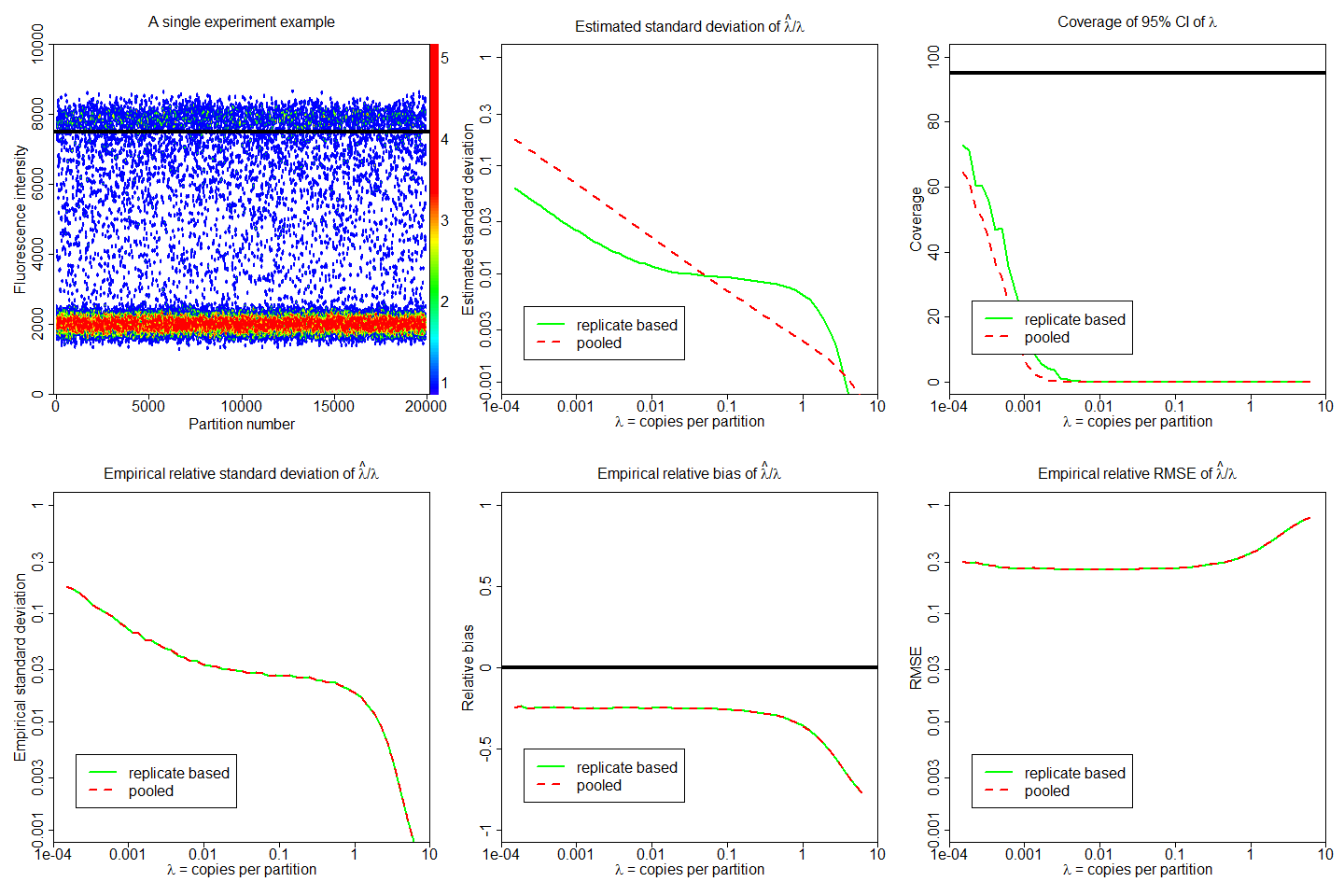

Supplement: Supplementary file 4 — Additional file 4: Interactive tool. In this mini-website, we provide an interactive tool to study the influence of specific sources of variation on the performance of the concentration estimators. This can serve as a guide when designing an experiment. All results are relative to the true concentration and based on 1000 simulations with 8 technical replicates. (ZIP 17 MB) [file 12859_2014_6687_MOESM4_ESM.zip › Additional file 4/RES/RES2513B.png]

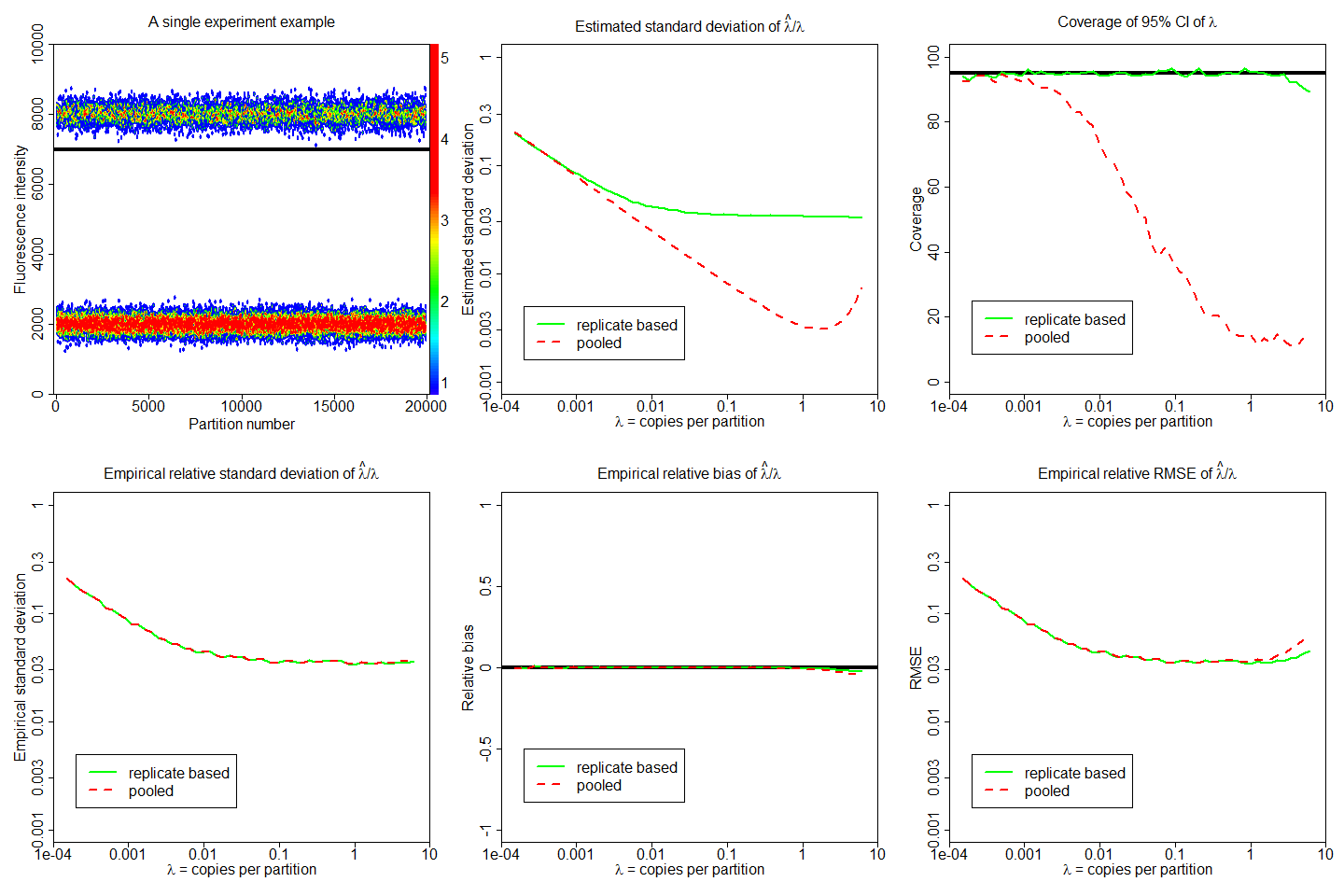

Supplement: Supplementary file 4 — Additional file 4: Interactive tool. In this mini-website, we provide an interactive tool to study the influence of specific sources of variation on the performance of the concentration estimators. This can serve as a guide when designing an experiment. All results are relative to the true concentration and based on 1000 simulations with 8 technical replicates. (ZIP 17 MB) [file 12859_2014_6687_MOESM4_ESM.zip › Additional file 4/RES/RES2521B.png]

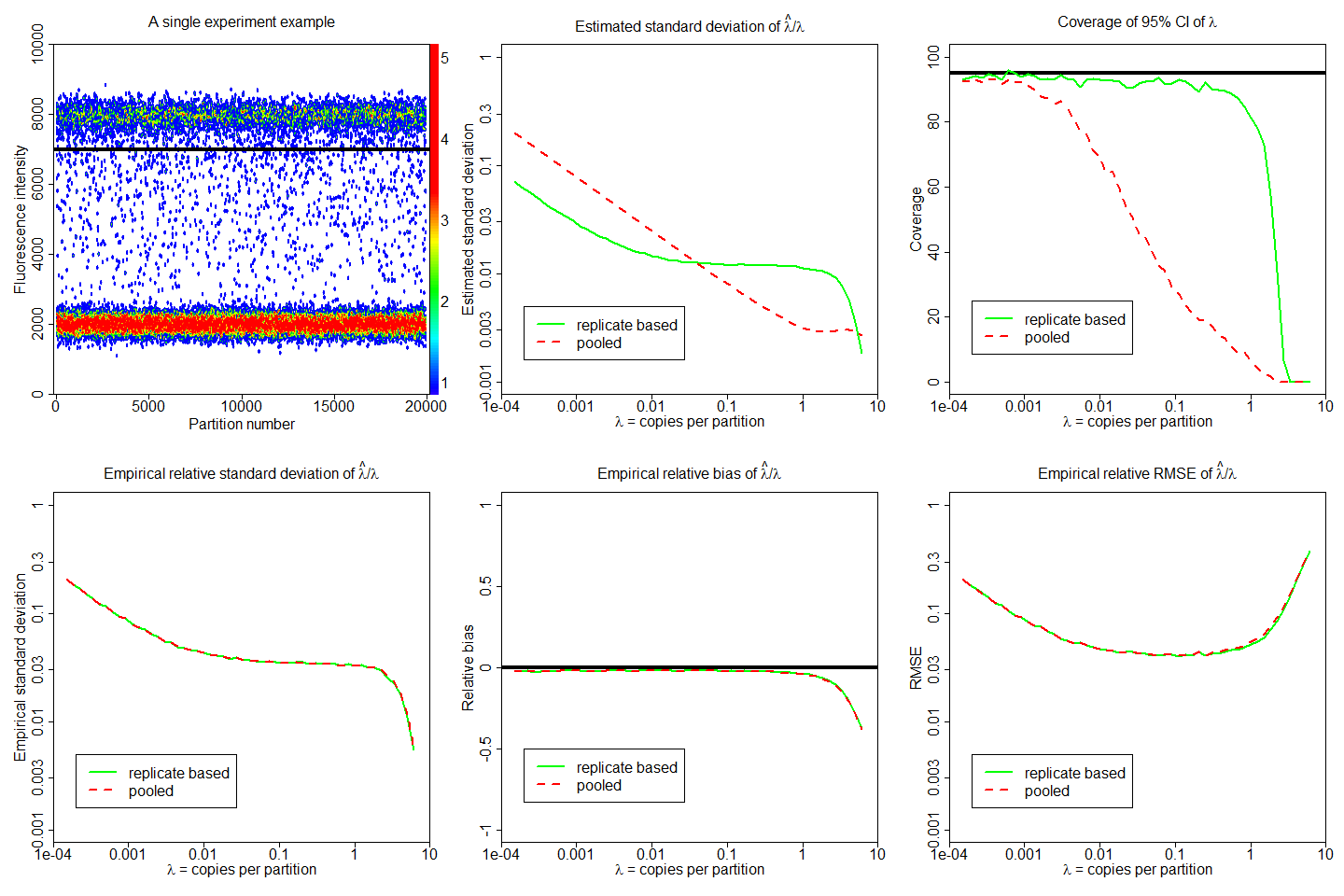

Supplement: Supplementary file 4 — Additional file 4: Interactive tool. In this mini-website, we provide an interactive tool to study the influence of specific sources of variation on the performance of the concentration estimators. This can serve as a guide when designing an experiment. All results are relative to the true concentration and based on 1000 simulations with 8 technical replicates. (ZIP 17 MB) [file 12859_2014_6687_MOESM4_ESM.zip › Additional file 4/RES/RES2522B.png]

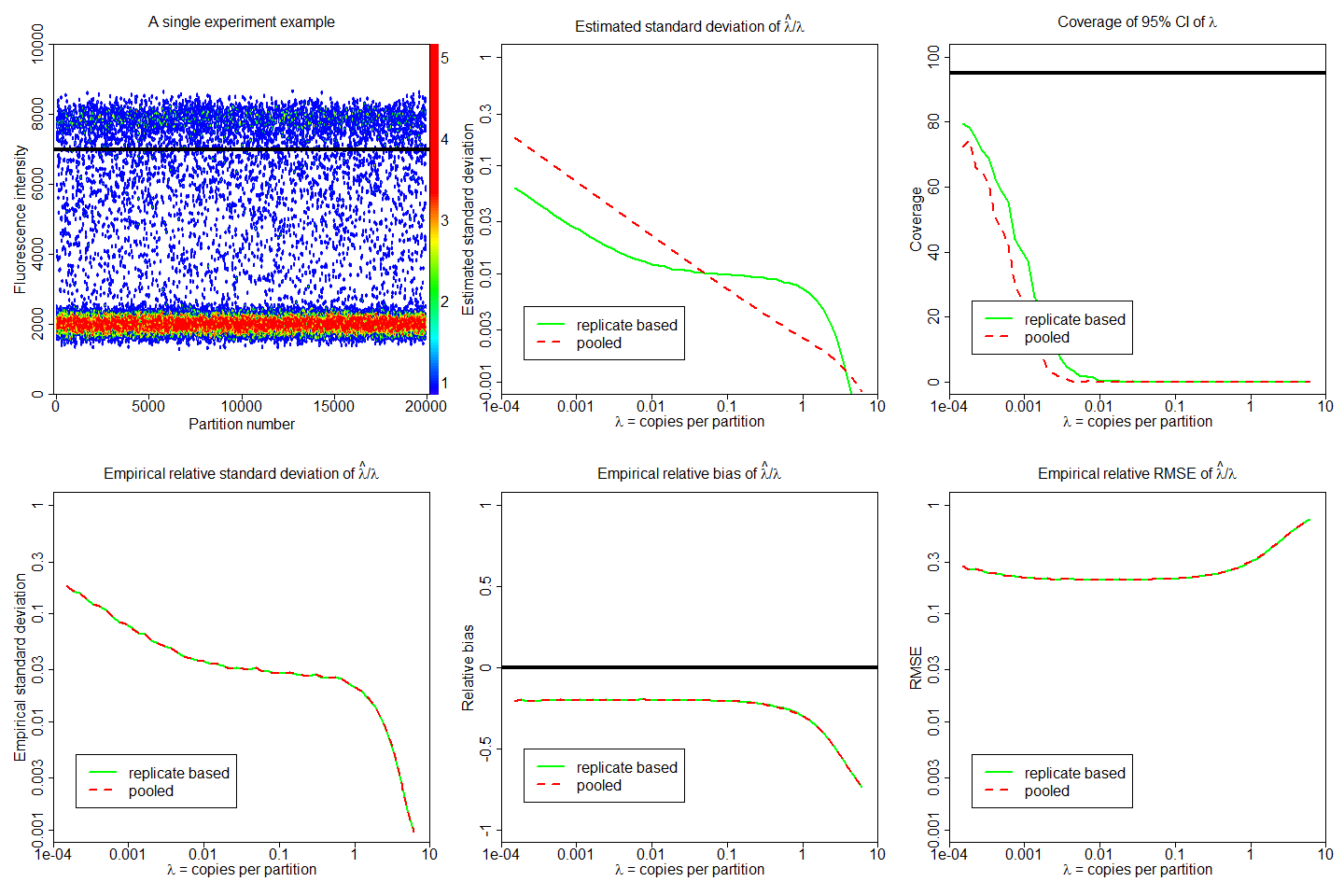

Supplement: Supplementary file 4 — Additional file 4: Interactive tool. In this mini-website, we provide an interactive tool to study the influence of specific sources of variation on the performance of the concentration estimators. This can serve as a guide when designing an experiment. All results are relative to the true concentration and based on 1000 simulations with 8 technical replicates. (ZIP 17 MB) [file 12859_2014_6687_MOESM4_ESM.zip › Additional file 4/RES/RES2523B.png]

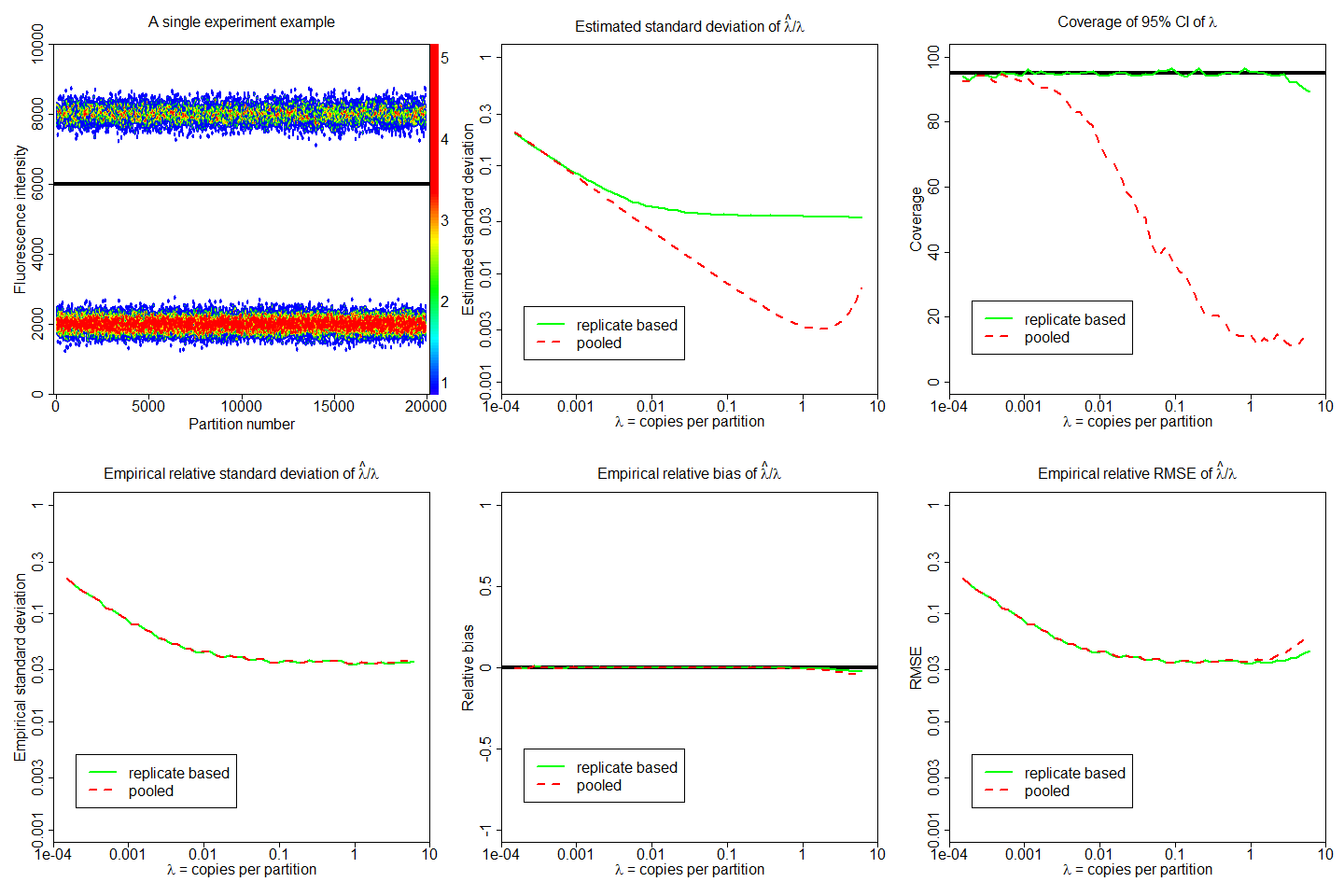

Supplement: Supplementary file 4 — Additional file 4: Interactive tool. In this mini-website, we provide an interactive tool to study the influence of specific sources of variation on the performance of the concentration estimators. This can serve as a guide when designing an experiment. All results are relative to the true concentration and based on 1000 simulations with 8 technical replicates. (ZIP 17 MB) [file 12859_2014_6687_MOESM4_ESM.zip › Additional file 4/RES/RES2531B.png]

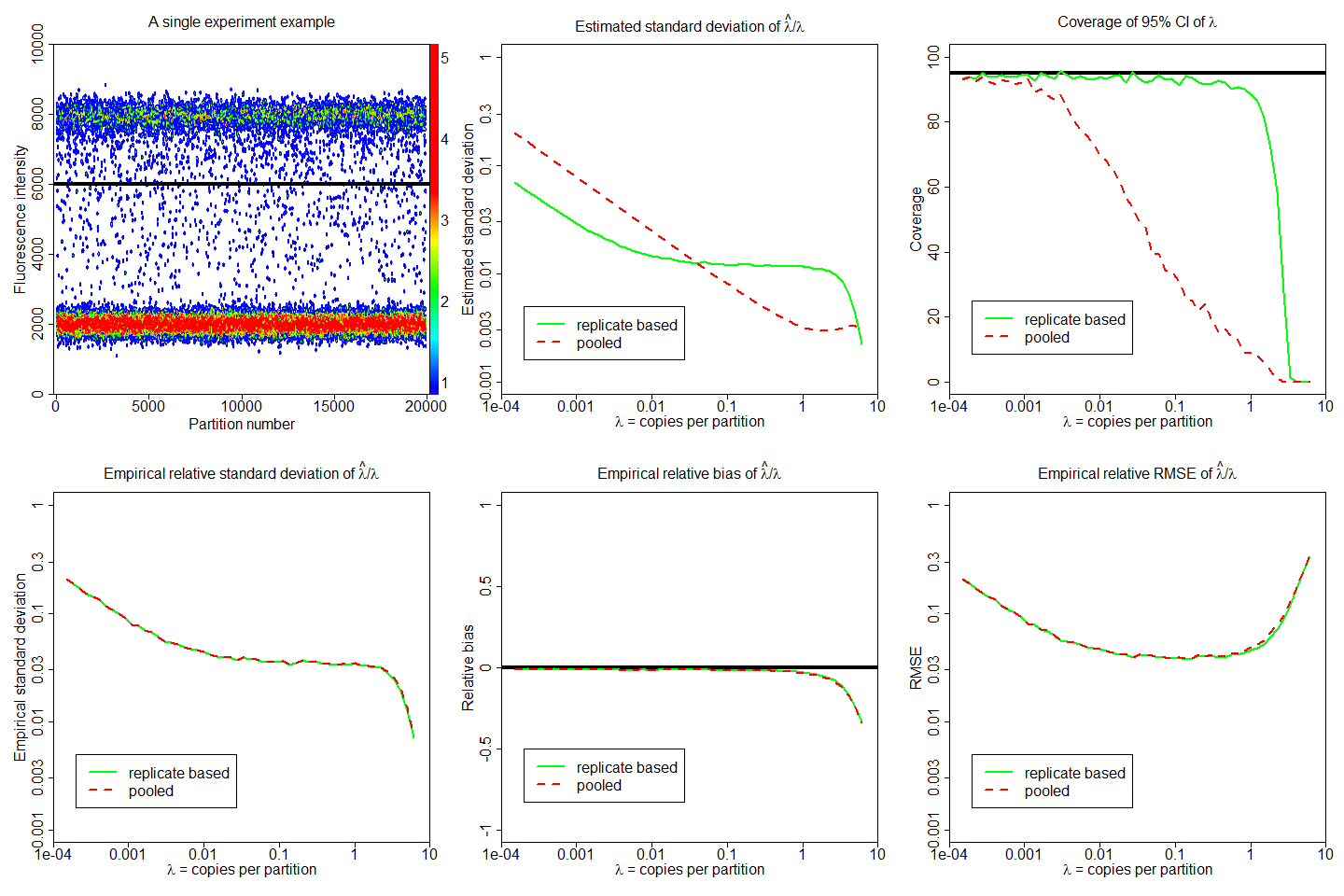

Supplement: Supplementary file 4 — Additional file 4: Interactive tool. In this mini-website, we provide an interactive tool to study the influence of specific sources of variation on the performance of the concentration estimators. This can serve as a guide when designing an experiment. All results are relative to the true concentration and based on 1000 simulations with 8 technical replicates. (ZIP 17 MB) [file 12859_2014_6687_MOESM4_ESM.zip › Additional file 4/RES/RES2532B.png]

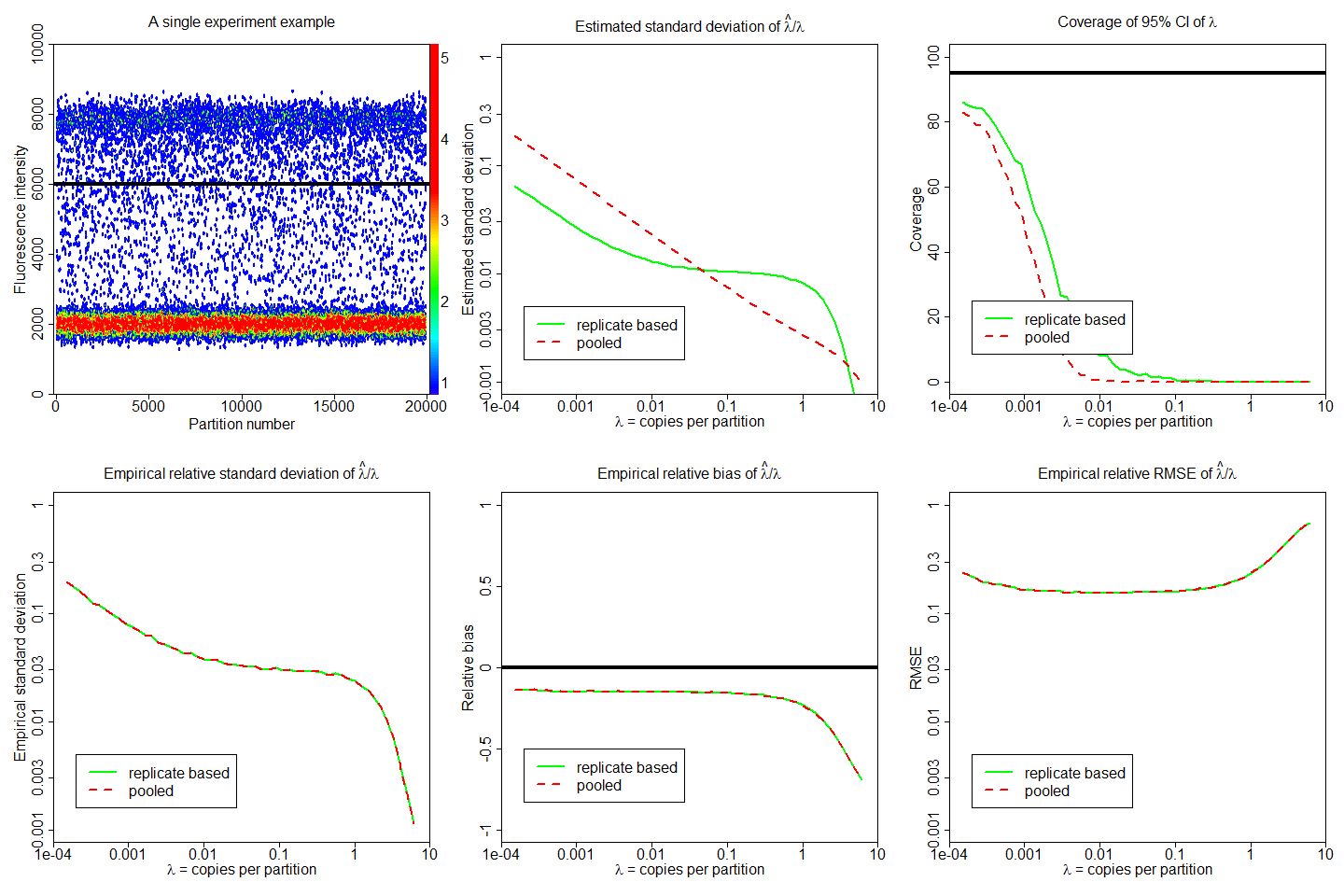

Supplement: Supplementary file 4 — Additional file 4: Interactive tool. In this mini-website, we provide an interactive tool to study the influence of specific sources of variation on the performance of the concentration estimators. This can serve as a guide when designing an experiment. All results are relative to the true concentration and based on 1000 simulations with 8 technical replicates. (ZIP 17 MB) [file 12859_2014_6687_MOESM4_ESM.zip › Additional file 4/RES/RES2533B.png]

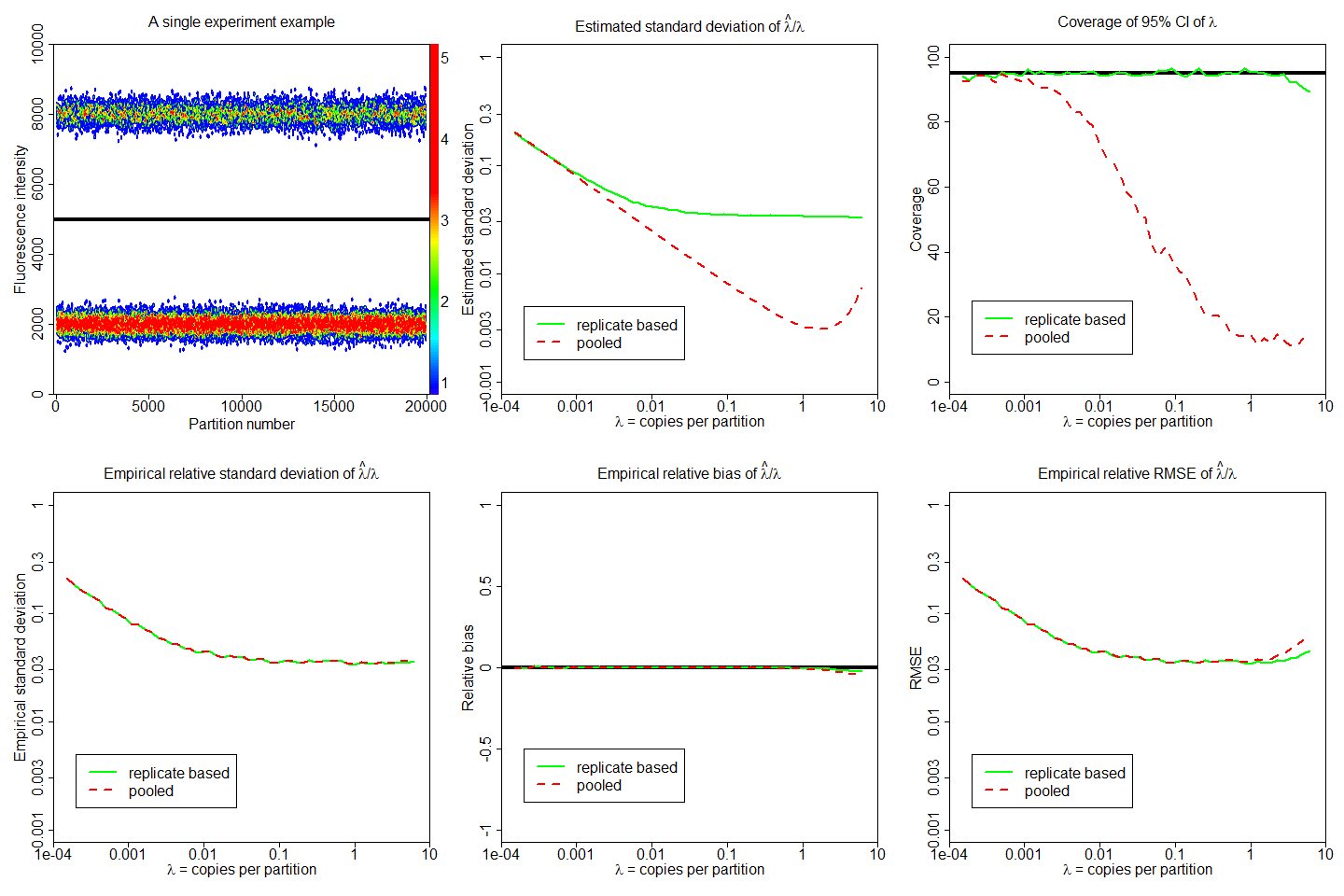

Supplement: Supplementary file 4 — Additional file 4: Interactive tool. In this mini-website, we provide an interactive tool to study the influence of specific sources of variation on the performance of the concentration estimators. This can serve as a guide when designing an experiment. All results are relative to the true concentration and based on 1000 simulations with 8 technical replicates. (ZIP 17 MB) [file 12859_2014_6687_MOESM4_ESM.zip › Additional file 4/RES/RES2541B.png]
