# Supplementary figures and images for: Impact of variance components on reliability of absolute quantification using digital PCR (part 1 of 2)
Source: BMC Bioinformatics. 2014 Aug 22;15(1):283. doi: 10.1186/1471-2105-15-283 (PMC4261249; doi:10.1186/1471-2105-15-283)

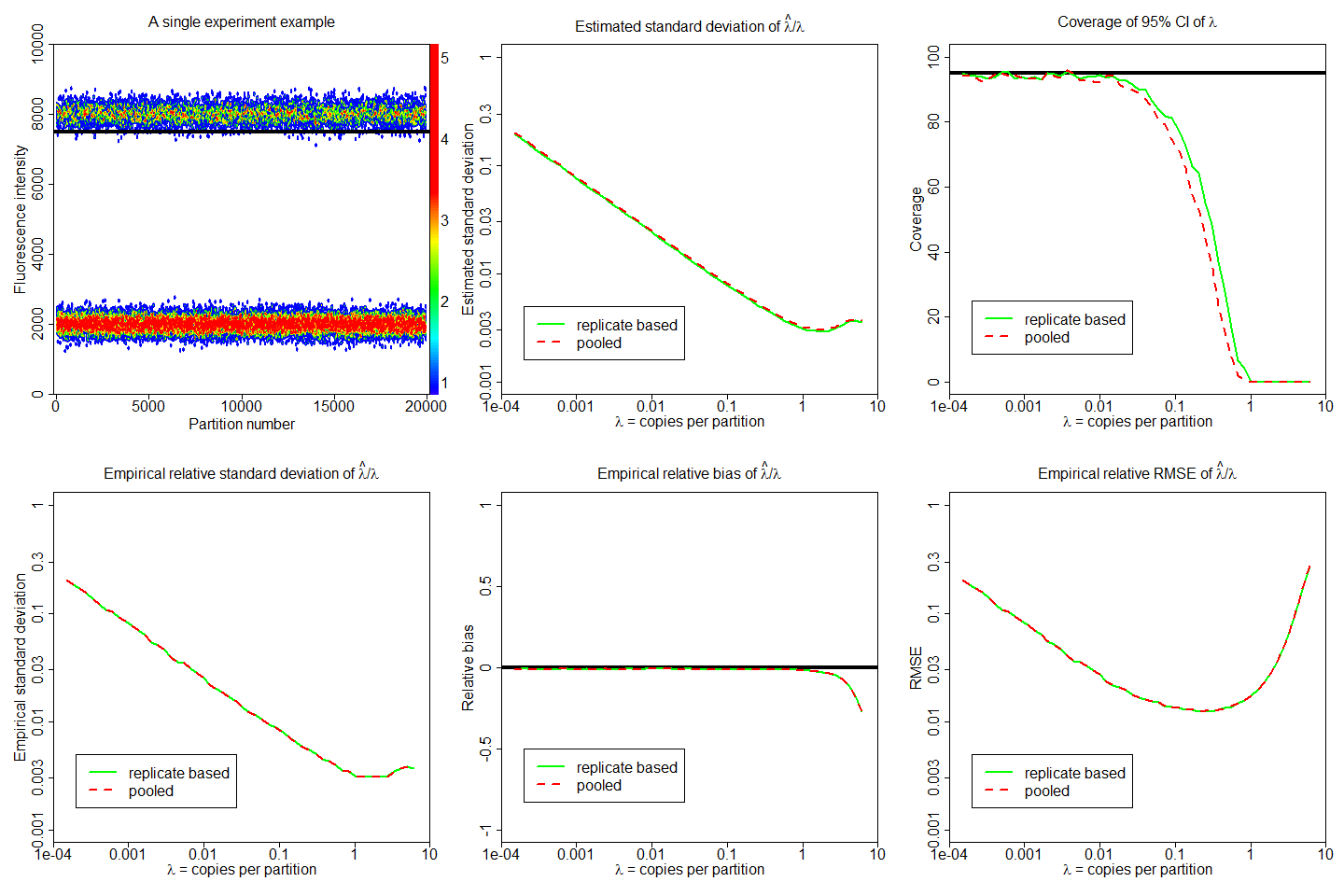

Supplement: Supplementary file 4 — Additional file 4: Interactive tool. In this mini-website, we provide an interactive tool to study the influence of specific sources of variation on the performance of the concentration estimators. This can serve as a guide when designing an experiment. All results are relative to the true concentration and based on 1000 simulations with 8 technical replicates. (ZIP 17 MB) [file 12859_2014_6687_MOESM4_ESM.zip › Additional file 4/RES/RES1111B.png]

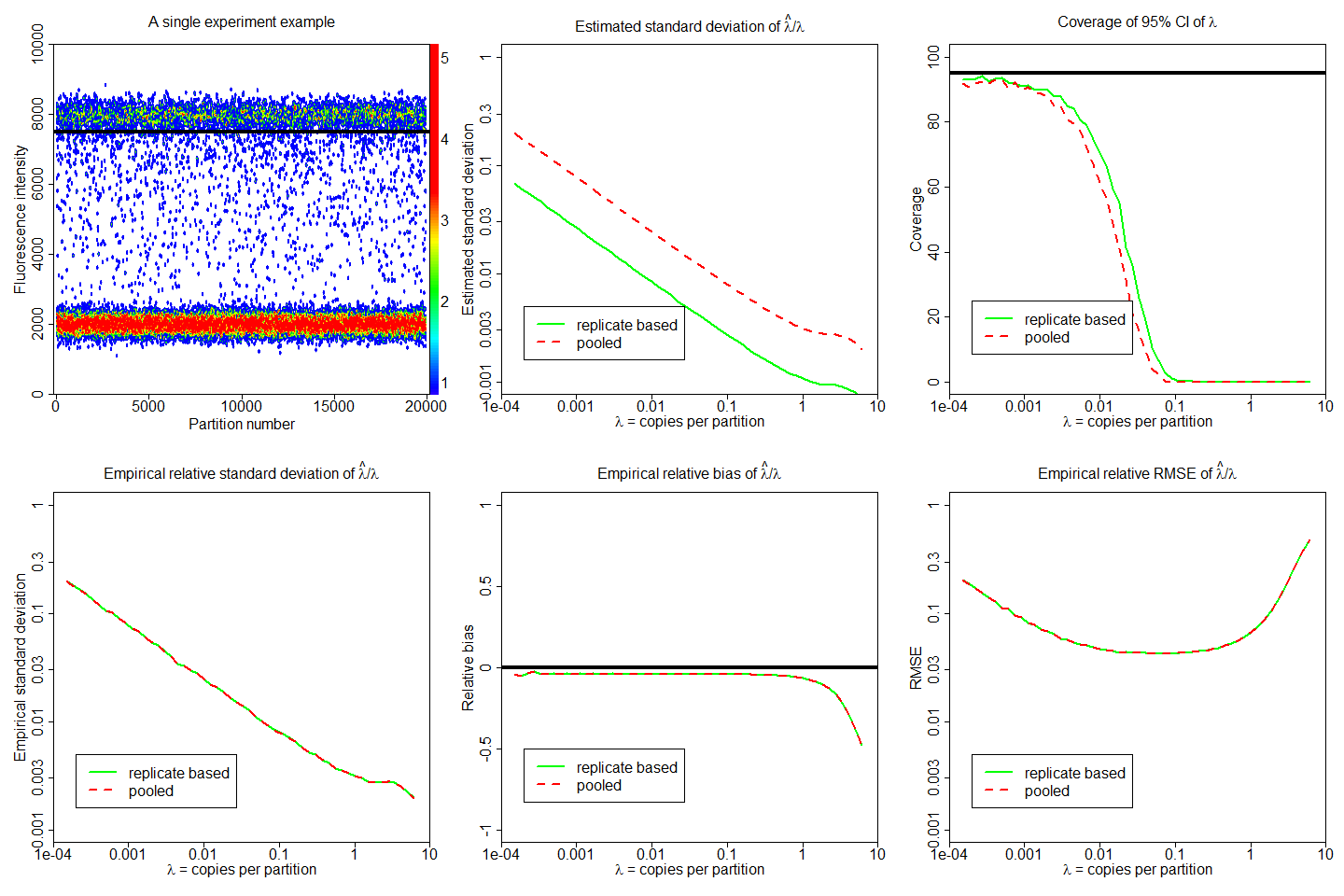

Supplement: Supplementary file 4 — Additional file 4: Interactive tool. In this mini-website, we provide an interactive tool to study the influence of specific sources of variation on the performance of the concentration estimators. This can serve as a guide when designing an experiment. All results are relative to the true concentration and based on 1000 simulations with 8 technical replicates. (ZIP 17 MB) [file 12859_2014_6687_MOESM4_ESM.zip › Additional file 4/RES/RES1112B.png]

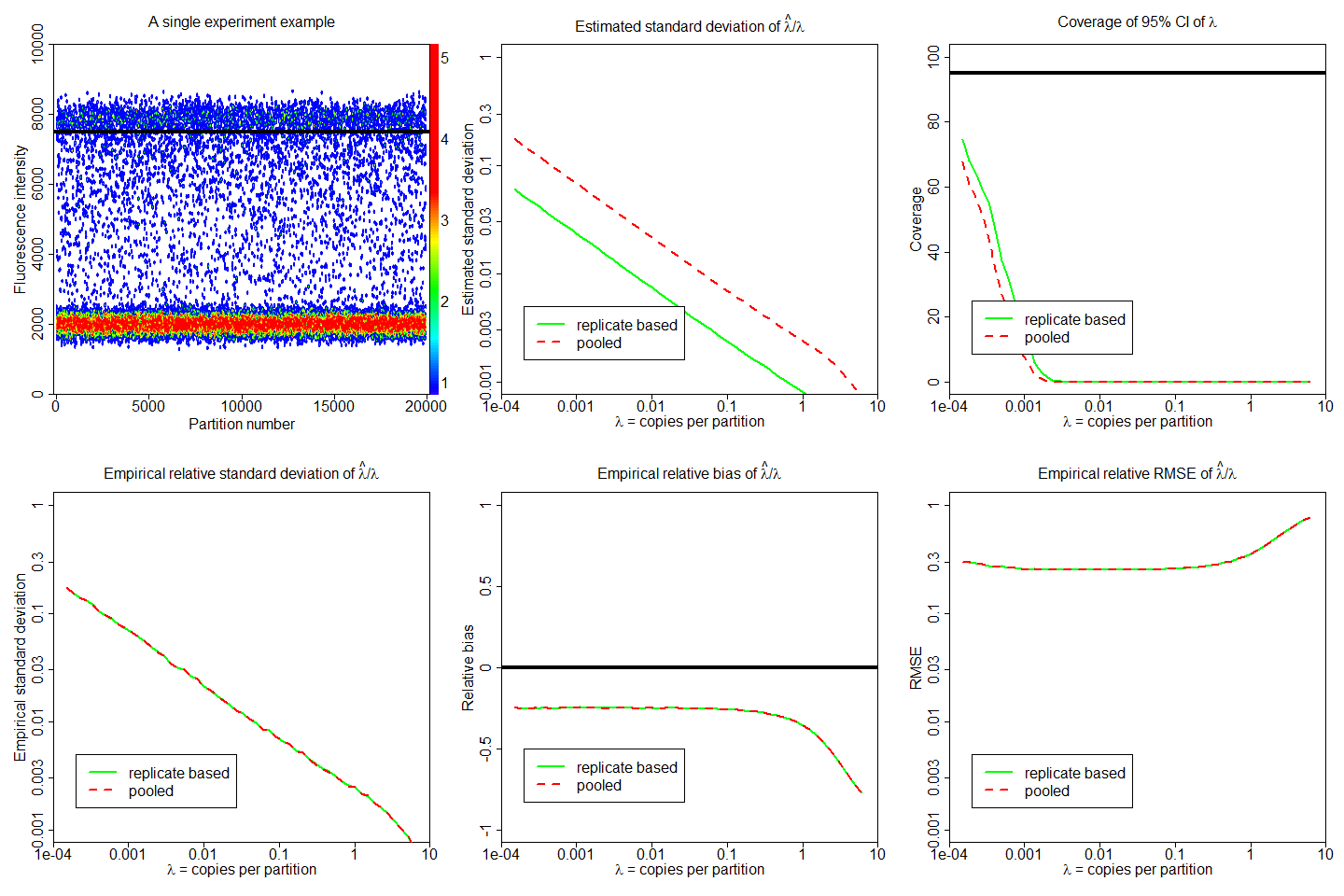

Supplement: Supplementary file 4 — Additional file 4: Interactive tool. In this mini-website, we provide an interactive tool to study the influence of specific sources of variation on the performance of the concentration estimators. This can serve as a guide when designing an experiment. All results are relative to the true concentration and based on 1000 simulations with 8 technical replicates. (ZIP 17 MB) [file 12859_2014_6687_MOESM4_ESM.zip › Additional file 4/RES/RES1113B.png]

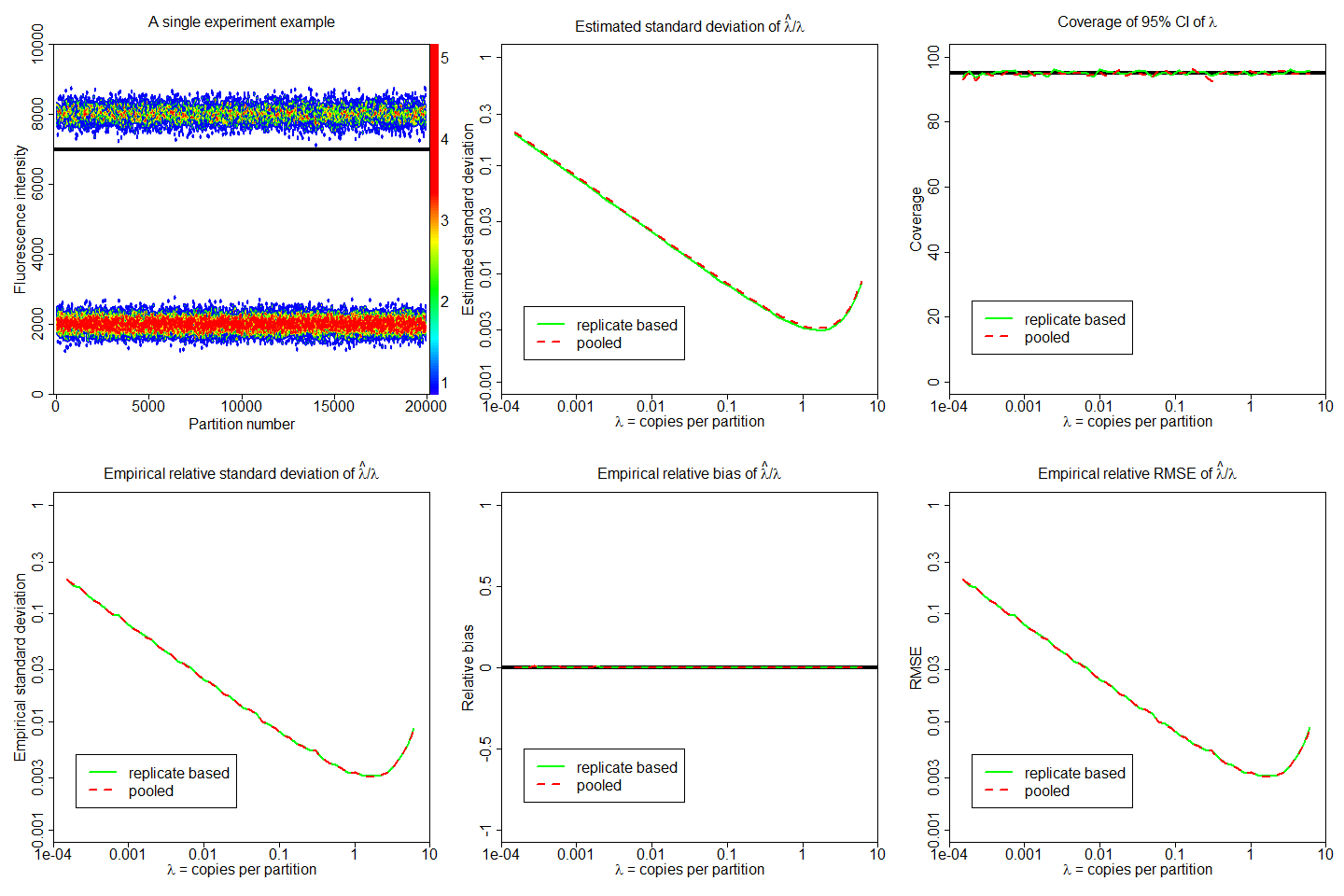

Supplement: Supplementary file 4 — Additional file 4: Interactive tool. In this mini-website, we provide an interactive tool to study the influence of specific sources of variation on the performance of the concentration estimators. This can serve as a guide when designing an experiment. All results are relative to the true concentration and based on 1000 simulations with 8 technical replicates. (ZIP 17 MB) [file 12859_2014_6687_MOESM4_ESM.zip › Additional file 4/RES/RES1121B.png]

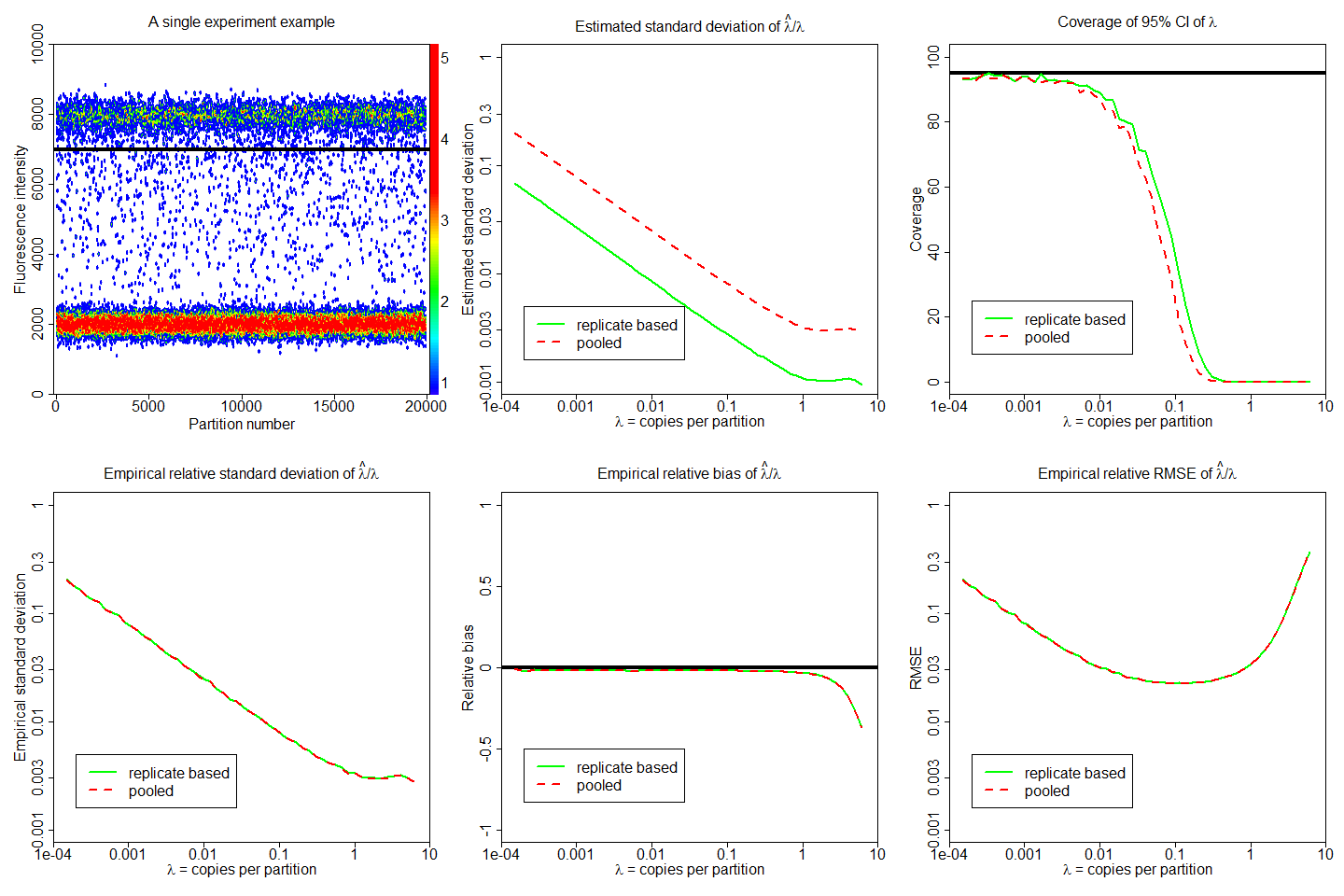

Supplement: Supplementary file 4 — Additional file 4: Interactive tool. In this mini-website, we provide an interactive tool to study the influence of specific sources of variation on the performance of the concentration estimators. This can serve as a guide when designing an experiment. All results are relative to the true concentration and based on 1000 simulations with 8 technical replicates. (ZIP 17 MB) [file 12859_2014_6687_MOESM4_ESM.zip › Additional file 4/RES/RES1122B.png]

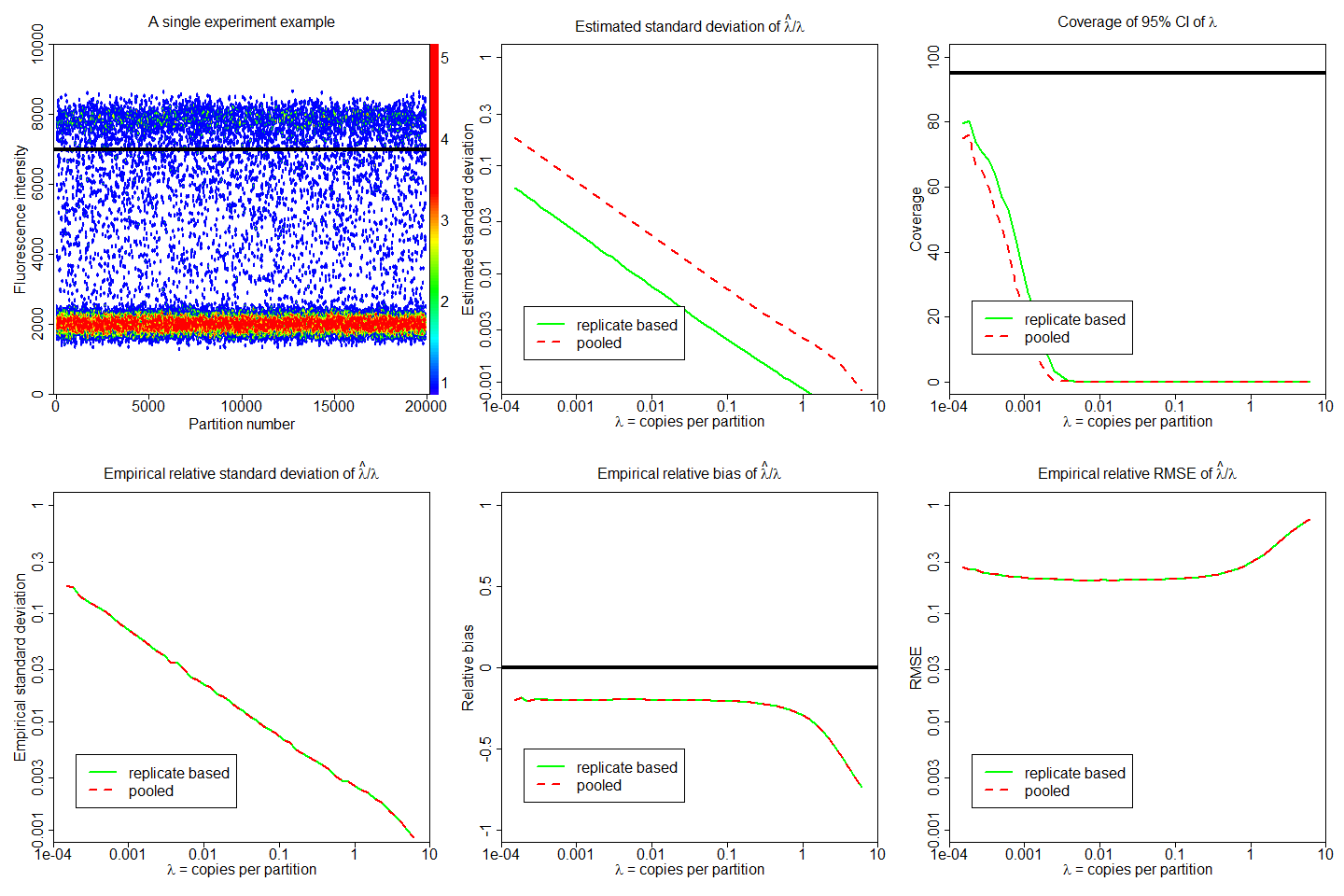

Supplement: Supplementary file 4 — Additional file 4: Interactive tool. In this mini-website, we provide an interactive tool to study the influence of specific sources of variation on the performance of the concentration estimators. This can serve as a guide when designing an experiment. All results are relative to the true concentration and based on 1000 simulations with 8 technical replicates. (ZIP 17 MB) [file 12859_2014_6687_MOESM4_ESM.zip › Additional file 4/RES/RES1123B.png]

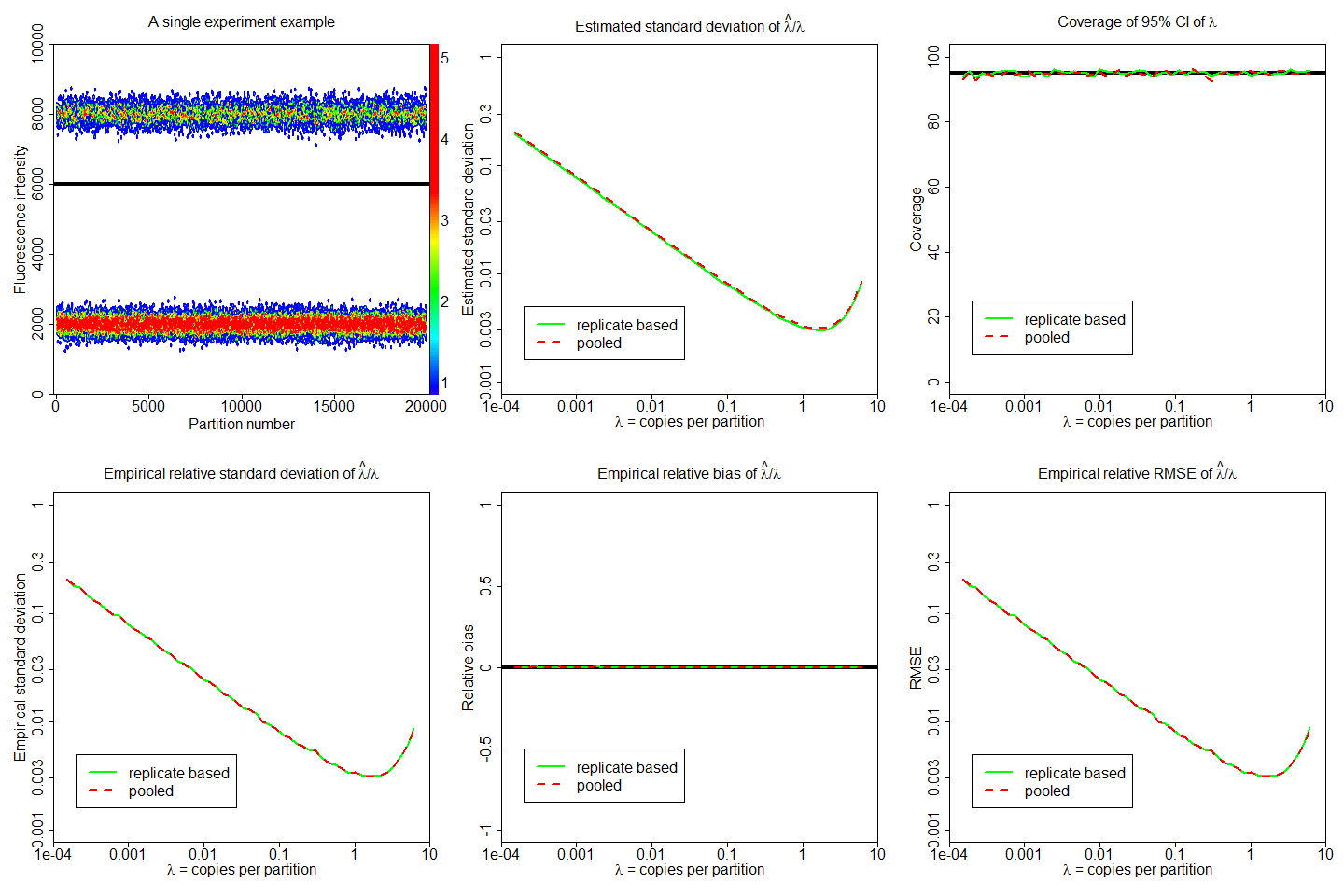

Supplement: Supplementary file 4 — Additional file 4: Interactive tool. In this mini-website, we provide an interactive tool to study the influence of specific sources of variation on the performance of the concentration estimators. This can serve as a guide when designing an experiment. All results are relative to the true concentration and based on 1000 simulations with 8 technical replicates. (ZIP 17 MB) [file 12859_2014_6687_MOESM4_ESM.zip › Additional file 4/RES/RES1131B.png]

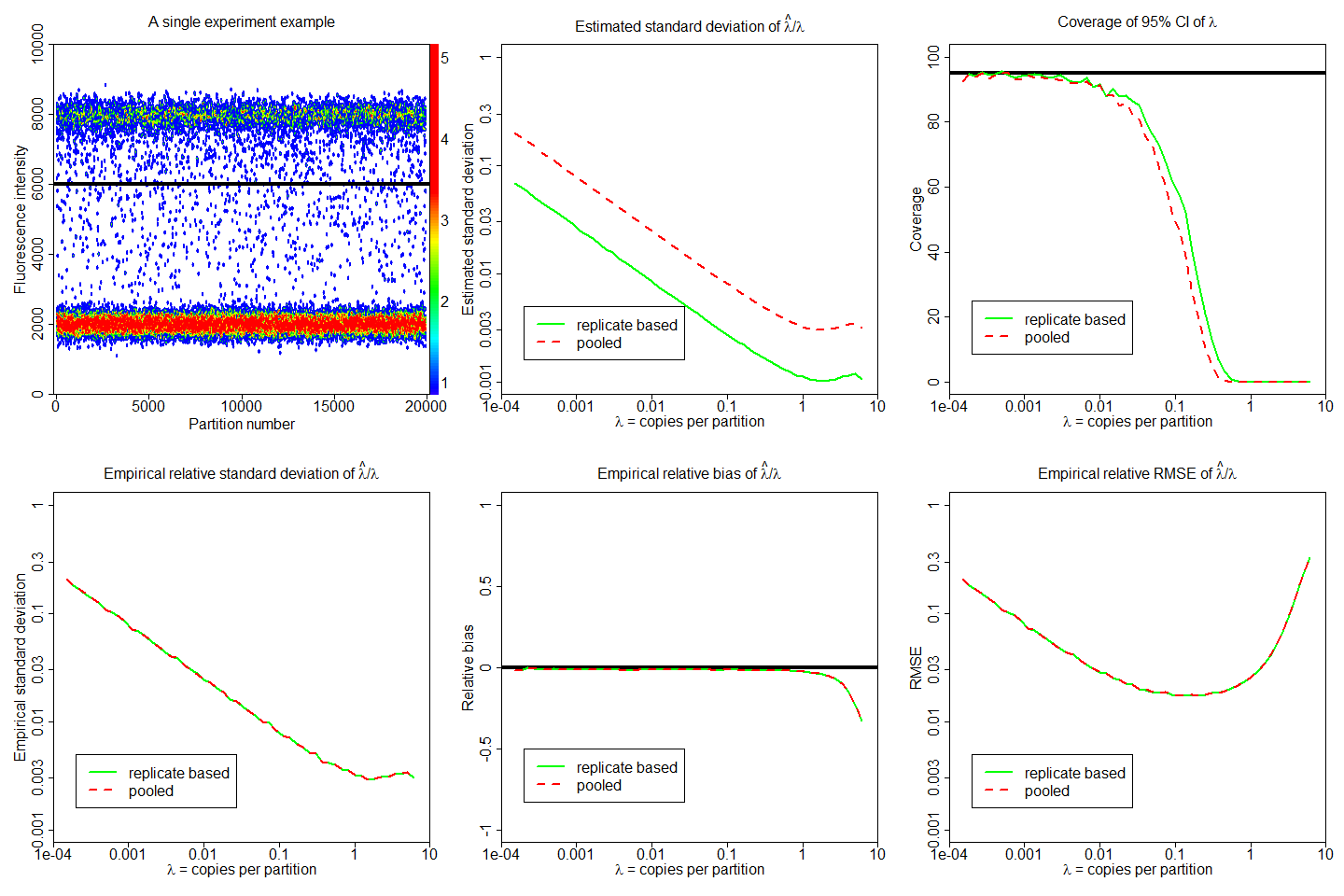

Supplement: Supplementary file 4 — Additional file 4: Interactive tool. In this mini-website, we provide an interactive tool to study the influence of specific sources of variation on the performance of the concentration estimators. This can serve as a guide when designing an experiment. All results are relative to the true concentration and based on 1000 simulations with 8 technical replicates. (ZIP 17 MB) [file 12859_2014_6687_MOESM4_ESM.zip › Additional file 4/RES/RES1132B.png]

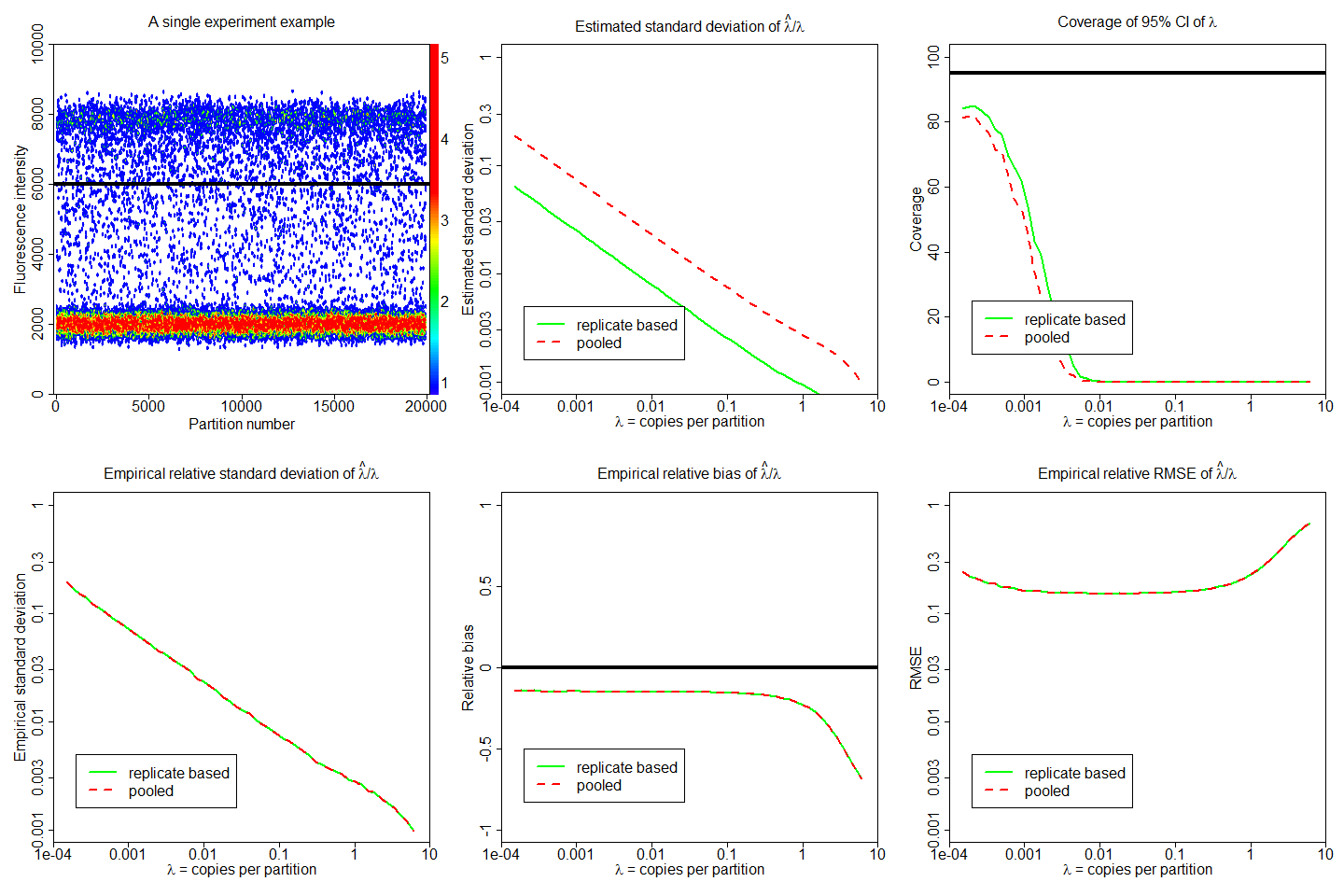

Supplement: Supplementary file 4 — Additional file 4: Interactive tool. In this mini-website, we provide an interactive tool to study the influence of specific sources of variation on the performance of the concentration estimators. This can serve as a guide when designing an experiment. All results are relative to the true concentration and based on 1000 simulations with 8 technical replicates. (ZIP 17 MB) [file 12859_2014_6687_MOESM4_ESM.zip › Additional file 4/RES/RES1133B.png]

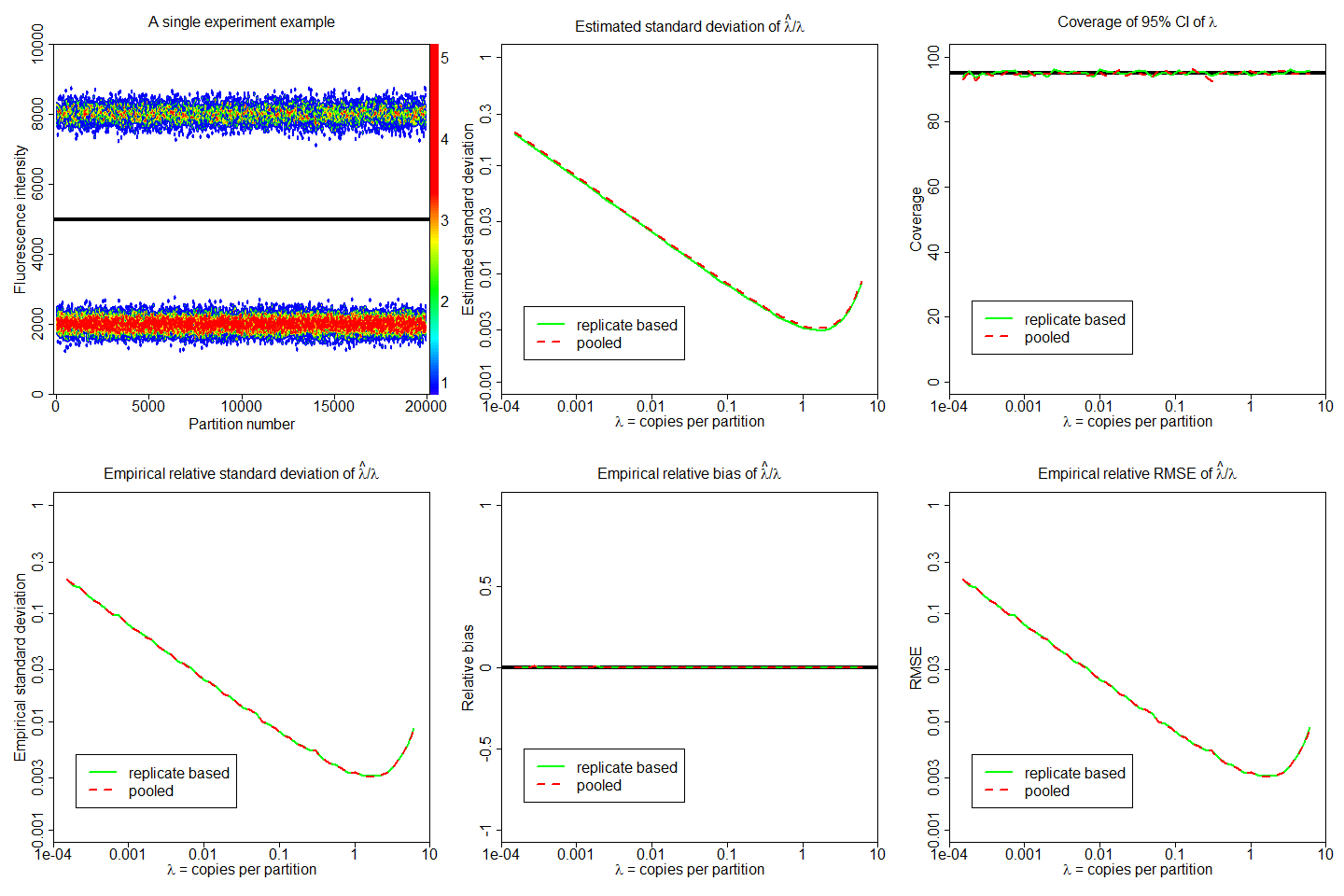

Supplement: Supplementary file 4 — Additional file 4: Interactive tool. In this mini-website, we provide an interactive tool to study the influence of specific sources of variation on the performance of the concentration estimators. This can serve as a guide when designing an experiment. All results are relative to the true concentration and based on 1000 simulations with 8 technical replicates. (ZIP 17 MB) [file 12859_2014_6687_MOESM4_ESM.zip › Additional file 4/RES/RES1141B.png]

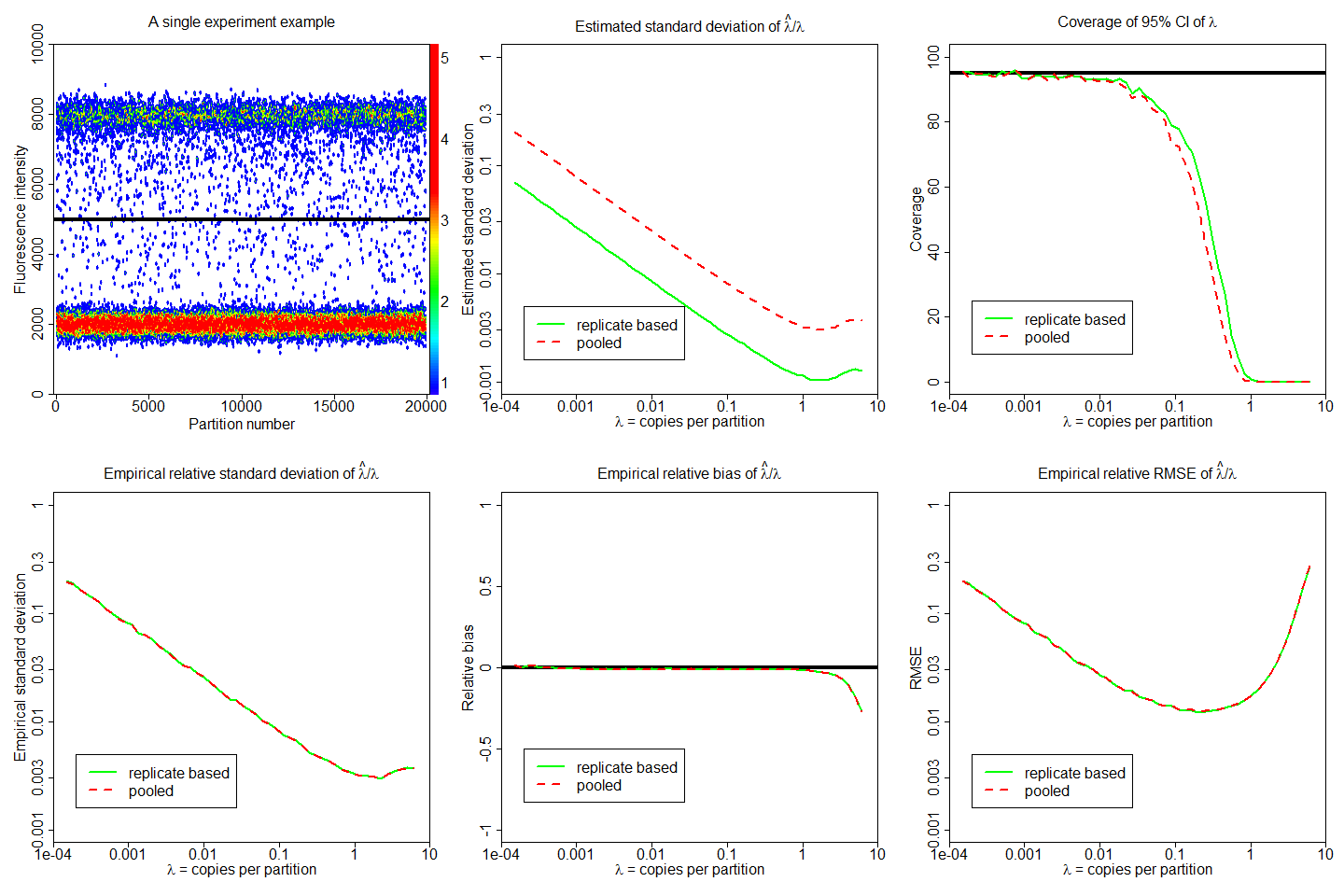

Supplement: Supplementary file 4 — Additional file 4: Interactive tool. In this mini-website, we provide an interactive tool to study the influence of specific sources of variation on the performance of the concentration estimators. This can serve as a guide when designing an experiment. All results are relative to the true concentration and based on 1000 simulations with 8 technical replicates. (ZIP 17 MB) [file 12859_2014_6687_MOESM4_ESM.zip › Additional file 4/RES/RES1142B.png]

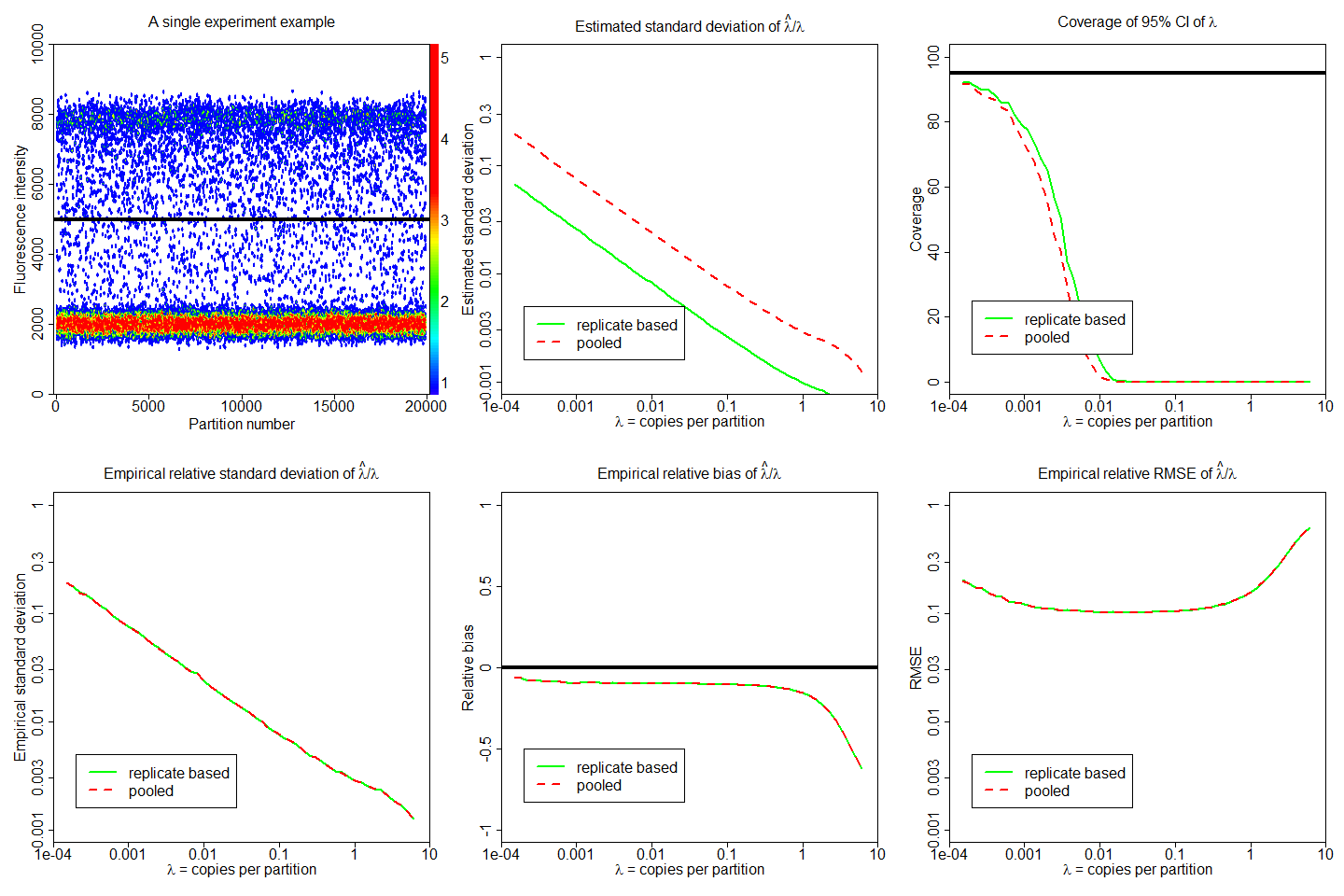

Supplement: Supplementary file 4 — Additional file 4: Interactive tool. In this mini-website, we provide an interactive tool to study the influence of specific sources of variation on the performance of the concentration estimators. This can serve as a guide when designing an experiment. All results are relative to the true concentration and based on 1000 simulations with 8 technical replicates. (ZIP 17 MB) [file 12859_2014_6687_MOESM4_ESM.zip › Additional file 4/RES/RES1143B.png]

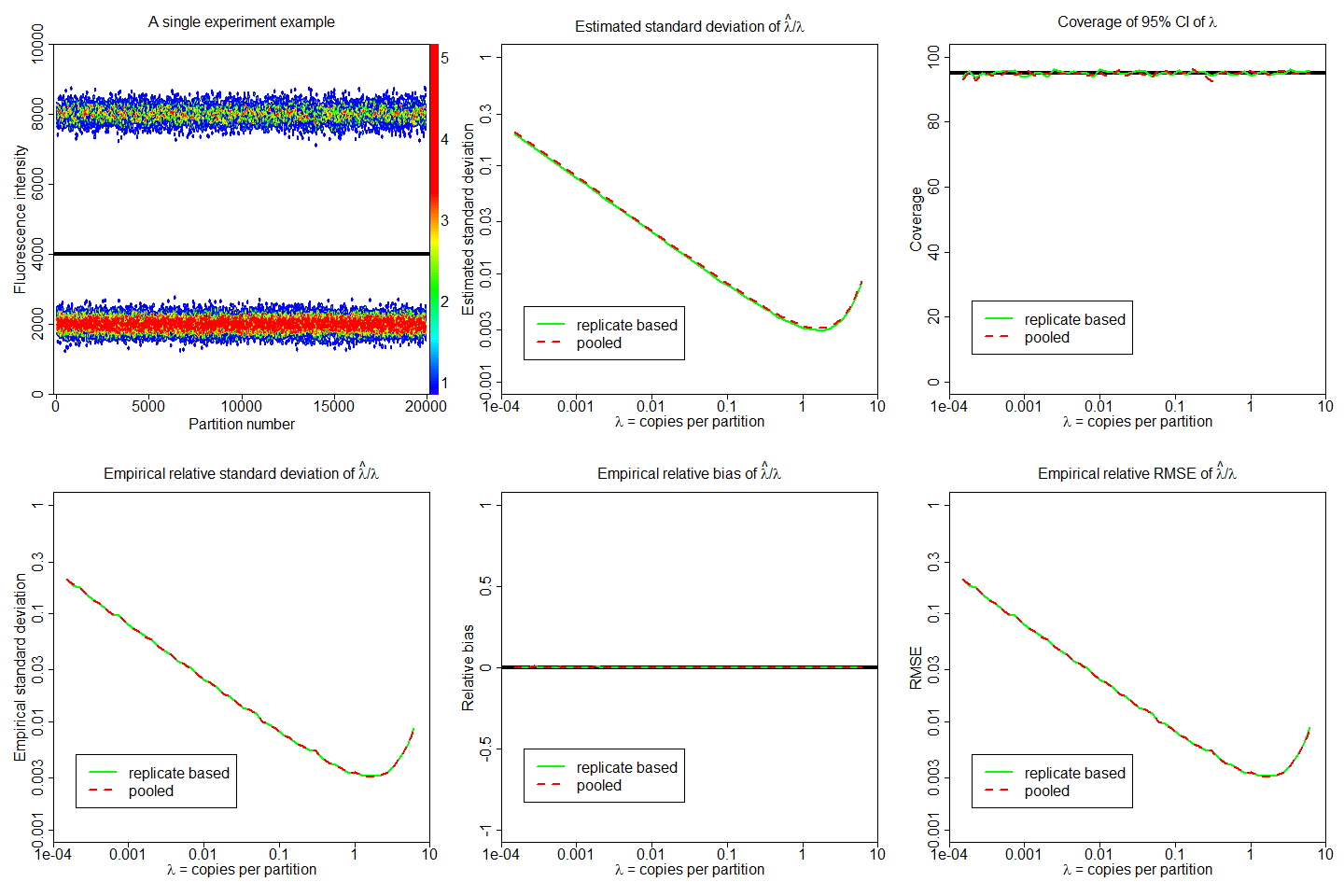

Supplement: Supplementary file 4 — Additional file 4: Interactive tool. In this mini-website, we provide an interactive tool to study the influence of specific sources of variation on the performance of the concentration estimators. This can serve as a guide when designing an experiment. All results are relative to the true concentration and based on 1000 simulations with 8 technical replicates. (ZIP 17 MB) [file 12859_2014_6687_MOESM4_ESM.zip › Additional file 4/RES/RES1151B.png]

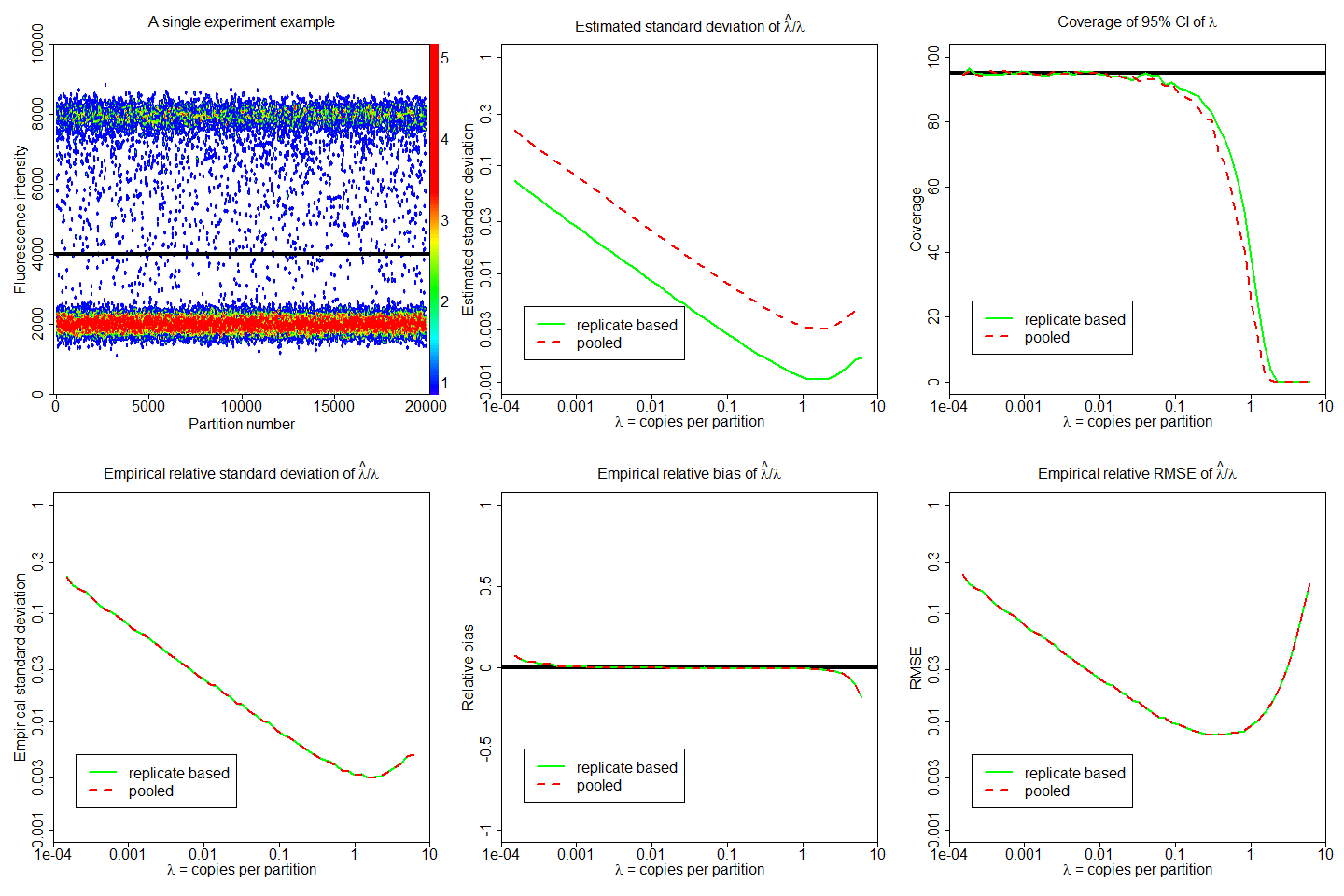

Supplement: Supplementary file 4 — Additional file 4: Interactive tool. In this mini-website, we provide an interactive tool to study the influence of specific sources of variation on the performance of the concentration estimators. This can serve as a guide when designing an experiment. All results are relative to the true concentration and based on 1000 simulations with 8 technical replicates. (ZIP 17 MB) [file 12859_2014_6687_MOESM4_ESM.zip › Additional file 4/RES/RES1152B.png]

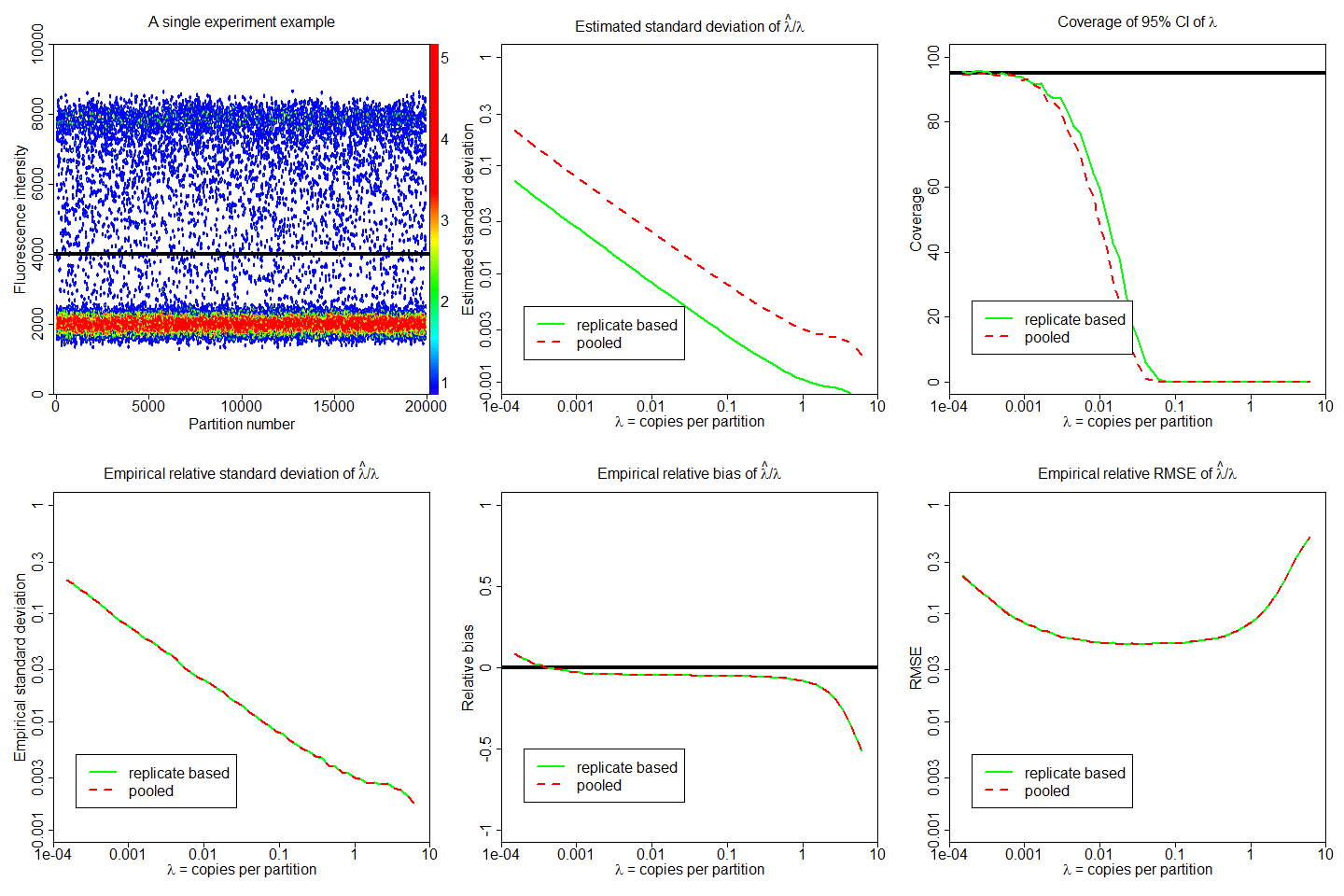

Supplement: Supplementary file 4 — Additional file 4: Interactive tool. In this mini-website, we provide an interactive tool to study the influence of specific sources of variation on the performance of the concentration estimators. This can serve as a guide when designing an experiment. All results are relative to the true concentration and based on 1000 simulations with 8 technical replicates. (ZIP 17 MB) [file 12859_2014_6687_MOESM4_ESM.zip › Additional file 4/RES/RES1153B.png]

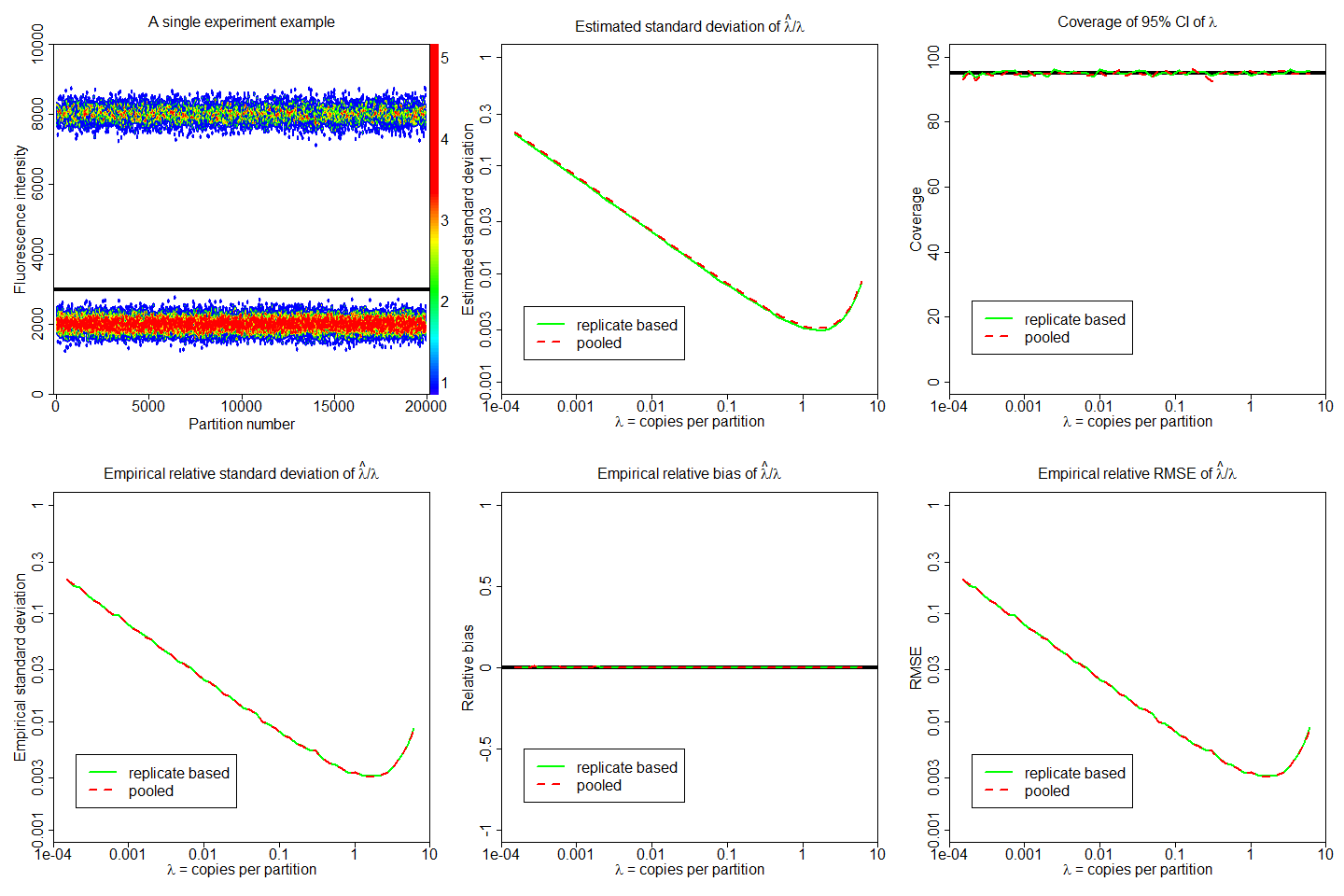

Supplement: Supplementary file 4 — Additional file 4: Interactive tool. In this mini-website, we provide an interactive tool to study the influence of specific sources of variation on the performance of the concentration estimators. This can serve as a guide when designing an experiment. All results are relative to the true concentration and based on 1000 simulations with 8 technical replicates. (ZIP 17 MB) [file 12859_2014_6687_MOESM4_ESM.zip › Additional file 4/RES/RES1161B.png]

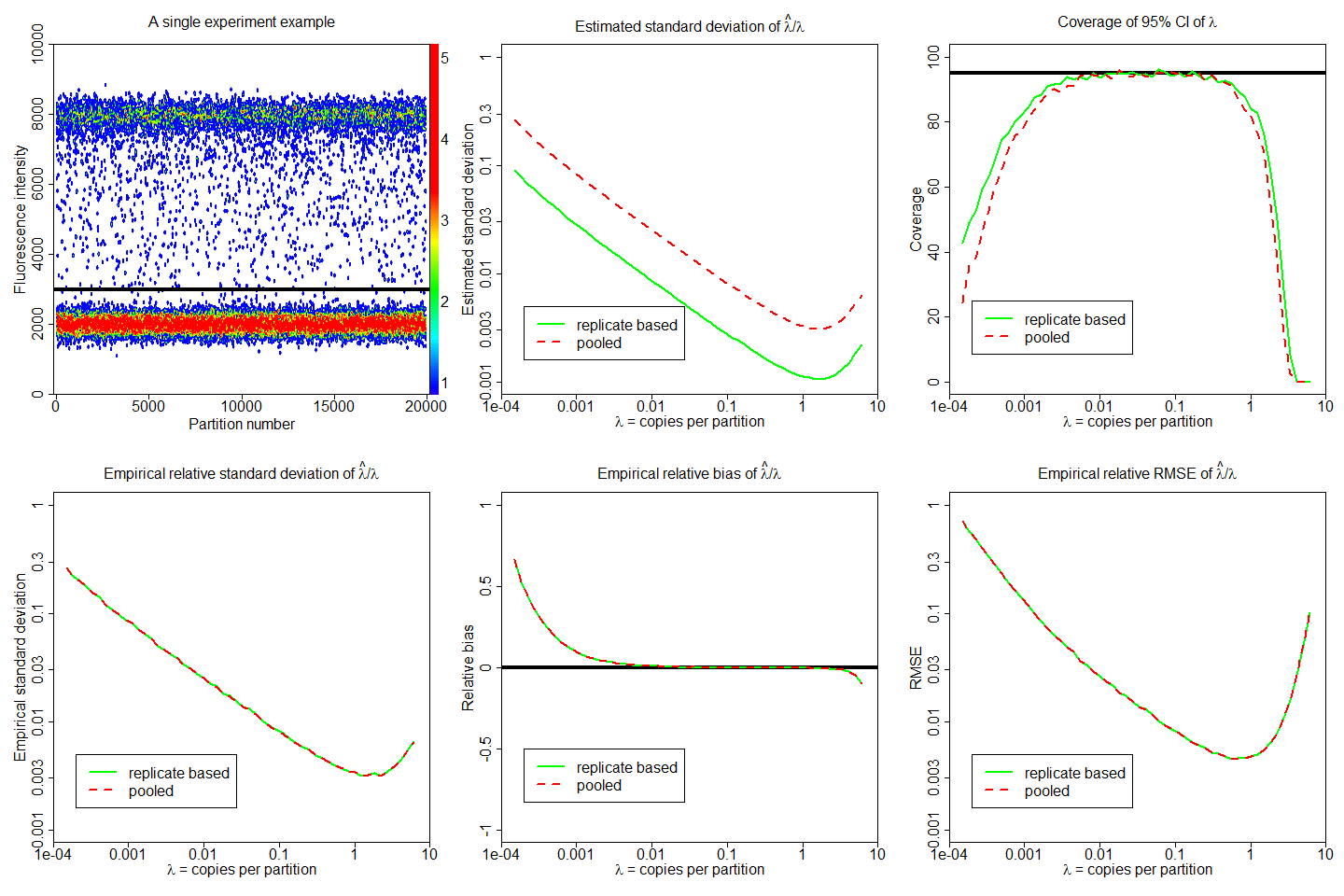

Supplement: Supplementary file 4 — Additional file 4: Interactive tool. In this mini-website, we provide an interactive tool to study the influence of specific sources of variation on the performance of the concentration estimators. This can serve as a guide when designing an experiment. All results are relative to the true concentration and based on 1000 simulations with 8 technical replicates. (ZIP 17 MB) [file 12859_2014_6687_MOESM4_ESM.zip › Additional file 4/RES/RES1162B.png]

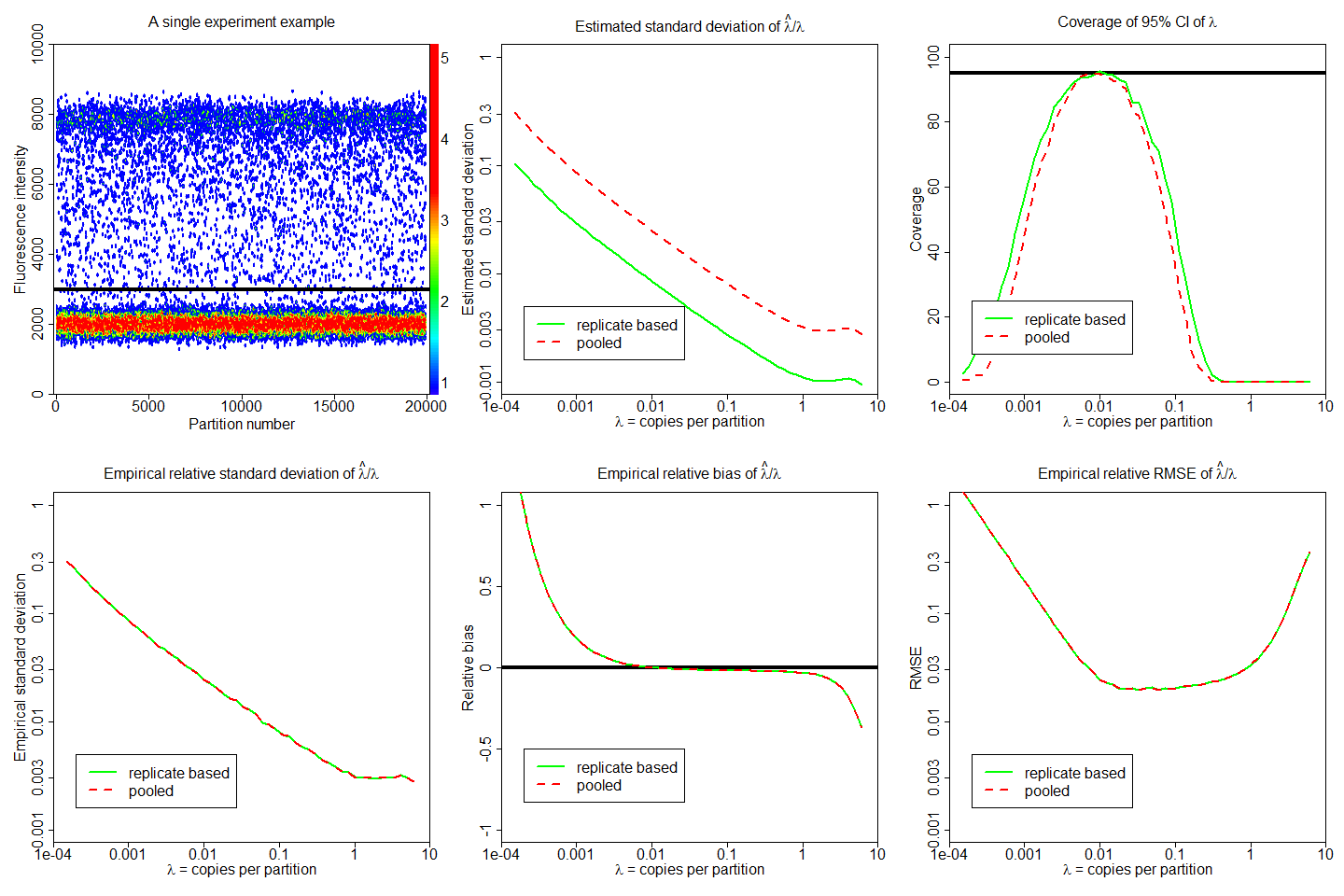

Supplement: Supplementary file 4 — Additional file 4: Interactive tool. In this mini-website, we provide an interactive tool to study the influence of specific sources of variation on the performance of the concentration estimators. This can serve as a guide when designing an experiment. All results are relative to the true concentration and based on 1000 simulations with 8 technical replicates. (ZIP 17 MB) [file 12859_2014_6687_MOESM4_ESM.zip › Additional file 4/RES/RES1163B.png]

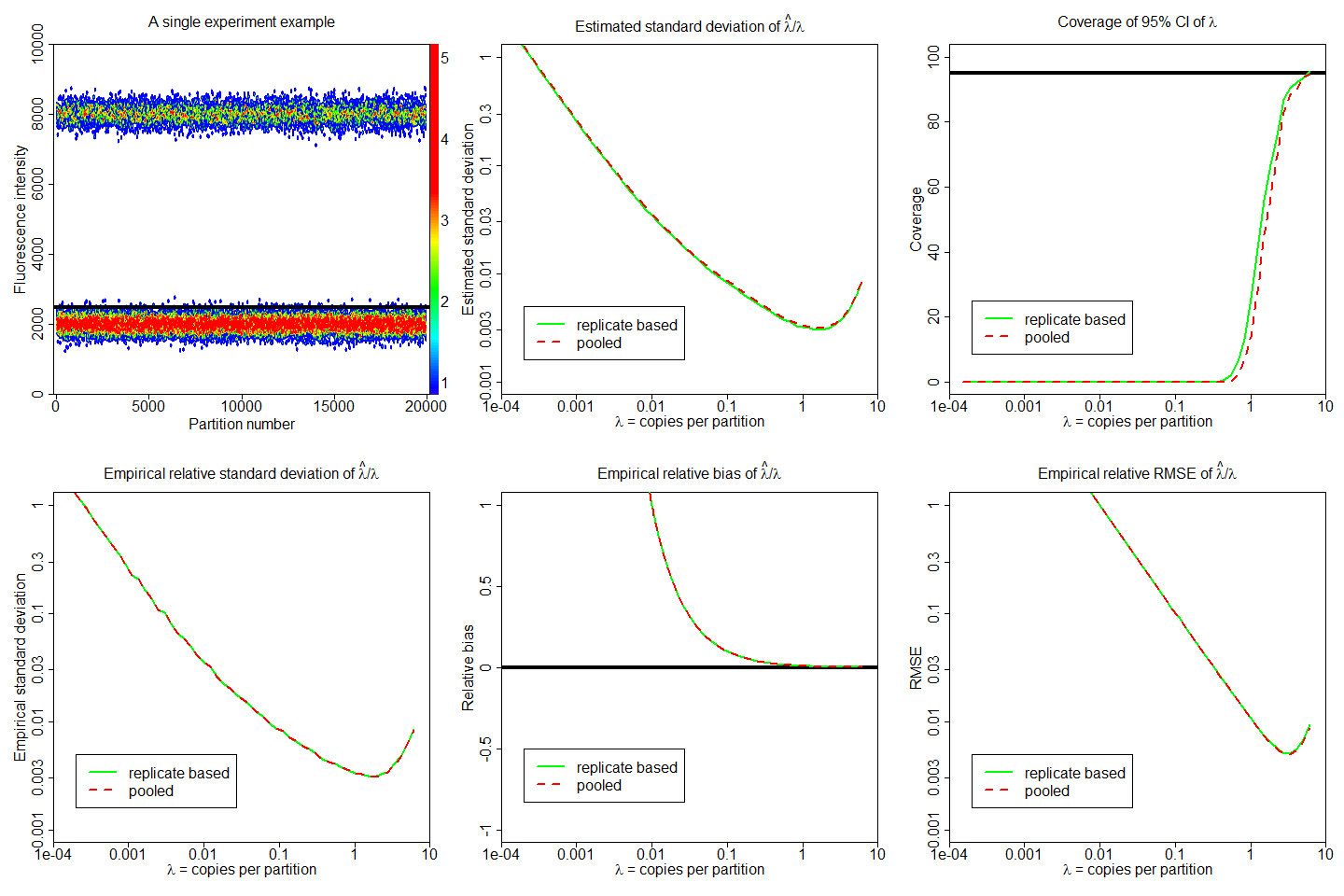

Supplement: Supplementary file 4 — Additional file 4: Interactive tool. In this mini-website, we provide an interactive tool to study the influence of specific sources of variation on the performance of the concentration estimators. This can serve as a guide when designing an experiment. All results are relative to the true concentration and based on 1000 simulations with 8 technical replicates. (ZIP 17 MB) [file 12859_2014_6687_MOESM4_ESM.zip › Additional file 4/RES/RES1171B.png]

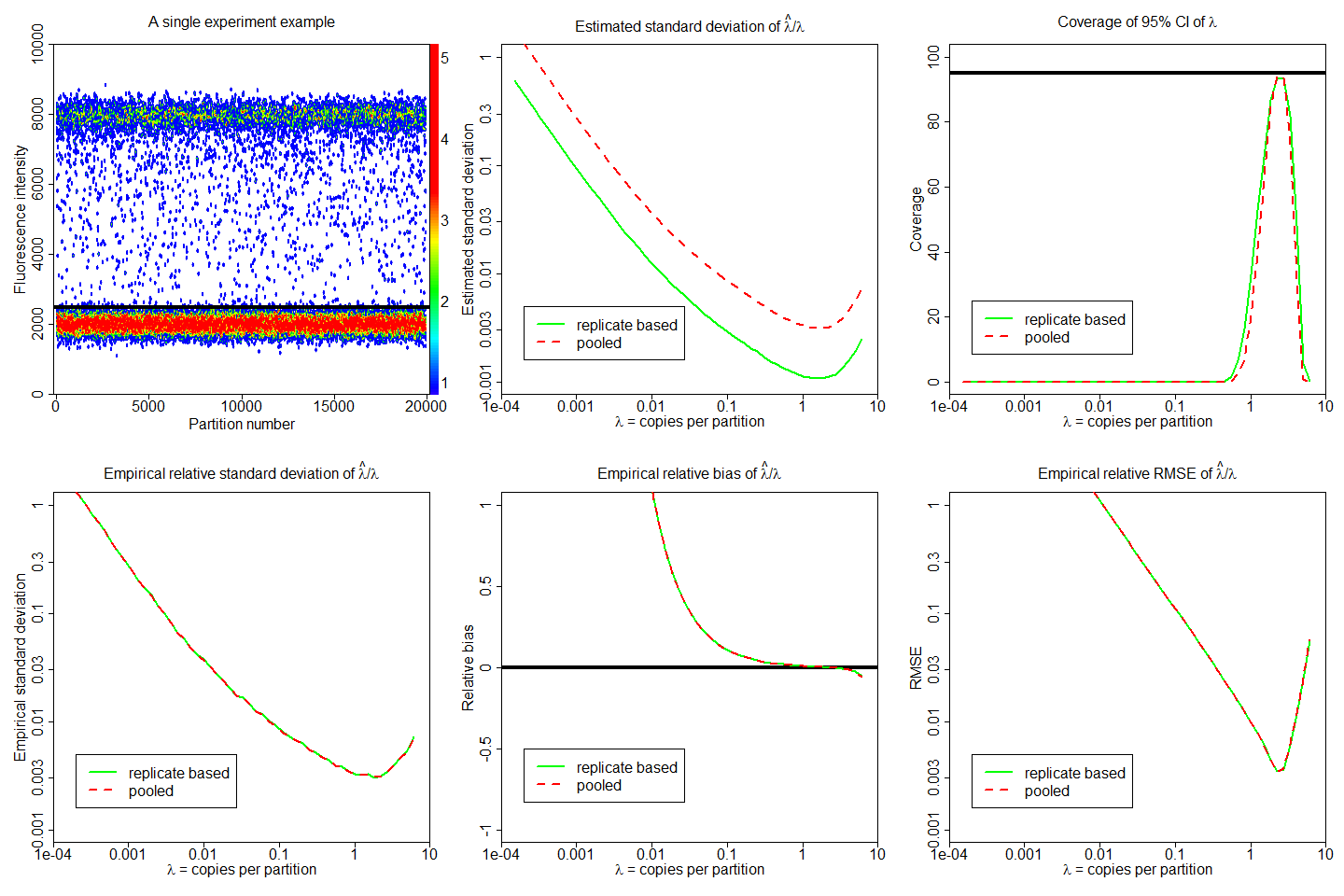

Supplement: Supplementary file 4 — Additional file 4: Interactive tool. In this mini-website, we provide an interactive tool to study the influence of specific sources of variation on the performance of the concentration estimators. This can serve as a guide when designing an experiment. All results are relative to the true concentration and based on 1000 simulations with 8 technical replicates. (ZIP 17 MB) [file 12859_2014_6687_MOESM4_ESM.zip › Additional file 4/RES/RES1172B.png]

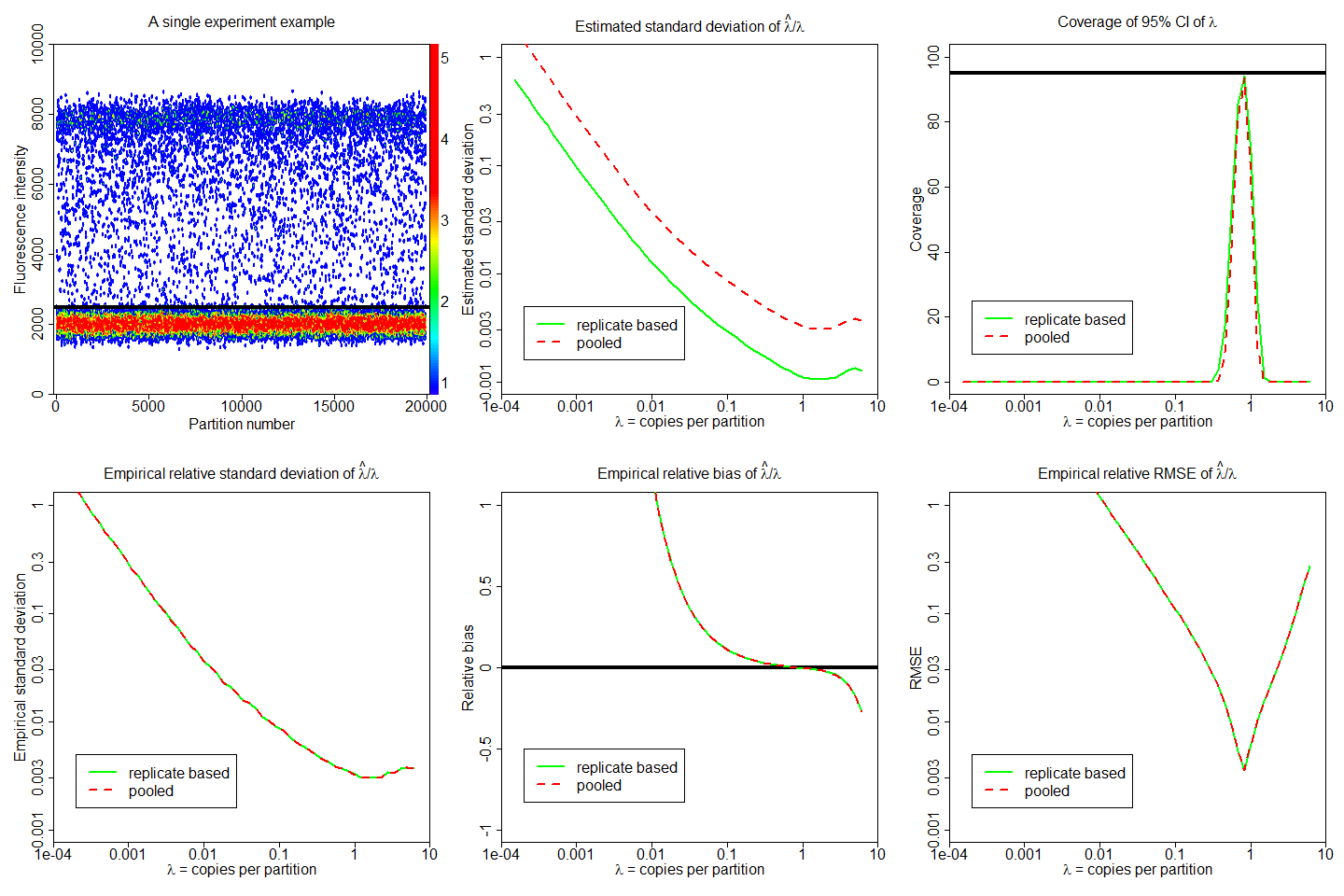

Supplement: Supplementary file 4 — Additional file 4: Interactive tool. In this mini-website, we provide an interactive tool to study the influence of specific sources of variation on the performance of the concentration estimators. This can serve as a guide when designing an experiment. All results are relative to the true concentration and based on 1000 simulations with 8 technical replicates. (ZIP 17 MB) [file 12859_2014_6687_MOESM4_ESM.zip › Additional file 4/RES/RES1173B.png]

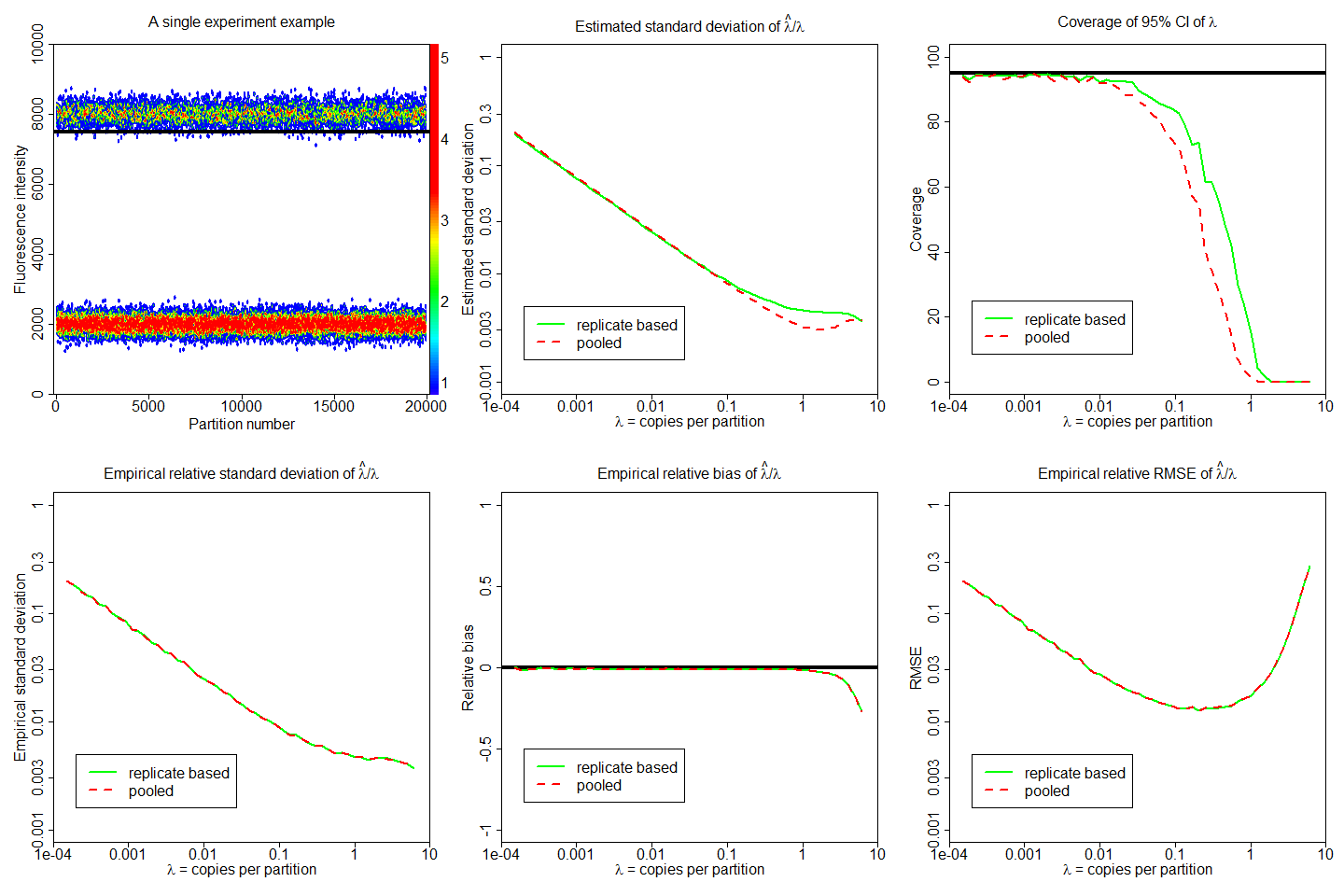

Supplement: Supplementary file 4 — Additional file 4: Interactive tool. In this mini-website, we provide an interactive tool to study the influence of specific sources of variation on the performance of the concentration estimators. This can serve as a guide when designing an experiment. All results are relative to the true concentration and based on 1000 simulations with 8 technical replicates. (ZIP 17 MB) [file 12859_2014_6687_MOESM4_ESM.zip › Additional file 4/RES/RES1211B.png]

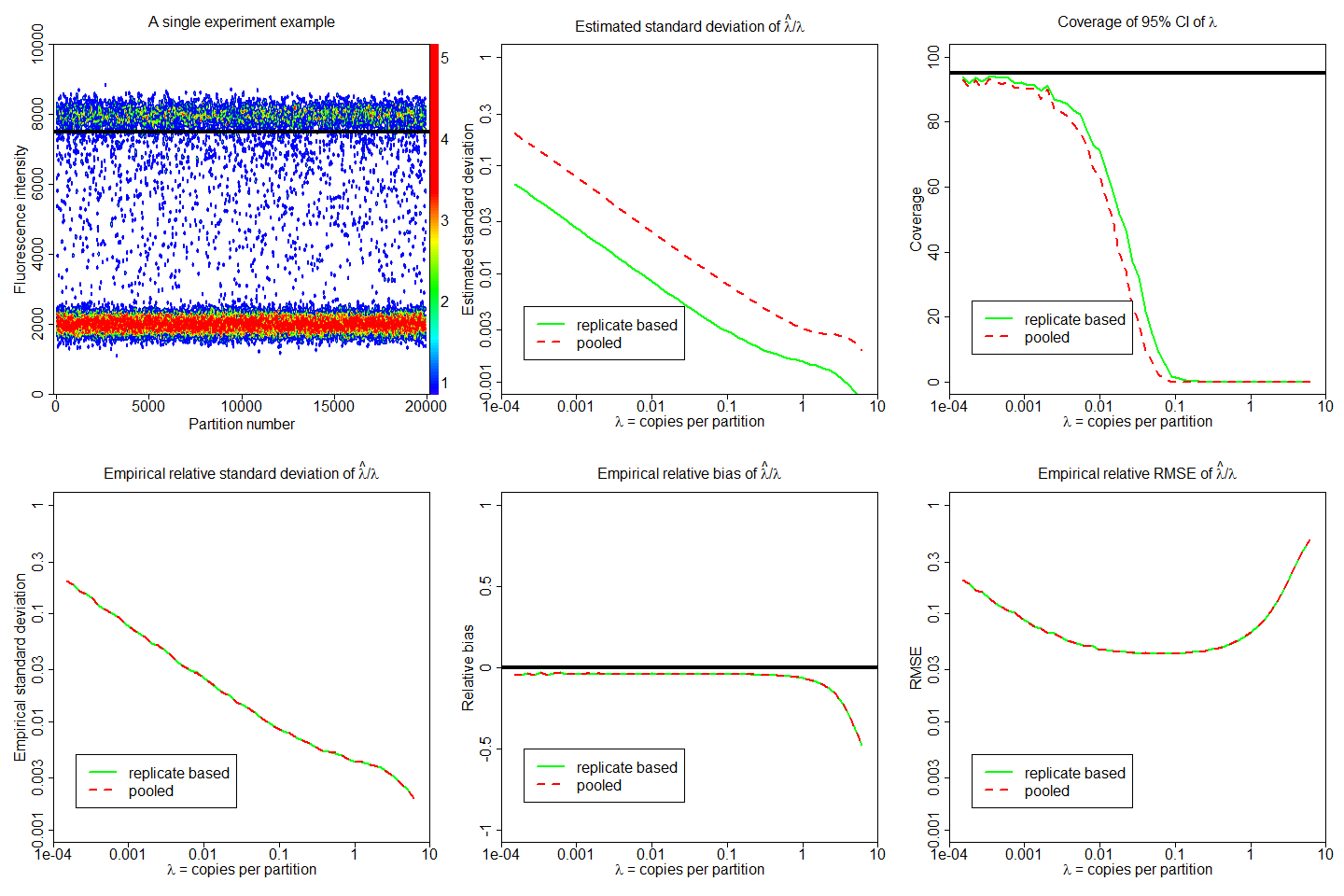

Supplement: Supplementary file 4 — Additional file 4: Interactive tool. In this mini-website, we provide an interactive tool to study the influence of specific sources of variation on the performance of the concentration estimators. This can serve as a guide when designing an experiment. All results are relative to the true concentration and based on 1000 simulations with 8 technical replicates. (ZIP 17 MB) [file 12859_2014_6687_MOESM4_ESM.zip › Additional file 4/RES/RES1212B.png]

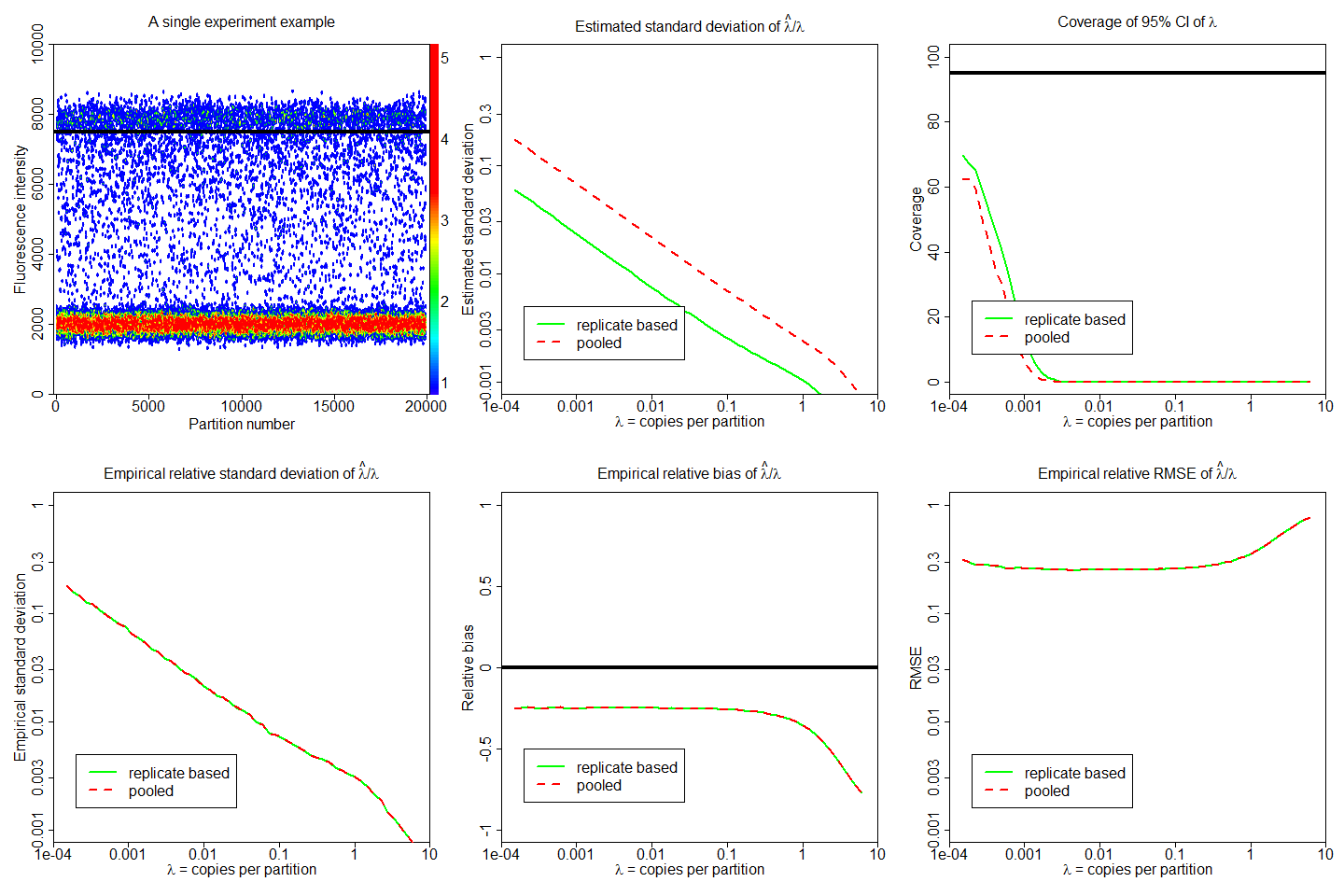

Supplement: Supplementary file 4 — Additional file 4: Interactive tool. In this mini-website, we provide an interactive tool to study the influence of specific sources of variation on the performance of the concentration estimators. This can serve as a guide when designing an experiment. All results are relative to the true concentration and based on 1000 simulations with 8 technical replicates. (ZIP 17 MB) [file 12859_2014_6687_MOESM4_ESM.zip › Additional file 4/RES/RES1213B.png]

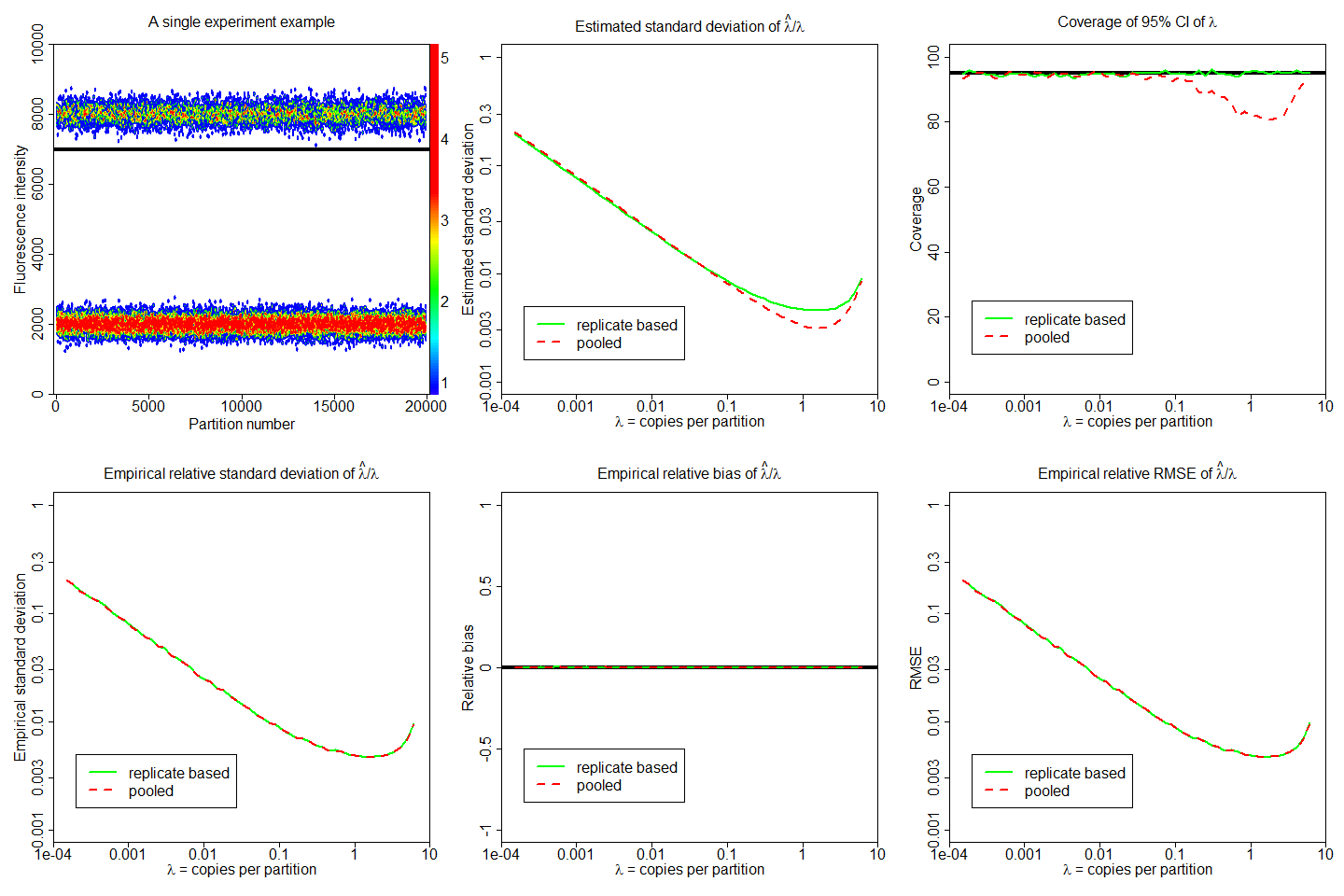

Supplement: Supplementary file 4 — Additional file 4: Interactive tool. In this mini-website, we provide an interactive tool to study the influence of specific sources of variation on the performance of the concentration estimators. This can serve as a guide when designing an experiment. All results are relative to the true concentration and based on 1000 simulations with 8 technical replicates. (ZIP 17 MB) [file 12859_2014_6687_MOESM4_ESM.zip › Additional file 4/RES/RES1221B.png]

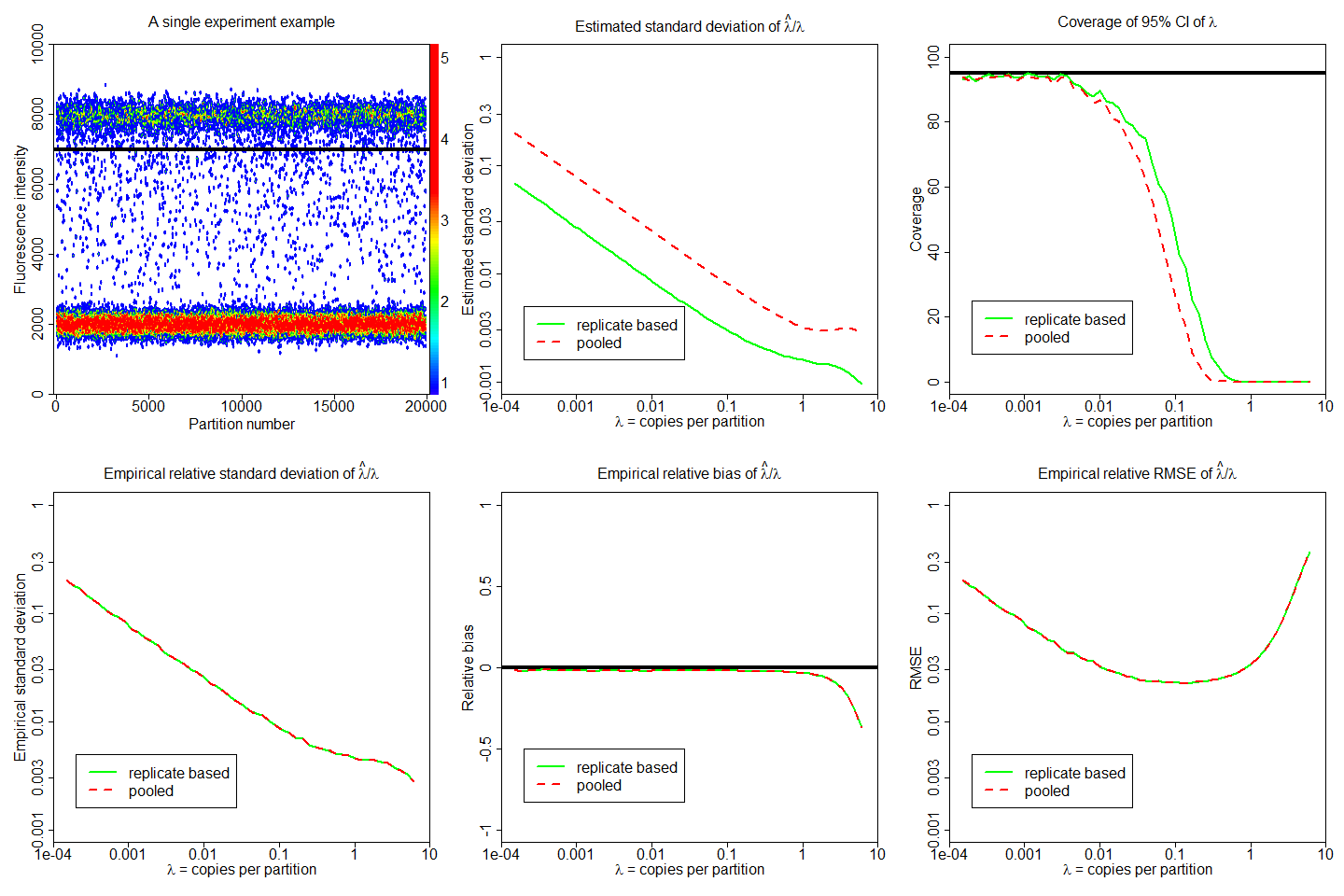

Supplement: Supplementary file 4 — Additional file 4: Interactive tool. In this mini-website, we provide an interactive tool to study the influence of specific sources of variation on the performance of the concentration estimators. This can serve as a guide when designing an experiment. All results are relative to the true concentration and based on 1000 simulations with 8 technical replicates. (ZIP 17 MB) [file 12859_2014_6687_MOESM4_ESM.zip › Additional file 4/RES/RES1222B.png]

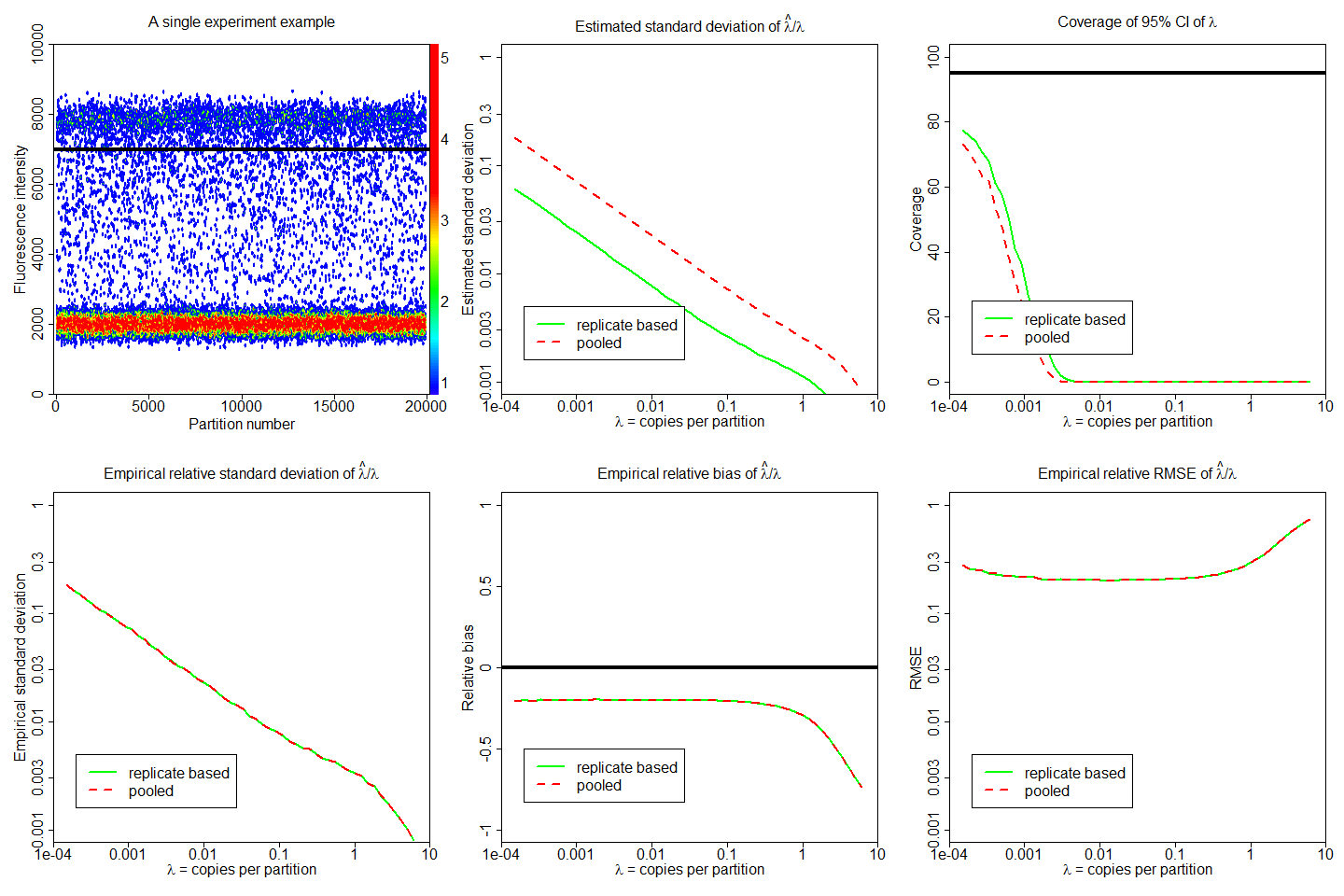

Supplement: Supplementary file 4 — Additional file 4: Interactive tool. In this mini-website, we provide an interactive tool to study the influence of specific sources of variation on the performance of the concentration estimators. This can serve as a guide when designing an experiment. All results are relative to the true concentration and based on 1000 simulations with 8 technical replicates. (ZIP 17 MB) [file 12859_2014_6687_MOESM4_ESM.zip › Additional file 4/RES/RES1223B.png]

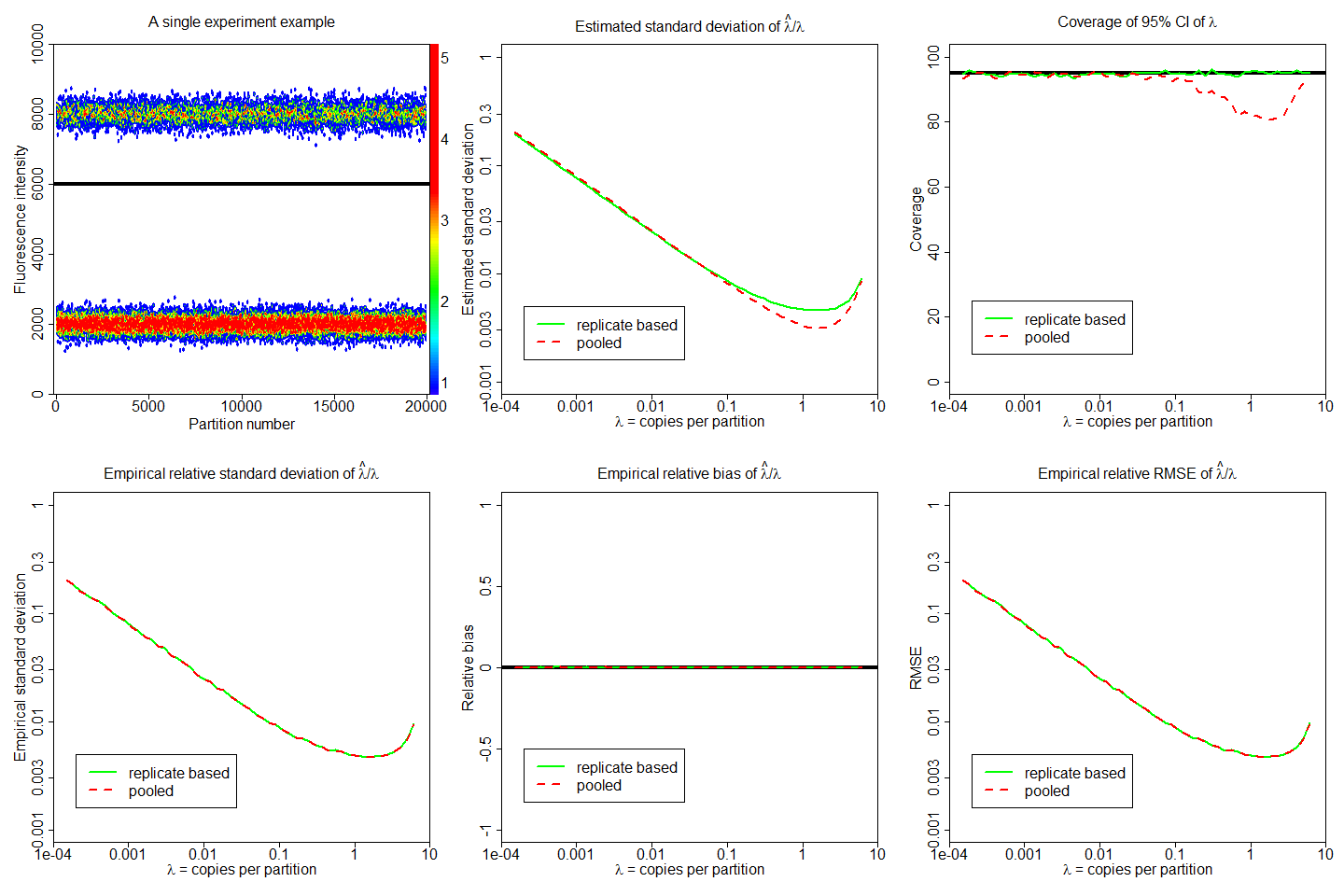

Supplement: Supplementary file 4 — Additional file 4: Interactive tool. In this mini-website, we provide an interactive tool to study the influence of specific sources of variation on the performance of the concentration estimators. This can serve as a guide when designing an experiment. All results are relative to the true concentration and based on 1000 simulations with 8 technical replicates. (ZIP 17 MB) [file 12859_2014_6687_MOESM4_ESM.zip › Additional file 4/RES/RES1231B.png]

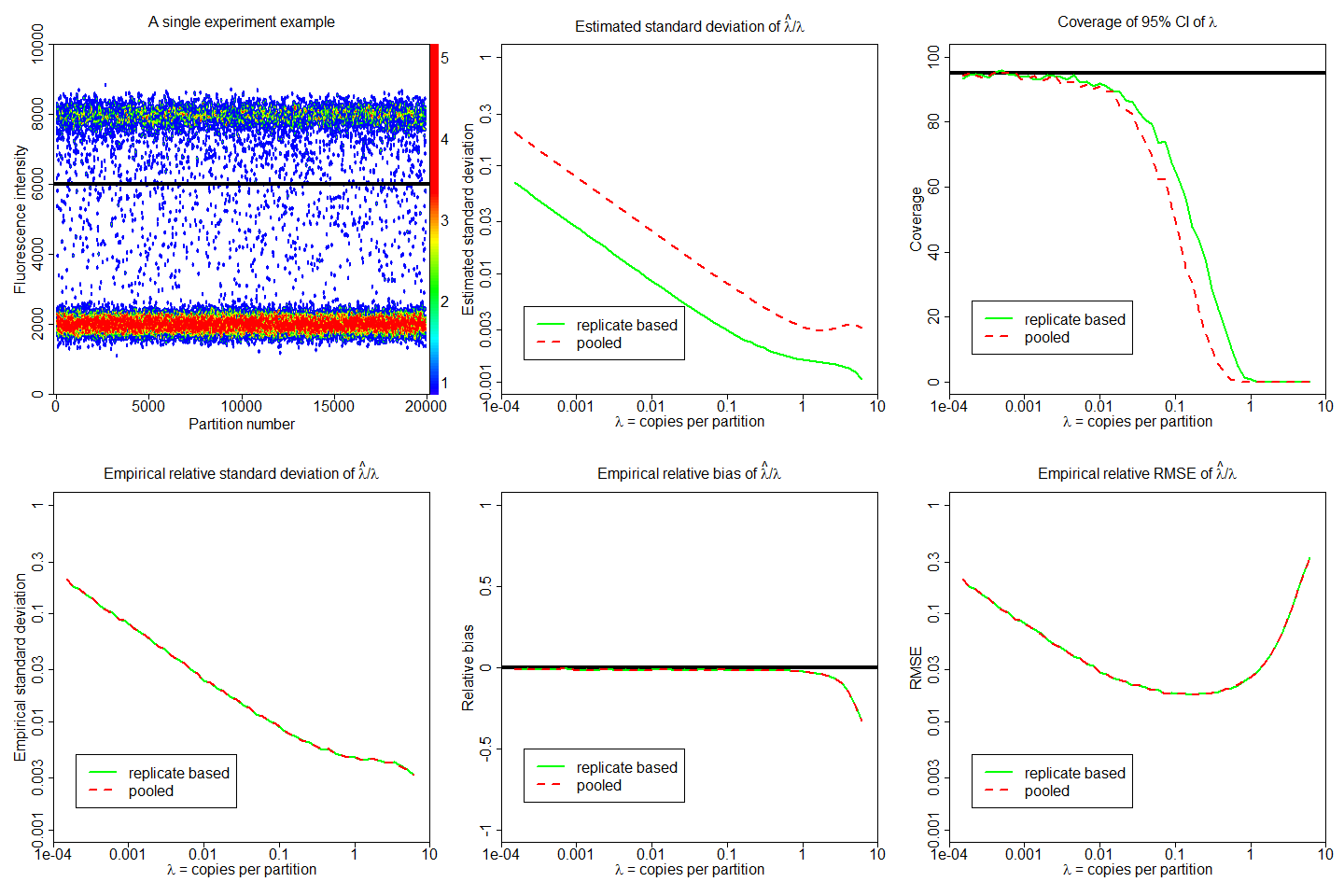

Supplement: Supplementary file 4 — Additional file 4: Interactive tool. In this mini-website, we provide an interactive tool to study the influence of specific sources of variation on the performance of the concentration estimators. This can serve as a guide when designing an experiment. All results are relative to the true concentration and based on 1000 simulations with 8 technical replicates. (ZIP 17 MB) [file 12859_2014_6687_MOESM4_ESM.zip › Additional file 4/RES/RES1232B.png]

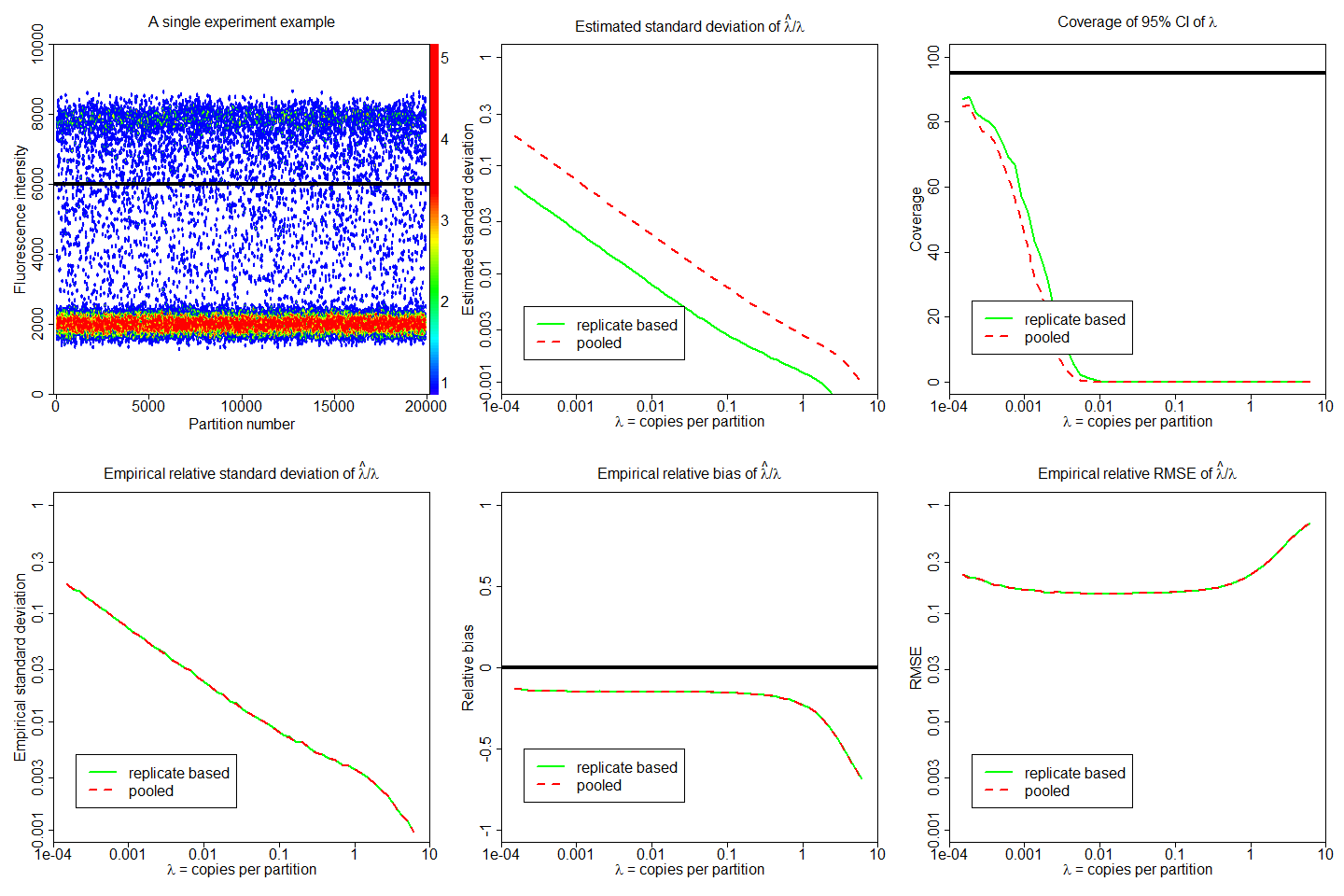

Supplement: Supplementary file 4 — Additional file 4: Interactive tool. In this mini-website, we provide an interactive tool to study the influence of specific sources of variation on the performance of the concentration estimators. This can serve as a guide when designing an experiment. All results are relative to the true concentration and based on 1000 simulations with 8 technical replicates. (ZIP 17 MB) [file 12859_2014_6687_MOESM4_ESM.zip › Additional file 4/RES/RES1233B.png]

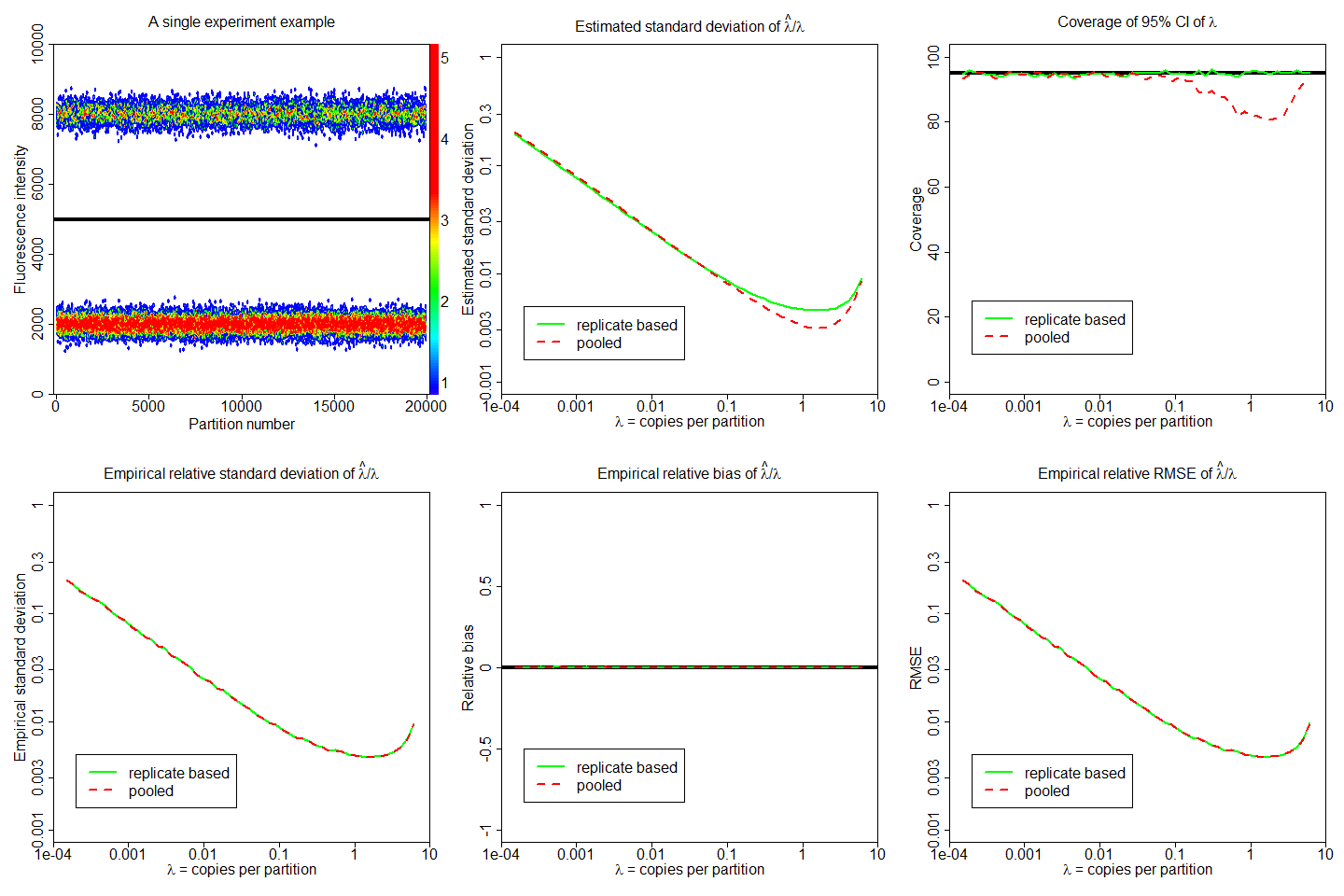

Supplement: Supplementary file 4 — Additional file 4: Interactive tool. In this mini-website, we provide an interactive tool to study the influence of specific sources of variation on the performance of the concentration estimators. This can serve as a guide when designing an experiment. All results are relative to the true concentration and based on 1000 simulations with 8 technical replicates. (ZIP 17 MB) [file 12859_2014_6687_MOESM4_ESM.zip › Additional file 4/RES/RES1241B.png]

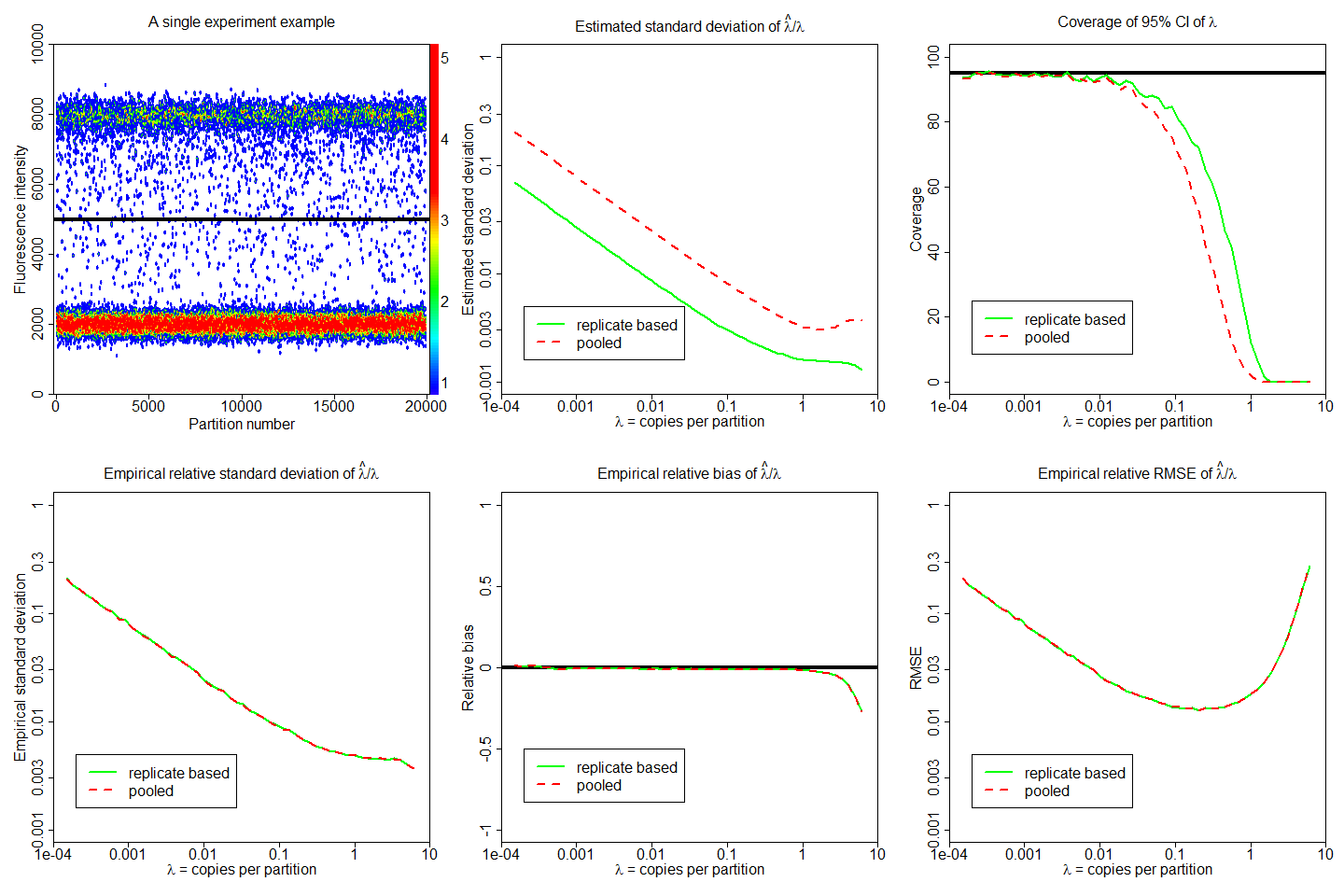

Supplement: Supplementary file 4 — Additional file 4: Interactive tool. In this mini-website, we provide an interactive tool to study the influence of specific sources of variation on the performance of the concentration estimators. This can serve as a guide when designing an experiment. All results are relative to the true concentration and based on 1000 simulations with 8 technical replicates. (ZIP 17 MB) [file 12859_2014_6687_MOESM4_ESM.zip › Additional file 4/RES/RES1242B.png]

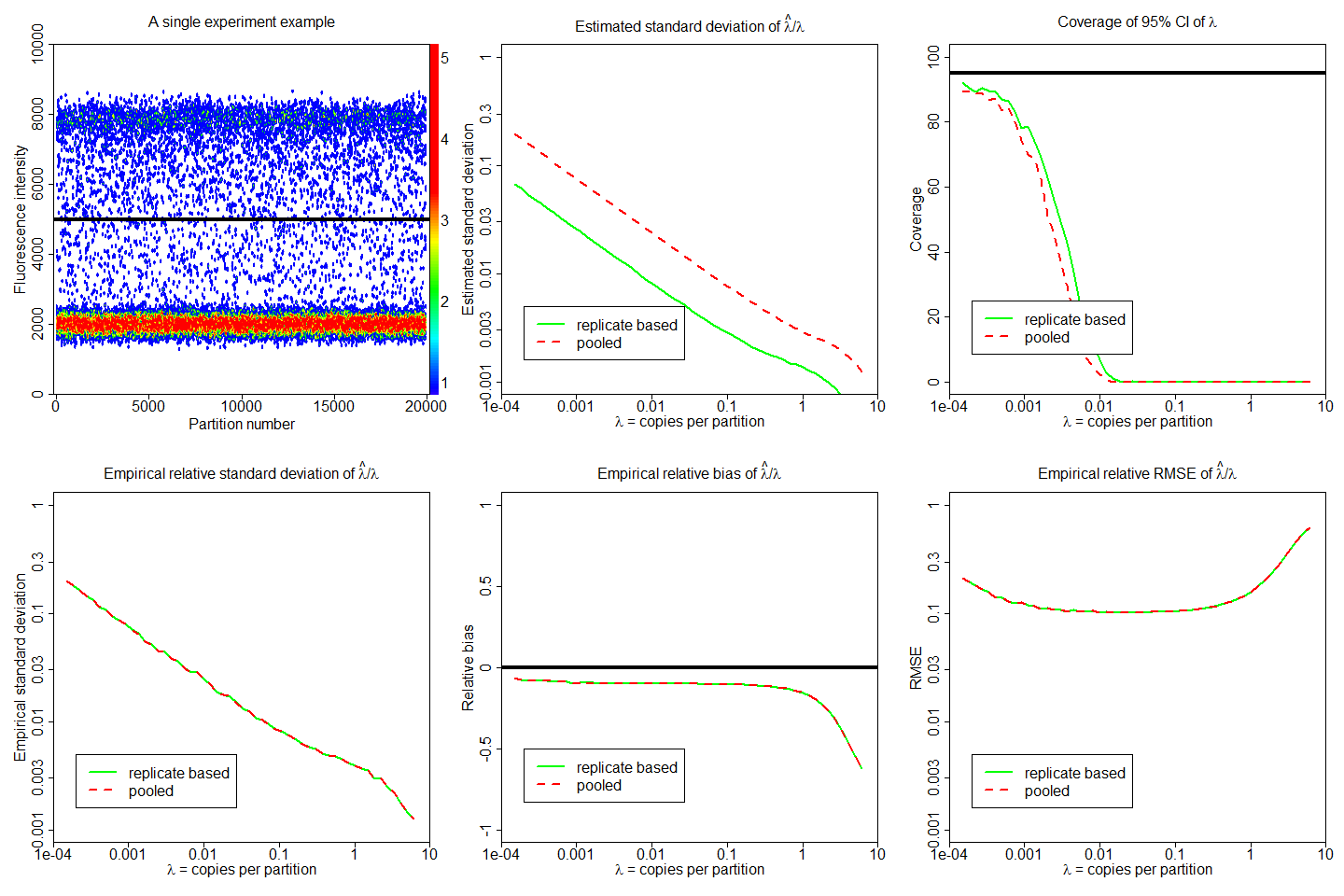

Supplement: Supplementary file 4 — Additional file 4: Interactive tool. In this mini-website, we provide an interactive tool to study the influence of specific sources of variation on the performance of the concentration estimators. This can serve as a guide when designing an experiment. All results are relative to the true concentration and based on 1000 simulations with 8 technical replicates. (ZIP 17 MB) [file 12859_2014_6687_MOESM4_ESM.zip › Additional file 4/RES/RES1243B.png]

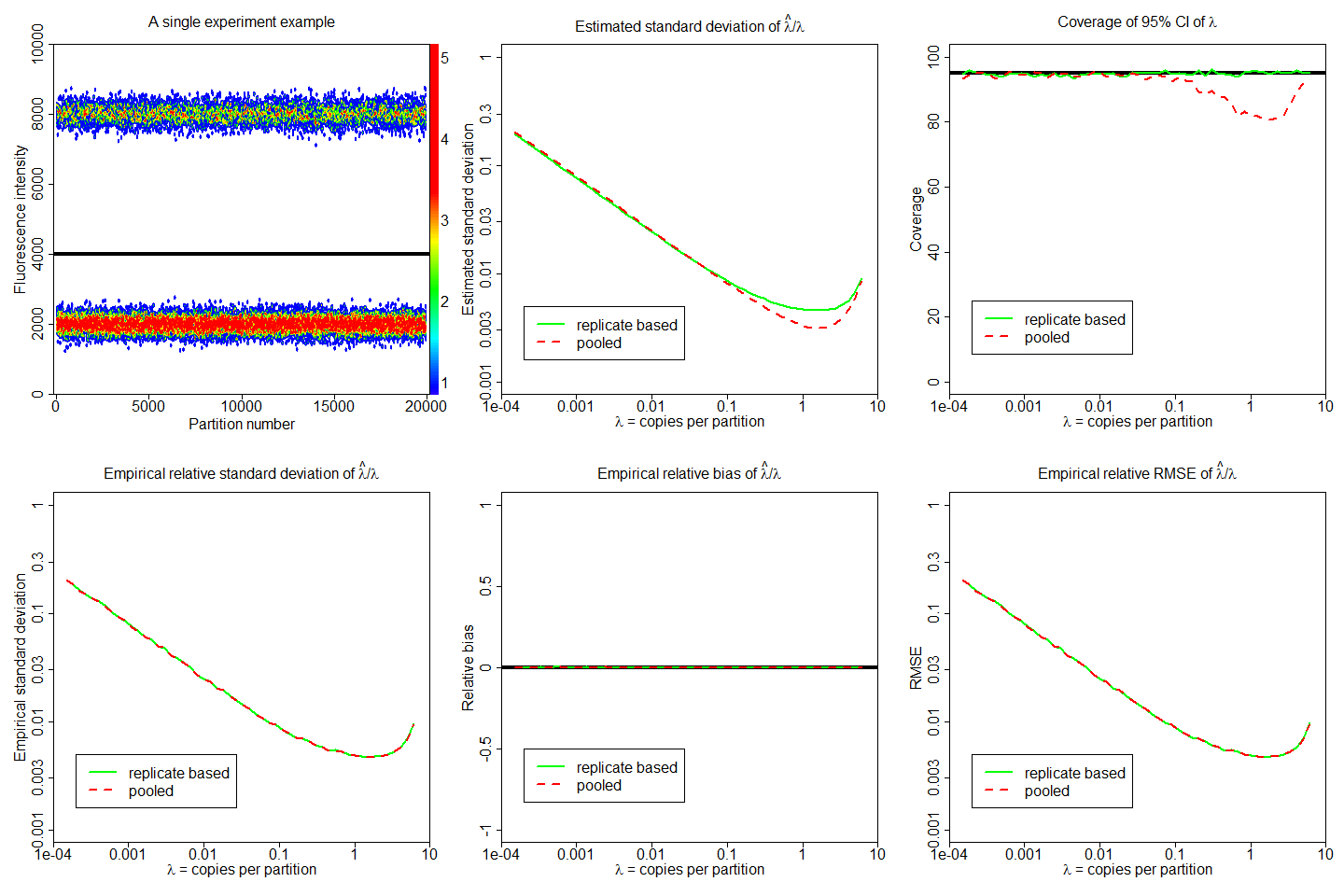

Supplement: Supplementary file 4 — Additional file 4: Interactive tool. In this mini-website, we provide an interactive tool to study the influence of specific sources of variation on the performance of the concentration estimators. This can serve as a guide when designing an experiment. All results are relative to the true concentration and based on 1000 simulations with 8 technical replicates. (ZIP 17 MB) [file 12859_2014_6687_MOESM4_ESM.zip › Additional file 4/RES/RES1251B.png]

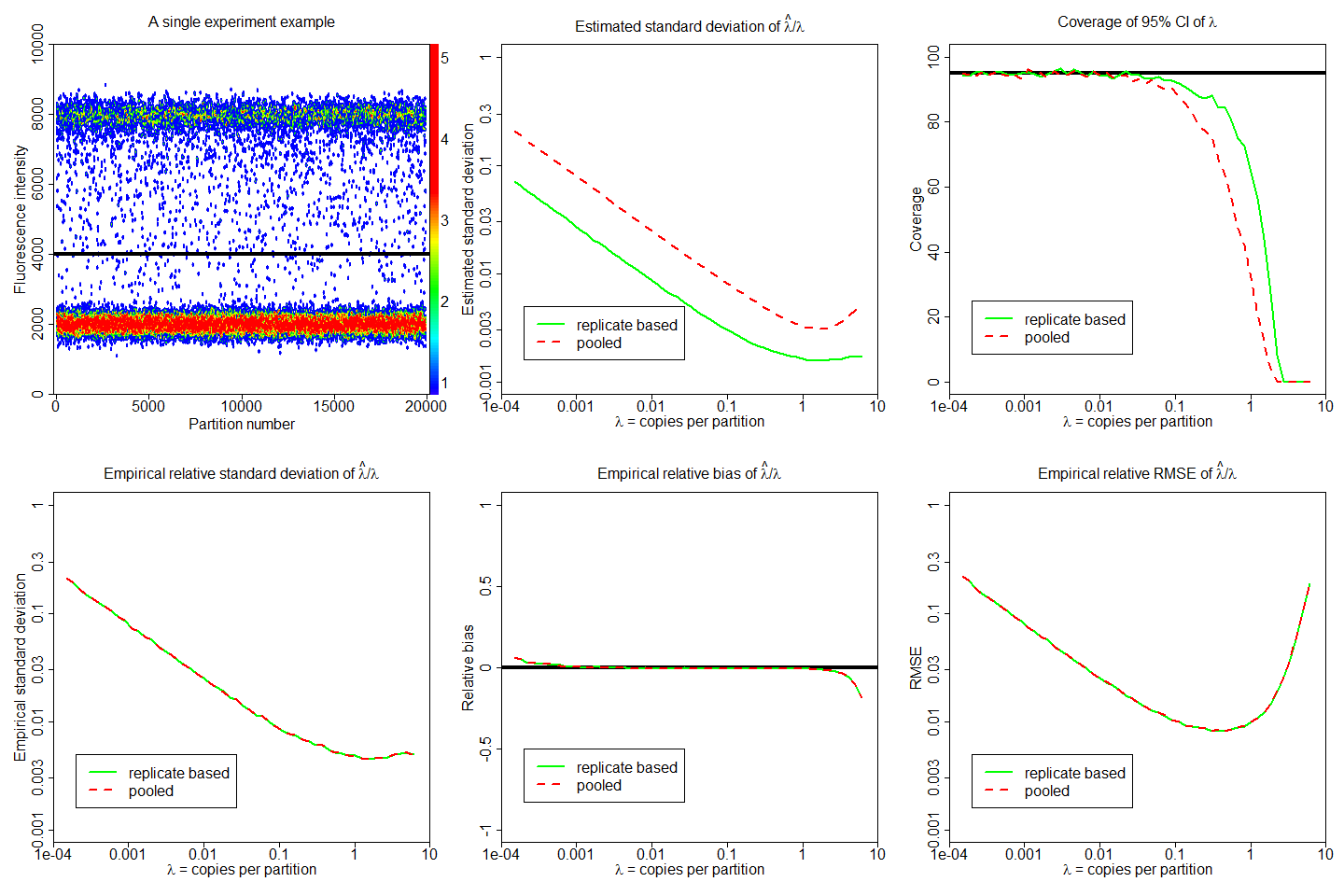

Supplement: Supplementary file 4 — Additional file 4: Interactive tool. In this mini-website, we provide an interactive tool to study the influence of specific sources of variation on the performance of the concentration estimators. This can serve as a guide when designing an experiment. All results are relative to the true concentration and based on 1000 simulations with 8 technical replicates. (ZIP 17 MB) [file 12859_2014_6687_MOESM4_ESM.zip › Additional file 4/RES/RES1252B.png]

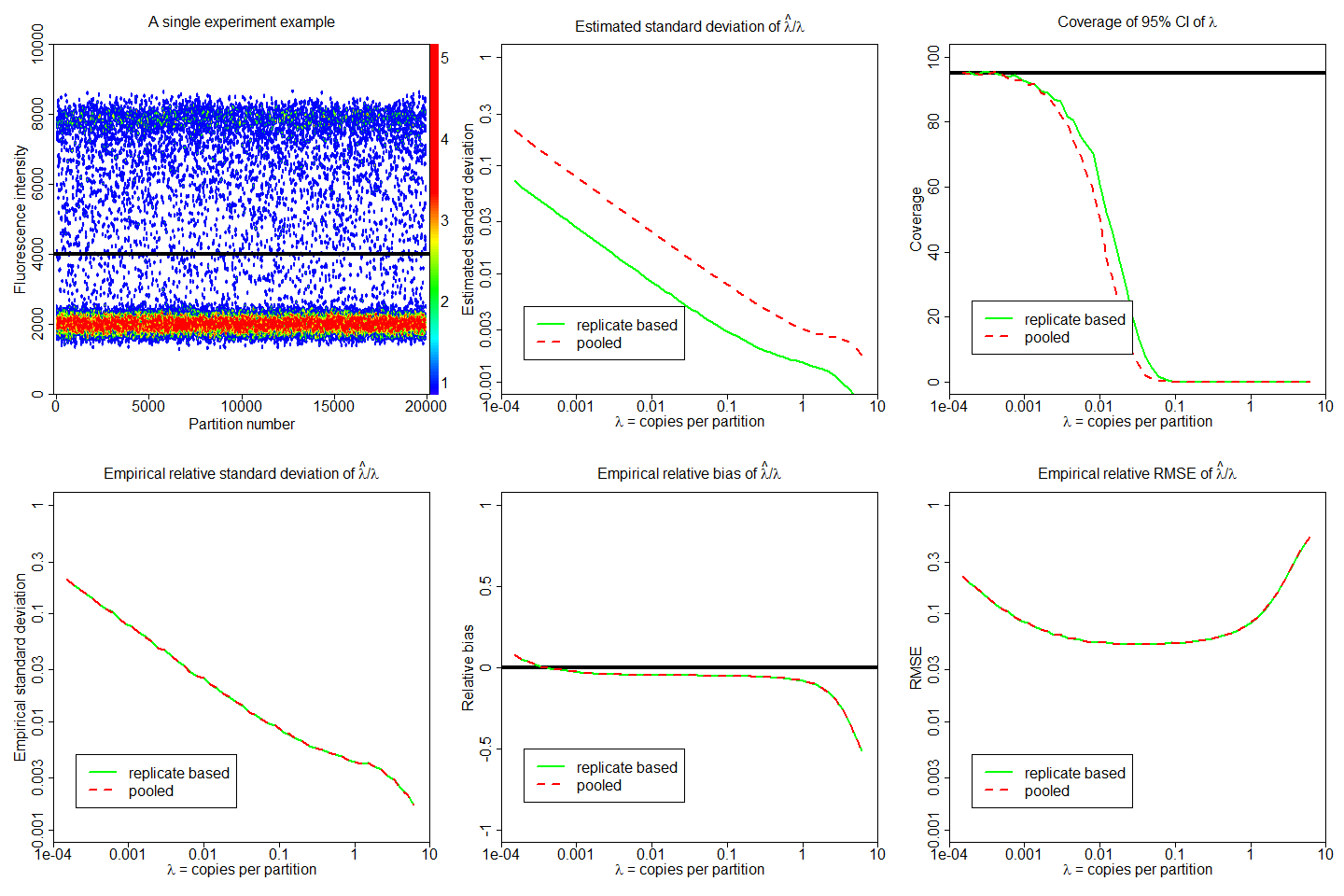

Supplement: Supplementary file 4 — Additional file 4: Interactive tool. In this mini-website, we provide an interactive tool to study the influence of specific sources of variation on the performance of the concentration estimators. This can serve as a guide when designing an experiment. All results are relative to the true concentration and based on 1000 simulations with 8 technical replicates. (ZIP 17 MB) [file 12859_2014_6687_MOESM4_ESM.zip › Additional file 4/RES/RES1253B.png]

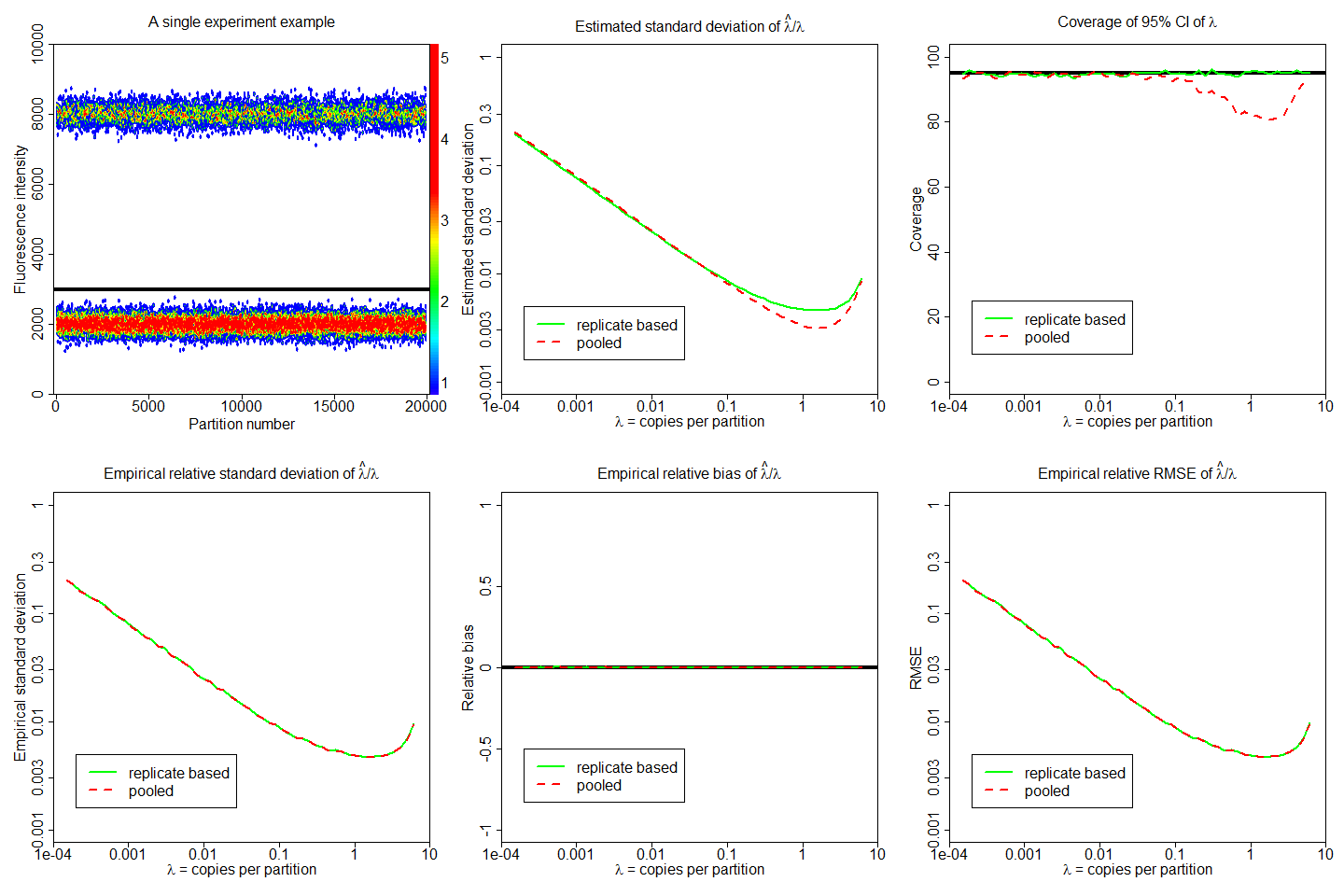

Supplement: Supplementary file 4 — Additional file 4: Interactive tool. In this mini-website, we provide an interactive tool to study the influence of specific sources of variation on the performance of the concentration estimators. This can serve as a guide when designing an experiment. All results are relative to the true concentration and based on 1000 simulations with 8 technical replicates. (ZIP 17 MB) [file 12859_2014_6687_MOESM4_ESM.zip › Additional file 4/RES/RES1261B.png]

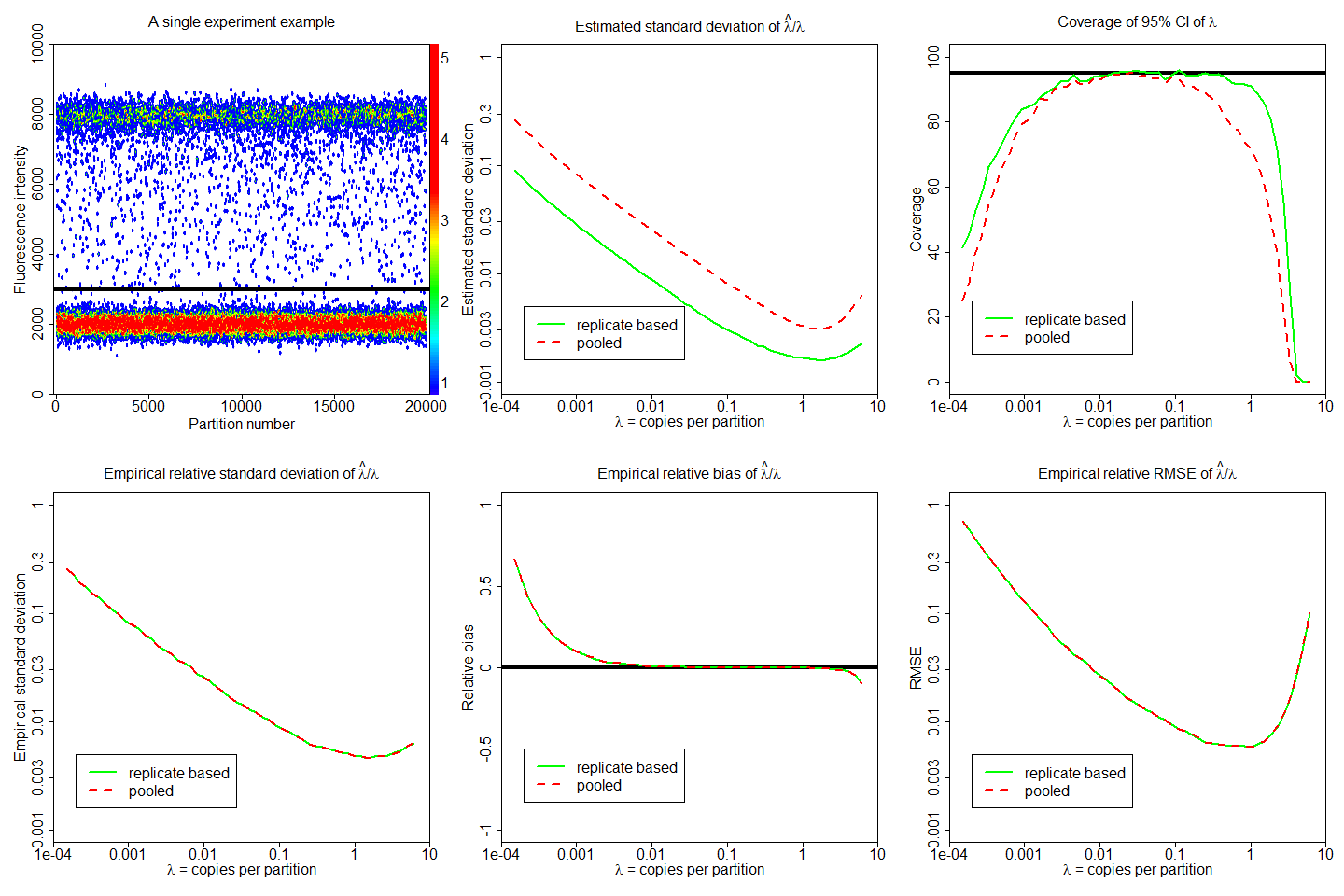

Supplement: Supplementary file 4 — Additional file 4: Interactive tool. In this mini-website, we provide an interactive tool to study the influence of specific sources of variation on the performance of the concentration estimators. This can serve as a guide when designing an experiment. All results are relative to the true concentration and based on 1000 simulations with 8 technical replicates. (ZIP 17 MB) [file 12859_2014_6687_MOESM4_ESM.zip › Additional file 4/RES/RES1262B.png]

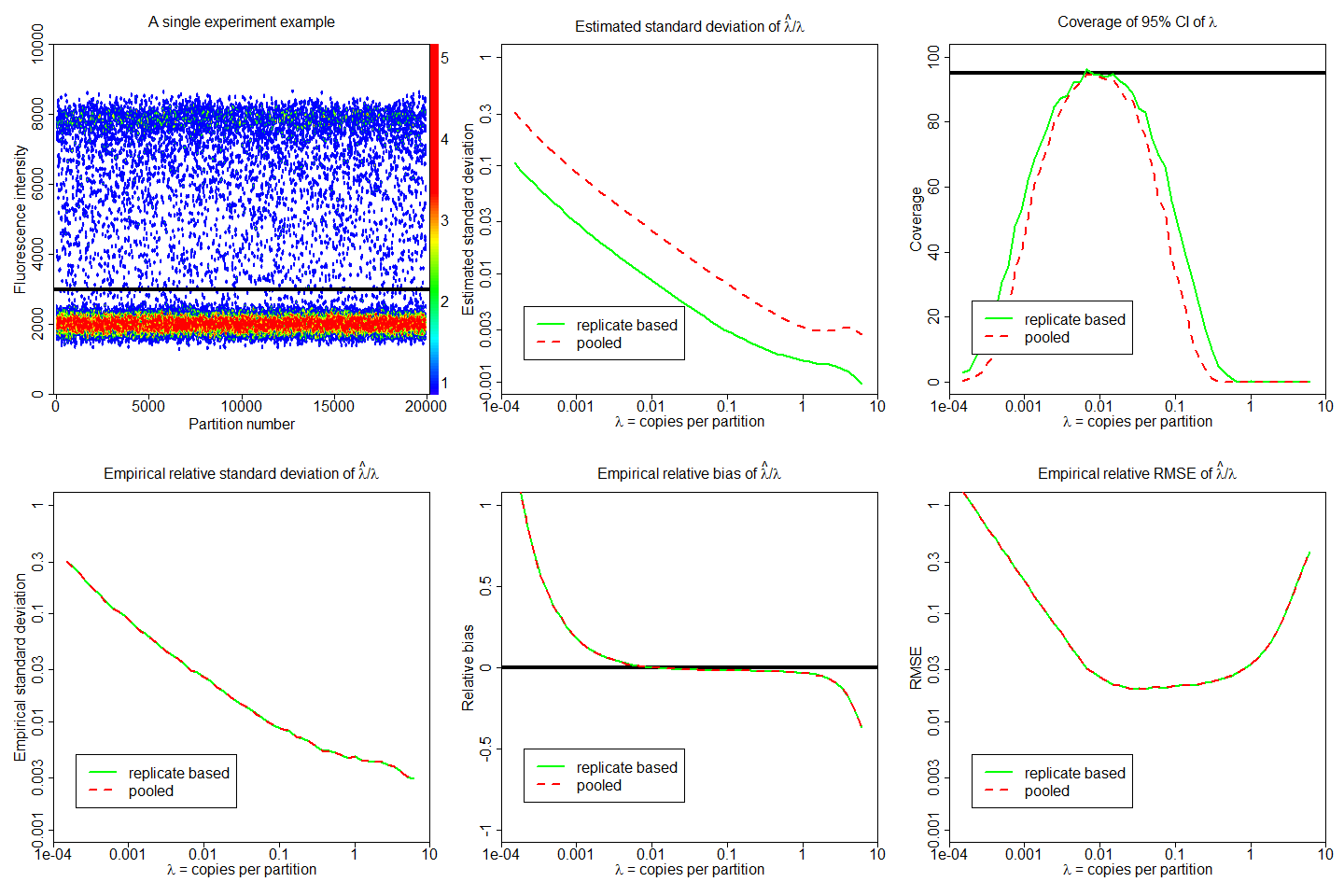

Supplement: Supplementary file 4 — Additional file 4: Interactive tool. In this mini-website, we provide an interactive tool to study the influence of specific sources of variation on the performance of the concentration estimators. This can serve as a guide when designing an experiment. All results are relative to the true concentration and based on 1000 simulations with 8 technical replicates. (ZIP 17 MB) [file 12859_2014_6687_MOESM4_ESM.zip › Additional file 4/RES/RES1263B.png]

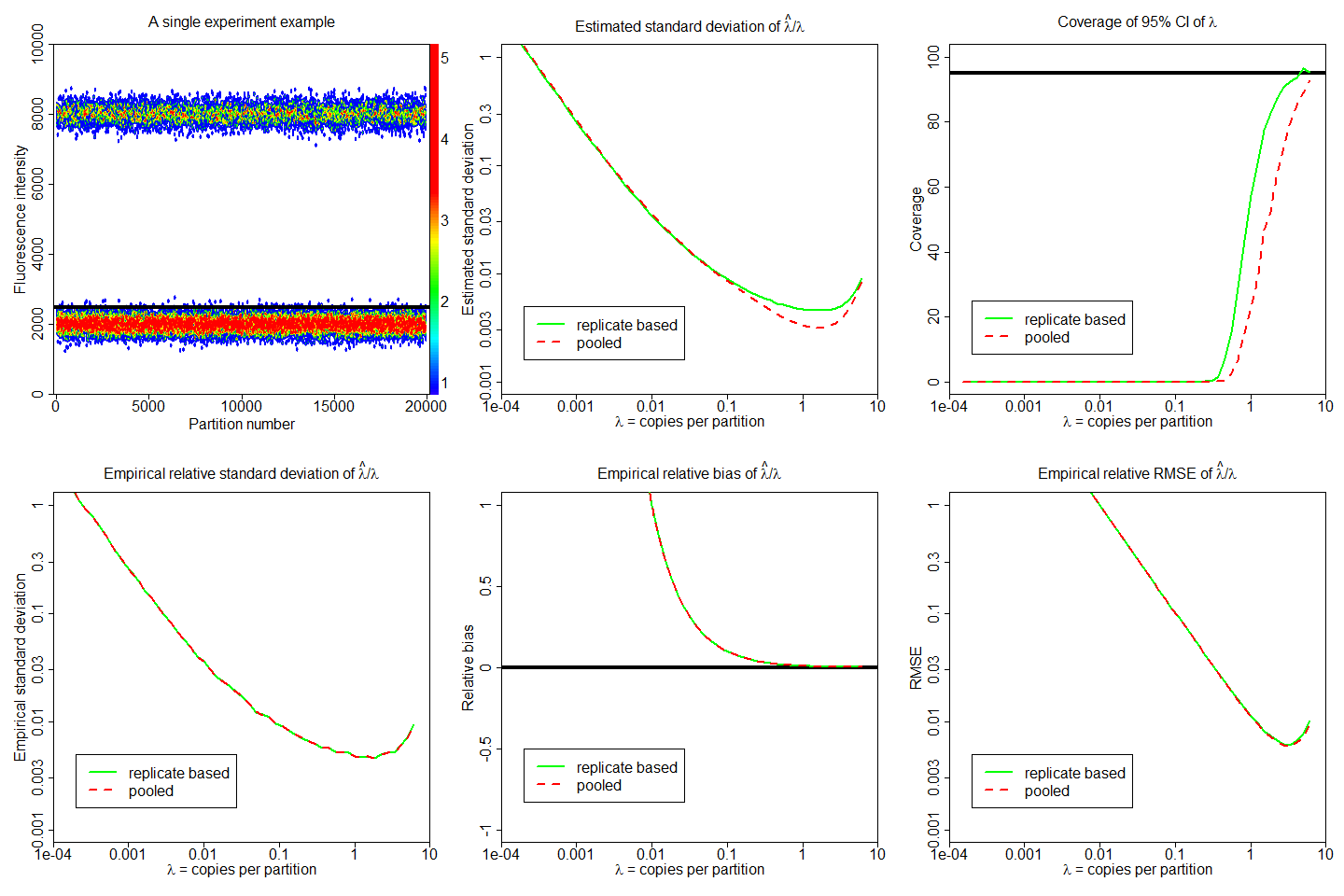

Supplement: Supplementary file 4 — Additional file 4: Interactive tool. In this mini-website, we provide an interactive tool to study the influence of specific sources of variation on the performance of the concentration estimators. This can serve as a guide when designing an experiment. All results are relative to the true concentration and based on 1000 simulations with 8 technical replicates. (ZIP 17 MB) [file 12859_2014_6687_MOESM4_ESM.zip › Additional file 4/RES/RES1271B.png]

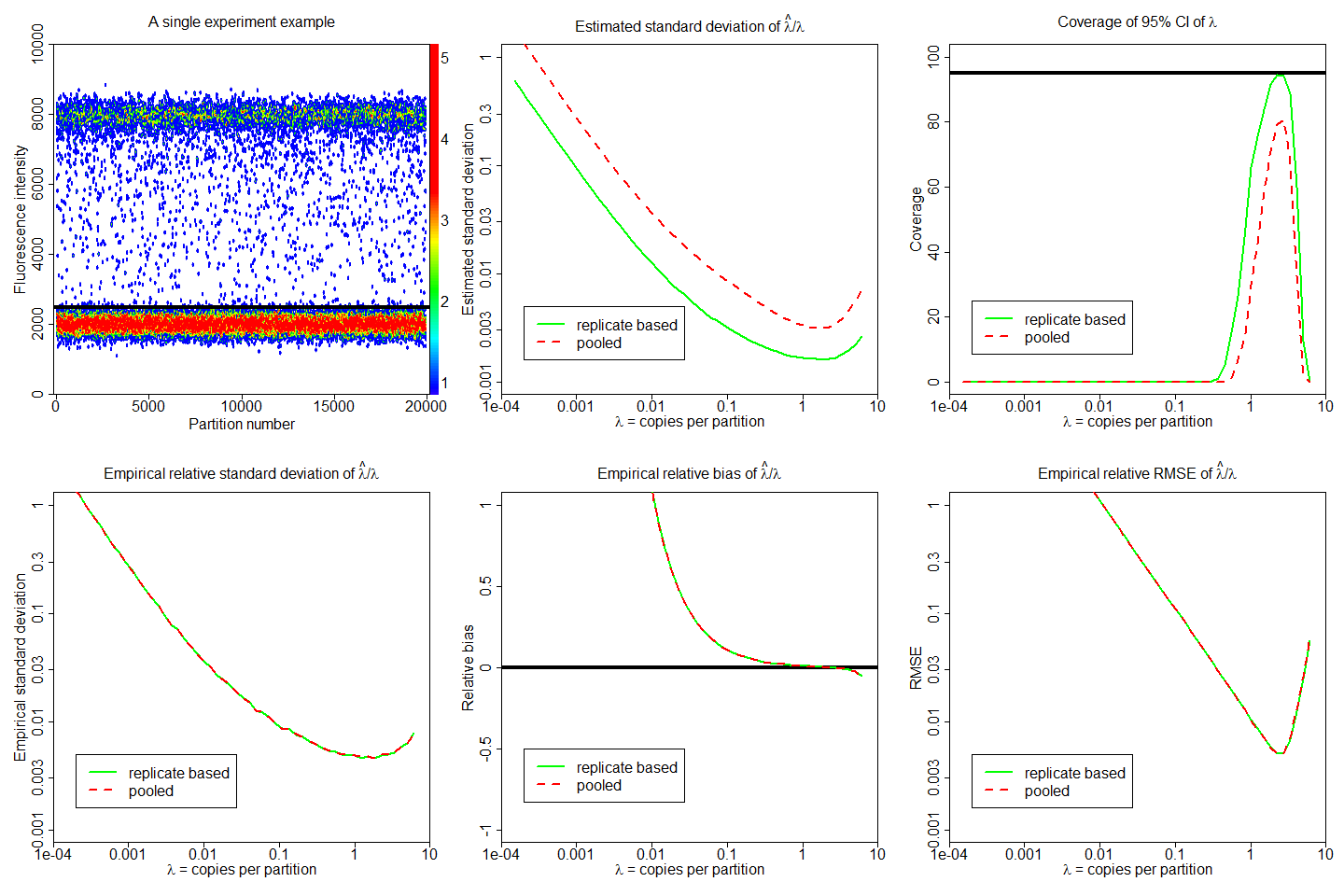

Supplement: Supplementary file 4 — Additional file 4: Interactive tool. In this mini-website, we provide an interactive tool to study the influence of specific sources of variation on the performance of the concentration estimators. This can serve as a guide when designing an experiment. All results are relative to the true concentration and based on 1000 simulations with 8 technical replicates. (ZIP 17 MB) [file 12859_2014_6687_MOESM4_ESM.zip › Additional file 4/RES/RES1272B.png]

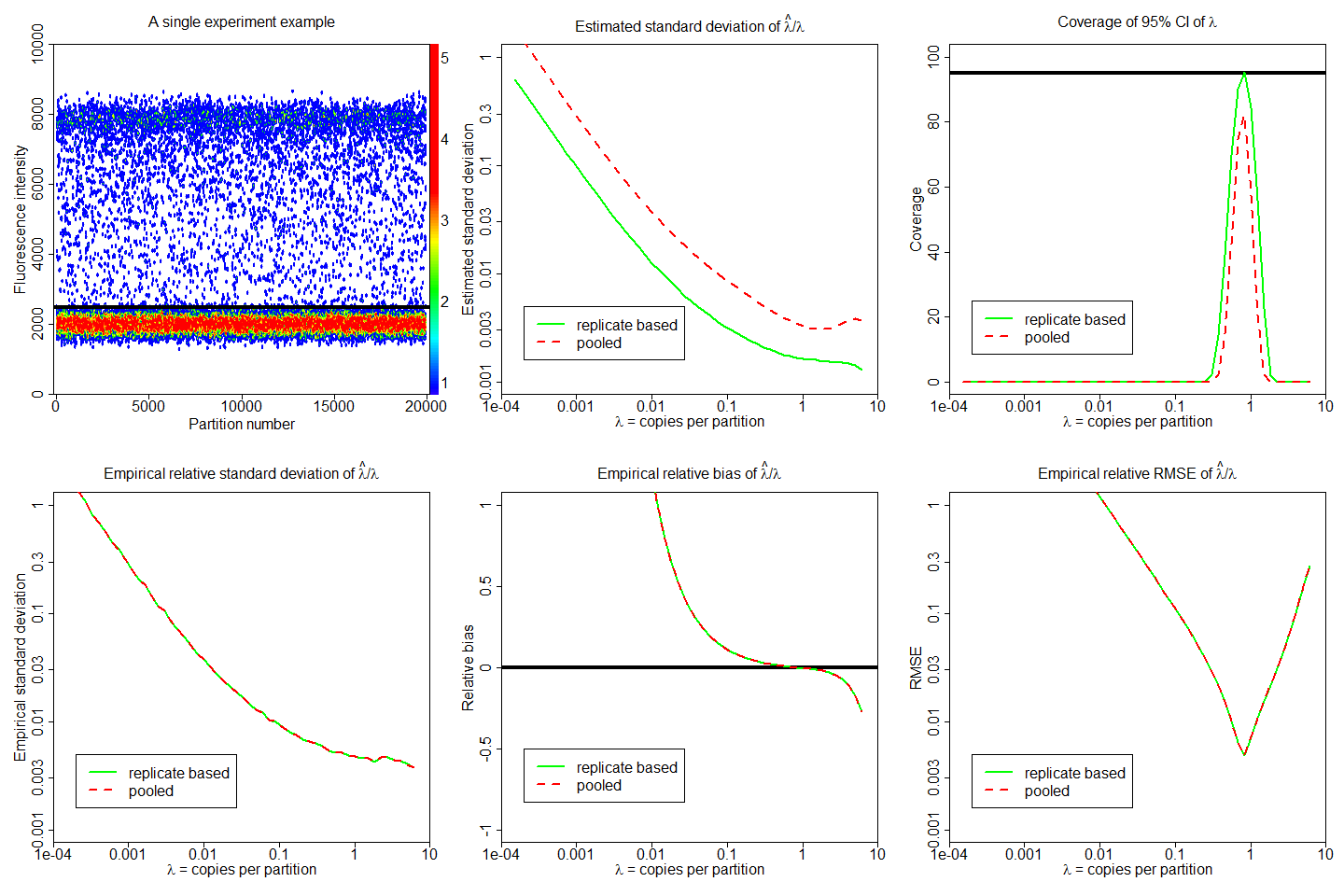

Supplement: Supplementary file 4 — Additional file 4: Interactive tool. In this mini-website, we provide an interactive tool to study the influence of specific sources of variation on the performance of the concentration estimators. This can serve as a guide when designing an experiment. All results are relative to the true concentration and based on 1000 simulations with 8 technical replicates. (ZIP 17 MB) [file 12859_2014_6687_MOESM4_ESM.zip › Additional file 4/RES/RES1273B.png]

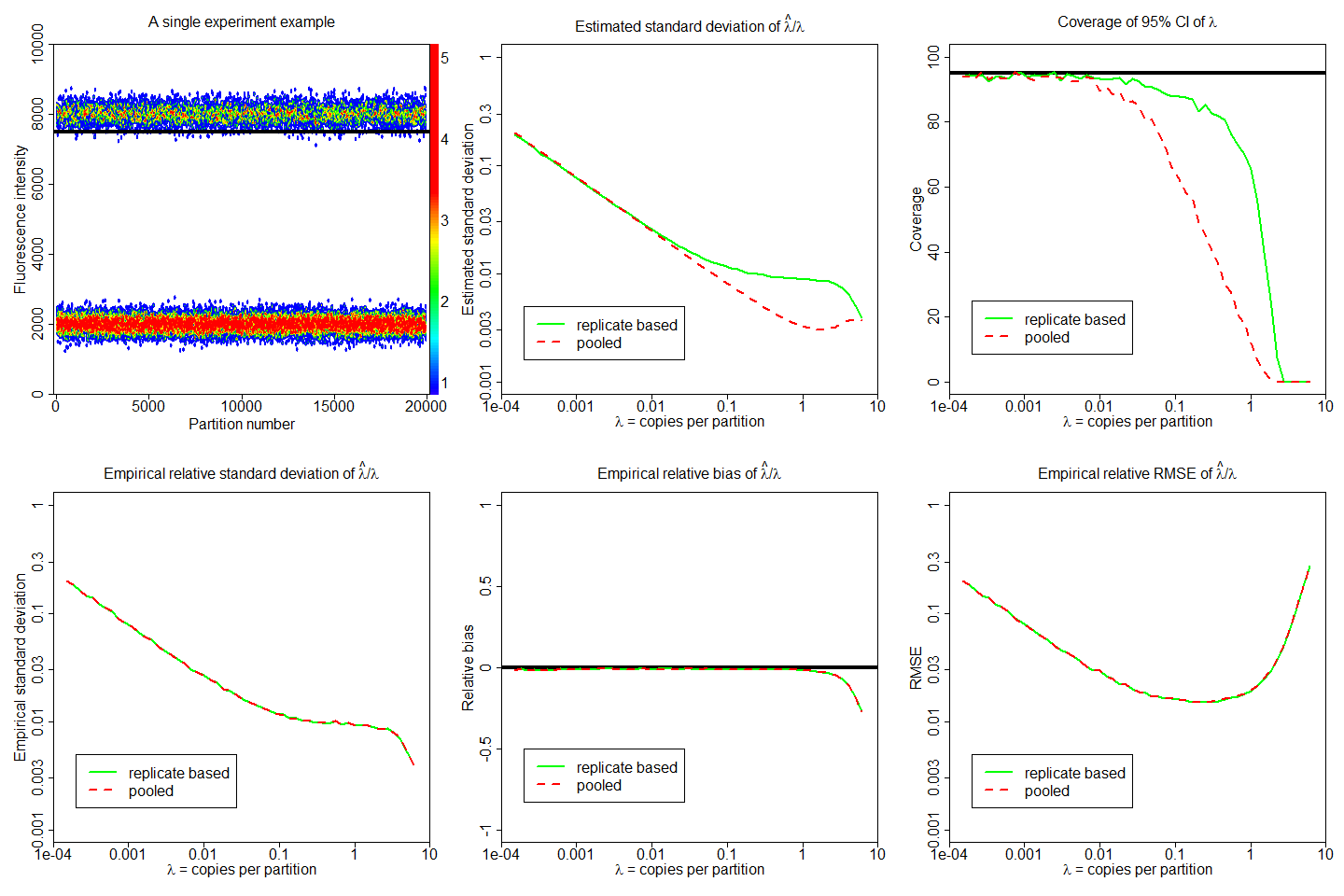

Supplement: Supplementary file 4 — Additional file 4: Interactive tool. In this mini-website, we provide an interactive tool to study the influence of specific sources of variation on the performance of the concentration estimators. This can serve as a guide when designing an experiment. All results are relative to the true concentration and based on 1000 simulations with 8 technical replicates. (ZIP 17 MB) [file 12859_2014_6687_MOESM4_ESM.zip › Additional file 4/RES/RES1311B.png]

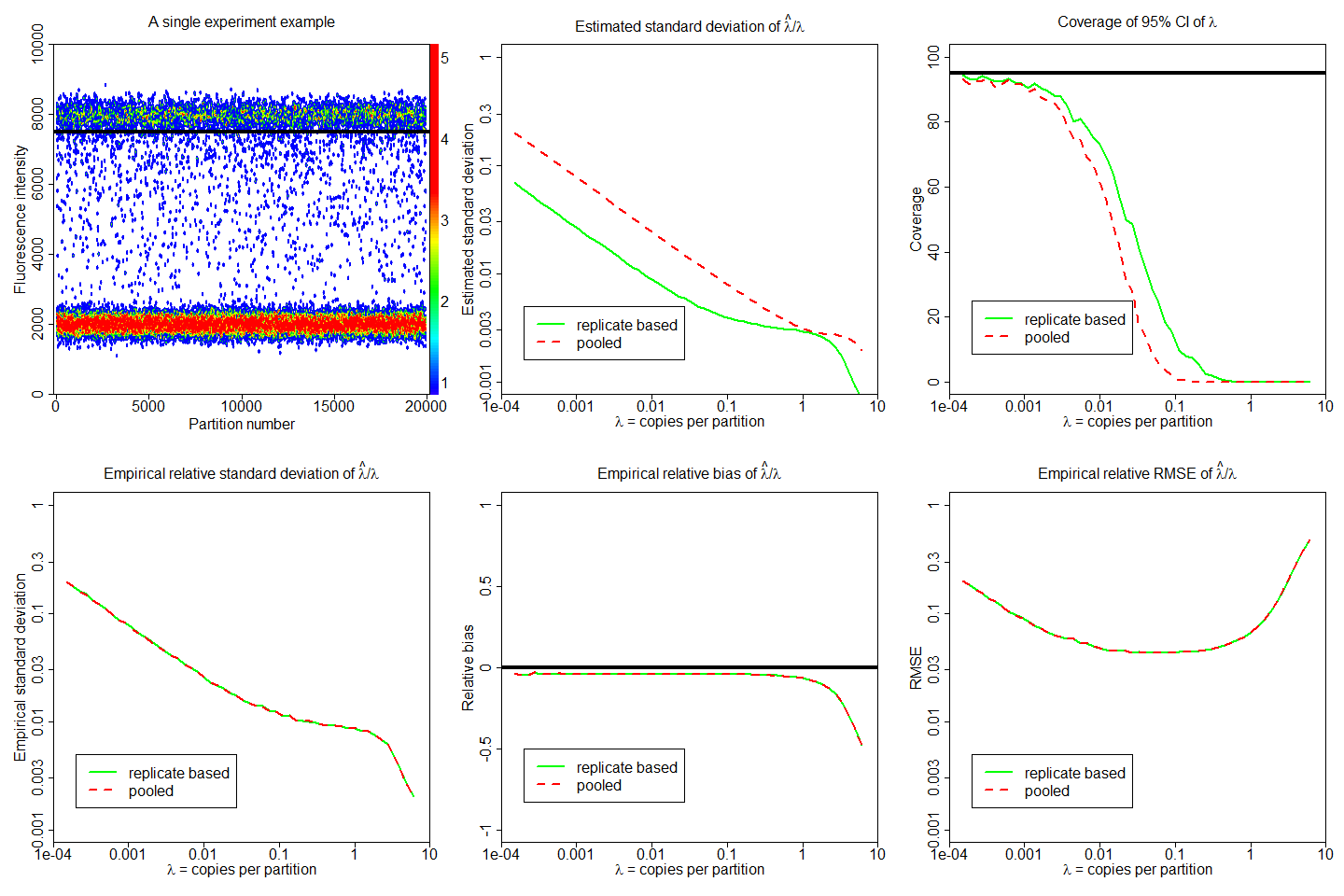

Supplement: Supplementary file 4 — Additional file 4: Interactive tool. In this mini-website, we provide an interactive tool to study the influence of specific sources of variation on the performance of the concentration estimators. This can serve as a guide when designing an experiment. All results are relative to the true concentration and based on 1000 simulations with 8 technical replicates. (ZIP 17 MB) [file 12859_2014_6687_MOESM4_ESM.zip › Additional file 4/RES/RES1312B.png]

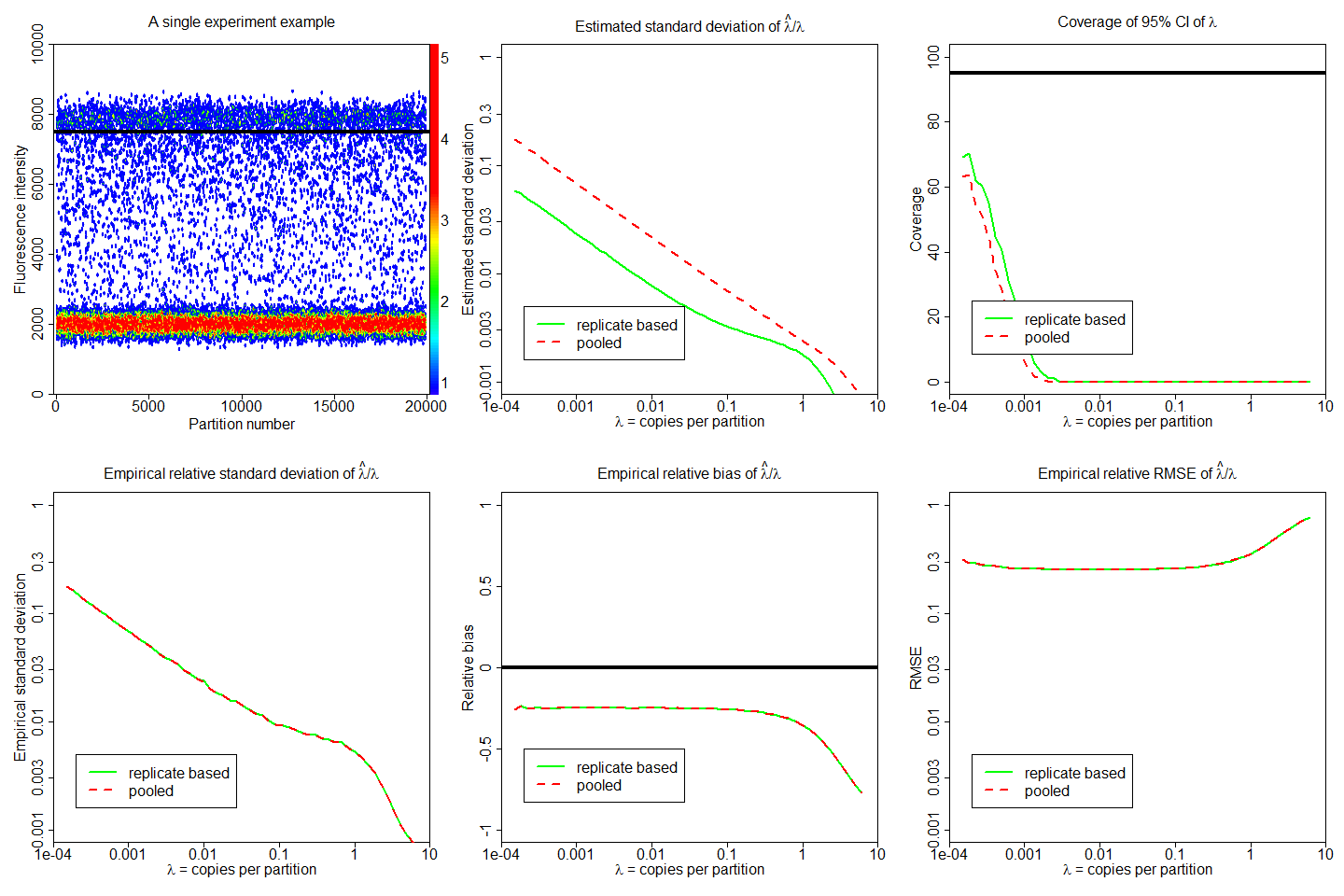

Supplement: Supplementary file 4 — Additional file 4: Interactive tool. In this mini-website, we provide an interactive tool to study the influence of specific sources of variation on the performance of the concentration estimators. This can serve as a guide when designing an experiment. All results are relative to the true concentration and based on 1000 simulations with 8 technical replicates. (ZIP 17 MB) [file 12859_2014_6687_MOESM4_ESM.zip › Additional file 4/RES/RES1313B.png]

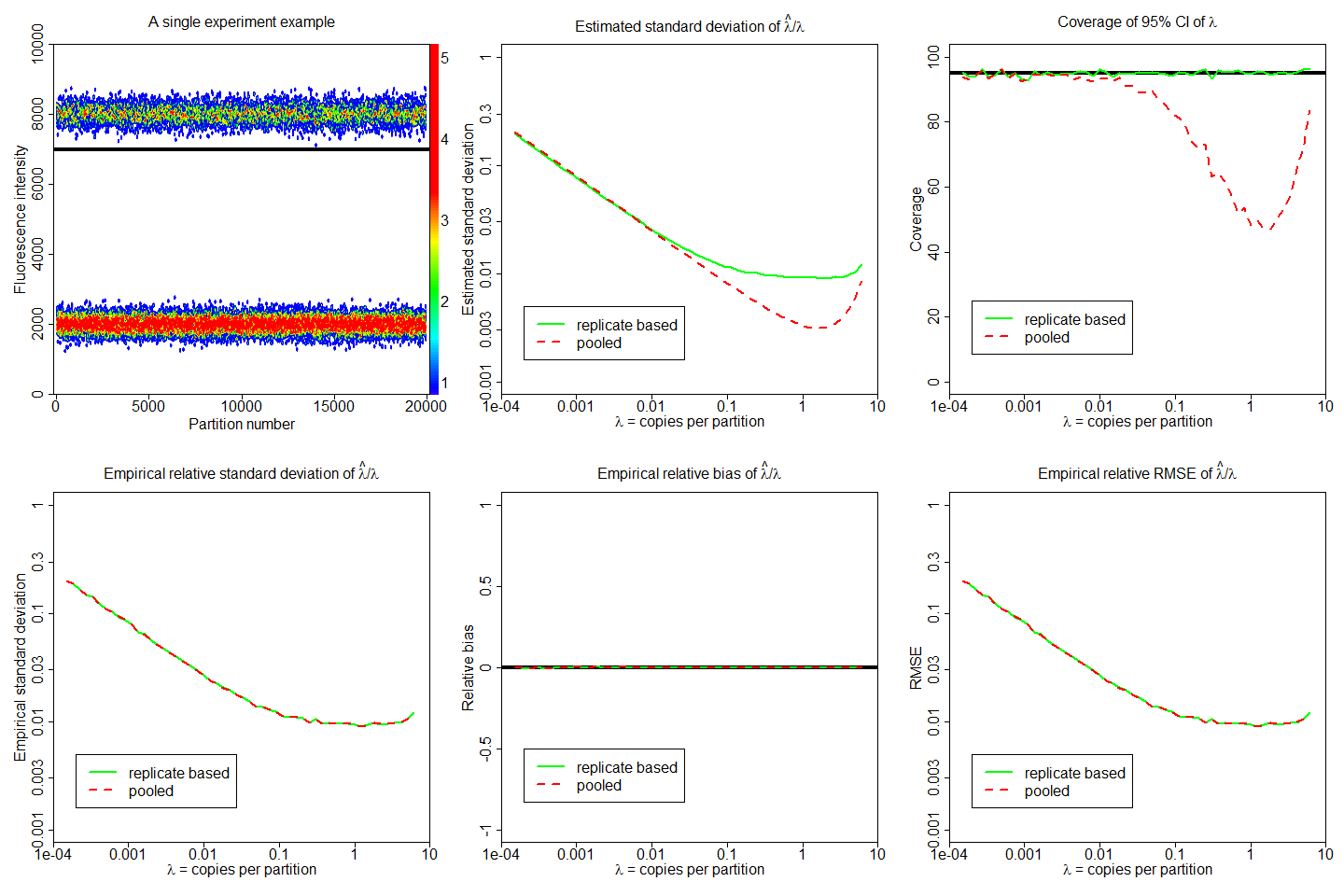

Supplement: Supplementary file 4 — Additional file 4: Interactive tool. In this mini-website, we provide an interactive tool to study the influence of specific sources of variation on the performance of the concentration estimators. This can serve as a guide when designing an experiment. All results are relative to the true concentration and based on 1000 simulations with 8 technical replicates. (ZIP 17 MB) [file 12859_2014_6687_MOESM4_ESM.zip › Additional file 4/RES/RES1321B.png]

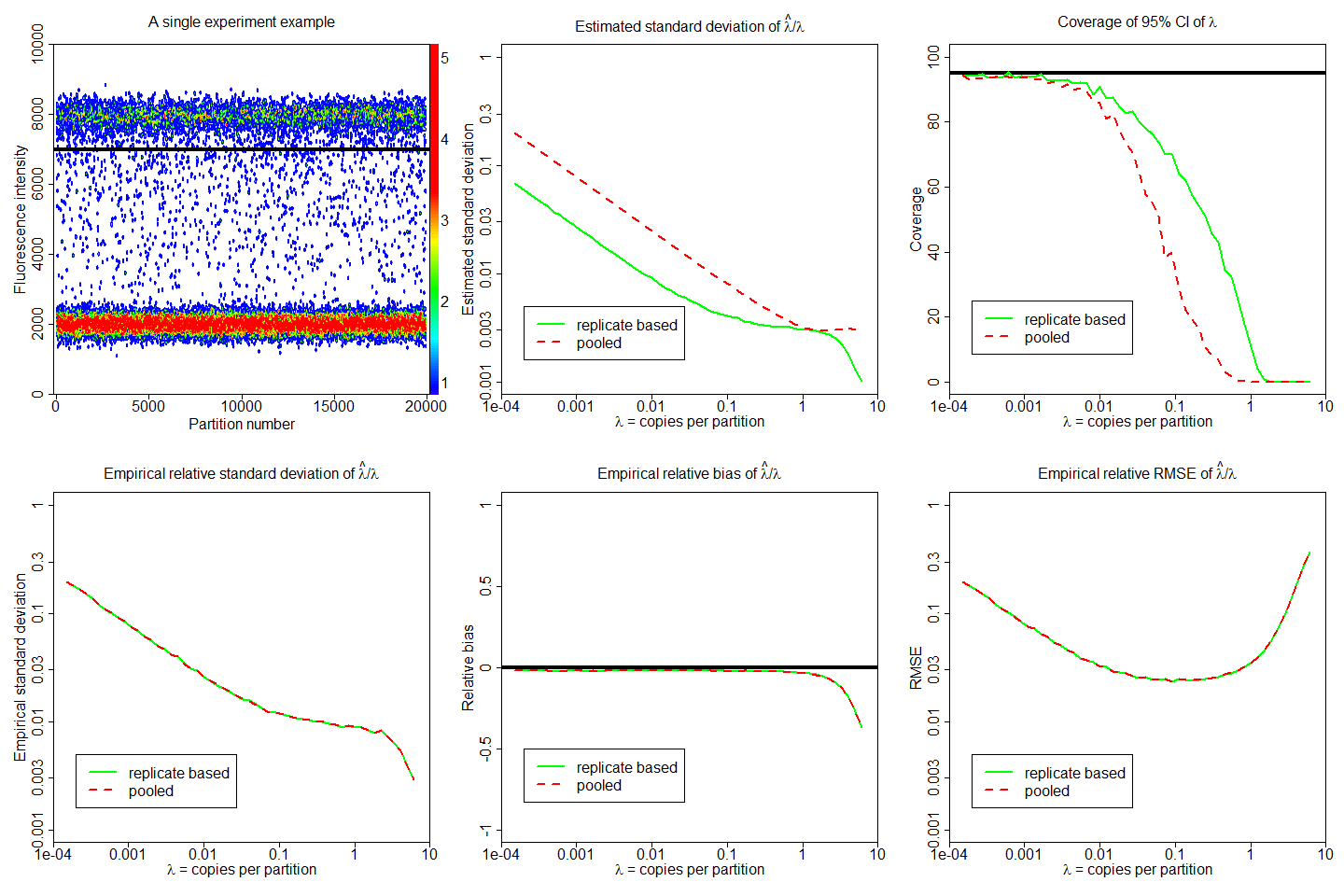

Supplement: Supplementary file 4 — Additional file 4: Interactive tool. In this mini-website, we provide an interactive tool to study the influence of specific sources of variation on the performance of the concentration estimators. This can serve as a guide when designing an experiment. All results are relative to the true concentration and based on 1000 simulations with 8 technical replicates. (ZIP 17 MB) [file 12859_2014_6687_MOESM4_ESM.zip › Additional file 4/RES/RES1322B.png]

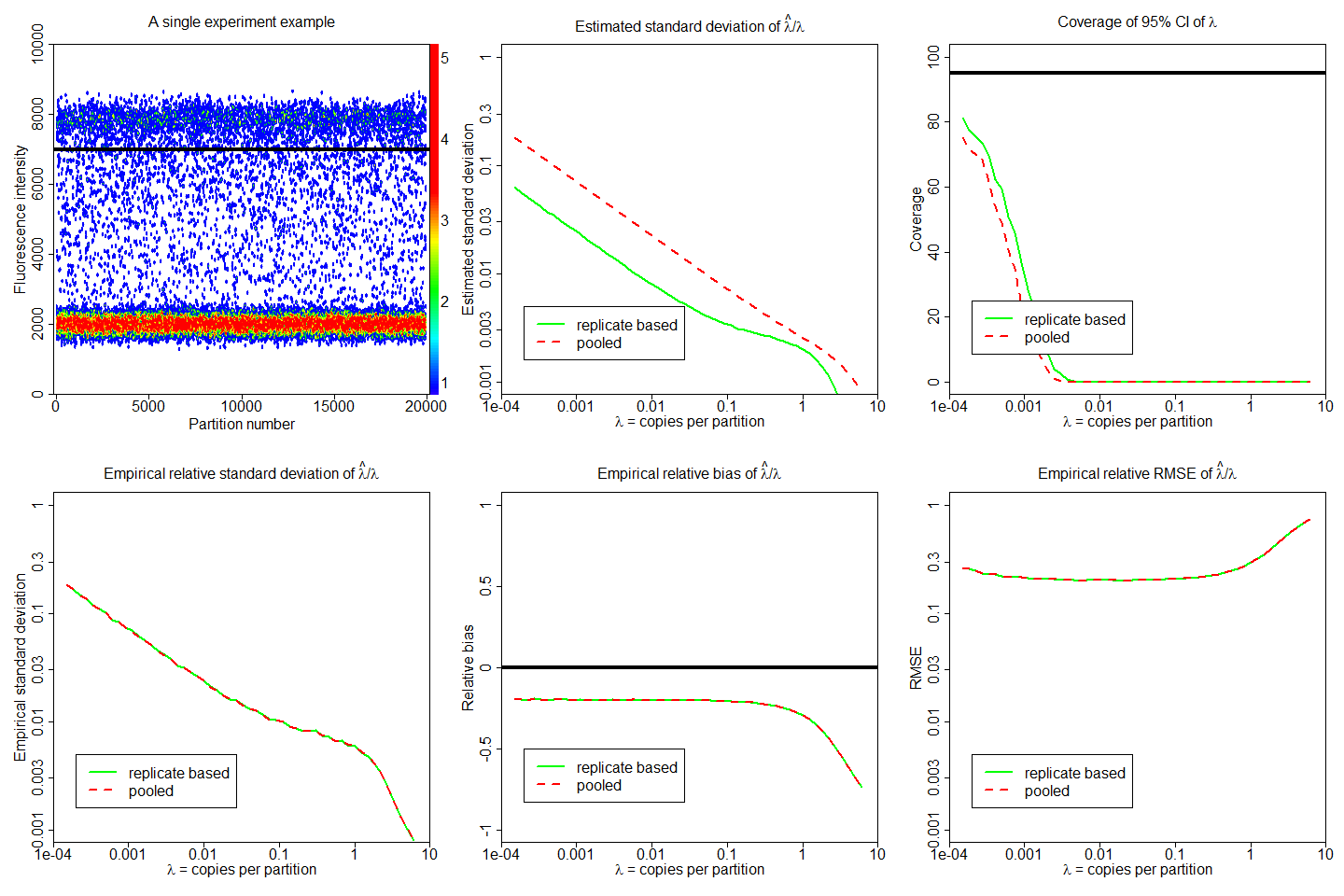

Supplement: Supplementary file 4 — Additional file 4: Interactive tool. In this mini-website, we provide an interactive tool to study the influence of specific sources of variation on the performance of the concentration estimators. This can serve as a guide when designing an experiment. All results are relative to the true concentration and based on 1000 simulations with 8 technical replicates. (ZIP 17 MB) [file 12859_2014_6687_MOESM4_ESM.zip › Additional file 4/RES/RES1323B.png]

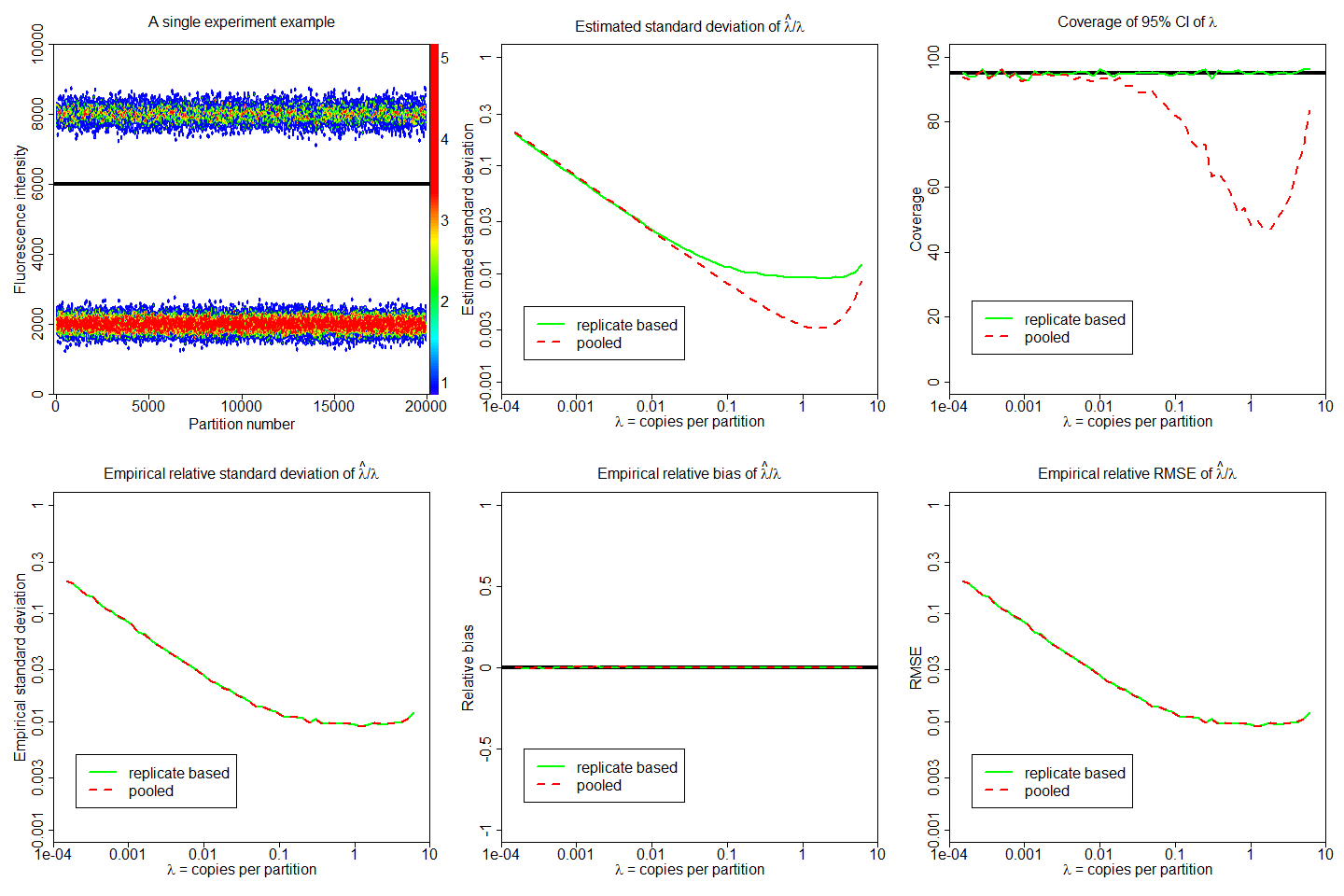

Supplement: Supplementary file 4 — Additional file 4: Interactive tool. In this mini-website, we provide an interactive tool to study the influence of specific sources of variation on the performance of the concentration estimators. This can serve as a guide when designing an experiment. All results are relative to the true concentration and based on 1000 simulations with 8 technical replicates. (ZIP 17 MB) [file 12859_2014_6687_MOESM4_ESM.zip › Additional file 4/RES/RES1331B.png]

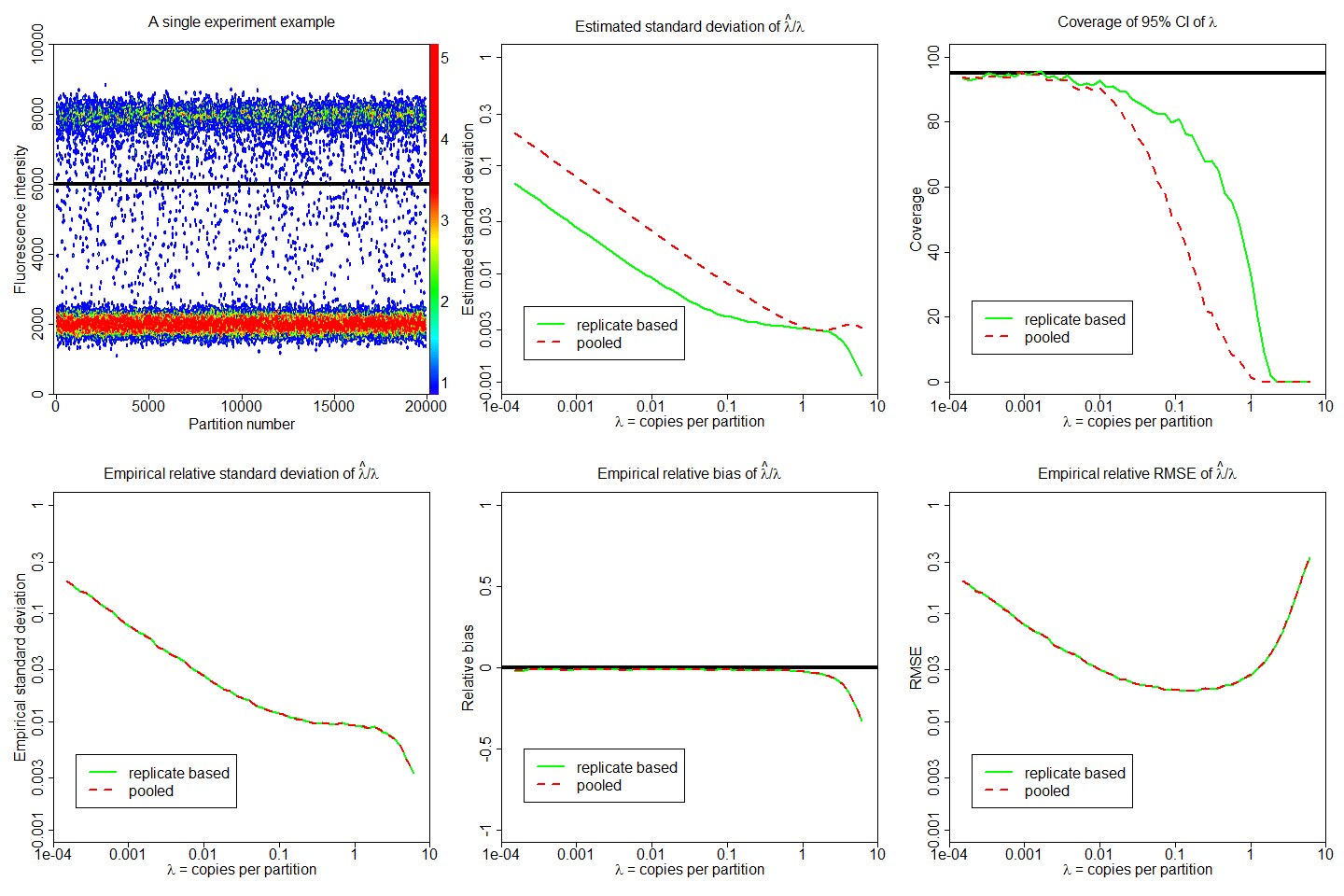

Supplement: Supplementary file 4 — Additional file 4: Interactive tool. In this mini-website, we provide an interactive tool to study the influence of specific sources of variation on the performance of the concentration estimators. This can serve as a guide when designing an experiment. All results are relative to the true concentration and based on 1000 simulations with 8 technical replicates. (ZIP 17 MB) [file 12859_2014_6687_MOESM4_ESM.zip › Additional file 4/RES/RES1332B.png]

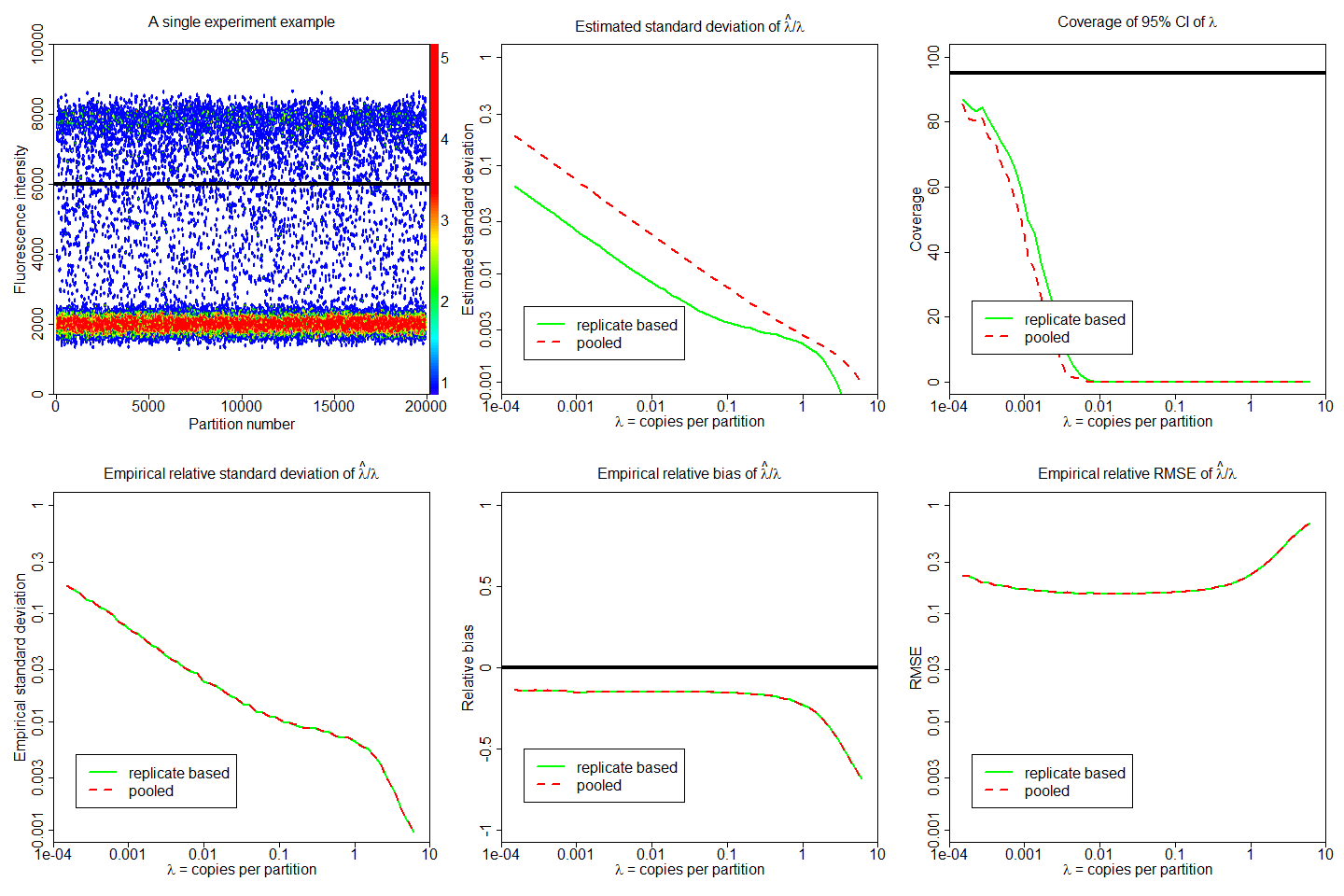

Supplement: Supplementary file 4 — Additional file 4: Interactive tool. In this mini-website, we provide an interactive tool to study the influence of specific sources of variation on the performance of the concentration estimators. This can serve as a guide when designing an experiment. All results are relative to the true concentration and based on 1000 simulations with 8 technical replicates. (ZIP 17 MB) [file 12859_2014_6687_MOESM4_ESM.zip › Additional file 4/RES/RES1333B.png]

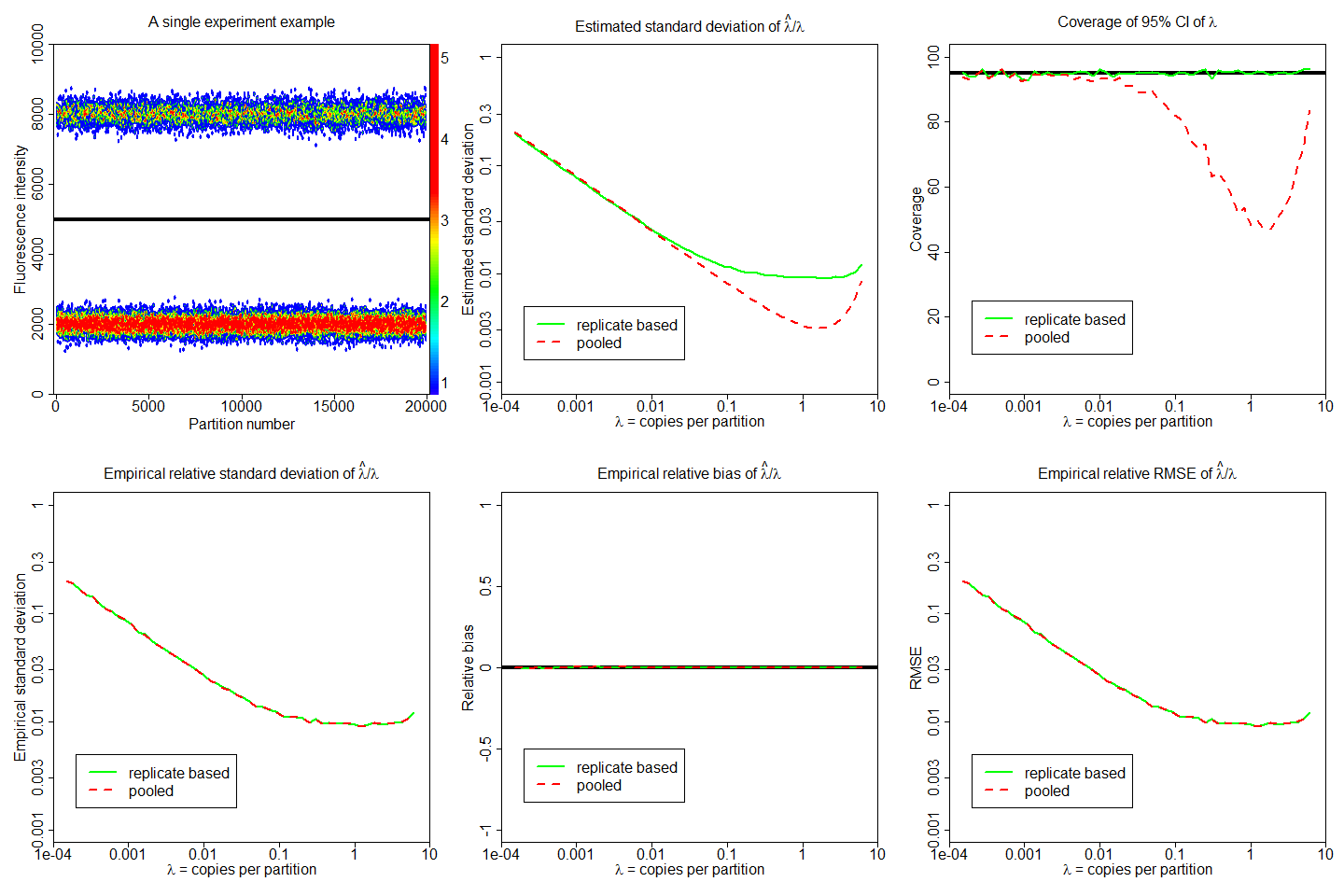

Supplement: Supplementary file 4 — Additional file 4: Interactive tool. In this mini-website, we provide an interactive tool to study the influence of specific sources of variation on the performance of the concentration estimators. This can serve as a guide when designing an experiment. All results are relative to the true concentration and based on 1000 simulations with 8 technical replicates. (ZIP 17 MB) [file 12859_2014_6687_MOESM4_ESM.zip › Additional file 4/RES/RES1341B.png]

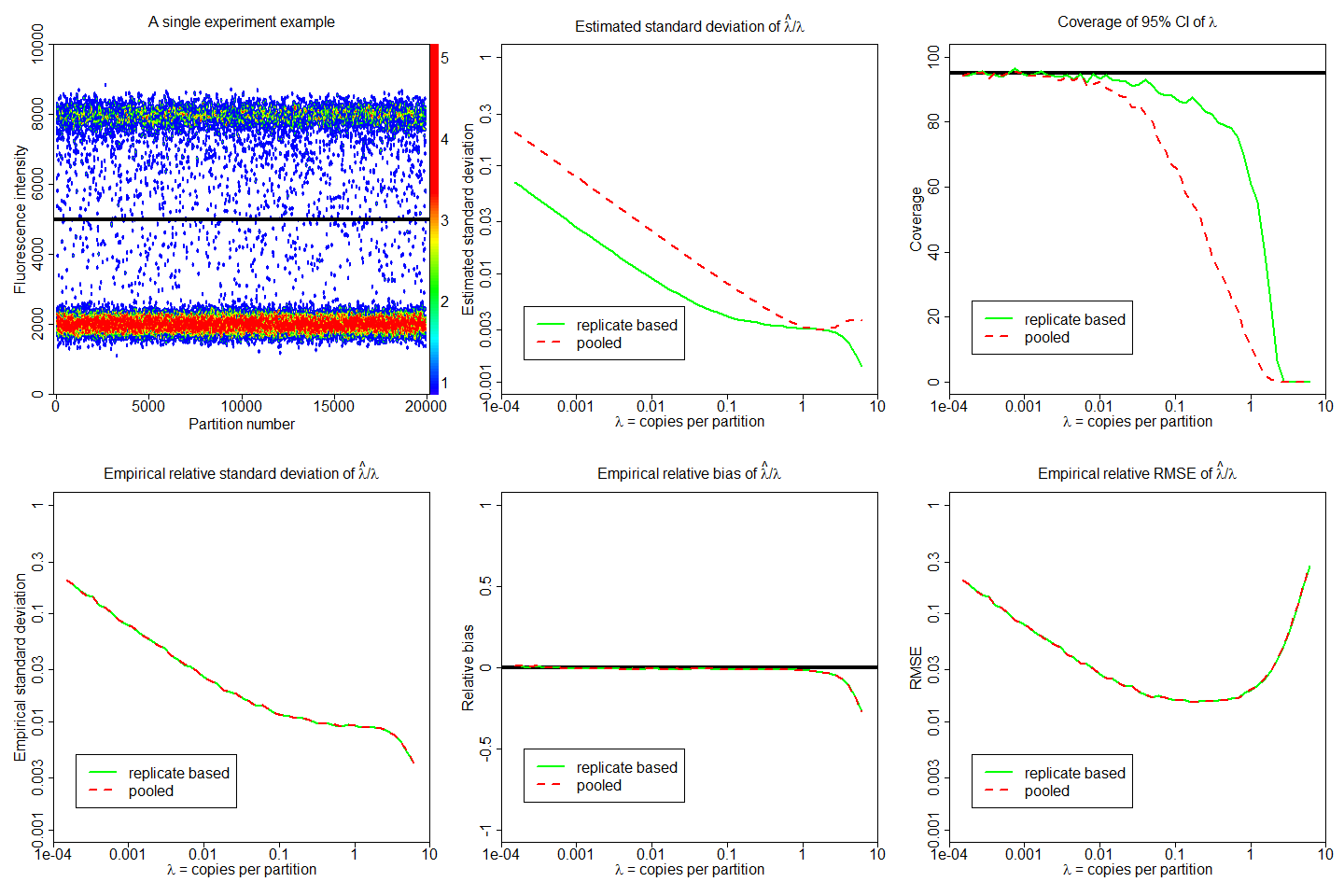

Supplement: Supplementary file 4 — Additional file 4: Interactive tool. In this mini-website, we provide an interactive tool to study the influence of specific sources of variation on the performance of the concentration estimators. This can serve as a guide when designing an experiment. All results are relative to the true concentration and based on 1000 simulations with 8 technical replicates. (ZIP 17 MB) [file 12859_2014_6687_MOESM4_ESM.zip › Additional file 4/RES/RES1342B.png]

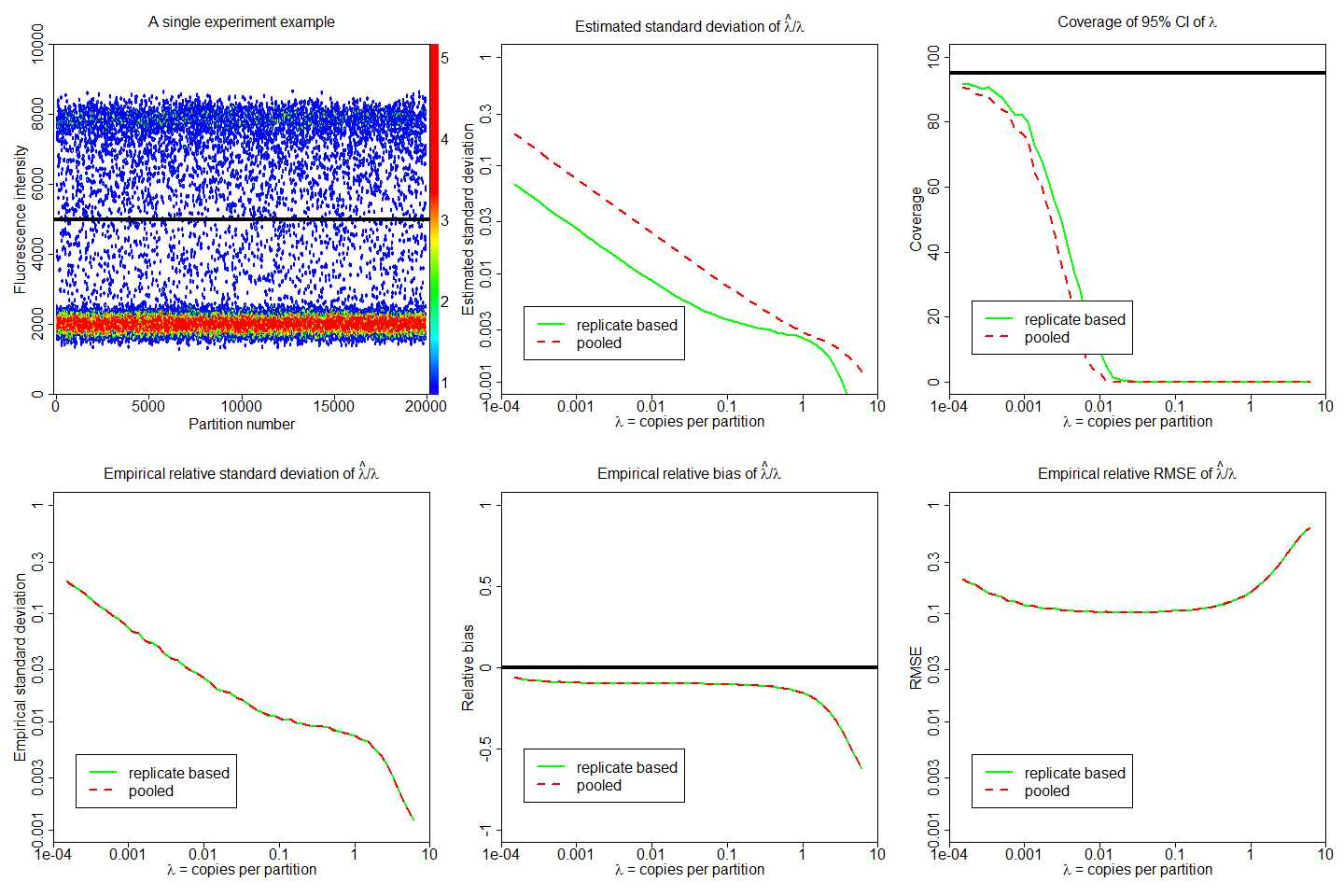

Supplement: Supplementary file 4 — Additional file 4: Interactive tool. In this mini-website, we provide an interactive tool to study the influence of specific sources of variation on the performance of the concentration estimators. This can serve as a guide when designing an experiment. All results are relative to the true concentration and based on 1000 simulations with 8 technical replicates. (ZIP 17 MB) [file 12859_2014_6687_MOESM4_ESM.zip › Additional file 4/RES/RES1343B.png]

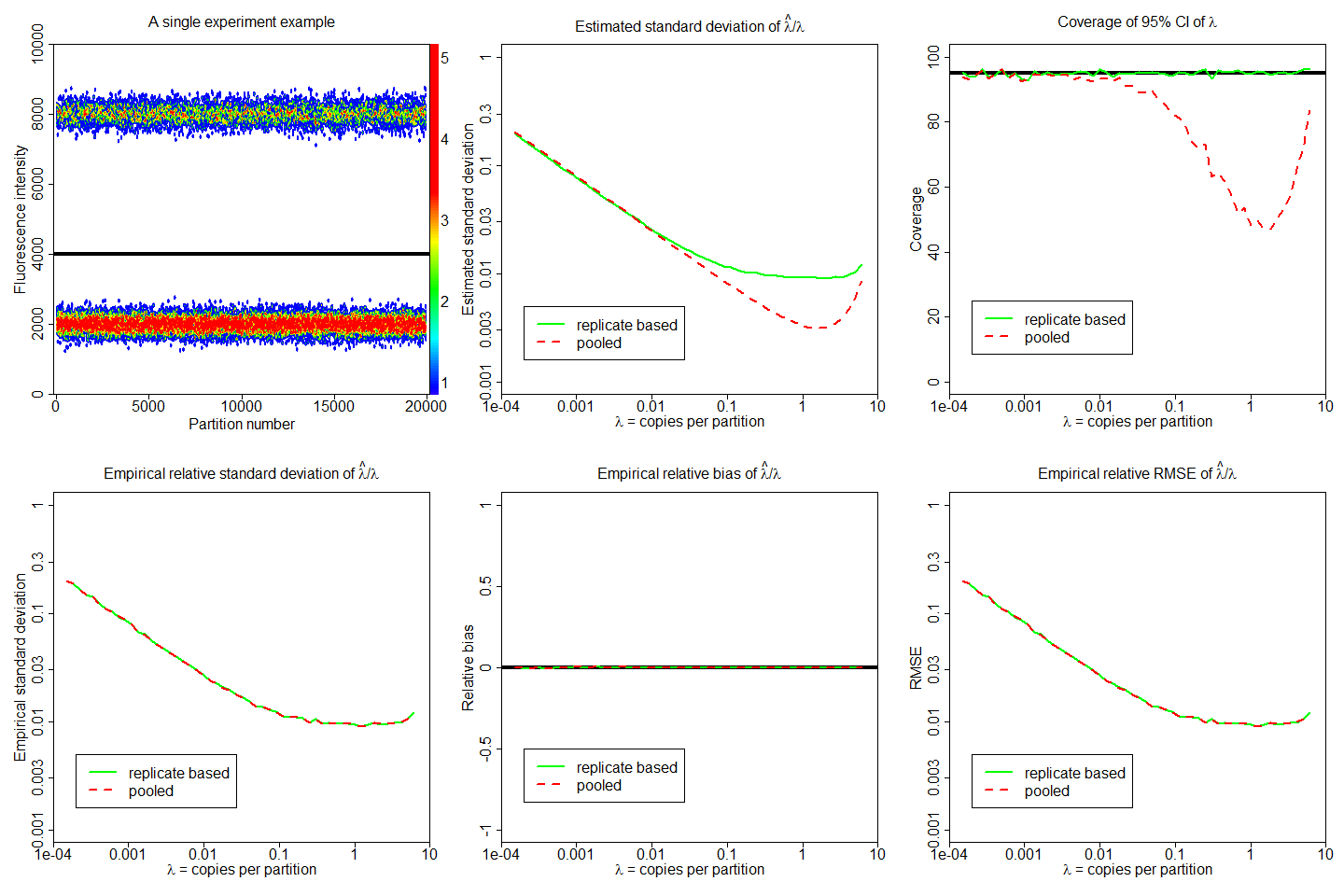

Supplement: Supplementary file 4 — Additional file 4: Interactive tool. In this mini-website, we provide an interactive tool to study the influence of specific sources of variation on the performance of the concentration estimators. This can serve as a guide when designing an experiment. All results are relative to the true concentration and based on 1000 simulations with 8 technical replicates. (ZIP 17 MB) [file 12859_2014_6687_MOESM4_ESM.zip › Additional file 4/RES/RES1351B.png]

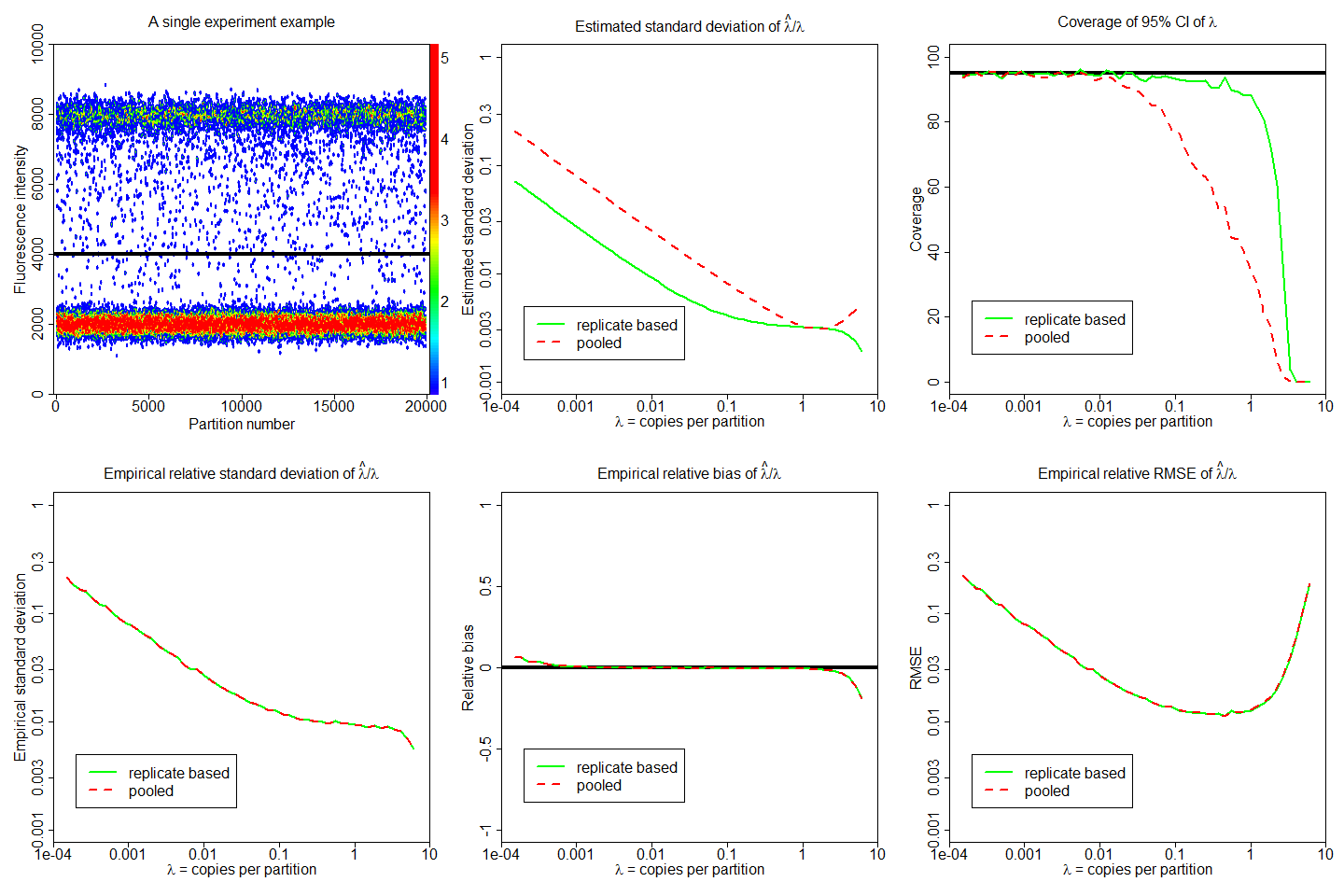

Supplement: Supplementary file 4 — Additional file 4: Interactive tool. In this mini-website, we provide an interactive tool to study the influence of specific sources of variation on the performance of the concentration estimators. This can serve as a guide when designing an experiment. All results are relative to the true concentration and based on 1000 simulations with 8 technical replicates. (ZIP 17 MB) [file 12859_2014_6687_MOESM4_ESM.zip › Additional file 4/RES/RES1352B.png]

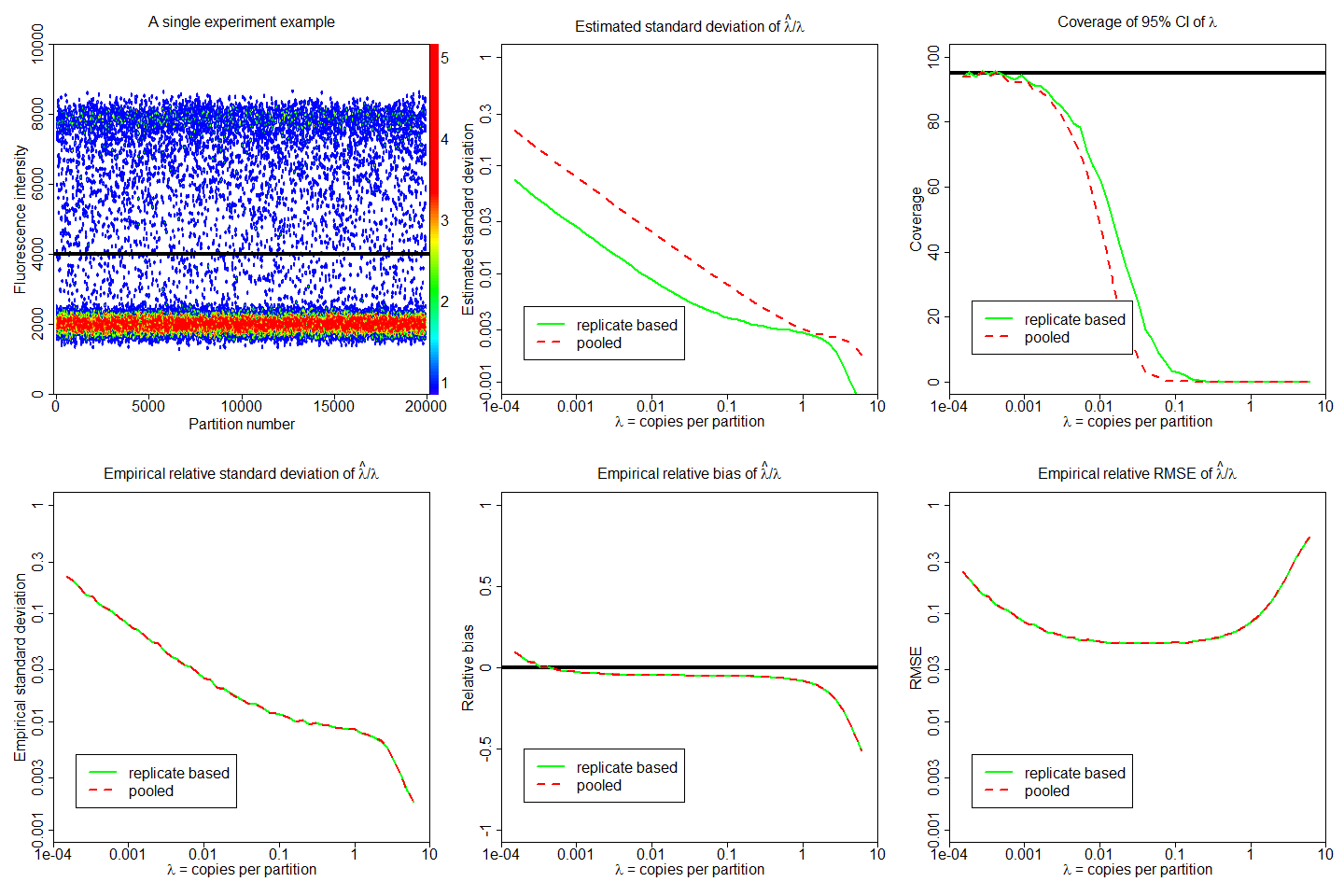

Supplement: Supplementary file 4 — Additional file 4: Interactive tool. In this mini-website, we provide an interactive tool to study the influence of specific sources of variation on the performance of the concentration estimators. This can serve as a guide when designing an experiment. All results are relative to the true concentration and based on 1000 simulations with 8 technical replicates. (ZIP 17 MB) [file 12859_2014_6687_MOESM4_ESM.zip › Additional file 4/RES/RES1353B.png]

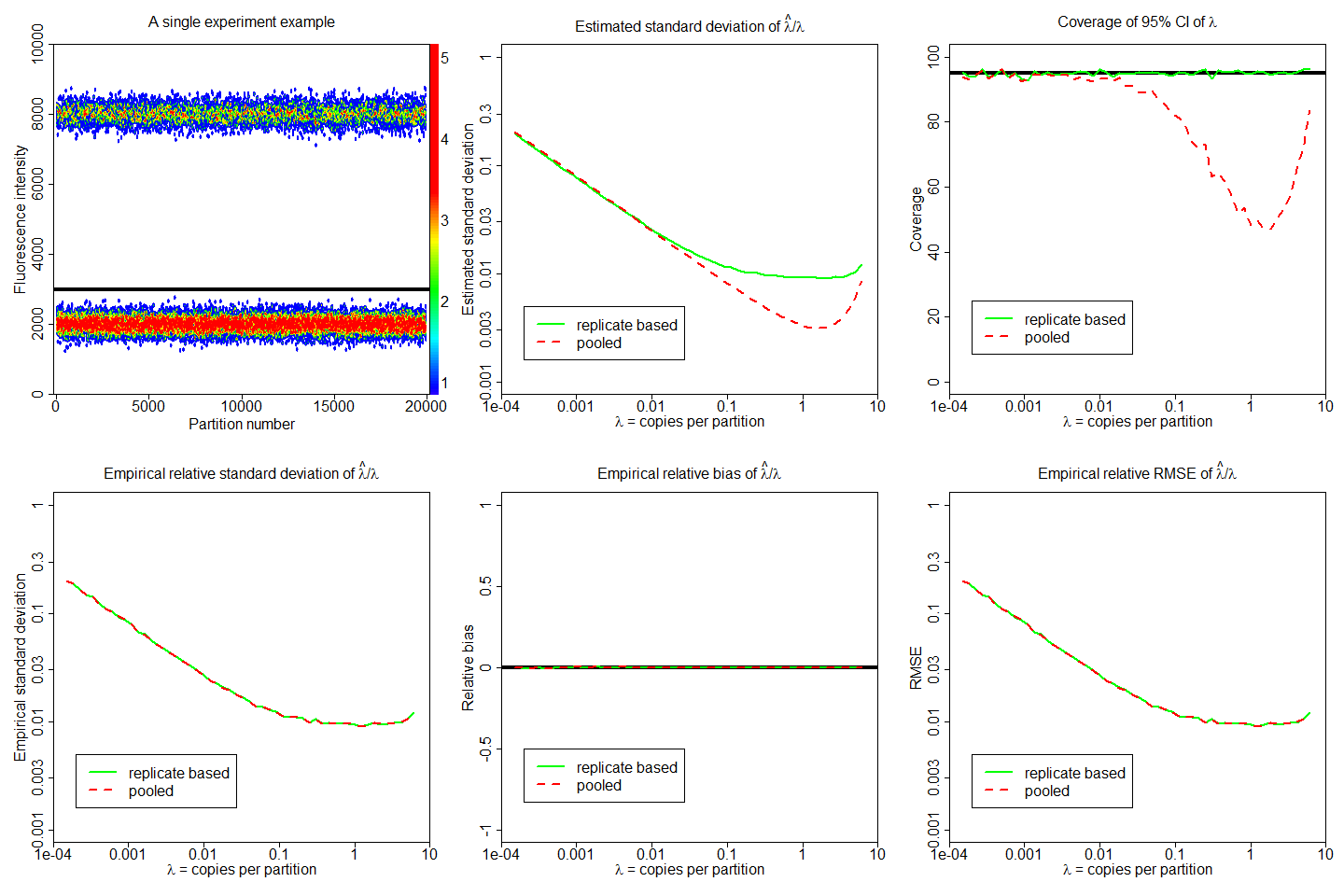

Supplement: Supplementary file 4 — Additional file 4: Interactive tool. In this mini-website, we provide an interactive tool to study the influence of specific sources of variation on the performance of the concentration estimators. This can serve as a guide when designing an experiment. All results are relative to the true concentration and based on 1000 simulations with 8 technical replicates. (ZIP 17 MB) [file 12859_2014_6687_MOESM4_ESM.zip › Additional file 4/RES/RES1361B.png]

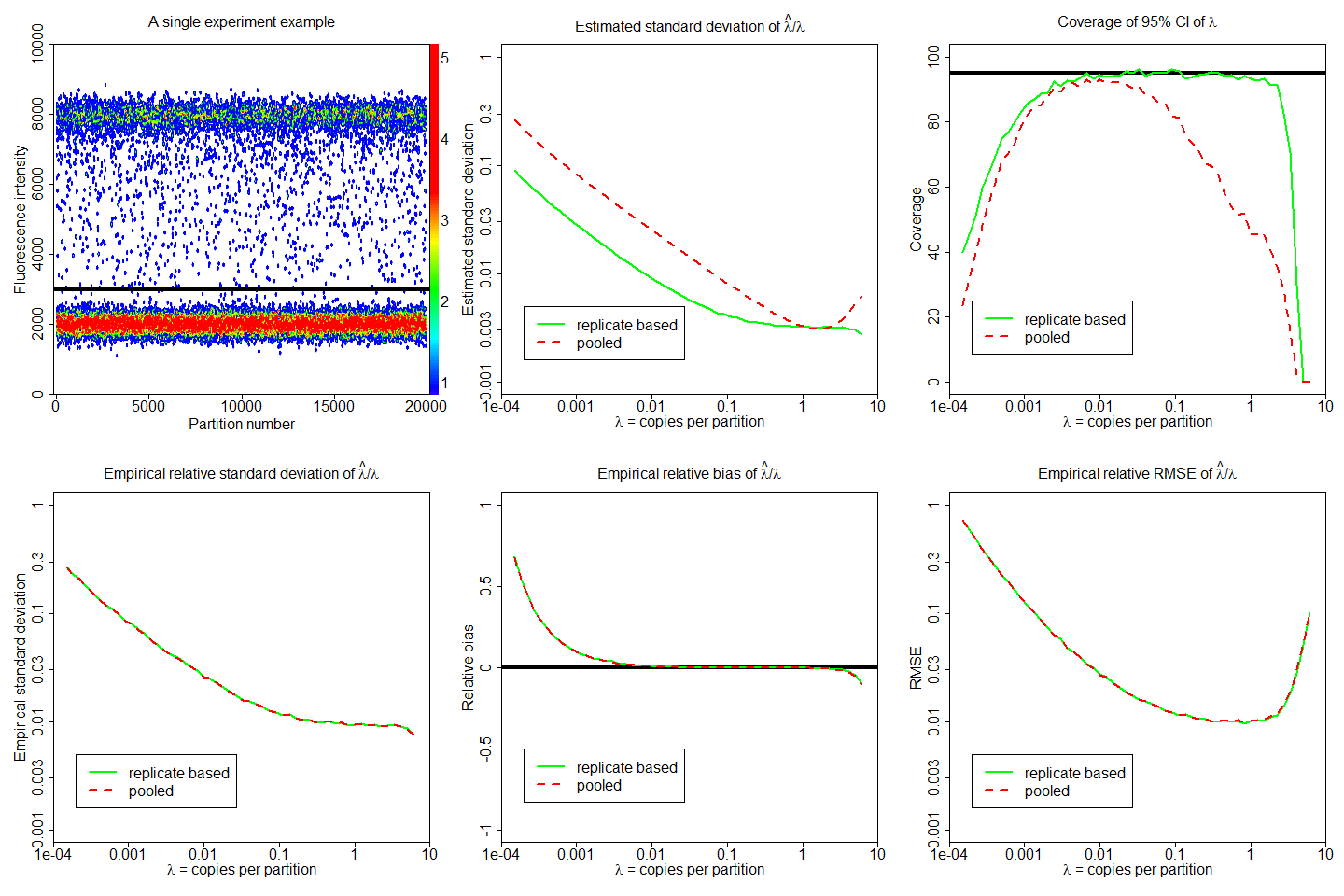

Supplement: Supplementary file 4 — Additional file 4: Interactive tool. In this mini-website, we provide an interactive tool to study the influence of specific sources of variation on the performance of the concentration estimators. This can serve as a guide when designing an experiment. All results are relative to the true concentration and based on 1000 simulations with 8 technical replicates. (ZIP 17 MB) [file 12859_2014_6687_MOESM4_ESM.zip › Additional file 4/RES/RES1362B.png]

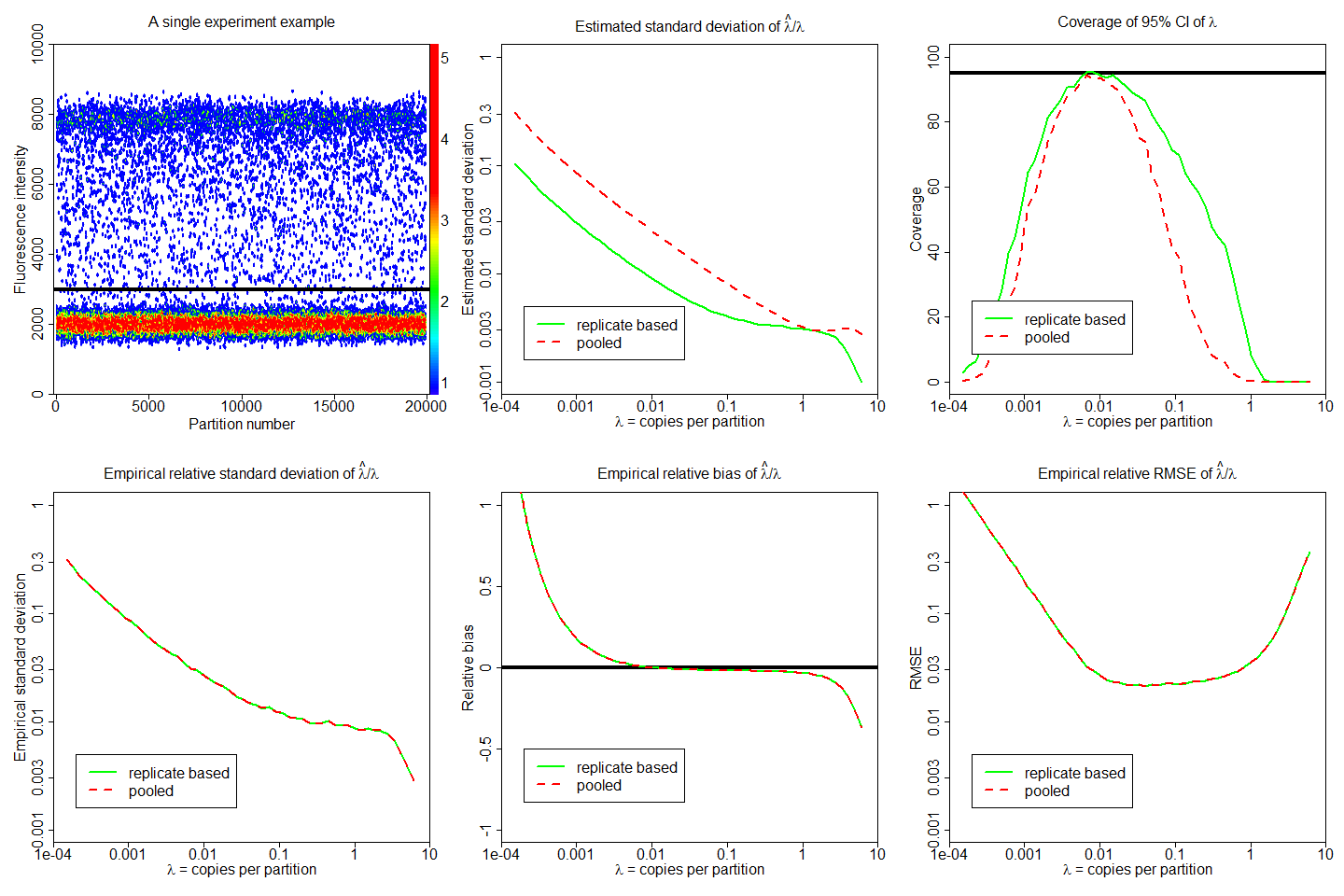

Supplement: Supplementary file 4 — Additional file 4: Interactive tool. In this mini-website, we provide an interactive tool to study the influence of specific sources of variation on the performance of the concentration estimators. This can serve as a guide when designing an experiment. All results are relative to the true concentration and based on 1000 simulations with 8 technical replicates. (ZIP 17 MB) [file 12859_2014_6687_MOESM4_ESM.zip › Additional file 4/RES/RES1363B.png]

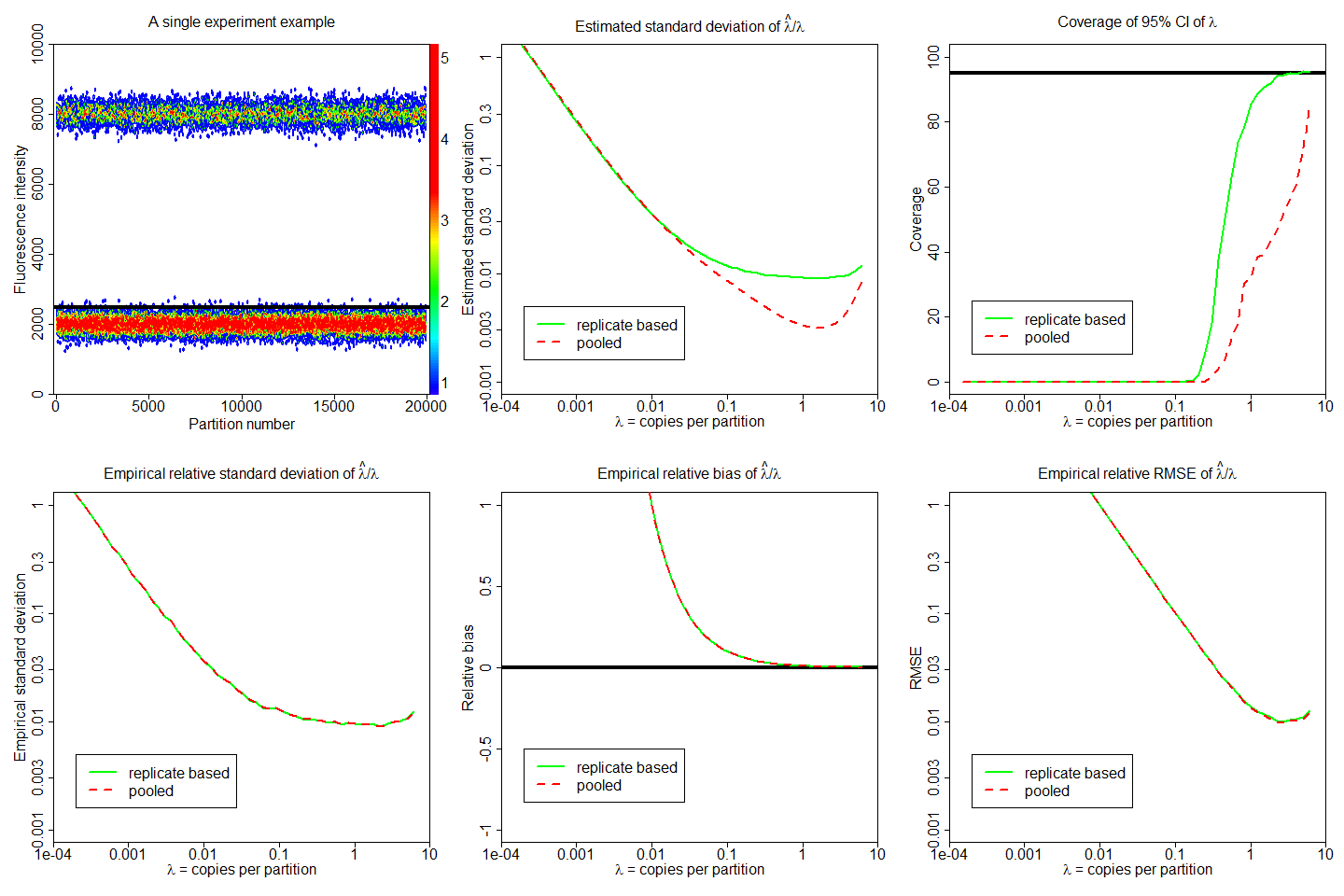

Supplement: Supplementary file 4 — Additional file 4: Interactive tool. In this mini-website, we provide an interactive tool to study the influence of specific sources of variation on the performance of the concentration estimators. This can serve as a guide when designing an experiment. All results are relative to the true concentration and based on 1000 simulations with 8 technical replicates. (ZIP 17 MB) [file 12859_2014_6687_MOESM4_ESM.zip › Additional file 4/RES/RES1371B.png]

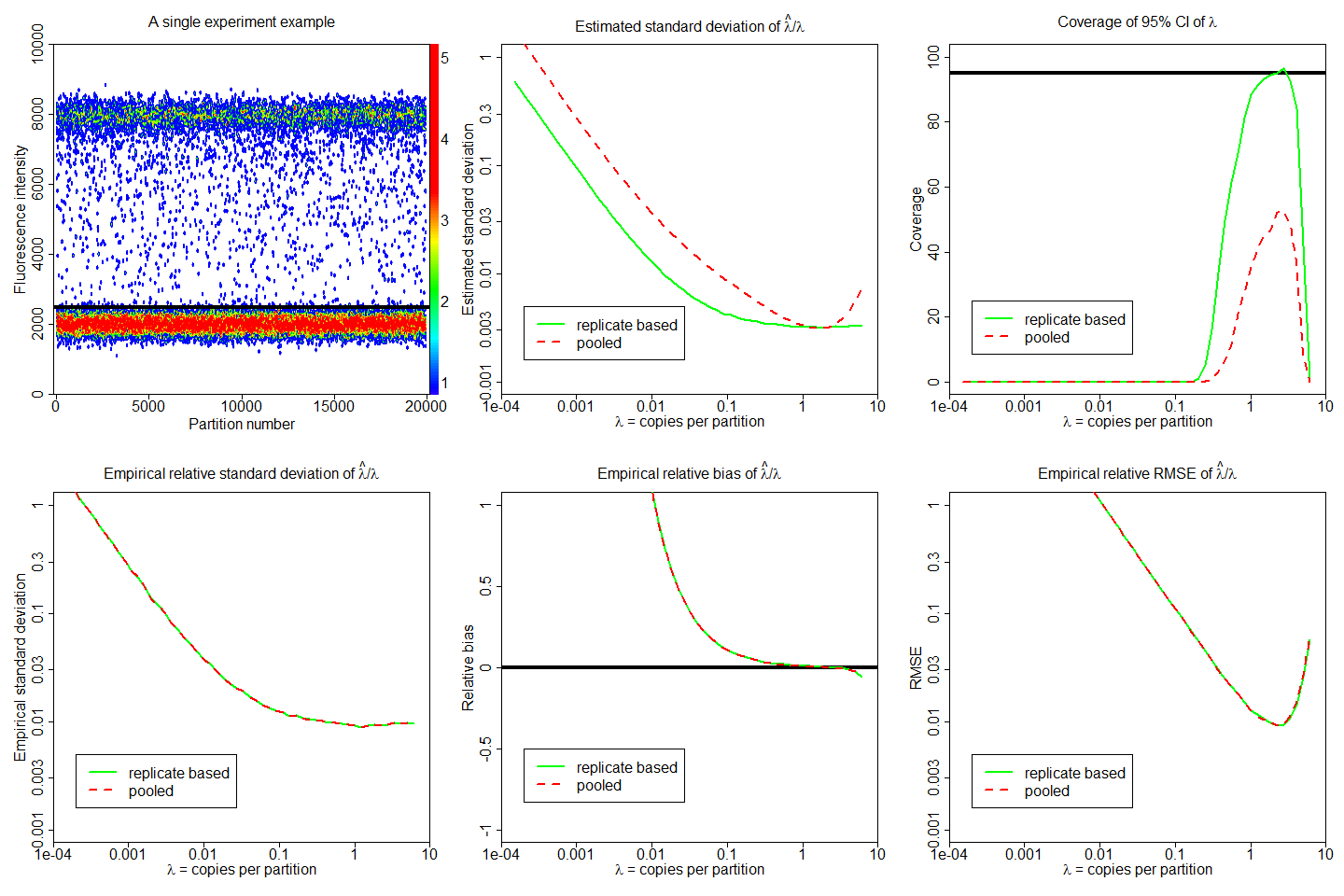

Supplement: Supplementary file 4 — Additional file 4: Interactive tool. In this mini-website, we provide an interactive tool to study the influence of specific sources of variation on the performance of the concentration estimators. This can serve as a guide when designing an experiment. All results are relative to the true concentration and based on 1000 simulations with 8 technical replicates. (ZIP 17 MB) [file 12859_2014_6687_MOESM4_ESM.zip › Additional file 4/RES/RES1372B.png]

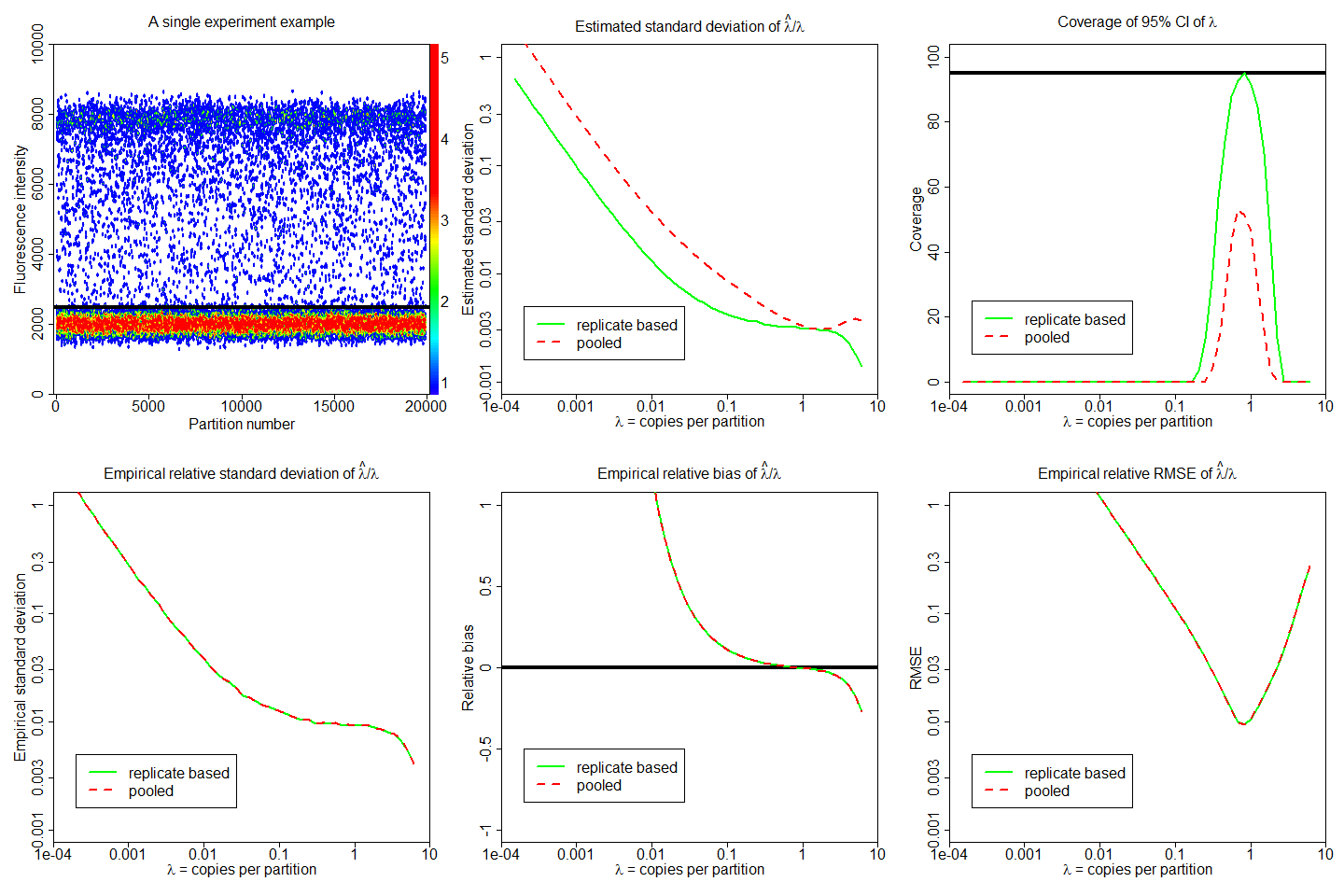

Supplement: Supplementary file 4 — Additional file 4: Interactive tool. In this mini-website, we provide an interactive tool to study the influence of specific sources of variation on the performance of the concentration estimators. This can serve as a guide when designing an experiment. All results are relative to the true concentration and based on 1000 simulations with 8 technical replicates. (ZIP 17 MB) [file 12859_2014_6687_MOESM4_ESM.zip › Additional file 4/RES/RES1373B.png]

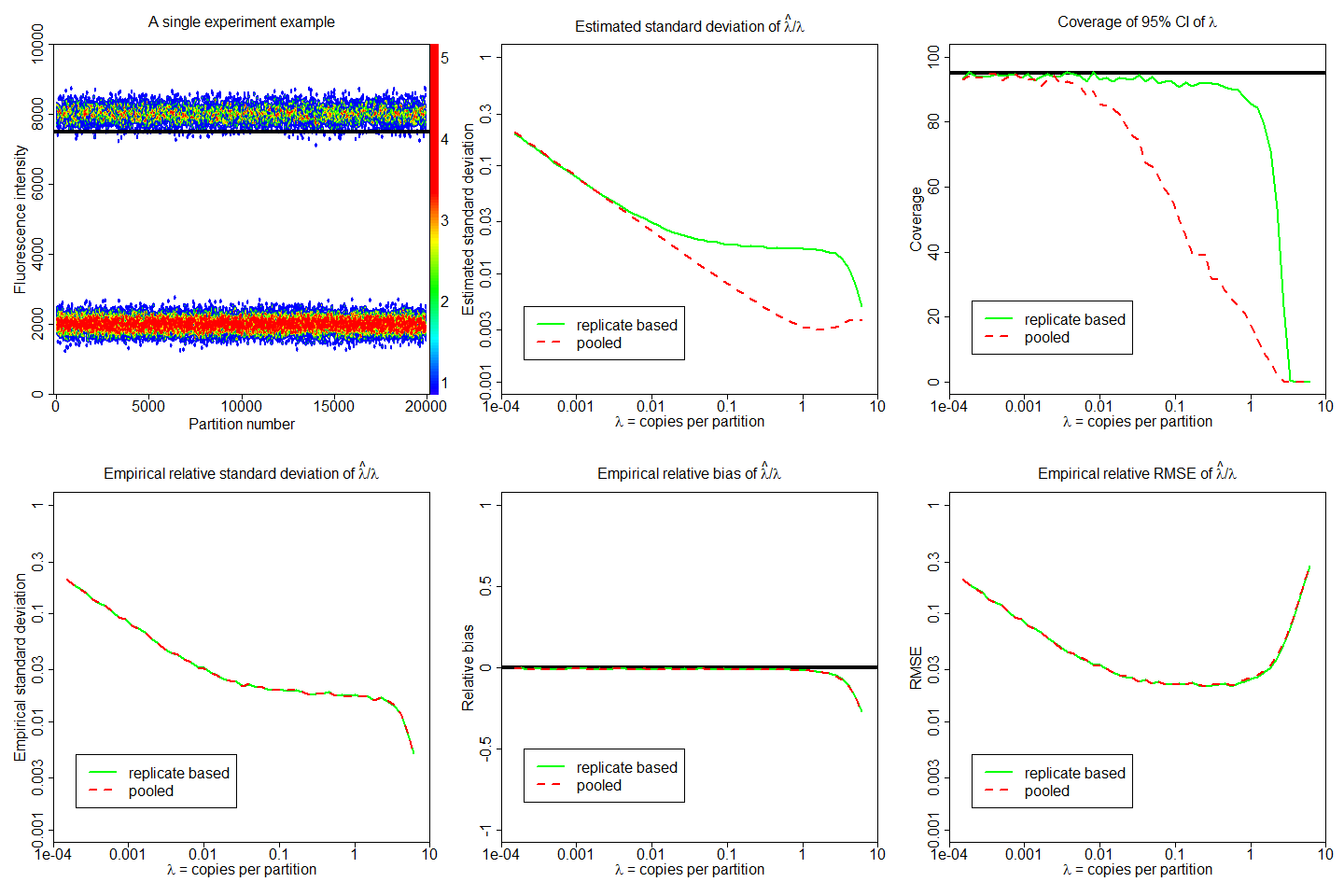

Supplement: Supplementary file 4 — Additional file 4: Interactive tool. In this mini-website, we provide an interactive tool to study the influence of specific sources of variation on the performance of the concentration estimators. This can serve as a guide when designing an experiment. All results are relative to the true concentration and based on 1000 simulations with 8 technical replicates. (ZIP 17 MB) [file 12859_2014_6687_MOESM4_ESM.zip › Additional file 4/RES/RES1411B.png]

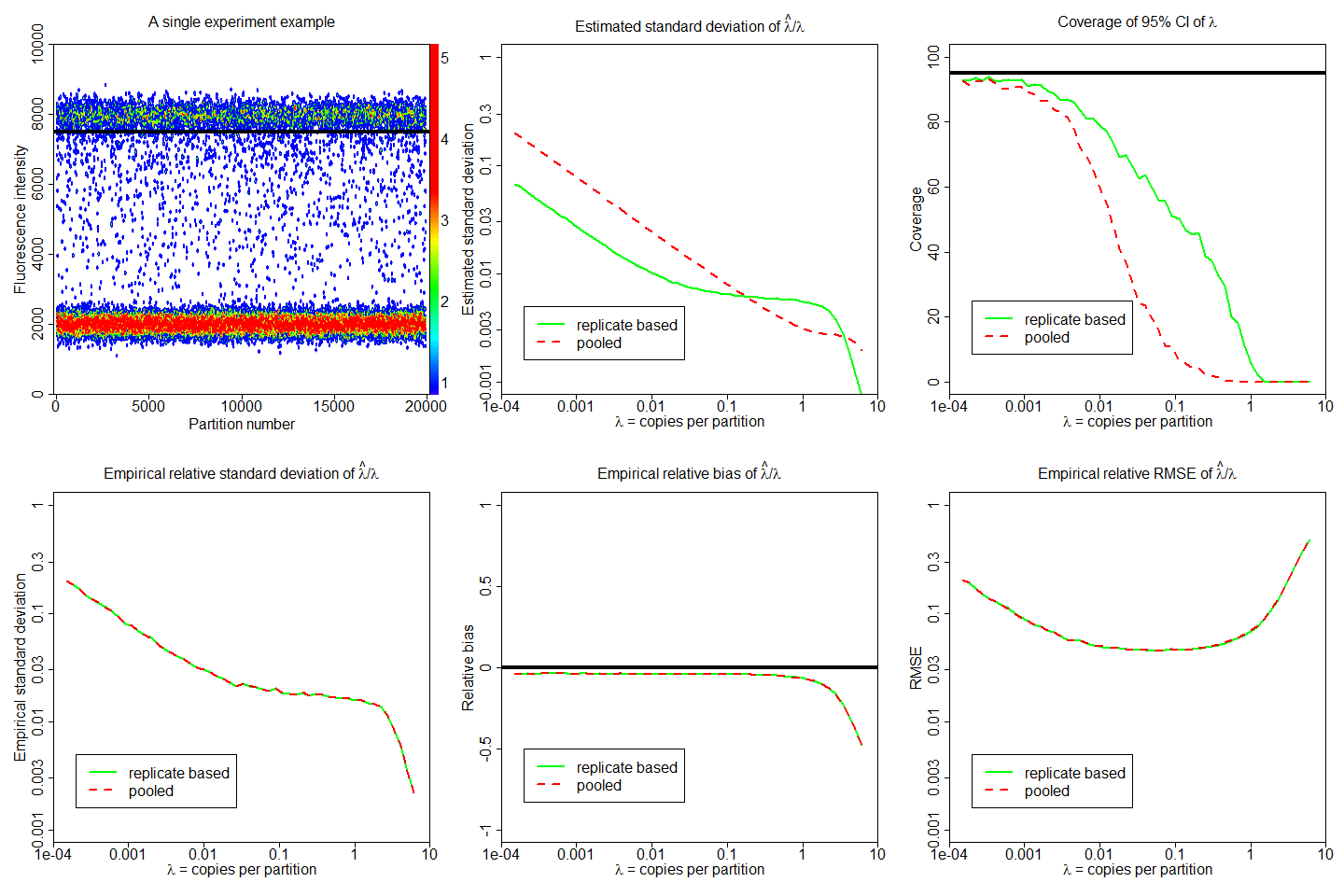

Supplement: Supplementary file 4 — Additional file 4: Interactive tool. In this mini-website, we provide an interactive tool to study the influence of specific sources of variation on the performance of the concentration estimators. This can serve as a guide when designing an experiment. All results are relative to the true concentration and based on 1000 simulations with 8 technical replicates. (ZIP 17 MB) [file 12859_2014_6687_MOESM4_ESM.zip › Additional file 4/RES/RES1412B.png]

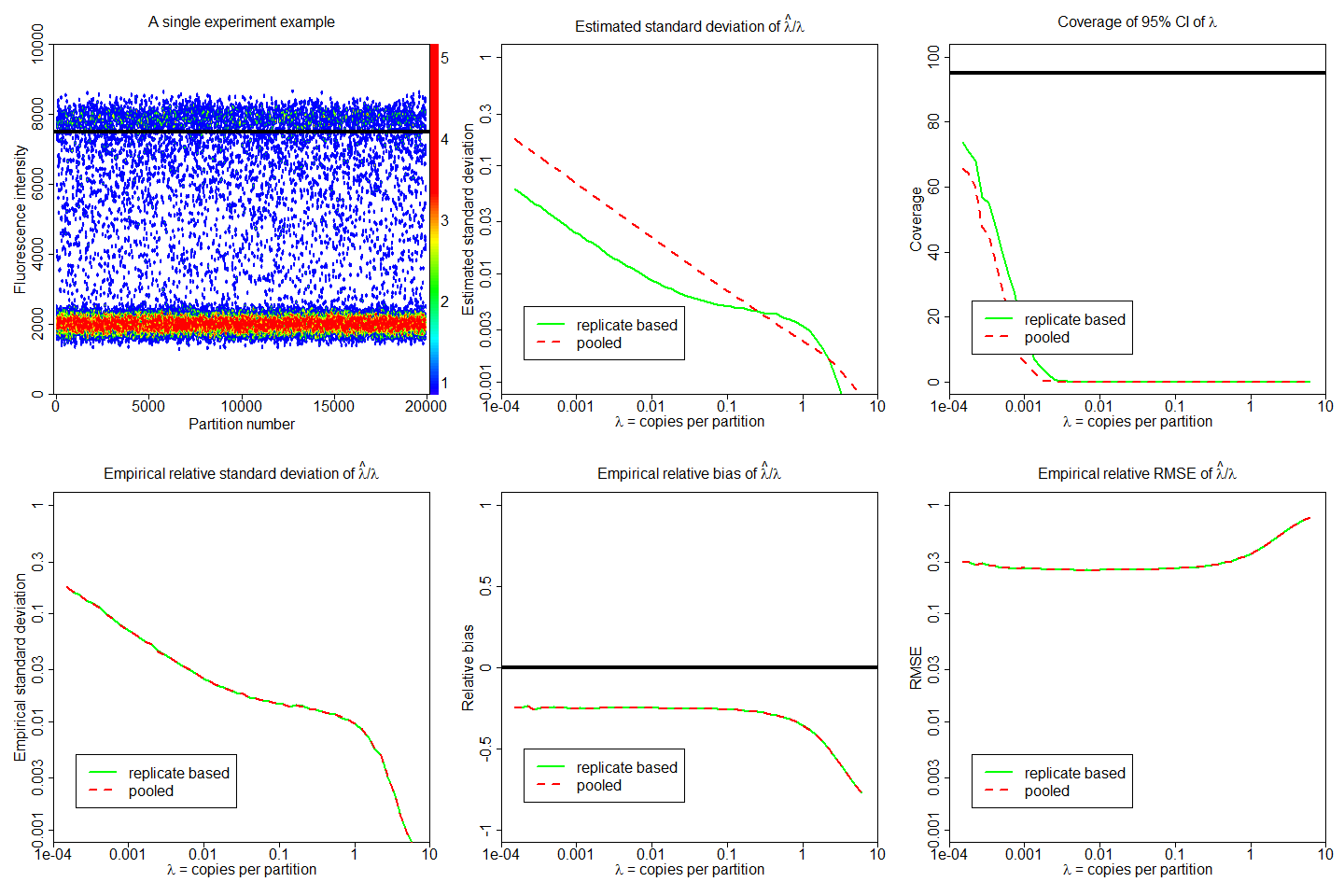

Supplement: Supplementary file 4 — Additional file 4: Interactive tool. In this mini-website, we provide an interactive tool to study the influence of specific sources of variation on the performance of the concentration estimators. This can serve as a guide when designing an experiment. All results are relative to the true concentration and based on 1000 simulations with 8 technical replicates. (ZIP 17 MB) [file 12859_2014_6687_MOESM4_ESM.zip › Additional file 4/RES/RES1413B.png]

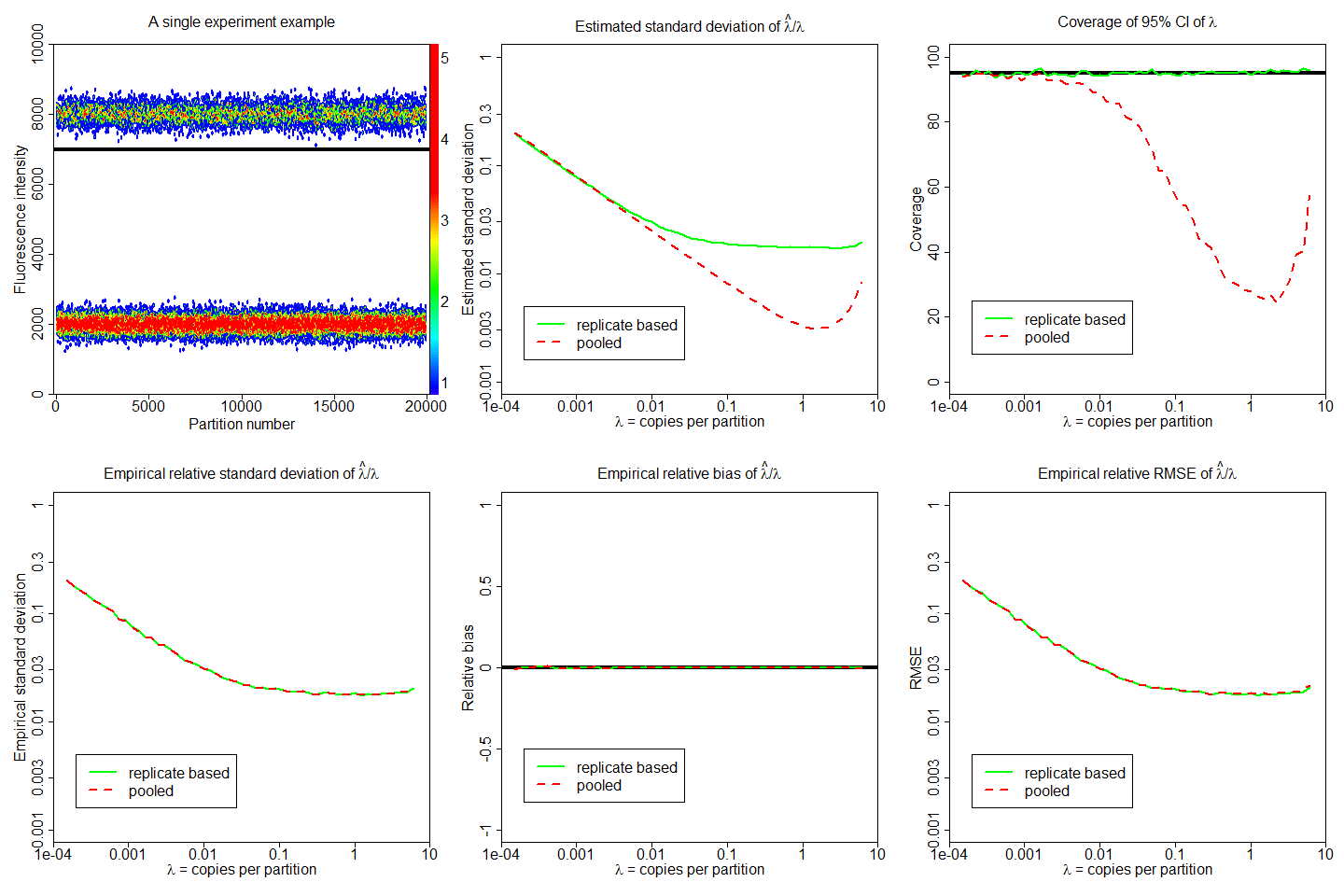

Supplement: Supplementary file 4 — Additional file 4: Interactive tool. In this mini-website, we provide an interactive tool to study the influence of specific sources of variation on the performance of the concentration estimators. This can serve as a guide when designing an experiment. All results are relative to the true concentration and based on 1000 simulations with 8 technical replicates. (ZIP 17 MB) [file 12859_2014_6687_MOESM4_ESM.zip › Additional file 4/RES/RES1421B.png]

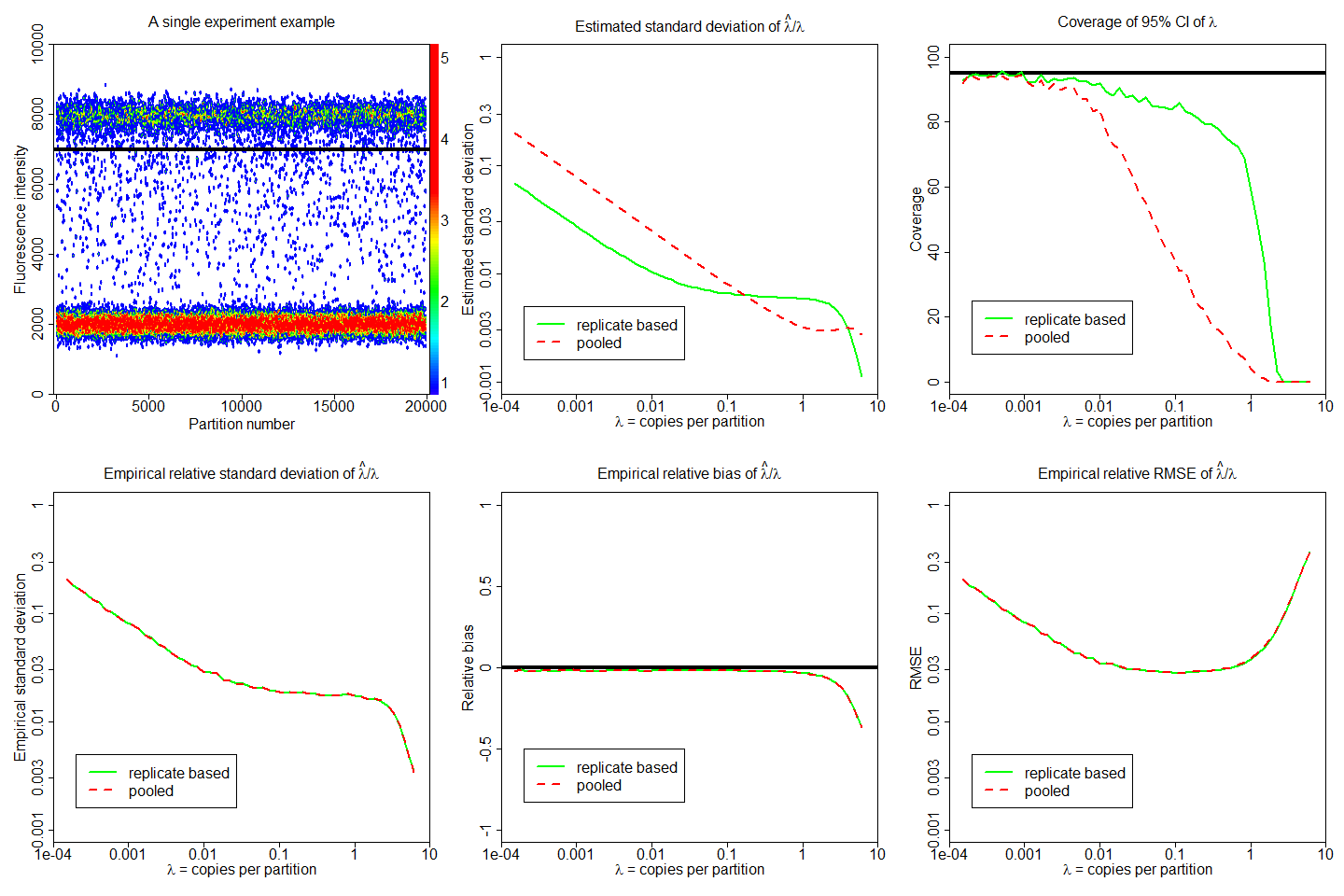

Supplement: Supplementary file 4 — Additional file 4: Interactive tool. In this mini-website, we provide an interactive tool to study the influence of specific sources of variation on the performance of the concentration estimators. This can serve as a guide when designing an experiment. All results are relative to the true concentration and based on 1000 simulations with 8 technical replicates. (ZIP 17 MB) [file 12859_2014_6687_MOESM4_ESM.zip › Additional file 4/RES/RES1422B.png]

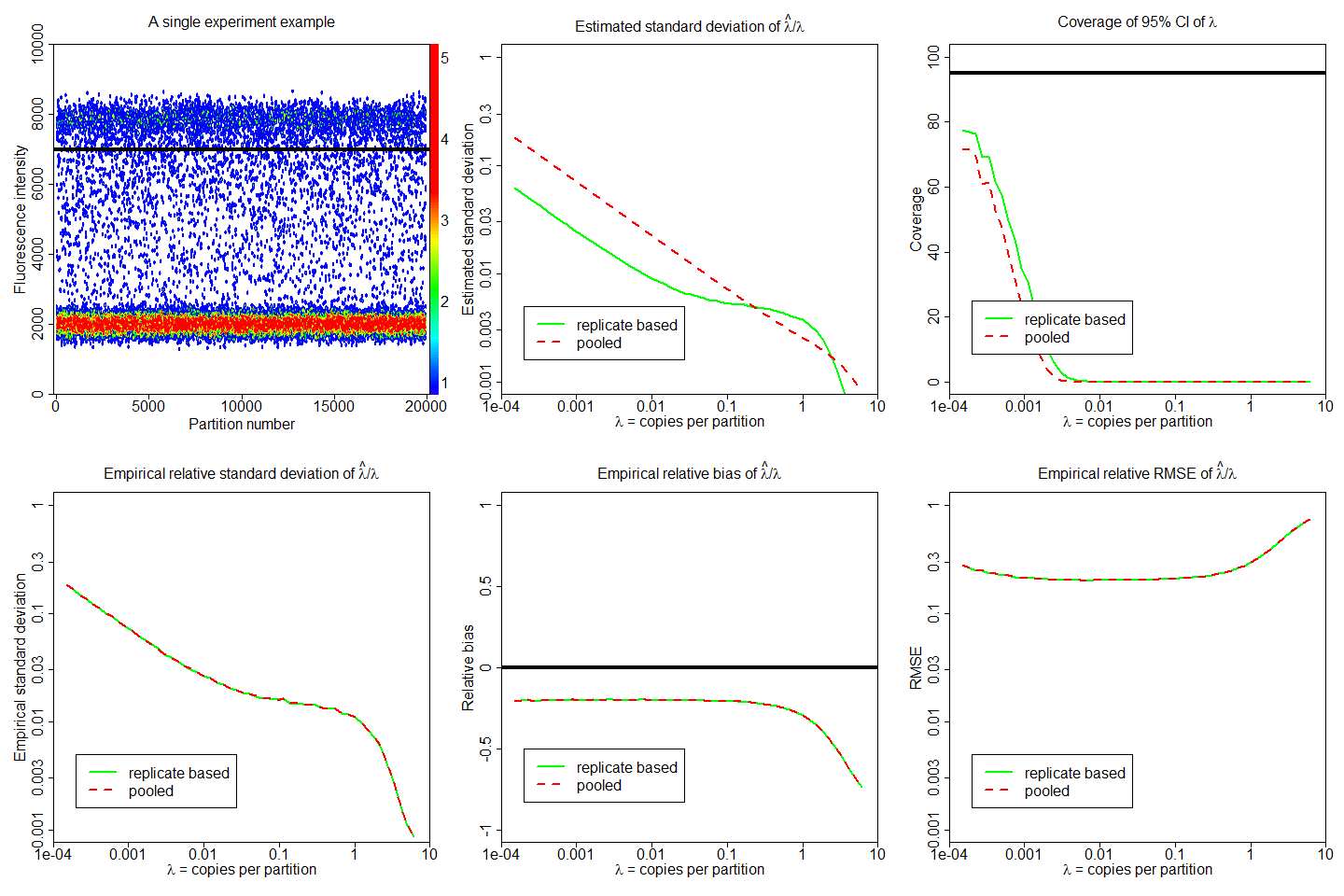

Supplement: Supplementary file 4 — Additional file 4: Interactive tool. In this mini-website, we provide an interactive tool to study the influence of specific sources of variation on the performance of the concentration estimators. This can serve as a guide when designing an experiment. All results are relative to the true concentration and based on 1000 simulations with 8 technical replicates. (ZIP 17 MB) [file 12859_2014_6687_MOESM4_ESM.zip › Additional file 4/RES/RES1423B.png]

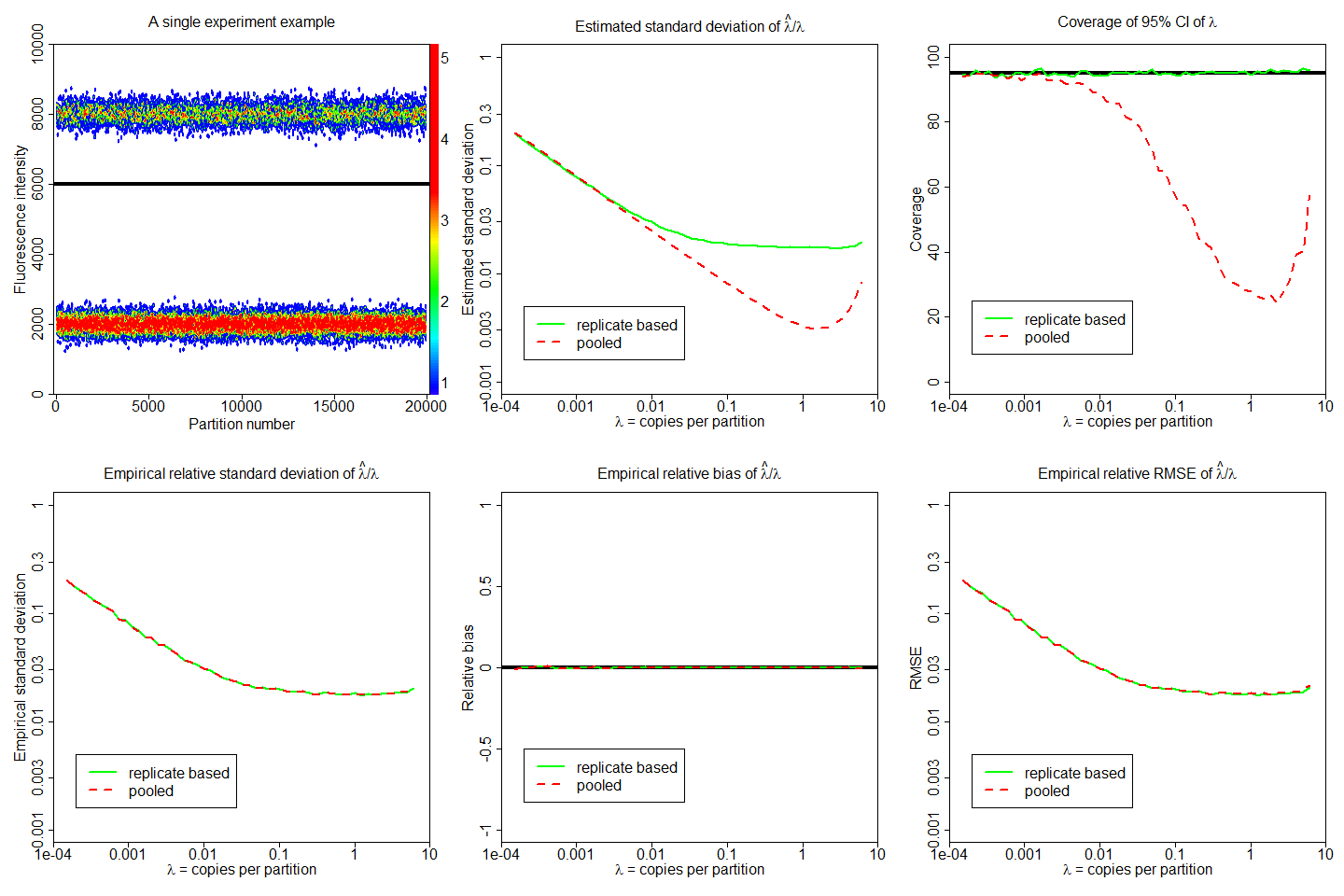

Supplement: Supplementary file 4 — Additional file 4: Interactive tool. In this mini-website, we provide an interactive tool to study the influence of specific sources of variation on the performance of the concentration estimators. This can serve as a guide when designing an experiment. All results are relative to the true concentration and based on 1000 simulations with 8 technical replicates. (ZIP 17 MB) [file 12859_2014_6687_MOESM4_ESM.zip › Additional file 4/RES/RES1431B.png]

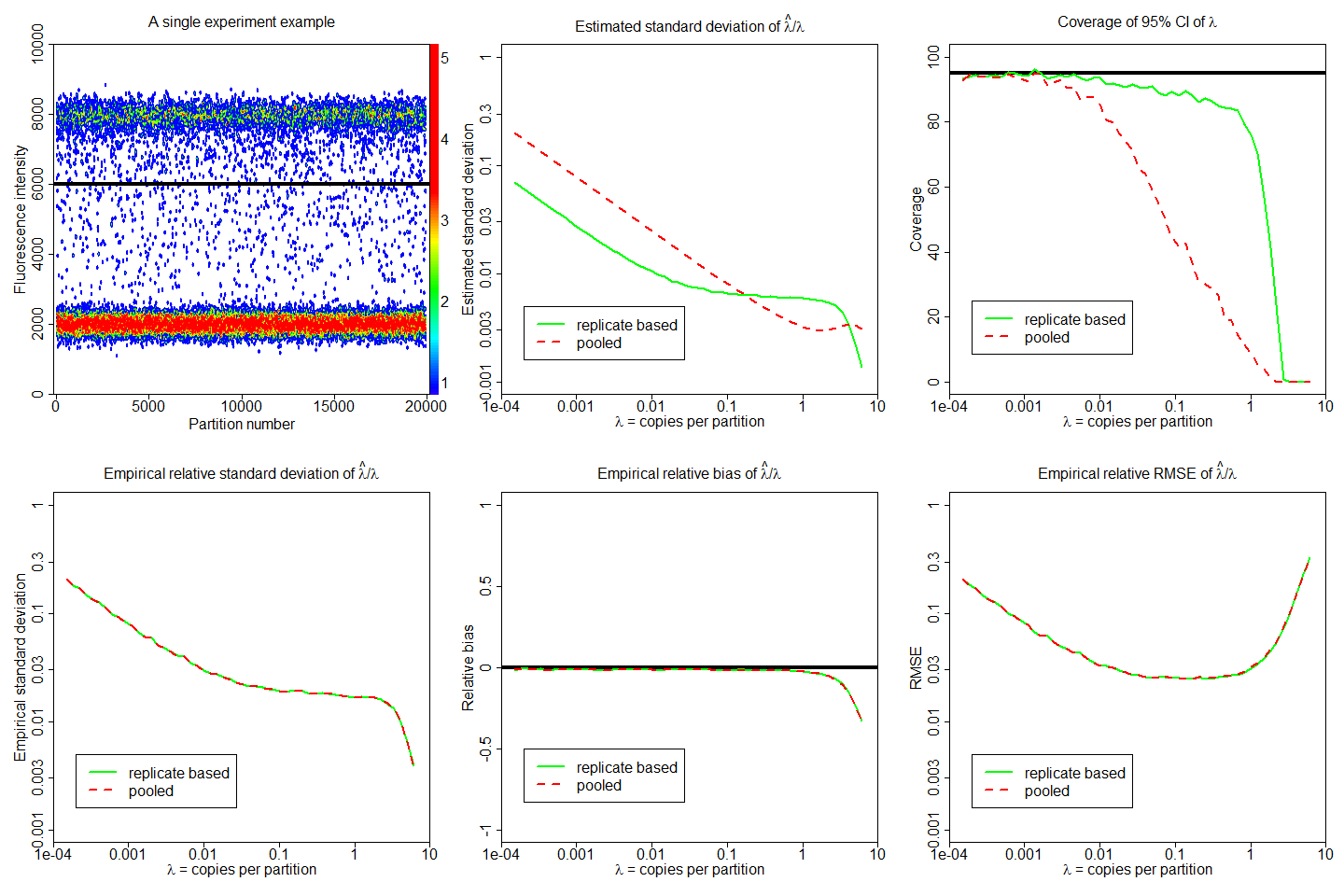

Supplement: Supplementary file 4 — Additional file 4: Interactive tool. In this mini-website, we provide an interactive tool to study the influence of specific sources of variation on the performance of the concentration estimators. This can serve as a guide when designing an experiment. All results are relative to the true concentration and based on 1000 simulations with 8 technical replicates. (ZIP 17 MB) [file 12859_2014_6687_MOESM4_ESM.zip › Additional file 4/RES/RES1432B.png]

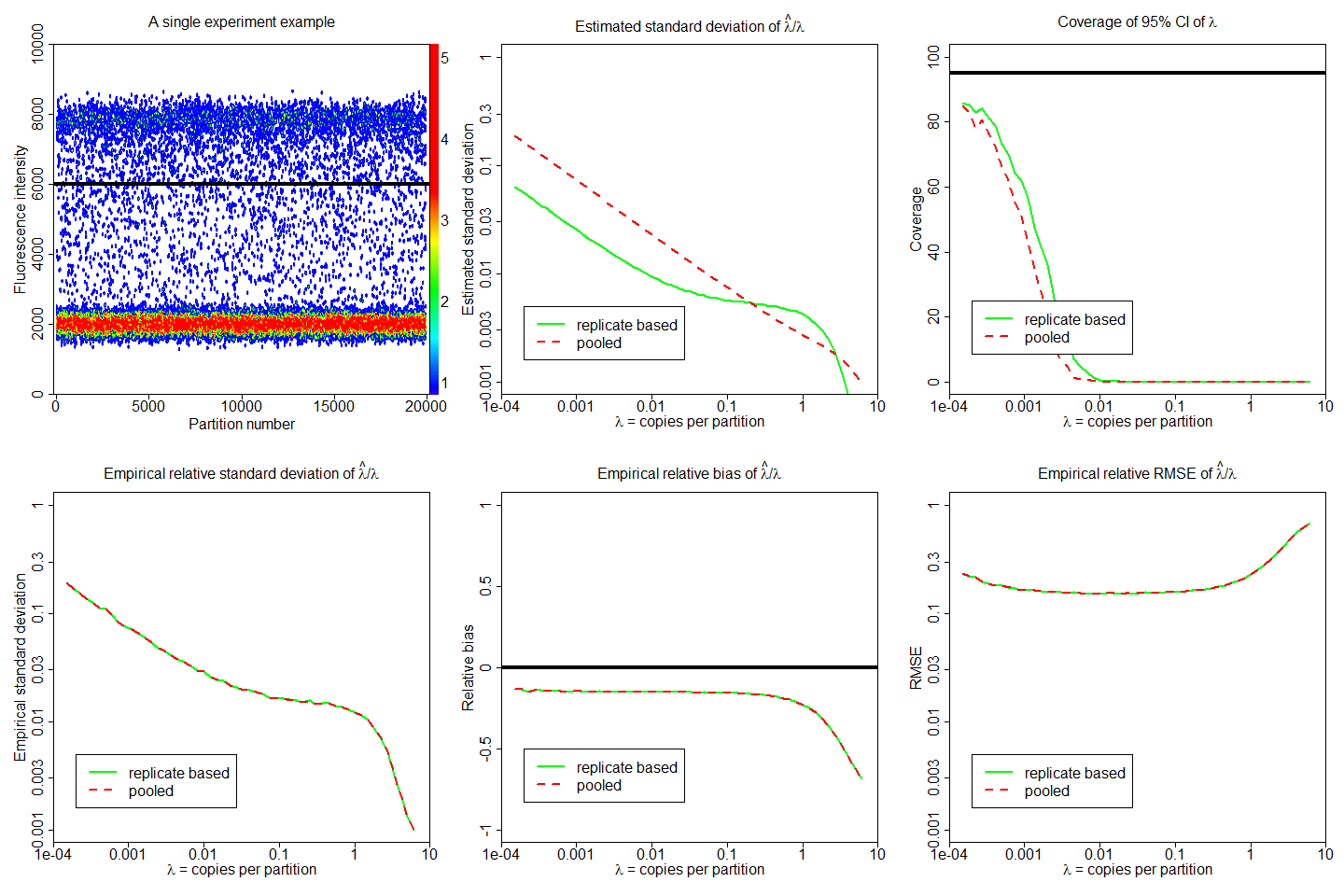

Supplement: Supplementary file 4 — Additional file 4: Interactive tool. In this mini-website, we provide an interactive tool to study the influence of specific sources of variation on the performance of the concentration estimators. This can serve as a guide when designing an experiment. All results are relative to the true concentration and based on 1000 simulations with 8 technical replicates. (ZIP 17 MB) [file 12859_2014_6687_MOESM4_ESM.zip › Additional file 4/RES/RES1433B.png]

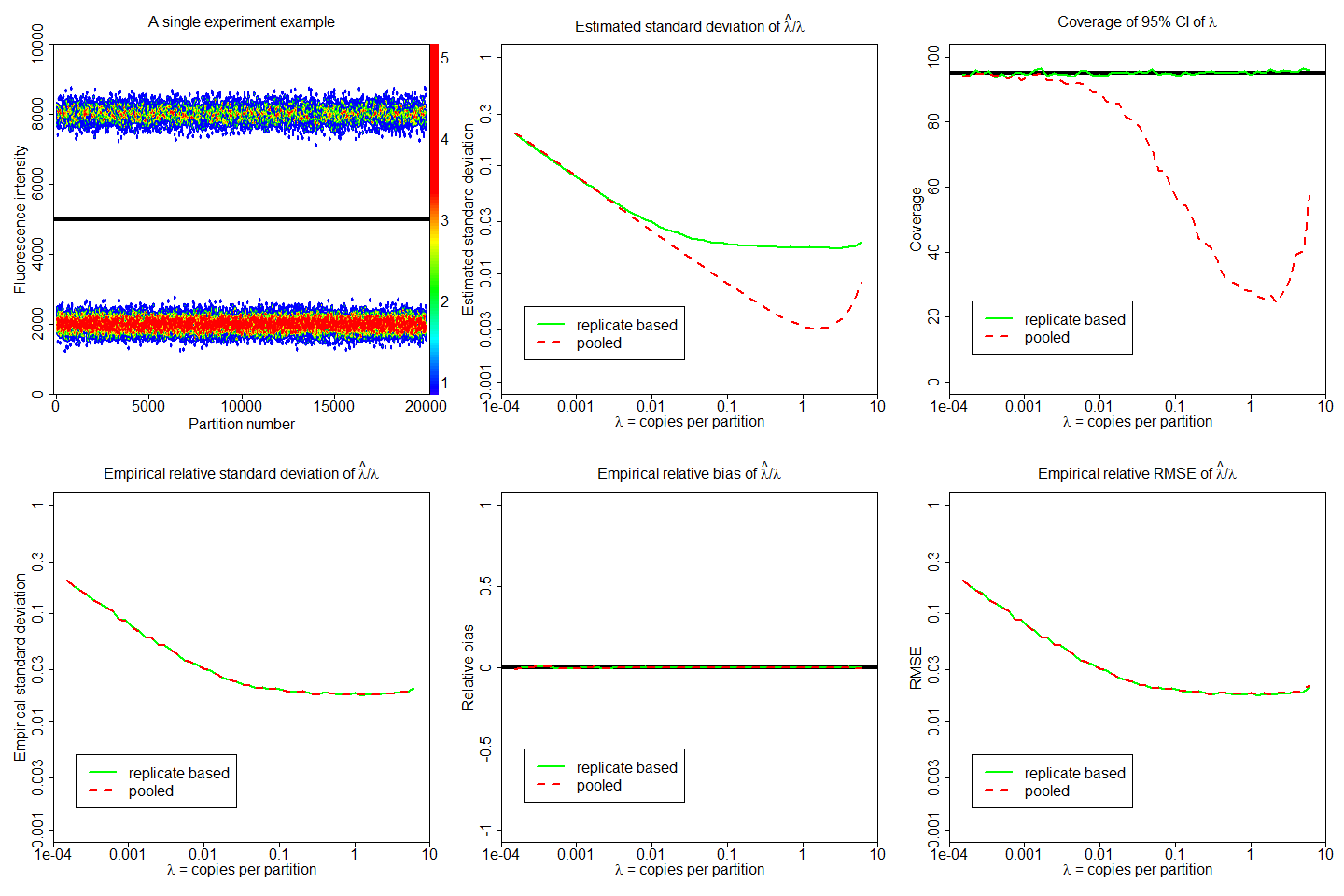

Supplement: Supplementary file 4 — Additional file 4: Interactive tool. In this mini-website, we provide an interactive tool to study the influence of specific sources of variation on the performance of the concentration estimators. This can serve as a guide when designing an experiment. All results are relative to the true concentration and based on 1000 simulations with 8 technical replicates. (ZIP 17 MB) [file 12859_2014_6687_MOESM4_ESM.zip › Additional file 4/RES/RES1441B.png]

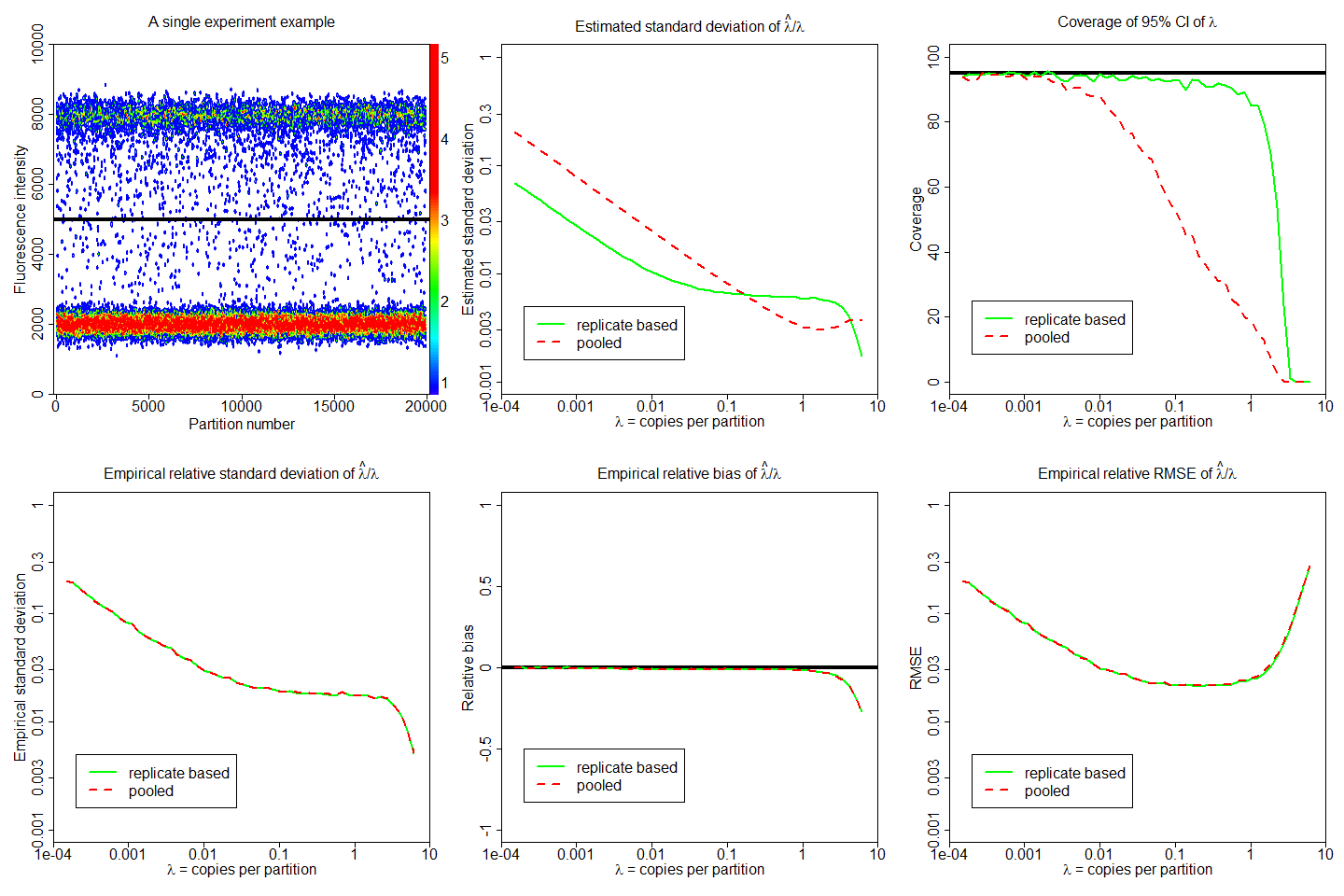

Supplement: Supplementary file 4 — Additional file 4: Interactive tool. In this mini-website, we provide an interactive tool to study the influence of specific sources of variation on the performance of the concentration estimators. This can serve as a guide when designing an experiment. All results are relative to the true concentration and based on 1000 simulations with 8 technical replicates. (ZIP 17 MB) [file 12859_2014_6687_MOESM4_ESM.zip › Additional file 4/RES/RES1442B.png]

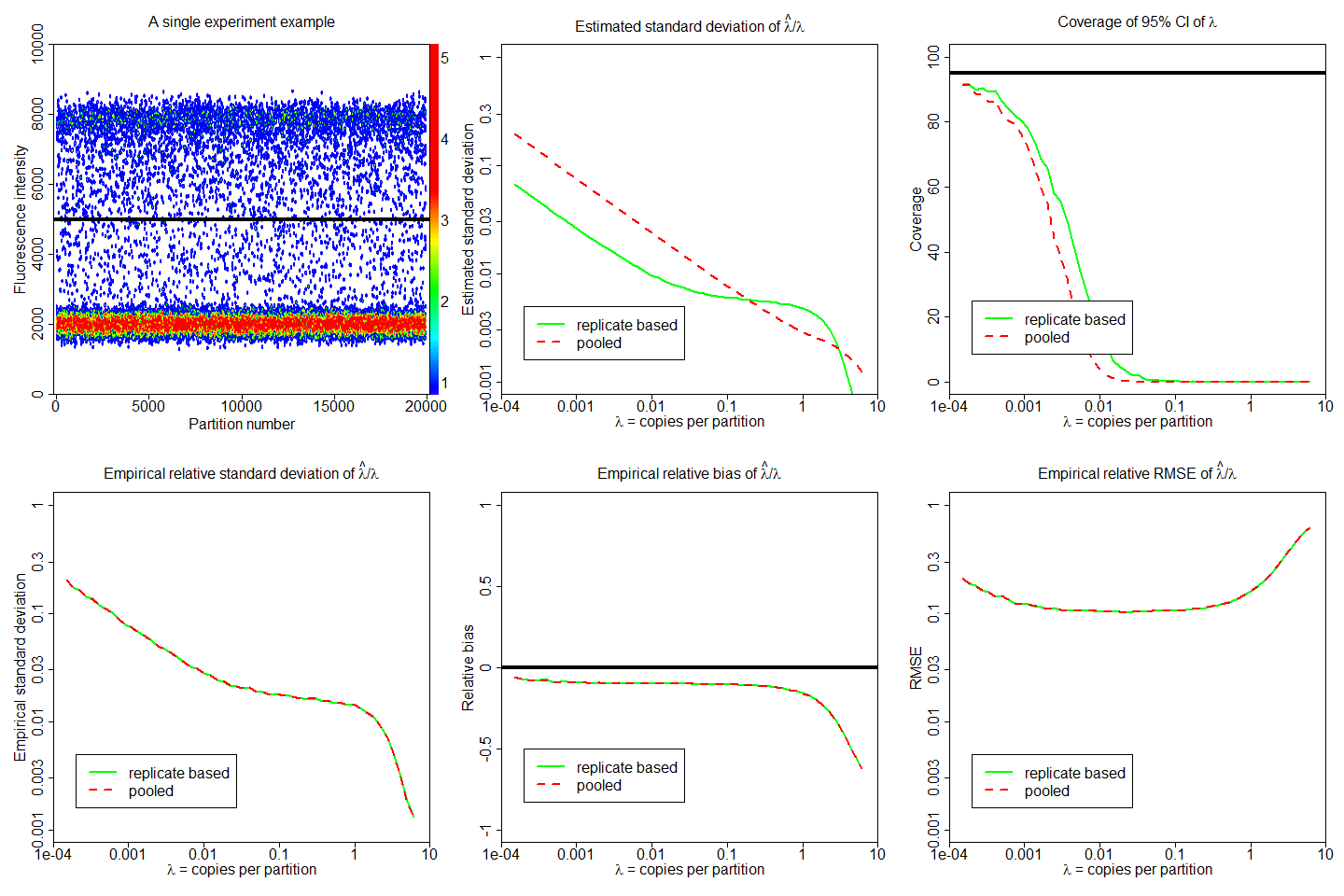

Supplement: Supplementary file 4 — Additional file 4: Interactive tool. In this mini-website, we provide an interactive tool to study the influence of specific sources of variation on the performance of the concentration estimators. This can serve as a guide when designing an experiment. All results are relative to the true concentration and based on 1000 simulations with 8 technical replicates. (ZIP 17 MB) [file 12859_2014_6687_MOESM4_ESM.zip › Additional file 4/RES/RES1443B.png]

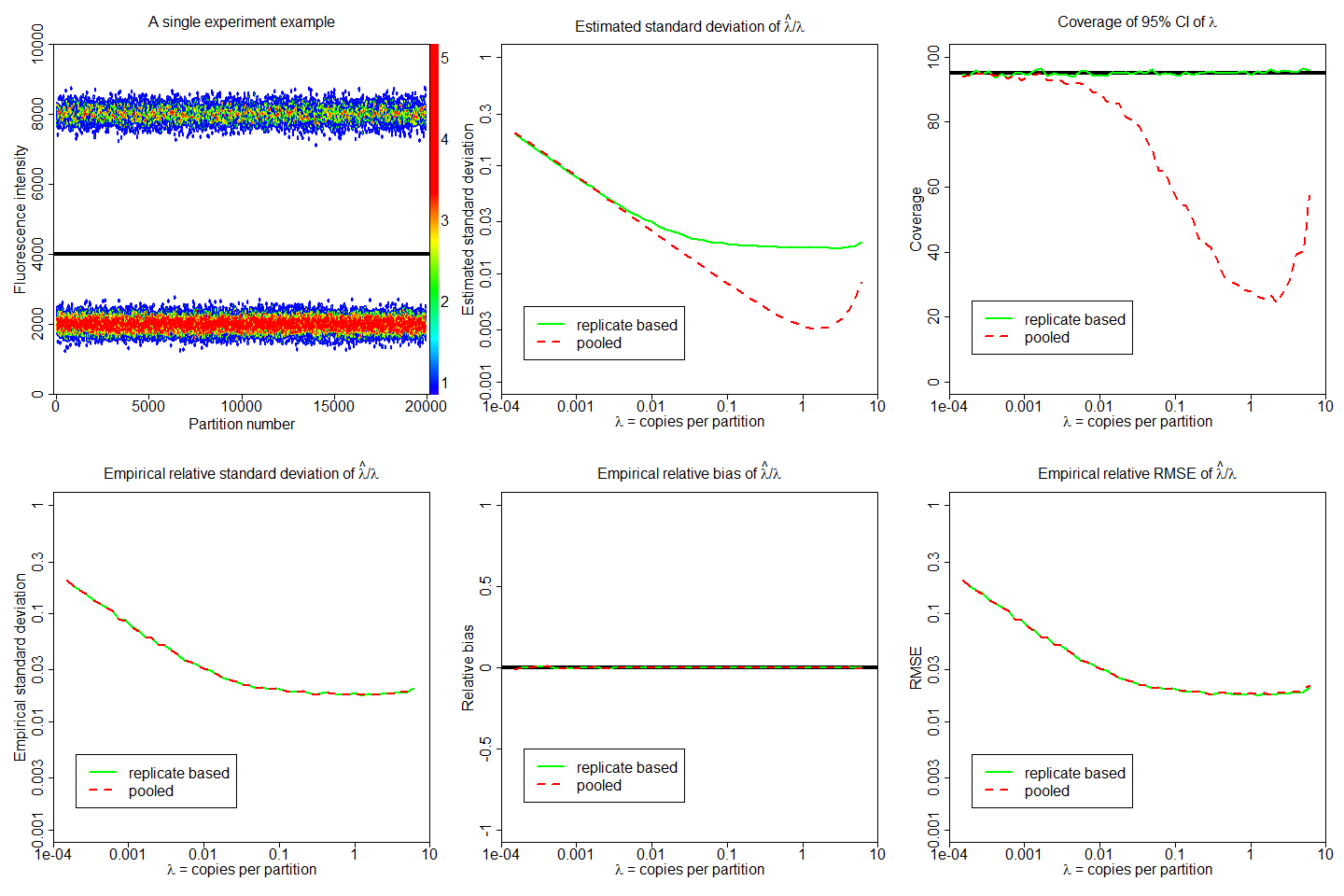

Supplement: Supplementary file 4 — Additional file 4: Interactive tool. In this mini-website, we provide an interactive tool to study the influence of specific sources of variation on the performance of the concentration estimators. This can serve as a guide when designing an experiment. All results are relative to the true concentration and based on 1000 simulations with 8 technical replicates. (ZIP 17 MB) [file 12859_2014_6687_MOESM4_ESM.zip › Additional file 4/RES/RES1451B.png]

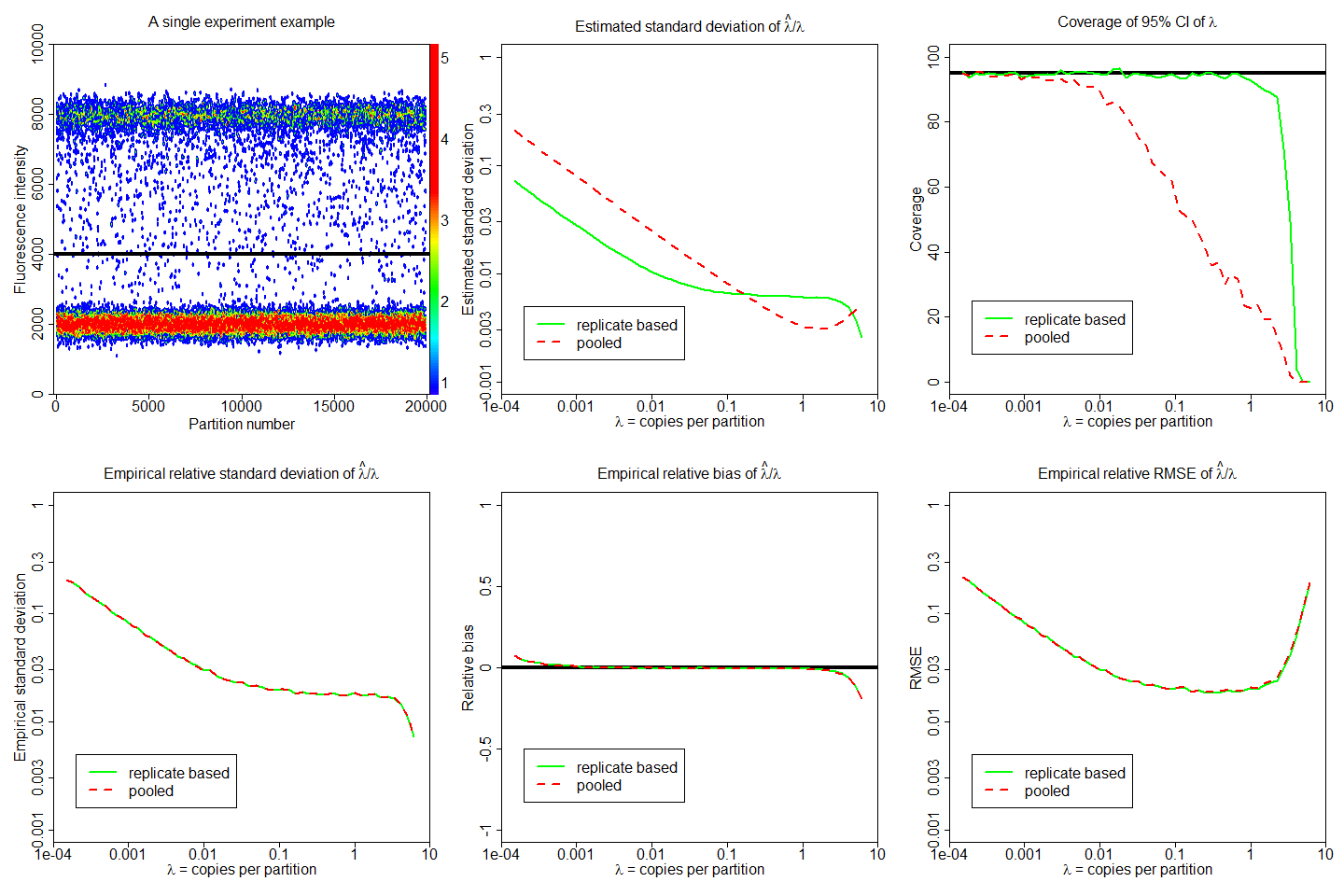

Supplement: Supplementary file 4 — Additional file 4: Interactive tool. In this mini-website, we provide an interactive tool to study the influence of specific sources of variation on the performance of the concentration estimators. This can serve as a guide when designing an experiment. All results are relative to the true concentration and based on 1000 simulations with 8 technical replicates. (ZIP 17 MB) [file 12859_2014_6687_MOESM4_ESM.zip › Additional file 4/RES/RES1452B.png]

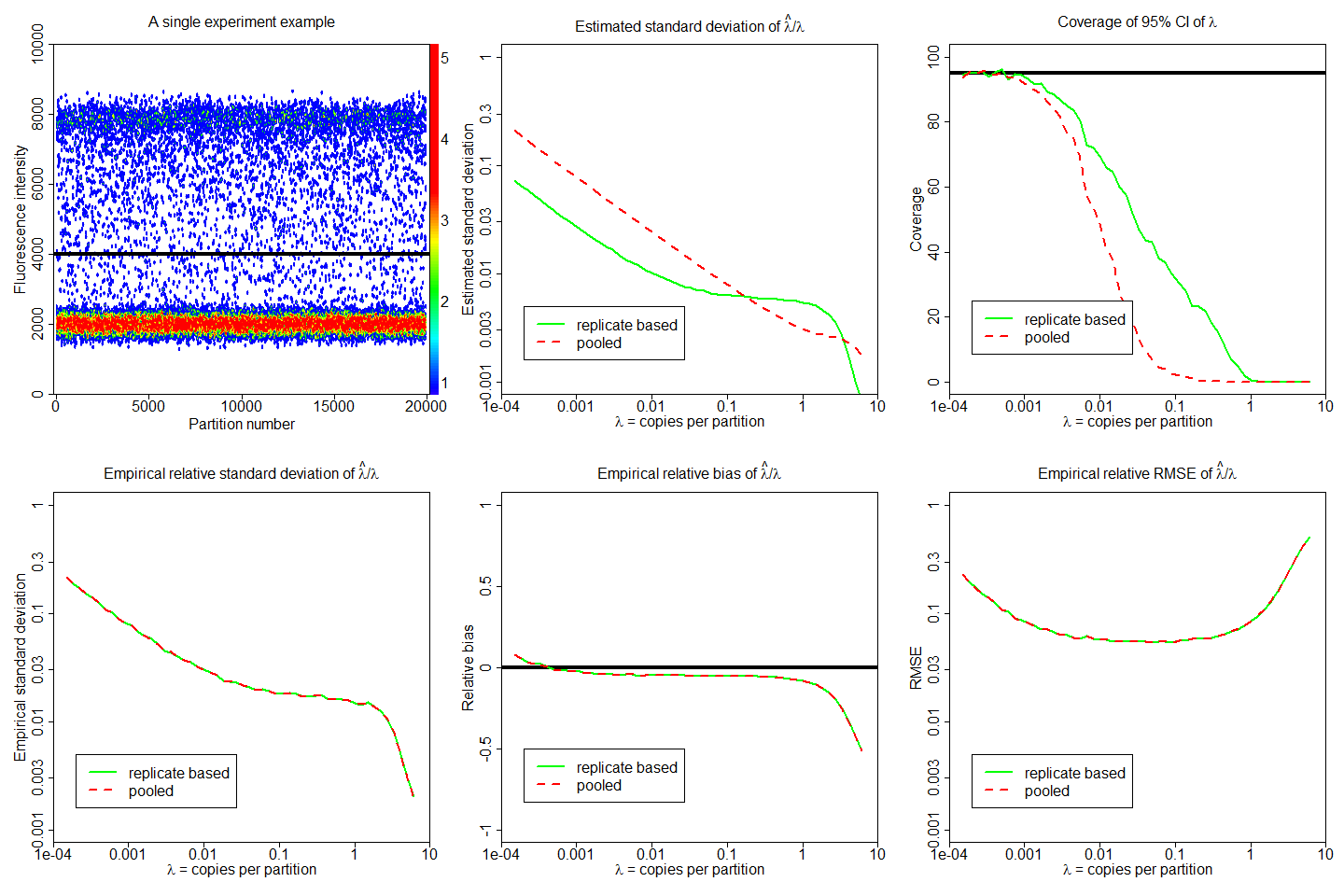

Supplement: Supplementary file 4 — Additional file 4: Interactive tool. In this mini-website, we provide an interactive tool to study the influence of specific sources of variation on the performance of the concentration estimators. This can serve as a guide when designing an experiment. All results are relative to the true concentration and based on 1000 simulations with 8 technical replicates. (ZIP 17 MB) [file 12859_2014_6687_MOESM4_ESM.zip › Additional file 4/RES/RES1453B.png]

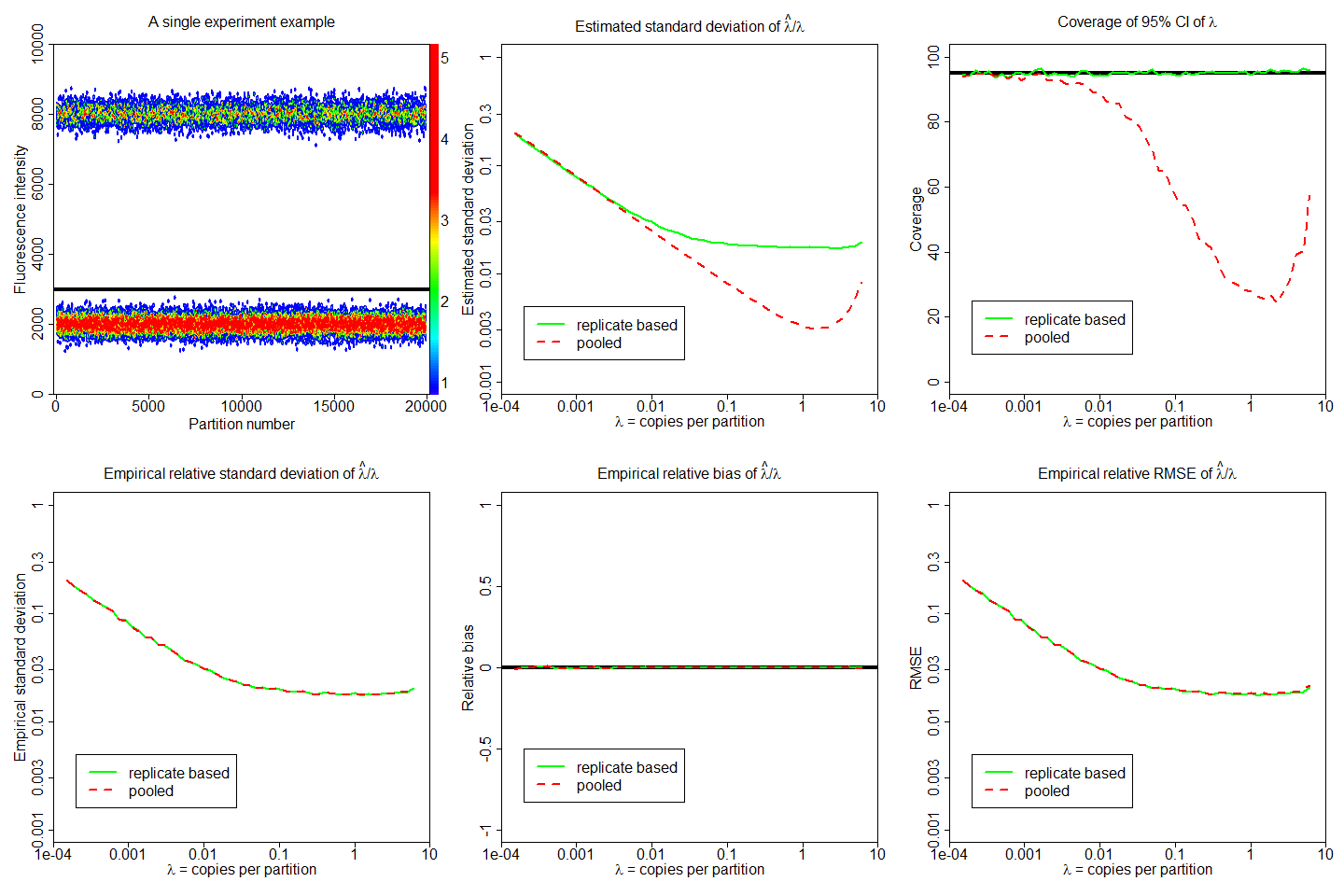

Supplement: Supplementary file 4 — Additional file 4: Interactive tool. In this mini-website, we provide an interactive tool to study the influence of specific sources of variation on the performance of the concentration estimators. This can serve as a guide when designing an experiment. All results are relative to the true concentration and based on 1000 simulations with 8 technical replicates. (ZIP 17 MB) [file 12859_2014_6687_MOESM4_ESM.zip › Additional file 4/RES/RES1461B.png]

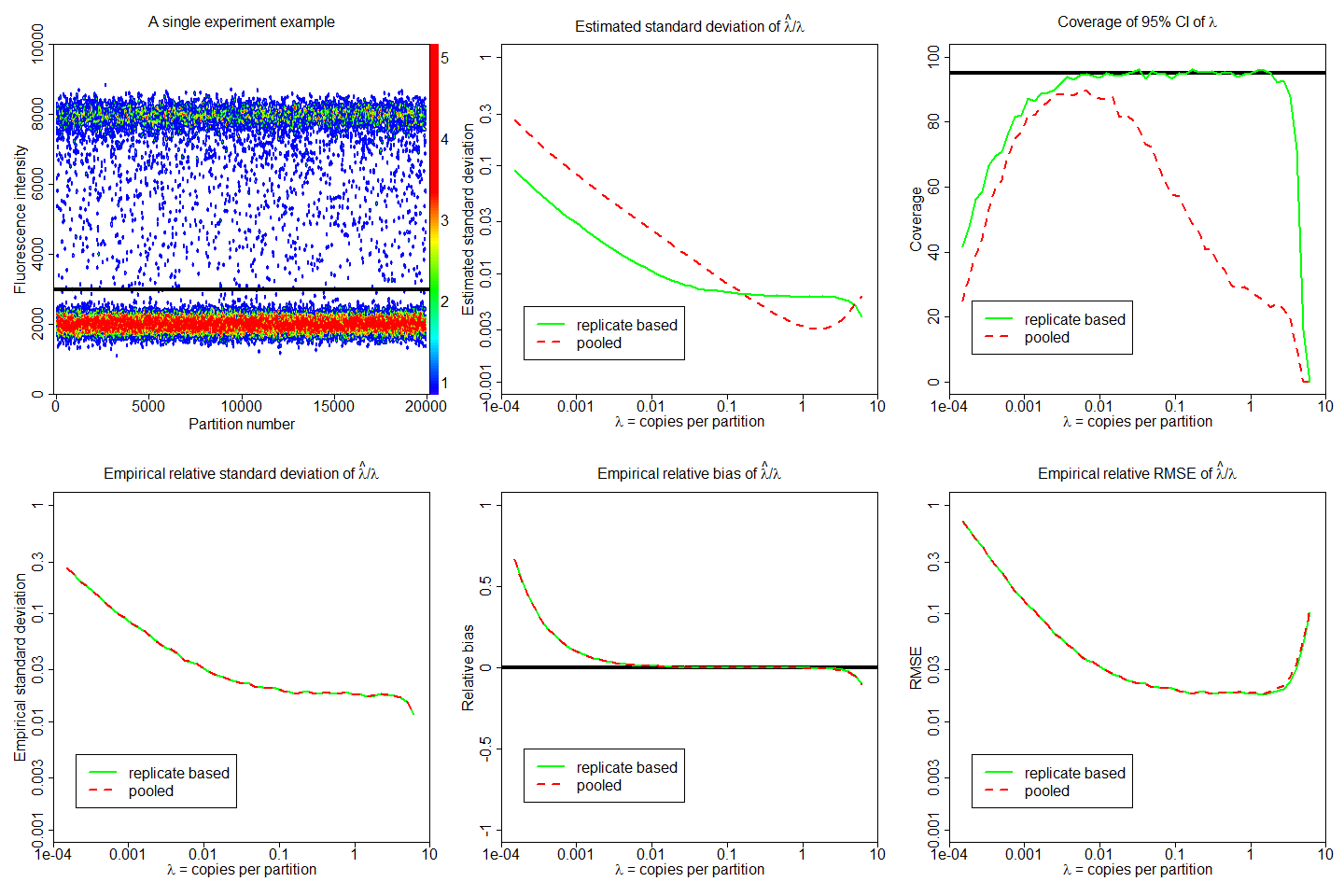

Supplement: Supplementary file 4 — Additional file 4: Interactive tool. In this mini-website, we provide an interactive tool to study the influence of specific sources of variation on the performance of the concentration estimators. This can serve as a guide when designing an experiment. All results are relative to the true concentration and based on 1000 simulations with 8 technical replicates. (ZIP 17 MB) [file 12859_2014_6687_MOESM4_ESM.zip › Additional file 4/RES/RES1462B.png]

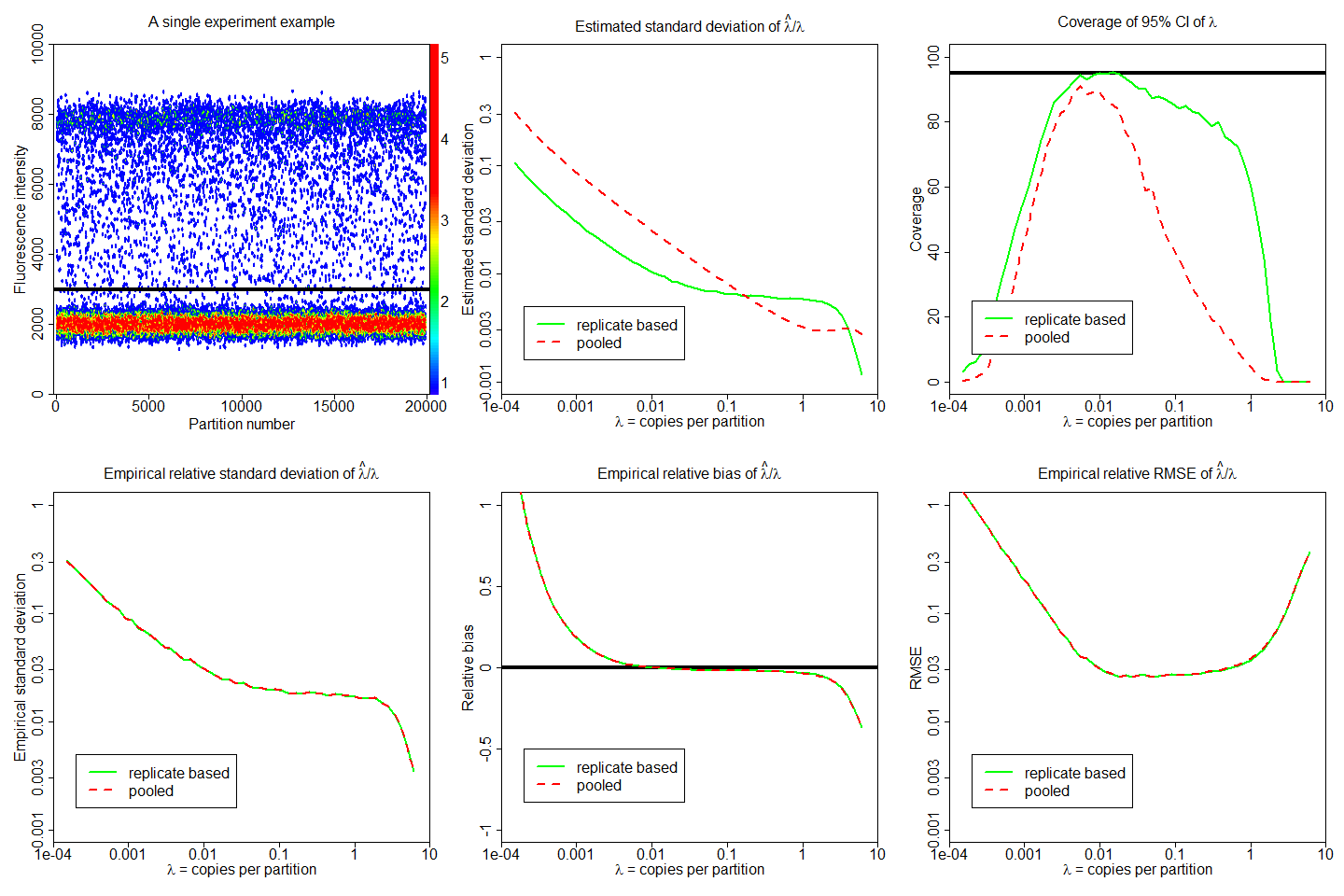

Supplement: Supplementary file 4 — Additional file 4: Interactive tool. In this mini-website, we provide an interactive tool to study the influence of specific sources of variation on the performance of the concentration estimators. This can serve as a guide when designing an experiment. All results are relative to the true concentration and based on 1000 simulations with 8 technical replicates. (ZIP 17 MB) [file 12859_2014_6687_MOESM4_ESM.zip › Additional file 4/RES/RES1463B.png]

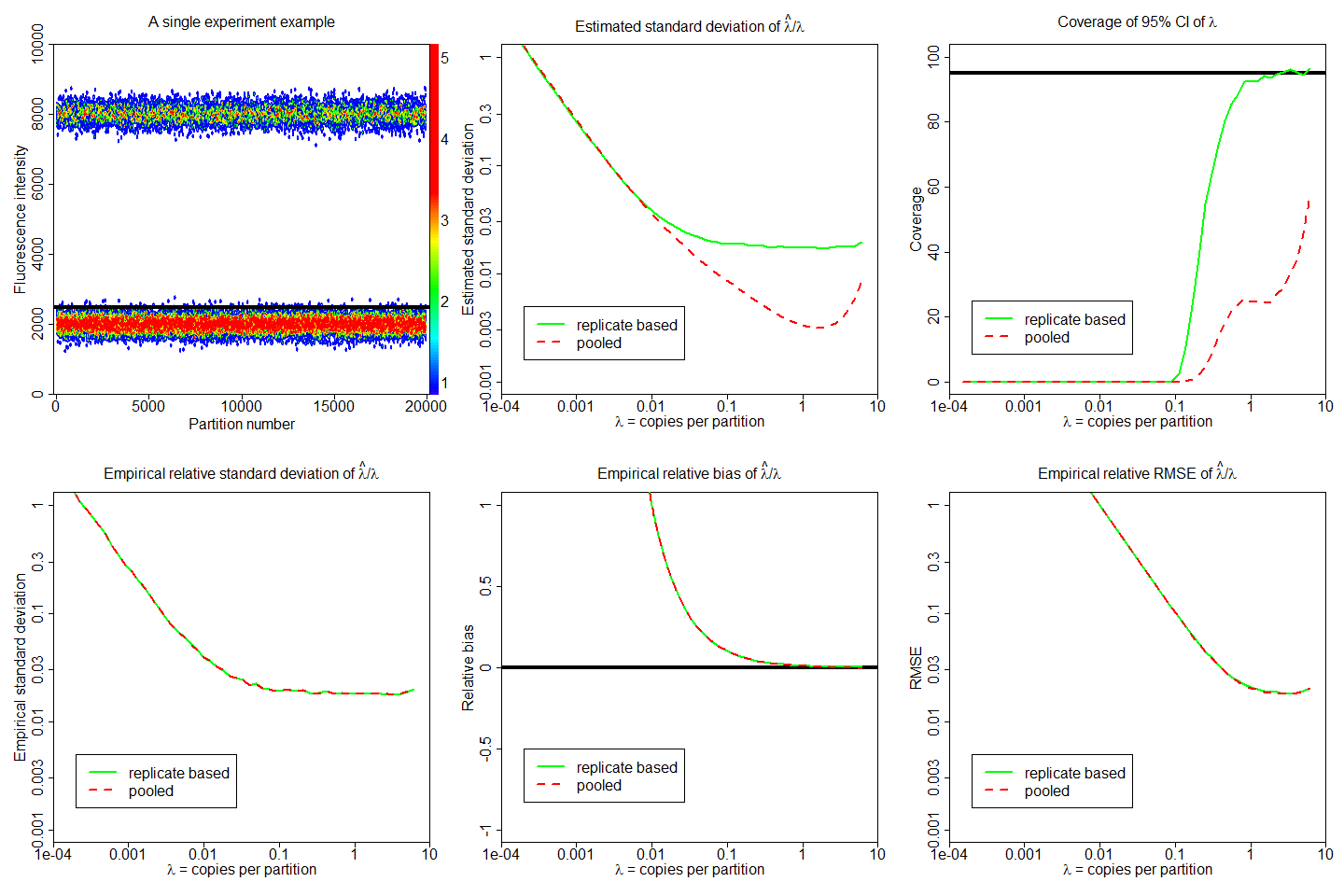

Supplement: Supplementary file 4 — Additional file 4: Interactive tool. In this mini-website, we provide an interactive tool to study the influence of specific sources of variation on the performance of the concentration estimators. This can serve as a guide when designing an experiment. All results are relative to the true concentration and based on 1000 simulations with 8 technical replicates. (ZIP 17 MB) [file 12859_2014_6687_MOESM4_ESM.zip › Additional file 4/RES/RES1471B.png]

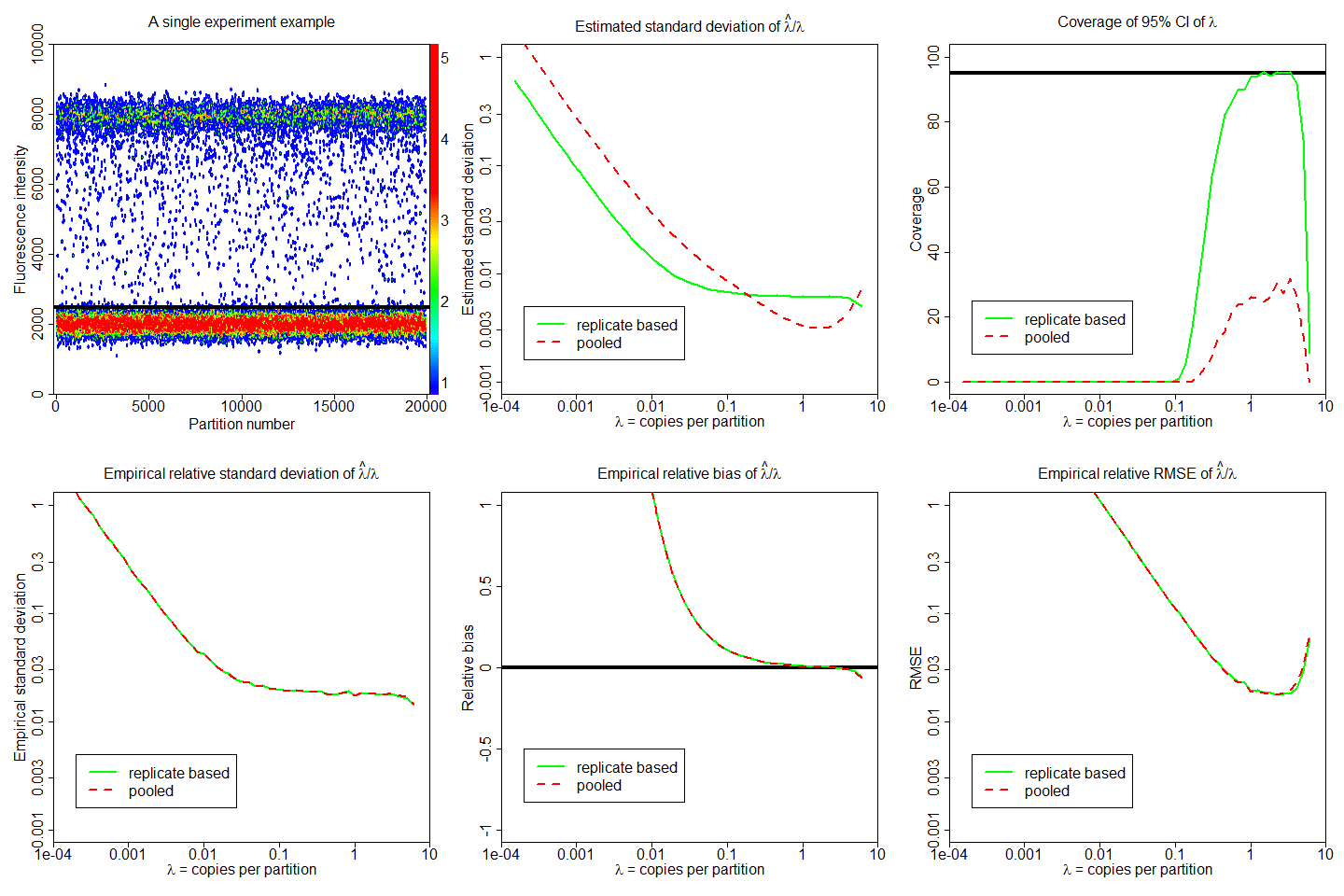

Supplement: Supplementary file 4 — Additional file 4: Interactive tool. In this mini-website, we provide an interactive tool to study the influence of specific sources of variation on the performance of the concentration estimators. This can serve as a guide when designing an experiment. All results are relative to the true concentration and based on 1000 simulations with 8 technical replicates. (ZIP 17 MB) [file 12859_2014_6687_MOESM4_ESM.zip › Additional file 4/RES/RES1472B.png]

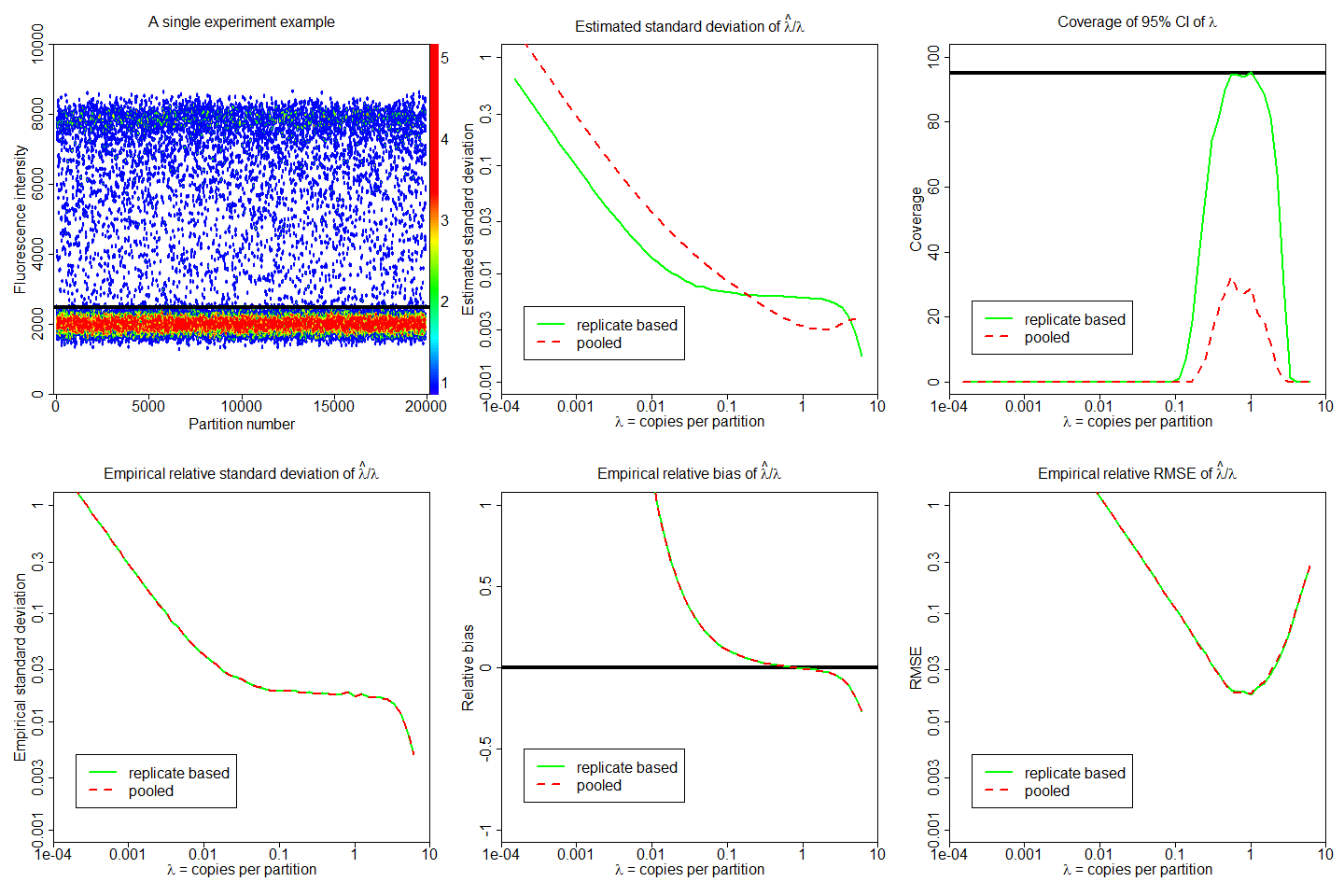

Supplement: Supplementary file 4 — Additional file 4: Interactive tool. In this mini-website, we provide an interactive tool to study the influence of specific sources of variation on the performance of the concentration estimators. This can serve as a guide when designing an experiment. All results are relative to the true concentration and based on 1000 simulations with 8 technical replicates. (ZIP 17 MB) [file 12859_2014_6687_MOESM4_ESM.zip › Additional file 4/RES/RES1473B.png]

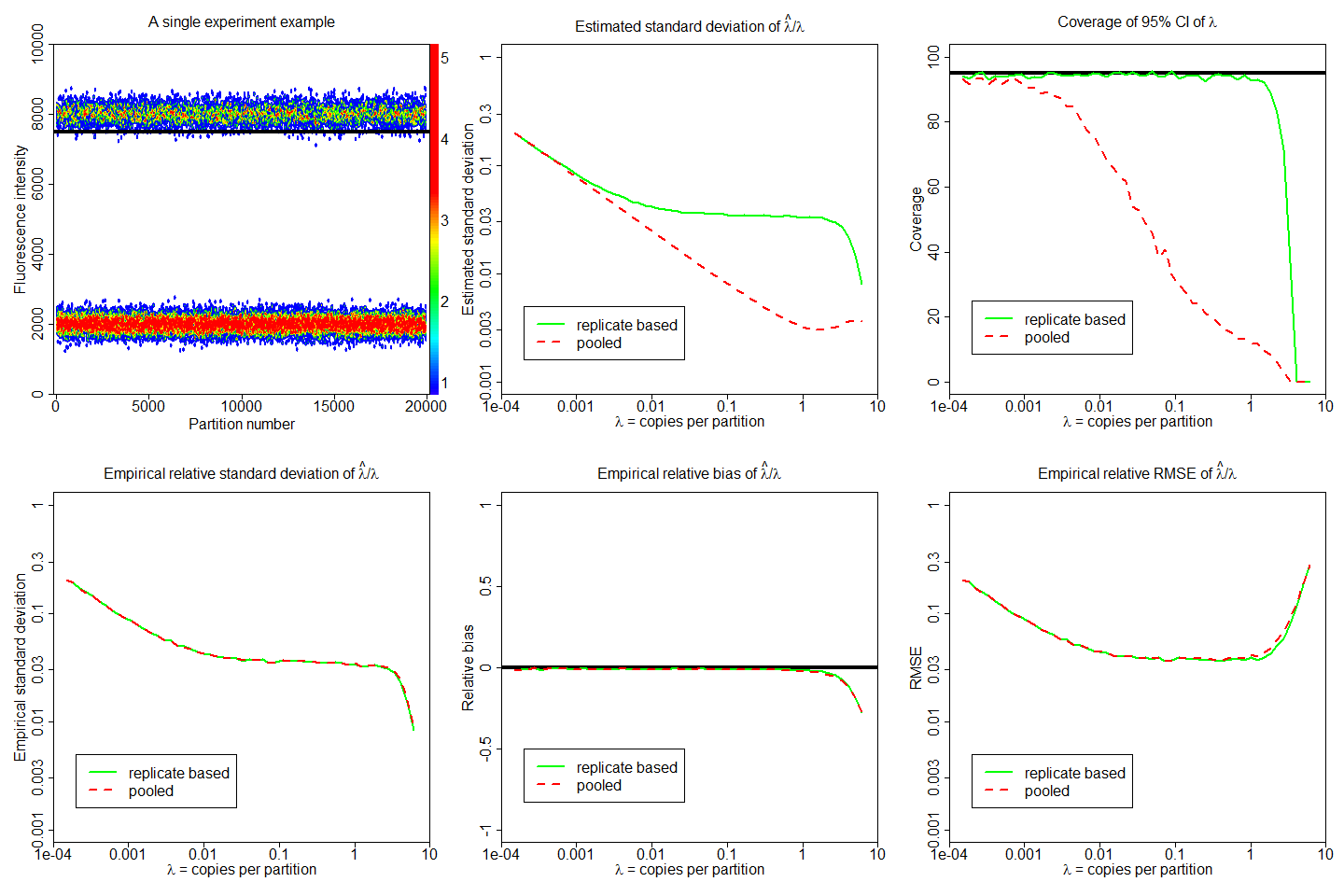

Supplement: Supplementary file 4 — Additional file 4: Interactive tool. In this mini-website, we provide an interactive tool to study the influence of specific sources of variation on the performance of the concentration estimators. This can serve as a guide when designing an experiment. All results are relative to the true concentration and based on 1000 simulations with 8 technical replicates. (ZIP 17 MB) [file 12859_2014_6687_MOESM4_ESM.zip › Additional file 4/RES/RES1511B.png]

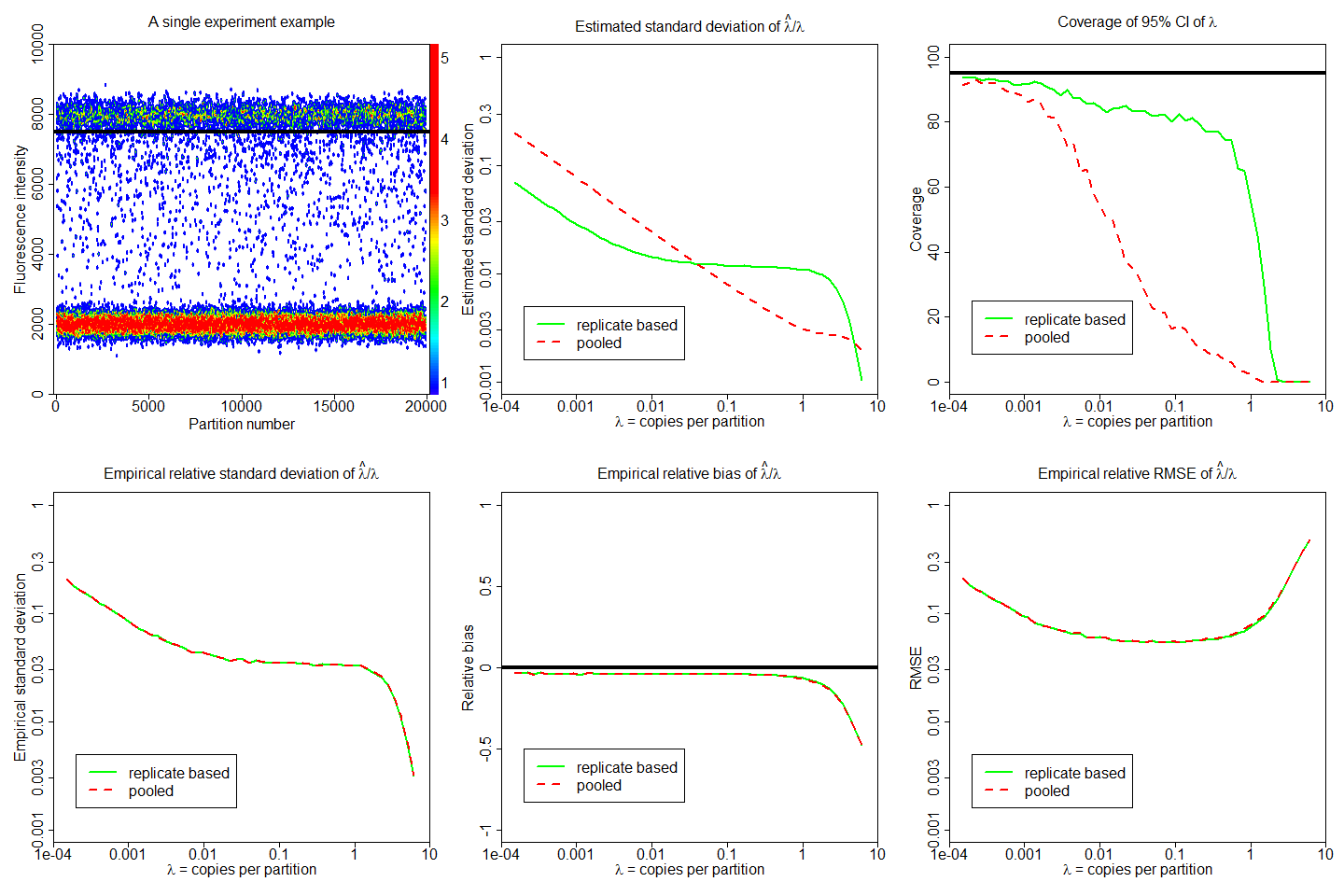

Supplement: Supplementary file 4 — Additional file 4: Interactive tool. In this mini-website, we provide an interactive tool to study the influence of specific sources of variation on the performance of the concentration estimators. This can serve as a guide when designing an experiment. All results are relative to the true concentration and based on 1000 simulations with 8 technical replicates. (ZIP 17 MB) [file 12859_2014_6687_MOESM4_ESM.zip › Additional file 4/RES/RES1512B.png]

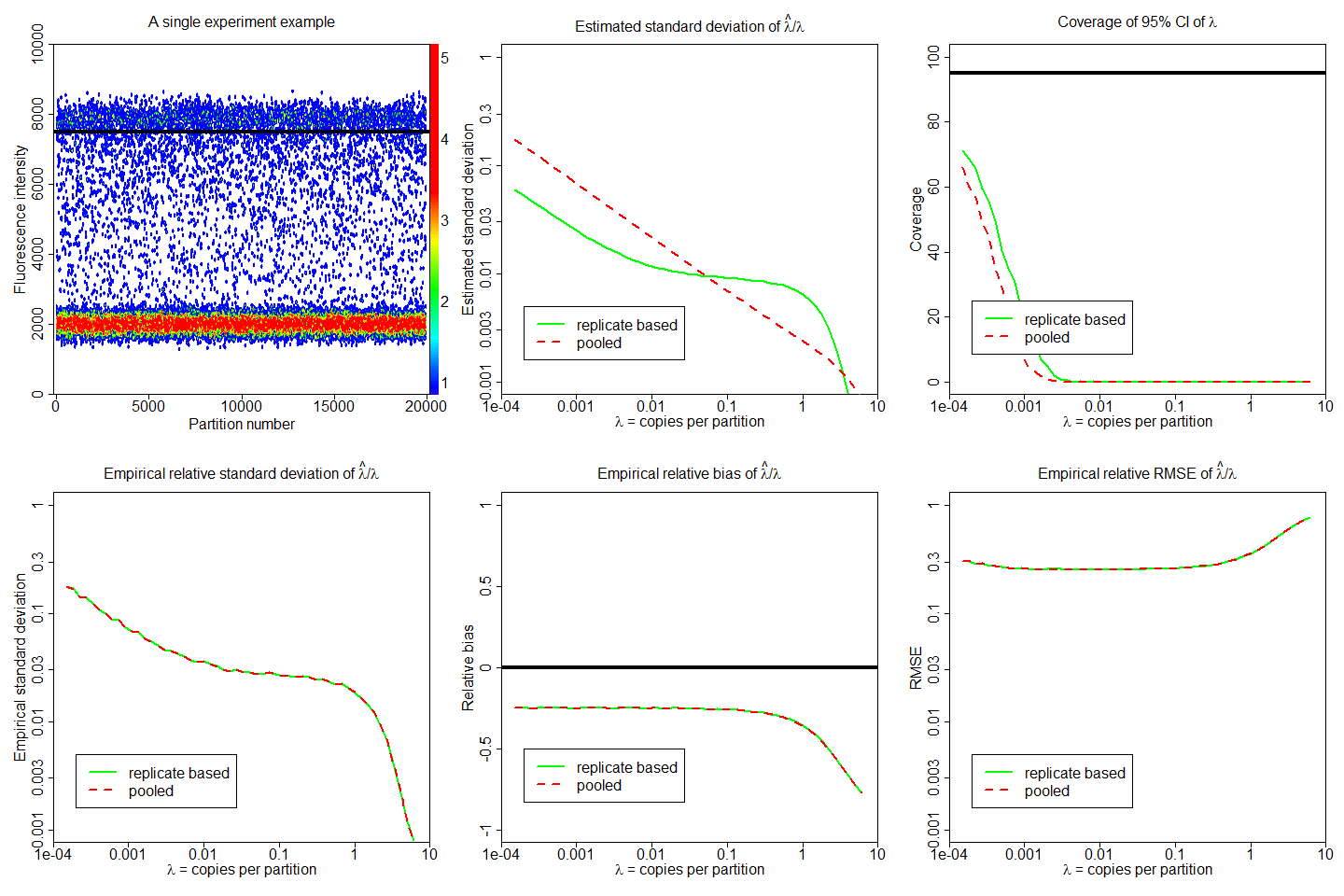

Supplement: Supplementary file 4 — Additional file 4: Interactive tool. In this mini-website, we provide an interactive tool to study the influence of specific sources of variation on the performance of the concentration estimators. This can serve as a guide when designing an experiment. All results are relative to the true concentration and based on 1000 simulations with 8 technical replicates. (ZIP 17 MB) [file 12859_2014_6687_MOESM4_ESM.zip › Additional file 4/RES/RES1513B.png]

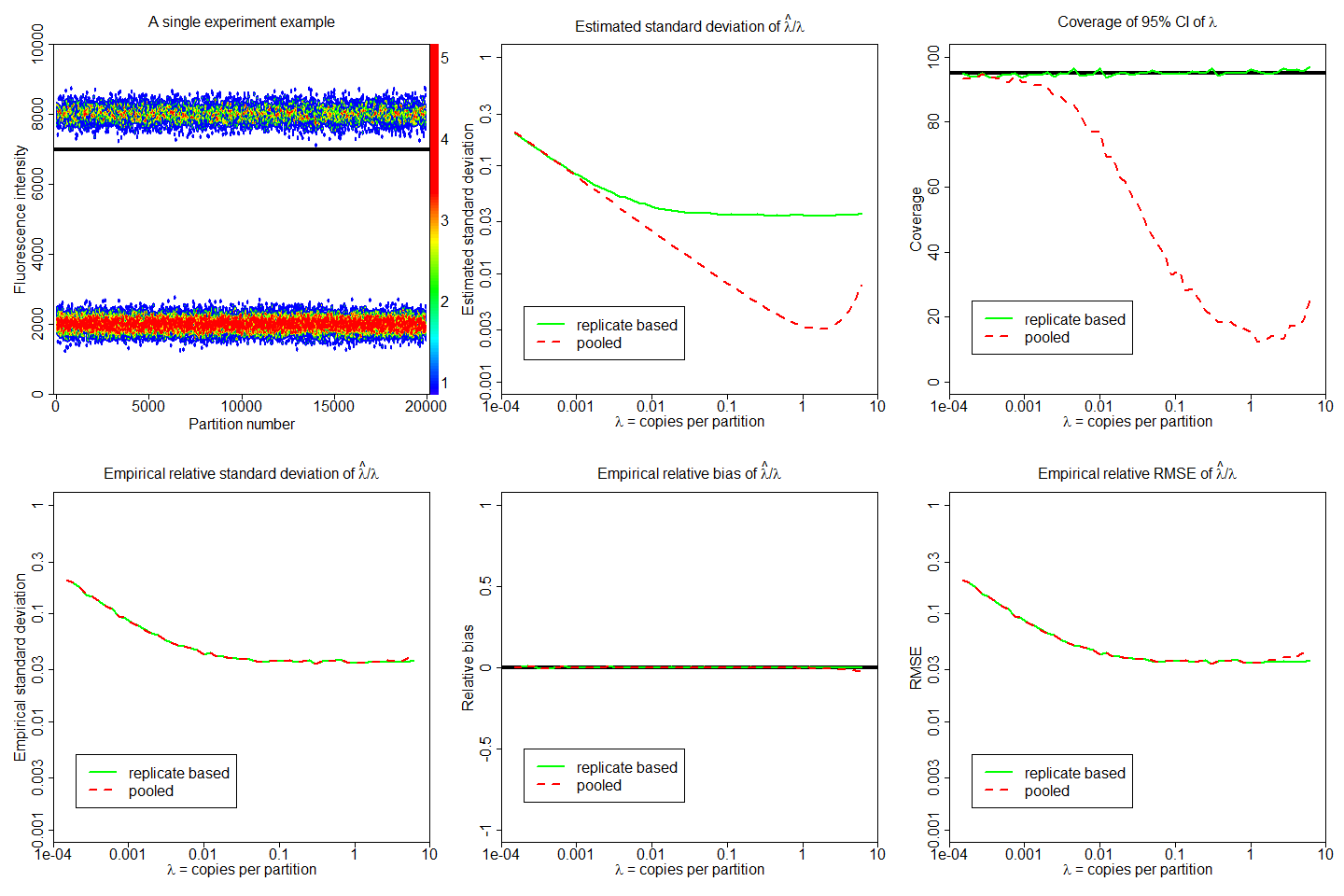

Supplement: Supplementary file 4 — Additional file 4: Interactive tool. In this mini-website, we provide an interactive tool to study the influence of specific sources of variation on the performance of the concentration estimators. This can serve as a guide when designing an experiment. All results are relative to the true concentration and based on 1000 simulations with 8 technical replicates. (ZIP 17 MB) [file 12859_2014_6687_MOESM4_ESM.zip › Additional file 4/RES/RES1521B.png]

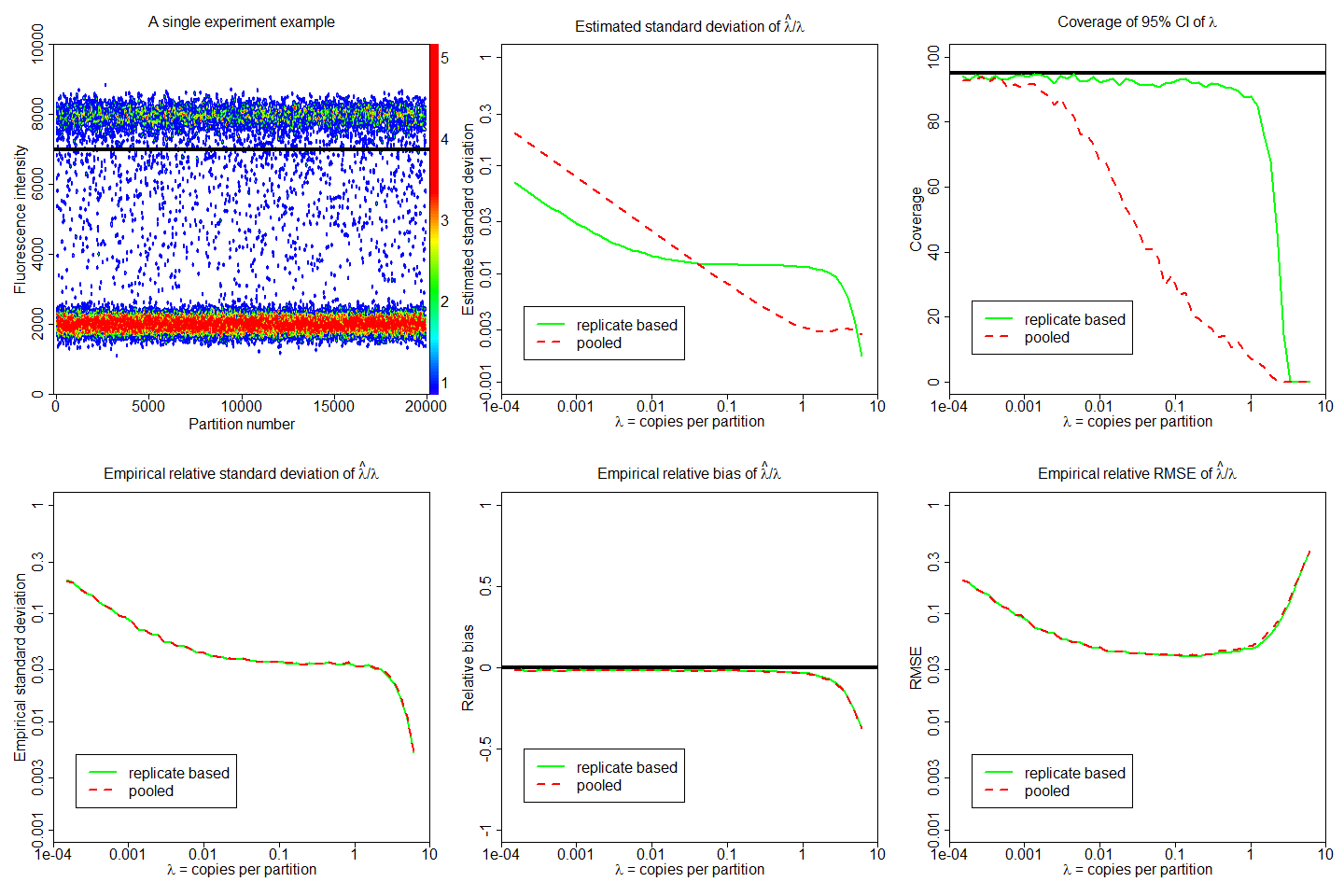

Supplement: Supplementary file 4 — Additional file 4: Interactive tool. In this mini-website, we provide an interactive tool to study the influence of specific sources of variation on the performance of the concentration estimators. This can serve as a guide when designing an experiment. All results are relative to the true concentration and based on 1000 simulations with 8 technical replicates. (ZIP 17 MB) [file 12859_2014_6687_MOESM4_ESM.zip › Additional file 4/RES/RES1522B.png]

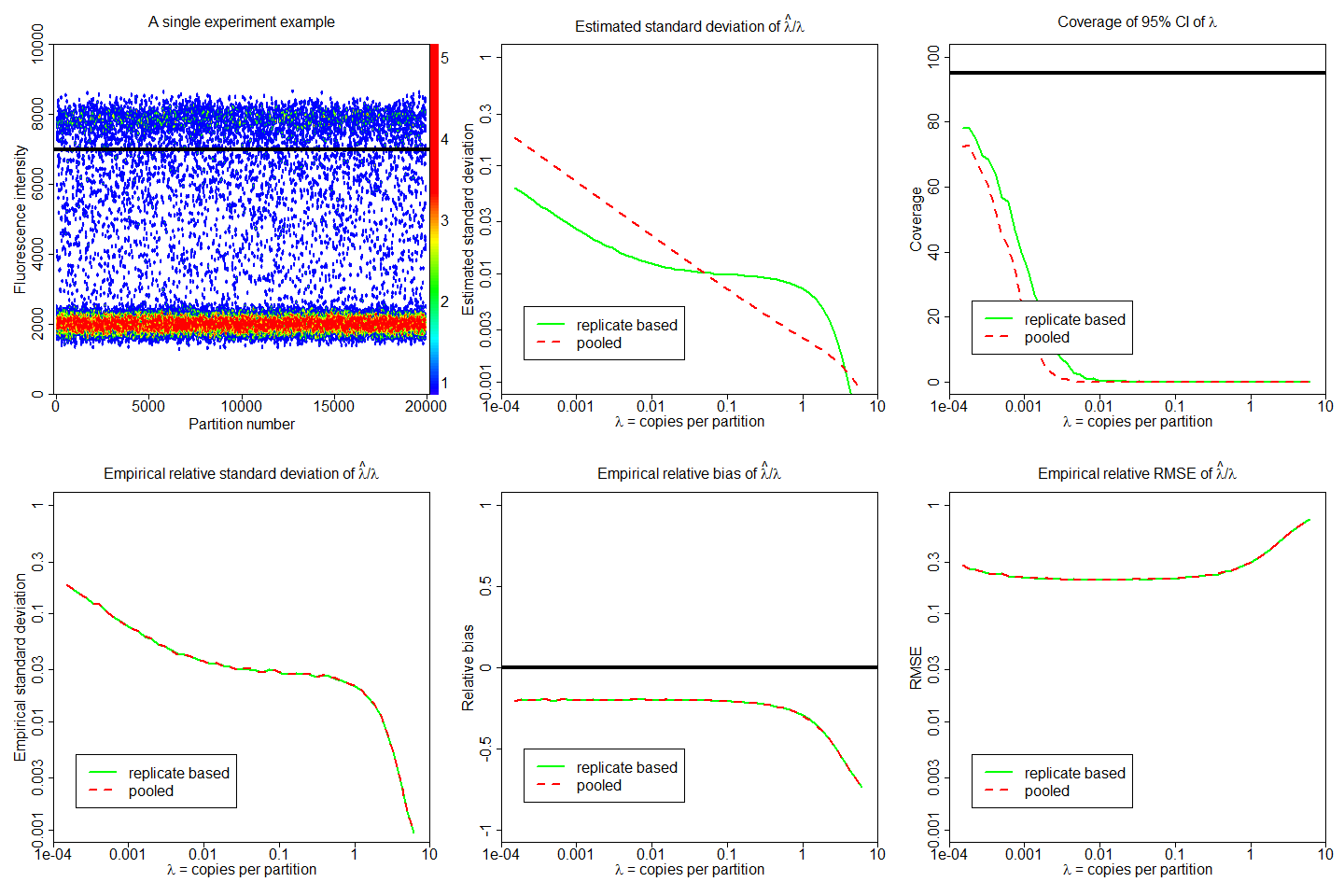

Supplement: Supplementary file 4 — Additional file 4: Interactive tool. In this mini-website, we provide an interactive tool to study the influence of specific sources of variation on the performance of the concentration estimators. This can serve as a guide when designing an experiment. All results are relative to the true concentration and based on 1000 simulations with 8 technical replicates. (ZIP 17 MB) [file 12859_2014_6687_MOESM4_ESM.zip › Additional file 4/RES/RES1523B.png]

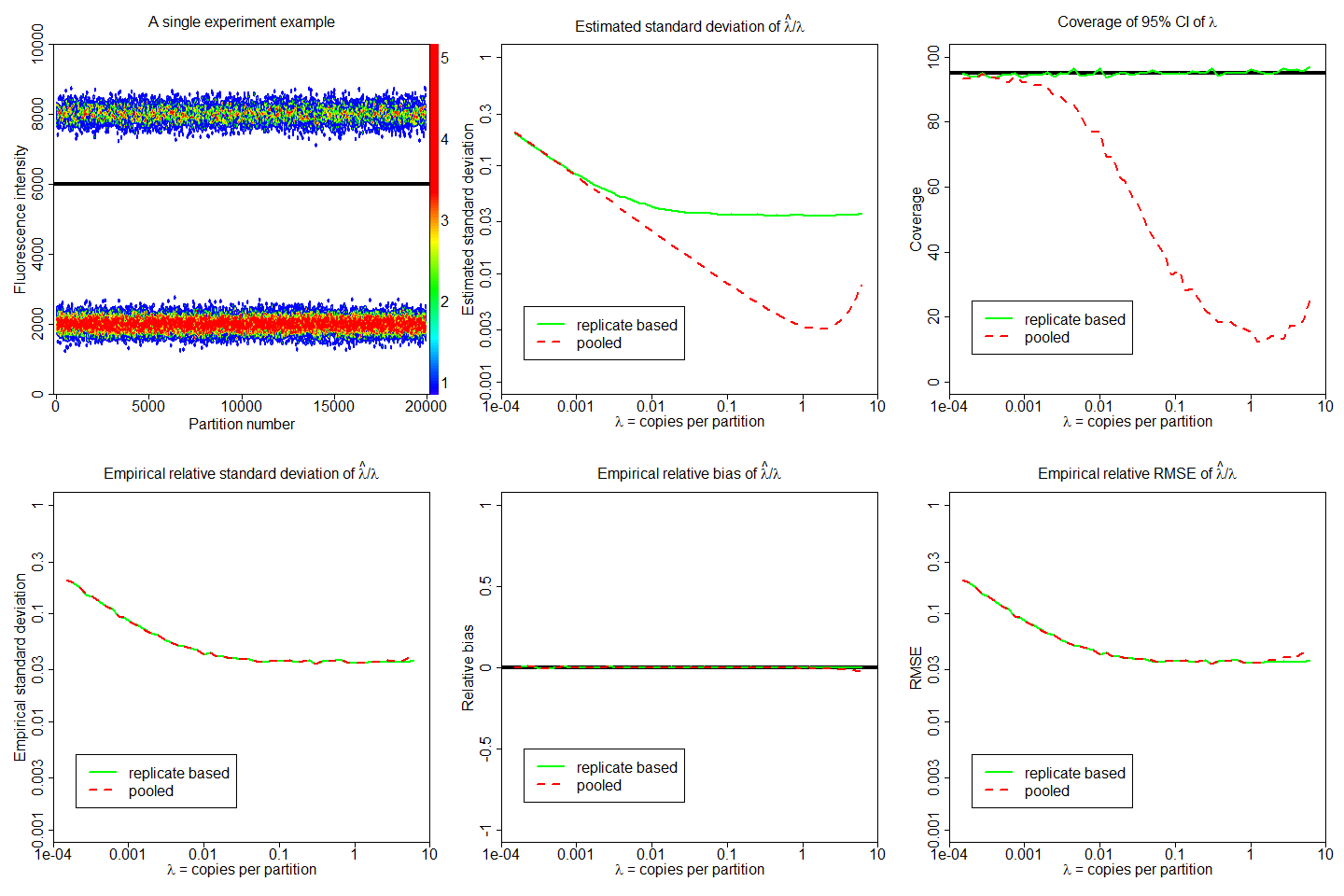

Supplement: Supplementary file 4 — Additional file 4: Interactive tool. In this mini-website, we provide an interactive tool to study the influence of specific sources of variation on the performance of the concentration estimators. This can serve as a guide when designing an experiment. All results are relative to the true concentration and based on 1000 simulations with 8 technical replicates. (ZIP 17 MB) [file 12859_2014_6687_MOESM4_ESM.zip › Additional file 4/RES/RES1531B.png]

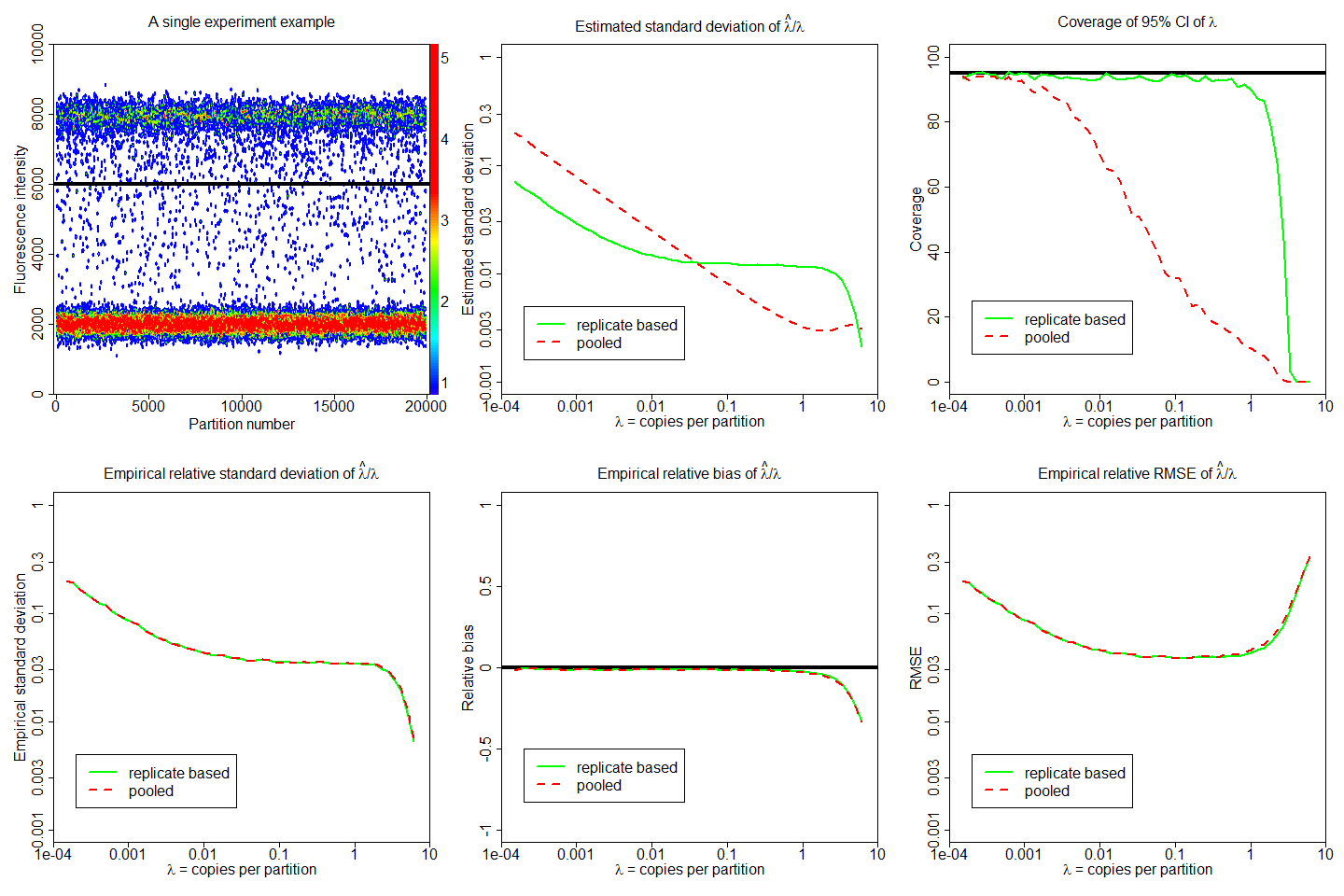

Supplement: Supplementary file 4 — Additional file 4: Interactive tool. In this mini-website, we provide an interactive tool to study the influence of specific sources of variation on the performance of the concentration estimators. This can serve as a guide when designing an experiment. All results are relative to the true concentration and based on 1000 simulations with 8 technical replicates. (ZIP 17 MB) [file 12859_2014_6687_MOESM4_ESM.zip › Additional file 4/RES/RES1532B.png]

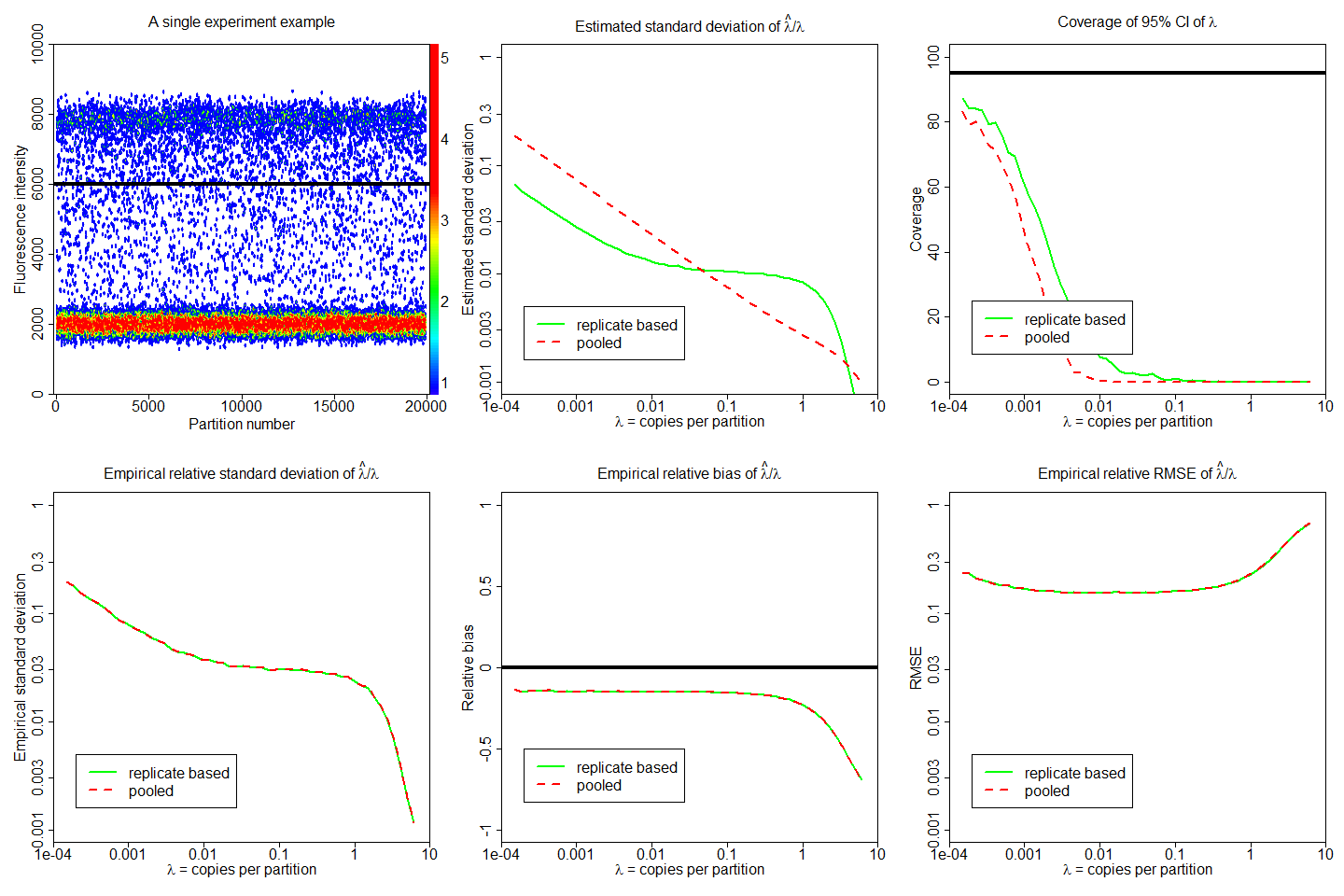

Supplement: Supplementary file 4 — Additional file 4: Interactive tool. In this mini-website, we provide an interactive tool to study the influence of specific sources of variation on the performance of the concentration estimators. This can serve as a guide when designing an experiment. All results are relative to the true concentration and based on 1000 simulations with 8 technical replicates. (ZIP 17 MB) [file 12859_2014_6687_MOESM4_ESM.zip › Additional file 4/RES/RES1533B.png]

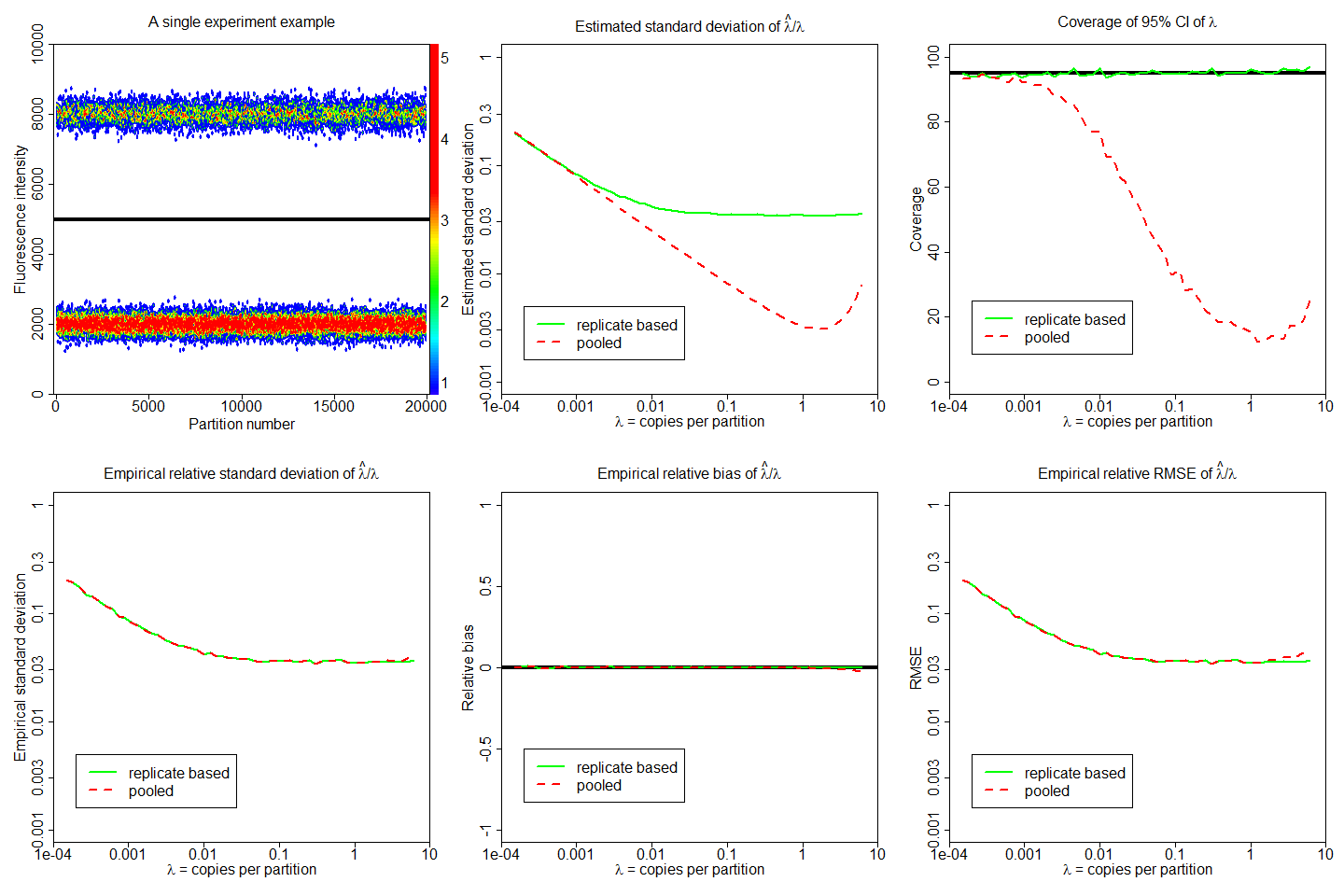

Supplement: Supplementary file 4 — Additional file 4: Interactive tool. In this mini-website, we provide an interactive tool to study the influence of specific sources of variation on the performance of the concentration estimators. This can serve as a guide when designing an experiment. All results are relative to the true concentration and based on 1000 simulations with 8 technical replicates. (ZIP 17 MB) [file 12859_2014_6687_MOESM4_ESM.zip › Additional file 4/RES/RES1541B.png]

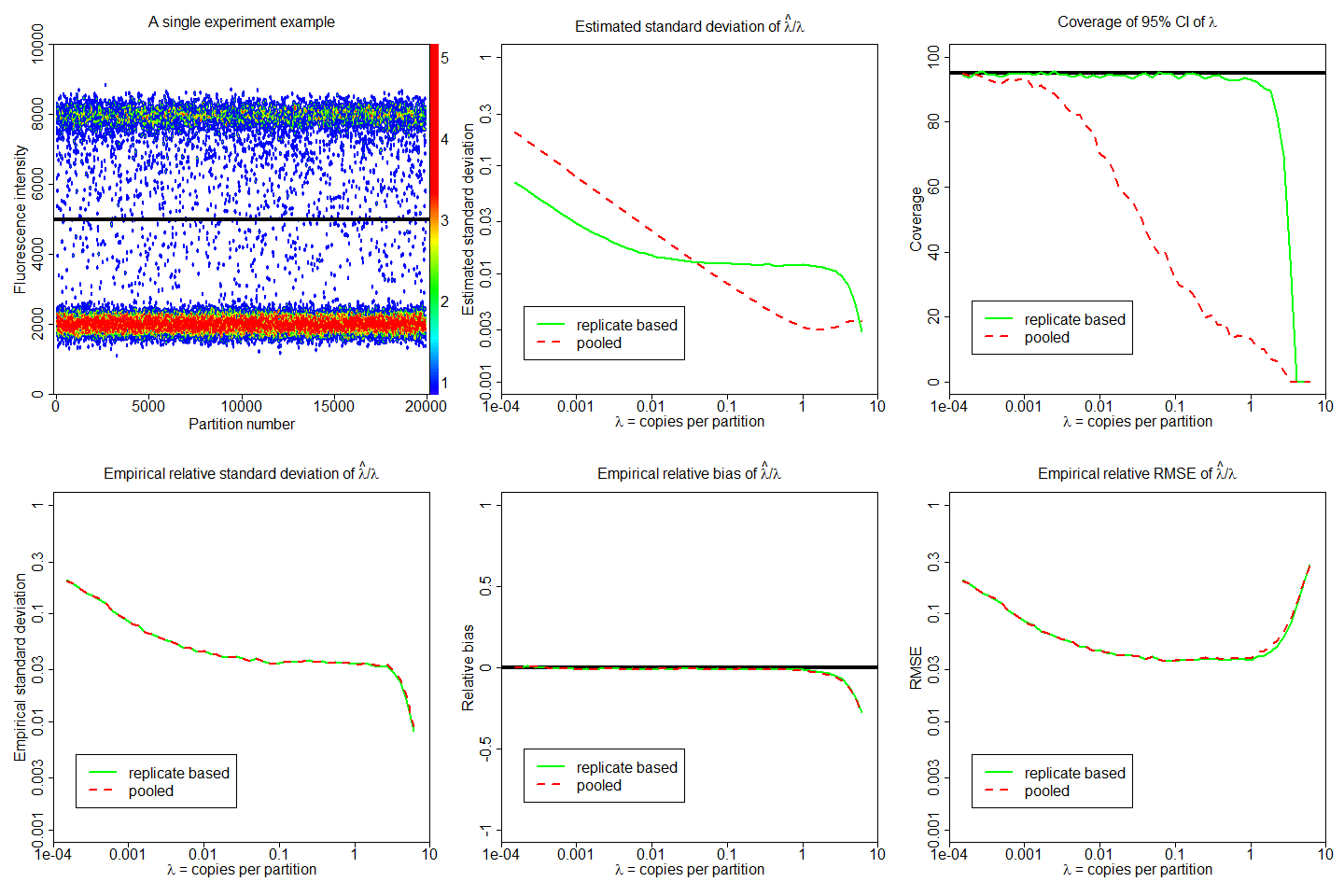

Supplement: Supplementary file 4 — Additional file 4: Interactive tool. In this mini-website, we provide an interactive tool to study the influence of specific sources of variation on the performance of the concentration estimators. This can serve as a guide when designing an experiment. All results are relative to the true concentration and based on 1000 simulations with 8 technical replicates. (ZIP 17 MB) [file 12859_2014_6687_MOESM4_ESM.zip › Additional file 4/RES/RES1542B.png]

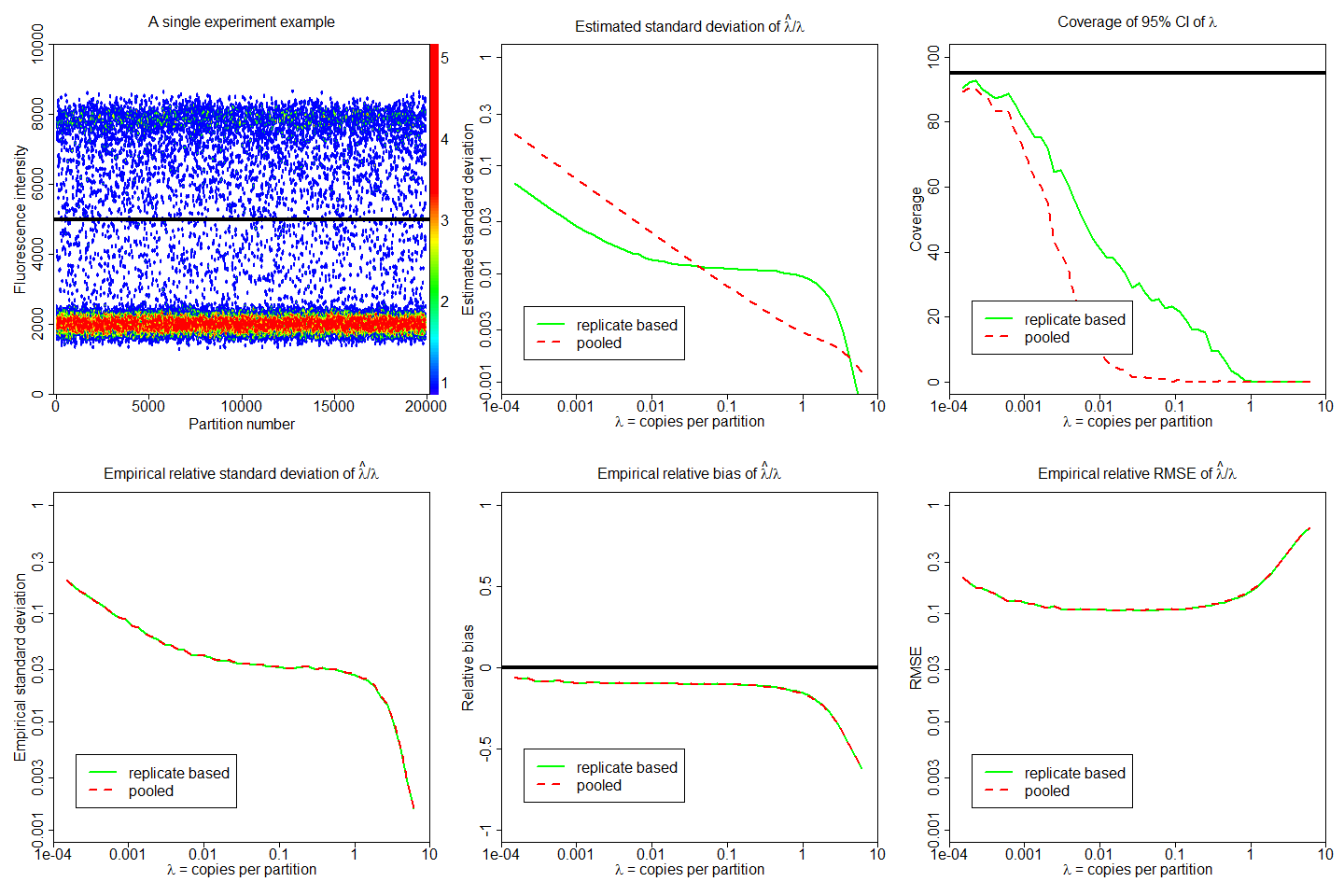

Supplement: Supplementary file 4 — Additional file 4: Interactive tool. In this mini-website, we provide an interactive tool to study the influence of specific sources of variation on the performance of the concentration estimators. This can serve as a guide when designing an experiment. All results are relative to the true concentration and based on 1000 simulations with 8 technical replicates. (ZIP 17 MB) [file 12859_2014_6687_MOESM4_ESM.zip › Additional file 4/RES/RES1543B.png]

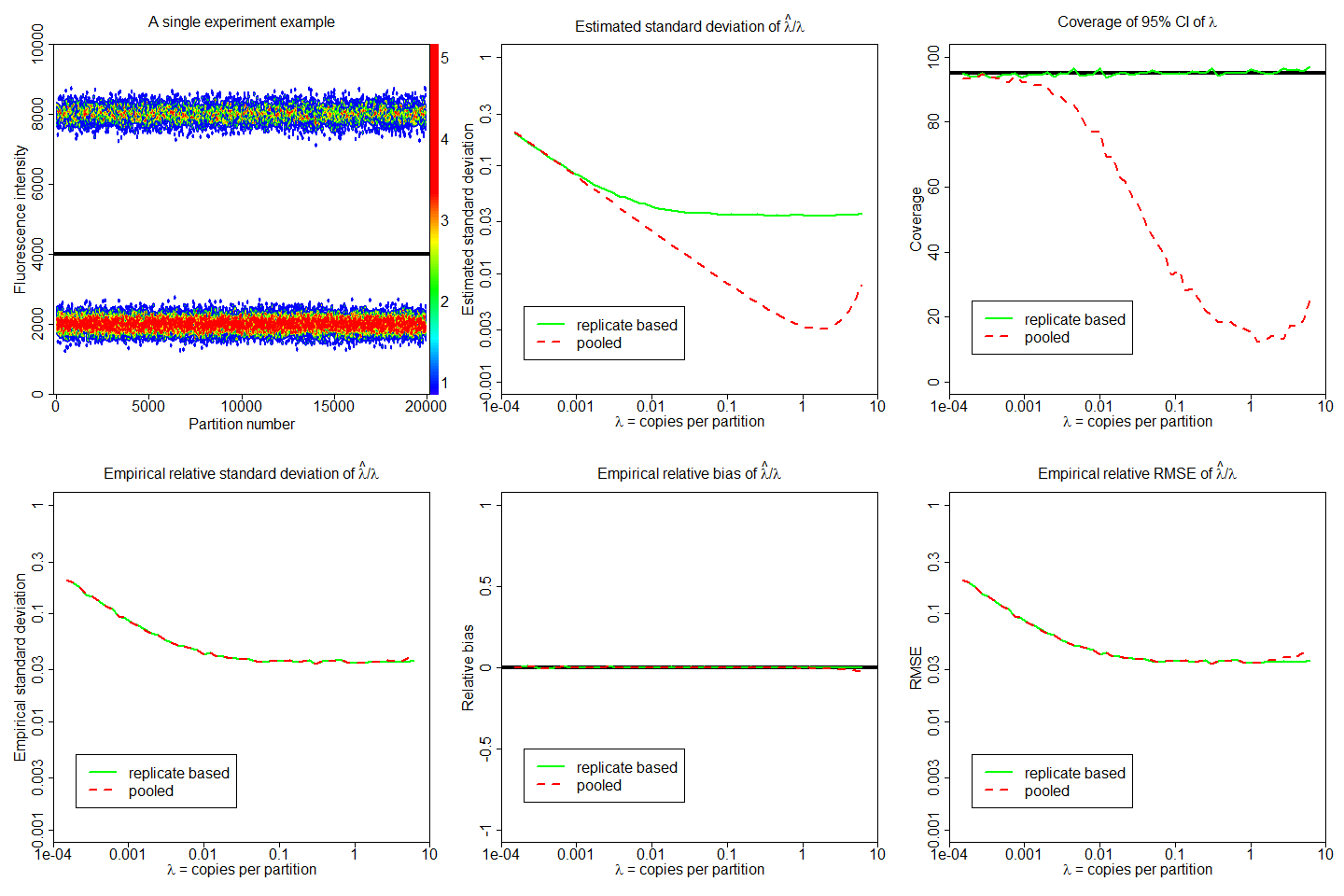

Supplement: Supplementary file 4 — Additional file 4: Interactive tool. In this mini-website, we provide an interactive tool to study the influence of specific sources of variation on the performance of the concentration estimators. This can serve as a guide when designing an experiment. All results are relative to the true concentration and based on 1000 simulations with 8 technical replicates. (ZIP 17 MB) [file 12859_2014_6687_MOESM4_ESM.zip › Additional file 4/RES/RES1551B.png]

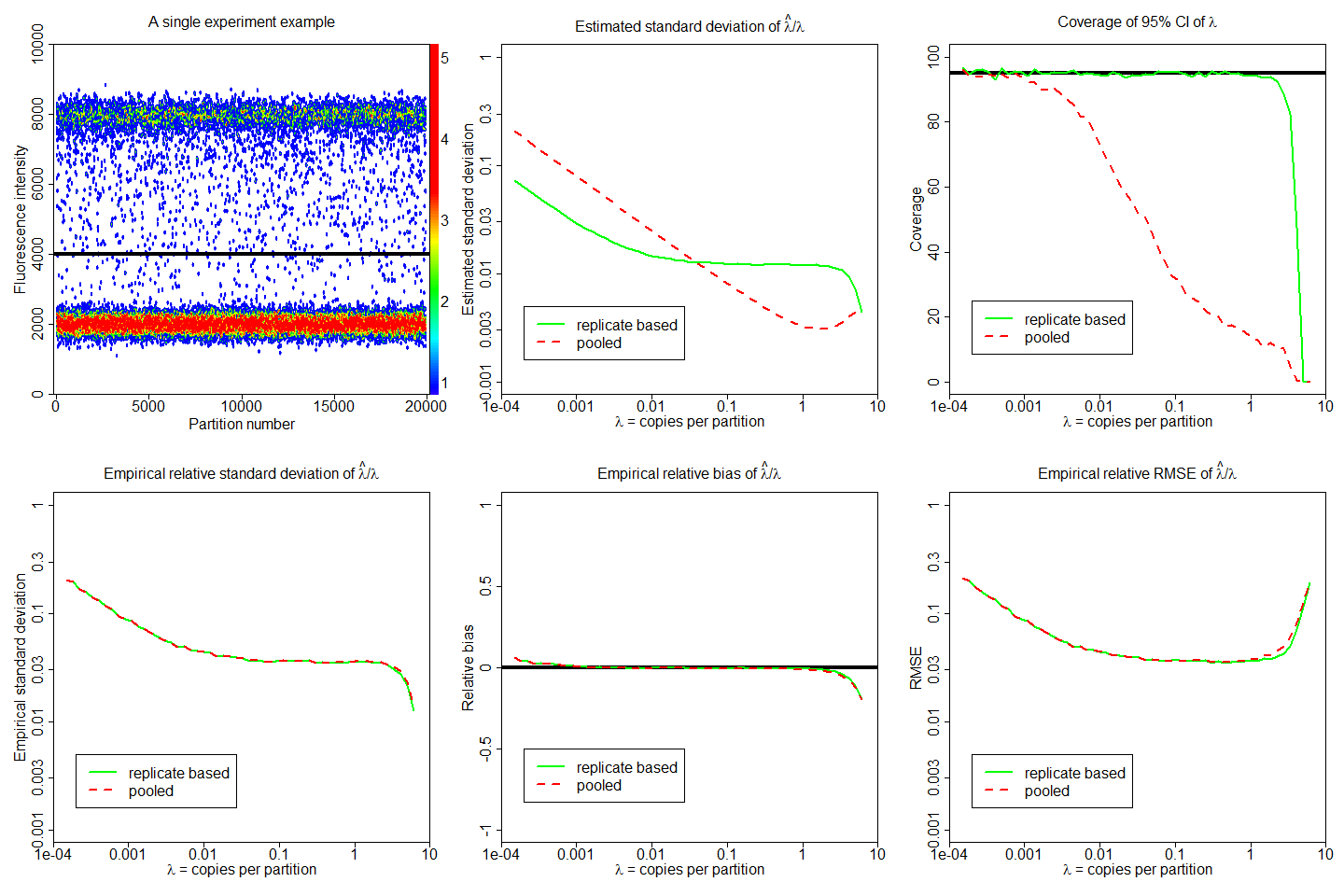

Supplement: Supplementary file 4 — Additional file 4: Interactive tool. In this mini-website, we provide an interactive tool to study the influence of specific sources of variation on the performance of the concentration estimators. This can serve as a guide when designing an experiment. All results are relative to the true concentration and based on 1000 simulations with 8 technical replicates. (ZIP 17 MB) [file 12859_2014_6687_MOESM4_ESM.zip › Additional file 4/RES/RES1552B.png]

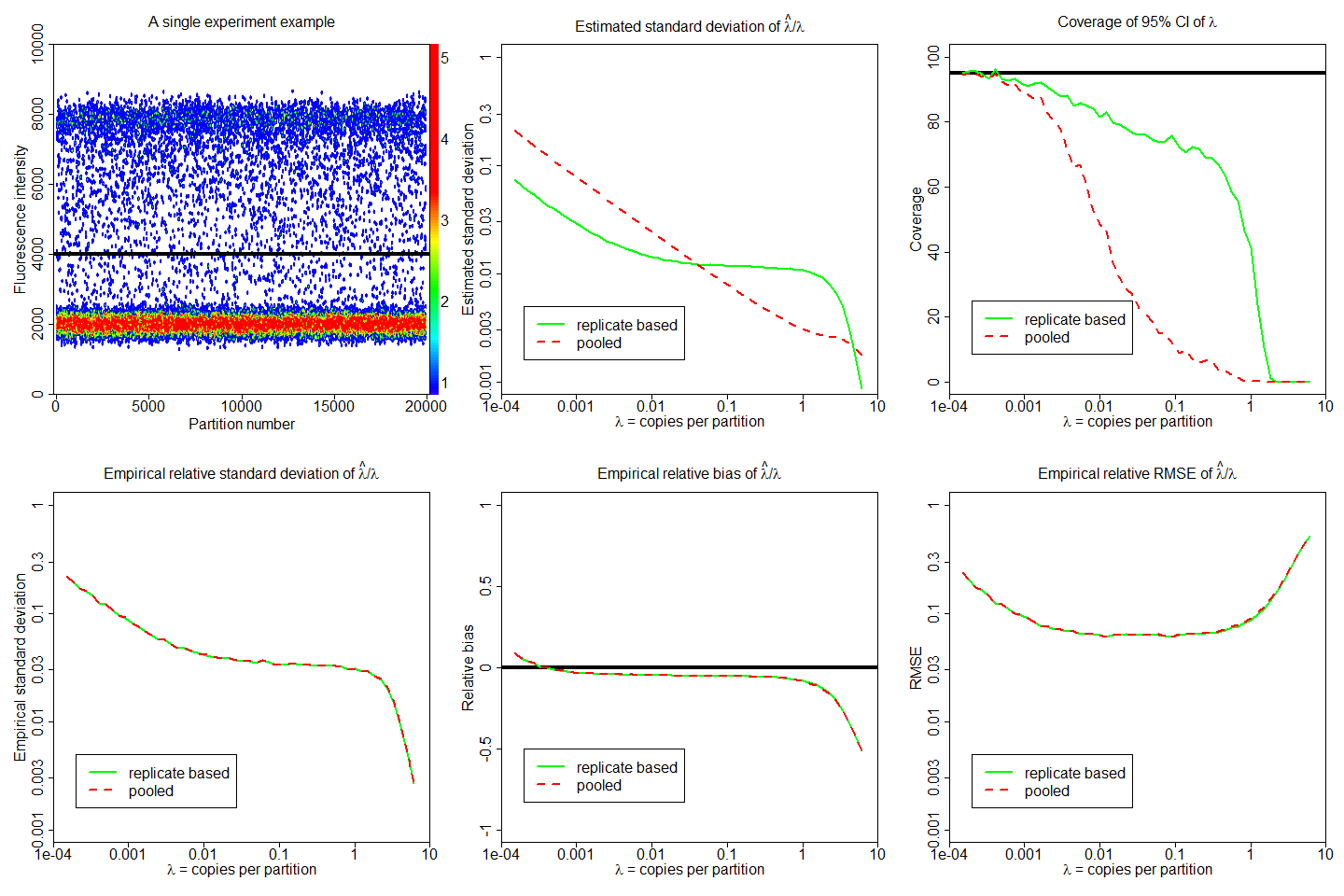

Supplement: Supplementary file 4 — Additional file 4: Interactive tool. In this mini-website, we provide an interactive tool to study the influence of specific sources of variation on the performance of the concentration estimators. This can serve as a guide when designing an experiment. All results are relative to the true concentration and based on 1000 simulations with 8 technical replicates. (ZIP 17 MB) [file 12859_2014_6687_MOESM4_ESM.zip › Additional file 4/RES/RES1553B.png]

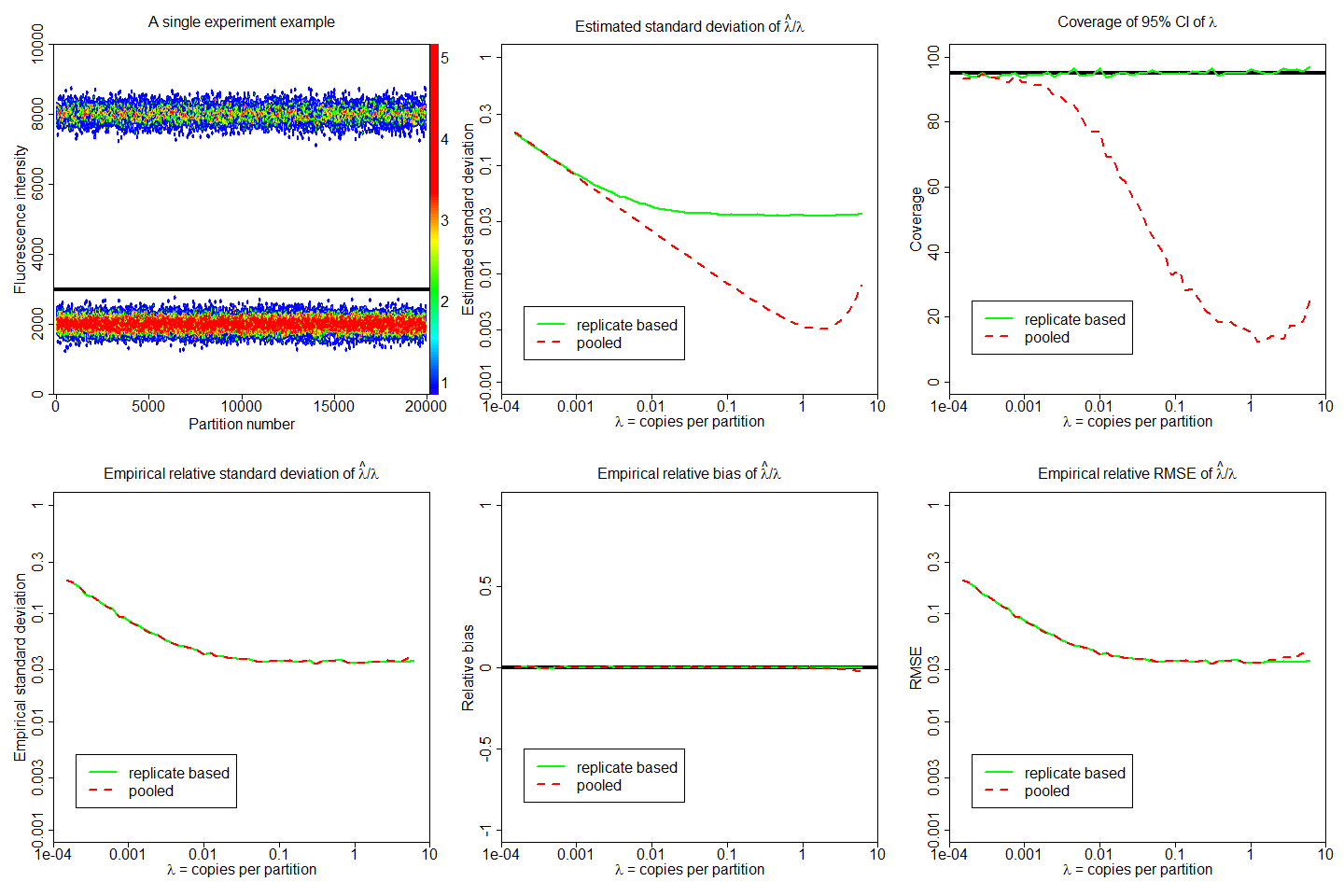

Supplement: Supplementary file 4 — Additional file 4: Interactive tool. In this mini-website, we provide an interactive tool to study the influence of specific sources of variation on the performance of the concentration estimators. This can serve as a guide when designing an experiment. All results are relative to the true concentration and based on 1000 simulations with 8 technical replicates. (ZIP 17 MB) [file 12859_2014_6687_MOESM4_ESM.zip › Additional file 4/RES/RES1561B.png]
